# Supplementary material for: Accelerating reliable multiscale quantum refinement of protein–drug systems enabled by machine learning
Source: Nat Commun. 2024 May 16;15:4181. doi: 10.1038/s41467-024-48453-4 (PMC11099068; doi:10.1038/s41467-024-48453-4)
Supplement: Supplementary file 1 — Supplementary Information [file 41467_2024_48453_MOESM1_ESM.pdf]

Supplementary information

Accelerating Reliable Multiscale Quantum Refinement of Protein–Drug Systems Enabled by Machine Learning

Zeyin Yan[1], Dacong Wei[1], Xin Li[1] & and Lung Wa Chung[1*]

[1]*Shenzhen Grubbs Institute, Department of Chemistry and Guangdong Provincial Key Laboratory of Catalysis, Southern University of Science and Technology, Shenzhen 518055, China*

List of Figures 2

List of Tables 29

1. General computational details 34

2. Result analysis 47

  2.1 Drugs/inhibitors in the gas phase 49

  2.2 Drugs/inhibitors in the proteins 65

List of Figures

Supplementary Figure 1: Chemical structures of drug/inhibitors (part 1). Chemical structures of our selected 27 drug/inhibitor molecules containing H, C, N, O elements in this study (QR50 dataset). Their drug name, related target protein name and PDB ID are included beneath these structures. 34

Supplementary Figure 2: Chemical structures of drug/inhibitors (part 1 continued). Chemical structures of our selected 27 drug/inhibitor molecules containing H, C, N, O elements in this study (QR50 dataset). Their drug name, related target protein name and PDB ID are included beneath these structures. 35

Supplementary Figure 3: Chemical structures of drug/inhibitors (part 2). Chemical structures of our selected 20 drug/inhibitor molecules containing H, C, N, O, F, S, and/or Cl elements in this study (QR50 dataset). Their drug name, related target protein name and PDB ID are included beneath these structures. Dashed lines represent the ONIOM(MLP-CC:MLP-DFT) boundary within the drug/inhibitor molecules containing F, Cl and/or S elements, when AIQM1 or ANI-1ccx was used. The region containing only C, H, O, N is described by AIQM1 or ANI-1ccx, where the rest containing F, Cl and/or S by ANI-2x. 36

Supplementary Figure 4: Chemical structures of drug/inhibitors (part 3). Chemical structures of our 3 selected drug/inhibitor molecules containing H, C, N, O, S, Br, and/or P elements in this study (QR50 dataset). Their drug name, related target protein name and PDB ID are included beneath these structures. Dashed lines represent the ONIOM2(MLP:SE) boundary within the drug/inhibitor molecules containing P and/or Br elements, when AIQM1 or ANI-2x was used. The region containing only C, H, O, N is described by AIQM1 or ANI-1ccx, where the rest containing P or Br by GFN2-xTB. 37

Supplementary Figure 5: Functional groups. The 40 most frequent functional groups occurring in bioactive molecules described reported and adapted from a recent paper.[1] The number indicates the percentage of molecules containing this functional group. R indicates aliphatic or aromatic carbon (Adapted with permission from Ref. 1. Copyright 2020 American Chemical Society.). 37

Supplementary Figure 6: Comparison of different density functional theory (DFT) methods in the gas phase. (**a**) Violin plot of derivations of the rotatable dihedrals (Δϕ, n = 259) of the selected 10 drug/inhibitor (shown in Fig. 1) (**b**) Supposition of rivaroxaban and CPI-0610 (shows outliers of violin plot) and (**c**) Violin plot of derivations of the rotatable dihedrals (Δϕ, n = 234) without rivaroxaban and CPI-0610 based on optimization in the gas phase using different functionals and basis sets compared to the DFT (ωB97X-D/6-31G(d)) method. Inner boxplots indicate interquartile range 47

Supplementary Figure 7 Comparison of the optimized structures in gas phase. Violin plot of derivations of the refined (**a**) bond distances (Δr), (**b**) angles (Δθ), (**c**) rotatable dihedrals (Δϕ) of the selected 10 drug/inhibitor (shown in Fig. 1) structures optimized in the gas phase (neutral and charged groups), using QDpi, AIQM1, ANI-1ccx, ANI-2x, ANI-1x and GFN2-xTB methods compared to the DFT (ωB97X-D/6-31G(d)) method. ONIOM2(MLP:ANI-2x) method was used for the molecules containing F, Cl and/or S elements, when AIQM1, QDpi, ANI-1ccx or ANI-1x was used. The number of data was marked in each plot. The white dots indicate the median values and inner boxplots indicate interquartile range. 49

Supplementary Figure 8: Optimized structures in the gas phase. Structure superimpositions of the selected 10 drugs/inhibitors (shown in Fig. 1) optimized in the gas phase using the AIQM1 (red), ANI-1ccx (pink), ANI-2x (blue), ANI-1x (yellow) and GFN2-xTB (green) and DFT (ωB97X-D/6-31G(d), black) methods. 53

Supplementary Figure 9: Optimized structures in the gas phase. Structure superimpositions of the selected 50 drugs/inhibitors (shown in Supplementary Figs. 1-4) optimized in the gas phase using the AIQM1 (red), ANI-2x (blue), and GFN2-xTB (green) and DFT (ωB97X-D/6-31G(d), black) methods. 54

Supplementary Figure 10: Chemical structures of some drug/inhibitors in PB20-QM-3k. Chemical structures of the drug/inhibitor molecules in PB20-QM-3k dataset containing elements beyond H, C, N, O elements in this study. Dashed lines represent the ONIOM boundary with the drug/inhibitor molecules containing F, Cl and/or S elements, when ONIOM2(MLP:ANI-2x) method was used for the 15 molecules containing F, Cl and/or S elements, when AIQM1 was used. ONIOM2(MLP:SE) method was used for the 16 molecules containing B, P, Se, Br and/or I elements, when AIQM1 or ANI-2x was used. 59

Supplementary Figure 11: Chemical structures of some drug/inhibitors in PB20-QM-3k). Chemical structures of the drug/inhibitor molecules in PB20-QM-3k dataset containing elements beyond H, C, N, O elements in this study. Dashed lines represent the ONIOM boundary with the drug/inhibitor molecules containing F, Cl and/or S elements, when ONIOM2(MLP:ANI-2x) method was used for the 15 molecules containing F, Cl and/or S elements, when AIQM1 was used. ONIOM2(MLP:SE) method was used for the 16 molecules containing B, P, Se, Br and/or I elements, when AIQM1 or ANI-2x was used. 60

Supplementary Figure 12: Comparison of the optimized structures in the gas phase (PB20-QM-3k). Violin plots of deviation in (**a**) bond distances (Δr), (**b**) angles ($\Delta\theta$) and (**c**) rotatable dihedrals ($\Delta\phi$) for the 3156 drug/inhibitor structures (PB20-QM-3k dataset) optimized in the gas phase, using MLPs (MLP-CC: AIQM1, MLP-DFT: ANI-2x) and the (SE) GFN2-xTB method compared to the (QM) ωB97X-D/6-31G(d) method. ONIOM2(MLP:ANI-2x) method was used for the 15 molecules containing F, Cl and/or S elements, when AIQM1 was used. ONIOM2(MLP:SE) method was used for the 16 molecules containing B, P, Se, Br and/or I elements, when AIQM1 or ANI-2x was used. The white dots in violin plots indicate the median values and inner boxplots indicate interquartile range. The number of data was marked in each plot. 61

Supplementary Figure 13: Comparison of the optimized structures in the gas phase (PB20-QM-8k). Violin plots of deviation in (**a**) bond distances (Δr), (**b**) angles ($\Delta\theta$) and (**c**) rotatable dihedrals ($\Delta\phi$) for the 8776 drug/inhibitor structures (PB20-QM-8k dataset) optimized in the gas phase, using ANI-2x and the (SE) GFN2-xTB method compared to the (QM) ωB97X-D/6-31G(d) method. The white dots in violin plots indicate the median values and inner boxplots indicate interquartile range. The number of data was marked in each plot. 63

Supplementary Figure 14: Comparison of quantum refinement results (10 systems). Box plots of deviation of (**a**) real-space Z-difference (RSZD) scores as well as (**b**) strain energy ($\Delta\Delta E$, kcal·mol^{-1}), boxplots indicate median values, interquartile range, minimum and maximum value, and individual data points. and violin plots of the deviations (**c**) bond distances (Δr), (**d**) angles ($\Delta\theta$) and (**e**) rotatable dihedrals ($\Delta\phi$) of drugs/inhibitors in the selected 10 (shown in Fig. 1b) protein–drug/inhibitor systems after **M1-M10** quantum refinement approaches compared to **M7** ONIOM3(DFT:SE:MM)). The X-ray results were taken from the experimental structures without further refinement. The white dots in violin plots indicate the median values and inner boxplots indicate interquartile range. The number of data was marked in each plot. 65

Supplementary Figure 15: Correlation of different methods. Correlation (R^2) of the refined bond distances, angles, rotatable dihedrals, real-space Z-difference (RSZD) score and strain energy for our (**a**) neutral (number of data: 312, 452, 219, 7, 7) and (**b**) charged (number of data: 87， 120， 53， 3， 3) drug/inhibitor cases in the selected 10 protein-drug/inhibitor systems (shown in Fig. 1) from **M1-M10** QRs with respect to **M7** as the reference. 69

Supplementary Figure 16: Structural comparison in the gas phase and in proteins (10 systems). Root mean square deviation (RMSD) of the selected 10 structures (shown in Fig. 1b) optimized using the ANI-2x, ANI-1ccx, AIQM1, and GFN2-xTB methods compared to the ωB97X/6-31G(d) method for the neutral drug/inhibitor cases in the gas phase (orange) and in the proteins after quantum refinement (blue) and for the charged drug/inhibitor cases in the gas

phase (red) and in the proteins after quantum refinements (green). The QR data by the **M7**, **M8**, **M9**, **M10** and **M6R** schemes (ONIOM3(DFT:SE:MM), ONIOM3(ANI-2x:SE:MM), ONIOM3(ANI-1ccx:SE:MM), ONIOM3(AIQM1:SE:MM) and ONIOM2(SE:MM), respectively) were used. 70
Supplementary Figure 17: Correlation of different methods (QR50). Correlation (R^2) of the refined bond distances, angles, rotatable dihedrals, real-space Z-difference (RSZD) score and strain energy for our (**a**) neutral (number of data: 901, 1292, 486, 24, 24) and (**b**) charged (number of data: 704, 993, 408, 26, 26) drug/inhibitor cases in the all 50 protein-drug/inhibitor systems from **M1-M10** QRs with respect to **M7** as the reference. 72
Supplementary Figure 18: Imatinib in spleen tyrosine kinase. (**a**) Crystal structure of spleen tyrosine kinase with imatinib (STI). ONIOM layers by different colors: yellow: high layer; red: medium layer; green: low layer. Ligand imatinib is presented in stick and balls. (**b**) Structure of imatinib. 98
Supplementary Figure 19: Real-space Z-difference (RSZD) of imatinib. RSZD+ (green) and RSZD- (red) scores of imatinib (STI) in spleen tyrosine kinase from various quantum refinement schemes (**M1-M10**). Those results for X-ray were taken from the experimental structure without our further refinement. 99
Supplementary Figure 20: Strain energy of imatinib. Strain energy (ΔE, kcal·mol^{-1}) at ωB97X-D/6-31G(d) level for imatinib (STI) in spleen tyrosine kinase determined by various quantum refinement schemes (**M1-M10**). Those results for X-ray were taken from the experimental structure without our further refinement... 99
Supplementary Figure 21: Electron density maps of imatinib. Structures for imatinib (STI) in spleen tyrosine kinase from various quantum refinement schemes (**M1-M10**), including the electron density maps (2mFo-DFc maps, contoured at 1.0 σ (blue), mFo-DFc maps, contoured at +3.0 σ (green), and mFo-DFc maps, contoured at -3.0 σ (red)). Those results for X-ray were taken from the experimental structure without our further refinement. 100
Supplementary Figure 22: Key coordinates of quantum refinement results of imatinib. Deviation in the refined bond distances (Δr, n = 41), angles (Δθ, n = 56) and dihedrals (Δϕ, n = 20) of imatinib (STI) in spleen tyrosine kinase from various quantum refinement schemes (**M1-M10**) and X-ray structure which are compared to those obtained from the most reliable **M7** scheme. The solid line represents the upper and lower values. 101
Supplementary Figure 23: Ibrutinib in Bruton's tyrosine kinase. (**a**) Crystal structure of Bruton's tyrosine kinase with ibrutinib (1E8). ONIOM layer by different color: yellow: high layer; red: medium layer; green: low layer. Ligand imatinib is presented in stick and balls. (**b**) Structure of ibrutinib. 102
Supplementary Figure 24: Real-space Z-difference (RSZD) of ibrutinib. RSZD+ (green) and RSZD- (red) scores of ibrutinib (1E8) in Bruton’s tyrosine kinase from various quantum refinement schemes (**M1-M10**). Those results for X-ray were taken from the experimental structure without our further refinement. 103
Supplementary Figure 25: Strain energy of ibrutinib. Strain energy (ΔE, kcal·mol^{-1}) at ωB97X-D/6-31G(d) level for ibrutinib (1E8) in Bruton's tyrosine kinase determined by various quantum refinement schemes (**M1-M10**). Those results for X-ray were taken from the experimental structure without our further refinement.. 103
Supplementary Figure 26: Electron density maps of ibrutinib. Structures for ibrutinib (1E8) in Bruton’s tyrosine kinase from various quantum refinement schemes (**M1-M10**), including the electron density maps (2mFo-DFc maps, contoured at 1.0 σ (blue), mFo-DFc maps, contoured at +3.0 σ (green), and mFo-DFc maps, contoured at -3.0 σ (red)). Those results for X-ray were taken from the experimental structure without our further refinement. 104
Supplementary Figure 27: Key coordinates of quantum refinement results of ibrutinib. Deviation in the refined bond distances (Δr, n = 37), angles (Δθ, n = 52) and dihedrals (Δϕ, n = 18) of ibrutinib (1E8) in Bruton's tyrosine kinase from various quantum refinement schemes

(**M1-M10**) and X-ray structure which are compared to those obtained from the most reliable **M7** scheme. The solid line represents the upper and lower values. 105
Supplementary Figure 28: 2D diagram of N-Glycosylation in 4HZZ 106
Supplementary Figure 29: Oseltamivir in influenza neuraminidase. (**a**) Crystal structure of influenza neuraminidase with oseltamivir (G39). ONIOM layer by different color: yellow: high layer; red: medium layer; green: low layer. Ligand oseltamivir is presented in stick and balls. (**b**) Structure of oseltamivir. 107
Supplementary Figure 30: Real-space Z-difference (RSZD) of oseltamivir. RSZD+ (green) and RSZD- (red) scores of oseltamivir (G39) in influenza neuraminidase from various quantum refinement schemes (**M1-M10**). Those results for X-ray were taken from the experimental structure without our further refinement. 108
Supplementary Figure 31: Strain energy of oseltamivir. Strain energy (ΔE, $kcal \cdot mol^{-1}$) at ωB97X-D/6-31G(d) level for oseltamivir (G39) in influenza neuraminidase determined by various quantum refinement schemes (**M1-M10**). Those results for X-ray were taken from the experimental structure without our further refinement.. 108
Supplementary Figure 32: Electron density maps of oseltamivir. Structures for oseltamivir (G39) in influenza neuraminidase from various quantum refinement schemes (**M1-M10**), including the electron density maps (2mFo-DFc maps, contoured at 1.0 σ (blue), mFo-DFc maps, contoured at +3.0 σ (green), and mFo-DFc maps, contoured at -3.0 σ (red)). Those results for X-ray were taken from the experimental structure without our further refinement......... 109
Supplementary Figure 33: Key coordinates of quantum refinement results of oseltamivir. Deviation in the refined bond distances (Δr, n = 20), angles (Δθ, n = 27) and dihedrals (Δϕ, n = 16) of oseltamivir (G39) in influenza neuraminidase from various quantum refinement schemes (**M1-M10**) and X-ray structure which are compared to those obtained from the most reliable **M7** scheme. The solid line represents the upper and lower values. 110
Supplementary Figure 34: Osimertinib in EGFR. (**a**) Crystal structure of EGFR with osimertinib (YY3). ONIOM layer by different color: yellow: high layer; red: medium layer; green: low layer. Ligand osimertinib is presented in stick and balls. (**b**) Structure of osimertinib. 111
Supplementary Figure 35: Real-space Z-difference (RSZD) of osimertinib. RSZD+ (green) and RSZD- (red) scores of osimertinib (YY3) in EGFR from various quantum refinement schemes (**M1-M10**). Those results for X-ray were taken from the experimental structure without our further refinement. 112
Supplementary Figure 36: Strain energy of osimertinib. Strain energy (ΔE, $kcal \cdot mol^{-1}$) at ωB97X-D/6-31G(d) level for osimertinib (YY3) in EGFR determined by various quantum refinement schemes (**M1-M10**). Those results for X-ray were taken from the experimental structure without our further refinement. 112
Supplementary Figure 37: Electron density maps of osimertinib. Structures for osimertinib (YY3) in EGFR from various quantum refinement schemes (**M1-M10**), including the electron density maps (2mFo-DFc maps, contoured at 1.0 σ (blue), mFo-DFc maps, contoured at +3.0 σ (green), and mFo-DFc maps, contoured at -3.0 σ (red)). Those results for X-ray were taken from the experimental structure without our further refinement. 113
Supplementary Figure 38: Key coordinates of quantum refinement results of osimertinib. Deviation in the refined bond distances (Δr, n = 40), angles (Δθ, n = 56) and dihedrals (Δϕ, n = 25) of osimertinib (YY3) in EGFR from various quantum refinement schemes (**M1-M10**) and X-ray structure which are compared to those obtained from the most reliable **M7** scheme. The solid line represents the upper and lower values. 114
Supplementary Figure 39: Nirmatrelvir in SARS-CoV-2 main protease. (**a,c**) Crystal structure of the bonded form SARS-CoV-2 main protease with nirmatrelvir (4WI) and (**b, d**) its nonbonded form; ONIOM layer by different color: yellow: high layer; red: medium layer; green: low layer. Ligand nirmatrelvir and CYS are presented its stick and balls................. 116

Supplementary Figure 40: Real-space Z-difference (RSZD) of the bonded form nirmatrelvir. RSZD+ (green) and RSZD- (red) scores of the bonded form nirmatrelvir (4WI) + CYS in SARS-CoV-2 main protease from various quantum refinement schemes (**M1-M10**). Those results for X-ray were taken from the experimental structure without our further refinement. 118
Supplementary Figure 41: Strain energy of the bonded form nirmatrelvir. Strain energy (ΔE, kcal·mol^{-1}) at ωB97X-D/6-31G(d) level for the bonded form nirmatrelvir (4WI) in SARS-CoV-2 main protease determined by various quantum refinement schemes (**M1-M10**). Those results for X-ray were taken from the experimental structure without our further refinement. 118
Supplementary Figure 42: Electron density maps of the bonded form nirmatrelvir. Structures for the bonded form nirmatrelvir (4WI) in SARS-CoV-2 main protease from various quantum refinement schemes (**M1-M10**), including the electron density maps (2mFo-DFc maps, contoured at 1.0 σ (blue), mFo-DFc maps, contoured at +3.0 σ (green), and mFo-DFc maps, contoured at -3.0 σ (red)). Those results for X-ray were taken from the experimental structure without our further refinement. 119
Supplementary Figure 43: Key coordinates of quantum refinement results of the bonded form nirmatrelvir. Deviation in the refined bond distances (Δr, n = 47), angles (Δθ, n = 69) and dihedrals (Δϕ, n = 54) of the bonded form nirmatrelvir (4WI) in SARS-CoV-2 main protease from various quantum refinement schemes (**M1-M10**) and X-ray structure which are compared to those obtained from the most reliable **M7** scheme. The solid line represents the upper and lower values. 120
Supplementary Figure 44: Real-space Z-difference (RSZD) of the bonded and nonbonded forms nirmatrelvir (M1). RSZD+ (green) and RSZD- (red) scores of nirmatrelvir (4WI) + CYS in SARS-CoV-2 main protease with different occupations (bonded: nonbonded forms) from quantum refinement using **M1**. Those results for X-ray were taken from the experimental structure without our further refinement. 121
Supplementary Figure 45: Electron density maps of the bonded and nonbonded forms nirmatrelvir (M1). Structures for nirmatrelvir (4WI) in SARS-CoV-2 main protease with different occupations (bonded: nonbonded forms) from quantum refinement using **M1**, including the electron density maps (2mFo-DFc maps, contoured at 1.0 σ (blue), mFo-DFc maps, contoured at +3.0 σ (green), and mFo-DFc maps, contoured at -3.0 σ (red)). Those results for X-ray were taken from the experimental structure without our further refinement. 122
Supplementary Figure 46: Real-space Z-difference (RSZD) of the bonded and nonbonded forms nirmatrelvir (M7). RSZD+ (green) and RSZD- (red) scores of nirmatrelvir (4WI) + CYS in SARS-CoV-2 main protease with different occupations (bonded: nonbonded forms) from quantum refinement using **M7**. Those results for X-ray were taken from the experimental structure without our further refinement. 123
Supplementary Figure 47: Electron density maps of the bonded and nonbonded forms nirmatrelvir (M7). Structures for nirmatrelvir (4WI) in SARS-CoV-2 main protease with different occupations (bonded: nonbonded forms) from quantum refinement using **M7**, including the electron density maps (2mFo-DFc maps, contoured at 1.0 σ (blue), mFo-DFc maps, contoured at +3.0 σ (green), and mFo-DFc maps, contoured at -3.0 σ (red)). Those results for X-ray were taken from the experimental structure without our further refinement. 124
Supplementary Figure 48: Real-space Z-difference (RSZD) of the bonded and nonbonded forms nirmatrelvir (M9). RSZD+ (green) and RSZD- (red) scores of nirmatrelvir (4WI) + CYS in SARS-CoV-2 main protease with different occupations (bonded: nonbonded forms) from quantum refinement using **M9**. Those results for X-ray were taken from the experimental structure without our further refinement. 125
Supplementary Figure 49: Electron density maps of the bonded and nonbonded forms nirmatrelvir (M9). Structures for nirmatrelvir (4WI) in SARS-CoV-2 main protease with

different occupations (bonded: nonbonded forms) from quantum refinement using **M9**, including the electron density maps (2mFo-DFc maps, contoured at 1.0 σ (blue), mFo-DFc maps, contoured at +3.0 σ (green), and mFo-DFc maps, contoured at -3.0 σ (red)). Those results for X-ray were taken from the experimental structure without our further refinement......... 126
Supplementary Figure 50: Real-space Z-difference (RSZD) of the bonded and nonbonded forms nirmatrelvir (7:3). RSZD+ (green) and RSZD- (red) scores of nirmatrelvir (4WI) + CYS in SARS-CoV-2 main protease with 7:3 occupations (bonded: nonbonded forms) from various quantum refinement schemes (**M1-M10**). Those results for X-ray were taken from the experimental structure without our further refinement.. 127
Supplementary Figure 51: Real-space Z-difference (RSZD) of the bonded and nonbonded forms nirmatrelvir (7:3). Structures for nirmatrelvir (4WI) in SARS-CoV-2 main protease with 7:3 occupations (bonded: nonbonded forms) from various quantum refinement schemes (**M1-M10**) and X-ray, including the electron density maps (2mFo-DFc maps, contoured at 1.0 σ (blue), mFo-DFc maps, contoured at +3.0 σ (green), and mFo-DFc maps, contoured at -3.0 σ (red)). Those results for X-ray were taken from the experimental structure without our further refinement.. 127
Supplementary Figure 52: Electron density maps of the available wild-type SARS-CoV-2 main protease with nirmatrelvir. Available crystal structures for nirmatrelvir (4WI) in wild-type SARS-CoV-2 main protease including the electron density maps (2mFo-DFc maps, contoured at 1.0 σ (blue), mFo-DFc maps, contoured at +3.0 σ (green), and mFo-DFc maps, contoured at -3.0 σ (red))... 128
Supplementary Figure 53: Rivaroxaban in Factor Xa. (**a**) Crystal structure of Factor Xa with rivaroxaban (RIV). ONIOM layer by different color: yellow: high layer; red: medium layer; green: low layer. Ligand rivaroxaban is presented in stick and balls. (**b**) Structure of rivaroxaban. 129
Supplementary Figure 54: Real-space Z-difference (RSZD) of rivaroxaban. RSZD+ (green) and RSZD- (red) scores of rivaroxaban (RIV) in Factor Xa from various quantum refinement schemes (**M1-M10**). Those results for X-ray were taken from the experimental structure without our further refinement. 130
Supplementary Figure 55: Strain energy of rivaroxaban. Strain energy (ΔE, kcal·mol^{-1}) at ωB97X-D/6-31G(d) level for rivaroxaban (RIV) in Factor Xa determined by various quantum refinement schemes (**M1-M10**). Those results for X-ray were taken from the experimental structure without our further refinement. 130
Supplementary Figure 56: Electron density maps of rivaroxaban. Structures for rivaroxaban (RIV) in Factor Xa from various quantum refinement schemes (**M1-M10**), including the electron density maps (2mFo-DFc maps, contoured at 1.0 σ (blue), mFo-DFc maps, contoured at +3.0 σ (green), and mFo-DFc maps, contoured at -3.0 σ (red)). Those results for X-ray were taken from the experimental structure without our further refinement......... 131
Supplementary Figure 57: Key coordinates of quantum refinement results of rivaroxaban. Deviation in the refined bond distances (Δr, n = 32), angles (Δθ, n = 45) and dihedrals (Δϕ, n = 17) of rivaroxaban (RIV) in Factor Xa from various quantum refinement schemes (**M1-M10**) and X-ray structure which are compared to those obtained from the most reliable **M7** scheme. The solid line represents the upper and lower values. 132
Supplementary Figure 58: Darunavir in HIV-1. (**a**) Crystal structure of HIV-1 with darunavir (017). ONIOM layer by different color: yellow: high layer; red: medium layer; green: low layer. Ligand darunavir is presented in stick and balls. (**b**) Structure of darunavir........ 133
Supplementary Figure 59: Real-space Z-difference (RSZD) of darunavir. RSZD+ (green) and RSZD- (red) scores of darunavir (017) in HIV-1 from various quantum refinement schemes (**M1-M10**). Those results for X-ray were taken from the experimental structure without our further refinement... 134
Supplementary Figure 60: Strain energy of darunavir. Strain energy (ΔE, kcal·mol^{-1}) at

ωB97X-D/6-31G(d) level for darunavir (017) in HIV-1 determined by various quantum refinement schemes (**M1-M10**). Those results for X-ray were taken from the experimental structure without our further refinement. 134
Supplementary Figure 61: Electron density maps of darunavir. Structures for darunavir (017) in HIV-1 from various quantum refinement schemes (**M1-M10**), including the electron density maps (2mFo-DFc maps, contoured at 1.0 σ (blue), mFo-DFc maps, contoured at +3.0 σ (green), and mFo-DFc maps, contoured at -3.0 σ (red)). Those results for X-ray were taken from the experimental structure without our further refinement. 135
Supplementary Figure 62: Key coordinates of quantum refinement results of darunavir. Deviation in the refined bond distances (Δr, n = 41), angles (Δθ, n = 58) and dihedrals (Δϕ, n = 36) of darunavir (017) in HIV-1 from various quantum refinement schemes (**M1-M10**) and X-ray structure which are compared to those obtained from the most reliable **M7** scheme. The solid line represents the upper and lower values. 136
Supplementary Figure 63: CPI-0610 in Bromodomain. (**a**) Crystal structure of Bromodomain with CPI-0610 (62G). ONIOM layer by different color: yellow: high layer; red: medium layer; green: low layer. Ligand CPI-0610 is presented in stick and balls. (**b**) Structure of CPI-0610. 137
Supplementary Figure 64: Real-space Z-difference (RSZD) of CPI-0610. RSZD+ (green) and RSZD- (red) scores of CPI-0610 (62G) in Bromodomain from various quantum refinement schemes (**M1-M10**). Those results for X-ray were taken from the experimental structure without our further refinement. 138
Supplementary Figure 65: Strain energy of CPI-0610. Strain energy (ΔE, kcal·mol^{-1}) at ωB97X-D/6-31G(d) level for CPI-0610 (62G) in Bromodomain determined by various quantum refinement schemes (**M1-M10**). Those results for X-ray were taken from the experimental structure without our further refinement. 138
Supplementary Figure 66: Electron density maps of CPI-0610. Structures for CPI-0610 (62G) in Bromodomain from various quantum refinement schemes (**M1-M10**), including the electron density maps (2mFo-DFc maps, contoured at 1.0 σ (blue), mFo-DFc maps, contoured at +3.0 σ (green), and mFo-DFc maps, contoured at -3.0 σ (red)). Those results for X-ray were taken from the experimental structure without our further refinement. 139
Supplementary Figure 67: Key coordinates of quantum refinement results of CPI-0610. Deviation in the refined bond distances (Δr, n = 29), angles (Δθ, n = 42) and dihedrals (Δϕ, n = 8) of CPI-0610 (62G) in Bromodomain from various quantum refinement schemes (**M1-M10**) and X-ray structure which are compared to those obtained from the most reliable **M7** scheme. The solid line represents the upper and lower values. 140
Supplementary Figure 68: Mometasone in hormone receptor. (**a**) Crystal structure of hormone receptor with mometasone (MOF). ONIOM layer by different color: yellow: high layer; red: medium layer; green: low layer. Ligand mometasone is presented in stick and balls. (**b**) Structure of mometasone. 141
Supplementary Figure 69: Real-space Z-difference (RSZD) of mometasone. RSZD+ (green) and RSZD- (red) scores of mometasone (MOF) in hormone receptor from various quantum refinement schemes (**M1-M10**). Those results for X-ray were taken from the experimental structure without our further refinement. 142
Supplementary Figure 70: Strain energy of mometasone. Strain energy (ΔE, kcal·mol^{-1}) at ωB97X-D/6-31G(d) level for mometasone (MOF) in hormone receptor determined by various quantum refinement schemes (**M1-M10**). Those results for X-ray were taken from the experimental structure without our further refinement. 142
Supplementary Figure 71: Electron density maps of mometasone. Structures for mometasone (MOF) in hormone receptor from various quantum refinement schemes (**M1-M10**), including the electron density maps (2mFo-DFc maps, contoured at 1.0 σ (blue), mFo-DFc maps, contoured at +3.0 σ (green), and mFo-DFc maps, contoured at -3.0 σ (red)). Those

results for X-ray were taken from the experimental structure without our further refinement. 143
Supplementary Figure 72: Key coordinates of quantum refinement results of mometasone. Deviation in the refined bond distances (Δr, n = 39), angles (Δθ, n = 64) and dihedrals (Δϕ, n = 17) of mometasone (MOF) in hormone receptor from various quantum refinement schemes (**M1-M10**) and X-ray structure which are compared to those obtained from the most reliable **M7** scheme. The solid line represents the upper and lower values. 144
Supplementary Figure 73: Ciprofloxacin in salmonella typhi ompF. (**a**) Crystal structure of Salmonella typhi OmpF with ciprofloxacin (CPF). ONIOM layer by different color: yellow: high layer; red: medium layer; green: low layer. Ligand ciprofloxacin is presented in stick and balls. (**b**) Structure of ciprofloxacin. 146
Supplementary Figure 74: Real-space Z-difference (RSZD) of ciprofloxacin. RSZD+ (green) and RSZD- (red) scores of ciprofloxacin (CPF) in Salmonella typhi OmpF from various quantum refinement schemes (**M1-M10**). Those results for X-ray were taken from the experimental structure without our further refinement... 147
Supplementary Figure 75: Strain energy of ciprofloxacin. Strain energy (ΔE, kcal·mol^{-1}) at ωB97X-D/6-31G(d) level for ciprofloxacin (CPF) in Salmonella typhi OmpF determined by various quantum refinement schemes (**M1-M10**). Those results for X-ray were taken from the experimental structure without our further refinement... 147
Supplementary Figure 76: Electron density maps of ciprofloxacin. Structures for ciprofloxacin (CPF) in Salmonella typhi OmpF from various quantum refinement schemes (**M1-M10**), including the electron density maps (2mFo-DFc maps, contoured at 1.0 σ (blue), mFo-DFc maps, contoured at +3.0 σ (green), and mFo-DFc maps, contoured at -3.0 σ (red)). Those results for X-ray were taken from the experimental structure without our further refinement.. 148
Supplementary Figure 77: Key coordinates of quantum refinement results of ciprofloxacin. Deviation in the refined bond distances (Δr, n = 27), angles (Δθ, n = 37) and dihedrals (Δϕ, n = 12) of ciprofloxacin (CPF) in Salmonella typhi OmpF from various quantum refinement schemes (**M1-M10**) and X-ray structure which are compared to those obtained from the most reliable **M7** scheme. The solid line represents the upper and lower values............ 149
Supplementary Figure 78: CmeR-bile acid complexes from Campylobacter jejuni. (**a**) Crystal structure of CmeR with Taurocholic acid (TCH). ONIOM layers by different colors: yellow: high layer; red: medium layer; green: low layer. Ligand Taurocholic acid is presented in stick and balls. (**b**) Structure of Taurocholic acid. 150
Supplementary Figure 79: Real-space Z-difference (RSZD) of taurocholic acid. RSZD+ (green) and RSZD- (red) scores of taurocholic acid (TCH) in CmeR from various quantum refinement schemes (**M1-M10**). Those results for X-ray were taken from the experimental structure without our further refinement. 151
Supplementary Figure 80: Strain energy of taurocholic acid. Strain energy (ΔE, kcal·mol^{-1}) at ωB97X-D/6-31G(d) level for taurocholic acid (TCH) in CmeR determined by various quantum refinement schemes (**M1-M10**). Those results for X-ray were taken from the experimental structure without our further refinement... 151
Supplementary Figure 81: Electron density maps of taurocholic acid. Structures for taurocholic acid (TCH) in CmeR from various quantum refinement schemes (**M1-M10**), including the electron density maps (2mFo-DFc maps, contoured at 1.0 σ (blue), mFo-DFc maps, contoured at +3.0 σ (green), and mFo-DFc maps, contoured at -3.0 σ (red)). Those results for X-ray were taken from the experimental structure without our further refinement......... 152
Supplementary Figure 82: Key coordinates of quantum refinement results of taurocholic acid. Deviation in the refined bond distances (Δr, n = 38), angles (Δθ, n = 60) and dihedrals (Δϕ, n = 16) of taurocholic acid (TCH) in CmeR from various quantum refinement schemes (**M1-M10**) and X-ray structure which are compared to those obtained from the most reliable

M7 scheme. The solid line represents the upper and lower values. 153
Supplementary Figure 83: Hispidulin in proto-oncogene kinase Pim1. (**a**) Crystal structure of Pim1 with hispidulin (HUL). ONIOM layers by different colors: yellow: high layer; red: medium layer; green: low layer. Ligand imatinib is presented in stick and balls. (**b**) Structure of hispidulin... 154
Supplementary Figure 84: Real-space Z-difference (RSZD) of hispidulin. RSZD+ (green) and RSZD- (red) scores of hispidulin (HUL) in Pim1 from various quantum refinement schemes (**M1-M10**). Those results for X-ray were taken from the experimental structure without our further refinement. 155
Supplementary Figure 85: Strain energy of hispidulin. Strain energy (ΔE, kcal·mol^{-1}) at ωB97X-D/6-31G(d) level for hispidulin (HUL) in Pim1 determined by various quantum refinement schemes (**M1-M10**). Those results for X-ray were taken from the experimental structure without our further refinement. 155
Supplementary Figure 86: Electron density maps of hispidulin. Structures for hispidulin (HUL) in Pim1 from various quantum refinement schemes (**M1-M10**), including the electron density maps (2mFo-DFc maps, contoured at 1.0 σ (blue), mFo-DFc maps, contoured at +3.0 σ (green), and mFo-DFc maps, contoured at -3.0 σ (red)). Those results for X-ray were taken from the experimental structure without our further refinement... 156
Supplementary Figure 87: Key coordinates of quantum refinement results of hispidulin. Deviation in the refined bond distances (Δr, n = 24), angles (Δθ, n = 35) and dihedrals (Δϕ, n = 6) of hispidulin (HUL) in Pim1 from various quantum refinement schemes (**M1-M10**) and X-ray structure which are compared to those obtained from the most reliable **M7** scheme. The solid line represents the upper and lower values. 157
Supplementary Figure 88: Anticoagulants with dabigatran. (**a**) Crystal structure of anticoagulants with dabigatran (4CC). ONIOM layers by different colors: yellow: high layer; red: medium layer; green: low layer. Ligand dabigatran is presented in stick and balls. (**b**) Structure of dabigatran. 158
Supplementary Figure 89: Real-space Z-difference (RSZD) of dabigatran. RSZD+ (green) and RSZD- (red) scores of dabigatran (4CC) in anticoagulants from various quantum refinement schemes (**M1-M10**). Those results for X-ray were taken from the experimental structure without our further refinement. 159
Supplementary Figure 90: Strain energy of dabigatran. Strain energy (ΔE, kcal·mol^{-1}) at ωB97X-D/6-31G(d) level for dabigatran (4CC) in anticoagulants determined by various quantum refinement schemes (**M1-M10**). Those results for X-ray were taken from the experimental structure without our further refinement... 159
Supplementary Figure 91: Electron density maps of dabigatran. Structures for dabigatran (4CC) in anticoagulants from various quantum refinement schemes (**M1-M10**), including the electron density maps (2mFo-DFc maps, contoured at 1.0 σ (blue), mFo-DFc maps, contoured at +3.0 σ (green), and mFo-DFc maps, contoured at -3.0 σ (red)). Those results for X-ray were taken from the experimental structure without our further refinement. 160
Supplementary Figure 92: Key coordinates of quantum refinement results of dabigatran. Deviation in the refined bond distances (Δr, n = 38), angles (Δθ, n = 53) and dihedrals (Δϕ, n = 26) of dabigatran (4CC) in anticoagulants from various quantum refinement schemes (**M1-M10**) and X-ray structure which are compared to those obtained from the most reliable **M7** scheme. The solid line represents the upper and lower values.. 161
Supplementary Figure 93: Beta-lactamase in complex with ceftazidime. (**a**) Crystal structure of beta-lactamase in complex with ceftazidime (CAZ). ONIOM layers by different colors: yellow: high layer; red: medium layer; green: low layer. Ligand ceftazidime is presented in stick and balls. (**b**) Structure of ceftazidime.. 162
Supplementary Figure 94: Real-space Z-difference (RSZD) of ceftazidime. RSZD+ (green) and RSZD- (red) scores of ceftazidime (CAZ) in beta-lactamase from various quantum

refinement schemes (**M1-M10**). Those results for X-ray were taken from the experimental structure without our further refinement. 163
Supplementary Figure 95: Strain energy of ceftazidime. Strain energy (ΔE, kcal·mol^{-1}) at ωB97X-D/6-31G(d) level for ceftazidime (CAZ) in beta-lactamase determined by various quantum refinement schemes (**M1-M10**). Those results for X-ray were taken from the experimental structure without our further refinement.. 163
Supplementary Figure 96: Electron density maps of ceftazidime. Structures for ceftazidime (CAZ) in beta-lactamase from various quantum refinement schemes (**M1-M10**), including the electron density maps (2mFo-DFc maps, contoured at 1.0 σ (blue), mFo-DFc maps, contoured at +3.0 σ (green), and mFo-DFc maps, contoured at -3.0 σ (red)). Those results for X-ray were taken from the experimental structure without our further refinement. 164
Supplementary Figure 97: Key coordinates of quantum refinement results of ceftazidime. Deviation in the refined bond distances (Δr, n = 38), angles (Δθ, n = 54) and dihedrals (Δϕ, n = 41) of ceftazidime (CAZ) in beta-lactamase from various quantum refinement schemes (**M1-M10**) and X-ray structure which are compared to those obtained from the most reliable **M7** scheme. The solid line represents the upper and lower values. 165
Supplementary Figure 98: Ricin a-chain complexes with neopterin. (**a**) Crystal structure of ricin a-chain complexes with neopterin (NEO). ONIOM layers by different colors: yellow: high layer; red: medium layer; green: low layer. Ligand neopterin is presented in stick and balls. (**b**) Structure of neopterin.. 166
Supplementary Figure 99: Real-space Z-difference (RSZD) of neopterin. RSZD+ (green) and RSZD- (red) scores of neopterin (NEO) in ricin a-chain from various quantum refinement schemes (**M1-M10**). Those results for X-ray were taken from the experimental structure without our further refinement. 167
Supplementary Figure 100: Strain energy of neopterin. Strain energy (ΔE, kcal·mol^{-1}) at ωB97X-D/6-31G(d) level for neopterin (NEO) in ricin a-chain determined by various quantum refinement schemes (**M1-M10**). Those results for X-ray were taken from the experimental structure without our further refinement. 167
Supplementary Figure 101: Electron density maps of neopterin. Structures for neopterin (NEO) in ricin a-chain from various quantum refinement schemes (**M1-M10**), including the electron density maps (2mFo-DFc maps, contoured at 1.0 σ (blue), mFo-DFc maps, contoured at +3.0 σ (green), and mFo-DFc maps, contoured at -3.0 σ (red)). Those results for X-ray were taken from the experimental structure without our further refinement. 168
Supplementary Figure 102: Key coordinates of quantum refinement results of neopterin. Deviation in the refined bond distances (Δr, n = 19), angles (Δθ, n = 27) and dihedrals (Δϕ, n = 10) of neopterin (NEO) in ricin a-chain from various quantum refinement schemes (**M1-M10**) and X-ray structure which are compared to those obtained from the most reliable **M7** scheme. The solid line represents the upper and lower values... 169
Supplementary Figure 103: Streptococcus pneumoniae hyaluronate lyase cocrystallized with ascorbic acid. (**a**) Crystal structure of streptococcus pneumoniae hyaluronate lyase with ascorbic acid (ASC). ONIOM layers by different colors: yellow: high layer; red: medium layer; green: low layer. Ligand ascorbic acid is presented in stick and balls. (**b**) Structure of ascorbic acid. 170
Supplementary Figure 104: Real-space Z-difference (RSZD) of ascorbic acid. RSZD+ (green) and RSZD- (red) scores of ascorbic acid (ASC) in streptococcus pneumoniae hyaluronate lyase from various quantum refinement schemes (**M1-M10**). Those results for X-ray were taken from the experimental structure without our further refinement.................. 171
Supplementary Figure 105: Strain energy of ascorbic acid. Strain energy (ΔE, kcal·mol^{-1}) at ωB97X-D/6-31G(d) level for ascorbic acid (ASC) in streptococcus pneumoniae hyaluronate lyase determined by various quantum refinement schemes (**M1-M10**). Those results for X-ray were taken from the experimental structure without our further refinement........................ 171

Supplementary Figure 106: Electron density maps of ascorbic acid. Structures for ascorbic acid (ASC) in streptococcus pneumoniae hyaluronate lyase from various quantum refinement schemes (**M1-M10**), including the electron density maps (2mFo-DFc maps, contoured at 1.0 σ (blue), mFo-DFc maps, contoured at +3.0 σ (green), and mFo-DFc maps, contoured at -3.0 σ (red)). Those results for X-ray were taken from the experimental structure without our further refinement........ 172
Supplementary Figure 107: Key coordinates of quantum refinement results of ascorbic acid. Deviation in the refined bond distances (Δr, n = 12), angles (Δθ, n = 17) and dihedrals (Δϕ, n = 6) of ascorbic acid (ASC) in streptococcus pneumoniae hyaluronate lyase from various quantum refinement schemes (**M1-M10**) and X-ray structure which are compared to those obtained from the most reliable **M7** scheme. The solid line represents the upper and lower values........ 173
Supplementary Figure 108: Plasmepsin II in complex with the inhibitor EH58. (**a**) Crystal structure of Plasmepsin II in complex with the inhibitor EH58 (EH5). ONIOM layers by different colors: yellow: high layer; red: medium layer; green: low layer. Inhibitor EH58 is presented in stick and balls. (**b**) Structure of inhibitor EH58........ 174
Supplementary Figure 109: Real-space Z-difference (RSZD) of inhibitor EH58. RSZD+ (green) and RSZD- (red) scores of inhibitor EH58 (EH5) in Plasmepsin II from various quantum refinement schemes (**M1-M10**). Those results for X-ray were taken from the experimental structure without our further refinement........ 175
Supplementary Figure 110: Strain energy of inhibitor EH58. Strain energy (ΔE, kcal·mol^{-1}) at ωB97X-D/6-31G(d) level for inhibitor EH58 (EH5) in Plasmepsin II determined by various quantum refinement schemes (**M1-M10**). Those results for X-ray were taken from the experimental structure without our further refinement........ 175
Supplementary Figure 111: Electron density maps of inhibitor EH58. Structures for inhibitor EH58 (EH5) in Plasmepsin II from various quantum refinement schemes (**M1-M10**), including the electron density maps (2mFo-DFc maps, contoured at 1.0 σ (blue), mFo-DFc maps, contoured at +3.0 σ (green), and mFo-DFc maps, contoured at -3.0 σ (red)). Those results for X-ray were taken from the experimental structure without our further refinement........ 176
Supplementary Figure 112: Key coordinates of quantum refinement results of inhibitor EH58. Deviation in the refined bond distances (Δr, n = 65), angles (Δθ, n = 90) and dihedrals (Δϕ, n = 43) of inhibitor EH58 (EH5) in Plasmepsin II from various quantum refinement schemes (**M1-M10**) and X-ray structure which are compared to those obtained from the most reliable **M7** scheme. The solid line represents the upper and lower values........ 177
Supplementary Figure 113: Glutathione transferase in complex with oxidized glutathione. (**a**) Crystal structure of glutathione transferase in complex with oxidized glutathione (GDS). ONIOM layers by different colors: yellow: high layer; red: medium layer; green: low layer. Ligand neopterin is presented in stick and balls. (**b**) Structure of neopterin........ 178
Supplementary Figure 114: Real-space Z-difference (RSZD) of oxidized glutathione. RSZD+ (green) and RSZD- (red) scores of oxidized glutathione (GDS). in glutathione transferase from various quantum refinement schemes (**M1-M10**). Those results for X-ray were taken from the experimental structure without our further refinement........ 179
Supplementary Figure 115: Strain energy of oxidized glutathione. Strain energy (ΔE, kcal·mol^{-1}) at ωB97X-D/6-31G(d) level for oxidized glutathione (GDS). in glutathione transferase determined by various quantum refinement schemes (**M1-M10**). Those results for X-ray were taken from the experimental structure without our further refinement........ 179
Supplementary Figure 116: Electron density maps of oxidized glutathione. Structures for oxidized glutathione (GDS). in glutathione transferase from various quantum refinement schemes (**M1-M10**), including the electron density maps (2mFo-DFc maps, contoured at 1.0 σ (blue), mFo-DFc maps, contoured at +3.0 σ (green), and mFo-DFc maps, contoured at -3.0 σ (red)). Those results for X-ray were taken from the experimental structure without our further

refinement.. 180
Supplementary Figure 117: Key coordinates of quantum refinement results of oxidized glutathione. Deviation in the refined bond distances (Δr, n = 39), angles (Δθ, n = 50) and dihedrals (Δϕ, n = 51) of oxidized glutathione (GDS). in glutathione transferase from various quantum refinement schemes (**M1-M10**) and X-ray structure which are compared to those obtained from the most reliable **M7** scheme. The solid line represents the upper and lower values.. 181
Supplementary Figure 118: GluR2 ligand-binding core (S1S2J) mutant L650T in complex with quisqualic acid. (**a**) Crystal structure of GluR2 ligand-binding core (S1S2J) mutant L650T in complex with quisqualic acid (QUS). ONIOM layers by different colors: yellow: high layer; red: medium layer; green: low layer. Ligand quisqualic acid is presented in stick and balls. (**b**) Structure of quisqualic acid.. 182
Supplementary Figure 119: Real-space Z-difference (RSZD) of quisqualic acid. RSZD+ (green) and RSZD- (red) scores of quisqualic acid (QUS) in GluR2 from various quantum refinement schemes (**M1-M10**). Those results for X-ray were taken from the experimental structure without our further refinement. 183
Supplementary Figure 120: Strain energy of quisqualic acid. Strain energy (ΔE, kcal·mol^{-1}) at ωB97X-D/6-31G(d) level for quisqualic acid (QUS) in GluR2 determined by various quantum refinement schemes (**M1-M10**). Those results for X-ray were taken from the experimental structure without our further refinement... 183
Supplementary Figure 121: Electron density maps of quisqualic acid. Structures for quisqualic acid (QUS) in GluR2 from various quantum refinement schemes (**M1-M10**), including the electron density maps (2mFo-DFc maps, contoured at 1.0 σ (blue), mFo-DFc maps, contoured at +3.0 σ (green), and mFo-DFc maps, contoured at -3.0 σ (red)). Those results for X-ray were taken from the experimental structure without our further refinement......... 184
Supplementary Figure 122: Key coordinates of quantum refinement results of quisqualic acid. Deviation in the refined bond distances (Δr, n = 13), angles (Δθ, n = 18) and dihedrals (Δϕ, n = 8) of quisqualic acid (QUS) in GluR2 from various quantum refinement schemes (**M1-M10**) and X-ray structure which are compared to those obtained from the most reliable **M7** scheme. The solid line represents the upper and lower values.. 185
Supplementary Figure 123: Human PNP complexes with immucillin H. (**a**) Crystal structure of human PNP complexes with forodesine (IMH). ONIOM layers by different colors: yellow: high layer; red: medium layer; green: low layer. Ligand forodesine is presented in stick and balls. (**b**) Structure of forodesine.. 186
Supplementary Figure 124: Real-space Z-difference (RSZD) of forodesine. RSZD+ (green) and RSZD- (red) scores of forodesine (IMH) in human PNP from various quantum refinement schemes (**M1-M10**). Those results for X-ray were taken from the experimental structure without our further refinement. 187
Supplementary Figure 125: Strain energy of forodesine. Strain energy (ΔE, kcal·mol^{-1}) at ωB97X-D/6-31G(d) level for forodesine (IMH) in human PNP determined by various quantum refinement schemes (**M1-M10**). Those results for X-ray were taken from the experimental structure without our further refinement. 187
Supplementary Figure 126: Electron density maps of forodesine. Structures for forodesine (IMH) in human PNP from various quantum refinement schemes (**M1-M10**), including the electron density maps (2mFo-DFc maps, contoured at 1.0 σ (blue), mFo-DFc maps, contoured at +3.0 σ (green), and mFo-DFc maps, contoured at -3.0 σ (red)). Those results for X-ray were taken from the experimental structure without our further refinement. 188
Supplementary Figure 127: Key coordinates of quantum refinement results of forodesine. Deviation in the refined bond distances (Δr, n = 21), angles (Δθ, n = 31) and dihedrals (Δϕ, n = 6) of forodesine (IMH) in human PNP from various quantum refinement schemes (**M1-M10**) and X-ray structure which are compared to those obtained from the most reliable **M7** scheme.

The solid line represents the upper and lower values. 189
Supplementary Figure 128: P38 MAP kinase in complex with a dihydroquinazolinone inhibitor. (**a**) Crystal structure of p38 MAP kinase in complex with a dihydroquinazolinone inhibitor (DQQ). ONIOM layers by different colors: yellow: high layer; red: medium layer; green: low layer. Ligand dihydroquinazolinone inhibitor is presented in stick and balls. (**b**) Structure of dihydroquinazolinone inhibitor. 190
Supplementary Figure 129: Real-space Z-difference (RSZD) of dihydroquinazolinone inhibitor. RSZD+ (green) and RSZD- (red) scores of dihydroquinazolinone inhibitor (DQQ) in p38 MAP kinase from various quantum refinement schemes (**M1-M10**). Those results for X-ray were taken from the experimental structure without our further refinement. 191
Supplementary Figure 130: Strain energy of dihydroquinazolinone inhibitor. Strain energy (ΔE, kcal·mol^{-1}) at ωB97X-D/6-31G(d) level for dihydroquinazolinone inhibitor (DQQ) in p38 MAP kinase determined by various quantum refinement schemes (**M1-M10**). Those results for X-ray were taken from the experimental structure without our further refinement. 191
Supplementary Figure 131: Electron density maps of dihydroquinazolinone inhibitor. Structures for dihydroquinazolinone inhibitor (DQQ) in p38 MAP kinase from various quantum refinement schemes (**M1-M10**), including the electron density maps (2mFo-DFc maps, contoured at 1.0 σ (blue), mFo-DFc maps, contoured at +3.0 σ (green), and mFo-DFc maps, contoured at -3.0 σ (red)). Those results for X-ray were taken from the experimental structure without our further refinement. 192
Supplementary Figure 132: Key coordinates of quantum refinement results of dihydroquinazolinone inhibitor. Deviation in the refined bond distances (Δr, n = 37), angles (Δθ, n = 54) and dihedrals (Δϕ, n = 12) of dihydroquinazolinone inhibitor (DQQ) in p38 MAP kinase from various quantum refinement schemes (**M1-M10**) and X-ray structure which are compared to those obtained from the most reliable **M7** scheme. The solid line represents the upper and lower values. 193
Supplementary Figure 133: Yersinia Protein-Tyrosine Phosphatase complexed with pNCS. (**a**) Crystal structure of Yersinia protein-tyrosine phosphatase complexed with nitrocatechol sulfate (CSN). ONIOM layers by different colors: yellow: high layer; red: medium layer; green: low layer. Ligand nitrocatechol sulfate is presented in stick and balls. (**b**) Structure of nitrocatechol sulfate. 194
Supplementary Figure 134: Real-space Z-difference (RSZD) of nitrocatechol sulfate. RSZD+ (green) and RSZD- (red) scores of nitrocatechol sulfate (CSN) in Yersinia protein-tyrosine phosphatase various quantum refinement schemes (**M1-M10**). Those results for X-ray were taken from the experimental structure without our further refinement. 195
Supplementary Figure 135: Strain energy of nitrocatechol sulfate. Strain energy (ΔE, kcal·mol^{-1}) at ωB97X-D/6-31G(d) level for nitrocatechol sulfate (CSN) in Yersinia protein-tyrosine phosphatase determined by various quantum refinement schemes (**M1-M10**). Those results for X-ray were taken from the experimental structure without our further refinement. 195
Supplementary Figure 136: Electron density maps of nitrocatechol sulfate. Structures for nitrocatechol sulfate (CSN) in Yersinia protein-tyrosine phosphatase from various quantum refinement schemes (**M1-M10**), including the electron density maps (2mFo-DFc maps, contoured at 1.0 σ (blue), mFo-DFc maps, contoured at +3.0 σ (green), and mFo-DFc maps, contoured at -3.0 σ (red)). Those results for X-ray were taken from the experimental structure without our further refinement. 196
Supplementary Figure 137: Key coordinates of quantum refinement results of nitrocatechol sulfate. Deviation in the refined bond distances (Δr, n = 15), angles (Δθ, n = 22) and dihedrals (Δϕ, n = 9) of nitrocatechol sulfate (CSN) in Yersinia protein-tyrosine phosphatase from various quantum refinement schemes (**M1-M10**) and X-ray structure which

are compared to those obtained from the most reliable **M7** scheme. The solid line represents the upper and lower values. 197
Supplementary Figure 138: HIV-1 protease in complex with the cyclic urea inhibitor aha001. (**a**) Crystal structure of HIV-1 protease complexed with AH1. ONIOM layers by different colors: yellow: high layer; red: medium layer; green: low layer. Ligand AH1 is presented in stick and balls. (**b**) Structure of AH1. 198
Supplementary Figure 139: Real-space Z-difference (RSZD) of AH1. RSZD+ (green) and RSZD- (red) scores of AH1 in HIV-1 protease from various quantum refinement schemes (**M1-M10**). Those results for X-ray were taken from the experimental structure without our further refinement.. 199
Supplementary Figure 140: Strain energy of AH1. Strain energy (ΔE, kcal·mol^{-1}) at ωB97X-D/6-31G(d) level of AH1 in HIV-1 protease determined by various quantum refinement schemes (**M1-M10**). Those results for X-ray were taken from the experimental structure without our further refinement. 199
Supplementary Figure 141: Electron density maps of AH1. Structures for AH1 in HIV-1 protease from various quantum refinement schemes (**M1-M10**), including the electron density maps (2mFo-DFc maps, contoured at 1.0 σ (blue), mFo-DFc maps, contoured at +3.0 σ (green), and mFo-DFc maps, contoured at -3.0 σ (red)). Those results for X-ray were taken from the experimental structure without our further refinement.. 200
Supplementary Figure 142: Key coordinates of quantum refinement results of AH1. Deviation in the refined bond distances (Δr, n = 44), angles (Δθ, n = 59) and dihedrals (Δϕ, n = 18) of AH1 in HIV-1 protease from various quantum refinement schemes (**M1-M10**) and X-ray structure which are compared to those obtained from the most reliable **M7** scheme. The solid line represents the upper and lower values. 201
Supplementary Figure 143: HIV-1 protease in complex with the inhibitor BEA425. (**a**) Crystal structure of HIV-1 protease complexed with BEG. ONIOM layers by different colors: yellow: high layer; red: medium layer; green: low layer. Ligand BEG is presented in stick and balls. (**b**) Structure of BEG... 202
Supplementary Figure 144: Real-space Z-difference (RSZD) of BEG. RSZD+ (green) and RSZD- (red) scores of BEG in HIV-1 protease from various quantum refinement schemes (**M1-M10**). Those results for X-ray were taken from the experimental structure without our further refinement.. 203
Supplementary Figure 145: Strain energy of BEG. Strain energy (ΔE, kcal·mol^{-1}) at ωB97X-D/6-31G(d) level of BEG in HIV-1 protease determined by various quantum refinement schemes (**M1-M10**). Those results for X-ray were taken from the experimental structure without our further refinement. 203
Supplementary Figure 146: Electron density maps of BEG. Structures for BEG in HIV-1 protease from various quantum refinement schemes (**M1-M10**), including the electron density maps (2mFo-DFc maps, contoured at 1.0 σ (blue), mFo-DFc maps, contoured at +3.0 σ (green), and mFo-DFc maps, contoured at -3.0 σ (red)). Those results for X-ray were taken from the experimental structure without our further refinement.. 204
Supplementary Figure 147: Key coordinates of quantum refinement results of BEG. Deviation in the refined bond distances (Δr, n = 52), angles (Δθ, n = 72) and dihedrals (Δϕ, n = 34) of BEG in HIV-1 protease from various quantum refinement schemes (**M1-M10**) and X-ray structure which are compared to those obtained from the most reliable **M7** scheme. The solid line represents the upper and lower values. 205
Supplementary Figure 148: Human Phosphodiesterase 5A complexed with Tadalafil. (**a**) Crystal structure of Human Phosphodiesterase 5A complexed with tadalafil (CIA). ONIOM layers by different colors: yellow: high layer; red: medium layer; green: low layer. Ligand tadalafil is presented in stick and balls. (**b**) Structure of tadalafil. 206
Supplementary Figure 149: Real-space Z-difference (RSZD) of tadalafil. RSZD+ (green)

and RSZD- (red) scores of tadalafil (CIA) in Human Phosphodiesterase 5A from various quantum refinement schemes (**M1-M10**). Those results for X-ray were taken from the experimental structure without our further refinement.. 207
Supplementary Figure 150: Strain energy of tadalafil. Strain energy (ΔE, kcal·mol^{-1}) at ωB97X-D/6-31G(d) level for tadalafil (CIA) in Human Phosphodiesterase 5A determined by various quantum refinement schemes (**M1-M10**). Those results for X-ray were taken from the experimental structure without our further refinement.. 207
Supplementary Figure 151: Electron density maps of tadalafil. Structures for tadalafil (CIA) in Human Phosphodiesterase 5A from various quantum refinement schemes (**M1-M10**), including the electron density maps (2mFo-DFc maps, contoured at 1.0 σ (blue), mFo-DFc maps, contoured at +3.0 σ (green), and mFo-DFc maps, contoured at -3.0 σ (red)). Those results for X-ray were taken from the experimental structure without our further refinement......... 208
Supplementary Figure 152: Key coordinates of quantum refinement results of tadalafil. Deviation in the refined bond distances (Δr, n = 34), angles (Δθ, n = 52) and dihedrals (Δϕ, n = 4) of tadalafil (CIA) in Human Phosphodiesterase 5A from various quantum refinement schemes (**M1-M10**) and X-ray structure which are compared to those obtained from the most reliable **M7** scheme. The solid line represents the upper and lower values. 209
Supplementary Figure 153: Androgen receptor ligand binding domain T877A mutant in complex with hydroxyflutamide. (**a**) Crystal structure of androgen receptor ligand binding domain T877A mutant in complex with hydroxyflutamide (HFT). ONIOM layers by different colors: yellow: high layer; red: medium layer; green: low layer. Ligand hydroxyflutamide is presented in stick and balls. (**b**) Structure of hydroxyflutamide. 210
Supplementary Figure 154: Real-space Z-difference (RSZD) of hydroxyflutamide. RSZD+ (green) and RSZD- (red) scores of hydroxyflutamide (HFT) in androgen receptor various quantum refinement schemes (**M1-M10**). Those results for X-ray were taken from the experimental structure without our further refinement.. 211
Supplementary Figure 155: Strain energy of hydroxyflutamide. Strain energy (ΔE, kcal·mol^{-1}) at ωB97X-D/6-31G(d) level for hydroxyflutamide (HFT) in androgen receptor determined by various quantum refinement schemes (**M1-M10**). Those results for X-ray were taken from the experimental structure without our further refinement. 211
Supplementary Figure 156: Electron density maps of hydroxyflutamide. Structures for hydroxyflutamide (HFT) in androgen receptor from various quantum refinement schemes (**M1-M10**), including the electron density maps (2mFo-DFc maps, contoured at 1.0 σ (blue), mFo-DFc maps, contoured at +3.0 σ (green), and mFo-DFc maps, contoured at -3.0 σ (red)). Those results for X-ray were taken from the experimental structure without our further refinement. 212
Supplementary Figure 157: Key coordinates of quantum refinement results of hydroxyflutamide. Deviation in the refined bond distances (Δr, n = 20), angles (Δθ, n = 31) and dihedrals (Δϕ, n = 20) of hydroxyflutamide (HFT) in androgen receptor from various quantum refinement schemes (**M1-M10**) and X-ray structure which are compared to those obtained from the most reliable **M7** scheme. The solid line represents the upper and lower values.. 213
Supplementary Figure 158: Analogues of radicicol bound to HSP90. (**a**) Crystal structure of HSP90 complexed with analogues of radicicol (NP5). ONIOM layers by different colors: yellow: high layer; red: medium layer; green: low layer. Ligand analogues of radicicol is presented in stick and balls. (**b**) Structure of analogues of radicicol.................................... 214
Supplementary Figure 159: Real-space Z-difference (RSZD) of analogues of radicicol. RSZD+ (green) and RSZD- (red) scores of analogues of radicicol (NP5) in HSP90 from various quantum refinement schemes (**M1-M10**). Those results for X-ray were taken from the experimental structure without our further refinement.. 215
Supplementary Figure 160: Strain energy of analogues of radicicol. Strain energy (ΔE,

kcal·mol^{-1}) at ωB97X-D/6-31G(d) level for analogues of radicicol (NP5) in HSP90 determined by various quantum refinement schemes (**M1-M10**). Those results for X-ray were taken from the experimental structure without our further refinement.. 215
Supplementary Figure 161: Electron density maps of analogues of radicicol. Structures for analogues of radicicol (NP5) in HSP90 from various quantum refinement schemes (**M1-M10**), including the electron density maps (2mFo-DFc maps, contoured at 1.0 σ (blue), mFo-DFc maps, contoured at +3.0 σ (green), and mFo-DFc maps, contoured at -3.0 σ (red)). Those results for X-ray were taken from the experimental structure without our further refinement......... 216
Supplementary Figure 162: Key coordinates of quantum refinement results of analogues of radicicol. Deviation in the refined bond distances (Δr, n = 23), angles (Δθ, n = 31) of analogues of radicicol (NP5) in HSP90 from various quantum refinement schemes (**M1-M10**) and X-ray structure which are compared to those obtained from the most reliable **M7** scheme. The solid line represents the upper and lower values.. 217
Supplementary Figure 163: Afimoxifene in Estrogen related receptor gamma. (**a**) Crystal structure of estrogen related receptor gamma complexed with afimoxifene (OHT). ONIOM layers by different colors: yellow: high layer; red: medium layer; green: low layer. Ligand afimoxifene is presented in stick and balls. (**b**) Structure of afimoxifene............................ 218
Supplementary Figure 164: Real-space Z-difference (RSZD) of afimoxifene. RSZD+ (green) and RSZD- (red) scores of afimoxifene (OHT) in estrogen related receptor gamma from various quantum refinement schemes (**M1-M10**). Those results for X-ray were taken from the experimental structure without our further refinement.. 219
Supplementary Figure 165: Strain energy of afimoxifene. Strain energy (ΔE, kcal·mol^{-1}) at ωB97X-D/6-31G(d) level for afimoxifene (OHT) in estrogen related receptor gamma determined by various quantum refinement schemes (**M1-M10**). Those results for X-ray were taken from the experimental structure without our further refinement. 219
Supplementary Figure 166: Electron density maps of afimoxifene. Structures for afimoxifene (OHT) in estrogen related receptor gamma from various quantum refinement schemes (**M1-M10**), including the electron density maps (2mFo-DFc maps, contoured at 1.0 σ (blue), mFo-DFc maps, contoured at +3.0 σ (green), and mFo-DFc maps, contoured at -3.0 σ (red)). Those results for X-ray were taken from the experimental structure without our further refinement.. 220
Supplementary Figure 167: Key coordinates of quantum refinement results of afimoxifene. Deviation in the refined bond distances (Δr, n = 31), angles (Δθ, n = 41) and dihedrals (Δϕ, n = 20) of afimoxifene (OHT) in estrogen related receptor gamma from various quantum refinement schemes (**M1-M10**) and X-ray structure which are compared to those obtained from the most reliable **M7** scheme. The solid line represents the upper and lower values.. 221
Supplementary Figure 168: Human HSP90-alpha with 8-Benzo[1,3]dioxol-,5-ylmethyl-9-butyl-9H-purin-6-ylamine. (**a**) Crystal structure of human HSP90-alpha complexed with inhibitor PU6. ONIOM layers by different colors: yellow: high layer; red: medium layer; green: low layer. Ligand inhibitor PU6 is presented in stick and balls. (**b**) Structure of inhibitor PU6. 222
Supplementary Figure 169: Real-space Z-difference (RSZD) of inhibitor PU6. RSZD+ (green) and RSZD- (red) scores of inhibitor PU6 in human HSP90-alpha from various quantum refinement schemes (**M1-M10**). Those results for X-ray were taken from the experimental structure without our further refinement. 223
Supplementary Figure 170: Strain energy of inhibitor PU6. Strain energy (ΔE, kcal·mol^{-1}) at ωB97X-D/6-31G(d) level inhibitor PU6 in human HSP90-alpha determined by various quantum refinement schemes (**M1-M10**). Those results for X-ray were taken from the experimental structure without our further refinement.. 223
Supplementary Figure 171: Electron density maps of inhibitor PU6. Structures for inhibitor

PU6 in human HSP90-alpha from various quantum refinement schemes (**M1-M10**), including the electron density maps (2mFo-DFc maps, contoured at 1.0 σ (blue), mFo-DFc maps, contoured at +3.0 σ (green), and mFo-DFc maps, contoured at -3.0 σ (red)). Those results for X-ray were taken from the experimental structure without our further refinement. 224
Supplementary Figure 172: Key coordinates of quantum refinement results of inhibitor PU6. Deviation in the refined bond distances (Δr, n = 27), angles (Δθ, n = 38) and dihedrals (Δϕ, n = 8) of inhibitor PU6 in human HSP90-alpha from various quantum refinement schemes (**M1-M10**) and X-ray structure which are compared to those obtained from the most reliable **M7** scheme. The solid line represents the upper and lower values. 225
Supplementary Figure 173: Prolyl oligopeptidase complexed with R-Pro-(decarboxy-Pro)-Type inhibitors. (**a**) Crystal structure of prolyl oligopeptidase complexed with inhibitor X99. ONIOM layers by different colors: yellow: high layer; red: medium layer; green: low layer. Ligand inhibitor X99 is presented in stick and balls. (**b**) Structure of inhibitor X99.. 226
Supplementary Figure 174: Real-space Z-difference (RSZD) of inhibitor X99. RSZD+ (green) and RSZD- (red) scores of inhibitor X99 in prolyl oligopeptidase from various quantum refinement schemes (**M1-M10**). Those results for X-ray were taken from the experimental structure without our further refinement. 227
Supplementary Figure 175: Strain energy of inhibitor X99. Strain energy (ΔE, kcal·mol^{-1}) at ωB97X-D/6-31G(d) level for inhibitor X99 in prolyl oligopeptidase determined by various quantum refinement schemes (**M1-M10**). Those results for X-ray were taken from the experimental structure without our further refinement... 227
Supplementary Figure 176: Electron density maps of inhibitor X99. Structures for inhibitor X99 in prolyl oligopeptidase from various quantum refinement schemes (**M1-M10**), including the electron density maps (2mFo-DFc maps, contoured at 1.0 σ (blue), mFo-DFc maps, contoured at +3.0 σ (green), and mFo-DFc maps, contoured at -3.0 σ (red)). Those results for X-ray were taken from the experimental structure without our further refinement. 228
Supplementary Figure 177: Key coordinates of quantum refinement results of inhibitor X99. Deviation in the refined bond distances (Δr, n = 32), angles (Δθ, n = 48) and dihedrals (Δϕ, n = 17) of inhibitor X99 in prolyl oligopeptidase from various quantum refinement schemes (**M1-M10**) and X-ray structure which are compared to those obtained from the most reliable **M7** scheme. The solid line represents the upper and lower values. 229
Supplementary Figure 178: Nitroxoline in bromodomain of BRD4. (**a**) Crystal structure of bromodomain of BRD4 complexed with nitroxoline (HNQ). ONIOM layers by different colors: yellow: high layer; red: medium layer; green: low layer. Ligand nitroxoline is presented in stick and balls. (**b**) Structure of nitroxoline. 230
Supplementary Figure 179: Real-space Z-difference (RSZD) of nitroxoline. RSZD+ (green) and RSZD- (red) scores of nitroxoline (HNQ) in BRD4 from various quantum refinement schemes (**M1-M10**). Those results for X-ray were taken from the experimental structure without our further refinement. 231
Supplementary Figure 180: Strain energy of nitroxoline. Strain energy (ΔE, kcal·mol^{-1}) at ωB97X-D/6-31G(d) level for nitroxoline (HNQ) in BRD4 determined by various quantum refinement schemes (**M1-M10**). Those results for X-ray were taken from the experimental structure without our further refinement. 231
Supplementary Figure 181: Electron density maps of nitroxoline. Structures for nitroxoline (HNQ) in BRD4 from various quantum refinement schemes (**M1-M10**), including the electron density maps (2mFo-DFc maps, contoured at 1.0 σ (blue), mFo-DFc maps, contoured at +3.0 σ (green), and mFo-DFc maps, contoured at -3.0 σ (red)). Those results for X-ray were taken from the experimental structure without our further refinement... 232
Supplementary Figure 182: Key coordinates of quantum refinement results of nitroxoline. Deviation in the refined bond distances (Δr, n = 15), angles (Δθ, n = 21) and dihedrals (Δϕ, n = 4) of nitroxoline (HNQ) in BRD4 from various quantum refinement schemes (**M1-M10**) and

X-ray structure which are compared to those obtained from the most reliable **M7** scheme. The solid line represents the upper and lower values. 233
Supplementary Figure 183: YfiR complexed with GMP. (**a**) Crystal structure of YfiR complexed with guanosine-5'-monophosphate (5GP). ONIOM layers by different colors: yellow: high layer; red: medium layer; green: low layer. Ligand guanosine-5'-monophosphate is presented in stick and balls. (**b**) Structure of guanosine-5'-monophosphate...................... 234
Supplementary Figure 184: Real-space Z-difference (RSZD) of guanosine-5'-monophosphate. RSZD+ (green) and RSZD- (red) scores of guanosine-5'-monophosphate (5GP) in YfiR from various quantum refinement schemes (**M1-M10**). Those results for X-ray were taken from the experimental structure without our further refinement......................... 235
Supplementary Figure 185: Strain energy of guanosine-5'-monophosphate. Strain energy (ΔE, kcal·mol^{-1}) at ωB97X-D/6-31G(d) level for guanosine-5'-monophosphate (5GP) in YfiR determined by various quantum refinement schemes (**M1-M10**). Those results for X-ray were taken from the experimental structure without our further refinement. 235
Supplementary Figure 186: Electron density maps of guanosine-5'-monophosphate. Structures for guanosine-5'-monophosphate (5GP) in YfiR from various quantum refinement schemes (**M1-M10**), including the electron density maps (2mFo-DFc maps, contoured at 1.0 σ (blue), mFo-DFc maps, contoured at +3.0 σ (green), and mFo-DFc maps, contoured at -3.0 σ (red)). Those results for X-ray were taken from the experimental structure without our further refinement.. 236
Supplementary Figure 187: Key coordinates of quantum refinement results of guanosine-5'-monophosphate. Deviation in the refined bond distances (Δr, n = 26), angles (Δθ, n = 40) and dihedrals (Δϕ, n = 10) of guanosine-5'-monophosphate (5GP) in YfiR from various quantum refinement schemes (**M1-M10**) and X-ray structure which are compared to those obtained from the most reliable **M7** scheme. The solid line represents the upper and lower values.. 237
Supplementary Figure 188: NADH oxidase (NOX) from Thermus thermophilus. (**a**) Crystal structure of NOX complexed with flavin mononucleotide (FMN). ONIOM layers by different colors: yellow: high layer; red: medium layer; green: low layer. Ligand flavin mononucleotide is presented in stick and balls. (**b**) Structure of flavin mononucleotide...... 238
Supplementary Figure 189: Real-space Z-difference (RSZD) of flavin mononucleotide. RSZD+ (green) and RSZD- (red) scores of flavin mononucleotide (FMN) in NOX from various quantum refinement schemes (**M1-M10**). Those results for X-ray were taken from the experimental structure without our further refinement... 239
Supplementary Figure 190: Strain energy of flavin mononucleotide. Strain energy (ΔE, kcal·mol^{-1}) at ωB97X-D/6-31G(d) level for flavin mononucleotide (FMN) in NOX determined by various quantum refinement schemes (**M1-M10**). Those results for X-ray were taken from the experimental structure without our further refinement... 239
Supplementary Figure 191: Electron density maps of flavin mononucleotide. Structures for flavin mononucleotide (FMN) in NOX from various quantum refinement schemes (**M1-M10**), including the electron density maps (2mFo-DFc maps, contoured at 1.0 σ (blue), mFo-DFc maps, contoured at +3.0 σ (green), and mFo-DFc maps, contoured at -3.0 σ (red)). Those results for X-ray were taken from the experimental structure without our further refinement......... 240
Supplementary Figure 192: Key coordinates of quantum refinement results of flavin mononucleotide. Deviation in the refined bond distances (Δr, n = 33), angles (Δθ, n = 50) and dihedrals (Δϕ, n = 18) of flavin mononucleotide (FMN) in NOX from various quantum refinement schemes (**M1-M10**) and X-ray structure which are compared to those obtained from the most reliable **M7** scheme. The solid line represents the upper and lower values............ 241
Supplementary Figure 193: Prolyl-tRNA Synthetase from Methanothermobacter thermautotrophicus bound to cysteine sulfamoyl adenylate. (**a**) Crystal structure of Prolyl-tRNA synthetase complexed with inhibitor 5CA. ONIOM layers by different colors: yellow:

high layer; red: medium layer; green: low layer. Inhibitor 5CA is presented in stick and balls. (**b**) Structure of inhibitor 5CA. 242
Supplementary Figure 194: Real-space Z-difference (RSZD) of inhibitor 5CA. RSZD+ (green) and RSZD- (red) scores of inhibitor 5CA in Prolyl-tRNA synthetase from various quantum refinement schemes (**M1-M10**). Those results for X-ray were taken from the experimental structure without our further refinement. 243
Supplementary Figure 195: Strain energy of inhibitor 5CA. Strain energy (ΔE, kcal·mol^{-1}) at ωB97X-D/6-31G(d) level for inhibitor 5CA in Prolyl-tRNA synthetase determined by various quantum refinement schemes (**M1-M10**). Those results for X-ray were taken from the experimental structure without our further refinement. 243
Supplementary Figure 196: Electron density maps of inhibitor 5CA. Structures for inhibitor 5CA in Prolyl-tRNA synthetase from various quantum refinement schemes (**M1-M10**), including the electron density maps (2mFo-DFc maps, contoured at 1.0 σ (blue), mFo-DFc maps, contoured at +3.0 σ (green), and mFo-DFc maps, contoured at -3.0 σ (red)). Those results for X-ray were taken from the experimental structure without our further refinement. 244
Supplementary Figure 197: Key coordinates of quantum refinement results of inhibitor 5CA. Deviation in the refined bond distances (Δr, n = 31), angles (Δθ, n = 46) and dihedrals (Δϕ, n = 21) of inhibitor 5CA in Prolyl-tRNA synthetase from various quantum refinement schemes (**M1-M10**) and X-ray structure which are compared to those obtained from the most reliable **M7** scheme. The solid line represents the upper and lower values. 245
Supplementary Figure 198: Hormone Binding and Coactivator Assembly by Mineralocorticoid Receptor. (**a**) Crystal structure of mineralocorticoid receptor complexed with corticosterone (C0R). ONIOM layers by different colors: yellow: high layer; red: medium layer; green: low layer. Ligand corticosterone is presented in stick and balls. (**b**) Structure of corticosterone. 246
Supplementary Figure 199: Real-space Z-difference (RSZD) of corticosterone. RSZD+ (green) and RSZD- (red) scores of corticosterone (C0R) in mineralocorticoid receptor from various quantum refinement schemes (**M1-M10**). Those results for X-ray were taken from the experimental structure without our further refinement. 247
Supplementary Figure 200: Strain energy of corticosterone. Strain energy (ΔE, kcal·mol^{-1}) at ωB97X-D/6-31G(d) level for corticosterone (C0R) in mineralocorticoid receptor determined by various quantum refinement schemes (**M1-M10**). Those results for X-ray were taken from the experimental structure without our further refinement. 247
Supplementary Figure 201: Electron density maps of corticosterone. Structures for corticosterone (C0R) in mineralocorticoid receptor from various quantum refinement schemes (**M1-M10**), including the electron density maps (2mFo-DFc maps, contoured at 1.0 σ (blue), mFo-DFc maps, contoured at +3.0 σ (green), and mFo-DFc maps, contoured at -3.0 σ (red)). Those results for X-ray were taken from the experimental structure without our further refinement. 248
Supplementary Figure 202: Key coordinates of quantum refinement results of corticosterone. Deviation in the refined bond distances (Δr, n = 28), angles (Δθ, n = 45) and dihedrals (Δϕ, n = 6) of corticosterone (C0R) in mineralocorticoid receptor from various quantum refinement schemes (**M1-M10**) and X-ray structure which are compared to those obtained from the most reliable **M7** scheme. The solid line represents the upper and lower values. 249
Supplementary Figure 203: NK1 fragment of HGF/SF complexed with CHES. (**a**) Crystal structure of NK1 fragment of HGF/SF complexed with N-cyclohexyltaurine (NHE). ONIOM layers by different colors: yellow: high layer; red: medium layer; green: low layer. Ligand N-cyclohexyltaurine is presented in stick and balls. (**b**) Structure of N-cyclohexyltaurine. 250
Supplementary Figure 204: Real-space Z-difference (RSZD) of N-cyclohexyltaurine.

RSZD+ (green) and RSZD- (red) scores of N-cyclohexyltaurine (NHE) in HGF/SF from various quantum refinement schemes (**M1-M10**). Those results for X-ray were taken from the experimental structure without our further refinement.. 251
Supplementary Figure 205: Strain energy of N-cyclohexyltaurine. Strain energy (ΔE, kcal·mol^{-1}) at ωB97X-D/6-31G(d) level for N-cyclohexyltaurine (NHE) in HGF/SF determined by various quantum refinement schemes (**M1-M10**). Those results for X-ray were taken from the experimental structure without our further refinement.. 251
Supplementary Figure 206: Electron density maps of N-cyclohexyltaurine. Structures for N-cyclohexyltaurine (NHE) in HGF/SF from various quantum refinement schemes (**M1-M10**), including the electron density maps (2mFo-DFc maps, contoured at 1.0 σ (blue), mFo-DFc maps, contoured at +3.0 σ (green), and mFo-DFc maps, contoured at -3.0 σ (red)). Those results for X-ray were taken from the experimental structure without our further refinement......... 252
Supplementary Figure 207: Key coordinates of quantum refinement results of N-cyclohexyltaurine. Deviation in the refined bond distances (Δr, n = 13), angles (Δθ, n = 17) and dihedrals (Δϕ, n = 7) of N-cyclohexyltaurine (NHE) in HGF/SF from various quantum refinement schemes (**M1-M10**) and X-ray structure which are compared to those obtained from the most reliable **M7** scheme. The solid line represents the upper and lower values............ 253
Supplementary Figure 208: DJ-1 with isatin. (**a**) Crystal structure of DJ-1 complexed with Isatin (ISN). ONIOM layers by different colors: yellow: high layer; red: medium layer; green: low layer. Ligand Isatin is presented in stick and balls. (**b**) Structure of Isatin (bonded and non-bonded)... 254
Supplementary Figure 209: Real-space Z-difference (RSZD) of Isatin (ISN) and CYS106. RSZD scores of Isatin (ISN) and CYS106 in DJ-1 with different occupations (bonded: Non-bonded) from **M7** quantum refinement schemes. Those results for X-ray were taken from the experimental structure without our further refinement.. 255
Supplementary Figure 210: Real-space Z-difference (RSZD) of Isatin (ISN) and CYS106. RSZD+ (green) and RSZD- (red) scores of sum of Isatin (ISN) and CYS(CYM)106 (bonded: Non-bonded=5:5) in DJ-1 from various quantum refinement schemes (**M1-M10**). Those results for X-ray were taken from the experimental structure without our further refinement......... 255
Supplementary Figure 211: Strain energy of Isatin (ISN) and CYM106 (bonded). Strain energy (ΔE, kcal·mol^{-1}) at ωB97X-D/6-31G(d) level for Isatin (ISN) and CYM106 in DJ-1 (bonded case) determined by various quantum refinement schemes (**M1-M10**). Those results for X-ray were taken from the experimental structure without our further refinement......... 256
Supplementary Figure 212: Strain energy of Isatin (ISN) and CYM106 (non-bonded). Strain energy (ΔE, kcal·mol^{-1}) at ωB97X-D/6-31G(d) level for Isatin (ISN) and CYS106 in DJ-1 (non-bonded case) determined by various quantum refinement schemes (**M1-M10**). Those results for X-ray were taken from the experimental structure without our further refinement. 256
Supplementary Figure 213: Electron density maps of Isatin and CYS106. Structures for Isatin (ISN) and CYS(CYM)106 (bonded: non-bonded=5:5) in DJ-1 from various quantum refinement schemes (**M1-M10**), including the electron density maps (2mFo-DFc maps, contoured at 1.0 σ (blue), mFo-DFc maps, contoured at +3.0 σ (green), and mFo-DFc maps, contoured at -3.0 σ (red)). Those results for X-ray were taken from the experimental structure without our further refinement. 257
Supplementary Figure 214: Key coordinates of quantum refinement results of Isatin and CYM106 (bonded). Deviation in the refined bond distances (Δr, n = 18), angles (Δθ, n = 26) and dihedrals (Δϕ, n = 8) of Isatin (ISN) and CYM106 in DJ-1 (bonded case) from various quantum refinement schemes (**M1-M10**) and X-ray structure which are compared to those obtained from the most reliable **M7** scheme. 258
Supplementary Figure 215: Key coordinates of quantum refinement results of Isatin and CYM106 (non-bonded). Deviation in the refined bond distances (Δr, n = 18), angles (Δθ, n =

26) and dihedrals (Δϕ, n = 4) of Isatin (ISN) and CYM106 in DJ-1 (non-bonded case) from various quantum refinement schemes (**M1-M10**) and X-ray structure which are compared to those obtained from the most reliable **M7** scheme. The solid line represents the upper and lower values. 259
Supplementary Figure 216: Type B Chloramphenicol Acetyltransferase from Vibrio cholerae in the Complex with Crystal Violet. (**a**) Crystal structure of type B chloramphenicol acetyltransferase complexed with gentian violet cation (CVI). ONIOM layers by different colors: yellow: high layer; red: medium layer; green: low layer. Ligand gentian violet cation is presented in stick and balls. (**b**) Structure of gentian violet cation. 260
Supplementary Figure 217: Real-space Z-difference (RSZD) of gentian violet cation. RSZD+ (green) and RSZD- (red) scores of gentian violet cation (CVI) in type B chloramphenicol acetyltransferase from various quantum refinement schemes (**M1-M10**). Those results for X-ray were taken from the experimental structure without our further refinement. 261
Supplementary Figure 218: Strain energy of gentian violet cation. Strain energy (ΔE, kcal·mol^{-1}) at ωB97X-D/6-31G(d) level for gentian violet cation (CVI) in type B chloramphenicol acetyltransferase determined by various quantum refinement schemes (**M1-M10**). Those results for X-ray were taken from the experimental structure without our further refinement. 261
Supplementary Figure 219: Electron density maps of gentian violet cation. Structures for gentian violet cation (CVI) in type B chloramphenicol acetyltransferase from various quantum refinement schemes (**M1-M10**), including the electron density maps (2mFo-DFc maps, contoured at 1.0 σ (blue), mFo-DFc maps, contoured at +3.0 σ (green), and mFo-DFc maps, contoured at -3.0 σ (red)). Those results for X-ray were taken from the experimental structure without our further refinement. 262
Supplementary Figure 220: Key coordinates of quantum refinement results of gentian violet cation. Deviation in the refined bond distances (Δr, n = 30), angles (Δθ, n = 42) and dihedrals (Δϕ, n = 24) of gentian violet cation (CVI) in type B chloramphenicol acetyltransferase from various quantum refinement schemes (**M1-M10**) and X-ray structure which are compared to those obtained from the most reliable **M7** scheme. The solid line represents the upper and lower values. 263
Supplementary Figure 221: Substituted 2-Naphthamidine Inhibitors of Urokinase. (**a**) Crystal structure of urokinase complexed with inhibitor 303. ONIOM layers by different colors: yellow: high layer; red: medium layer; green: low layer. Inhibitor 303 is presented in stick and balls. (**b**) Structure of inhibitor 303. 264
Supplementary Figure 222: Real-space Z-difference (RSZD) of inhibitor 303. RSZD+ (green) and RSZD- (red) scores of inhibitor 303 in urokinase from various quantum refinement schemes (**M1-M10**). Those results for X-ray were taken from the experimental structure without our further refinement. 265
Supplementary Figure 223: Strain energy of inhibitor 303. Strain energy (ΔE, kcal·mol^{-1}) at ωB97X-D/6-31G(d) level for inhibitor 303 in urokinase determined by various quantum refinement schemes (**M1-M10**). Those results for X-ray were taken from the experimental structure without our further refinement. 265
Supplementary Figure 224: Electron density maps of inhibitor 303. Structures for inhibitor 303 in urokinase from various quantum refinement schemes (**M1-M10**), including the electron density maps (2mFo-DFc maps, contoured at 1.0 σ (blue), mFo-DFc maps, contoured at +3.0 σ (green), and mFo-DFc maps, contoured at -3.0 σ (red)). Those results for X-ray were taken from the experimental structure without our further refinement. 266
Supplementary Figure 225: Key coordinates of quantum refinement results of inhibitor 303. Deviation in the refined bond distances (Δr, n = 32), angles (Δθ, n = 46) and dihedrals (Δϕ, n = 16) of inhibitor 303 in urokinase from various quantum refinement schemes (**M1-M10**) and

X-ray structure which are compared to those obtained from the most reliable **M7** scheme. The solid line represents the upper and lower values. 267
Supplementary Figure 226: PTP1B-inhibitor complex. (**a**) Crystal structure of PTP1B complexed with inhibitor 910. ONIOM layers by different colors: yellow: high layer; red: medium layer; green: low layer. Inhibitor 910 is presented in stick and balls. (**b**) Structure of inhibitor 910. 268
Supplementary Figure 227: Real-space Z-difference (RSZD) of inhibitor 910. RSZD+ (green) and RSZD- (red) scores of inhibitor 910 in PTP1B from various quantum refinement schemes (**M1-M10**). Those results for X-ray were taken from the experimental structure without our further refinement. 269
Supplementary Figure 228: Strain energy of inhibitor 910. Strain energy (ΔE, kcal·mol^{-1}) at ωB97X-D/6-31G(d) level for inhibitor 910 in PTP1B determined by various quantum refinement schemes (**M1-M10**). Those results for X-ray were taken from the experimental structure without our further refinement. 269
Supplementary Figure 229: Electron density maps of inhibitor 910. Structures for inhibitor 910 in PTP1B from various quantum refinement schemes (**M1-M10**), including the electron density maps (2mFo-DFc maps, contoured at 1.0 σ (blue), mFo-DFc maps, contoured at +3.0 σ (green), and mFo-DFc maps, contoured at -3.0 σ (red)). Those results for X-ray were taken from the experimental structure without our further refinement. 270
Supplementary Figure 230: Key coordinates of quantum refinement results of inhibitor 910. Deviation in the refined bond distances (Δr, n = 30), angles (Δθ, n = 41) and dihedrals (Δϕ, n = 18) of inhibitor 910 in PTP1B from various quantum refinement schemes (**M1-M10**) and X-ray structure which are compared to those obtained from the most reliable **M7** scheme. The solid line represents the upper and lower values. 271
Supplementary Figure 231: Improved calcineurin inhibition by yeast FKBP12-drug complexes. (**a**) Crystal structure of calcineurin complexed with tacrolimus (FK5). ONIOM layers by different colors: yellow: high layer; red: medium layer; green: low layer. Ligand tacrolimus is presented in stick and balls. (**b**) Structure of tacrolimus. 272
Supplementary Figure 232: Real-space Z-difference (RSZD) of tacrolimus. RSZD+ (green) and RSZD- (red) scores of tacrolimus (FK5) in calcineurin from various quantum refinement schemes (**M1-M10**). Those results for X-ray were taken from the experimental structure without our further refinement. 273
Supplementary Figure 233: Strain energy of tacrolimus. Strain energy (ΔE, kcal·mol^{-1}) at ωB97X-D/6-31G(d) level for tacrolimus (FK5) in calcineurin determined by various quantum refinement schemes (**M1-M10**). Those results for X-ray were taken from the experimental structure without our further refinement. 273
Supplementary Figure 234: Electron density maps of tacrolimus. Structures for tacrolimus (FK5) in calcineurin from various quantum refinement schemes (**M1-M10**), including the electron density maps (2mFo-DFc maps, contoured at 1.0 σ (blue), mFo-DFc maps, contoured at +3.0 σ (green), and mFo-DFc maps, contoured at -3.0 σ (red)). Those results for X-ray were taken from the experimental structure without our further refinement. 274
Supplementary Figure 235: Key coordinates of quantum refinement results of tacrolimus. Deviation in the refined bond distances (Δr, n = 60), angles (Δθ, n = 86) and dihedrals (Δϕ, n = 15) of tacrolimus (FK5) in calcineurin from various quantum refinement schemes (**M1-M10**) and X-ray structure which are compared to those obtained from the most reliable **M7** scheme. The solid line represents the upper and lower values. 275
Supplementary Figure 236: Human estrogen receptor alpha ligand-binding domain in complex with compound 18. (**a**) Crystal structure of human estrogen receptor alpha complexed with inhibitor AIJ. ONIOM layers by different colors: yellow: high layer; red: medium layer; green: low layer. Inhibitor AIJ is presented in stick and balls. (**b**) Structure of inhibitor AIJ. 276

Supplementary Figure 237: Real-space Z-difference (RSZD) of inhibitor AIJ. RSZD+ (green) and RSZD- (red) scores of inhibitor AIJ in human estrogen receptor alpha from various quantum refinement schemes (**M1-M10**). Those results for X-ray were taken from the experimental structure without our further refinement.. 277
Supplementary Figure 238: Strain energy of inhibitor AIJ. Strain energy (ΔE, kcal·mol^{-1}) at ωB97X-D/6-31G(d) level for inhibitor AIJ in human estrogen receptor alpha determined by various quantum refinement schemes (**M1-M10**). Those results for X-ray were taken from the experimental structure without our further refinement.. 277
Supplementary Figure 239: Electron density maps of inhibitor AIJ. Structures for inhibitor AIJ in human estrogen receptor alpha from various quantum refinement schemes (**M1-M10**), including the electron density maps (2mFo-DFc maps, contoured at 1.0 σ (blue), mFo-DFc maps, contoured at +3.0 σ (green), and mFo-DFc maps, contoured at -3.0 σ (red)). Those results for X-ray were taken from the experimental structure without our further refinement......... 278
Supplementary Figure 240: Key coordinates of quantum refinement results of inhibitor AIJ. Deviation in the refined bond distances (Δr, n = 37), angles (Δθ, n = 52) and dihedrals (Δϕ, n = 17) of inhibitor AIJ in human estrogen receptor alpha from various quantum refinement schemes (**M1-M10**) and X-ray structure which are compared to those obtained from the most reliable **M7** scheme. The solid line represents the upper and lower values. 279
Supplementary Figure 241: Complex of UNG2 and a small Molecule synthetic Inhibitor. (**a**) Crystal structure of UNG2 complexed with inhibitor 302. ONIOM layers by different colors: yellow: high layer; red: medium layer; green: low layer. Inhibitor 302 is presented in stick and balls. (**b**) Structure of inhibitor 302. 280
Supplementary Figure 242: Real-space Z-difference (RSZD) of inhibitor 302. RSZD+ (green) and RSZD- (red) scores of inhibitor 302 in UNG2 from various quantum refinement schemes (**M1-M10**). Those results for X-ray were taken from the experimental structure without our further refinement. 281
Supplementary Figure 243: Strain energy of inhibitor 302. Strain energy (ΔE, kcal·mol^{-1}) at ωB97X-D/6-31G(d) level for inhibitor 302 in UNG2 determined by various quantum refinement schemes (**M1-M10**). Those results for X-ray were taken from the experimental structure without our further refinement. 281
Supplementary Figure 244: Electron density maps of inhibitor 302. Structures for inhibitor 302 in UNG2 from various quantum refinement schemes (**M1-M10**), including the electron density maps (2mFo-DFc maps, contoured at 1.0 σ (blue), mFo-DFc maps, contoured at +3.0 σ (green), and mFo-DFc maps, contoured at -3.0 σ (red)). Those results for X-ray were taken from the experimental structure without our further refinement. 282
Supplementary Figure 245: Key coordinates of quantum refinement results of inhibitor 302. Deviation in the refined bond distances (Δr, n = 26), angles (Δθ, n = 33) and dihedrals (Δϕ, n = 14) of inhibitor 302 in UNG2 from various quantum refinement schemes (**M1-M10**) and X-ray structure which are compared to those obtained from the most reliable **M7** scheme. The solid line represents the upper and lower values. 283
Supplementary Figure 246: BtGH84 in complex with n-butyl pugnac. (**a**) Crystal structure of BtGH84 complexed with inhibitor NP6. ONIOM layers by different colors: yellow: high layer; red: medium layer; green: low layer. Inhibitor NP6 is presented in stick and balls. (**b**) Structure of inhibitor NP6. 284
Supplementary Figure 247: Real-space Z-difference (RSZD) of inhibitor NP6. RSZD+ (green) and RSZD- (red) scores of inhibitor NP6 in BtGH84 from various quantum refinement schemes (**M1-M10**). Those results for X-ray were taken from the experimental structure without our further refinement. 285
Supplementary Figure 248: Strain energy of inhibitor NP6. Strain energy (ΔE, kcal·mol^{-1}) at ωB97X-D/6-31G(d) level for inhibitor NP6 in BtGH84 determined by various quantum refinement schemes (**M1-M10**). Those results for X-ray were taken from the experimental

structure without our further refinement. 285
Supplementary Figure 249: Electron density maps of inhibitor NP6. Structures for inhibitor NP6 in BtGH84 from various quantum refinement schemes (**M1-M10**), including the electron density maps (2mFo-DFc maps, contoured at 1.0 σ (blue), mFo-DFc maps, contoured at +3.0 σ (green), and mFo-DFc maps, contoured at -3.0 σ (red)). Those results for X-ray were taken from the experimental structure without our further refinement. 286
Supplementary Figure 250: Key coordinates of quantum refinement results of inhibitor NP6. Deviation in the refined bond distances (Δr, n = 28), angles (Δθ, n = 37) and dihedrals (Δϕ, n = 16) of inhibitor NP6 in BtGH84 from various quantum refinement schemes (**M1-M10**) and X-ray structure which are compared to those obtained from the most reliable **M7** scheme. The solid line represents the upper and lower values. 287
Supplementary Figure 251: Orally Active 2-Amino Thienopyrimidine Inhibitors of the HSP90 Chaperone. (**a**) Crystal structure of HSP90 chaperone complexed with inhibitor ZZ4. ONIOM layers by different colors: yellow: high layer; red: medium layer; green: low layer. Inhibitor ZZ4 is presented in stick and balls. (**b**) Structure of inhibitor ZZ4. 288
Supplementary Figure 252: Real-space Z-difference (RSZD) of inhibitor ZZ4. RSZD+ (green) and RSZD- (red) scores of inhibitor ZZ4 in HSP90 chaperone from various quantum refinement schemes (**M1-M10**). Those results for X-ray were taken from the experimental structure without our further refinement. 289
Supplementary Figure 253: Strain energy of inhibitor ZZ4. Strain energy (ΔE, kcal·mol^{-1}) at ωB97X-D/6-31G(d) level for inhibitor ZZ4 in HSP90 chaperone determined by various quantum refinement schemes (**M1-M10**). Those results for X-ray were taken from the experimental structure without our further refinement... 289
Supplementary Figure 254: Electron density maps of inhibitor ZZ4. Structures for inhibitor ZZ4 in HSP90 chaperone from various quantum refinement schemes (**M1-M10**), including the electron density maps (2mFo-DFc maps, contoured at 1.0 σ (blue), mFo-DFc maps, contoured at +3.0 σ (green), and mFo-DFc maps, contoured at -3.0 σ (red)). Those results for X-ray were taken from the experimental structure without our further refinement. 290
Supplementary Figure 255: Key coordinates of quantum refinement results of inhibitor ZZ4. Deviation in the refined bond distances (Δr, n = 25), angles (Δθ, n = 34) and dihedrals (Δϕ, n = 9) of inhibitor ZZ4 in HSP90 chaperone from various quantum refinement schemes (**M1-M10**) and X-ray structure which are compared to those obtained from the most reliable **M7** scheme. The solid line represents the upper and lower values. 291
Supplementary Figure 256: Human HSP90-alpha with 8-(2,5-dimethoxy-benzyl)-2-fluoro-9H-purin-6-ylamine. (**a**) Crystal structure of human HSP90-alpha complexed with inhibitor PU2. ONIOM layers by different colors: yellow: high layer; red: medium layer; green: low layer. Inhibitor PU2 is presented in stick and balls. (**b**) Structure of inhibitor PU2....... 292
Supplementary Figure 257: Real-space Z-difference (RSZD) of inhibitor PU2. RSZD+ (green) and RSZD- (red) scores of inhibitor PU2 in human HSP90-alpha from various quantum refinement schemes (**M1-M10**). Those results for X-ray were taken from the experimental structure without our further refinement. 293
Supplementary Figure 258: Strain energy of inhibitor PU2. Strain energy (ΔE, kcal·mol^{-1}) at ωB97X-D/6-31G(d) level for inhibitor PU2 in human HSP90-alpha determined by various quantum refinement schemes (**M1-M10**). Those results for X-ray were taken from the experimental structure without our further refinement... 293
Supplementary Figure 259: Electron density maps of inhibitor PU2. Structures for inhibitor PU2 in human HSP90-alpha from various quantum refinement schemes (**M1-M10**), including the electron density maps (2mFo-DFc maps, contoured at 1.0 σ (blue), mFo-DFc maps, contoured at +3.0 σ (green), and mFo-DFc maps, contoured at -3.0 σ (red)). Those results for X-ray were taken from the experimental structure without our further refinement. 294
Supplementary Figure 260: Key coordinates of quantum refinement results of inhibitor

PU2. Deviation in the refined bond distances (Δr, n = 24), angles (Δθ, n = 34) and dihedrals (Δϕ, n = 8) of inhibitor PU2 in human HSP90-alpha from various quantum refinement schemes (**M1-M10**) and X-ray structure which are compared to those obtained from the most reliable **M7** scheme. The solid line represents the upper and lower values. 295
Supplementary Figure 261: 2-Amino Thienopyrimidine Inhibitors of the HSP90 Chaperone. (**a**) Crystal structure of HSP90 chaperone complexed with inhibitor ZZ2. ONIOM layers by different colors: yellow: high layer; red: medium layer; green: low layer. Inhibitor ZZ2 is presented in stick and balls. (**b**) Structure of inhibitor ZZ2. 296
Supplementary Figure 262: Real-space Z-difference (RSZD) of inhibitor ZZ2. RSZD+ (green) and RSZD- (red) scores of inhibitor ZZ2 in HSP90 chaperone from various quantum refinement schemes (**M1-M10**). Those results for X-ray were taken from the experimental structure without our further refinement. 297
Supplementary Figure 263: Strain energy of inhibitor ZZ2. Strain energy (ΔE, kcal·mol^{-1}) at ωB97X-D/6-31G(d) level for inhibitor ZZ2 in HSP90 chaperone determined by various quantum refinement schemes (**M1-M10**). Those results for X-ray were taken from the experimental structure without our further refinement. 297
Supplementary Figure 264: Electron density maps of inhibitor ZZ2. Structures for inhibitor ZZ2 in HSP90 chaperone from various quantum refinement schemes (**M1-M10**), including the electron density maps (2mFo-DFc maps, contoured at 1.0 σ (blue), mFo-DFc maps, contoured at +3.0 σ (green), and mFo-DFc maps, contoured at -3.0 σ (red)). Those results for X-ray were taken from the experimental structure without our further refinement. 298
Supplementary Figure 265: Key coordinates of quantum refinement results of inhibitor ZZ2. Deviation in the refined bond distances (Δr, n = 13), angles (Δθ, n = 16) and dihedrals (Δϕ, n = 5) of inhibitor ZZ2 in HSP90 chaperone from various quantum refinement schemes (**M1-M10**) and X-ray structure which are compared to those obtained from the most reliable **M7** scheme. The solid line represents the upper and lower values. 299
Supplementary Figure 266: N-Benzyl-indolo carboxylic acids in adipocyte Fatty-Acid Binding Protein (A-FABP). (**a**) Crystal structure of A-FABP complexed with inhibitor 8CA. ONIOM layers by different colors: yellow: high layer; red: medium layer; green: low layer. Inhibitor 8CA is presented in stick and balls. (**b**) Structure of inhibitor 8CA. 300
Supplementary Figure 267: Real-space Z-difference (RSZD) of inhibitor 8CA. RSZD+ (green) and RSZD- (red) scores of inhibitor 8CA in A-FABP from various quantum refinement schemes (**M1-M10**). Those results for X-ray were taken from the experimental structure without our further refinement. 301
Supplementary Figure 268: Strain energy of inhibitor 8CA. Strain energy (ΔE, kcal·mol^{-1}) at ωB97X-D/6-31G(d) level for inhibitor 8CA in A-FABP determined by various quantum refinement schemes (**M1-M10**). Those results for X-ray were taken from the experimental structure without our further refinement. 301
Supplementary Figure 269: Electron density maps of inhibitor 8CA. Structures for inhibitor 8CA in A-FABP from various quantum refinement schemes (**M1-M10**), including the electron density maps (2mFo-DFc maps, contoured at 1.0 σ (blue), mFo-DFc maps, contoured at +3.0 σ (green), and mFo-DFc maps, contoured at -3.0 σ (red)). Those results for X-ray were taken from the experimental structure without our further refinement. 302
Supplementary Figure 270: Key coordinates of quantum refinement results of inhibitor 8CA. Deviation in the refined bond distances (Δr, n = 26), angles (Δθ, n = 37) and dihedrals (Δϕ, n = 8) of inhibitor 8CA in A-FABP from various quantum refinement schemes (**M1-M10**) and X-ray structure which are compared to those obtained from the most reliable **M7** scheme. The solid line represents the upper and lower values. 303
Supplementary Figure 271: Trypsin complexed with 2-(1H-indol-3-yl)ethanamine. (**a**) Crystal structure of trypsin complexed with tryptamine. ONIOM layers by different colors: yellow: high layer; red: medium layer; green: low layer. Ligand tryptamine is presented in stick

and balls. (**b**) Structure of tryptamine.. 304
Supplementary Figure 272: Real-space Z-difference (RSZD) of tryptamine. RSZD+ (green) and RSZD- (red) scores of tryptamine in trypsin from various quantum refinement schemes (**M1-M10**). Those results for X-ray were taken from the experimental structure without our further refinement... 305
Supplementary Figure 273: Strain energy of tryptamine. Strain energy (ΔE, kcal·mol^{-1}) at ωB97X-D/6-31G(d) level for tryptamine in trypsin determined by various quantum refinement schemes (**M1-M10**). Those results for X-ray were taken from the experimental structure without our further refinement. 305
Supplementary Figure 274: Electron density maps of tryptamine. Structures for tryptamine in trypsin various quantum refinement schemes (**M1-M10**), including the electron density maps (2mFo-DFc maps, contoured at 1.0 σ (blue), mFo-DFc maps, contoured at +3.0 σ (green), and mFo-DFc maps, contoured at -3.0 σ (red)). Those results for X-ray were taken from the experimental structure without our further refinement... 306
Supplementary Figure 275: Key coordinates of quantum refinement results of tryptamine. Deviation in the refined bond distances (Δr, n = 13), angles (Δθ, n = 17) and dihedrals (Δϕ, n = 3) of tryptamine in trypsin from various quantum refinement schemes (**M1-M10**) and X-ray structure which are compared to those obtained from the most reliable **M7** scheme. The solid line represents the upper and lower values... 307
Supplementary Figure 276: Human Smoothened complexed with Vismodegib. (**a**) Crystal structure of human smoothened complexed with vismodegib. ONIOM layers by different colors: yellow: high layer; red: medium layer; green: low layer. Ligand vismodegib is presented in stick and balls. (**b**) Structure of vismodegib... 309
Supplementary Figure 277: Real-space Z-difference (RSZD) of vismodegib (Chain A). RSZD+ (green) and RSZD- (red) scores of vismodegib in human smoothened (Chain A) from various quantum refinement schemes (**M1-M10**). Those results for X-ray were taken from the experimental structure without our further refinement.. 310
Supplementary Figure 278: Real-space Z-difference (RSZD) of vismodegib (Chain B). RSZD+ (green) and RSZD- (red) scores of vismodegib in human smoothened (Chain B) from various quantum refinement schemes (**M1-M10**). Those results for X-ray were taken from the experimental structure without our further refinement.. 310
Supplementary Figure 279: Strain energy of vismodegib. Strain energy (ΔE, kcal·mol^{-1}) at ωB97X-D/6-31G(d) level for vismodegib in human smoothened (Chain A and Chain B) determined by various quantum refinement schemes (**M1-M10**). Those results for X-ray were taken from the experimental structure without our further refinement. 311
Supplementary Figure 280: Electron density maps of vismodegib (chain A). Structures for vismodegib in human smoothened (chain A) from various quantum refinement schemes (**M1-M10**), including the electron density maps (2mFo-DFc maps, contoured at 1.0 σ (blue), mFo-DFc maps, contoured at +3.0 σ (green), and mFo-DFc maps, contoured at -3.0 σ (red)). Those results for X-ray were taken from the experimental structure without our further refinement. 312
Supplementary Figure 281: Electron density maps of vismodegib (chain B). Structures vismodegib in human smoothened (chain B) from various quantum refinement schemes (**M1-M10**), including the electron density maps (2mFo-DFc maps, contoured at 1.0 σ (blue), mFo-DFc maps, contoured at +3.0 σ (green), and mFo-DFc maps, contoured at -3.0 σ (red)). Those results for X-ray were taken from the experimental structure without our further refinement. 313
Supplementary Figure 282: Key coordinates of quantum refinement results of vismodegib. Deviation in the refined bond distances (Δr, n = 58), angles (Δθ, n = 84) and dihedrals (Δϕ, n = 36) of vismodegib in human smoothened from various quantum refinement schemes (**M1-M10**) and X-ray structure which are compared to those obtained from the most reliable **M7** scheme.

The solid line represents the upper and lower values.. 314

List of Tables

Supplementary Table 1: Functional groups in drug/inhibitors. The 40 most frequent functional groups (FGs, Supplementary Figure 5) occurring in our 50 selected drug/inhibitor molecules in this study (QR50 dataset).. 38
Supplementary Table 2: Comparison of different density functional theory (DFT) methods in the gas phase. Median absolute deviation (MAD), root mean square distance (RMSD) and Correlation (R^2) of the optimized bond distances, angles, rotatable dihedrals for the selected 10 drugs/inhibitors (shown in Fig. 1) based on optimization in the gas phase using different functionals and basis sets compared to the DFT (ωB97X/6-31G(d)) method......... 47
Supplementary Table 3: Comparison of different density functional theory (DFT) methods in quantum refinement. Median absolute deviation (MAD), root mean square distance (RMSD) and Correlation (R^2) of the refined bond distances (n = 390), angles (n = 562), rotatable dihedrals (n=259) for the selected 10 drugs/inhibitors (shown in Fig. 1b) based on **M1** quantum refinements using different functionals and basis sets compared to the DFT (ωB97X/6-31G(d)) method. 48
Supplementary Table 4: Comparison of the optimized structures (all systems) in gas phase. Correlation (R^2) and median absolute deviation (MAD) of the refined bond distances (n = 390), angles (n = 560), rotatable dihedrals (n = 259) for the selected 10 drug/inhibitor (shown in Fig. 1) based on optimization in the gas phase using AIQM1, ANI-1ccx, ANI-2x, ANI-1x and GFN2-xTB methods compared to the DFT (ωB97X-D/6-31G(d)) method. ONIOM2(MLP:ANI-2x) method was used for the molecules containing F, Cl and/or S elements, when AIQM1, QDpi, ANI-1ccx or ANI-1x was used... 50
Supplementary Table 5: Comparison of the optimized structures (neutral cases) in gas phase. Correlation (R^2) and median absolute deviation (MAD) of the refined bond distances (n = 303), angles (n = 442), rotatable dihedrals (n = 206) for the neutral cases of the selected 10 drug/inhibitor (shown in Fig. 1) based on optimization in the gas phase using AIQM1, ANI-1ccx, ANI-2x, ANI-1x and GFN2-xTB methods compared to the DFT (ωB97X-D/6-31G(d)) method. ONIOM2(MLP:ANI-2x) method was used for the molecules containing F, Cl and/or S elements, when AIQM1, QDpi, ANI-1ccx or ANI-1x was used. 50
Supplementary Table 6: Comparison of the optimized structures (charged cases) in gas phase. Correlation (R^2) and median absolute deviation (MAD) of the refined bond distances (n = 87), angles (n = 120), rotatable dihedrals (n = 53) for the charged cases of the selected 10 drug/inhibitor (shown in Fig. 1b) based on optimization in the gas phase using AIQM1, ANI-1ccx, ANI-2x, ANI-1x and GFN2-xTB methods compared to the DFT (ωB97X-D/6-31G(d)) method. ONIOM2(MLP:ANI-2x) method was used for the molecules containing F, Cl and/or S elements, when AIQM1, QDpi, ANI-1ccx or ANI-1x was used. 50
Supplementary Table 7: Comparison of the optimized structures (all systems) in the gas phase. Correlation (R^2) and median absolute deviation (MAD) of the refined bond distances (n = 1595), angles (n = 2271), rotatable dihedrals (n = 881) for the selected 50 drugs/inhibitors (QR50) based on optimization in the gas phase using AIQM1, ANI-2x, and GFN2-xTB methods compared to the DFT (ωB97X-D/6-31G(d)) method. ONIOM2(MLP:ANI-2x) method was used for the molecules containing F, Cl and/or S elements, when AIQM1 was used. ONIOM2(MLP:SE) method was used for the molecules containing P and/or Br elements, when AIQM1 or ANI-2x was used. 51
Supplementary Table 8: Comparison of the optimized structure (neutral cases) in the gas phase. Correlation (R^2) and median absolute deviation (MAD) of the refined bond distances (n = 891), angles (n = 1278), rotatable dihedrals (n = 473) for the neutral cases out of the selected 50 drugs/inhibitors (QR50) based on optimization in the gas phase using AIQM1, ANI-2x, and GFN2-xTB methods compared to the DFT (ωB97X-D/6-31G(d)) method. ONIOM2(MLP:ANI-2x) method was used for the molecules containing F, Cl and/or S elements,

when AIQM1 was used. ONIOM2(MLP:SE) method was used for the molecules containing P and/or Br elements, when AIQM1 or ANI-2x was used. 51
Supplementary Table 9: Comparison of the optimized structures (charged cases) in the gas phase. Correlation (R^2) and median absolute deviation (MAD) of the refined bond distances (n = 704), angles (n = 993), rotatable dihedrals (n = 408) for the charged cases out of the selected 50 drugs/inhibitors (QR50) based on optimization in the gas phase using AIQM1, ANI-2x, and GFN2-xTB methods compared to the DFT (ωB97X-D/6-31G(d)) method. ONIOM2(MLP:ANI-2x) method was used for the molecules containing F, Cl and/or S elements, when AIQM1 was used. ONIOM2(MLP:SE) method was used for the molecules containing P and/or Br elements, when AIQM1 or ANI-2x was used. 52
Supplementary Table 10: Comparison of the optimized structures in the gas phase (PB20-QM-3k). Correlation (R^2) and median absolute deviation (MAD) of the refined bond distances (n = 138517), angles (n = 238375), rotatable dihedrals (n = 39777) for the 3156 drug/inhibitor structures (PB20-QM-3k dataset) optimized in the gas phase, using MLPs (MLP-CC: AIQM1, MLP-DFT: ANI-2x) and the (SE) GFN2-xTB method compared to the (QM) ωB97X-D/6-31G(d) method. ONIOM2(MLP:ANI-2x) method was used for the molecules containing F, Cl and/or S elements, when AIQM1 was used. ONIOM2(MLP:SE) method was used for the molecules containing B, P, Se, Br and/or I elements, when AIQM1 or ANI-2x was used..... 61
Supplementary Table 11: Comparison of the optimized structures in the gas phase (neutral cases in PB20-QM-3k). Correlation (R^2) and median absolute deviation (MAD) of the refined bond distances (n = 133796), angles (n = 229905), rotatable dihedrals (n = 37443) for the neutral cases out of the 3156 drug/inhibitor structures (PB20-QM-3k dataset) optimized in the gas phase, using MLPs (MLP-CC: AIQM1, MLP-DFT: ANI-2x) and the (SE) GFN2-xTB method compared to the (QM) ωB97X-D/6-31G(d) method. ONIOM2(MLP:ANI-2x) method was used for the molecules containing F, Cl and/or S elements, when AIQM1 was used. ONIOM2(MLP:SE) method was used for the molecules containing B, P, Se, Br and/or I elements, when AIQM1 or ANI-2x was used. 62
Supplementary Table 12: Comparison of the optimized structures in the gas phase (charged cases in PB20-QM-3k). Correlation (R^2) and median absolute deviation (MAD) of the refined bond distances (n = 4721), angles (n = 8470), rotatable dihedrals (n = 2334) for the charged cases out of the 3156 drug/inhibitor structures (PB20-QM-3k dataset) optimized in the gas phase, using MLPs (MLP-CC: AIQM1, MLP-DFT: ANI-2x) and the (SE) GFN2-xTB method compared to the (QM) ωB97X-D/6-31G(d) method. ONIOM2(MLP:ANI-2x) method was used for the molecules containing F, Cl and/or S elements, when AIQM1 was used. ONIOM2(MLP:SE) method was used for the molecules containing B, P, Se, Br and/or I elements, when AIQM1 or ANI-2x was used. 62
Supplementary Table 13: Comparison of the optimized structures in the gas phase (PB20-QM-8k). Correlation (R^2) and median absolute deviation (MAD) of the refined bond distances (n = 450564), angles (n = 786977), rotatable dihedrals (n = 153593) for the 8776 drug/inhibitor structures (PB20-QM-8k dataset) optimized in the gas phase, using ANI-2x and the (SE) GFN2-xTB method compared to the (QM) ωB97X-D/6-31G(d) method. 63
Supplementary Table 14: Comparison of the optimized structures in the gas phase (neutral cases in PB20-QM-8k). Correlation (R^2) and median absolute deviation (MAD) of the refined bond distances (n = 352815), angles (n = 609657), rotatable dihedrals (n = 114486) for the neutral cases out of the 8776 drug/inhibitor structures (PB20-QM-8k dataset) optimized in the gas phase, using ANI-2x and the (SE) GFN2-xTB method compared to the (QM) ωB97X-D/6-31G(d) method. 64
Supplementary Table 15: Comparison of the optimized structures in the gas phase (charged cases in PB20-QM-3k). Correlation (R^2) and median absolute deviation (MAD) of the refined bond distances (n = 97749), angles (n = 177320), rotatable dihedrals (n = 39107) for the charged cases out of the 8776 drug/inhibitor structures (PB20-QM-8k dataset) optimized

in the gas phase, using ANI-2x and the (SE) GFN2-xTB method compared to the (QM) ωB97X-D/6-31G(d) method. 64
Supplementary Table 16: Real-space Z-difference (RSZD) scores of quantum refinements. RSZD scores of the selected 10 drugs/inhibitors (shown in Fig. 1) in the proteins from our quantum refinement calculations (**M1-M10**). Those results for X-ray were taken from the experimental structure without our further refinement.. 66
Supplementary Table 17: Strain energy of quantum refinements. Strain energy (ΔE, kcal·mol^{-1}) of the selected 10 drugs/inhibitors (shown in Fig. 1) in the proteins from our quantum refinement calculations (**M1-M10**). Those results for X-ray were taken from the experimental structure without our further refinement.. 67
Supplementary Table 18: Selected coordinates. Selected key bonds distances, angles and dihedrals marked in Figure 1b from our **M1-M10** quantum refinement calculations. Those results for X-ray were taken from the experimental structure without our further refinement. 68
Supplementary Table 19: Comparison of quantum refinement results (10 systems). Correlation (R^2) and median absolute deviation (MAD) of the refined bond distances (n = 399), angles (n = 572), rotatable dihedrals (n = 272) for all drugs/inhibitors in the selected 10 protein-drug/inhibitor systems (shown in Fig. 1) from **M1-M10** QRs with respect to **M7** as the reference. Those results for X-ray were taken from the experimental structure without our further refinement.. 68
Supplementary Table 20: Comparison of quantum refinement results (7 neutral cases). Correlation (R^2) and median absolute deviation (MAD) of the refined bond distances (n = 312), angles (n = 452), rotatable dihedrals (n = 219) for the neutral drug/inhibitor cases in the selected 10 protein-drug/inhibitor systems (shown in Fig. 1) from **M1-M10** QRs with respect to **M7** as the reference. Those results for X-ray were taken from the experimental structure without our further refinement. 68
Supplementary Table 21: Comparison of quantum refinement results (3 charged cases). Correlation (R^2) and median absolute deviation (MAD) of the refined bond distances (n = 87), angles (n = 120), rotatable dihedrals (n = 53) for charged drug/inhibitor cases in the selected 10 protein-drug/inhibitor systems (shown in Fig. 1) from **M1-M10** QRs with respect to **M7** as the reference. Those results for X-ray were taken from the experimental structure without our further refinement. 69
Supplementary Table 22: Comparison of quantum refinement results (QR50). Correlation (R^2) and median absolute deviation (MAD) of the refined bond distances (n = 1605), angles (n = 2285), rotatable dihedrals (n = 894) for all drugs/inhibitors in the selected 50 protein-drug/inhibitor systems from **M1-M10** QRs with respect to **M7** as the reference. Those results for X-ray were taken from the experimental structure without our further refinement........... 71
Supplementary Table 23: Comparison of quantum refinement results (neutral cases in QR50). Correlation (R^2) and median absolute deviation (MAD) of the refined bond distances (n = 901), angles (n = 1292), rotatable dihedrals (n = 486) for the neutral drug/inhibitor cases in the selected 50 protein-drug/inhibitor systems from **M1-M10** QRs with respect to **M7** as the reference. Those results for X-ray were taken from the experimental structure without our further refinement. 71
Supplementary Table 24: Comparison of quantum refinement results (charged cases in QR50). Correlation (R^2) and median absolute deviation (MAD) of the refined bond distances (n = 704), angles (n = 993), rotatable dihedrals (n = 408) for the charged drug/inhibitor cases in the selected 50 protein-drug/inhibitor systems from **M1-M10** QRs with respect to **M7** as the reference. Those results for X-ray were taken from the experimental structure without our further refinement. 72
Supplementary Table 25: Real-space Z-difference (RSZD) of quantum refinments. RSZD scores of the all 50 drugs/inhibitors in the proteins (QR50) from our quantum refinement

calculations (**M1-M10**). Those results for X-ray were taken from the experimental structure without our further refinement. 73
Supplementary Table 26: Strain energy of quantum refinements. Strain energy (ΔE, kcal·mol^{-1}) at ωB97X-D/6-31G(d) level of the all 50 drugs/inhibitors in the proteins (QR50) from our quantum refinement calculations (**M1-M10**). Those results for X-ray were taken from the experimental structure without our further refinement.. 74
Supplementary Table 27: Root mean square deviation (RMSD) in bonds of quantum refinements. RMSD (Å) in bonds of the all 50 drugs/inhibitors in the proteins (QR50) from our quantum refinement calculations (**M1-M10**) with respect to **M7** as the reference. Those results for X-ray were taken from the experimental structure without our further refinement. 76
Supplementary Table 28: Median absolute deviation (MAD) in bonds of quantum refinements. MAD (Å) in bonds of the all 50 drugs/inhibitors in the proteins (QR50) from our quantum refinement calculations (**M1-M10**) with respect to **M7** as the reference. Those results for X-ray were taken from the experimental structure without our further refinement........... 78
Supplementary Table 29: R^2 in bonds of quantum refinements. R^2 in bonds of the all 50 drugs/inhibitors in the proteins (QR50) from our quantum refinement calculations (**M1-M10**) with respect to **M7** as the reference. Those results for X-ray were taken from the experimental structure without our further refinement. 80
Supplementary Table 30: Root mean square deviation (RMSD) in angles of quantum refinements. RMSD (°) in angles of the all 50 drugs/inhibitors in the proteins (QR50) from our quantum refinement calculations (**M1-M10**) with respect to **M7** as the reference. Those results for X-ray were taken from the experimental structure without our further refinement........... 82
Supplementary Table 31: Median absolute deviation (MAD) in bonds of quantum refinements. MAD (°) in angles of the all 50 drugs/inhibitors in the proteins (QR50) from our quantum refinement calculations (**M1-M10**) with respect to **M7** as the reference. Those results for X-ray were taken from the experimental structure without our further refinement........... 84
Supplementary Table 32: R^2 in angles of quantum refinements. R^2 in angles of the all 50 drugs/inhibitors in the proteins (QR50) from our quantum refinement calculations (**M1-M10**) with respect to **M7** as the reference. Those results for X-ray were taken from the experimental structure without our further refinement. 86
Supplementary Table 33: Root mean square deviation (RMSD) in dihedrals of quantum refinements. RMSD (°) in dihedrals of the all 50 drugs/inhibitors in the proteins (QR50) from our quantum refinement calculations (**M1-M10**) with respect to **M7** as the reference. Those results for X-ray were taken from the experimental structure without our further refinement. 88
Supplementary Table 34: Median absolute deviation (MAD) in dihedral of quantum refinements. MAD (°) in dihedrals of the all 50 drugs/inhibitors in the proteins (QR50) from our quantum refinement calculations (**M1-M10**) with respect to **M7** as the reference. Those results for X-ray were taken from the experimental structure without our further refinement. 90
Supplementary Table 35: R^2 in dihedral of quantum refinements. R^2 in dihedral of the all 50 drugs/inhibitors in the proteins (QR50) from our quantum refinement calculations (**M1-M10**) with respect to **M7** as the reference. Those results for X-ray were taken from the experimental structure without our further refinement. 92
Supplementary Table 36: Root mean square deviation (RMSD) of the optimized structures in the gas phase and quantum refinements. RMSD of the all 50 drugs/inhibitors (QR50) optimized in gas phase using ANI-2x, AIQM1 and GFN2-xTB compared to the DFT (ωB97X-D/6-31G(d)) method and RMSD of the all 50 drugs/inhibitors refined in the proteins using **M8**, **M10** and **M6R** with respect to **M7** as the reference (the charged cases are highlighted

by red).. 94
Supplementary Table 37: Computational efficiency of quantum refinements. Computational cost (CPU core-hours) of different quantum refinements for different systems. All **M3** (sole MLP-based) computations were performed on a single CPU, while the other calculations (**M1**, **M5-M10**) were performed on 12/24 Intel(R) Xeon(R) Platinum 9242 CPU. 96
Supplementary Table 38: Real-space Z-difference (RSZD) of the bonded form nirmatrelvir. RSZD scores of the residues around the bonded form nirmatrelvir (4WI) in SARS-CoV-2 main protease from various quantum refinement schemes (**M1-M10**). Those results for X-ray were taken from the experimental structure without our further refinement. 117
Supplementary Table 39: Real-space Z-difference (RSZD) of the bonded and nonbonded forms nirmatrelvir (M1). RSZD scores of the residues around the nirmatrelvir (4WI) in SARS-CoV-2 main protease with different occupations (bonded: nonbonded forms) from quantum refinement using **M1**. Those results for X-ray were taken from the experimental structure without our further refinement. 121
Supplementary Table 40: Real-space Z-difference (RSZD) of the bonded and nonbonded forms nirmatrelvir (M7). RSZD scores of the residues around the nirmatrelvir (4WI) in SARS-CoV-2 main protease with different occupations (bonded: nonbonded forms) from quantum refinement using **M7**. Those results for X-ray were taken from the experimental structure without our further refinement. 123
Supplementary Table 41: Real-space Z-difference (RSZD) of the bonded and nonbonded forms nirmatrelvir (M9). RSZD scores of the residues around the nirmatrelvir (4WI) in SARS-CoV-2 main protease with different occupations (bonded: nonbonded forms) from quantum refinement using **M9**. Those results for X-ray were taken from the experimental structure without our further refinement. 125
Supplementary Table 42: Wild-type SARS-CoV-2 main protease with nirmatrelvir. Reported available structures of wild-type SARS-CoV-2 main protease with nirmatrelvir (link to CYS). Values in parentheses of 7RFW are the results of our quantum refinement for the bonded form by **M7**.. 128

1. General computational details

In order to evaluate the performance and reliability of a few MLPs in our quantum refinements, 50 drug/inhibitor molecules containing only H, C, N, O, F, S, Cl, P, Br elements were selected (QR50 dataset, Supplementary Figs. 1-4). 27 of these molecules comprise only H, C, N, O elements, 20 molecules contain H, C, N, O, F, S, and/or Cl elements, and 3 molecules contain H, C, N, O, S, Br, P elements.

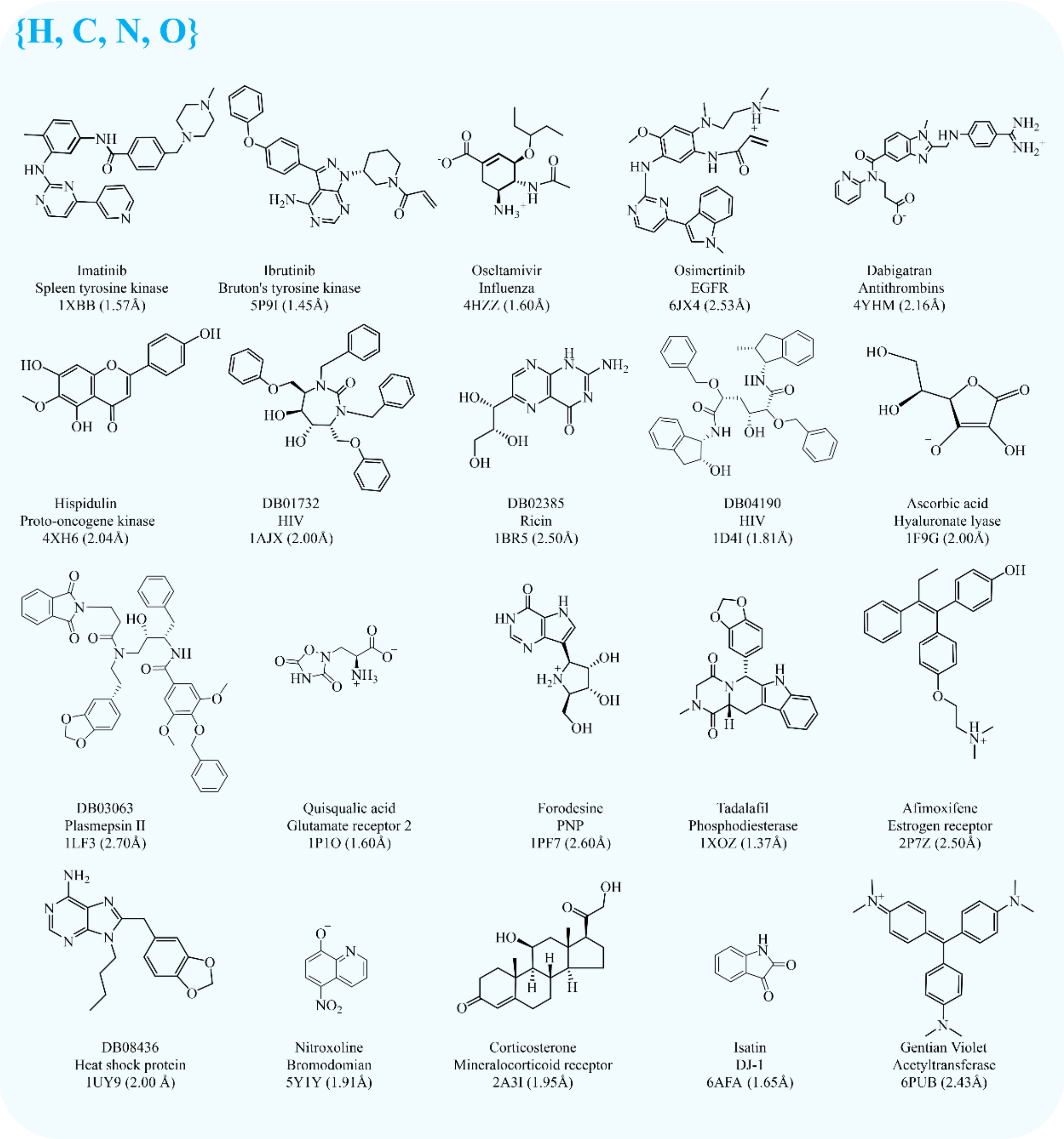

Supplementary Figure 1: Chemical structures of drug/inhibitors (part 1). Chemical structures of our selected 27 drug/inhibitor molecules containing H, C, N, O elements in this study (QR50 dataset). Their drug name, related target protein name and PDB ID are included beneath these structures.

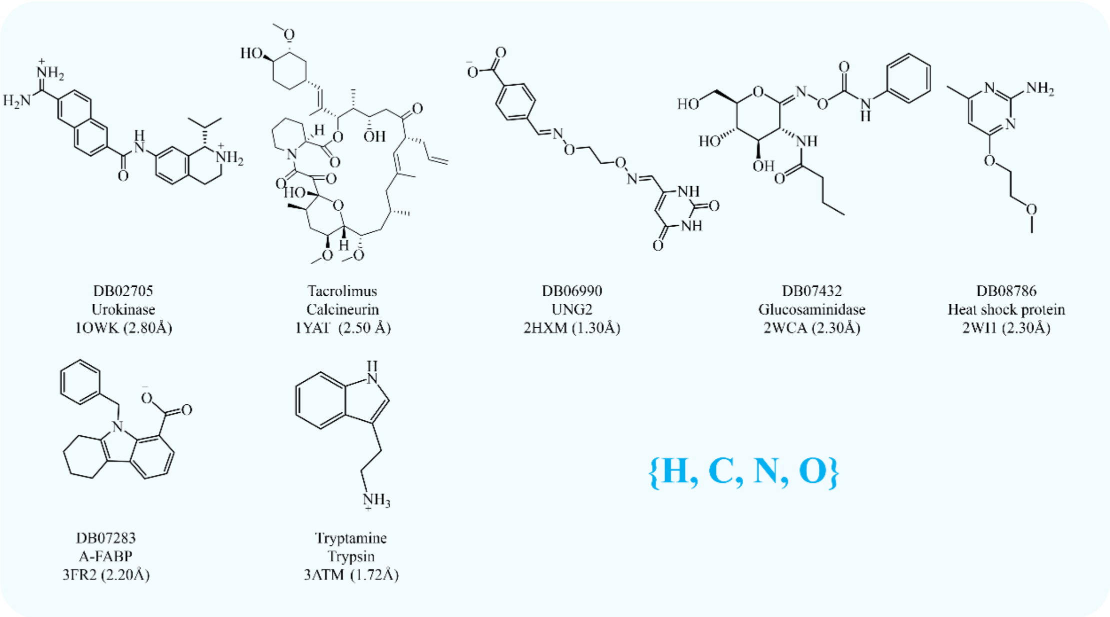

Supplementary Figure 2: Chemical structures of drug/inhibitors (part 1 continued). Chemical structures of our selected 27 drug/inhibitor molecules containing H, C, N, O elements in this study (QR50 dataset). Their drug name, related target protein name and PDB ID are included beneath these structures.

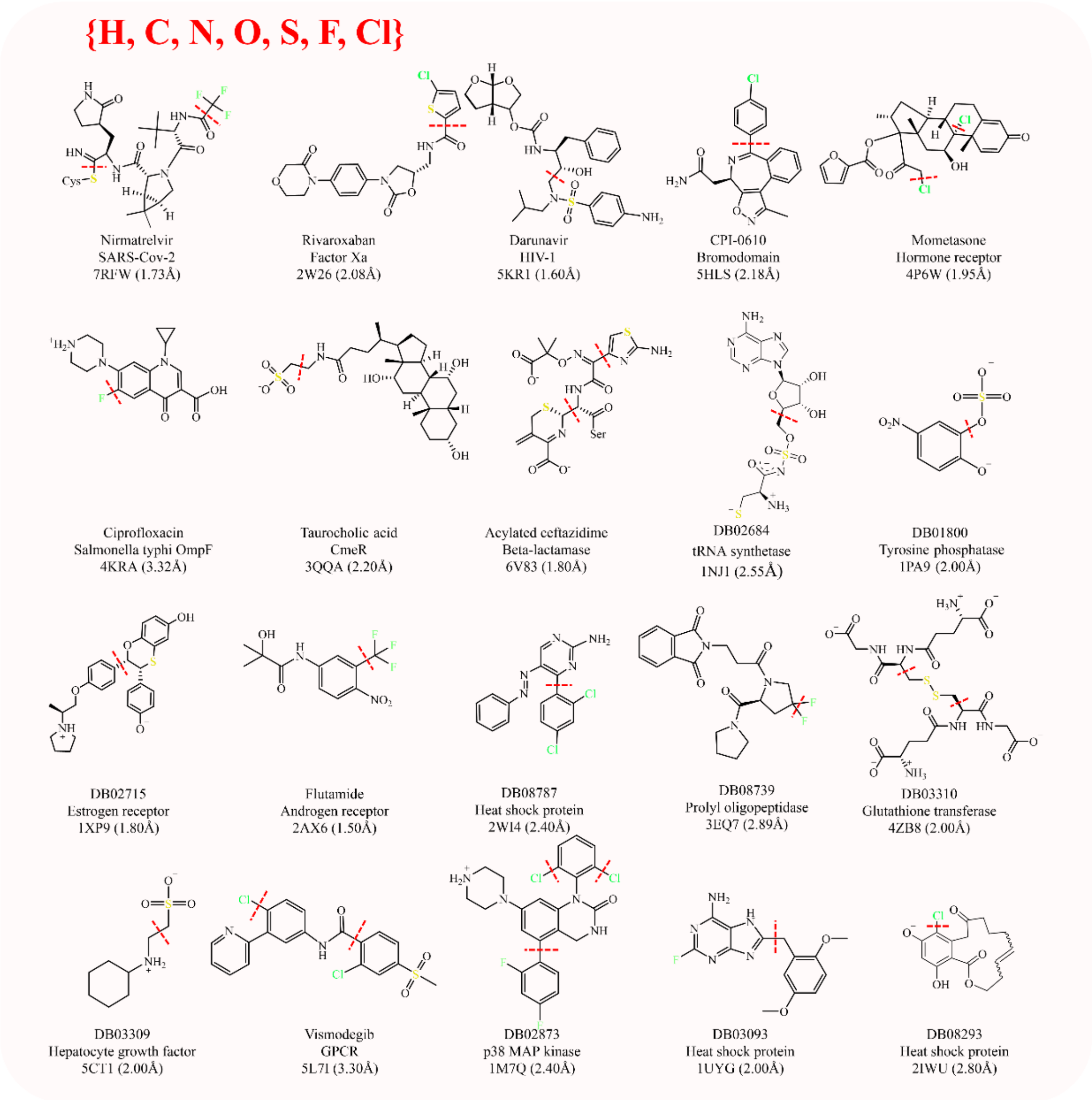

Supplementary Figure 3: Chemical structures of drug/inhibitors (part 2). Chemical structures of our selected 20 drug/inhibitor molecules containing H, C, N, O, F, S, and/or Cl elements in this study (QR50 dataset). Their drug name, related target protein name and PDB ID are included beneath these structures. Dashed lines represent the ONIOM(MLP-CC:MLP-DFT) boundary within the drug/inhibitor molecules containing F, Cl and/or S elements, when AIQM1 or ANI-1ccx was used. The region containing only C, H, O, N is described by AIQM1 or ANI-1ccx, where the rest containing F, Cl and/or S by ANI-2x.

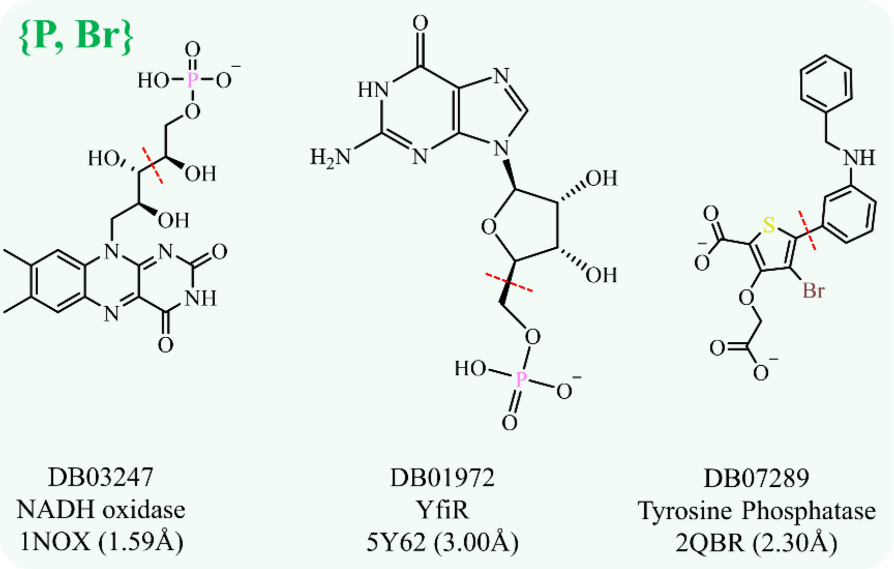

Supplementary Figure 4: Chemical structures of drug/inhibitors (part 3). Chemical structures of our 3 selected drug/inhibitor molecules containing H, C, N, O, S, Br, and/or P elements in this study (QR50 dataset). Their drug name, related target protein name and PDB ID are included beneath these structures. Dashed lines represent the ONIOM2(MLP:SE) boundary within the drug/inhibitor molecules containing P and/or Br elements, when AIQM1 or ANI-2x was used. The region containing only C, H, O, N is described by AIQM1 or ANI-1ccx, where the rest containing P or Br by GFN2-xTB.

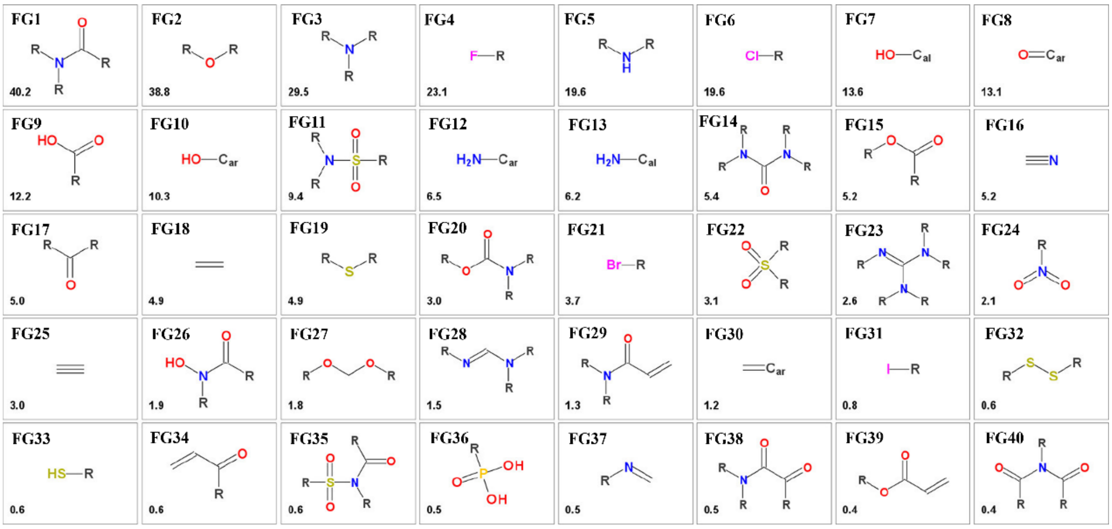

Supplementary Figure 5: Functional groups. The 40 most frequent functional groups occurring in bioactive molecules described reported and adapted from a recent paper.[1] The number indicates the percentage of molecules containing this functional group. R indicates aliphatic or aromatic carbon (Adapted with permission from Ref. 1. Copyright 2020 American Chemical Society.).

Supplementary Table 1: Functional groups in drug/inhibitors. The 40 most frequent functional groups (FGs, Supplementary Figure 5) occurring in our 50 selected drug/inhibitor molecules in this study (QR50 dataset).

FGs	PDB ID	FGs	PDB ID	FGs	PDB ID
**FG1**	1XBB, 5P9I, 4HZZ, 4YHM, 6JX4, 7RF3, 1BR5, 1D4I, 1LF3, 1OWK, 1PF7, 1XOZ, 2WCA, 7RFW, 2W26, 5HLS, 3QQA, 6V83, 2AX6, 3EQ7, 4ZB8, 5L7I	**FG2**	5P9I, 4HZZ, 4XH6, 6JX4, 1AJX, 1D4I, 1LF3, 1YAT, 2P7Z, 2WCA, 2WI1, 1NJ1, 1PA9, 2QBR, 5Y62, 1UYG	**FG3**	1XBB, 4YHM, 6JX4, 2P7Z, 3FR2, 6PUB, 4KRA, 1NOX, 1PA9, 1M7Q
**FG4**	7RFW, 4KRA, 2AX6, 3EQ7, 1M7Q, 1UYG	**FG5**	1XBB, 4YHM, 6JX4, 7RF3, 1OWK, 1PF7, 1XOZ, 3ATM, 4KRA, 2QBR, 5CT1, 1M7Q	**FG6**	2W26, 5HLS, 4P6W, 2QBR, 5L7I, 1M7Q, 2IWU
**FG7**	1AJX, 1BR5, 1D4I, 1FG9, 1LF3, 1PF7, 1YAT, 2A3I, 2WCA, 5KR1, 3QQA, 1NJ1, 1NOX, 2AX6, 5Y62	**FG8**	4XH6, 1BR5, 1PF7, 4KRA	**FG9**	4HZZ, 4YHM, 1P1O, 2HXM, 3FR2, 4KRA, 6V83, 2QBR, 4ZB8
**FG10**	2P7Z, 5Y1Y, 1PA9, 2IWU	**FG11**	5KR1	**FG12**	5P9I, 1UY9, 2WI1, 5KR1, 6V83, 1NJ1, 2QBR, 2UYG
**FG13**	4HZZ, 1P1O, 3ATM, 1NJ1, 44ZB8	**FG14**	1AJX	**FG15**	1FG9, 1YAT, 4P6W, 2IWU
**FG16**	7RFW	**FG17**	2A3I, 4P6W, 2IWU	**FG18**	1YAT, 2P7Z, 6V83, 2IWU
**FG19**	2W26, 6V83, 1PA9, 2QBR	**FG20**	2WCA, 2W26, 5KR1	**FG21**	2QBR
**FG22**	5L7I, 3QQA, 1PA9, 5CT1	**FG23**	1BR5, 5Y62	**FG24**	5Y1Y, 1PA9, 2AX6
**FG25**	None	**FG26**	None	**FG27**	1LF3, 1UY9, 1XOZ, 5KR1
**FG28**	4YHM, 1OWK, 1UY9, 1NJ1, 5Y62, 1UYG	**FG29**	6JX4, 5P9I	**FG30**	6PUB
**FG31**	None	**FG32**	4ZB8	**FG33**	1NJ1
**FG34**	4XH6, 2A3I, 4P6W, 4KRA	**FG35**	1NJ1	**FG36**	1NOX, 5Y62
**FG37**	5HLS, 6V83	**FG38**	1YAT, 6AFA	**FG39**	1F9G
**FG40**	1LF3, 3EQ7, 1M7Q				

All experimental data and protein-drug crystal structures were obtained from the Protein Data Bank (PDB).[2-48] The CNS topology and parameter files of these drug molecules can be generated from PRODRG[49] and ATB[50] servers. Prior to our ONIOM QR calculations, the proteins were first prepared by determining the protonated state and the rotamer of some amino-acid side chains (see below for the setup details in each protein system), as well as addition of hydrogen atoms. The rotamer of the amino-acid side chain was determined by WHATCHECK and our visual examination.[51] The addition of hydrogen atoms and optimization of hydrogen-bond network, repair/addition of the missing atoms (*vide infra*) were then performed using PDB2PQR 3.5.2 program.[52] The protonated state of the titratable residues were assigned on the basis of estimated pKa results computed from PROPKA 3.0 program at the pH of crystallization,[53] while the protonated state of the drug molecules were generally assigned by cross-validation of MolGpka,[54] Graph-pKa[55] and pkasolver[56]. All added

hydrogens were firstly optimized using ONIOM2(DFT:MM) with fixing the heavy atoms.

Various ONIOM-based ONIOM2(QM:MM), ONIOM2(MLP:MM), ONIOM2(SE:MM), ONIOM3(QM:SE:MM), ONIOM3(MLP:SE:MM) and ONIOM4(MLP1: MLP2:SE:MM) schemes were mainly conducted using Gaussian 16.[57] A popular ωB97X-D functionals as the DFT method combined with 6-31G(d) basis sets was employed as the QM method.[58-60] ANI-1ccx (MLP-CC), ANI-1x (MLP-DFT), ANI-2x (MLP-DFT) and AIQM1 (MLP-CC) methods were used as MLPs. GFN2-xTB method was employed as the SE method,[61] as well as Amber ff14SB force fields served as the MM method.[62] The ANI and AIQM1 methods were performed using TorchANI[63] and MLatom,[64,65] respectively, and were called through our Gaussian external interface to achieve novel two-layer ONIOM2(MLP-CC:MLP-DFT) (such as ONIOM2(ANI-1ccx:ANI-2x) and ONIOM2(AIQM1:ANI-2x), eq 1), three-layer ONIOM3(MLP-CC:MLP-DFT:MM) and four-layer ONIOM4(MLP-CC:MLP-DFT:SE:MM) schemes. The ONIOM2(MLP-CC:MLP-DFT) part (eq. 1) was used to replace the high-level QM method in ONIOM2(QM:MM) and ONIOM3(QM:SE:MM) schemes to achieve the abovementioned ONIOM3(MLP-CC:MLP-DFT:MM) and four-layer ONIOM4(MLP-CC:MLP-DFT:SE:MM) schemes, respectively.

$$E_{ONIOM2(MLP-CC:MLP-DFT)} = E_{MLP-CC,model} + E_{MLP-DFT,real} - E_{MLP-DFT,model} \quad (1)$$

For our two- and three-layer ONIOM QR calculations/setup (see below for setup details in each system), the drug molecules (except nirmatrelvir in SARS-CoV-2 M^{pro}, beta-lactamase with ceftazidime and DJ-1 with isatin) were set as the high-layer model part and the optimized region, as well as the medium layer in the three-layer ONIOM methods included neighboring residues within a radius of 3.0 Å to the model part. In the case of the SARS-CoV-2 system, the nirmatrelvir drug and its linkage CYS145 residue were defined as the high-layer model part and optimized region. Similarly, in the case of the beta-lactamase with ceftazidime and DJ-1 with isatin systems, the drug/inhibitor and its covalent linkage residue were defined as the high-layer model part and optimized region. For the systems containing H, C, N, O elements, (see Supplementary Fig. 1-2), **M4**, **M5**, **M9** and **M10** QR schemes were used. Whereas,

for the 20 selected drug/inhibitor systems containing H, C, N, O, F, Cl, S elements (see Supplementary Fig. 3), **M4a**, **M5a**, **M9a** and **M10a** QR schemes were used. ONIOM2(MLP:SE) method was used for the 3 selected molecules containing P and/or Br elements (see Supplementary Fig. 4), when AIQM1 or ANI-2x was used. Furthermore, the highest-methods combination used in this study was considered to be the most reliable computational method: ONIOM3(DFT:SE:MM) (**M7**). All refined results by different methods were generally used to compare with those by **M7**.

Computational chemistry methods in our quantum refinements[a]

	Methods
**M1**	ONIOM2(DFT:MM)
**M2**	ONIOM2-EE(DFT:MM)
**M3**	ONIOM2(ANI-2x:MM)
**M4**	ONIOM2(ANI-1ccx:MM)
**M4a**	ONIOM3(ANI-1ccx:ANI-2x:MM)[b]
**M5**	ONIOM2(AIQM1:MM)[c]
**M5a**	ONIOM3(AIQM1:ANI-2x:MM)[b]
**M6**	ONIOM2(SE:MM)
**M7**	ONIOM3(DFT:SE:MM)
**M8**	ONIOM3(ANI-2x:SE:MM)
**M9**	ONIOM3(ANI-1ccx:SE:MM)
**M9a**	ONIOM4(ANI-1ccx:ANI-2x:SE:MM)[b]
**M10**	ONIOM3(AIQM1:SE:MM)[c]
**M10a**	ONIOM4(AIQM1:ANI-2x:SE:MM)[b]
**M6R**	ONIOM2(SE:MM)[d]

(a). ωB97X-D/6-31G(d) as the density functional theory (DFT) method, ANI-2x (machine learning potentials with DFT accuracy MLP-DFT), ANI-1ccx (machine learning potentials with coupled-cluster accuracy (MLP-CC)) and AIQM1 (MLP-CC) as machine learning potentials (MLPs), Amber ff14SB as the MM method and GFN2-xTB as the SE method. (b). Two machine learning methods were used to describe the drug/inhibitor molecules due to the element limitations (C, H, O, N) of ANI-1ccx and AIQM1. (c) AIQM1 combined with GFN2-xTB methods were used to describe the drug/inhibitor molecules containing P or Br elements due to the element limitations (C, H, O, N) of AIQM1. (d). The high layer of **M6R** includes the drug/inhibitor molecule and neighboring residues within 3Å.

Additionally, optimization of these drug/inhibitor molecules in the gas phase using ANI-2x, ANI-1x, ANI-1ccx, AIQM1, GFN2-xTB and ωB97X-D/6-31G(d) methods as well as ONIOM2(MLP-CC: MLP-DFT) (for the drug/inhibitor molecules (C, H, O, N, F, Cl, S) due to the element limitation (C, H, O, N) in MLP-CC) and ONIOM2(MLP-CC:SE) for the drug/inhibitor molecules (C, H, O, N, F, Cl, S, Br, P) due to the element limitation (C, H, O, N, F, Cl, S) in ANI-2x) were carried out to analyze the MLPs performance using Gaussian 16 as the geometry optimizer.[57] AIQM1 method was computed using MLatom,[64,65] where ODM2[66] computations were conducted using MNDO[67] and DFT-D4[68] methods was used to calculate the dispersion contribution. (SE) GFN2-xTB method was computed using xTB program.[61] Amber ff14SB force fields served as the MM method using "amber=softonly" keyword with additional ff14SB force fields parameters.[62]

On the basis of our previous ONIOM-based QR method, the total energy function of the entire system is given by eq. 2:

$$E_{total} = E_{ONIOM} + \omega_{\alpha} * E_{xray} \quad (2)$$

$$E_{ONIOM2(high:low)} = E_{high,model} + E_{low,real} - E_{low,intermediate} \quad (3)$$

$$E_{ONIOM3(high:medium:low)} = E_{high,model} + E_{medium,intermediate} - E_{medium,model} + E_{low,real} - E_{low,intermediate} \quad (4)$$

where E_{ONIOM} represents the energy contribution from ONIOM-based calculations,[69] E_{xray} stands for the energy contribution derived from the crystallographic penalty and ω_{α} is the weighting factor that balances the contributions of each term. The weighting factor ω_{α} was derived from CNS for each system.[70,71] Eqs. 3 and 4 represent the two- and three-layer ONIOM schemes, respectively. The related gradient functions can be analogously derived and used for geometry optimization. The optimization convergence conditions (atomic unit) for our QR calculations were kept consistent with the default settings in Gaussian 16: $\Delta E < 10^{-5}$, $Step_{max} < 1.8\times10^{-2}$, $Step_{RMS} < 1.2\times10^{-2}$, $\nabla E_{max} < 4.5\times10^{-3}$, $\nabla E_{RMS} < 3\times10^{-3}$.

RESP (Restrained Electrostatic Potential)[72] charges calculated at B3LYP/6-31G(d,p) level and GAFF's atom types and parameters were applied for our drug/inhibitor molecules.

Our refined result analysis (including electron density, and real-space difference density Z score (RSZD)) were carried out using Refmac5,[73] and Edstats[74] modules implemented in CCP4i2 8.0.[75] Electron density maps were drawn using Pymol.[76] The strain energy was determined by calculating the QM energy difference between the fully-optimized ligand in the gas phase and the refined ligand extracted from the protein at the ωB97X-D/6-31G(d) level. The list of all bond distances, angles and dihedrals were generated through Gaussian 16's internal coordination. In addition, the rotatable dihedrals were determined using AutoDockTools 1.5.6.[77]

References:

1. Ertl P., Altmann E., McKenna J. M. The Most Common Functional Groups in

Bioactive Molecules and How Their Popularity Has Evolved over Time. *J. Med. Chem.* **63**, 8408-8418 (2020).
2. Berman H. M., *et al.* The Protein Data Bank. *Acta Crystallogr., Sect. D: Struct. Biol.* **58**, 899-907 (2002).
3. Atwell S., *et al.* A novel mode of Gleevec binding is revealed by the structure of spleen tyrosine kinase. *J. Biol. Chem.* **279**, 55827-55832 (2004).
4. Bender A. T., *et al.* Ability of Bruton's Tyrosine Kinase Inhibitors to Sequester Y551 and Prevent Phosphorylation Determines Potency for Inhibition of Fc Receptor but not B-Cell Receptor Signaling. *Mol. Pharmacol.* **91**, 208-219 (2017).
5. Li Q., *et al.* Functional and Structural Analysis of Influenza Virus Neuraminidase N3 Offers Further Insight into the Mechanisms of Oseltamivir Resistance. *J. Virol.* **87**, 10016-10024 (2013).
6. Yan X. E., *et al.* Structural Basis of AZD9291 Selectivity for EGFR T790M. *J. Med. Chem.* **63**, 8502-8511 (2020).
7. Owen D. R., *et al.* An oral SARS-CoV-2 M-pro inhibitor clinical candidate for the treatment of COVID-19. *Science* **374**, 1586-1593 (2021).
8. Roehrig S., *et al.* Discovery of the novel antithrombotic agent 5-chloro-N-({(5S)-2-oxo-3 4-(3-oxomorpholin-4-yl)phenyl -1,3-oxazolidin- 5-yl}methyl)thiophene-2-carboxamide (BAY 59-7939): An oral, direct factor Xa inhibitor. *J. Med. Chem.* **48**, 5900-5908 (2005).
9. Liu Z. L., *et al.* Effects of Hinge-region Natural Polymorphisms on Human Immunodeficiency Virus-Type 1 Protease Structure, Dynamics, and Drug Pressure Evolution. *J. Biol. Chem.* **291**, 22741-22756 (2016).
10. Albrecht B. K., *et al.* Identification of a Benzoisoxazoloazepine Inhibitor (CPI-0610) of the Bromodomain and Extra-Terminal (BET) Family as a Candidate for Human Clinical Trials. *J. Med. Chem.* **59**, 1330-1339 (2016).
11. He Y. Z., *et al.* Structures and mechanism for the design of highly potent glucocorticoids. *Cell Res.* **24**, 713-726 (2014).
12. Rotonda J., Burbaum J. J., Chan H. K., Marcy A. I., Becker J. W. Improved calcineurin inhibition by yeast FKBP12-drug complexes. Crystallographic and functional analysis. *J. Biol. Chem.* **268**, 7607-7609 (1993).
13. Hecht H. J., Erdmann H., Park H. J., Sprinzl M., Schmid R. D. Crystal structure of NADH oxidase from Thermus thermophilus. *Nat. Struct. Biol.* **2**, 1109-1114 (1995).
14. Bäckbro K., *et al.* Unexpected Binding Mode of a Cyclic Sulfamide HIV-1 Protease Inhibitor. *J. Med. Chem.* **40**, 898-902 (1997).
15. Yan X., *et al.* Structure-based identification of a ricin inhibitor. *J. Mol. Biol.* **266**, 1043-1049 (1997).
16. Li S., Taylor K. B., Kelly S. J., Jedrzejas M. J. Vitamin C inhibits the enzymatic activity of Streptococcus pneumoniae hyaluronate lyase. *J. Biol. Chem.* **276**, 15125-15130 (2001).
17. Andersson H. O., *et al.* Optimization of P1-P3 groups in symmetric and asymmetric HIV-1 protease inhibitors. *Eur. J. Biochem.* **270**, 1746-1758 (2003).
18. Armstrong N., Mayer M., Gouaux E. Tuning activation of the AMPA-sensitive GluR2 ion channel by genetic adjustment of agonist-induced conformational changes. *Proc. Natl. Acad. Sci. U. S. A.* **100**, 5736-5741 (2003).
19. Asojo O. A., *et al.* Novel uncomplexed and complexed structures of plasmepsin II, an aspartic protease from Plasmodium falciparum. *J. Mol. Biol.* **327**, 173-181 (2003).
20. Filgueira de Azevedo W., Jr., *et al.* Structural basis for inhibition of human PNP by immucillin-H. *Biochem. Biophys. Res. Commun.* **309**, 917-922 (2003).
21. Kamtekar S., Kennedy W. D., Wang J., Stathopoulos C., Söll D., Steitz T. A. The structural basis of cysteine aminoacylation of tRNAPro by prolyl-tRNA synthetases. *Proc. Natl. Acad. Sci. U. S. A.* **100**, 1673-1678 (2003).
22. Stelmach J. E., *et al.* Design and synthesis of potent, orally bioavailable dihydroquinazolinone inhibitors of p38 MAP kinase. *Bioorg. Med. Chem. Lett.* **13**, 277-280 (2003).

23. Sun J. P., Wu L., Fedorov A. A., Almo S. C., Zhang Z. Y. Crystal structure of the Yersinia protein-tyrosine phosphatase YopH complexed with a specific small molecule inhibitor. *J. Biol. Chem.* **278**, 33392-33399 (2003).
24. Card G. L., *et al.* Structural basis for the activity of drugs that inhibit phosphodiesterases. *Structure* **12**, 2233-2247 (2004).
25. Wendt M. D., *et al.* Identification of novel binding interactions in the development of potent, selective 2-naphthamidine inhibitors of urokinase. Synthesis, structural analysis, and SAR of N-phenyl amide 6-substitution. *J. Med. Chem.* **47**, 303-324 (2004).
26. Wright L., *et al.* Structure-activity relationships in purine-based inhibitor binding to HSP90 isoforms. *Chem. Biol.* **11**, 775-785 (2004).
27. Blizzard T. A., *et al.* Estrogen receptor ligands. Part 9: Dihydrobenzoxathiin SERAMs with alkyl substituted pyrrolidine side chains and linkers. *Bioorg. Med. Chem. Lett.* **15**, 107-113 (2005).
28. Bohl C. E., Miller D. D., Chen J., Bell C. E., Dalton J. T. Structural basis for accommodation of nonsteroidal ligands in the androgen receptor. *J. Biol. Chem.* **280**, 37747-37754 (2005).
29. Li Y., Suino K., Daugherty J., Xu H. E. Structural and biochemical mechanisms for the specificity of hormone binding and coactivator assembly by mineralocorticoid receptor. *Mol. Cell* **19**, 367-380 (2005).
30. Krosky D. J., Bianchet M. A., Seiple L., Chung S., Amzel L. M., Stivers J. T. Mimicking damaged DNA with a small molecule inhibitor of human UNG2. *Nucleic Acids Res.* **34**, 5872-5879 (2006).
31. Proisy N., *et al.* Inhibition of Hsp90 with synthetic macrolactones: synthesis and structural and biological evaluation of ring and conformational analogs of radicicol. *Chem. Biol.* **13**, 1203-1215 (2006).
32. Wilson D. P., *et al.* Structure-based optimization of protein tyrosine phosphatase 1B inhibitors: from the active site to the second phosphotyrosine binding site. *J. Med. Chem.* **50**, 4681-4698 (2007).
33. Abad M. C., *et al.* Structural determination of estrogen-related receptor gamma in the presence of phenol derivative compounds. *J. Steroid Biochem. Mol. Biol.* **108**, 44-54 (2008).
34. Kánai K., *et al.* Prolyl oligopeptidase inhibition by N-acyl-pro-pyrrolidine-type molecules. *J. Med. Chem.* **51**, 7514-7522 (2008).
35. Balcewich M. D., *et al.* Insight into a strategy for attenuating AmpC-mediated beta-lactam resistance: structural basis for selective inhibition of the glycoside hydrolase NagZ. *Protein Sci.* **18**, 1541-1551 (2009).
36. Barf T., *et al.* N-Benzyl-indolo carboxylic acids: Design and synthesis of potent and selective adipocyte fatty-acid binding protein (A-FABP) inhibitors. *Bioorg. Med. Chem. Lett.* **19**, 1745-1748 (2009).
37. Brough P. A., *et al.* Combining hit identification strategies: fragment-based and in silico approaches to orally active 2-aminothieno[2,3-d]pyrimidine inhibitors of the Hsp90 molecular chaperone. *J. Med. Chem.* **52**, 4794-4809 (2009).
38. Lei H. T., *et al.* Crystal structures of CmeR-bile acid complexes from Campylobacter jejuni. *Protein Sci.* **20**, 712-723 (2011).
39. Yamane J., *et al.* In-crystal affinity ranking of fragment hit compounds reveals a relationship with their inhibitory activities. *J. Appl. Crystallogr.* **44**, 798-804 (2011).
40. Chao S.-W., Su M.-Y., Chiou L.-C., Chen L.-C., Chang C.-I., Huang W.-J. Total Synthesis of Hispidulin and the Structural Basis for Its Inhibition of Proto-oncogene Kinase Pim-1. *J. Nat. Prod.* **78**, 1969-1976 (2015).
41. Roret T., Thuillier A., Favier F., Gelhaye E., Didierjean C., Morel-Rouhier M. Evolutionary divergence of Ure2pA glutathione transferases in wood degrading fungi. *Fungal Genet. Biol.* **83**, 103-112 (2015).
42. Schiele F., van Ryn J., Litzenburger T., Ritter M., Seeliger D., Nar H. Structure-guided residence time optimization of a dabigatran reversal agent. *mAbs* **7**, 871-880 (2015).
43. Sigurdardottir A. G., *et al.* Exploring the chemical space of the lysine-binding pocket

of the first kringle domain of hepatocyte growth factor/scatter factor (HGF/SF) yields a new class of inhibitors of HGF/SF-MET binding. *Chem. Sci.* **6**, 6147-6157 (2015).
44. Byrne E. F. X., *et al.* Structural basis of Smoothened regulation by its extracellular domains. *Nature* **535**, 517-522 (2016).
45. Jiang H., *et al.* Discovery of novel BET inhibitors by drug repurposing of nitroxoline and its analogues. *Org. Biomol. Chem.* **15**, 9352-9361 (2017).
46. Zhou L., Xu M., Jiang T. Structural insights into the functional role of GMP in modulating the YfiBNR system. *Biochem. Biophys. Res. Commun.* **493**, 637-642 (2017).
47. Tashiro S., *et al.* Discovery and Optimization of Inhibitors of the Parkinson's Disease Associated Protein DJ-1. *ACS Chem. Biol.* **13**, 2783-2793 (2018).
48. Brown C. A., *et al.* Antagonism between substitutions in β-lactamase explains a path not taken in the evolution of bacterial drug resistance. *J. Biol. Chem.* **295**, 7376-7390 (2020).
49. Schuttelkopf A. W., van Aalten D. M. F. PRODRG: a tool for high-throughput crystallography of protein-ligand complexes. *Acta Crystallogr., Sect. D: Struct. Biol.* **60**, 1355-1363 (2004).
50. Stroet M., Caron B., Visscher K. M., Geerke D. P., Malde A. K., Mark A. E. Automated Topology Builder Version 3.0: Prediction of Solvation Free Enthalpies in Water and Hexane. *J. Chem. Theory Comput.* **14**, 5834-5845 (2018).
51. Hooft R. W. W., Vriend G., Sander C., Abola E. E. Errors in protein structures. *Nature* **381**, 272-272 (1996).
52. Unni S., *et al.* Web Servers and Services for Electrostatics Calculations with APBS and PDB2PQR. *J. Comput. Chem.* **32**, 1488-1491 (2011).
53. Olsson M. H. M., Søndergaard C. R., Rostkowski M., Jensen J. H. PROPKA3: Consistent Treatment of Internal and Surface Residues in Empirical pKa Predictions. *J. Chem. Theory Comput.* **7**, 525-537 (2011).
54. Pan X. L., Wang H., Li C. Y., Zhang J. Z. H., Ji C. G. MolGpka: A Web Server for Small Molecule pK(a) Prediction Using a Graph-Convolutional Neural Network. *J. Chem. Inf. Model.* **61**, 3159-3165 (2021).
55. Xiong J. C., *et al.* Multi-instance learning of graph neural networks for aqueous pK(a) prediction. *Bioinformatics* **38**, 792-798 (2022).
56. Mayr F., Wieder M., Wieder O., Langer T. Improving Small Molecule pK(a) Prediction Using Transfer Learning With Graph Neural Networks. *Front. Chem.* **10**, 866585 (2022).
57. Gaussian 16 Rev. A.03 (Wallingford, CT, 2016).
58. Chai J. D., Head-Gordon M. Systematic optimization of long-range corrected hybrid density functionals. *J. Chem. Phys.* **128**, 084106 (2008).
59. Ditchfield R., Hehre W. J., Pople J. A. Self-Consistent Molecular-Orbital Methods .9. Extended Gaussian-Type Basis for Molecular-Orbital Studies of Organic Molecules. *J. Chem. Phys.* **54**, 724-728 (1971).
60. Harihara.Pc, Pople J. A. The influence of polarization functions on molecular orbital hydrogenation energies. *Theor. Chim. Acta* **28**, 213-222 (1973).
61. Bannwarth C., Ehlert S., Grimme S. GFN2-xTB-An Accurate and Broadly Parametrized Self-Consistent Tight-Binding Quantum Chemical Method with Multipole Electrostatics and Density-Dependent Dispersion Contributions. *J. Chem. Theory Comput.* **15**, 1652-1671 (2019).
62. Maier J. A., Martinez C., Kasavajhala K., Wickstrom L., Hauser K. E., Simmerling C. ff14SB: Improving the Accuracy of Protein Side Chain and Backbone Parameters from ff99SB. *J. Chem. Theory Comput.* **11**, 3696-3713 (2015).
63. Gao X., Ramezanghorbani F., Isayev O., Smith J. S., Roitberg A. E. TorchANI: A Free and Open Source PyTorch-Based Deep Learning Implementation of the ANI Neural Network Potentials. *J. Chem. Inf. Model.* **60**, 3408-3415 (2020).
64. Dral P. O. MLatom: A program package for quantum chemical research assisted by machine learning. *J. Comput. Chem.* **40**, 2339-2347 (2019).

65. Dral P. O., *et al.* MLatom 2: An Integrative Platform for Atomistic Machine Learning. *Top. Curr. Chem.* **379**, 27 (2021).
66. Dral P. O., Wu X., Thiel W. Semiempirical Quantum-Chemical Methods with Orthogonalization and Dispersion Corrections. *J. Chem. Theory Comput.* **15**, 1743-1760 (2019).
67. MNDO2020 (Max-Planck-Institut fur Kohlenforschung, Mulheim an der Ruhr, 2020).
68. Caldeweyher E., Bannwarth C., Grimme S. Extension of the D3 dispersion coefficient model. *J. Chem. Phys.* **147**, 034112 (2017).
69. Chung L. W., Hirao H., Li X., Morokuma K. The ONIOM method: its foundation and applications to metalloenzymes and photobiology. *Wiley Interdiscip. Rev.: Comput. Mol. Sci.* **2**, 327-350 (2012).
70. Brunger A. T. Version 1.2 of the Crystallography and NMR system. *Nat. Protoc.* **2**, 2728-2733 (2007).
71. Brunger A. T., *et al.* Crystallography & NMR system: A new software suite for macromolecular structure determination. *Acta Crystallogr., Sect. D: Biol. Crystallogr.* **54**, 905-921 (1998).
72. Bayly C. I., Cieplak P., Cornell W. D., Kollman P. A. A Well-Behaved Electrostatic Potential Based Method Using Charge Restraints for Deriving Atomic Charges - the Resp Model. *J. Phys. Chem.* **97**, 10269-10280 (1993).
73. Murshudov G. N., *et al.* REFMAC5 for the refinement of macromolecular crystal structures. *Acta Crystallogr., Sect. D: Struct. Biol.* **67**, 355-367 (2011).
74. Tickle I. J. Statistical quality indicators for electron-density maps. *Acta Crystallogr., Sect. D: Biol. Crystallogr.* **68**, 454-467 (2012).
75. Potterton L., *et al.* CCP4i2: the new graphical user interface to the CCP4 program suite. *Acta Crystallogr., Sect. D: Struct. Biol.* **74**, 68-84 (2018).
76. The PyMOL Molecular Graphics System, Version 2.0 (2015).
77. Morris G. M., *et al.* AutoDock4 and AutoDockTools4: Automated Docking with Selective Receptor Flexibility. *J. Comput. Chem.* **30**, 2785-2791 (2009).

2. Result analysis

Supplementary Table 2: Comparison of different density functional theory (DFT) methods in the gas phase. Median absolute deviation (MAD), root mean square distance (RMSD) and Correlation (R^2) of the optimized bond distances, angles, rotatable dihedrals for the selected 10 drugs/inhibitors (shown in Fig. 1) based on optimization in the gas phase using different functionals and basis sets compared to the DFT (ωB97X/6-31G(d)) method.

		ωB97X-D/6-31+G(d)	ωB97X-D/6-31G(d)	M06-2x/6-31G(d)
Bonds (Å)	MAD	0.001	0.001	0.001
	RMSD	0.004	0.003	0.004
	R^2	1.000	1.000	1.000
Angles (º)	MAD	0.2	0.1	0.2
	RMSD	0.4	0.3	0.3
	R^2	0.997	0.998	0.998
Dihedrals (º)	MAD	16	13	7
	RMSD	65	59	43
	R^2	0.661	0.718	0.846

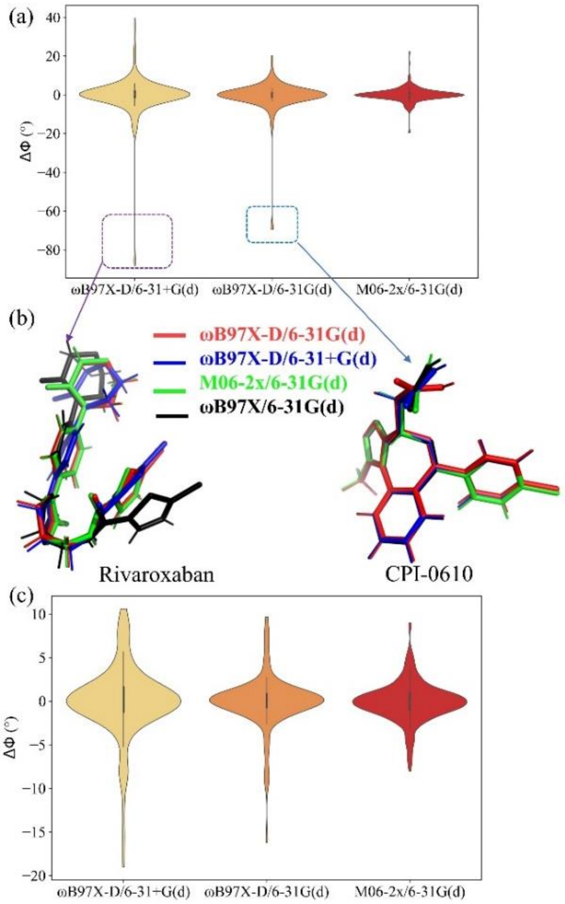

Supplementary Figure 6: Comparison of different density functional theory (DFT) methods in the gas phase. (**a**) Violin plot of derivations of the rotatable dihedrals (Δϕ, n = 259) of the selected 10 drug/inhibitor (shown in Fig. 1) (**b**) Supposition of rivaroxaban and CPI-0610 (shows outliers of violin plot) and (**c**) Violin plot of derivations of the rotatable dihedrals (Δϕ, n = 234) without rivaroxaban and CPI-0610 based on optimization in the gas phase using different functionals and basis sets compared to the DFT (ωB97X-D/6-31G(d)) method. Inner boxplots indicate interquartile range

Supplementary Table 3: Comparison of different density functional theory (DFT) methods in quantum refinement. Median absolute deviation (MAD), root mean square distance (RMSD) and Correlation (R^2) of the refined bond distances (n = 390), angles (n = 562), rotatable dihedrals (n=259) for the selected 10 drugs/inhibitors (shown in Fig. 1b) based on **M1** quantum refinements using different functionals and basis sets compared to the DFT (ωB97X/6-31G(d)) method.

		ωB97X-D/6-31+G(d)	ωB97X-D/6-31G(d)	M06-2x/6-31G(d)
Bonds (Å)	MAD	0.001	0.000	0.001
	RMSD	0.002	0.002	0.002
	R^2	1.000	1.000	1.000
Angles (º)	MAD	0.1	0.0	0.1
	RMSD	0.2	0.1	0.2
	R^2	0.999	1.000	0.999
Dihedrals (º)	MAD	0.3	0.0	0.2
	RMSD	0.6	0.0	0.5
	R^2	1.000	1.000	1.000

2.1 Drugs/inhibitors in the gas phase
Test on selected 10 drug/inhibitor (shown in Fig. 1**)**

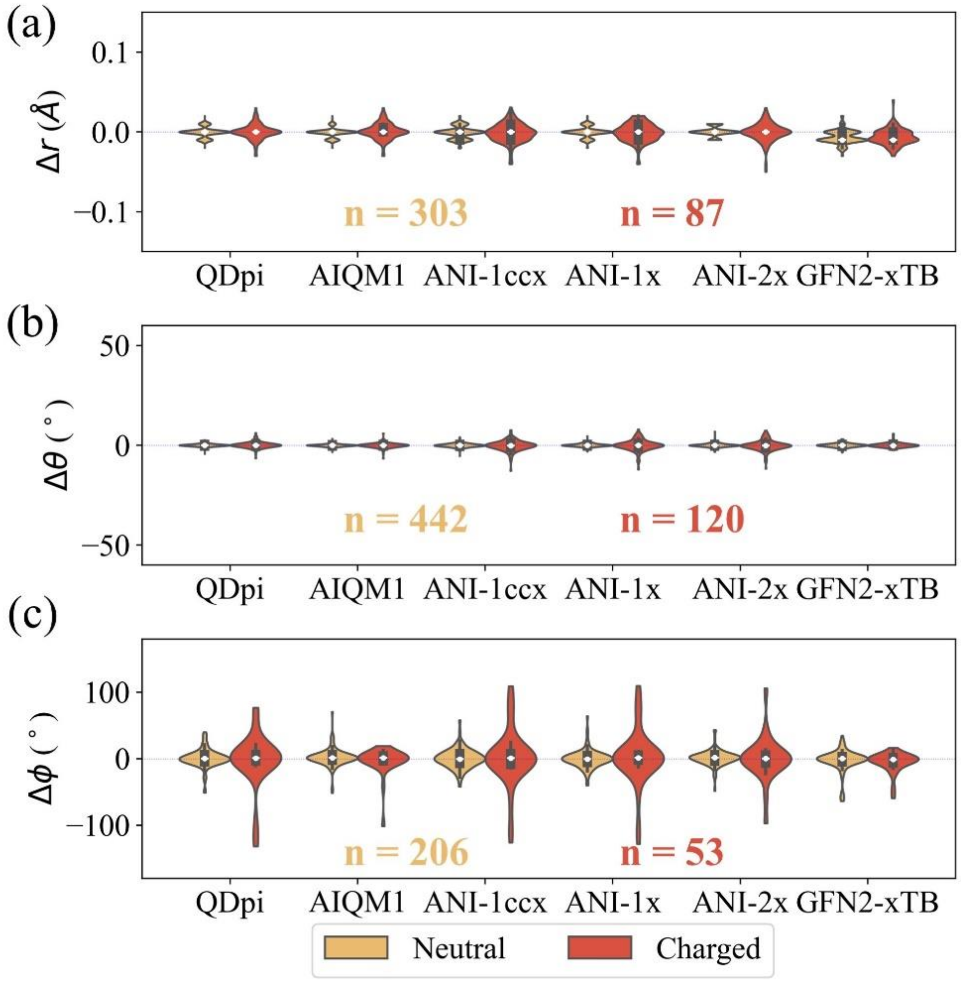

Supplementary Figure 7 Comparison of the optimized structures in gas phase. Violin plot of derivations of the refined (**a**) bond distances (Δr), (**b**) angles (Δθ), (**c**) rotatable dihedrals (Δϕ) of the selected 10 drug/inhibitor (shown in Fig. 1) structures optimized in the gas phase (neutral and charged groups), using QDpi, AIQM1, ANI-1ccx, ANI-2x, ANI-1x and GFN2-xTB methods compared to the DFT (ωB97X-D/6-31G(d)) method. ONIOM2(MLP:ANI-2x) method was used for the molecules containing F, Cl and/or S elements, when AIQM1, QDpi, ANI-1ccx or ANI-1x was used. The number of data was marked in each plot. The white dots indicate the median values and inner boxplots indicate interquartile range.

Supplementary Table 4: Comparison of the optimized structures (all systems) in gas phase. Correlation (R^2) and median absolute deviation (MAD) of the refined bond distances (n = 390), angles (n = 560), rotatable dihedrals (n = 259) for the selected 10 drug/inhibitor (shown in Fig. 1) based on optimization in the gas phase using AIQM1, ANI-1ccx, ANI-2x, ANI-1x and GFN2-xTB methods compared to the DFT (ωB97X-D/6-31G(d)) method. ONIOM2(MLP:ANI-2x) method was used for the molecules containing F, Cl and/or S elements, when AIQM1, QDpi, ANI-1ccx or ANI-1x was used.

		QDpi	AIQM1	ANI-1ccx	ANI-1x	ANI-2x	GFN2-xTB
	Bond	0.995	0.996	0.993	0.994	0.995	0.989
**R^2**	Angle	0.981	0.986	0.960	0.959	0.960	0.980
	Rotatable Dihedral	0.182	0.345	0.297	0.435	0.333	0.655
	Bond (Å)	0.004	0.004	0.006	0.005	0.004	0.008
**MAD**	Angle (°)	0.64	0.57	0.92	0.85	0.84	0.73
	Rotatable Dihedral (°)	38.51	30.39	36.35	30.06	32.07	20.45

Supplementary Table 5: Comparison of the optimized structures (neutral cases) in gas phase. Correlation (R^2) and median absolute deviation (MAD) of the refined bond distances (n = 303), angles (n = 442), rotatable dihedrals (n = 206) for the neutral cases of the selected 10 drug/inhibitor (shown in Fig. 1) based on optimization in the gas phase using AIQM1, ANI-1ccx, ANI-2x, ANI-1x and GFN2-xTB methods compared to the DFT (ωB97X-D/6-31G(d)) method. ONIOM2(MLP:ANI-2x) method was used for the molecules containing F, Cl and/or S elements, when AIQM1, QDpi, ANI-1ccx or ANI-1x was used.

		QDpi	AIQM1	ANI-1ccx	ANI-1x	ANI-2x	GFN2-xTB
	Bond	0.997	0.997	0.995	0.996	0.997	0.991
**R^2**	Angle	0.989	0.991	0.983	0.987	0.986	0.983
	Rotatable Dihedral	0.224	0.319	0.330	0.471	0.309	0.620
	Bond (Å)	0.004	0.003	0.005	0.004	0.004	0.008
**MAD**	Angle (°)	0.54	0.50	0.70	0.60	0.60	0.71
	Rotatable Dihedral (°)	35.26	31.07	33.22	26.84	31.16	21.63

Supplementary Table 6: Comparison of the optimized structures (charged cases) in gas phase. Correlation (R^2) and median absolute deviation (MAD) of the refined bond distances (n = 87), angles (n = 120), rotatable dihedrals (n = 53) for the charged cases of the selected 10 drug/inhibitor (shown in Fig. 1b) based on optimization in the gas phase using AIQM1, ANI-1ccx, ANI-2x, ANI-1x and GFN2-xTB methods compared to the DFT (ωB97X-D/6-31G(d)) method. ONIOM2(MLP:ANI-2x) method was used for the molecules containing F, Cl and/or S elements, when AIQM1, QDpi, ANI-1ccx or ANI-1x was used.

		QDpi	AIQM1	ANI-1ccx	ANI-1x	ANI-2x	GFN2-xTB
	Bond	0.987	0.986	0.979	0.980	0.980	0.979
**R^2**	Angle	0.937	0.957	0.835	0.807	0.818	0.966
	Rotatable Dihedral	0.022	0.443	0.171	0.298	0.423	0.790
	Bond (Å)	0.005	0.006	0.008	0.008	0.007	0.009
**MAD**	Angle (°)	1.03	0.81	1.72	1.77	1.71	0.78
	Rotatable Dihedral (°)	51.16	27.74	48.53	42.55	35.65	15.85

Computations of the selected 50 systems (Supplementary Figs. 1-4) in the gas phase

Supplementary Table 7: Comparison of the optimized structures (all systems) in the gas phase. Correlation (R^2) and median absolute deviation (MAD) of the refined bond distances (n = 1595), angles (n = 2271), rotatable dihedrals (n = 881) for the selected 50 drugs/inhibitors (QR50) based on optimization in the gas phase using AIQM1, ANI-2x, and GFN2-xTB methods compared to the DFT (ωB97X-D/6-31G(d)) method. ONIOM2(MLP:ANI-2x) method was used for the molecules containing F, Cl and/or S elements, when AIQM1 was used. ONIOM2(MLP:SE) method was used for the molecules containing P and/or Br elements, when AIQM1 or ANI-2x was used.

		**AIQM1**	**ANI-2x**	**GFN2-xTB**
**R^2**	Bond	0.994	0.988	0.988
	Angle	0.977	0.916	0.968
	Rotatable Dihedral	0.953	0.932	0.955
**MAD**	Bond (Å)	0.005	0.006	0.008
	Angle (°)	0.6	0.9	0.8
	Rotatable Dihedral (°)	11.6	16.1	11.2

Supplementary Table 8: Comparison of the optimized structure (neutral cases) in the gas phase. Correlation (R^2) and median absolute deviation (MAD) of the refined bond distances (n = 891), angles (n = 1278), rotatable dihedrals (n = 473) for the neutral cases out of the selected 50 drugs/inhibitors (QR50) based on optimization in the gas phase using AIQM1, ANI-2x, and GFN2-xTB methods compared to the DFT (ωB97X-D/6-31G(d)) method. ONIOM2(MLP:ANI-2x) method was used for the molecules containing F, Cl and/or S elements, when AIQM1 was used. ONIOM2(MLP:SE) method was used for the molecules containing P and/or Br elements, when AIQM1 or ANI-2x was used.

		**AIQM1**	**ANI-2x**	**GFN2-xTB**
**R^2**	Bond	0.995	0.996	0.990
	Angle	0.986	0.982	0.976
	Rotatable Dihedral	0.962	0.954	0.961
**MAD**	Bond (Å)	0.004	0.003	0.007
	Angle (°)	0.5	0.6	0.7
	Rotatable Dihedral (°)	10.6	12.0	10.6

Supplementary Table 9: Comparison of the optimized structures (charged cases) in the gas phase. Correlation (R^2) and median absolute deviation (MAD) of the refined bond distances (n = 704), angles (n = 993), rotatable dihedrals (n = 408) for the charged cases out of the selected 50 drugs/inhibitors (QR50) based on optimization in the gas phase using AIQM1, ANI-2x, and GFN2-xTB methods compared to the DFT (ωB97X-D/6-31G(d)) method. ONIOM2(MLP:ANI-2x) method was used for the molecules containing F, Cl and/or S elements, when AIQM1 was used. ONIOM2(MLP:SE) method was used for the molecules containing P and/or Br elements, when AIQM1 or ANI-2x was used.

		**AIQM1**	**ANI-2x**	**GFN2-xTB**
**R^2**	Bond	0.992	0.979	0.985
	Angle	0.966	0.826	0.957
	Rotatable Dihedral	0.943	0.907	0.949
**MAD**	Bond (Å)	0.006	0.009	0.010
	Angle (°)	0.7	1.3	0.9
	Rotatable Dihedral (°)	12.7	21.0	11.9

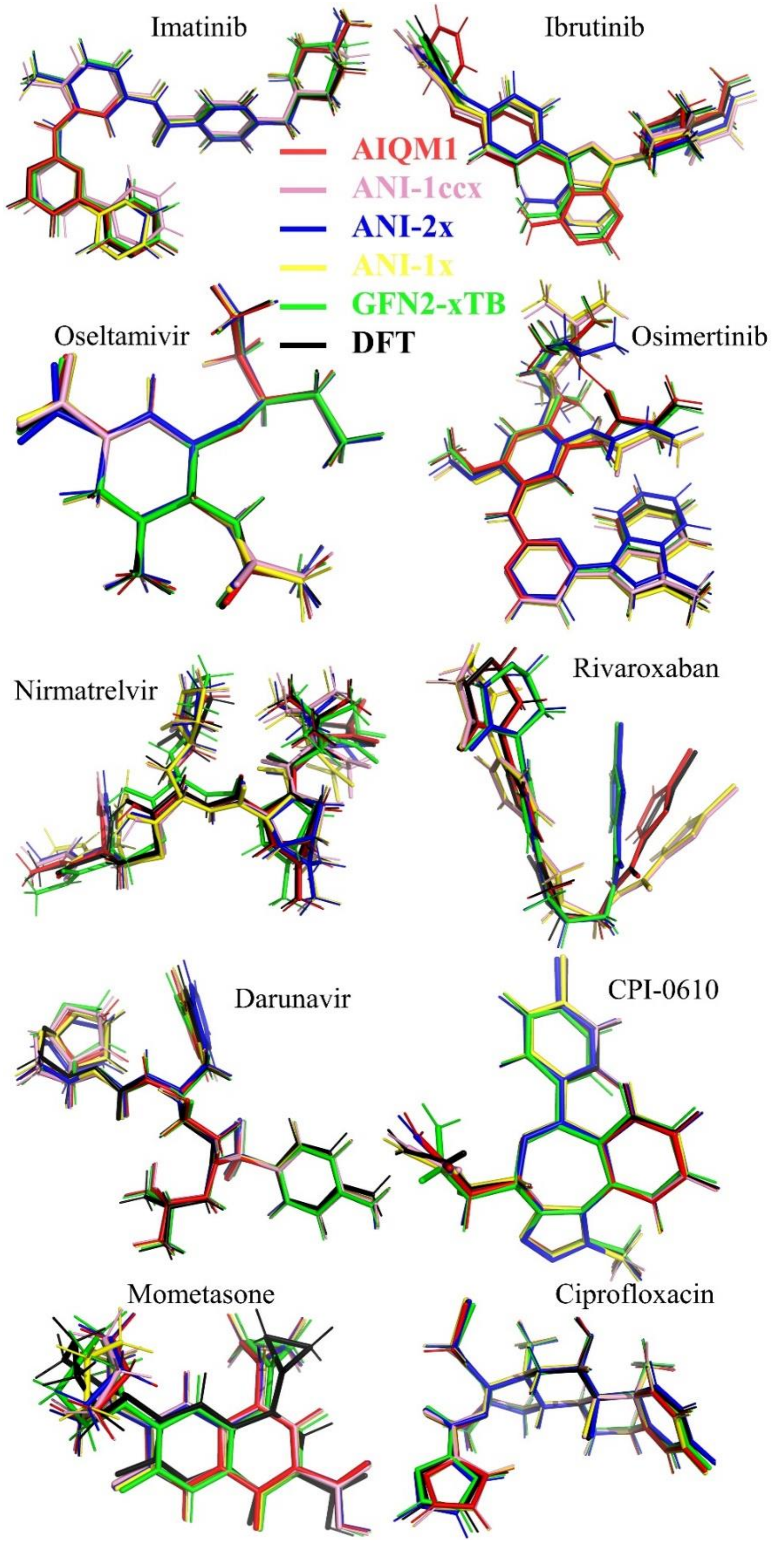

Supplementary Figure 8: Optimized structures in the gas phase. Structure superimpositions of the selected 10 drugs/inhibitors (shown in Fig. 1) optimized in the gas phase using the AIQM1 (red), ANI-1ccx (pink), ANI-2x (blue), ANI-1x (yellow) and GFN2-xTB (green) and DFT (ωB97X-D/6-31G(d), black) methods.

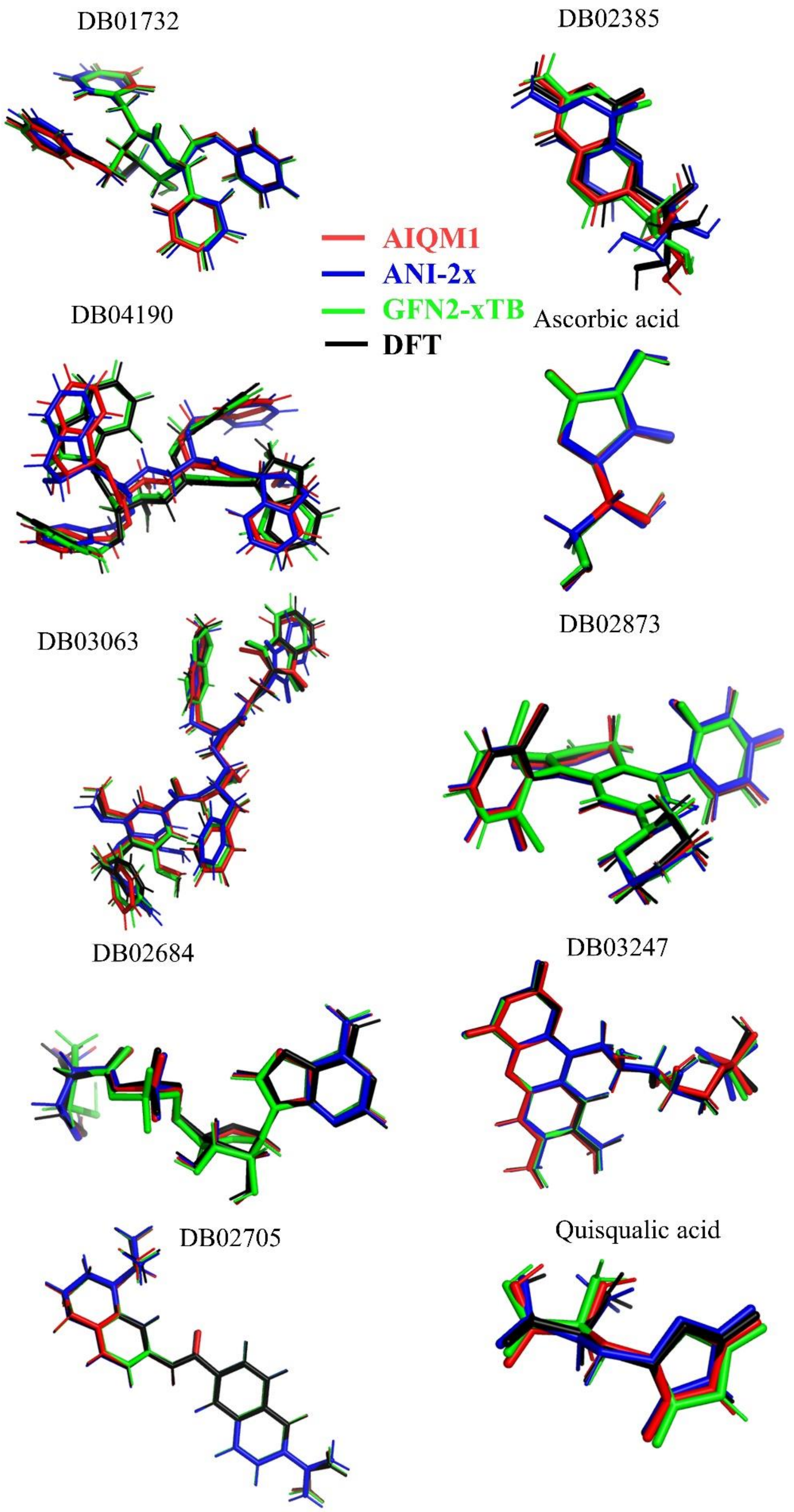

Supplementary Figure 9: Optimized structures in the gas phase. Structure superimpositions of the selected 50 drugs/inhibitors (shown in Supplementary Figs. 1-4) optimized in the gas phase using the AIQM1 (red), ANI-2x (blue), and GFN2-xTB (green) and DFT (ωB97X-D/6-31G(d), black) methods.

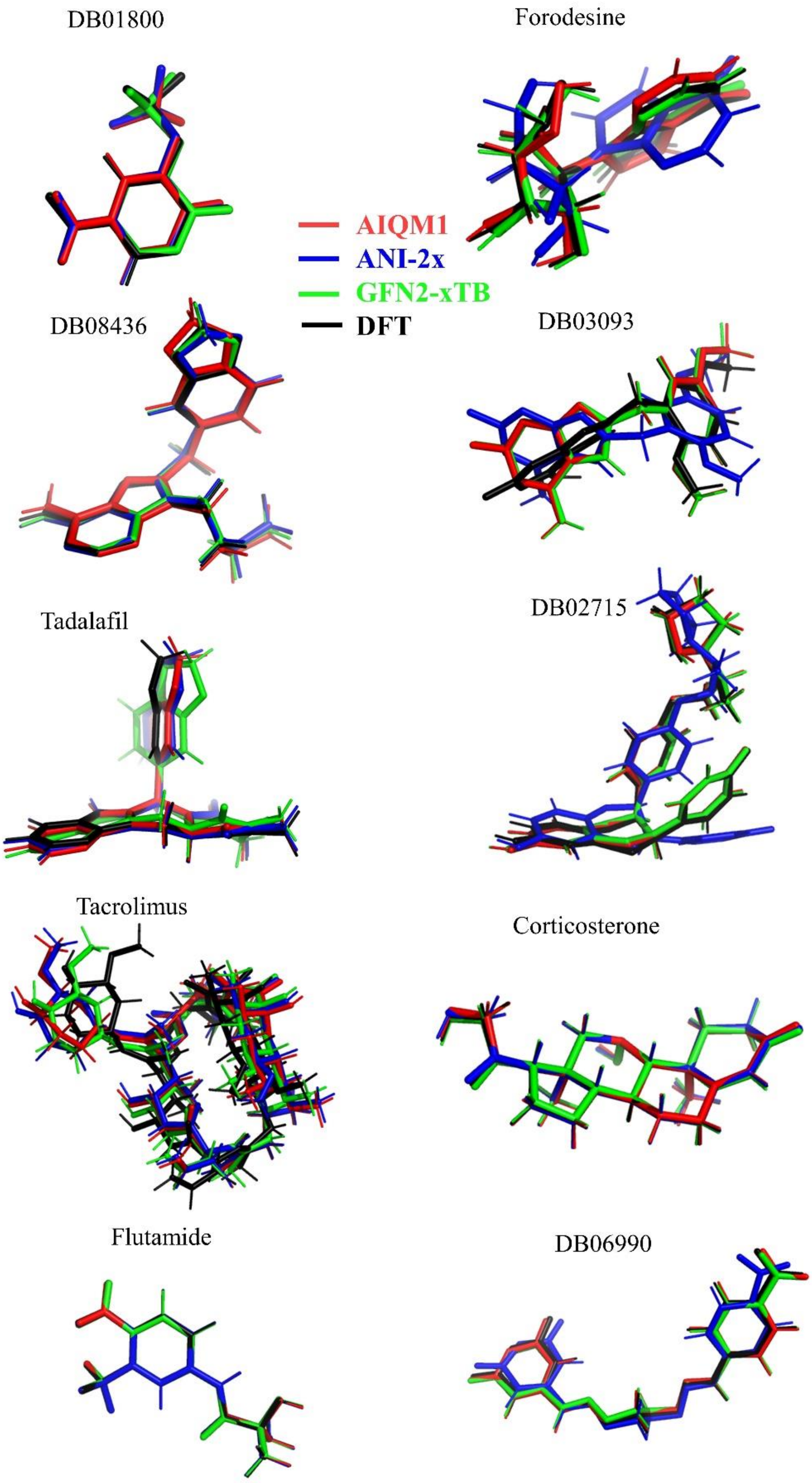

Supplementary Figure 9 (Continued): Optimized structures in the gas phase. Structure superimpositions of the selected 50 drugs/inhibitors (shown in Supplementary Figs. 1-4) optimized in the gas phase using the AIQM1 (red), ANI-2x (blue), and GFN2-xTB (green) and DFT (ωB97X-D/6-31G(d), black) methods.

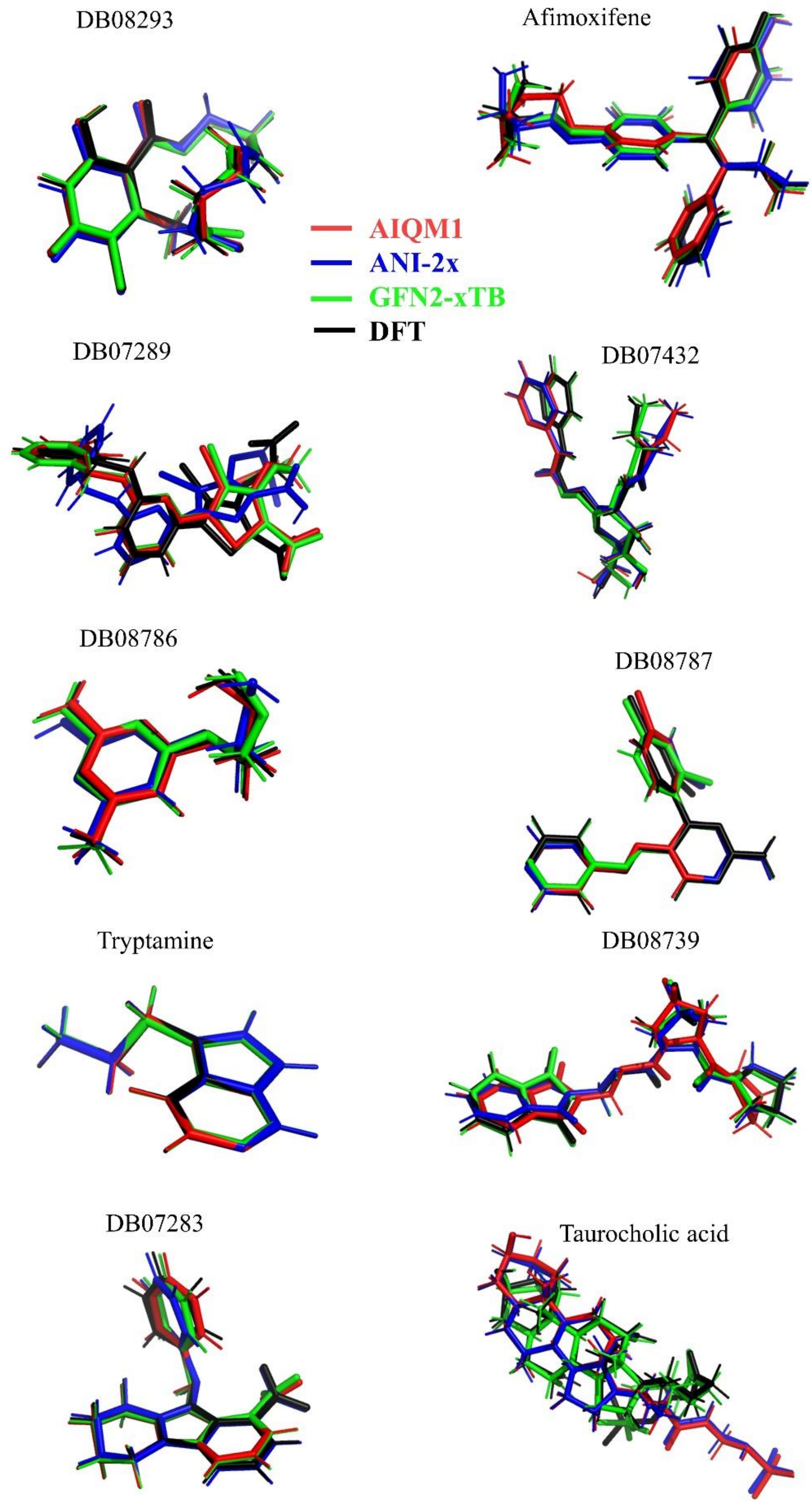

Supplementary Figure 9 (Continued): Optimized structures in the gas phase. Structure superimpositions of the selected 50 drugs/inhibitors (shown in Supplementary Figs. 1-4) optimized in the gas phase using the AIQM1 (red), ANI-2x (blue), and GFN2-xTB (green) and DFT (ωB97X-D/6-31G(d), black) methods.

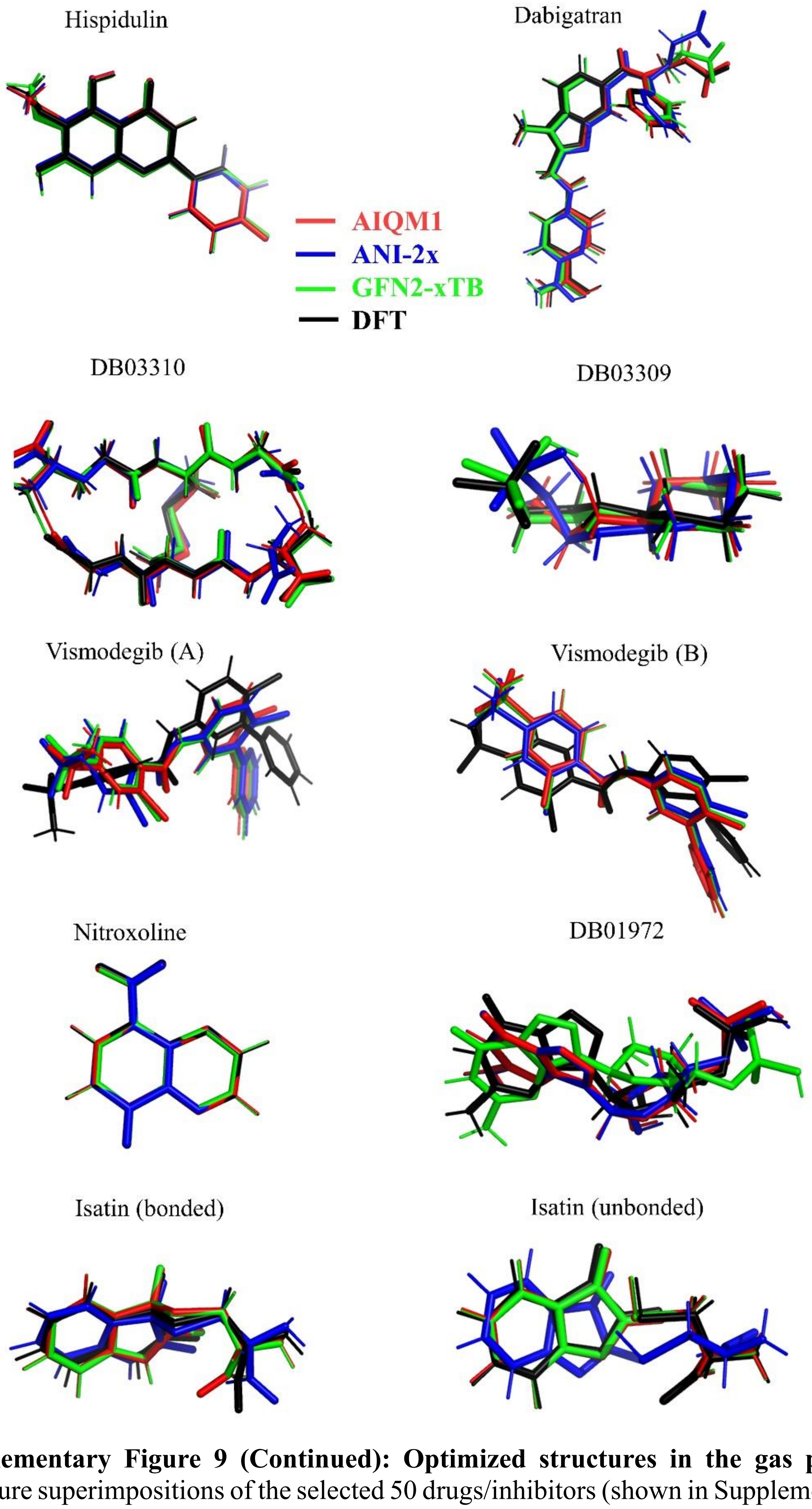

Supplementary Figure 9 (Continued): Optimized structures in the gas phase. Structure superimpositions of the selected 50 drugs/inhibitors (shown in Supplementary Figs. 1-4) optimized in the gas phase using the AIQM1 (red), ANI-2x (blue), and GFN2-xTB (green) and DFT (ωB97X-D/6-31G(d), black) methods.

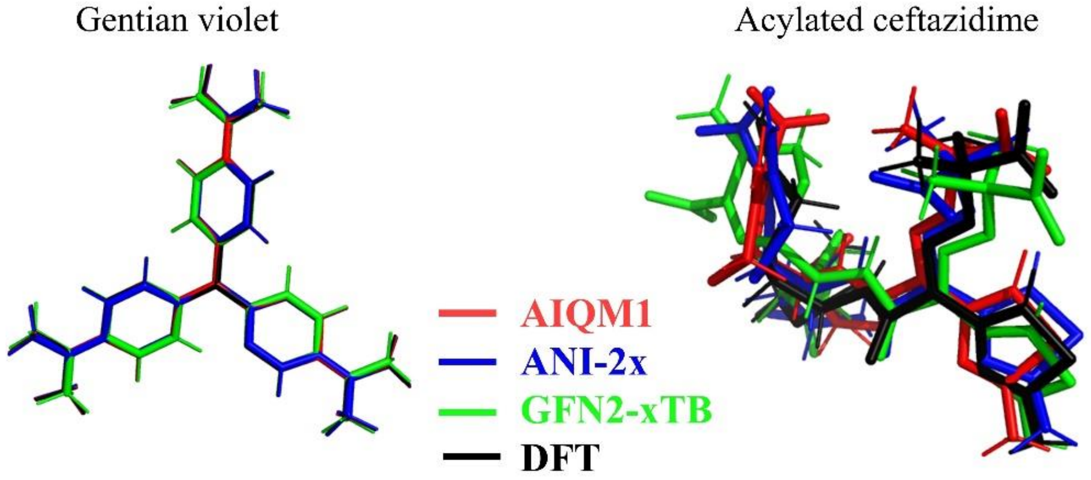

Supplementary Figure 9 (Continued): Optimized structures in the gas phase. Structure superimpositions of the selected 50 drugs/inhibitors (shown in Supplementary Figs. 1-4) optimized in the gas phase using the AIQM1 (red), ANI-2x (blue), and GFN2-xTB (green) and DFT (ωB97X-D/6-31G(d), black) methods.

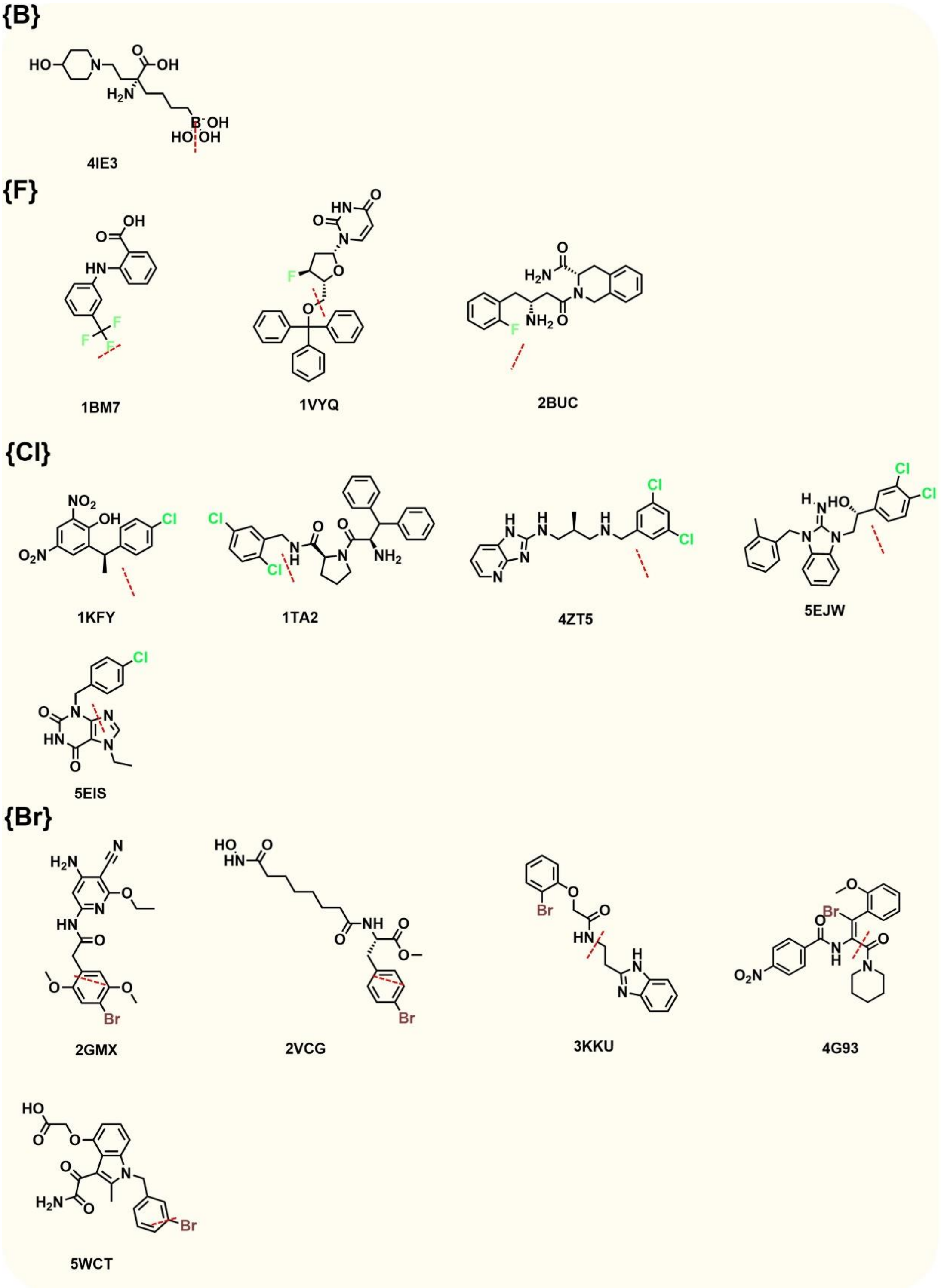

Supplementary Figure 10: Chemical structures of some drug/inhibitors in PB20-QM-3k. Chemical structures of the drug/inhibitor molecules in PB20-QM-3k dataset containing elements beyond H, C, N, O elements in this study. Dashed lines represent the ONIOM boundary with the drug/inhibitor molecules containing F, Cl and/or S elements, when ONIOM2(MLP:ANI-2x) method was used for the 15 molecules containing F, Cl and/or S elements, when AIQM1 was used. ONIOM2(MLP:SE) method was used for the 16 molecules containing B, P, Se, Br and/or I elements, when AIQM1 or ANI-2x was used.

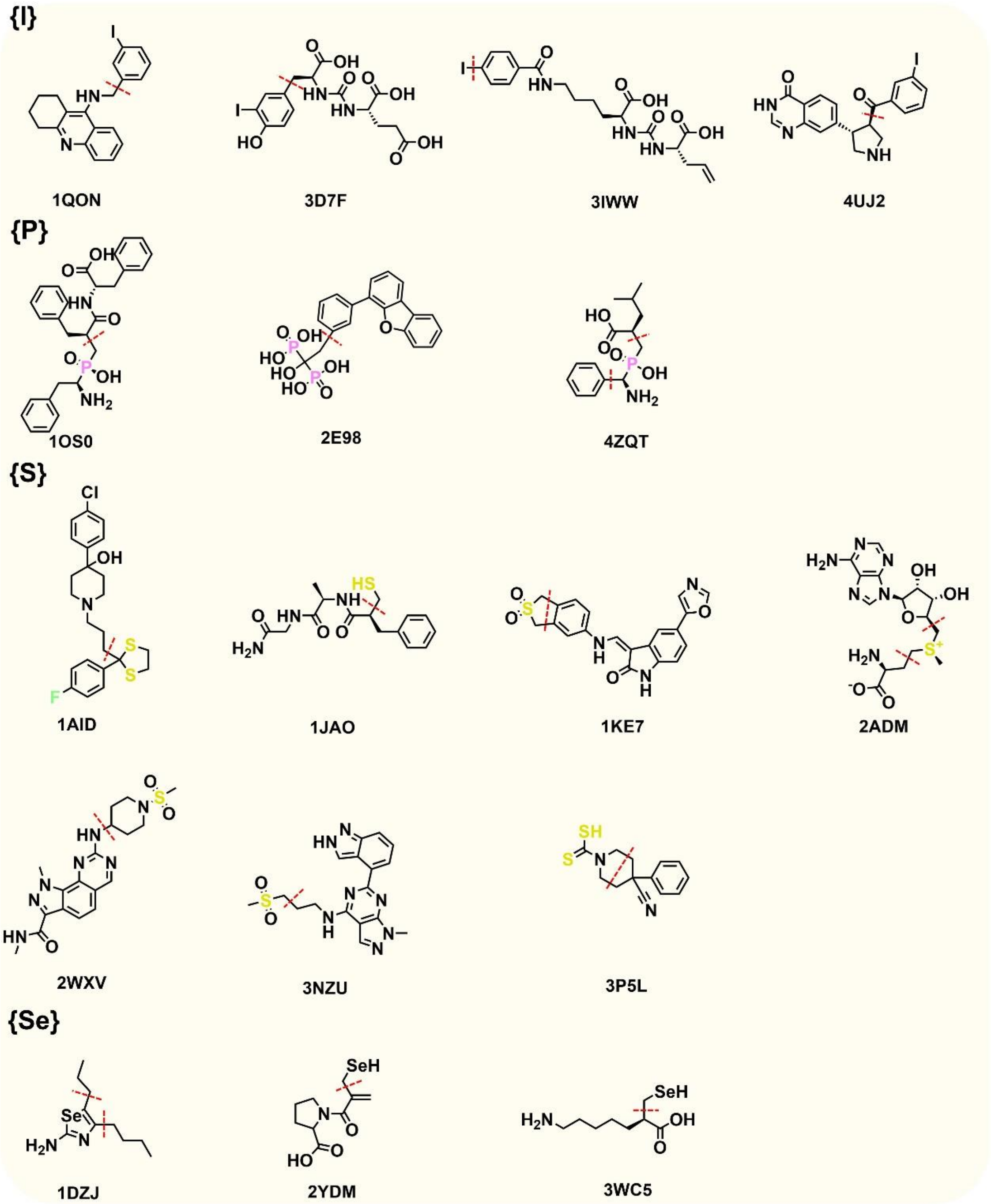

Supplementary Figure 11: Chemical structures of some drug/inhibitors in PB20-QM-3k). Chemical structures of the drug/inhibitor molecules in PB20-QM-3k dataset containing elements beyond H, C, N, O elements in this study. Dashed lines represent the ONIOM boundary with the drug/inhibitor molecules containing F, Cl and/or S elements, when ONIOM2(MLP:ANI-2x) method was used for the 15 molecules containing F, Cl and/or S elements, when AIQM1 was used. ONIOM2(MLP:SE) method was used for the 16 molecules containing B, P, Se, Br and/or I elements, when AIQM1 or ANI-2x was used.

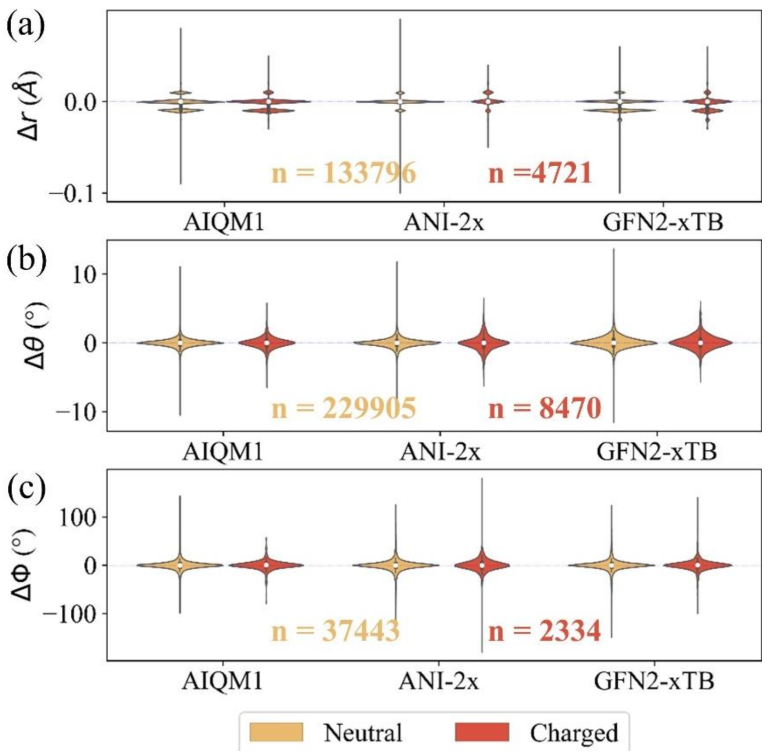

Supplementary Figure 12: Comparison of the optimized structures in the gas phase (PB20-QM-3k). Violin plots of deviation in (**a**) bond distances (Δr), (**b**) angles (Δθ) and (**c**) rotatable dihedrals (Δϕ) for the 3156 drug/inhibitor structures (PB20-QM-3k dataset) optimized in the gas phase, using MLPs (MLP-CC: AIQM1, MLP-DFT: ANI-2x) and the (SE) GFN2-xTB method compared to the (QM) ωB97X-D/6-31G(d) method. ONIOM2(MLP:ANI-2x) method was used for the 15 molecules containing F, Cl and/or S elements, when AIQM1 was used. ONIOM2(MLP:SE) method was used for the 16 molecules containing B, P, Se, Br and/or I elements, when AIQM1 or ANI-2x was used. The white dots in violin plots indicate the median values and inner boxplots indicate interquartile range. The number of data was marked in each plot.

Supplementary Table 10: Comparison of the optimized structures in the gas phase (PB20-QM-3k). Correlation (R^2) and median absolute deviation (MAD) of the refined bond distances (n = 138517), angles (n = 238375), rotatable dihedrals (n = 39777) for the 3156 drug/inhibitor structures (PB20-QM-3k dataset) optimized in the gas phase, using MLPs (MLP-CC: AIQM1, MLP-DFT: ANI-2x) and the (SE) GFN2-xTB method compared to the (QM) ωB97X-D/6-31G(d) method. ONIOM2(MLP:ANI-2x) method was used for the molecules containing F, Cl and/or S elements, when AIQM1 was used. ONIOM2(MLP:SE) method was used for the molecules containing B, P, Se, Br and/or I elements, when AIQM1 or ANI-2x was used.

		**AIQM1**	**ANI-2x**	**GFN2-xTB**
**R^2**	Bond	0.999	0.999	0.998
	Angle	0.991	0.989	0.982
	Rotatable Dihedral	0.421	0.304	0.405
**MAD**	Bond (Å)	0.004	0.003	0.006
	Angle (°)	0.4	0.5	0.6
	Rotatable Dihedral (°)	26.7	32.0	28.0

Supplementary Table 11: Comparison of the optimized structures in the gas phase (neutral cases in PB20-QM-3k). Correlation (R^2) and median absolute deviation (MAD) of the refined bond distances (n = 133796), angles (n = 229905), rotatable dihedrals (n = 37443) for the neutral cases out of the 3156 drug/inhibitor structures (PB20-QM-3k dataset) optimized in the gas phase, using MLPs (MLP-CC: AIQM1, MLP-DFT: ANI-2x) and the (SE) GFN2-xTB method compared to the (QM) ωB97X-D/6-31G(d) method. ONIOM2(MLP:ANI-2x) method was used for the molecules containing F, Cl and/or S elements, when AIQM1 was used. ONIOM2(MLP:SE) method was used for the molecules containing B, P, Se, Br and/or I elements, when AIQM1 or ANI-2x was used.

		**AIQM1**	**ANI-2x**	**GFN2-xTB**
**R^2**	Bond	0.999	0.999	0.998
	Angle	0.992	0.989	0.983
	Rotatable Dihedral	0.409	0.293	0.391
**MAD**	Bond (Å)	0.004	0.003	0.006
	Angle (°)	0.4	0.4	0.6
	Rotatable Dihedral (°)	27.2	32.5	28.6

Supplementary Table 12: Comparison of the optimized structures in the gas phase (charged cases in PB20-QM-3k). Correlation (R^2) and median absolute deviation (MAD) of the refined bond distances (n = 4721), angles (n = 8470), rotatable dihedrals (n = 2334) for the charged cases out of the 3156 drug/inhibitor structures (PB20-QM-3k dataset) optimized in the gas phase, using MLPs (MLP-CC: AIQM1, MLP-DFT: ANI-2x) and the (SE) GFN2-xTB method compared to the (QM) ωB97X-D/6-31G(d) method. ONIOM2(MLP:ANI-2x) method was used for the molecules containing F, Cl and/or S elements, when AIQM1 was used. ONIOM2(MLP:SE) method was used for the molecules containing B, P, Se, Br and/or I elements, when AIQM1 or ANI-2x was used.

		**AIQM1**	**ANI-2x**	**GFN2-xTB**
**R^2**	Bond	0.999	0.999	0.998
	Angle	0.980	0.964	0.966
	Rotatable Dihedral	0.629	0.487	0.634
**MAD**	Bond (Å)	0.005	0.004	0.005
	Angle (°)	0.6	0.8	0.8
	Rotatable Dihedral (°)	18.2	25.0	18.5

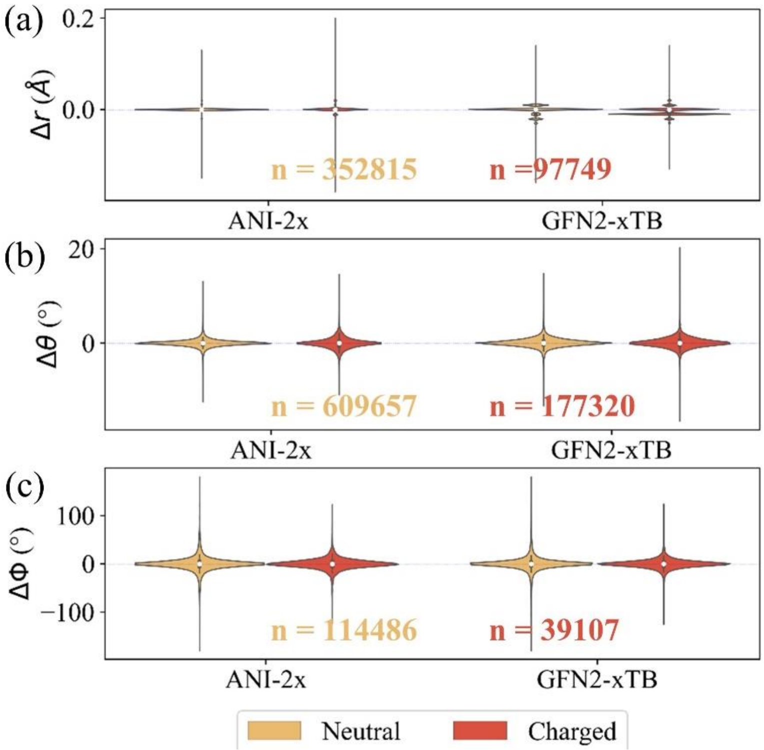

Supplementary Figure 13: Comparison of the optimized structures in the gas phase (PB20-QM-8k). Violin plots of deviation in (**a**) bond distances (Δr), (**b**) angles (Δθ) and (**c**) rotatable dihedrals (Δϕ) for the 8776 drug/inhibitor structures (PB20-QM-8k dataset) optimized in the gas phase, using ANI-2x and the (SE) GFN2-xTB method compared to the (QM) ωB97X-D/6-31G(d) method. The white dots in violin plots indicate the median values and inner boxplots indicate interquartile range. The number of data was marked in each plot.

Supplementary Table 13: Comparison of the optimized structures in the gas phase (PB20-QM-8k). Correlation (R^2) and median absolute deviation (MAD) of the refined bond distances (n = 450564), angles (n = 786977), rotatable dihedrals (n = 153593) for the 8776 drug/inhibitor structures (PB20-QM-8k dataset) optimized in the gas phase, using ANI-2x and the (SE) GFN2-xTB method compared to the (QM) ωB97X-D/6-31G(d) method.

		**ANI-2x**	**GFN2-xTB**
	Bond	0.999	0.998
**R^2**	Angle	0.981	0.977
	Rotatable Dihedral	0.316	0.401
	Bond (Å)	0.003	0.006
**MAD**	Angle (°)	0.6	0.7
	Rotatable Dihedral (°)	32.6	29.0

Supplementary Table 14: Comparison of the optimized structures in the gas phase (neutral cases in PB20-QM-8k). Correlation (R^2) and median absolute deviation (MAD) of the refined bond distances (n = 352815), angles (n = 609657), rotatable dihedrals (n = 114486) for the neutral cases out of the 8776 drug/inhibitor structures (PB20-QM-8k dataset) optimized in the gas phase, using ANI-2x and the (SE) GFN2-xTB method compared to the (QM) ωB97X-D/6-31G(d) method.

		**ANI-2x**	**GFN2-xTB**
**R^2**	Bond	0.999	0.998
	Angle	0.985	0.978
	Rotatable Dihedral	0.261	0.347
**MAD**	Bond (Å)	0.003	0.006
	Angle (°)	0.5	0.7
	Rotatable Dihedral (°)	35.2	31.6

Supplementary Table 15: Comparison of the optimized structures in the gas phase (charged cases in PB20-QM-3k). Correlation (R^2) and median absolute deviation (MAD) of the refined bond distances (n = 97749), angles (n = 177320), rotatable dihedrals (n = 39107) for the charged cases out of the 8776 drug/inhibitor structures (PB20-QM-8k dataset) optimized in the gas phase, using ANI-2x and the (SE) GFN2-xTB method compared to the (QM) ωB97X-D/6-31G(d) method.

		**ANI-2x**	**GFN2-xTB**
**R^2**	Bond	0.998	0.998
	Angle	0.963	0.968
	Rotatable Dihedral	0.480	0.565
**MAD**	Bond (Å)	0.004	0.006
	Angle (°)	0.8	0.8
	Rotatable Dihedral (°)	25.9	21.5

2.2 Drugs/inhibitors in the proteins

Test on the 10 selected drug/inhibitor (shown in Fig. 1)

MLPs-CC (**M4-M5** and **M9-M10**) can produce the lowest RSZD scores among our ONIOM2- and ONIOM3-based quantum refinements for imatinib, ibrutinib, osimertinib and darunavir systems (shown in Supplementary Fig. 14). The ANI-2x-based schemes (**M3** and **M8**) yield identical RSZD scores as the DFT methods (**M1** and **M7**) for the oseltamivir and CPI-0610 systems. On the other hand, the AIQM1 (**M5** and **M10**) and ANI-2x based schemes (**M3** and **M8**) exhibit the lowest strain energies for the imatinib, ibrutinib and darunavir, CPI-0610, mometasone systems, respectively (Supplementary Table 5). As the ANI series potentials were not developed to describe charged systems, the ANI-2x (**M3** and **M8**) and ANI-1ccx (**M4** and **M9**) based schemes give very large strain energies for the drugs containing charged group(s). However, they still exhibit very similar RSZD scores to the DFT-based schemes (**M1-M2** and **M7**) for the three charged cases (oseltamivir, osimertinib and ciprofloxacin).

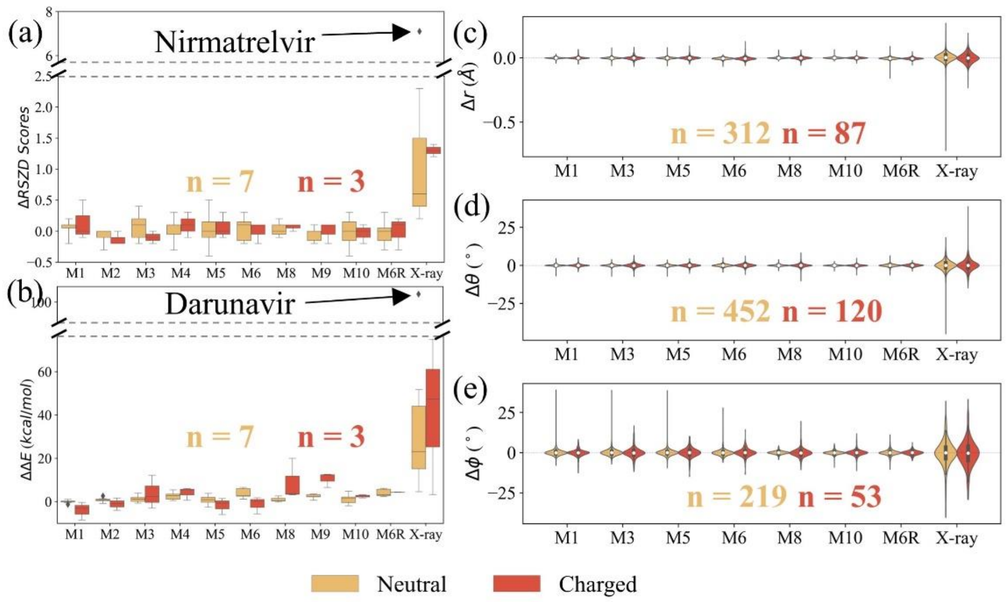

Supplementary Figure 14: Comparison of quantum refinement results (10 systems). Box plots of deviation of (**a**) real-space Z-difference (RSZD) scores as well as (**b**) strain energy (ΔΔE, kcal·mol^{-1}), boxplots indicate median values, interquartile range, minimum and maximum value, and individual data points. and violin plots of the deviations (**c**) bond distances (Δr), (**d**) angles (Δθ) and (**e**) rotatable dihedrals (Δϕ) of drugs/inhibitors in the selected 10 (shown in Fig. 1b) protein–drug/inhibitor systems after **M1-M10** quantum refinement approaches compared to **M7** ONIOM3(DFT:SE:MM)). The X-ray results were taken from the experimental structures without further refinement. The white dots in violin plots indicate the median values and inner boxplots indicate interquartile range. The number of data was marked in each plot.

Supplementary Table 16: Real-space Z-difference (RSZD) scores of quantum refinements. RSZD scores of the selected 10 drugs/inhibitors (shown in Fig. 1) in the proteins from our quantum refinement calculations (**M1-M10**). Those results for X-ray were taken from the experimental structure without our further refinement.

Drugs	Imatinib	Ibrutinib	Oseltamivir	Osimertinib	Nirmatrelvir	Rivaroxaban	Darunavir	CPI-0610	Mometasone	Ciprofloxacin	Average
**M1**	3.9	1.8	1.3	0.4	0.9	0.3	4.4	0.8	5.5	1.9	2.1
**M2**	3.6	1.7	1.2	0.4	0.5	0.4	4.3	0.6	5.4	1.2	1.9
**M3**	3.7	1.8	1.3	0.4	0.9	0.3	4.3	0.8	5.7	1.2	2.0
**M4**	3.6	1.8	1.3	0.5	-	-	-	-	-	-	2.1
**M4a**	-	-	-	-	0.8	0.6	4.3	0.8	5.3	1.7	
**M5**	3.5	1.7	1.3	0.4	-	-	-	-	-	-	2.1
**M5a**	-	-	-	-	1.0	0.4	4.2	0.7	5.7	1.7	
**M6**	3.7	1.8	1.5	0.5	0.8	0.3	4.5	0.6	5.5	1.2	2.0
**M7**	3.9	1.7	1.4	0.4	0.4	0.5	4.3	0.7	5.4	1.4	2.0
**M8**	3.8	1.8	1.4	0.5	0.6	0.4	4.3	0.7	5.6	1.5	2.1
**M9**	3.7	1.7	1.5	0.5	-	-	-	-	-	-	2.0
**M9a**	-	-	-	-	1.2	0.3	4.3	0.7	5.3	1.2	
**M10**	3.5	1.8	1.5	0.4	-	-	-	-	-	-	2.0
**M10a**	-	-	-	-	0.7	0.3	4.2	0.7	5.7	1.2	
**M6R**	3.8	1.8	1.6	0.5	0.5	0.3	4.6	0.7	5.1	1.1	2.0
**X-ray**	4.1	1.9	2.8	1.6	7.6	1.1	5.0	1.3	7.7	2.7	3.6

Supplementary Table 17: Strain energy of quantum refinements. Strain energy (ΔE, kcal·mol^{-1}) of the selected 10 drugs/inhibitors (shown in Fig. 1) in the proteins from our quantum refinement calculations (**M1-M10**). Those results for X-ray were taken from the experimental structure without our further refinement.

Drugs	Imatinib	Ibrutinib	Oseltamivir	Osimertinib	Nirmatrelvir	Rivaroxaban	Darunavir	CPI-0610	Mometasone	Ciprofloxacin	Average
M1	18.29	5.32	19.75	60.71	29.24	13.42	15.67	8.46	7.07	46.22	22.41
M2	19.24	5.84	24.23	60.19	30.39	14.85	16.71	8.87	7.32	51.31	23.90
M3	17.28	5.29	34.66	63.38	32.40	14.93	16.11	9.76	8.83	52.29	25.49
M4	19.21	5.98	28.53	66.58							26.42
M4a					33.62	15.22	17.44	11.13	10.62	55.91	
M5	15.22	4.03	21.72	62.51							23.5
M5a					32.57	15.33	16.07	9.32	8.88	49.36	
M6	18.79	7.60	22.69	62.56	34.90	17.22	20.41	10.79	12.60	49.66	25.72
M7	17.88	4.99	22.61	60.99	29.49	15.05	14.08	8.41	6.95	55.31	23.58
M8	17.66	5.42	42.44	64.24	32.05	15.07	15.92	9.13	8.38	58.46	26.88
M9	21.27	5.78	35.10	67.72							28.42
M9a					32.69	16.99	16.39	10.58	10.09	67.61	
M10	15.83	4.01	24.20	63.54							24.97
M10a					33.36	15.02	16.31	10.47	8.41	58.51	
M6R	20.87	12.92	26.94	61.48	34.61	17.70	19.97	10.77	12.29	59.55	27.71
X-ray	40.40	12.90	96.31	108.58	60.94	37.35	117.26	12.83	57.51	58.53	60.26

Supplementary Table 18: Selected coordinates. Selected key bonds distances, angles and dihedrals marked in Figure 1b from our **M1-M10** quantum refinement calculations. Those results for X-ray were taken from the experimental structure without our further refinement.

	M1	M2	M3	M4	M5	M6	M7	M8	M9	M10	M6R	X-ray
**Bond Length (Å)**												
b1	1.33	1.33	1.33	1.32	1.34	1.32	1.34	1.34	1.33	1.34	1.33	**1.53**
b2	1.34	1.33	1.33	1.34	1.34	1.33	1.34	1.34	1.34	1.35	1.33	**1.53**
b3	1.54	1.54	1.55	1.54	1.54	1.55	1.54	1.55	1.54	1.54	1.55	**1.26**
b4	1.53	1.53	1.53	1.52	1.53	1.53	1.53	1.53	1.52	1.53	1.53	**1.28**
b5	1.87	-	1.85	-	1.86	1.89	1.86	1.85	-	1.85	1.89	**1.67**
**Bond Angle (°)**												
a1	126.3	126.8	125.2	125.1	125.3	123.8	127.7	126.1	126.0	126.8	125.9	**110.2**
**Dihedral (°)**												
d1	41.9	42.6	44.5	54.1	45.3	42.9	44.3	47.3	49.6	48.6	47.8	**4.2**
d2	47.9	47.2	50.7	50.8	48.5	49.7	47.2	50.2	49.9	48.1	48.9	**12.1**

Supplementary Table 19: Comparison of quantum refinement results (10 systems). Correlation (R^2) and median absolute deviation (MAD) of the refined bond distances (n = 399), angles (n = 572), rotatable dihedrals (n = 272) for all drugs/inhibitors in the selected 10 protein-drug/inhibitor systems (shown in Fig. 1) from **M1-M10** QRs with respect to **M7** as the reference. Those results for X-ray were taken from the experimental structure without our further refinement.

		M1	M2	M3	M4	M5	M6	M8	M9	M10	M6R	X-ray
**R^2**	Bond	0.99	1.00	0.99	0.99	0.99	0.99	0.99	0.99	1.00	0.99	0.85
	Angle	0.99	0.99	0.98	0.98	0.98	0.98	0.98	0.99	0.99	0.98	0.71
	All Dihedral	0.92	0.90	0.92	0.92	0.92	0.92	1.00	0.99	1.00	1.00	0.51
	Rotatable Dihedral	0.90	0.90	0.86	0.85	0.95	0.85	0.86	0.95	0.95	0.90	0.67
**MAD**	Bond (Å)	0.00	0.00	0.01	0.01	0.01	0.01	0.00	0.01	0.00	0.01	0.03
	Angle (°)	0.5	0.4	0.7	0.7	0.6	0.7	0.5	0.6	0.4	0.6	2.4
	All Dihedral (°)	4.0	4.3	4.4	4.6	4.3	4.2	1.1	1.8	1.0	1.2	20.8
	Rotatable Dihedral (°)	4.5	4.3	6.6	6.8	3.1	6.4	6.1	3.1	2.7	4.7	17.2

Supplementary Table 20: Comparison of quantum refinement results (7 neutral cases). Correlation (R^2) and median absolute deviation (MAD) of the refined bond distances (n = 312), angles (n = 452), rotatable dihedrals (n = 219) for the neutral drug/inhibitor cases in the selected 10 protein-drug/inhibitor systems (shown in Fig. 1) from **M1-M10** QRs with respect to **M7** as the reference. Those results for X-ray were taken from the experimental structure without our further refinement.

		M1	M2	M3	M4	M5	M6	M8	M9	M10	M6R	X-ray
**R^2**	Bond	1.00	1.00	0.99	0.99	0.99	0.99	1.00	1.00	1.00	0.99	0.88
	Angle	0.99	0.99	0.99	0.99	0.99	0.98	0.99	0.99	0.99	0.98	0.73
	All Dihedral	0.93	0.91	0.93	0.93	0.93	0.93	1.00	0.98	1.00	1.00	0.55
	Rotatable Dihedral	0.87	0.93	0.93	0.93	0.93	0.87	0.93	1.00	0.93	0.93	0.68
**MAD**	Bond (Å)	0.00	0.00	0.00	0.01	0.00	0.01	0.00	0.00	0.00	0.01	0.02
	Angle (°)	0.4	0.4	0.6	0.6	0.6	0.7	0.4	0.5	0.4	0.7	2.2
	All Dihedral (°)	3.4	3.8	3.7	3.9	3.7	3.7	0.9	1.8	1.0	1.2	18.7
	Rotatable Dihedral (°)	5.4	3.1	3.5	3.7	3.6	5.7	3.1	1.2	3.3	3.6	16.1

Supplementary Table 21: Comparison of quantum refinement results (3 charged cases). Correlation (R^2) and median absolute deviation (MAD) of the refined bond distances (n = 87), angles (n = 120), rotatable dihedrals (n = 53) for charged drug/inhibitor cases in the selected 10 protein-drug/inhibitor systems (shown in Fig. 1) from **M1-M10** QRs with respect to **M7** as the reference. Those results for X-ray were taken from the experimental structure without our further refinement.

		**M1**	**M2**	**M3**	**M4**	**M5**	**M6**	**M8**	**M9**	**M10**	**M6R**	**X-ray**
**R^2**	Bond	0.98	0.98	0.98	0.98	0.96	0.96	0.98	0.98	0.99	0.97	0.63
	Angle	0.98	0.99	0.93	0.94	0.98	0.98	0.93	0.95	0.99	0.98	0.57
	All Dihedral	0.88	0.88	0.88	0.88	0.88	0.88	1.00	1.00	1.00	1.00	0.41
	Rotatable Dihedral	1.00	0.81	0.63	0.62	1.00	0.81	0.63	0.82	1.00	0.82	0.63
**MAD**	Bond (Å)	0.01	0.01	0.01	0.01	0.01	0.01	0.01	0.01	0.00	0.01	0.03
	Angle (°)	0.6	0.5	1.1	1.1	0.7	0.7	0.9	1.0	0.5	0.6	2.9
	All Dihedral (°)	6.3	5.8	6.9	7.2	6.3	6.2	2.0	1.9	0.8	1.2	28.7
	Rotatable Dihedral (°)	1.7	8.1	16.0	16.4	1.6	8.6	15.7	9.1	1.0	8.3	20.5

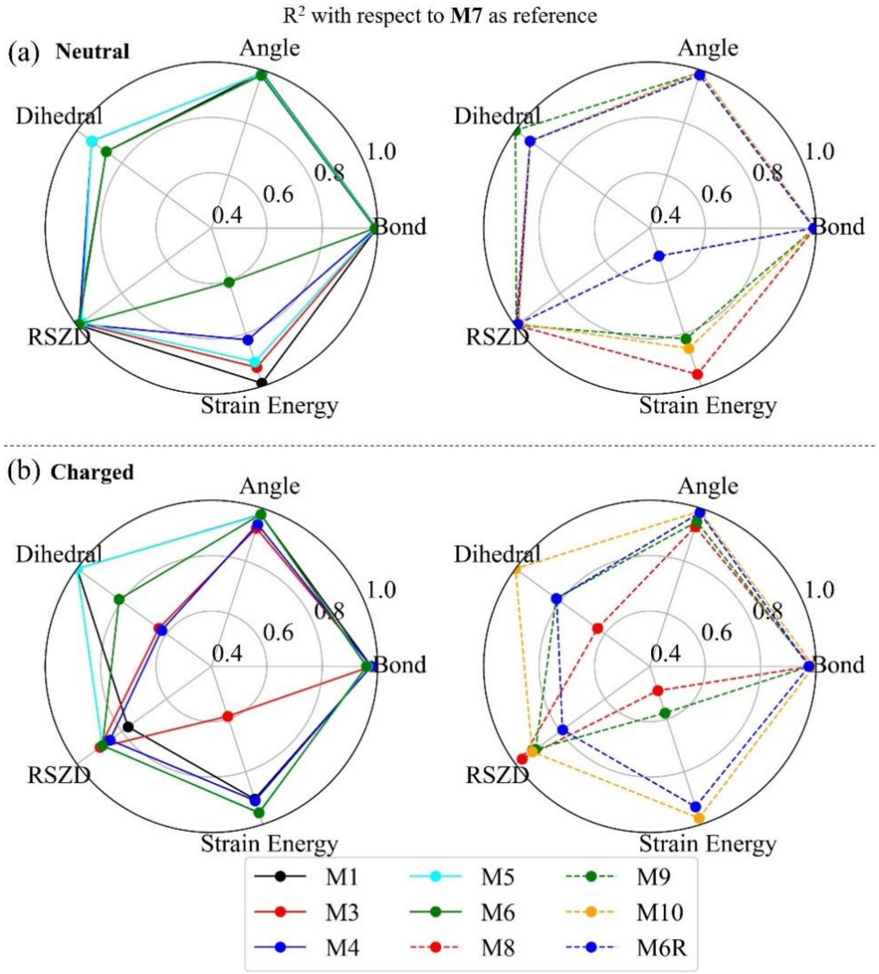

Supplementary Figure 15: Correlation of different methods. Correlation (R^2) of the refined bond distances, angles, rotatable dihedrals, real-space Z-difference (RSZD) score and strain energy for our (**a**) neutral (number of data: 312, 452, 219, 7, 7) and (**b**) charged (number of data: 87， 120， 53， 3， 3) drug/inhibitor cases in the selected 10 protein-drug/inhibitor systems (shown in Fig. 1) from **M1-M10** QRs with respect to **M7** as the reference.

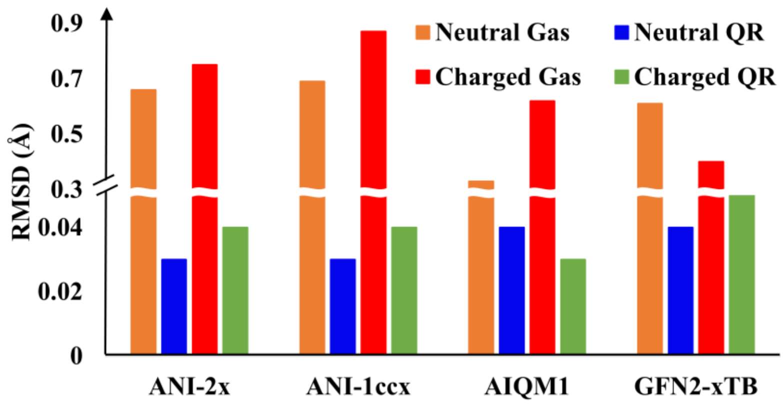

Supplementary Figure 16: Structural comparison in the gas phase and in proteins (10 systems). Root mean square deviation (RMSD) of the selected 10 structures (shown in Fig. 1b) optimized using the ANI-2x, ANI-1ccx, AIQM1, and GFN2-xTB methods compared to the ωB97X/6-31G(d) method for the neutral drug/inhibitor cases in the gas phase (orange) and in the proteins after quantum refinement (blue) and for the charged drug/inhibitor cases in the gas phase (red) and in the proteins after quantum refinements (green). The QR data by the **M7**, **M8**, **M9**, **M10** and **M6R** schemes (ONIOM3(DFT:SE:MM), ONIOM3(ANI-2x:SE:MM), ONIOM3(ANI-1ccx:SE:MM), ONIOM3(AIQM1:SE:MM) and ONIOM2(SE:MM), respectively) were used.

Computations of the selected 50 protein-drug/inhibitor systems (Supplementary Figs. 1-4)

After tests on selected 10 protein-drug/inhibitor systems (shown in Fig. 1), the CC-level AIQM1 based methods (**M5**, **M5a**, **M10** and **M10a**) yields better results than the other CC-level ANI-1ccx based methods (**M4**, **M4a**, **M9** and **M9a**). Therefore, we chose AIQM1 as the only one CC-level method for the following quantum refinements. ONIOM2(AIQM1:ANI-2x) based method (**M5a** and **M10a**) were used to describe the drug/inhibitor molecules (C, H, O, N, F, Cl, S) due to the element limitation (C, H, O, N) in AIQM1. For the three drug/inhibitor molecules (C, H, O, N, F, Cl, S, Br, P) in 2QBR, 1NOX and 5Y62, ONIOM2(AIQM1:GFN2-xTB) were used due to the element limitation (C, H, O, N) in AIQM1.

Supplementary Table 22: Comparison of quantum refinement results (QR50). Correlation (R^2) and median absolute deviation (MAD) of the refined bond distances (n = 1605), angles (n = 2285), rotatable dihedrals (n = 894) for all drugs/inhibitors in the selected 50 protein-drug/inhibitor systems from **M1-M10** QRs with respect to **M7** as the reference. Those results for X-ray were taken from the experimental structure without our further refinement.

		**M1**	**M3**	**M5**	**M6**	**M8**	**M10**	**M6R**	**X-ray**
	Bond	0.995	0.990	0.991	0.986	0.993	0.996	0.991	0.843
**R^2**	Angle	0.990	0.978	0.985	0.980	0.981	0.992	0.985	0.717
	Rotatable Dihedral	0.999	0.999	0.999	0.999	1.000	1.000	1.000	0.992
	Bond (Å)	0.005	0.007	0.007	0.009	0.005	0.004	0.007	0.027
**MAD**	Angle (°)	0.4	0.7	0.6	0.7	0.6	0.4	0.6	2.5
	Rotatable Dihedral (°)	1.2	1.7	1.6	1.5	1.2	1.0	1.1	6.6

Supplementary Table 23: Comparison of quantum refinement results (neutral cases in QR50). Correlation (R^2) and median absolute deviation (MAD) of the refined bond distances (n = 901), angles (n = 1292), rotatable dihedrals (n = 486) for the neutral drug/inhibitor cases in the selected 50 protein-drug/inhibitor systems from **M1-M10** QRs with respect to **M7** as the reference. Those results for X-ray were taken from the experimental structure without our further refinement.

		**M1**	**M3**	**M5**	**M6**	**M8**	**M10**	**M6R**	**X-ray**
	Bond	0.997	0.995	0.994	0.992	0.998	0.997	0.991	0.841
**R^2**	Angle	0.992	0.989	0.989	0.985	0.995	0.995	0.982	0.755
	Rotatable Dihedral	0.999	0.999	0.999	1.000	1.000	1.000	1.000	0.990
	Bond (Å)	0.004	0.005	0.005	0.007	0.003	0.004	0.007	0.024
**MAD**	Angle (°)	0.4	0.5	0.5	0.6	0.4	0.3	0.6	2.3
	Rotatable Dihedral (°)	1.1	1.3	1.3	1.3	0.8	0.9	1.2	7.0

Supplementary Table 24: Comparison of quantum refinement results (charged cases in QR50). Correlation (R^2) and median absolute deviation (MAD) of the refined bond distances (n = 704), angles (n = 993), rotatable dihedrals (n = 408) for the charged drug/inhibitor cases in the selected 50 protein-drug/inhibitor systems from **M1-M10** QRs with respect to **M7** as the reference. Those results for X-ray were taken from the experimental structure without our further refinement.

		**M1**	**M3**	**M5**	**M6**	**M8**	**M10**	**M6R**	**X-ray**
	Bond	0.993	0.983	0.986	0.978	0.988	0.995	0.991	0.846
$\mathbf{R^2}$	Angle	0.987	0.964	0.979	0.973	0.962	0.987	0.988	0.661
	Rotatable Dihedral	1.000	0.999	0.999	0.999	0.999	1.000	1.000	0.994
	Bond (Å)	0.006	0.009	0.008	0.011	0.008	0.005	0.008	0.030
**MAD**	Angle (°)	0.5	0.9	0.7	0.8	0.8	0.5	0.6	2.7
	Rotatable Dihedral (°)	1.3	2.1	2.0	1.8	1.6	1.2	0.9	6.2

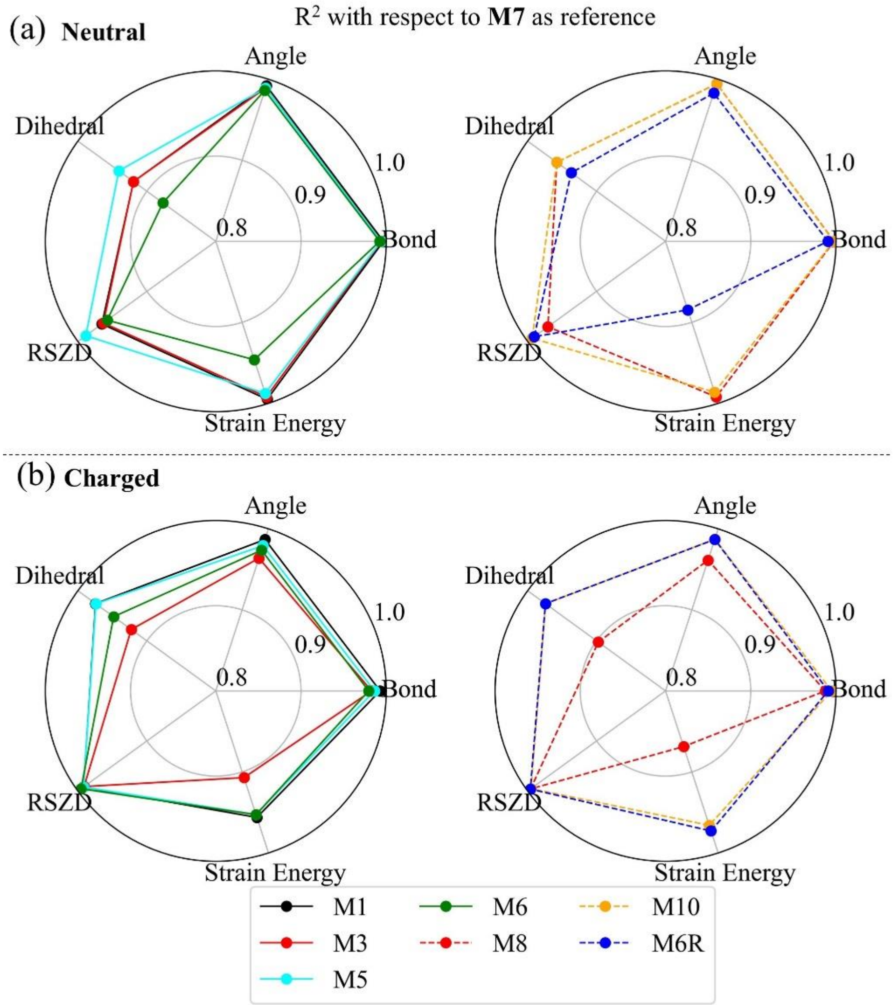

Supplementary Figure 17: Correlation of different methods (QR50). Correlation (R^2) of the refined bond distances, angles, rotatable dihedrals, real-space Z-difference (RSZD) score and strain energy for our (**a**) neutral (number of data: 901, 1292, 486, 24, 24) and (**b**) charged (number of data: 704, 993, 408, 26, 26) drug/inhibitor cases in the all 50 protein-drug/inhibitor systems from **M1-M10** QRs with respect to **M7** as the reference.

Supplementary Table 25: Real-space Z-difference (RSZD) of quantum refinments. RSZD scores of the all 50 drugs/inhibitors in the proteins (QR50) from our quantum refinement calculations (**M1-M10**). Those results for X-ray were taken from the experimental structure without our further refinement.

PDB ID	M1	M3	M5	M6	M7	M8	M10	M6R	X-ray
**1xbb**	3.9	3.7	3.5	3.7	3.9	3.8	3.5	3.8	4.1
**2w26**	0.3	0.3	0.4	0.3	0.5	0.4	0.3	0.3	1.1
**4hzz**	1.3	1.3	1.3	1.5	1.4	1.4	1.5	1.6	2.8
**4kra**	1.9	1.2	1.7	1.2	1.4	1.5	1.2	1.1	2.7
**4p6w**	5.6	5.6	5.5	5.4	5.6	5.5	5.5	5.2	7.7
**5hls**	0.8	0.9	0.8	0.7	0.7	0.6	0.6	0.8	1.3
**5kr1**	4.4	4.3	4.2	4.5	4.3	4.3	4.2	4.6	5.0
**5p9i**	1.8	1.8	1.7	1.8	1.7	1.8	1.8	1.8	1.9
**6jx4**	0.4	0.4	0.4	0.5	0.4	0.5	0.4	0.5	1.6
**7rfw**[a]	0.7	0.9	1.0	0.8	0.5	0.6	0.7	0.5	7.6
**3qqa**	2.8	2.8	2.8	2.8	2.8	2.8	2.8	2.8	2.7
**4xh6**	4.3	4.3	4.3	4.2	4.3	4.2	4.2	4.1	4.3
**4yhm**	3.1	3.1	3.0	3.1	3.1	3.1	3.1	3.1	3.8
**6v83**[a]	2.0	1.8	1.7	1.9	2.1	2.3	2.3	1.9	2.0
**1ajx**	1.1	1.1	1.1	1.0	1.0	1.1	1.0	0.9	1.9
**1br5**	0.7	0.7	0.7	0.9	0.9	0.8	0.7	0.7	0.6
**1d4i**	0.2	0.1	0.2	0.1	0.2	0.1	0.1	0.3	2.2
**1f9g**	0.3	0.4	0.3	0.3	0.3	0.4	0.3	0.3	0.4
**1lf3**	1.9	1.9	1.9	1.9	1.9	1.9	1.9	2.0	2.3
**4zb8**	1.9	1.9	1.9	1.9	1.6	1.7	1.6	1.7	3.7
**1p1o**	2.3	2.3	2.3	2.5	2.4	2.5	2.5	2.6	2.4
**1pf7**	0.4	0.4	0.8	0.4	0.3	0.3	0.3	0.4	1.3
**1m7q**	8.4	8.5	8.5	8.5	8.4	8.5	8.5	8.5	9.6
**1pa9**	0.3	0.2	0.3	0.6	0.4	0.3	0.2	0.3	2.3
**1xoz**	0.0	0.0	0.0	0.0	0.0	0.0	0.0	0.0	3.6
**2ax6**	1.6	1.6	1.6	1.6	1.6	1.6	1.6	1.6	1.8
**2iwu**	2.5	2.5	2.5	2.6	2.5	2.5	2.5	2.6	2.5
**2p7z**	0.9	0.9	0.8	0.9	0.5	0.8	0.5	0.5	1.8
**1uy9**	0.1	0.1	0.1	0.1	0.1	0.1	0.1	0.1	0.3
**3eq7**	2.1	2.2	2.1	2.0	2.1	2.1	2.1	2.0	2.4
**5y1y**	1.1	1	1.1	1.3	1.3	1.2	1.1	1.2	1.2
**5y62**	1.2	1.2	1.3	1.2	1.3	1.3	1.3	1.3	1.4
**1nox**	1.7	1.5	1.5	1.6	1.6	1.3	1.5	1.6	2.5
**1nj1**	2.5	2.5	2.3	2.1	2.0	2.2	2.3	1.7	3.9
**2a3i**	1.1	1.1	1.1	1.1	1.2	1.1	1.1	1.1	1.6
**5ct1**	2.1	2.1	2.2	2.1	2.1	2.1	2.1	2.1	2.1
**6afa**[a]	2.2	2.1	0.9	2.0	0.8	2.1	1.0	1.1	6.0
**6pub**	0.9	1.3	1.2	1.1	0.9	0.9	0.8	0.9	2.2
**1owk**	1.4	1.4	1.4	1.4	1.5	1.4	1.4	1.4	1.7
**2qbr**	2.4	2.4	2.4	2.4	2.6	2.5	2.6	2.7	3.9
**1yat**	2.6	2.6	2.5	2.6	2.6	2.5	2.5	2.6	3.1
**1xp9**	0.4	0.4	0.4	0.4	0.4	0.4	0.4	0.4	1.2
**2hxm**	9.4	9.5	9.2	9.4	9.3	9.4	9.1	9.2	10.4
**2wca**	1.1	1.1	1.1	1.2	0.6	0.6	0.6	0.6	2.5
**2wi4**	1.5	1.6	1.7	1.8	1.5	1.5	1.6	1.7	3.5
**1uyg**	0.0	0.0	0.0	0.0	0.0	0.0	0.0	0.0	0.6
**2wi1**	2.1	2.1	2.1	2.1	2.1	2.0	2.0	2.1	4.4
**3fr2**	0.7	0.5	0.7	0.7	0.8	0.5	0.4	0.5	0.8
**3atm**	1.8	1.8	1.8	1.9	1.8	1.8	1.8	1.8	2.1

**5l7i**	1.2	1.2	1.3	1.5	1.2	1.2	1.2	1.5	2.8
**Average**	1.9	1.9	1.9	1.9	1.9	1.9	1.8	1.8	2.9

a. The RSZD scores contributed from the drug/inhibitor and its covalent linkage residue.

Supplementary Table 26: Strain energy of quantum refinements. Strain energy (ΔE, kcal·mol^{-1}) at ωB97X-D/6-31G(d) level of the all 50 drugs/inhibitors in the proteins (QR50) from our quantum refinement calculations (**M1-M10**). Those results for X-ray were taken from the experimental structure without our further refinement.

PDB ID	M1	M3	M5	M6	M7	M8	M10	M6R	X-ray
**1xbb**	18.29	17.28	15.22	18.79	17.88	17.66	15.83	20.87	40.40
**4hzz**	19.75	34.66	21.72	22.69	22.61	42.44	24.20	26.94	96.31
**5p9i**	5.32	5.29	4.03	7.60	4.99	5.42	4.01	12.92	12.90
**6jx4**	60.71	63.38	62.51	62.56	60.99	64.24	63.54	61.48	108.58
**2w26**	13.42	14.93	15.33	17.22	15.05	15.07	15.02	17.70	37.35
**4kra**	46.22	52.29	49.36	49.66	55.31	58.46	58.51	59.55	58.53
**4p6w**	7.07	8.83	8.88	12.60	6.95	8.38	8.41	12.29	57.51
**5hls**	8.46	9.76	9.32	10.79	8.41	9.13	10.47	10.77	12.83
**5kr1**	15.67	16.11	16.07	20.41	14.08	15.92	16.31	19.97	117.26
**7rfw**[a]	29.24	32.40	32.57	34.90	29.49	32.05	33.36	34.61	60.94
**1ajx**	17.94	18.76	18.55	19.98	17.97	20.42	19.08	21.10	46.10
**1br5**	19.00	19.72	19.51	19.98	18.81	19.61	19.86	22.60	45.38
**1d4i**	29.19	30.42	30.96	33.41	31.45	32.00	32.67	34.52	41.89
**1f9g**	10.94	15.68	12.40	12.34	9.53	15.26	11.56	11.40	37.76
**1lf3**	57.16	57.63	59.73	61.74	57.27	57.75	60.36	62.08	121.07
**1m7q**	15.41	18.17	16.69	18.97	16.05	19.36	17.81	19.60	99.64
**1nj1**	29.19	34.15	37.02	38.79	42.22	35.16	35.26	47.50	40.91
**1nox**	14.17	16.20	16.00	16.18	14.97	15.37	15.41	16.19	79.05
**1owk**	22.12	32.99	23.27	27.27	17.42	25.53	18.93	21.54	22.11
**1p1o**	26.27	28.95	26.95	28.07	35.75	47.42	37.09	37.84	34.55
**1pa9**	5.53	9.39	6.51	7.71	7.11	12.00	9.74	8.60	44.48
**1pf7**	34.85	37.80	35.95	36.43	34.50	37.56	35.45	36.26	55.30
**1uy9**	2.38	2.87	3.10	4.55	2.43	2.59	2.53	4.87	18.96
**1uyg**	8.27	8.59	8.48	9.26	8.99	9.79	9.37	10.55	46.12
**1xoz**	3.28	3.93	3.87	4.10	3.40	3.93	4.02	4.46	21.23
**1xp9**	44.01	48.54	48.26	48.54	45.52	52.61	49.88	50.41	88.32
**1yat**	31.33	32.63	32.32	34.08	31.44	32.80	32.72	34.94	65.54
**2a3i**	12.52	12.45	12.81	14.31	11.06	11.68	12.25	15.85	42.33
**2ax6**	3.03	3.38	3.14	4.85	3.93	3.62	3.88	6.10	56.67
**2hxm**	5.45	12.82	7.08	9.76	8.02	13.29	9.51	13.14	99.07
**2iwu**	21.42	23.97	22.38	22.16	21.21	23.79	22.48	22.36	54.00
**2p7z**	15.48	18.53	17.98	17.97	12.29	18.01	14.92	15.82	33.00
**2qbr**	15.37	18.91	18.73	19.16	16.26	28.81	20.27	18.23	40.73
**2wca**	16.08	16.99	16.55	18.02	16.25	16.85	16.56	18.08	17.34
**2wi1**	13.01	14.06	13.75	14.63	13.30	13.25	13.45	14.56	34.29
**2wi4**	8.22	8.60	9.43	11.85	7.52	7.07	8.91	11.41	18.49

**3atm**	1.69	3.48	2.28	2.83	2.49	5.04	2.78	4.29	5.71
**3eq7**	16.63	16.81	16.77	18.74	16.77	16.79	16.65	18.07	37.57
**3fr2**	10.86	13.46	11.35	12.70	10.85	14.29	11.68	13.01	38.13
**3qqa**	79.14	85.57	78.66	84.73	80.66	83.98	83.48	84.00	239.82
**4xh6**	15.51	18.15	18.09	17.84	16.20	17.36	17.82	17.97	25.63
**4yhm**	41.56	48.48	43.31	48.80	39.96	48.41	41.71	43.10	61.92
**4zb8**	66.89	75.81	71.30	73.65	73.62	82.89	77.53	77.67	89.45
**5ct1**	37.27	41.56	42.13	38.84	38.06	43.72	43.00	39.56	59.43
**5y1y**	5.50	7.40	5.92	6.42	6.61	8.05	7.58	7.59	31.86
**5y62**	37.06	38.47	38.47	42.24	34.54	36.38	36.78	37.48	90.46
**6afa**[b]	26.37	27.12	27.00	31.63	28.99	29.66	29.63	44.16	48.45
**6pub**	6.66	12.40	10.53	8.34	6.80	9.61	8.60	7.33	28.95
**6v83**	52.56	65.47	64.35	60.22	48.53	65.99	63.45	57.82	66.62
**5l7i**[c]	79.21	79.59	81.24	89.54	79.04	80.36	80.46	89.39	21.39
**Average**	23.65	26.70	25.36	26.96	24.47	27.74	26.10	27.97	55.05

a. The strain energy contributed from the bonded (70 %) and nonbonded (30 %) forms. b. The strain energy contributed from the bonded (50 %) and nonbonded (50 %) forms. c. Total strain energy of drugs/inhibitors in chain A and chain B.

Supplementary Table 27: Root mean square deviation (RMSD) in bonds of quantum refinements. RMSD (Å) in bonds of the all 50 drugs/inhibitors in the proteins (QR50) from our quantum refinement calculations (**M1-M10**) with respect to **M7** as the reference. Those results for X-ray were taken from the experimental structure without our further refinement.

PDB ID	M1	M3	M5	M6	M8	M10	M6R	X-ray
**1xbb**	0.006	0.005	0.005	0.008	0.002	0.004	0.007	0.020
**2w26**	0.008	0.010	0.009	0.012	0.008	0.008	0.011	0.037
**4hzz**	0.014	0.009	0.025	0.019	0.012	0.007	0.007	0.059
**4kra**	0.017	0.017	0.016	0.020	0.008	0.007	0.015	0.025
**4p6w**	0.003	0.007	0.007	0.008	0.006	0.006	0.007	0.042
**5hls**	0.004	0.006	0.008	0.012	0.006	0.008	0.010	0.022
**5kr1**	0.004	0.006	0.005	0.010	0.004	0.005	0.006	0.075
**5p9i**	0.005	0.005	0.006	0.009	0.003	0.004	0.015	0.015
**6jx4**	0.006	0.007	0.008	0.012	0.008	0.006	0.007	0.052
**7rfw (bonded)**	0.007	0.008	0.008	0.010	0.008	0.009	0.011	0.030
**7rfw (non-bonded)**	0.007	0.008	0.010	0.009	0.004	0.004	0.007	0.030
**3qqa**	0.003	0.006	0.007	0.010	0.006	0.006	0.009	0.046
**4xh6**	0.007	0.008	0.006	0.009	0.006	0.005	0.005	0.023
**4yhm**	0.009	0.016	0.009	0.021	0.011	0.005	0.010	0.024
**6v83**	0.012	0.018	0.016	0.021	0.014	0.011	0.016	0.030
**1ajx**	0.005	0.005	0.008	0.008	0.004	0.004	0.007	0.025
**1br5**	0.011	0.013	0.011	0.012	0.006	0.006	0.009	0.046
**1d4i**	0.006	0.007	0.007	0.010	0.004	0.004	0.006	0.023
**1f9g**	0.004	0.023	0.010	0.010	0.023	0.010	0.009	0.039
**1lf3**	0.003	0.004	0.005	0.008	0.003	0.004	0.007	0.038
**4zb8**	0.012	0.021	0.017	0.025	0.014	0.007	0.012	0.044
**1p1o**	0.020	0.011	0.027	0.039	0.011	0.005	0.013	0.018
**1pf7**	0.007	0.013	0.011	0.012	0.011	0.008	0.007	0.055
**1m7q**	0.005	0.007	0.006	0.009	0.006	0.005	0.008	0.060
**1pa9**	0.021	0.027	0.019	0.025	0.022	0.012	0.012	0.058
**1xoz**	0.002	0.004	0.003	0.005	0.003	0.003	0.005	0.022
**2ax6**	0.010	0.011	0.010	0.011	0.006	0.006	0.008	0.066
**2iwu**	0.006	0.017	0.013	0.010	0.015	0.011	0.008	0.058
**2p7z**	0.007	0.008	0.008	0.009	0.009	0.004	0.010	0.033
**1uy9**	0.003	0.002	0.005	0.008	0.004	0.005	0.011	0.018
**3eq7**	0.006	0.007	0.007	0.010	0.003	0.003	0.006	0.037
**5y1y**	0.010	0.021	0.010	0.013	0.017	0.005	0.008	0.053
**5y62**	0.006	0.010	0.011	0.014	0.010	0.011	0.011	0.056
**1nox**	0.004	0.011	0.009	0.011	0.008	0.007	0.010	0.041
**1nj1**	0.011	0.018	0.019	0.017	0.017	0.016	0.012	0.029
**2a3i**	0.003	0.005	0.006	0.005	0.004	0.004	0.007	0.053
**5ct1**	0.009	0.013	0.011	0.022	0.014	0.007	0.013	0.054
**6afa (bonded)**	0.018	0.023	0.023	0.014	0.007	0.007	0.025	0.043

**6afa (non-bonded)**	0.011	0.022	0.023	0.020	0.015	0.016	0.038	0.180
**6pub**	0.003	0.012	0.007	0.008	0.012	0.005	0.008	0.034
**1owk**	0.010	0.021	0.009	0.008	0.014	0.004	0.008	0.030
**2qbr**	0.011	0.018	0.019	0.020	0.017	0.008	0.008	0.038
**1yat**	0.003	0.006	0.006	0.006	0.004	0.004	0.006	0.025
**1xp9**	0.006	0.011	0.011	0.010	0.012	0.008	0.009	0.045
**2hxm**	0.007	0.010	0.009	0.017	0.009	0.005	0.012	0.042
**2wca**	0.008	0.010	0.010	0.011	0.004	0.004	0.009	0.025
**2wi4**	0.006	0.007	0.009	0.014	0.005	0.006	0.015	0.031
**1uyg**	0.006	0.005	0.005	0.008	0.005	0.004	0.008	0.066
**2wi1**	0.005	0.004	0.005	0.006	0.003	0.004	0.005	0.013
**3fr2**	0.004	0.005	0.005	0.008	0.006	0.006	0.007	0.040
**3atm**	0.008	0.005	0.013	0.008	0.007	0.004	0.009	0.026
**5l7i**	0.006	0.006	0.006	0.013	0.004	0.005	0.011	0.022

Supplementary Table 28: Median absolute deviation (MAD) in bonds of quantum refinements. MAD (Å) in bonds of the all 50 drugs/inhibitors in the proteins (QR50) from our quantum refinement calculations (**M1-M10**) with respect to **M7** as the reference. Those results for X-ray were taken from the experimental structure without our further refinement.

PDB ID	M1	M3	M5	M6	M8	M10	M6R	X-ray
**1xbb**	0.006	0.005	0.005	0.008	0.002	0.004	0.007	0.020
**2w26**	0.008	0.010	0.009	0.012	0.008	0.008	0.011	0.037
**4hzz**	0.014	0.009	0.025	0.019	0.012	0.007	0.007	0.059
**4kra**	0.017	0.017	0.016	0.020	0.008	0.007	0.015	0.025
**4p6w**	0.003	0.007	0.007	0.008	0.006	0.006	0.007	0.042
**5hls**	0.004	0.006	0.008	0.012	0.006	0.008	0.010	0.022
**5kr1**	0.004	0.006	0.005	0.010	0.004	0.005	0.006	0.075
**5p9i**	0.005	0.005	0.006	0.009	0.003	0.004	0.015	0.015
**6jx4**	0.006	0.007	0.008	0.012	0.008	0.006	0.007	0.052
**7rfw (bonded)**	0.007	0.008	0.008	0.010	0.008	0.009	0.011	0.030
**7rfw (non-bonded)**	0.007	0.008	0.010	0.009	0.004	0.004	0.007	0.030
**3qqa**	0.003	0.006	0.007	0.010	0.006	0.006	0.009	0.046
**4xh6**	0.007	0.008	0.006	0.009	0.006	0.005	0.005	0.023
**4yhm**	0.009	0.016	0.009	0.021	0.011	0.005	0.010	0.024
**6v83**	0.012	0.018	0.016	0.021	0.014	0.011	0.016	0.030
**1ajx**	0.005	0.005	0.008	0.008	0.004	0.004	0.007	0.025
**1br5**	0.011	0.013	0.011	0.012	0.006	0.006	0.009	0.046
**1d4i**	0.006	0.007	0.007	0.010	0.004	0.004	0.006	0.023
**1f9g**	0.004	0.023	0.010	0.010	0.023	0.010	0.009	0.039
**1lf3**	0.003	0.004	0.005	0.008	0.003	0.004	0.007	0.038
**4zb8**	0.012	0.021	0.017	0.025	0.014	0.007	0.012	0.044
**1p1o**	0.020	0.011	0.027	0.039	0.011	0.005	0.013	0.018
**1pf7**	0.007	0.013	0.011	0.012	0.011	0.008	0.007	0.055
**1m7q**	0.005	0.007	0.006	0.009	0.006	0.005	0.008	0.060
**1pa9**	0.021	0.027	0.019	0.025	0.022	0.012	0.012	0.058
**1xoz**	0.002	0.004	0.003	0.005	0.003	0.003	0.005	0.022
**2ax6**	0.010	0.011	0.010	0.011	0.006	0.006	0.008	0.066
**2iwu**	0.006	0.017	0.013	0.010	0.015	0.011	0.008	0.058
**2p7z**	0.007	0.008	0.008	0.009	0.009	0.004	0.010	0.033
**1uy9**	0.003	0.002	0.005	0.008	0.004	0.005	0.011	0.018
**3eq7**	0.006	0.007	0.007	0.010	0.003	0.003	0.006	0.037
**5y1y**	0.010	0.021	0.010	0.013	0.017	0.005	0.008	0.053
**5y62**	0.006	0.010	0.011	0.014	0.010	0.011	0.011	0.056
**1nox**	0.004	0.011	0.009	0.011	0.008	0.007	0.010	0.041
**1nj1**	0.011	0.018	0.019	0.017	0.017	0.016	0.012	0.029
**2a3i**	0.003	0.005	0.006	0.005	0.004	0.004	0.007	0.053
**5ct1**	0.009	0.013	0.011	0.022	0.014	0.007	0.013	0.054
**6afa (bonded)**	0.018	0.023	0.023	0.014	0.007	0.007	0.025	0.043

**6afa (non-bonded)**	0.011	0.022	0.023	0.020	0.015	0.016	0.038	0.180
**6pub**	0.003	0.012	0.007	0.008	0.012	0.005	0.008	0.034
**1owk**	0.010	0.021	0.009	0.008	0.014	0.004	0.008	0.030
**2qbr**	0.011	0.018	0.019	0.020	0.017	0.008	0.008	0.038
**1yat**	0.003	0.006	0.006	0.006	0.004	0.004	0.006	0.025
**1xp9**	0.006	0.011	0.011	0.010	0.012	0.008	0.009	0.045
**2hxm**	0.007	0.010	0.009	0.017	0.009	0.005	0.012	0.042
**2wca**	0.008	0.010	0.010	0.011	0.004	0.004	0.009	0.025
**2wi4**	0.006	0.007	0.009	0.014	0.005	0.006	0.015	0.031
**1uyg**	0.006	0.005	0.005	0.008	0.005	0.004	0.008	0.066
**2wi1**	0.005	0.004	0.005	0.006	0.003	0.004	0.005	0.013
**3fr2**	0.004	0.005	0.005	0.008	0.006	0.006	0.007	0.040
**3atm**	0.008	0.005	0.013	0.008	0.007	0.004	0.009	0.026
**5l7i**	0.006	0.006	0.006	0.013	0.004	0.005	0.011	0.022

Supplementary Table 29: R^2 in bonds of quantum refinements. R^2 in bonds of the all 50 drugs/inhibitors in the proteins (QR50) from our quantum refinement calculations (**M1-M10**) with respect to **M7** as the reference. Those results for X-ray were taken from the experimental structure without our further refinement.

PDB ID	M1	M3	M5	M6	M8	M10	M6R	X-ray
**1xbb**	0.991	0.993	0.993	0.982	0.999	0.996	0.988	0.894
**2w26**	0.996	0.994	0.995	0.991	0.996	0.996	0.993	0.911
**4hzz**	0.982	0.991	0.936	0.963	0.985	0.996	0.995	0.647
**4kra**	0.957	0.957	0.960	0.942	0.990	0.993	0.965	0.905
**4p6w**	0.999	0.997	0.997	0.996	0.998	0.998	0.997	0.892
**5hls**	0.998	0.996	0.992	0.982	0.996	0.993	0.987	0.941
**5kr1**	0.998	0.996	0.997	0.989	0.998	0.998	0.996	0.376
**5p9i**	0.996	0.995	0.993	0.984	0.998	0.996	0.955	0.955
**6jx4**	0.990	0.985	0.980	0.958	0.980	0.990	0.984	0.219
**7rfw (bonded)**	0.997	0.997	0.997	0.994	0.996	0.996	0.993	0.953
**7rfw (non-bonded)**	0.997	0.996	0.994	0.995	0.999	0.999	0.996	0.940
**3qqa**	0.999	0.996	0.993	0.986	0.996	0.995	0.989	0.726
**4xh6**	0.974	0.966	0.979	0.953	0.982	0.987	0.988	0.719
**4yhm**	0.986	0.956	0.986	0.924	0.978	0.995	0.984	0.903
**6v83**	0.995	0.987	0.989	0.982	0.992	0.995	0.990	0.965
**1ajx**	0.993	0.994	0.985	0.980	0.995	0.995	0.987	0.825
**1br5**	0.983	0.976	0.982	0.980	0.995	0.995	0.989	0.690
**1d4i**	0.991	0.990	0.989	0.981	0.997	0.996	0.993	0.890
**1f9g**	0.998	0.947	0.989	0.989	0.945	0.989	0.991	0.837
**1lf3**	0.999	0.996	0.996	0.988	0.998	0.997	0.989	0.712
**4zb8**	0.996	0.989	0.993	0.985	0.995	0.999	0.996	0.951
**1p1o**	0.968	0.990	0.943	0.883	0.991	0.998	0.987	0.976
**1pf7**	0.994	0.982	0.987	0.984	0.985	0.994	0.994	0.657
**1m7q**	0.997	0.994	0.996	0.990	0.996	0.997	0.993	0.610
**1pa9**	0.963	0.935	0.967	0.947	0.959	0.987	0.987	0.709
**1xoz**	0.999	0.997	0.998	0.995	0.998	0.999	0.994	0.907
**2ax6**	0.989	0.984	0.987	0.985	0.995	0.996	0.992	0.472
**2iwu**	0.998	0.981	0.988	0.994	0.984	0.991	0.995	0.766
**2p7z**	0.982	0.980	0.977	0.972	0.973	0.994	0.966	0.637
**1uy9**	0.998	0.999	0.993	0.984	0.996	0.994	0.973	0.926
**3eq7**	0.996	0.994	0.994	0.990	0.999	0.999	0.995	0.844
**5y1y**	0.978	0.906	0.981	0.964	0.938	0.994	0.987	0.395
**5y62**	0.996	0.991	0.989	0.983	0.992	0.988	0.989	0.726
**1nox**	0.998	0.987	0.991	0.987	0.992	0.995	0.989	0.813
**1nj1**	0.990	0.975	0.972	0.979	0.977	0.980	0.989	0.937
**2a3i**	0.999	0.997	0.996	0.998	0.998	0.998	0.995	0.673
**5ct1**	0.989	0.980	0.986	0.941	0.977	0.993	0.980	0.648
**6afa (bonded)**	0.989	0.983	0.982	0.993	0.998	0.998	0.979	0.937
**6afa**	0.999	0.996	0.995	0.996	0.998	0.998	0.986	0.702

**(non-bonded)**								
**6pub**	0.992	0.856	0.956	0.940	0.866	0.973	0.942	-0.061
**1owk**	0.983	0.921	0.984	0.987	0.961	0.997	0.988	0.832
**2qbr**	0.993	0.983	0.981	0.980	0.985	0.997	0.997	0.925
**1yat**	0.999	0.996	0.996	0.996	0.998	0.998	0.996	0.924
**1xp9**	0.997	0.988	0.990	0.991	0.987	0.995	0.993	0.815
**2hxm**	0.992	0.984	0.986	0.958	0.988	0.996	0.979	0.730
**2wca**	0.991	0.986	0.987	0.983	0.998	0.997	0.989	0.908
**2wi4**	0.997	0.995	0.993	0.983	0.998	0.996	0.981	0.911
**1uyg**	0.983	0.988	0.988	0.970	0.989	0.993	0.973	-1.052
**2wi1**	0.995	0.997	0.992	0.992	0.998	0.995	0.993	0.952
**3fr2**	0.997	0.995	0.995	0.986	0.993	0.993	0.991	0.667
**3atm**	0.976	0.991	0.932	0.975	0.983	0.993	0.967	0.726
**5l7i**	0.998	0.998	0.998	0.991	0.999	0.999	0.994	0.973

Supplementary Table 30: Root mean square deviation (RMSD) in angles of quantum refinements. RMSD (°) in angles of the all 50 drugs/inhibitors in the proteins (QR50) from our quantum refinement calculations (**M1-M10**) with respect to **M7** as the reference. Those results for X-ray were taken from the experimental structure without our further refinement.

PDB ID	M1	M3	M5	M6	M8	M10	M6R	X-ray
**1xbb**	1.1	0.8	1.1	1.2	0.5	1.0	1.5	3.4
**2w26**	0.6	0.8	0.8	0.8	0.6	0.7	0.7	3.2
**4hzz**	1.0	2.5	1.0	1.1	2.8	0.6	0.8	5.0
**4kra**	0.9	1.6	1.1	0.9	1.2	0.7	0.9	5.0
**4p6w**	0.4	0.7	0.5	1.0	0.6	0.4	0.8	3.6
**5hls**	0.4	0.9	0.7	0.6	0.9	0.8	0.6	1.9
**5kr1**	0.5	0.8	0.7	1.0	0.4	0.4	1.0	3.0
**5p9i**	0.3	0.5	0.5	0.8	0.3	0.3	2.1	2.1
**6jx4**	0.9	1.0	0.8	0.9	0.9	0.6	0.4	2.2
**7rfw (bonded)**	0.7	0.8	0.9	1.0	0.7	0.6	0.8	3.4
**7rfw (non-bonded)**	1.3	1.3	1.3	1.2	0.6	0.5	1.0	6.2
**3qqa**	0.2	0.6	0.7	0.5	0.6	0.5	0.5	5.3
**4xh6**	0.3	0.6	0.5	0.9	0.5	0.4	0.7	1.9
**4yhm**	0.8	1.1	0.7	1.4	1.2	0.4	0.7	3.1
**6v83**	1.1	2.1	1.8	1.6	2.1	1.7	1.1	3.0
**1ajx**	0.4	0.4	0.6	0.5	0.4	0.4	0.4	3.1
**1br5**	1.0	1.2	1.0	1.1	0.7	0.7	1.0	2.9
**1d4i**	0.3	0.3	0.4	0.5	0.3	0.3	0.6	2.1
**1f9g**	0.4	1.8	1.0	0.7	1.6	0.7	0.6	4.0
**1lf3**	0.3	0.5	0.5	0.6	0.4	0.4	0.6	3.6
**4zb8**	0.8	1.5	0.7	1.0	1.6	0.7	1.0	2.4
**1p1o**	1.6	1.6	1.8	2.4	2.0	0.5	1.0	3.1
**1pf7**	0.9	1.2	1.6	1.2	0.8	0.5	0.8	2.8
**1m7q**	0.3	0.5	0.4	0.6	0.5	0.4	0.5	3.2
**1pa9**	0.9	1.4	1.1	1.3	1.5	1.1	0.9	5.2
**1xoz**	0.2	0.4	0.3	0.4	0.3	0.3	0.4	2.7
**2ax6**	0.4	0.5	0.5	0.5	0.4	0.3	0.8	1.8
**2iwu**	0.7	1.1	1.0	0.9	1.0	0.9	0.8	4.5
**2p7z**	0.8	1.4	0.9	1.1	1.3	0.7	0.7	1.9
**1uy9**	0.4	0.3	0.5	0.8	0.4	0.4	0.9	3.0
**3eq7**	0.3	0.4	0.4	0.7	0.3	0.3	0.5	2.3
**5y1y**	0.7	0.9	0.6	0.7	1.0	0.5	0.4	2.6
**5y62**	0.5	0.6	0.6	1.8	0.5	0.6	0.8	5.2
**1nox**	0.5	0.8	0.6	0.6	0.6	0.5	0.6	5.6
**1nj1**	1.1	1.5	1.3	1.3	1.3	1.3	1.1	3.7
**2a3i**	0.5	0.6	0.5	0.6	0.4	0.4	0.6	3.7
**5ct1**	0.7	0.9	1.4	0.8	1.2	1.1	0.4	3.4
**6afa (bonded)**	0.8	1.3	1.4	1.1	1.0	1.0	2.0	4.9

**6afa (non-bonded)**	1.5	1.6	1.6	1.5	0.9	0.9	1.1	6.7
**6pub**	0.5	0.6	0.7	0.7	0.5	0.4	0.4	1.6
**1owk**	0.7	1.4	0.8	1.1	0.9	0.3	0.7	1.6
**2qbr**	1.1	1.2	1.2	1.2	2.3	0.7	0.6	2.3
**1yat**	0.2	0.5	0.4	0.5	0.4	0.3	0.5	2.6
**1xp9**	0.3	0.6	0.6	0.6	0.7	0.7	0.5	3.4
**2hxm**	0.8	1.9	0.9	1.2	1.7	0.6	1.0	8.6
**2wca**	0.4	0.6	0.5	1.0	0.4	0.3	0.8	2.4
**2wi4**	0.3	0.5	0.5	1.0	0.4	0.4	0.8	2.8
**1uyg**	0.5	0.5	0.6	0.6	0.5	0.5	0.6	2.3
**2wi1**	1.0	0.8	0.8	0.6	0.4	0.3	0.7	4.1
**3fr2**	0.6	1.1	0.6	0.7	1.1	0.5	0.6	3.4
**3atm**	0.3	0.5	0.4	0.3	0.5	0.2	0.3	2.1
**5l7i**	0.4	0.5	0.5	0.8	0.4	0.3	0.8	4.0

Supplementary Table 31: Median absolute deviation (MAD) in bonds of quantum refinements. MAD (°) in angles of the all 50 drugs/inhibitors in the proteins (QR50) from our quantum refinement calculations (**M1-M10**) with respect to **M7** as the reference. Those results for X-ray were taken from the experimental structure without our further refinement.

PDB ID	M1	M3	M5	M6	M8	M10	M6R	X-ray
**1xbb**	0.8	0.6	0.7	0.9	0.3	0.6	1.0	2.6
**2w26**	0.4	0.6	0.6	0.6	0.5	0.5	0.6	2.5
**4hzz**	0.7	1.8	0.7	0.9	1.8	0.4	0.6	4.0
**4kra**	0.7	1.3	0.9	0.7	0.9	0.6	0.7	3.8
**4p6w**	0.3	0.6	0.5	0.8	0.5	0.3	0.6	2.6
**5hls**	0.3	0.6	0.5	0.5	0.6	0.6	0.5	1.6
**5kr1**	0.4	0.5	0.5	0.7	0.3	0.3	0.7	2.2
**5p9i**	0.3	0.3	0.4	0.6	0.3	0.3	1.6	1.6
**6jx4**	0.5	0.7	0.6	0.6	0.5	0.4	0.3	1.7
**7rfw (bonded)**	0.5	0.6	0.7	0.7	0.5	0.5	0.7	2.1
**7rfw (non-bonded)**	0.7	0.7	0.7	0.9	0.5	0.4	0.7	2.6
**3qqa**	0.2	0.5	0.6	0.4	0.4	0.4	0.4	4.1
**4xh6**	0.2	0.5	0.5	0.7	0.4	0.3	0.5	1.5
**4yhm**	0.6	0.9	0.5	0.9	0.7	0.2	0.5	2.1
**6v83**	0.8	1.4	1.4	1.2	1.3	1.2	0.9	2.2
**1ajx**	0.3	0.3	0.4	0.4	0.3	0.3	0.3	2.2
**1br5**	0.8	1.0	0.8	0.9	0.5	0.5	0.8	2.5
**1d4i**	0.2	0.2	0.3	0.3	0.2	0.2	0.4	1.7
**1f9g**	0.4	1.5	0.8	0.6	1.3	0.6	0.5	3.0
**1lf3**	0.2	0.3	0.3	0.5	0.3	0.3	0.4	2.5
**4zb8**	0.6	1.2	0.5	0.7	1.2	0.5	0.8	1.9
**1p1o**	1.1	1.1	1.2	1.5	1.3	0.4	0.8	2.4
**1pf7**	0.7	1.0	1.1	0.9	0.6	0.4	0.7	2.2
**1m7q**	0.2	0.4	0.3	0.5	0.3	0.3	0.4	2.2
**1pa9**	0.6	1.1	0.9	1.0	1.2	0.9	0.8	4.1
**1xoz**	0.2	0.3	0.3	0.4	0.3	0.2	0.3	2.3
**2ax6**	0.3	0.4	0.4	0.4	0.3	0.2	0.7	1.3
**2iwu**	0.5	0.9	0.8	0.7	0.8	0.7	0.7	3.8
**2p7z**	0.6	1.0	0.6	0.8	0.9	0.4	0.5	1.4
**1uy9**	0.3	0.2	0.4	0.7	0.3	0.3	0.7	2.3
**3eq7**	0.2	0.3	0.3	0.5	0.2	0.2	0.4	1.8
**5y1y**	0.5	0.8	0.5	0.6	0.8	0.4	0.4	2.2
**5y62**	0.4	0.5	0.5	1.2	0.4	0.4	0.6	3.8
**1nox**	0.4	0.6	0.5	0.5	0.5	0.4	0.5	4.2
**1nj1**	0.8	1.1	1.0	1.1	0.9	0.9	0.9	2.8
**2a3i**	0.4	0.5	0.4	0.5	0.3	0.3	0.5	2.7
**5ct1**	0.5	0.8	1.1	0.7	1.0	0.8	0.4	2.7
**6afa (bonded)**	0.6	0.9	1.0	0.8	0.7	0.7	1.2	3.8

**6afa (non-bonded)**	1.1	1.3	1.3	1.1	0.5	0.6	0.8	4.7
**6pub**	0.3	0.5	0.6	0.6	0.3	0.3	0.4	1.3
**1owk**	0.5	1.0	0.6	0.9	0.7	0.3	0.6	1.4
**2qbr**	0.7	0.9	0.8	0.8	1.5	0.5	0.5	1.7
**1yat**	0.2	0.4	0.3	0.4	0.3	0.3	0.4	2.0
**1xp9**	0.3	0.5	0.5	0.5	0.6	0.5	0.4	2.6
**2hxm**	0.6	1.4	0.7	0.9	1.2	0.5	0.7	4.6
**2wca**	0.3	0.4	0.4	0.7	0.3	0.3	0.6	1.7
**2wi4**	0.2	0.4	0.4	0.8	0.3	0.3	0.6	2.2
**1uyg**	0.4	0.4	0.4	0.5	0.4	0.4	0.5	1.9
**2wi1**	0.8	0.6	0.7	0.5	0.4	0.3	0.6	3.3
**3fr2**	0.4	0.8	0.4	0.5	0.8	0.3	0.5	2.4
**3atm**	0.3	0.3	0.3	0.2	0.4	0.1	0.3	1.7
**5l7i**	0.3	0.4	0.4	0.6	0.3	0.2	0.6	2.8

Supplementary Table 32: R^2 in angles of quantum refinements. R^2 in angles of the all 50 drugs/inhibitors in the proteins (QR50) from our quantum refinement calculations (**M1-M10**) with respect to **M7** as the reference. Those results for X-ray were taken from the experimental structure without our further refinement.

PDB ID	M1	M3	M5	M6	M8	M10	M6R	X-ray
**1xbb**	0.924	0.966	0.924	0.916	0.982	0.945	0.875	0.324
**2w26**	0.992	0.987	0.986	0.987	0.992	0.989	0.989	0.797
**4hzz**	0.969	0.796	0.969	0.961	0.733	0.990	0.980	0.175
**4kra**	0.966	0.897	0.949	0.963	0.942	0.977	0.966	-0.032
**4p6w**	0.997	0.989	0.994	0.978	0.992	0.996	0.984	0.706
**5hls**	0.997	0.979	0.988	0.991	0.980	0.985	0.991	0.910
**5kr1**	0.994	0.987	0.990	0.979	0.997	0.997	0.978	0.796
**5p9i**	0.997	0.995	0.995	0.987	0.997	0.997	0.892	0.892
**6jx4**	0.982	0.975	0.985	0.982	0.983	0.993	0.997	0.879
**7rfw (bonded)**	0.988	0.984	0.980	0.975	0.990	0.991	0.984	0.740
**7rfw (non-bonded)**	0.978	0.978	0.978	0.982	0.995	0.997	0.987	0.499
**3qqa**	0.999	0.992	0.988	0.994	0.992	0.993	0.994	0.344
**4xh6**	0.982	0.937	0.951	0.866	0.960	0.973	0.928	0.389
**4yhm**	0.980	0.961	0.985	0.936	0.955	0.996	0.985	0.708
**6v83**	0.979	0.927	0.948	0.955	0.927	0.952	0.980	0.851
**1ajx**	0.990	0.990	0.978	0.988	0.989	0.992	0.992	0.471
**1br5**	0.953	0.935	0.957	0.944	0.980	0.981	0.952	0.623
**1d4i**	0.998	0.998	0.997	0.996	0.998	0.999	0.993	0.905
**1f9g**	0.998	0.959	0.987	0.993	0.968	0.993	0.995	0.798
**1lf3**	0.998	0.994	0.995	0.990	0.997	0.996	0.992	0.664
**4zb8**	0.987	0.950	0.990	0.979	0.945	0.990	0.976	0.873
**1p1o**	0.961	0.963	0.953	0.912	0.941	0.996	0.986	0.858
**1pf7**	0.989	0.978	0.964	0.978	0.991	0.996	0.992	0.890
**1m7q**	0.995	0.980	0.991	0.975	0.984	0.988	0.984	0.322
**1pa9**	0.982	0.957	0.974	0.962	0.945	0.972	0.980	0.365
**1xoz**	0.999	0.997	0.998	0.996	0.998	0.999	0.997	0.860
**2ax6**	0.995	0.992	0.993	0.993	0.997	0.998	0.982	0.912
**2iwu**	0.977	0.944	0.954	0.959	0.947	0.955	0.965	0.002
**2p7z**	0.954	0.861	0.947	0.906	0.878	0.965	0.967	0.739
**1uy9**	0.997	0.999	0.996	0.988	0.998	0.998	0.986	0.850
**3eq7**	0.999	0.997	0.998	0.993	0.998	0.998	0.997	0.931
**5y1y**	0.960	0.929	0.969	0.963	0.918	0.975	0.984	0.408
**5y62**	0.996	0.995	0.996	0.959	0.997	0.995	0.992	0.646
**1nox**	0.993	0.980	0.989	0.987	0.990	0.993	0.990	0.036
**1nj1**	0.983	0.966	0.974	0.975	0.973	0.974	0.983	0.794
**2a3i**	0.992	0.988	0.992	0.988	0.994	0.994	0.989	0.551
**5ct1**	0.978	0.959	0.901	0.967	0.927	0.940	0.990	0.444
**6afa (bonded)**	0.991	0.974	0.971	0.984	0.986	0.986	0.944	0.641
**6afa**	0.984	0.983	0.983	0.984	0.995	0.994	0.992	0.699

**(non-bonded)**								
**6pub**	0.953	0.916	0.898	0.897	0.954	0.964	0.956	0.445
**1owk**	0.981	0.924	0.977	0.949	0.965	0.996	0.981	0.896
**2qbr**	0.969	0.963	0.963	0.964	0.862	0.987	0.990	0.873
**1yat**	0.999	0.992	0.994	0.993	0.994	0.996	0.993	0.790
**1xp9**	0.997	0.988	0.988	0.990	0.985	0.988	0.994	0.671
**2hxm**	0.968	0.829	0.965	0.931	0.866	0.982	0.952	-2.545
**2wca**	0.994	0.990	0.993	0.971	0.996	0.997	0.982	0.817
**2wi4**	0.988	0.970	0.969	0.865	0.978	0.979	0.912	-0.074
**1uyg**	0.993	0.993	0.993	0.992	0.994	0.993	0.991	0.877
**2wi1**	0.896	0.946	0.933	0.963	0.982	0.989	0.955	-0.630
**3fr2**	0.993	0.974	0.991	0.990	0.974	0.996	0.991	0.764
**3atm**	0.998	0.996	0.998	0.999	0.996	1.000	0.998	0.937
**5l7i**	0.996	0.994	0.994	0.983	0.997	0.998	0.985	0.621

Supplementary Table 33: Root mean square deviation (RMSD) in dihedrals of quantum refinements. RMSD (°) in dihedrals of the all 50 drugs/inhibitors in the proteins (QR50) from our quantum refinement calculations (**M1-M10**) with respect to **M7** as the reference. Those results for X-ray were taken from the experimental structure without our further refinement.

PDB ID	M1	M3	M5	M6	M8	M10	M6R	X-ray
**1xbb**	1.5	2.6	3.5	4.1	1.8	3.2	3.9	18.3
**2w26**	3.3	2.3	1.9	1.1	1.5	2.9	2.3	7.8
**4hzz**	1.0	2.1	1.2	1.5	2.1	0.7	0.9	8.8
**4kra**	3.8	4.7	3.6	3.3	3.5	1.4	2.7	13.3
**4p6w**	1.3	2.7	2.3	1.7	1.8	1.5	1.2	6.1
**5hls**	0.8	2.5	2.6	0.8	2.6	2.4	0.6	4.3
**5kr1**	0.8	1.2	1.0	1.2	0.7	0.7	2.4	3.2
**5p9i**	0.7	0.7	1.3	0.9	0.5	0.6	3.0	3.0
**6jx4**	2.0	4.2	1.9	2.3	3.0	1.5	0.5	9.9
**7rfw (bonded)**	1.8	1.5	2.0	1.7	0.9	1.3	1.6	8.5
**7rfw (non-bonded)**	8.0	8.0	7.9	6.0	1.3	1.1	2.4	7.9
**3qqa**	0.6	0.7	1.4	0.7	0.9	0.9	0.5	5.4
**4xh6**	1.0	0.7	0.4	1.5	0.8	0.6	1.5	2.6
**4yhm**	1.0	1.0	1.7	1.6	1.6	0.7	2.1	8.5
**6v83**	3.8	5.2	5.3	3.8	5.4	5.1	1.6	8.9
**1ajx**	0.9	1.1	1.2	0.9	0.8	0.7	0.5	12.6
**1br5**	2.6	2.7	2.4	4.1	1.9	1.9	2.7	4.3
**1d4i**	0.5	0.7	0.6	0.7	0.6	0.5	0.8	5.0
**1f9g**	0.6	1.6	1.3	1.0	1.3	0.8	0.9	9.8
**1lf3**	0.8	1.5	1.1	1.5	1.0	1.0	1.6	13.5
**4zb8**	0.9	1.3	1.5	1.3	1.2	1.0	1.0	4.7
**1p1o**	2.9	2.6	3.2	2.9	1.7	0.9	1.7	3.7
**1pf7**	1.4	1.0	6.3	0.9	1.1	0.8	1.0	7.6
**1m7q**	0.6	1.8	0.9	1.5	1.1	1.3	1.0	6.7
**1pa9**	3.2	3.4	3.4	3.1	1.2	1.3	1.1	3.4
**1xoz**	0.2	0.7	0.4	0.7	0.8	0.5	0.7	2.2
**2ax6**	0.6	0.8	0.7	0.7	0.4	0.3	0.4	11.4
**2p7z**	4.0	4.4	3.3	4.3	4.0	1.7	1.4	9.6
**1uy9**	0.5	0.5	0.5	0.9	0.4	0.4	0.7	1.3
**3eq7**	0.7	1.0	1.1	1.1	0.6	0.6	0.6	6.9
**5y1y**	1.1	1.0	3.2	0.5	1.3	0.5	0.3	3.6
**5y62**	0.7	0.9	1.3	10.3	1.1	2.6	1.0	7.3
**1nox**	0.7	1.4	1.2	1.3	1.1	0.8	0.7	6.1
**1nj1**	1.4	1.9	1.8	1.6	1.8	1.7	1.7	9.9
**2a3i**	1.4	1.6	1.2	1.4	1.1	0.9	0.7	7.0
**5ct1**	0.3	0.7	2.4	0.7	0.8	0.3	0.6	1.2
**6afa (bonded)**	0.8	1.0	0.9	1.8	0.7	0.6	1.5	4.6
**6afa**	1.7	1.9	1.9	2.5	0.3	0.3	1.0	3.0

**(non-bonded)**								
**6pub**	1.3	7.7	5.9	3.3	1.8	1.2	0.7	19.8
**1owk**	1.1	1.7	1.4	1.2	1.3	0.8	1.0	5.5
**2qbr**	1.5	2.3	2.4	2.1	3.7	1.6	1.2	6.0
**1yat**	0.3	0.6	0.5	0.7	0.6	0.5	0.7	6.4
**1xp9**	0.9	0.8	0.8	1.3	1.0	0.7	0.8	5.9
**2hxm**	1.5	1.8	1.4	1.7	1.3	0.7	0.8	6.8
**2wca**	0.6	0.8	1.0	0.8	0.5	0.5	0.5	8.9
**2wi4**	0.9	1.2	0.8	0.7	1.8	0.8	0.5	16.3
**1uyg**	3.2	3.2	2.9	2.7	1.0	2.3	3.4	6.9
**2wi1**	4.0	1.6	1.7	0.6	1.1	1.0	0.5	12.5
**3fr2**	0.9	1.4	1.0	0.8	1.8	0.9	0.9	4.6
**3atm**	0.3	0.5	0.4	0.3	1.0	0.2	0.6	2.5
**5l7i**	0.4	0.7	1.0	1.4	0.8	0.8	1.6	25.2

Supplementary Table 34: Median absolute deviation (MAD) in dihedral of quantum refinements. MAD (°) in dihedrals of the all 50 drugs/inhibitors in the proteins (QR50) from our quantum refinement calculations (**M1-M10**) with respect to **M7** as the reference. Those results for X-ray were taken from the experimental structure without our further refinement.

PDB ID	M1	M3	M5	M6	M8	M10	M6R	X-ray
**1xbb**	1.2	1.9	2.3	3.1	1.4	2.3	2.6	15.2
**2w26**	2.6	1.8	1.5	0.9	1.1	2.2	2.0	5.9
**4hzz**	0.8	1.7	1.0	1.3	1.6	0.6	0.8	6.8
**4kra**	2.9	2.9	2.6	2.6	2.5	1.1	2.3	9.2
**4p6w**	0.9	1.9	1.7	1.3	1.3	1.3	1.0	5.1
**5hls**	0.6	2.3	2.1	0.7	2.3	2.0	0.5	3.5
**5kr1**	0.7	1.0	0.8	1.0	0.5	0.5	1.6	2.6
**5p9i**	0.6	0.6	1.0	0.7	0.4	0.5	1.9	1.9
**6jx4**	1.7	3.2	1.5	1.9	2.2	1.1	0.4	7.0
**7rfw (bonded)**	1.3	1.3	1.6	1.4	0.7	1.0	1.2	5.2
**7rfw (non-bonded)**	3.2	3.2	2.9	3.0	1.0	0.9	1.9	5.1
**3qqa**	0.5	0.6	1.3	0.5	0.7	0.7	0.4	4.5
**4xh6**	0.9	0.7	0.3	1.3	0.6	0.5	0.9	2.5
**4yhm**	0.8	0.8	1.4	1.2	1.3	0.5	1.9	6.0
**6v83**	2.6	3.7	4.0	2.8	3.6	4.0	1.3	6.7
**1ajx**	0.8	0.8	1.0	0.7	0.6	0.6	0.4	10.3
**1br5**	1.9	1.9	1.8	3.2	1.3	1.5	2.2	3.4
**1d4i**	0.4	0.6	0.5	0.5	0.5	0.4	0.6	4.0
**1f9g**	0.5	1.3	1.1	0.9	1.1	0.8	0.8	8.0
**1lf3**	0.6	1.2	0.9	1.2	0.8	0.8	1.3	9.3
**4zb8**	0.7	1.0	1.2	0.9	0.9	0.8	0.8	3.7
**1p1o**	2.2	1.8	2.4	2.1	1.3	0.7	1.3	2.9
**1pf7**	1.2	0.8	5.0	0.8	1.0	0.7	0.8	6.6
**1m7q**	0.4	1.5	0.8	1.1	0.9	1.1	0.8	5.2
**1pa9**	2.9	3.2	3.3	2.6	0.9	1.1	0.9	2.9
**1xoz**	0.1	0.6	0.3	0.6	0.7	0.4	0.7	1.8
**2ax6**	0.4	0.7	0.6	0.5	0.4	0.3	0.4	7.0
**2p7z**	3.2	3.5	2.4	3.4	3.1	1.1	1.0	7.8
**1uy9**	0.4	0.4	0.4	0.7	0.3	0.3	0.6	1.1
**3eq7**	0.6	0.9	0.9	0.9	0.5	0.5	0.5	6.4
**5y1y**	1.0	1.0	2.9	0.4	1.2	0.5	0.2	3.0
**5y62**	0.6	0.8	1.2	9.2	0.9	2.3	0.9	6.1
**1nox**	0.6	1.1	0.9	1.0	0.9	0.7	0.6	4.5
**1nj1**	1.2	1.5	1.3	1.2	1.4	1.4	1.4	7.3
**2a3i**	1.1	1.2	1.0	1.2	0.8	0.9	0.5	5.1
**5ct1**	0.2	0.7	2.4	0.5	0.6	0.3	0.5	1.0
**6afa (bonded)**	0.6	0.8	0.8	1.7	0.6	0.5	1.3	3.9
**6afa**	1.6	1.7	1.7	2.2	0.3	0.3	0.8	2.3

**(non-bonded)**								
**6pub**	1.0	6.8	5.0	2.7	1.6	0.9	0.6	17.6
**1owk**	0.8	1.4	1.1	0.9	1.0	0.7	0.9	4.8
**2qbr**	1.2	1.8	1.9	1.4	2.9	1.3	1.0	5.0
**1yat**	0.2	0.5	0.5	0.5	0.5	0.4	0.5	5.1
**1xp9**	0.7	0.6	0.6	1.0	0.8	0.6	0.6	4.8
**2hxm**	1.2	1.4	1.1	1.4	1.1	0.5	0.7	5.1
**2wca**	0.4	0.6	0.7	0.7	0.4	0.4	0.4	7.5
**2wi4**	0.8	1.0	0.7	0.6	1.4	0.7	0.4	14.0
**1uyg**	2.5	2.5	2.3	2.1	0.8	1.9	2.6	5.0
**2wi1**	3.1	1.6	1.5	0.5	0.8	0.8	0.4	11.0
**3fr2**	0.7	1.2	0.8	0.8	1.5	0.8	0.7	3.3
**3atm**	0.2	0.4	0.4	0.3	0.6	0.2	0.5	2.3
**5l7i**	0.3	0.5	0.8	1.2	0.7	0.7	1.4	19.5

Supplementary Table 35: R^2 in dihedral of quantum refinements. R^2 in dihedral of the all 50 drugs/inhibitors in the proteins (QR50) from our quantum refinement calculations (**M1-M10**) with respect to **M7** as the reference. Those results for X-ray were taken from the experimental structure without our further refinement.

PDB ID	M1	M3	M5	M6	M8	M10	M6R	X-ray
**1xbb**	1.000	0.999	0.999	0.999	1.000	0.999	0.999	0.973
**2w26**	0.999	0.999	1.000	1.000	1.000	0.999	0.999	0.993
**4hzz**	1.000	1.000	1.000	1.000	1.000	1.000	1.000	0.994
**4kra**	0.999	0.998	0.999	0.999	0.999	1.000	0.999	0.981
**4p6w**	1.000	0.999	1.000	1.000	1.000	1.000	1.000	0.997
**5hls**	1.000	0.999	0.999	1.000	0.999	1.000	1.000	0.998
**5kr1**	1.000	1.000	1.000	1.000	1.000	1.000	1.000	0.999
**5p9i**	1.000	1.000	1.000	1.000	1.000	1.000	0.999	0.999
**6jx4**	1.000	0.999	1.000	1.000	0.999	1.000	1.000	0.993
**7rfw (bonded)**	1.000	1.000	1.000	1.000	1.000	1.000	1.000	0.994
**7rfw (non-bonded)**	0.995	0.995	0.995	0.997	1.000	1.000	1.000	0.995
**3qqa**	1.000	1.000	1.000	1.000	1.000	1.000	1.000	0.998
**4xh6**	1.000	1.000	1.000	1.000	1.000	1.000	1.000	0.999
**4yhm**	1.000	1.000	1.000	1.000	1.000	1.000	1.000	0.993
**6v83**	0.999	0.998	0.998	0.999	0.998	0.998	1.000	0.994
**1ajx**	1.000	1.000	1.000	1.000	1.000	1.000	1.000	0.987
**1br5**	0.999	0.999	1.000	0.999	1.000	1.000	0.999	0.998
**1d4i**	1.000	1.000	1.000	1.000	1.000	1.000	1.000	0.998
**1f9g**	1.000	1.000	1.000	1.000	1.000	1.000	1.000	0.993
**1lf3**	1.000	1.000	1.000	1.000	1.000	1.000	1.000	0.983
**4zb8**	1.000	1.000	1.000	1.000	1.000	1.000	1.000	0.998
**1p1o**	0.999	0.999	0.999	0.999	1.000	1.000	1.000	0.999
**1pf7**	1.000	1.000	0.996	1.000	1.000	1.000	1.000	0.994
**1m7q**	1.000	1.000	1.000	1.000	1.000	1.000	1.000	0.994
**1pa9**	0.999	0.999	0.999	0.999	1.000	1.000	1.000	0.999
**1xoz**	1.000	1.000	1.000	1.000	1.000	1.000	1.000	0.999
**2ax6**	1.000	1.000	1.000	1.000	1.000	1.000	1.000	0.989
**2p7z**	0.998	0.998	0.999	0.998	0.998	1.000	1.000	0.991
**1uy9**	1.000	1.000	1.000	1.000	1.000	1.000	1.000	1.000
**3eq7**	1.000	1.000	1.000	1.000	1.000	1.000	1.000	0.996
**5y1y**	1.000	1.000	0.999	1.000	1.000	1.000	1.000	0.998
**5y62**	1.000	1.000	1.000	0.991	1.000	0.999	1.000	0.995
**1nox**	1.000	1.000	1.000	1.000	1.000	1.000	1.000	0.997
**1nj1**	1.000	1.000	1.000	1.000	1.000	1.000	1.000	0.991
**2a3i**	1.000	1.000	1.000	1.000	1.000	1.000	1.000	0.995
**5ct1**	1.000	1.000	1.000	1.000	1.000	1.000	1.000	1.000
**6afa (bonded)**	1.000	1.000	1.000	1.000	1.000	1.000	1.000	0.998
**6afa (non-bonded)**	1.000	1.000	1.000	1.000	1.000	1.000	1.000	0.999

**6pub**	1.000	0.995	0.997	0.999	1.000	1.000	1.000	0.970
**1owk**	1.000	1.000	1.000	1.000	1.000	1.000	1.000	0.997
**2qbr**	1.000	1.000	1.000	1.000	0.999	1.000	1.000	0.997
**1yat**	1.000	1.000	1.000	1.000	1.000	1.000	1.000	0.997
**1xp9**	1.000	1.000	1.000	1.000	1.000	1.000	1.000	0.997
**2hxm**	1.000	1.000	1.000	1.000	1.000	1.000	1.000	0.997
**2wca**	1.000	1.000	1.000	1.000	1.000	1.000	1.000	0.994
**2wi4**	1.000	1.000	1.000	1.000	1.000	1.000	1.000	0.971
**1uyg**	0.999	0.999	0.999	0.999	1.000	1.000	0.999	0.996
**2wi1**	0.998	1.000	1.000	1.000	1.000	1.000	1.000	0.980
**3fr2**	1.000	1.000	1.000	1.000	1.000	1.000	1.000	0.998
**3atm**	1.000	1.000	1.000	1.000	1.000	1.000	1.000	0.999
**5l7i**	1.000	1.000	1.000	1.000	1.000	1.000	1.000	0.944

Supplementary Table 36: Root mean square deviation (RMSD) of the optimized structures in the gas phase and quantum refinements. RMSD of the all 50 drugs/inhibitors (QR50) optimized in gas phase using ANI-2x, AIQM1 and GFN2-xTB compared to the DFT (ωB97X-D/6-31G(d)) method and RMSD of the all 50 drugs/inhibitors refined in the proteins using **M8**, **M10** and **M6R** with respect to **M7** as the reference (the charged cases are highlighted by red).

PDB ID	Gas phase			QR		
	ANI-2x	AIQM1	GFN2-xTB	M8	M10	M6R
1xbb	0.81	0.10	0.39	0.06	0.09	0.06
4hzz	0.40	0.22	0.12	0.04	0.02	0.02
5p9i	0.52	0.62	0.18	0.01	0.01	0.01
6jx4	0.78	0.20	0.23	0.03	0.02	0.05
2w26	0.23	1.05	0.10	0.02	0.03	0.04
4kra	0.90	0.98	0.60	0.05	0.03	0.03
4p6w	0.15	0.20	0.13	0.02	0.02	0.03
5hls	0.22	0.21	0.08	0.02	0.01	0.03
5kr1	0.44	0.26	0.26	0.01	0.01	0.02
7rfw (bonded)	0.74	1.43	1.71	0.02	0.02	0.04
7rfw (non-bonded)	0.35	0.96	0.54	0.03	0.02	0.07
1ajx	0.34	0.17	0.16	0.01	0.01	0.01
1br5	0.88	0.55	0.91	0.03	0.03	0.04
1d4i	2.01	1.50	0.42	0.01	0.01	0.01
1f9g	0.08	0.03	0.07	0.03	0.02	0.02
1lf3	1.46	0.54	0.39	0.01	0.02	0.03
1m7q	0.18	0.18	0.29	0.01	0.02	0.01
1nj1	0.32	0.39	0.56	0.04	0.04	0.03
1nox	0.27	0.17	0.16	0.01	0.01	0.01
1owk	0.31	0.08	0.08	0.03	0.01	0.02
1p1o	0.17	0.42	0.50	0.03	0.01	0.02
1pa9	0.81	0.17	0.16	0.03	0.03	0.02
1pf7	1.22	0.24	0.11	0.02	0.01	0.02
1uy9	0.14	0.26	0.10	0.01	0.01	0.01
1uyg	1.28	1.16	1.18	0.02	0.02	0.03
1xoz	0.41	0.36	0.78	0.01	0.01	0.01
1xp9	1.56	0.21	0.22	0.02	0.01	0.01
1yat	1.43	1.44	0.98	0.01	0.01	0.01
2a3i	0.12	0.08	0.07	0.01	0.01	0.02
2ax6	0.06	0.07	0.04	0.01	0.01	0.01
2hxm	0.40	0.15	0.23	0.03	0.01	0.02
2iwu	0.27	0.27	0.26	0.04	0.04	0.04
2p7z	0.53	0.64	0.16	0.08	0.02	0.03
2qbr	1.97	1.33	1.42	0.04	0.03	0.02
2wca	0.68	0.70	0.39	0.01	0.01	0.01
2wi1	0.52	0.15	0.15	0.01	0.01	0.01
2wi4	0.25	0.20	0.33	0.01	0.01	0.02

**3atm**	0.03	0.01	0.02	0.01	0.00	0.01
**3eq7**	0.49	0.25	0.22	0.01	0.01	0.01
**3fr2**	0.26	0.49	0.58	0.02	0.01	0.02
**3qqa**	3.34	3.25	0.88	0.01	0.01	0.01
**4xh6**	0.45	0.46	0.28	0.02	0.01	0.01
**4yhm**	1.03	0.13	0.53	0.03	0.01	0.02
**4zb8**	0.90	0.46	0.18	0.04	0.02	0.03
**5ct1**	0.85	0.18	0.28	0.02	0.03	0.02
**5y1y**	0.15	0.02	0.02	0.02	0.01	0.01
**5y62**	1.03	0.98	1.23	0.01	0.03	0.02
**6pub**	0.05	0.02	0.10	0.03	0.01	0.02
**6v83**	1.57	1.42	1.41	0.08	0.08	0.04
**5l7i**	1.60	1.81	1.80	0.02	0.01	0.03
**6afa (bonded)**	0.44	0.53	0.55	0.03	0.03	0.04
**6afa (non-bonded)**	1.34	0.21	0.20	0.03	0.03	0.05

Supplementary Table 37: Computational efficiency of quantum refinements. Computational cost (CPU core-hours) of different quantum refinements for different systems. All **M3** (sole MLP-based) computations were performed on a single CPU, while the other calculations (**M1**, **M5-M10**) were performed on 12/24 Intel(R) Xeon(R) Platinum 9242 CPU.

	**M1**	**M3**	**M5**	**M6**	**M7**	**M8**	**M10**	**M6R**
**3qqa**	58.8	8.4	24.6	1.7	67.9	26.7	19.6	9.2
**4xh6**	56.2	1.7	5.7	18.5	85.0	55.8	48.6	28.3
**4yhm**	110.3	7.2	42.8	13.5	163.6	60.5	20.2	446.4
**6v83**	54.8	3.4	24.4	18.4	288.4	238.3	185.5	86.1
**1ajx**	17.2	12.3	11.7	10.6	791.0	264.3	530.7	55.5
**1br5**	65.1	0.9	6.3	24.1	93.0	7.8	11.2	3.3
**1d4i**	272.0	2.8	7.1	5.3	439.4	23.2	367.8	155.1
**1f9g**	142.6	15.2	82.6	87.8	363.6	42.4	90.5	110.9
**1lf3**	96.0	4.0	41.4	7.1	148.4	18.4	111.6	20.5
**4zb8**	64.8	4.9	32.6	6.4	98.2	30.8	40.3	3.8
**1p1o**	3.8	1.5	6.3	3.5	26.4	16.7	28.9	18.9
**1pf7**	23.7	2.7	43.9	10.7	11.3	0.2	12.3	0.2
**1m7q**	117.3	2.6	89.2	26.9	194.4	79.8	495.7	38.6
**1pa9**	25.7	0.8	6.7	11.0	16.6	26.9	31.2	38.1
**1xoz**	154.1	1.3	13.5	16.6	248.7	9.8	28.8	32.1
**2ax6**	105.7	2.5	4.7	6.0	13.1	27.7	33.6	12.3
**2iwu**	126.6	2.0	25.0	21.3	280.6	66.8	9.4	14.3
**2p7z**	24.4	0.6	25.7	1.1	79.3	13.1	48.7	111.6
**1uy9**	42.8	1.1	8.6	14.6	20.1	45.9	92.1	32.3
**3eq7**	22.3	18.2	10.5	43.8	57.6	34.4	34.3	62.7
**5y1y**	7.3	4.6	5.9	1.5	7.5	3.9	16.0	4.2
**5y62**	105.0	5.4	6.4	56.3	225.9	56.7	57.2	28.7
**1nox**	111.9	6.7	11.7	1.3	92.6	14.4	316.6	78.3
**1nj1**	57.1	2.0	28.2	88.5	843.2	29.0	24.1	285.6
**2a3i**	12.1	3.3	50.9	6.3	231.2	178.5	33.7	72.3
**5ct1**	9.0	4.1	28.8	37.1	5.3	4.7	29.7	43.3
**6afa (bonded)**	6.3	2.0	2.9	3.0	21.6	8.2	7.5	14.2
**6afa (non-bonded)**	12.9	2.7	12.1	8.6	53.5	47.9	41.1	23.6
**6pub**	101.9	11.7	17.8	20.4	190.1	64.5	41.8	40.2
**1owk**	28.9	2.0	10.2	3.7	161.0	48.6	58.2	29.7
**2qbr**	166.3	20.7	5.2	5.0	76.2	30.1	7.6	14.4
**1yat**	91.1	1.4	17.1	6.6	192.4	19.2	9.0	117.0
**1xp9**	477.9	5.2	18.4	70.2	319.9	827.6	57.8	80.8
**2hxm**	14.3	2.9	6.6	4.8	17.5	6.5	18.1	10.0
**2wca**	42.8	3.6	14.8	11.8	27.2	25.4	9.0	18.4
**2wi4**	23.5	4.8	9.8	2.6	78.2	14.4	10.4	16.5
**1uyg**	4.7	4.6	7.3	17.8	197.6	20.0	20.3	84.6
**2wi1**	22.5	11.0	11.1	1.8	10.5	9.4	20.1	40.8
**3fr2**	98.9	5.1	9.9	3.5	118.1	6.6	49.9	9.4

**3atm**	21.8	1.4	72.4	7.8	4.8	12.6	66.3	5.4
**5l7i**	13.7	86.3	61.5	14.2	184.7	255.3	176.8	131.7

(i) 1XBB (Imatinib in spleen tyrosine kinase)

Protein preparations:

Resolution: 1.57 Å

Ligand: STI (Imatinib, Gleevec); $C_{29}H_{31}N_7O$

Residue flipped: Chain A: ASN457, HIS639

Addition of the missing atoms: 73 missing heavy atoms

Protonation states (pH = 8.5): Chain A: HID465, HID476, HID492, HID506, HID638, HID639

Optimized region: STI

High layer: STI

Medium layer: Chain A: LEU377, SER379, VAL385, ALA400, VAL433, MET448, GLU449, MET450, ALA451, GLU452, GLY454, PRO455, LYS458, LEU501, WAT676, WAT729, WAT803, WAT831, WAT876, WAT899, WAT900, WAT916, WAT949, WAT997, WAT998

$\omega_\alpha = 0.22622$

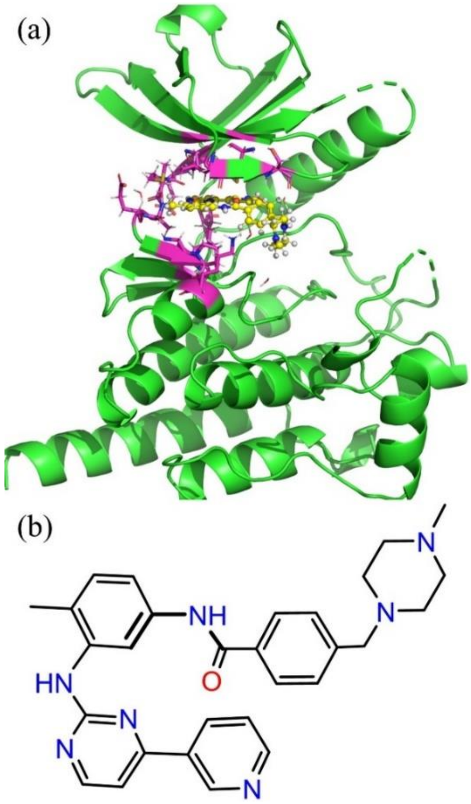

Supplementary Figure 18: Imatinib in spleen tyrosine kinase. (**a**) Crystal structure of spleen tyrosine kinase with imatinib (STI). ONIOM layers by different colors: yellow: high layer; red: medium layer; green: low layer. Ligand imatinib is presented in stick and balls. (**b**) Structure of imatinib.

Quantum refined structural results:

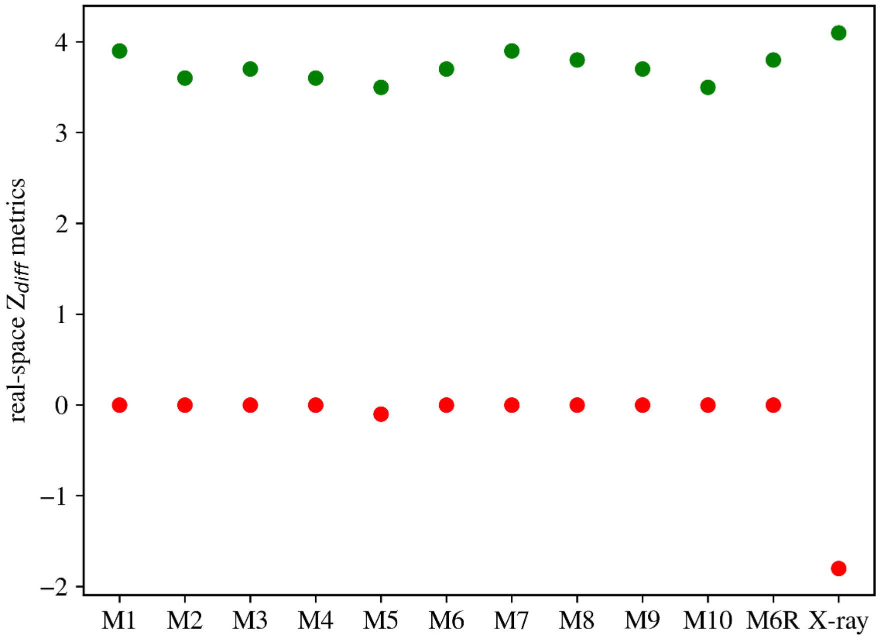

Supplementary Figure 19: Real-space Z-difference (RSZD) of imatinib. RSZD+ (green) and RSZD- (red) scores of imatinib (STI) in spleen tyrosine kinase from various quantum refinement schemes (**M1-M10**). Those results for X-ray were taken from the experimental structure without our further refinement.

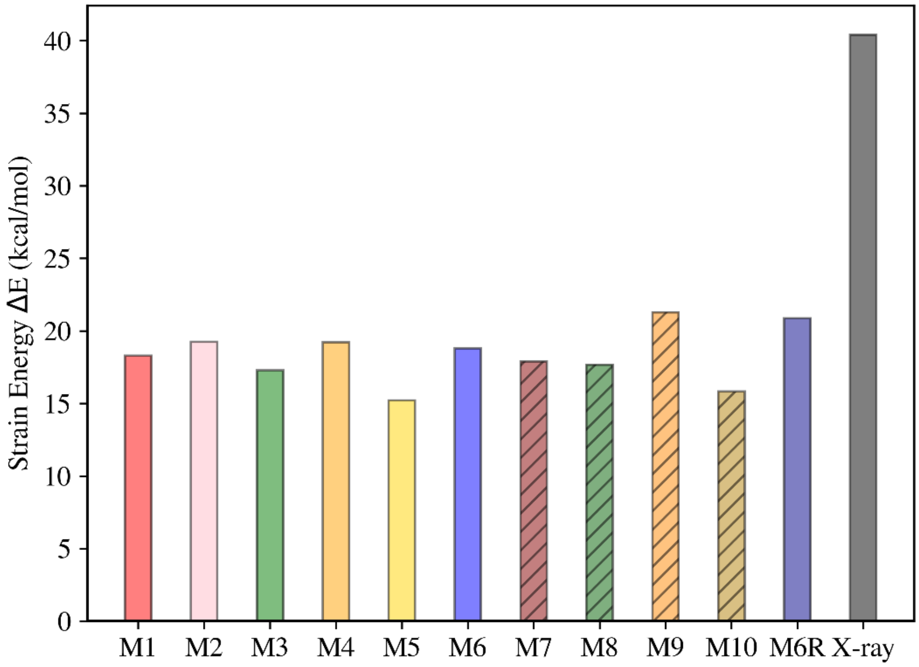

Supplementary Figure 20: Strain energy of imatinib. Strain energy (ΔE, kcal·mol^{-1}) at ωB97X-D/6-31G(d) level for imatinib (STI) in spleen tyrosine kinase determined by various quantum refinement schemes (**M1-M10**). Those results for X-ray were taken from the experimental structure without our further refinement.

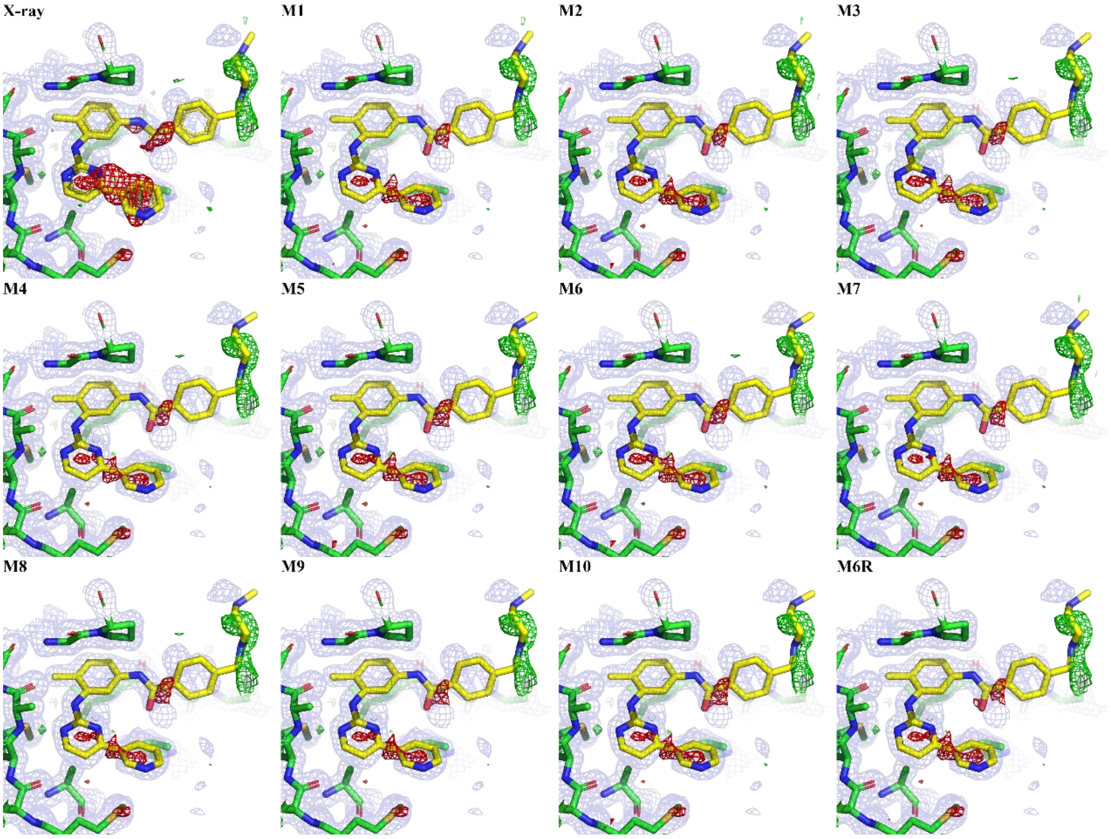

Supplementary Figure 21: Electron density maps of imatinib. Structures for imatinib (STI) in spleen tyrosine kinase from various quantum refinement schemes (**M1**-**M10**), including the electron density maps (2mFo-DFc maps, contoured at 1.0 σ (blue), mFo-DFc maps, contoured at +3.0 σ (green), and mFo-DFc maps, contoured at -3.0 σ (red)). Those results for X-ray were taken from the experimental structure without our further refinement.

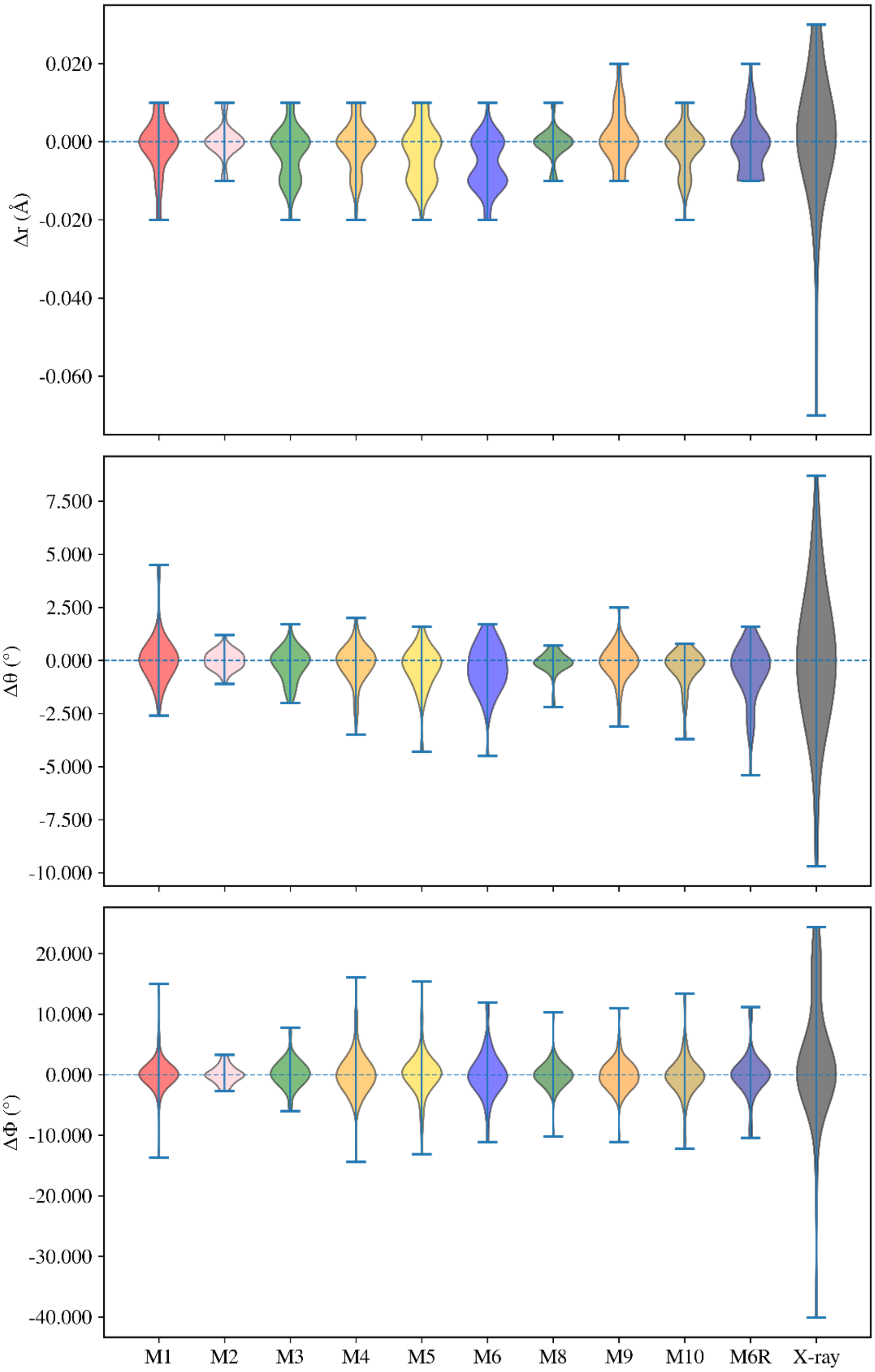

Supplementary Figure 22: Key coordinates of quantum refinement results of imatinib. Deviation in the refined bond distances (Δr, n = 41), angles (Δθ, n = 56) and dihedrals (Δϕ, n = 20) of imatinib (STI) in spleen tyrosine kinase from various quantum refinement schemes (**M1-M10**) and X-ray structure which are compared to those obtained from the most reliable **M7** scheme. The solid line represents the upper and lower values.

(ii) 5P9I (Ibrutinib in Bruton's tyrosine kinase)

Protein preparations

Resolution: 1.45 Å

Ligand: 1E8 (Ibrutinib, Imbruvica); $C_{25}H_{24}N_6O_2$

Residue flipped: Chain A: GLN424, GLN459, GLN494

Addition of the missing atoms: 66 missing heavy atoms

Protonation states (pH = 7.0): HID454, HIE491, HID519, HID609, HID620, HID635

Optimized region: 1E8

High layer: 1E8

Medium layer: LEU408, THR410, GLY411, VAL416, ALA428, LYS430, MET449, VAL458, ILE472, THR474, GLU475, TYR476, MET477, GLY480, CYS481, ASN484, LEU528, SER538, ASP539, PHE540, LEU542, WAT837, WAT862, WAT933, WAT939, WAT964, WAT965

$\omega_\alpha = 0.14880$

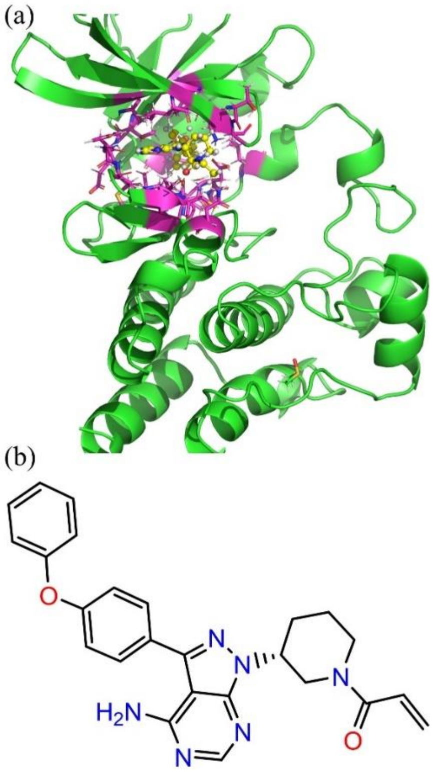

Supplementary Figure 23: Ibrutinib in Bruton's tyrosine kinase. (**a**) Crystal structure of Bruton's tyrosine kinase with ibrutinib (1E8). ONIOM layer by different color: yellow: high layer; red: medium layer; green: low layer. Ligand imatinib is presented in stick and balls. (**b**) Structure of ibrutinib.

Quantum refined structural results:

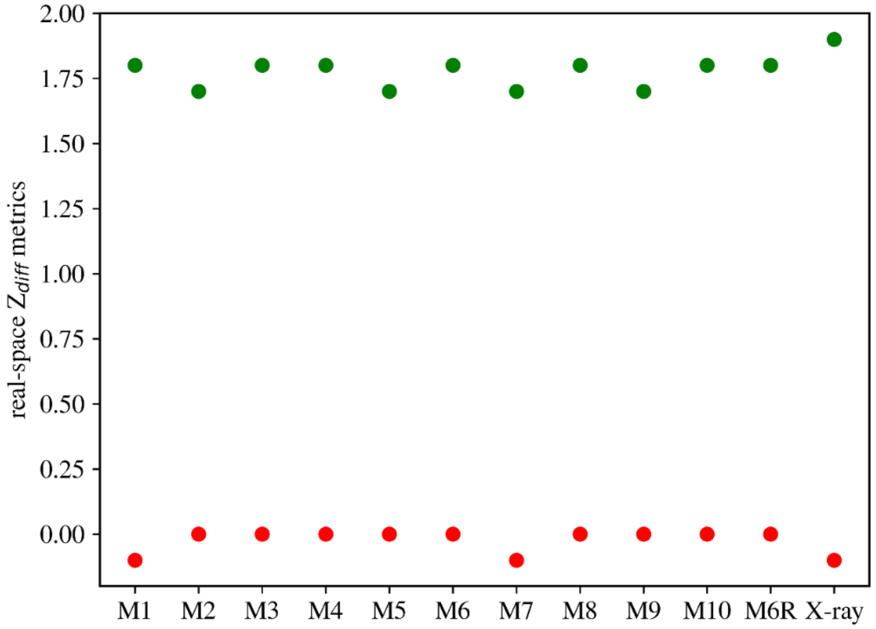

Supplementary Figure 24: Real-space Z-difference (RSZD) of ibrutinib. RSZD+ (green) and RSZD- (red) scores of ibrutinib (1E8) in Bruton's tyrosine kinase from various quantum refinement schemes (**M1-M10**). Those results for X-ray were taken from the experimental structure without our further refinement.

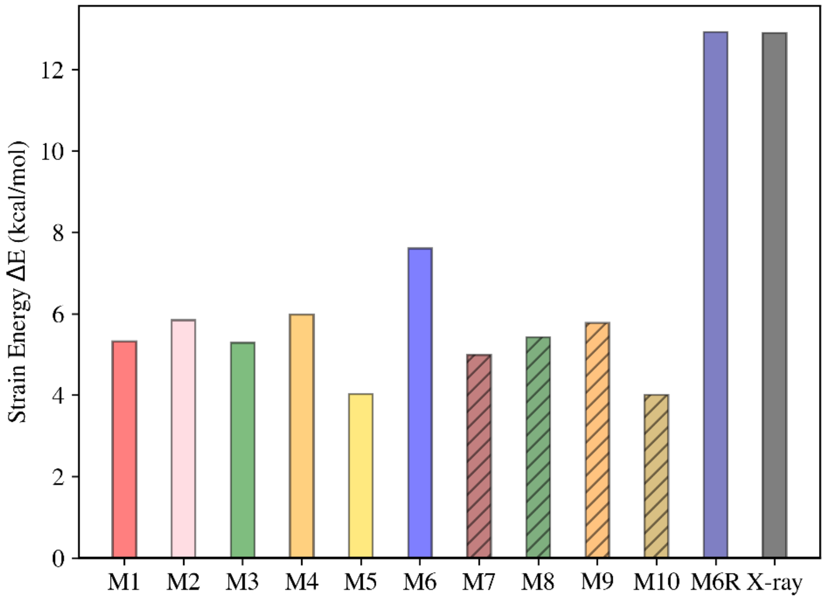

Supplementary Figure 25: Strain energy of ibrutinib. Strain energy (ΔE, kcal·mol^{-1}) at ωB97X-D/6-31G(d) level for ibrutinib (1E8) in Bruton's tyrosine kinase determined by various quantum refinement schemes (**M1-M10**). Those results for X-ray were taken from the experimental structure without our further refinement.

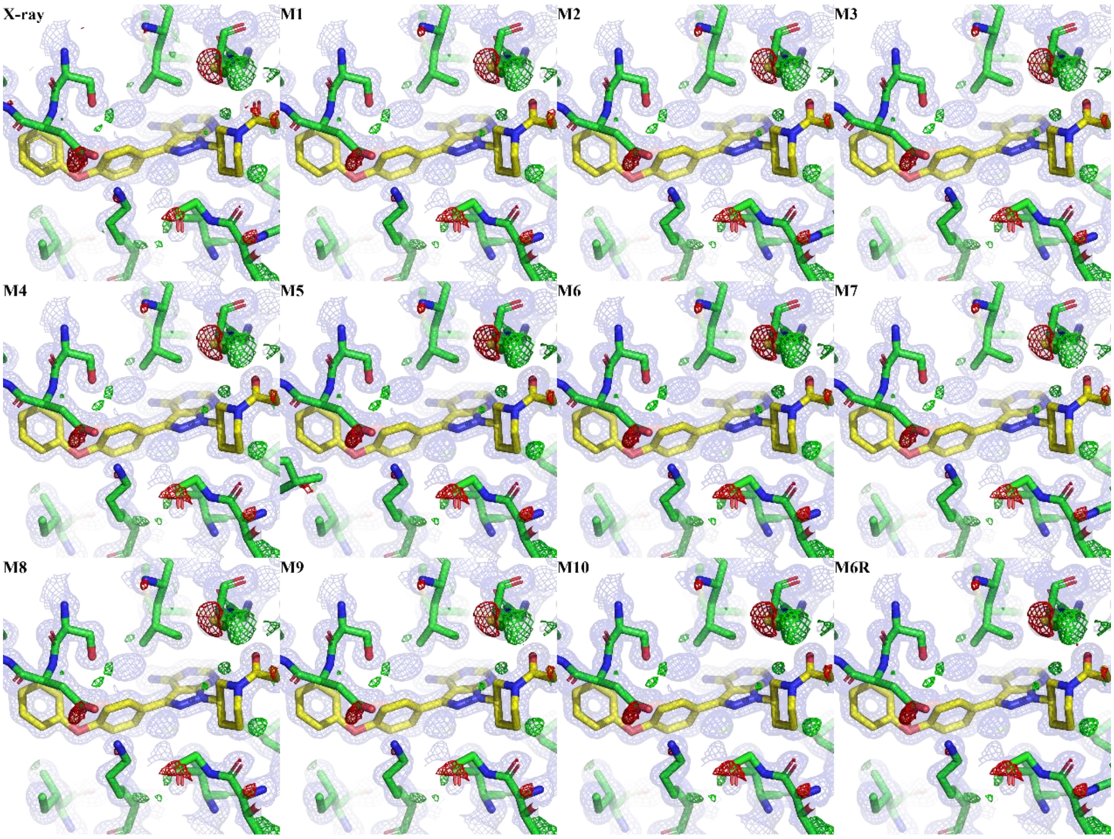

Supplementary Figure 26: Electron density maps of ibrutinib. Structures for ibrutinib (1E8) in Bruton's tyrosine kinase from various quantum refinement schemes (**M1**-**M10**), including the electron density maps (2mFo-DFc maps, contoured at 1.0 σ (blue), mFo-DFc maps, contoured at +3.0 σ (green), and mFo-DFc maps, contoured at -3.0 σ (red)). Those results for X-ray were taken from the experimental structure without our further refinement.

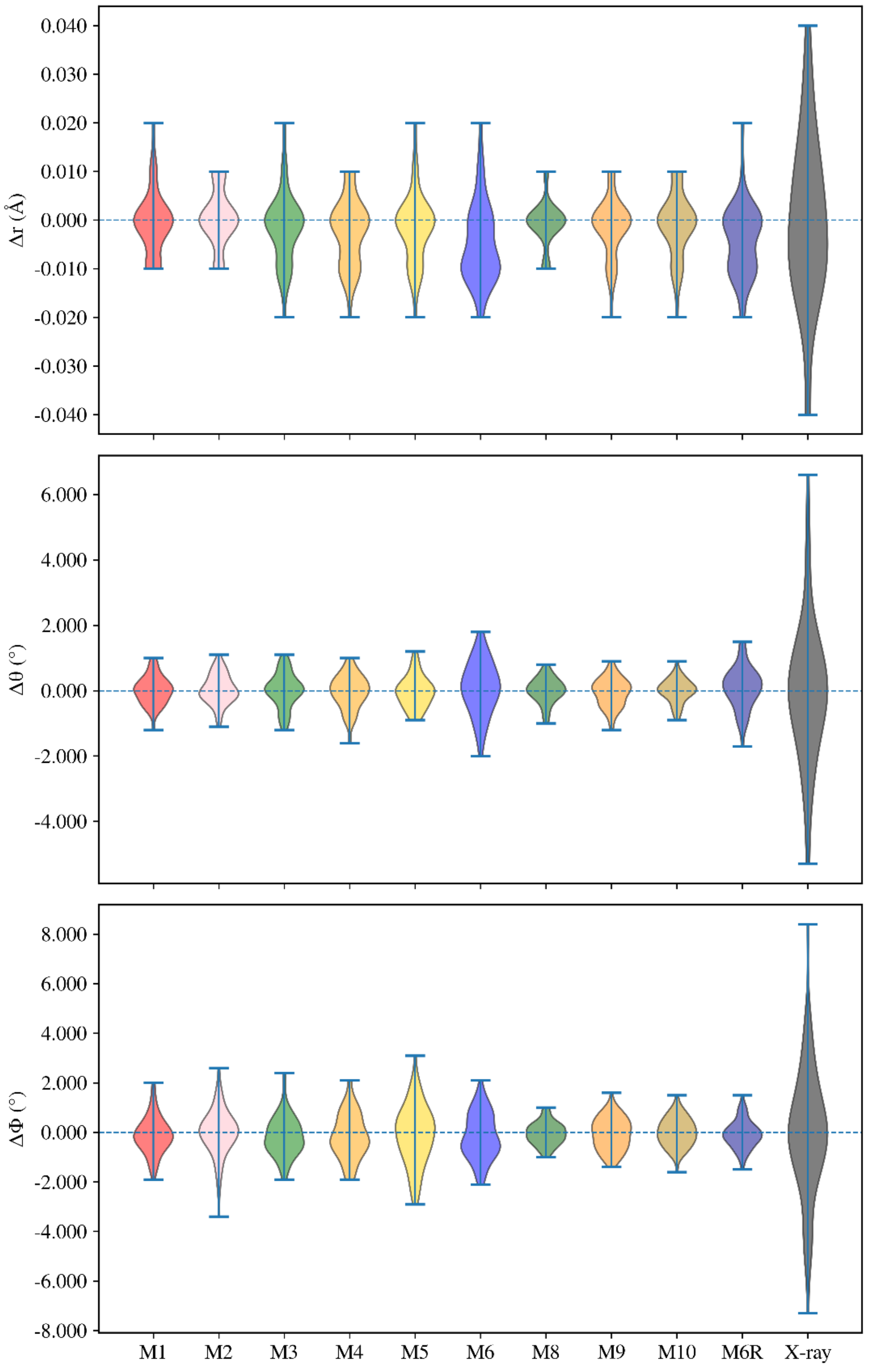

Supplementary Figure 27: Key coordinates of quantum refinement results of ibrutinib. Deviation in the refined bond distances (Δr, n = 37), angles (Δθ, n = 52) and dihedrals (Δϕ, n = 18) of ibrutinib (1E8) in Bruton's tyrosine kinase from various quantum refinement schemes (**M1-M10**) and X-ray structure which are compared to those obtained from the most reliable **M7** scheme. The solid line represents the upper and lower values.

(iii) 4HZZ (Oseltamivir in influenza neuraminidase)

Protein preparations

Resolution: 1.60 Å

Ligand: G39 (Oseltamivir, Tamiflu); $C_{14}H_{24}N_2O_4$

Residue flipped: ASN104, ASN111, ASN126, GLN226, ASN267, ASN306, ASN388.

Protonation states (pH = 8.0): HID101, HID144, HID191, HID322, LYN350, HIE389

CYX-CYX: CYX92-CYX417, CYX124-CYX129, CYX175-CYX193, CYX183-CYX230, CYX232-CYX237, CYX278-CYX291, CYX280-CYX289, CYX318-CYX337, CYX421-CYX447

Optimized region: G39

High layer: G39

Medium layer: ARG118, GLU119, ASP151, ARG152, TRP178, SER179, ILE222, ARG224, ALA246, GLU276, GLU277, ARG292, ARG371, TYR406, WAT615, WAT675, WAT693, WAT774, WAT789, WAT807, WAT812

$\omega_\alpha = 0.14795$

For the N-Glycosylation in 4HZZ, Amber GLYCAM_06j-1 force filed parameters were applied using Ambertools 21.10. As shown in Supplementary Fig. 28, the UYB unit in N-Glycosylation was connected to the ND2 atom of ASN residue (renamed NLN in GLYCAM_06j-1 force filed), the 0YB and 0fA units were connected the O4 and O6 in UYB, respectively.

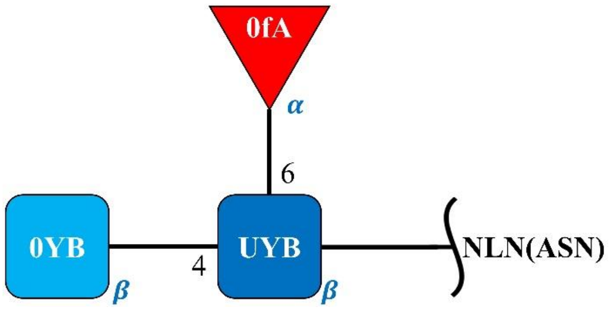

Supplementary Figure 28: 2D diagram of N-Glycosylation in 4HZZ

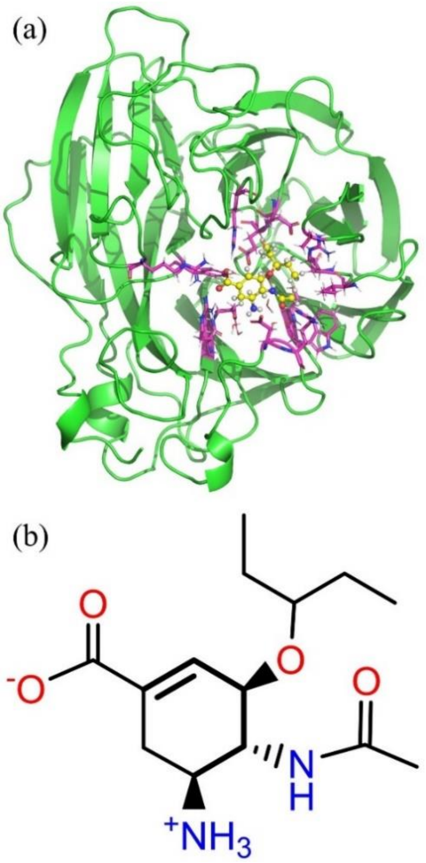

Supplementary Figure 29: Oseltamivir in influenza neuraminidase. (**a**) Crystal structure of influenza neuraminidase with oseltamivir (G39). ONIOM layer by different color: yellow: high layer; red: medium layer; green: low layer. Ligand oseltamivir is presented in stick and balls. (**b**) Structure of oseltamivir.

Quantum refined structural results:

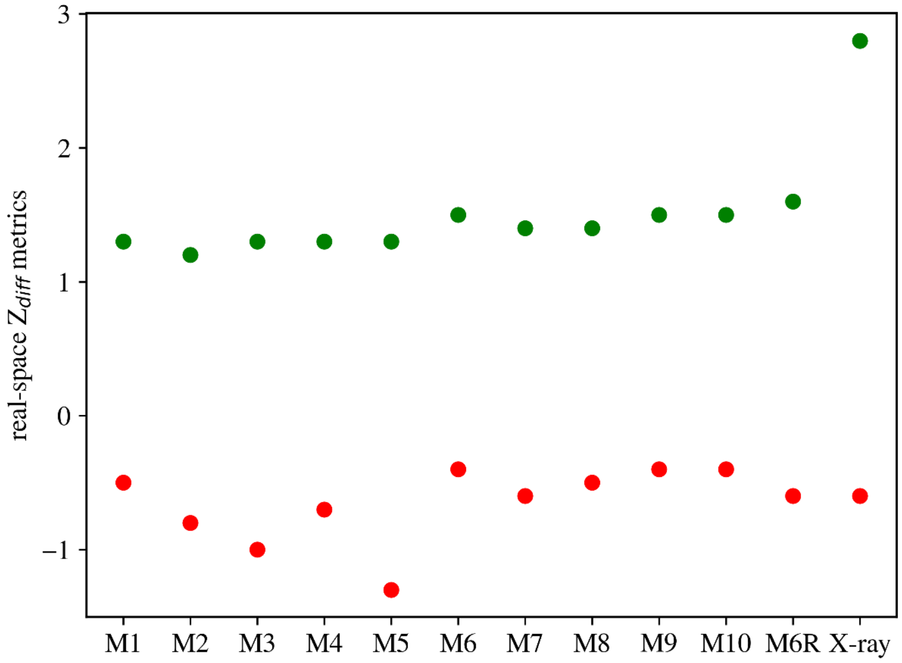

Supplementary Figure 30: Real-space Z-difference (RSZD) of oseltamivir. RSZD+ (green) and RSZD- (red) scores of oseltamivir (G39) in influenza neuraminidase from various quantum refinement schemes (**M1**-**M10**). Those results for X-ray were taken from the experimental structure without our further refinement.

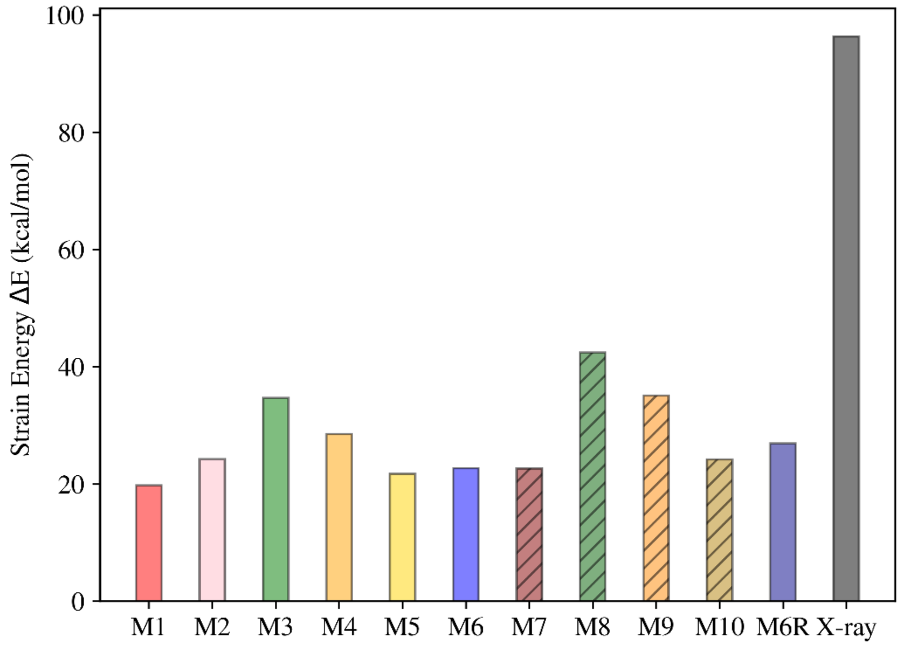

Supplementary Figure 31: Strain energy of oseltamivir. Strain energy (ΔE, kcal·mol^{-1}) at ωB97X-D/6-31G(d) level for oseltamivir (G39) in influenza neuraminidase determined by various quantum refinement schemes (**M1**-**M10**). Those results for X-ray were taken from the experimental structure without our further refinement.

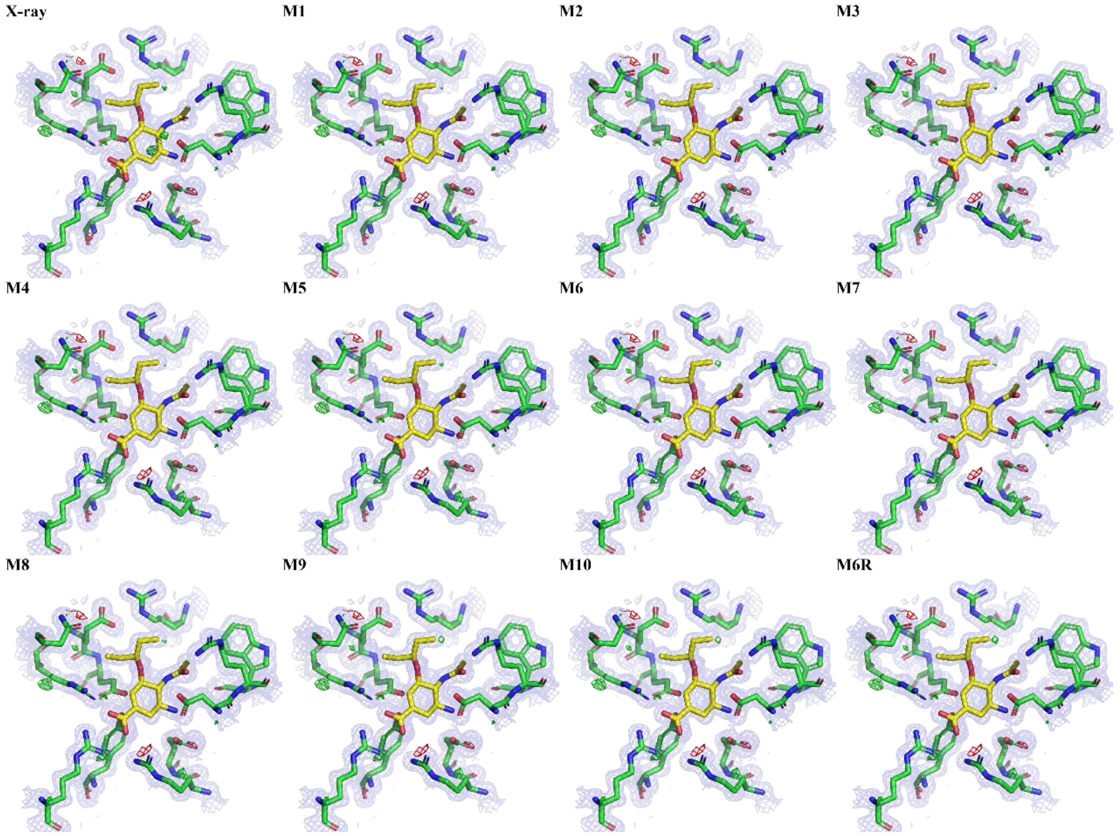

Supplementary Figure 32: Electron density maps of oseltamivir. Structures for oseltamivir (G39) in influenza neuraminidase from various quantum refinement schemes (**M1-M10**), including the electron density maps (2mFo-DFc maps, contoured at 1.0 σ (blue), mFo-DFc maps, contoured at +3.0 σ (green), and mFo-DFc maps, contoured at -3.0 σ (red)). Those results for X-ray were taken from the experimental structure without our further refinement.

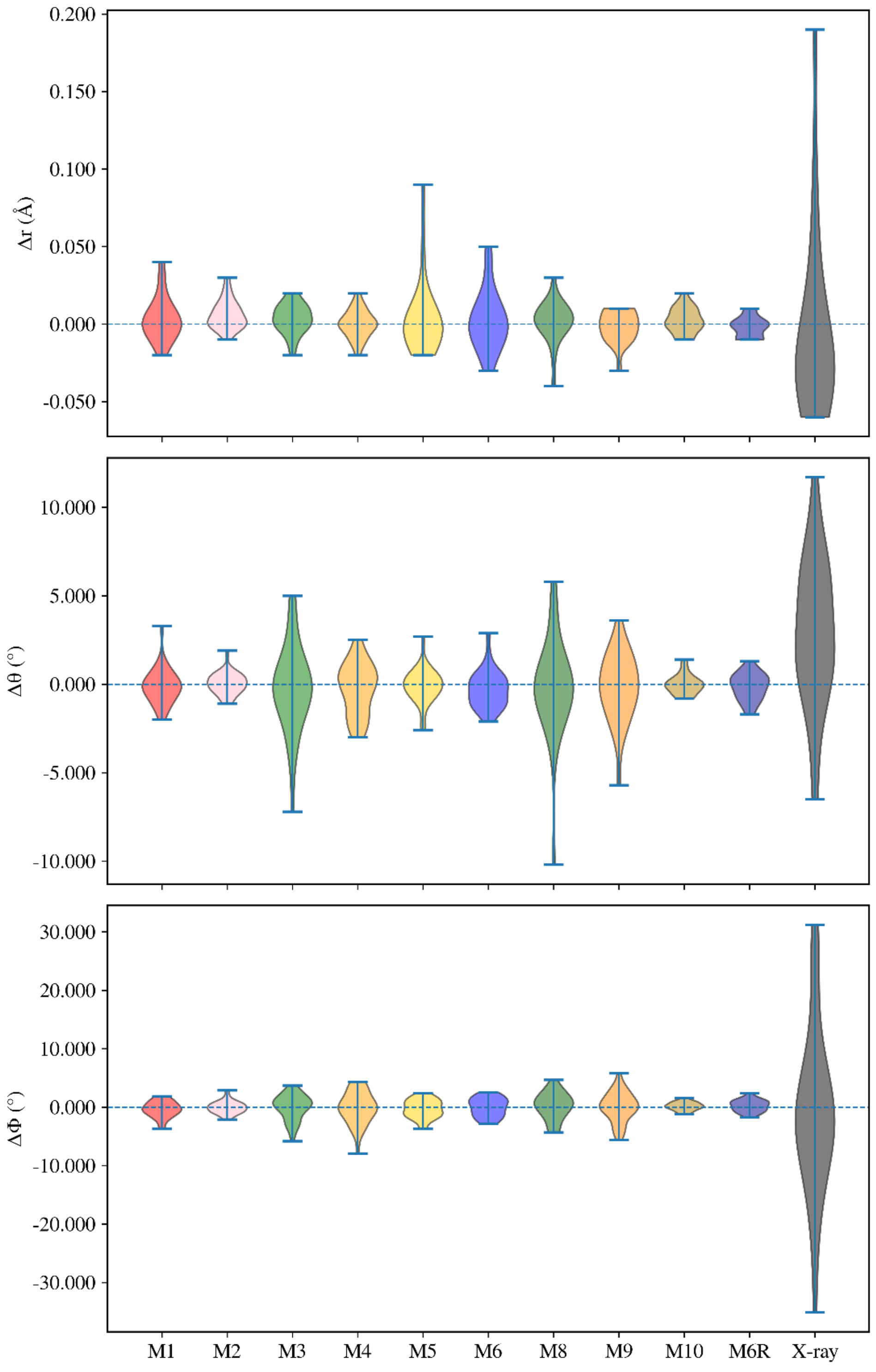

Supplementary Figure 33: Key coordinates of quantum refinement results of oseltamivir. Deviation in the refined bond distances (Δr, n = 20), angles (Δθ, n = 27) and dihedrals (Δϕ, n = 16) of oseltamivir (G39) in influenza neuraminidase from various quantum refinement schemes (**M1-M10**) and X-ray structure which are compared to those obtained from the most reliable **M7** scheme. The solid line represents the upper and lower values.

(iv) 6JX4 (Osimertinib in EGFR)

Protein preparations

Resolution: 2.53 Å

Ligand: YY3 (Osimertinib, Tagrisso); $C_{28}H_{33}N_7O_2$

Residue flipped: no

Addition of the missing atoms: 91 missing heavy atoms

Protonation states (pH = 8.0): Chain A: HID773, HID805, HIE835, HIE850, HID888, HIE893, HID988

Optimized region: YY3

High layer: YY3

Medium layer: Chain A: LEU718, GLY719, VAL726, ALA743, LYS745, MET790, GLN791, LEU792, MET793, PRO794, GLY796, CYS797, LEU844, LEU1001

$\omega_\alpha = 0.14880$

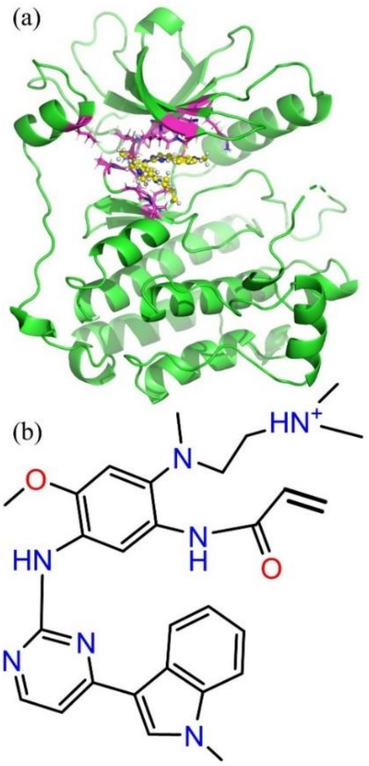

Supplementary Figure 34: Osimertinib in EGFR. (**a**) Crystal structure of EGFR with osimertinib (YY3). ONIOM layer by different color: yellow: high layer; red: medium layer; green: low layer. Ligand osimertinib is presented in stick and balls. (**b**) Structure of osimertinib.

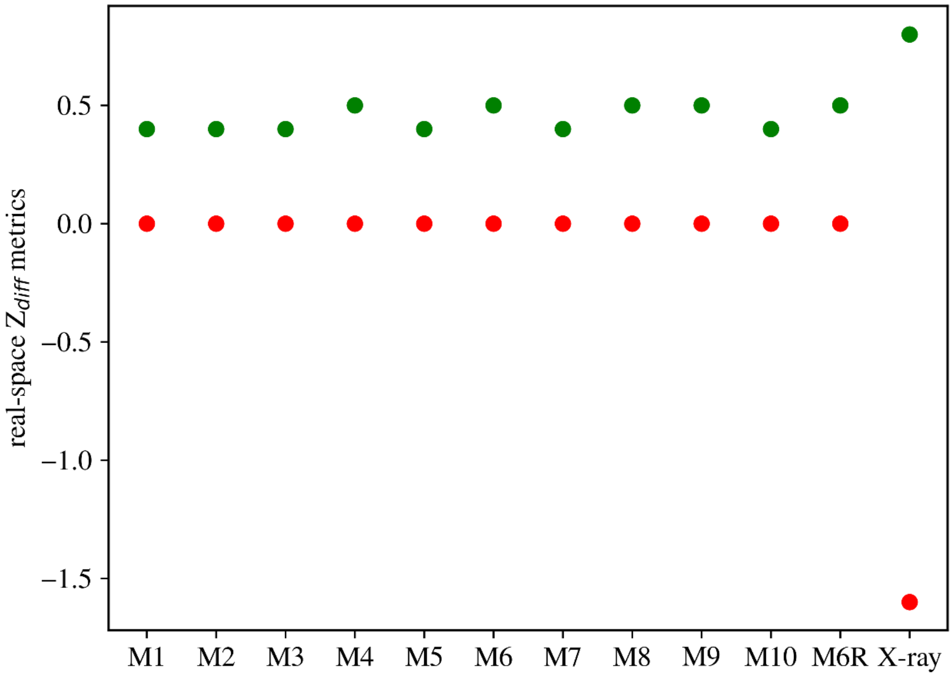

Supplementary Figure 35: Real-space Z-difference (RSZD) of osimertinib. RSZD+ (green) and RSZD- (red) scores of osimertinib (YY3) in EGFR from various quantum refinement schemes (**M1-M10**). Those results for X-ray were taken from the experimental structure without our further refinement.

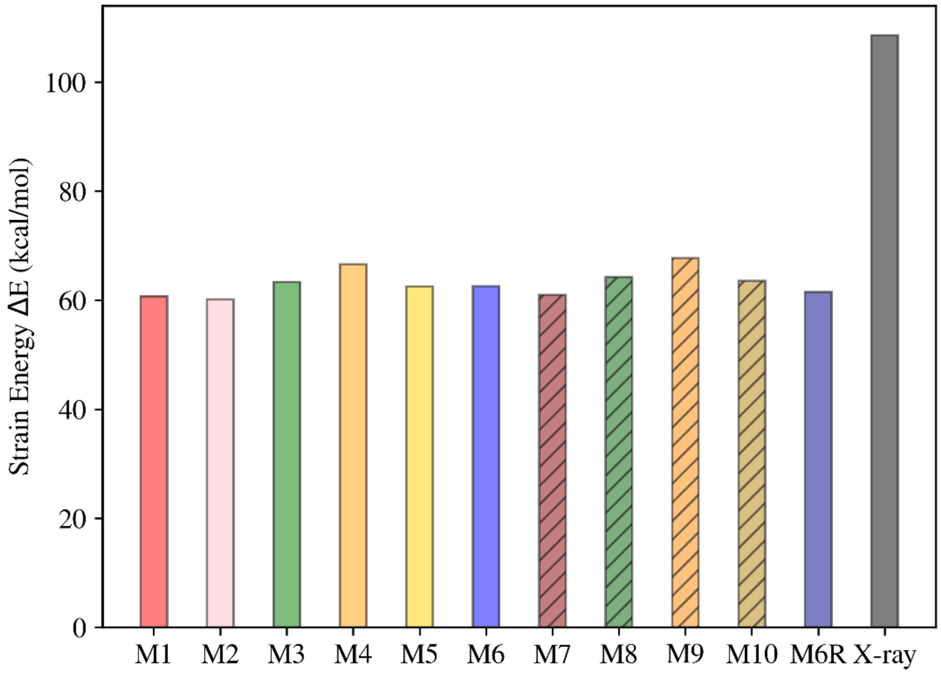

Supplementary Figure 36: Strain energy of osimertinib. Strain energy (ΔE, kcal·mol^{-1}) at ωB97X-D/6-31G(d) level for osimertinib (YY3) in EGFR determined by various quantum refinement schemes (**M1-M10**). Those results for X-ray were taken from the experimental structure without our further refinement.

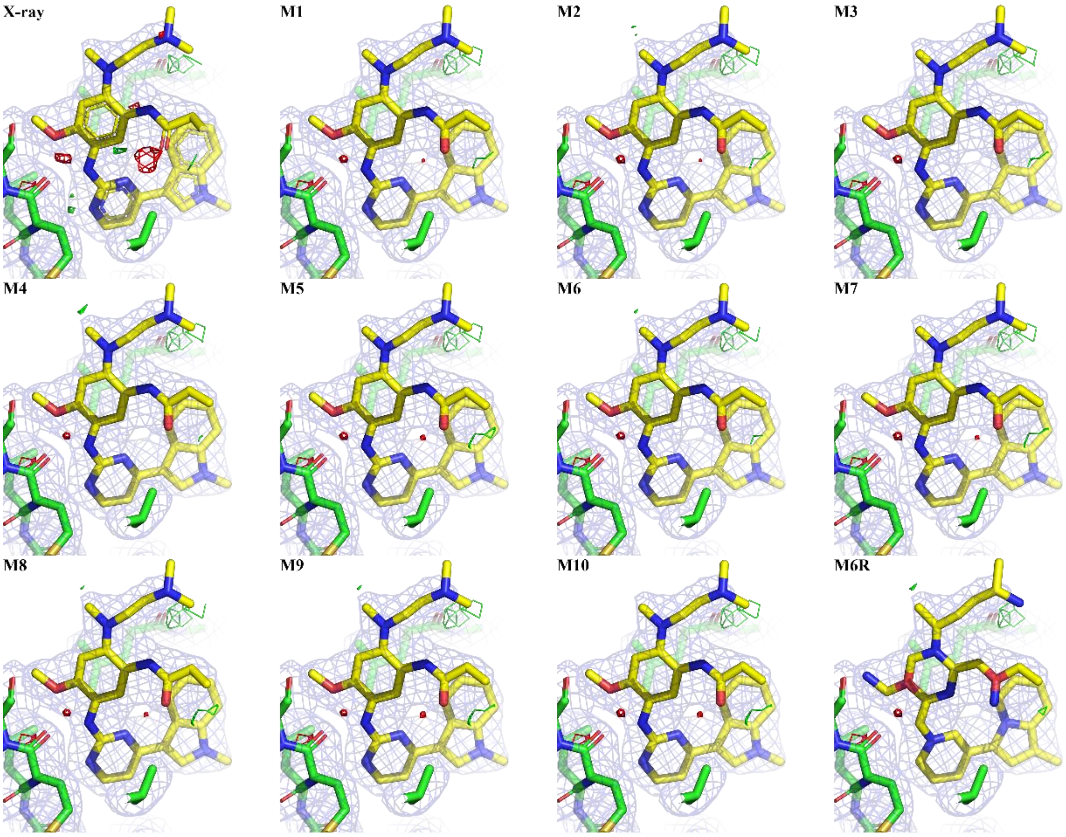

Supplementary Figure 37: Electron density maps of osimertinib. Structures for osimertinib (YY3) in EGFR from various quantum refinement schemes (**M1**-**M10**), including the electron density maps (2mFo-DFc maps, contoured at 1.0 σ (blue), mFo-DFc maps, contoured at +3.0 σ (green), and mFo-DFc maps, contoured at -3.0 σ (red)). Those results for X-ray were taken from the experimental structure without our further refinement.

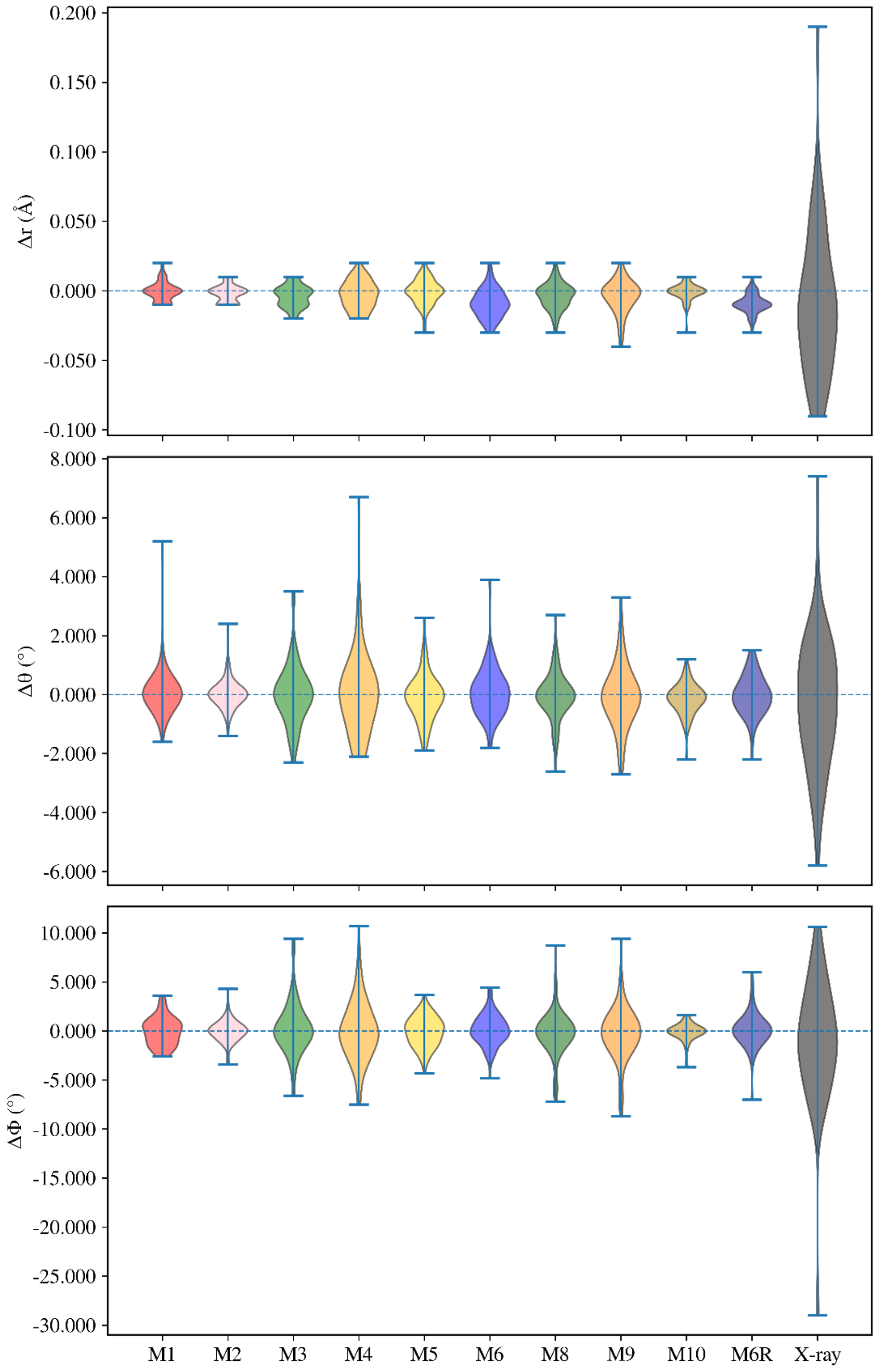

Supplementary Figure 38: Key coordinates of quantum refinement results of osimertinib. Deviation in the refined bond distances (Δr, n = 40), angles (Δθ, n = 56) and dihedrals (Δϕ, n = 25) of osimertinib (YY3) in EGFR from various quantum refinement schemes (**M1-M10**) and X-ray structure which are compared to those obtained from the most reliable **M7** scheme. The solid line represents the upper and lower values.

(v) 7RFW (Nirmatrelvir in SARS-CoV-2 main protease)

Protein preparations

Resolution: 1.73 Å

Ligand: 4WI (Nirmatrelvir, Paxlovid); $C_{23}H_{34}F_3N_5O_4$

Residues flipped: Chain A: ASN180

Protonation states (pH = 6.0): Chain A: HID41, HIP64, HID80, HIE163, HID164, HID172, HID246, GLH290

For the bonded form: CYM145;

For the nonbonded form: CYS145

Optimized region: 4WI + CYM(CYS)145

High layer: 4WI + CYM(CYS)145

Medium layer: Chain A: HID41, MET49, TYR54, PHE140, LEU141, ASN142, GLY143, SER144, CYM145, HIE163, HID164, MET165, GLU166, LEU167, PRO168, HID172, ASP187, ARG188, GLN189, THR190, WAT631

ω_α = 0.75739 (for bonded form) and ω_α = 0.78657 (for nonbonded form)

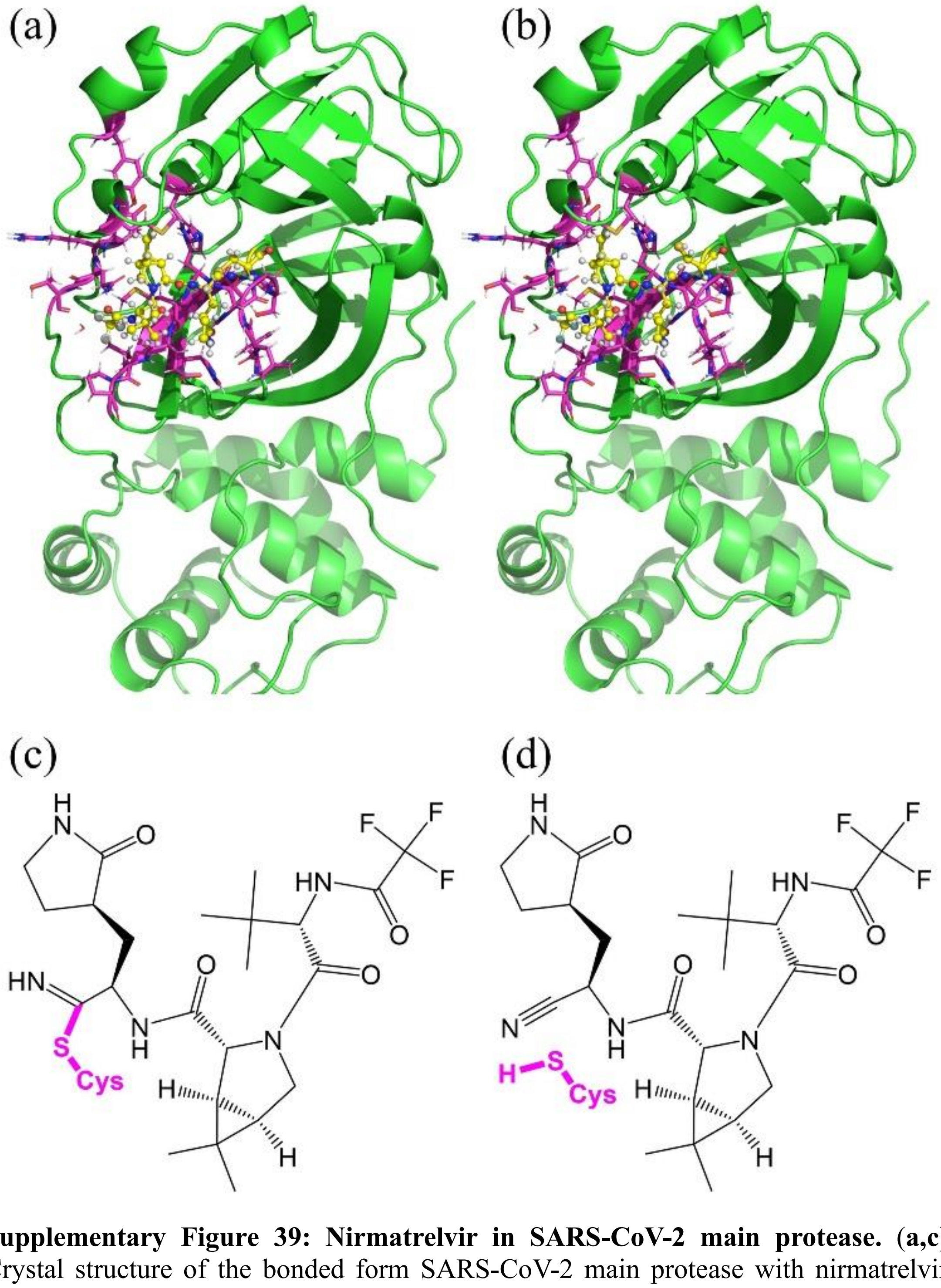

Supplementary Figure 39: Nirmatrelvir in SARS-CoV-2 main protease. (**a,c**) Crystal structure of the bonded form SARS-CoV-2 main protease with nirmatrelvir (4WI) and (**b, d**) its nonbonded form; ONIOM layer by different color: yellow: high layer; red: medium layer; green: low layer. Ligand nirmatrelvir and CYS are presented its stick and balls.

Quantum refined structural results:

Bonded form

Supplementary Table 38: Real-space Z-difference (RSZD) of the bonded form nirmatrelvir. RSZD scores of the residues around the bonded form nirmatrelvir (4WI) in SARS-CoV-2 main protease from various quantum refinement schemes (**M1**-**M10**). Those results for X-ray were taken from the experimental structure without our further refinement.

	M1	M2	M3	M4a	M5a	M6	M7	M8	M9a	M10a	M6R	X-ray
**HIS A 41**	0.3	0.3	0.3	0.3	0.3	0.3	0.3	0.3	0.3	0.3	0.3	0.5
**MET A 49**	5.5	5.5	5.6	5.5	5.6	5.5	5.5	5.6	5.6	5.6	5.5	5.1
**TYR A 54**	0.3	0.3	0.3	0.3	0.3	0.3	0.3	0.3	0.3	0.3	0.3	0.3
**PHE A 140**	0.1	0.1	0.1	0.1	0.1	0.1	0.1	0.1	0.1	0.1	0.1	0.1
**LEU A 141**	0.1	0.1	0.1	0.1	0.1	0.1	0.1	0.1	0.1	0.1	0.1	0.1
**ASN A 142**	1.5	1.5	1.5	1.5	1.5	1.5	1.5	1.4	1.4	1.5	1.5	1.3
**GLY A 143**	0.9	0.9	0.9	0.9	0.9	0.9	0.9	1.0	0.9	1.0	0.9	0.7
**SER A 144**	0.6	0.6	0.6	0.6	0.6	0.6	0.7	0.7	0.7	0.7	0.8	0.9
**CYS A 145**	6.1	5.6	6.1	6.0	6.1	6.3	5.7	5.7	5.7	5.7	5.8	6.5
**HIS A 163**	0.0	0.0	0.0	0.0	0.0	0.0	0.0	0.0	0.0	0.0	0.0	0.1
**HIS A 164**	1.5	1.5	1.5	1.5	1.5	1.5	1.5	1.5	1.6	1.6	1.5	1.7
**MET A 165**	3.3	3.4	3.3	3.3	3.3	3.3	3.4	3.4	3.4	3.5	3.4	3.5
**GLU A 166**	0.8	0.8	0.8	0.8	0.8	0.8	0.8	0.8	0.8	0.8	0.8	0.6
**LEU A 167**	1.6	1.6	1.6	1.6	1.6	1.6	1.6	1.6	1.6	1.6	1.6	1.5
**PRO A 168**	2.0	2.0	2.0	2.0	2.0	2.0	2.0	2.0	2.0	2.0	2.0	2.0
**HIS A 172**	0.0	0.0	0.0	0.0	0.0	0.0	0.0	0.0	0.0	0.0	0.0	0.0
**ASP A 187**	1.2	1.2	1.2	1.2	1.2	1.2	1.2	1.2	1.2	1.2	1.2	1.2
**ARG A 188**	0.3	0.3	0.2	0.3	0.2	0.3	0.3	0.2	0.2	0.3	0.3	0.4
**GLN A 189**	0.0	0.0	0.0	0.0	0.0	0.0	0.0	0.0	0.0	0.0	0.0	0.0
**THR A 190**	1.4	1.4	1.4	1.4	1.4	1.4	1.4	1.5	1.4	1.5	1.4	1.3
**4WI A 401**	0.2	0.3	0.2	0.3	0.3	0.3	0.1	0.2	0.2	0.2	0.3	1.1
**sum**	27.7	27.4	27.7	27.7	27.8	28.0	27.4	27.6	27.5	28.0	27.8	28.9

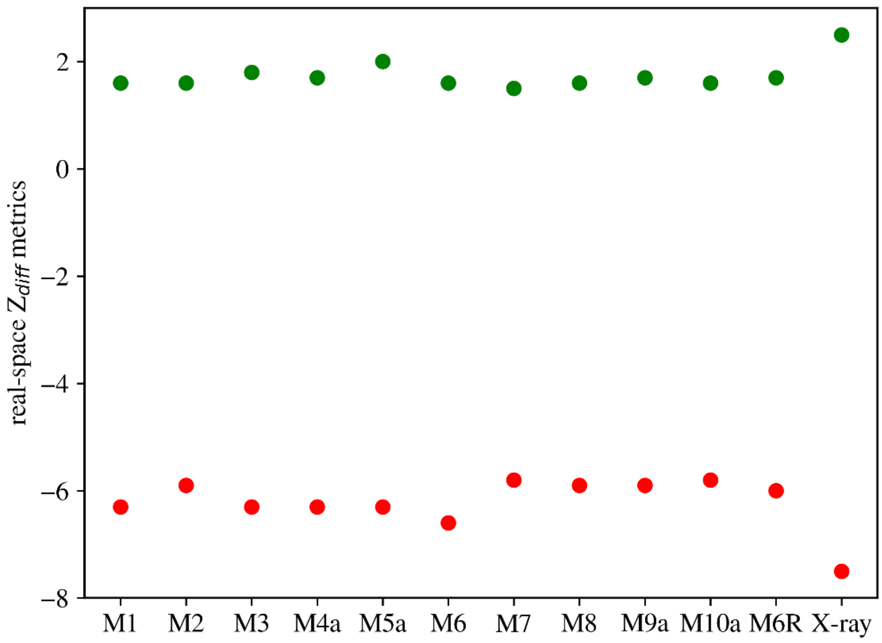

Supplementary Figure 40: Real-space Z-difference (RSZD) of the bonded form nirmatrelvir. RSZD+ (green) and RSZD- (red) scores of the bonded form nirmatrelvir (4WI) + CYS in SARS-CoV-2 main protease from various quantum refinement schemes (**M1-M10**). Those results for X-ray were taken from the experimental structure without our further refinement.

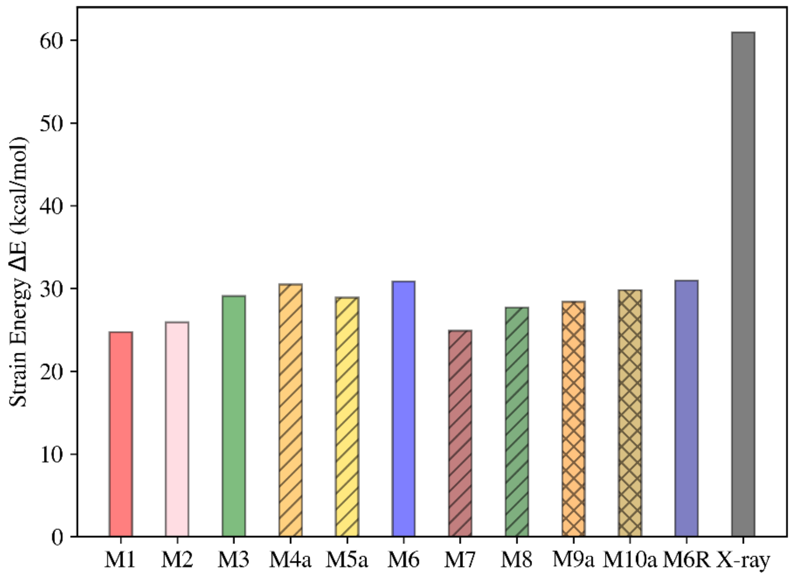

Supplementary Figure 41: Strain energy of the bonded form nirmatrelvir. Strain energy (ΔE, kcal·mol^{-1}) at ωB97X-D/6-31G(d) level for the bonded form nirmatrelvir (4WI) in SARS-CoV-2 main protease determined by various quantum refinement schemes (**M1-M10**). Those results for X-ray were taken from the experimental structure without our further refinement.

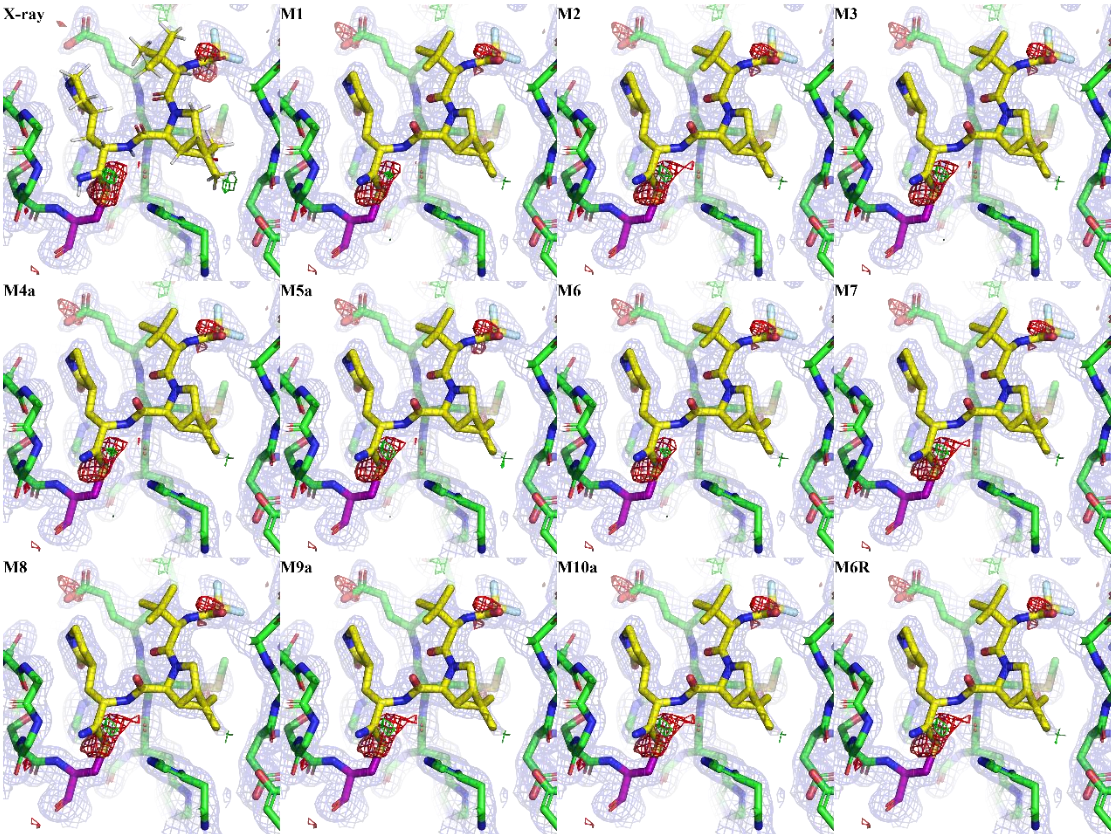

Supplementary Figure 42: Electron density maps of the bonded form nirmatrelvir. Structures for the bonded form nirmatrelvir (4WI) in SARS-CoV-2 main protease from various quantum refinement schemes (**M1**-**M10**), including the electron density maps (2mFo-DFc maps, contoured at 1.0 σ (blue), mFo-DFc maps, contoured at +3.0 σ (green), and mFo-DFc maps, contoured at -3.0 σ (red)). Those results for X-ray were taken from the experimental structure without our further refinement.

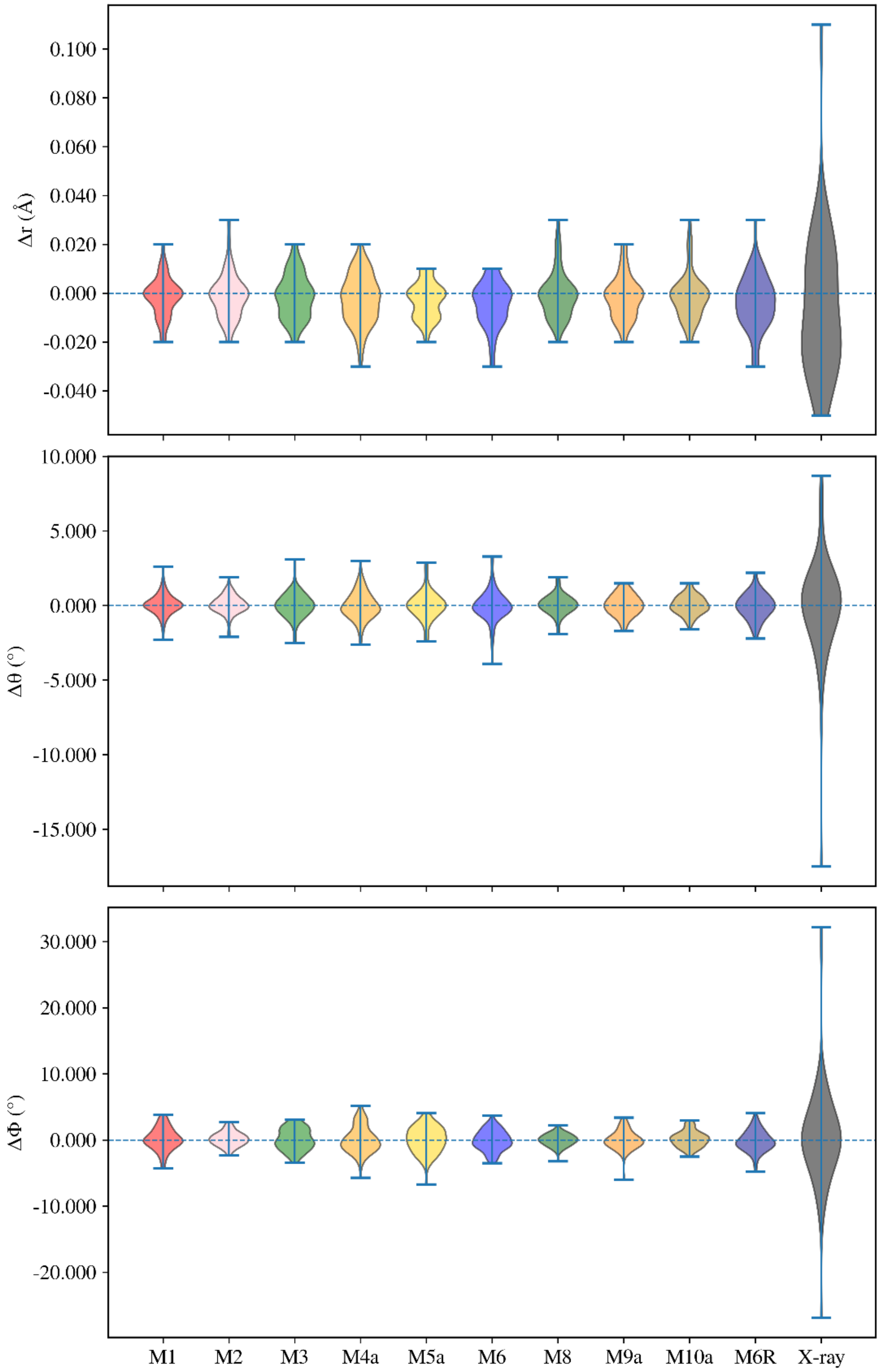

Supplementary Figure 43: Key coordinates of quantum refinement results of the bonded form nirmatrelvir. Deviation in the refined bond distances (Δr, n = 47), angles (Δθ, n = 69) and dihedrals (Δϕ, n = 54) of the bonded form nirmatrelvir (4WI) in SARS-CoV-2 main protease from various quantum refinement schemes (**M1-M10**) and X-ray structure which are compared to those obtained from the most reliable **M7** scheme. The solid line represents the upper and lower values.

Bonded + Nonbonded forms

Supplementary Table 39: Real-space Z-difference (RSZD) of the bonded and nonbonded forms nirmatrelvir (M1). RSZD scores of the residues around the nirmatrelvir (4WI) in SARS-CoV-2 main protease with different occupations (bonded: nonbonded forms) from quantum refinement using **M1**. Those results for X-ray were taken from the experimental structure without our further refinement.

	X-ray	1:0	9:1	8:2	7:3	6:4	5:5	4:6	3:7	2:8	1:9	0:1
**HIS A 41**	0.5	0.3	0.3	0.3	0.3	0.3	0.2	0.2	0.2	0.2	0.2	0.3
**MET A 49**	5.1	5.5	5.6	5.6	5.6	5.6	5.7	5.7	5.7	5.7	5.7	6.0
**TYR A 54**	0.3	0.3	0.3	0.3	0.3	0.3	0.3	0.2	0.2	0.2	0.2	0.3
**PHE A 140**	0.1	0.1	0.0	0.0	0.0	0.0	0.0	0.0	0.0	0.0	0.0	0.0
**LEU A 141**	0.1	0.1	0.1	0.1	0.1	0.1	0.1	0.1	0.1	0.1	0.2	0.1
**ASN A 142**	1.3	1.5	1.4	1.4	1.3	1.3	1.3	1.2	1.2	1.2	1.1	1.2
**GLY A 143**	0.7	0.9	0.8	0.8	0.7	0.7	0.8	0.7	0.7	0.8	0.8	0.8
**SER A 144**	0.9	0.6	0.6	0.5	0.5	0.5	0.4	0.4	0.4	0.3	0.3	0.3
**CYS A 145**	6.5	6.1	2.9	1.1	0.8	3.0	4.8	6.6	8.4	10.3	12.0	4.5
**HIS A 163**	0.1	0.0	0.0	0.0	0.0	0.0	0.0	0.0	0.0	0.0	0.0	0.0
**HIS A 164**	1.7	1.5	1.5	1.6	1.6	1.6	1.6	1.5	1.5	1.5	1.5	1.6
**MET A 165**	3.5	3.3	3.3	3.3	3.3	3.3	3.3	3.3	3.2	3.2	3.2	3.2
**GLU A 166**	0.6	0.8	0.9	0.8	0.8	0.8	0.9	0.8	0.8	0.8	0.8	0.8
**LEU A 167**	1.5	1.6	1.6	1.6	1.6	1.6	1.7	1.7	1.7	1.7	1.7	1.7
**PRO A 168**	2.0	2.0	2.0	2.0	2.0	2.0	2.0	2.0	2.0	2.0	2.0	2.0
**HIS A 172**	0.0	0.0	0.0	0.0	0.0	0.0	0.0	0.0	0.0	0.0	0.0	0.0
**ASP A 187**	1.2	1.2	1.2	1.3	1.3	1.3	1.3	1.3	1.4	1.4	1.4	1.4
**ARG A 188**	0.4	0.3	0.3	0.3	0.3	0.3	0.3	0.3	0.3	0.3	0.3	0.3
**GLN A 189**	0.0	0.0	0.0	0.1	0.1	0.1	0.1	0.1	0.2	0.2	0.2	0.2
**THR A 190**	1.3	1.4	1.4	1.4	1.5	1.5	1.5	1.5	1.5	1.5	1.5	1.4
**4WI A 401**	1.1	0.2	0.2	0.2	0.1	0.1	0.0	0.0	0.0	0.1	0.6	2.1
**sum**	28.9	27.7	24.4	22.7	22.2	24.4	26.3	27.6	29.5	31.5	33.7	28.2

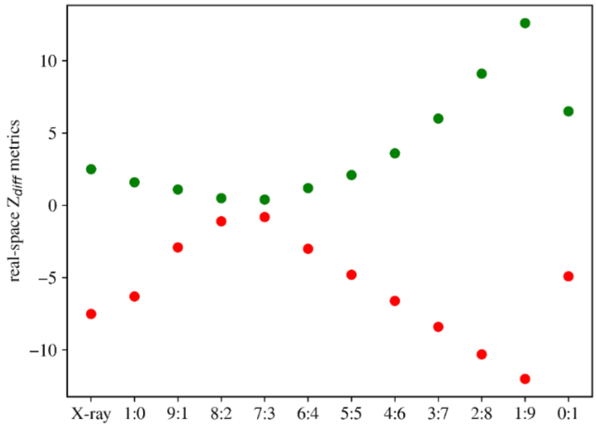

Supplementary Figure 44: Real-space Z-difference (RSZD) of the bonded and nonbonded forms nirmatrelvir (M1). RSZD+ (green) and RSZD- (red) scores of nirmatrelvir (4WI) + CYS in SARS-CoV-2 main protease with different occupations (bonded: nonbonded forms) from quantum refinement using **M1**. Those results for X-ray were taken from the experimental structure without our further refinement.

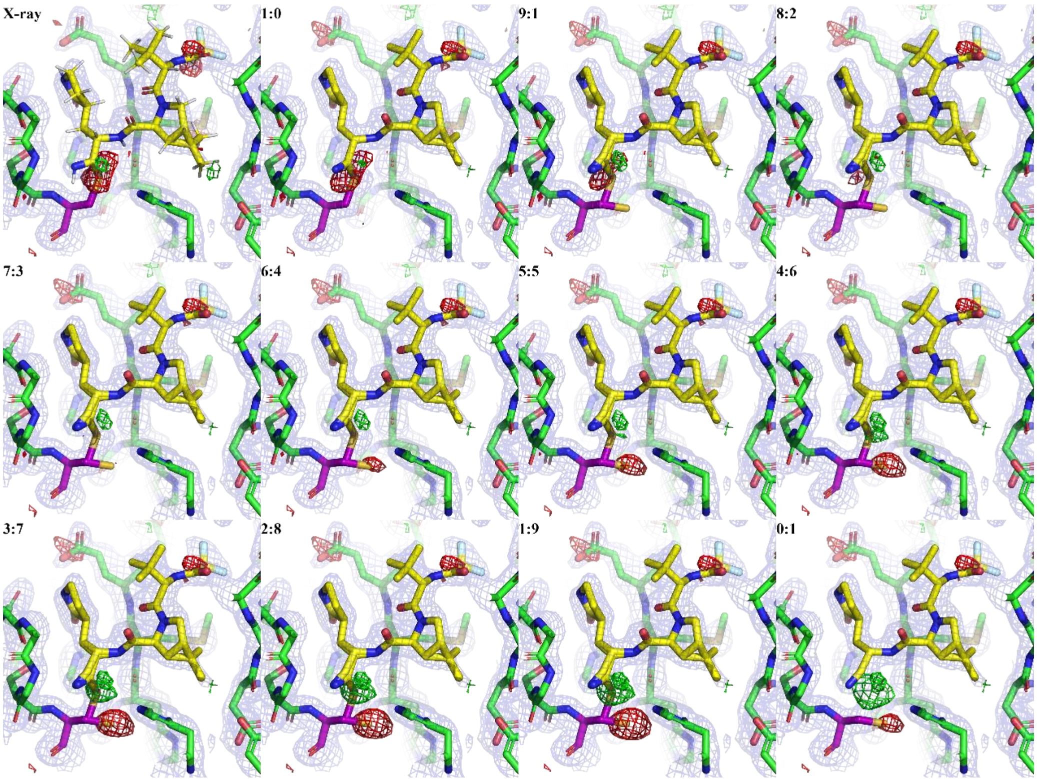

Supplementary Figure 45: Electron density maps of the bonded and nonbonded forms nirmatrelvir (M1). Structures for nirmatrelvir (4WI) in SARS-CoV-2 main protease with different occupations (bonded: nonbonded forms) from quantum refinement using **M1**, including the electron density maps (2mFo-DFc maps, contoured at 1.0 σ (blue), mFo-DFc maps, contoured at +3.0 σ (green), and mFo-DFc maps, contoured at -3.0 σ (red)). Those results for X-ray were taken from the experimental structure without our further refinement.

Supplementary Table 40: Real-space Z-difference (RSZD) of the bonded and nonbonded forms nirmatrelvir (M7). RSZD scores of the residues around the nirmatrelvir (4WI) in SARS-CoV-2 main protease with different occupations (bonded: nonbonded forms) from quantum refinement using **M7**. Those results for X-ray were taken from the experimental structure without our further refinement.

	X-ray	1:0	9:1	8:2	7:3	6:4	5:5	4:6	3:7	2:8	1:9	0:1
**HIS A 41**	0.5	0.3	0.3	0.3	0.3	0.3	0.3	0.3	0.3	0.3	0.3	0.4
**MET A 49**	5.1	5.5	5.6	5.6	5.7	5.7	5.7	5.8	5.8	5.8	5.8	6
**TYR A 54**	0.3	0.3	0.3	0.3	0.3	0.3	0.3	0.3	0.3	0.3	0.3	0.3
**PHE A 140**	0.1	0.1	0	0	0	0	0	0	0	0	0	0
**LEU A 141**	0.1	0.1	0.1	0.1	0.1	0.1	0.1	0.1	0.1	0.1	0.1	0.2
**ASN A 142**	1.3	1.5	1.4	1.4	1.3	1.2	1.2	1.1	1.1	1	1	1.2
**GLY A 143**	0.7	0.9	0.9	0.8	0.8	0.7	0.7	0.7	0.8	0.8	0.8	0.9
**SER A 144**	0.9	0.7	0.6	0.6	0.6	0.5	0.5	0.5	0.4	0.4	0.3	0.3
**CYS A 145**	6.5	5.7	2.4	0.6	0.2	1.4	3.8	6.1	8.3	10.5	12.6	5.4
**HIS A 163**	0.1	0	0	0	0	0	0	0	0	0	0	0
**HIS A 164**	1.7	1.5	1.6	1.6	1.6	1.5	1.5	1.5	1.5	1.5	1.5	1.6
**MET A 165**	3.5	3.4	3.3	3.3	3.3	3.3	3.2	3.1	3.1	3.1	3	3.1
**GLU A 166**	0.6	0.8	0.8	0.8	0.8	0.8	0.8	0.8	0.7	0.7	0.7	0.8
**LEU A 167**	1.5	1.6	1.6	1.6	1.6	1.7	1.7	1.7	1.7	1.7	1.7	1.7
**PRO A 168**	2	2	2	2	2	2	1.9	1.9	1.9	1.9	1.9	1.9
**HIS A 172**	0	0	0	0	0	0	0	0	0	0	0	0
**ASP A 187**	1.2	1.2	1.3	1.3	1.4	1.4	1.5	1.5	1.5	1.6	1.6	1.5
**ARG A 188**	0.4	0.3	0.3	0.3	0.3	0.3	0.3	0.3	0.3	0.3	0.3	0.3
**GLN A 189**	0	0	0.1	0.1	0.1	0.1	0.2	0.2	0.2	0.3	0.3	0.2
**THR A 190**	1.3	1.4	1.5	1.5	1.5	1.5	1.5	1.5	1.4	1.4	1.4	1.4
**4WI A 401**	1.1	0.1	0.4	0.3	0.2	0.1	0.1	0.1	0.1	0.2	0.6	2.1
**sum**	28.9	27.4	24.5	22.5	22.1	22.9	25.3	27.5	29.5	31.9	34.2	29.3

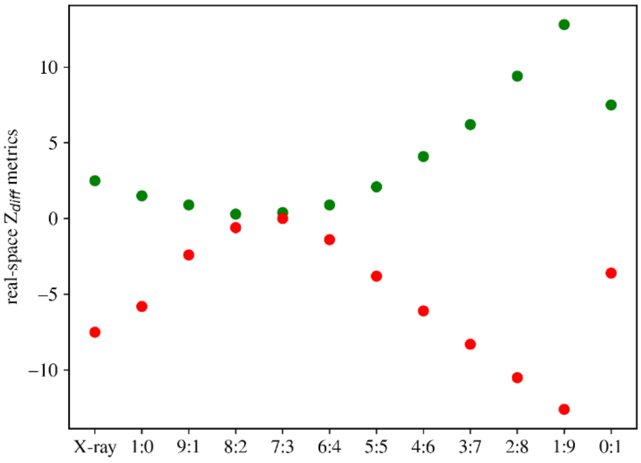

Supplementary Figure 46: Real-space Z-difference (RSZD) of the bonded and nonbonded forms nirmatrelvir (M7). RSZD+ (green) and RSZD- (red) scores of nirmatrelvir (4WI) + CYS in SARS-CoV-2 main protease with different occupations (bonded: nonbonded forms) from quantum refinement using **M7**. Those results for X-ray were taken from the experimental structure without our further refinement.

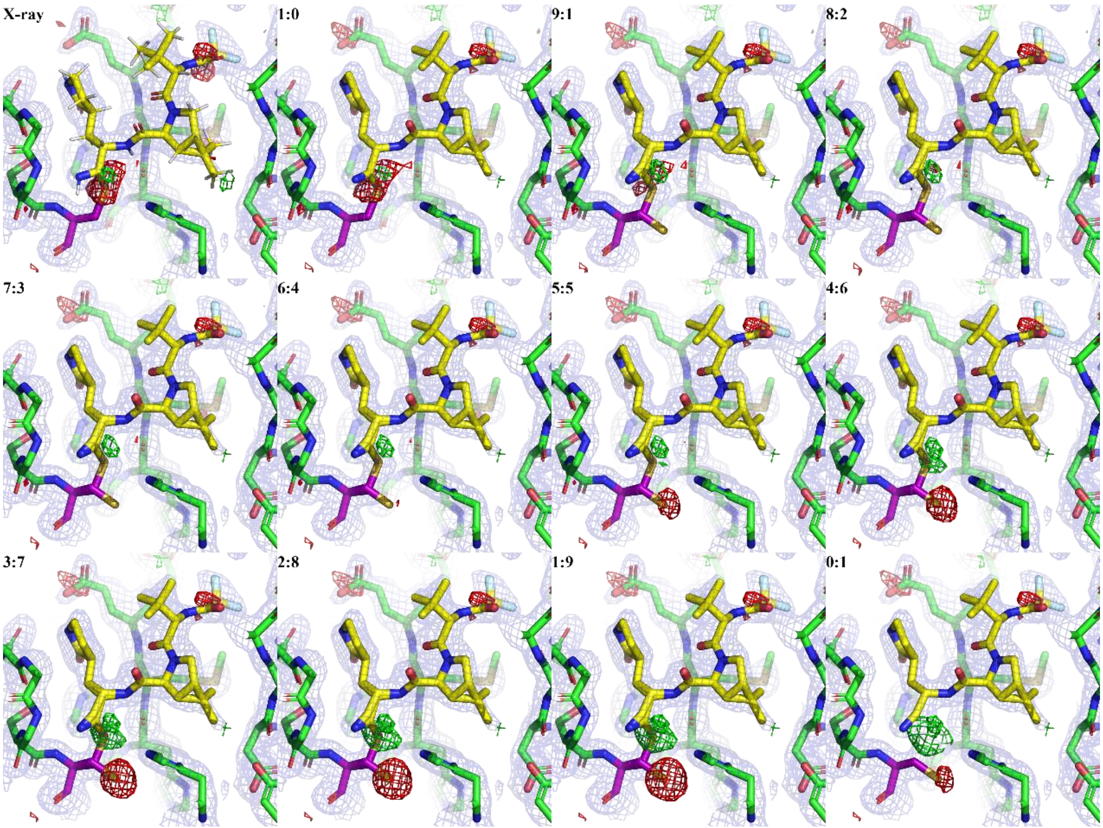

Supplementary Figure 47: Electron density maps of the bonded and nonbonded forms nirmatrelvir (M7). Structures for nirmatrelvir (4WI) in SARS-CoV-2 main protease with different occupations (bonded: nonbonded forms) from quantum refinement using **M7**, including the electron density maps (2mFo-DFc maps, contoured at 1.0 σ (blue), mFo-DFc maps, contoured at +3.0 σ (green), and mFo-DFc maps, contoured at -3.0 σ (red)). Those results for X-ray were taken from the experimental structure without our further refinement.

Supplementary Table 41: Real-space Z-difference (RSZD) of the bonded and nonbonded forms nirmatrelvir (M9). RSZD scores of the residues around the nirmatrelvir (4WI) in SARS-CoV-2 main protease with different occupations (bonded: nonbonded forms) from quantum refinement using **M9**. Those results for X-ray were taken from the experimental structure without our further refinement.

	X-ray	1:0	9:1	8:2	7:3	6:4	5:5	4:6	3:7	2:8	1:9	0:1
**HIS A 41**	0.5	0.3	0.3	0.3	0.3	0.3	0.2	0.2	0.2	0.2	0.2	0.3
**MET A 49**	5.1	5.6	5.5	5.5	5.5	5.6	5.6	5.7	5.7	5.7	5.7	6.0
**TYR A 54**	0.3	0.3	0.3	0.3	0.3	0.3	0.2	0.2	0.2	0.2	0.2	0.2
**PHE A 140**	0.1	0.1	0.0	0.0	0.0	0.0	0.0	0.0	0.0	0.0	0.0	0.0
**LEU A 141**	0.1	0.1	0.1	0.1	0.1	0.1	0.1	0.1	0.1	0.1	0.2	0.1
**ASN A 142**	1.3	1.5	1.4	1.4	1.4	1.3	1.3	1.3	1.2	1.2	1.2	1.2
**GLY A 143**	0.7	0.9	0.9	0.8	0.8	0.7	0.7	0.7	0.7	0.8	0.8	0.8
**SER A 144**	0.9	0.6	0.6	0.5	0.5	0.5	0.4	0.4	0.4	0.3	0.3	0.3
**CYS A 145**	6.5	6.1	2.9	1.0	0.9	3.0	4.9	6.6	8.5	10.4	12.0	4.5
**HIS A 163**	0.1	0.0	0.0	0.0	0.0	0.0	0.0	0.0	0.0	0.0	0.0	0.0
**HIS A 164**	1.7	1.5	1.5	1.5	1.5	1.5	1.5	1.5	1.5	1.5	1.5	1.6
**MET A 165**	3.5	3.3	3.3	3.3	3.3	3.2	3.3	3.2	3.2	3.1	3.1	3.2
**GLU A 166**	0.6	0.8	0.9	0.9	0.9	0.9	0.9	0.8	0.8	0.8	0.8	0.8
**LEU A 167**	1.5	1.6	1.6	1.6	1.6	1.6	1.6	1.6	1.7	1.7	1.7	1.7
**PRO A 168**	2.0	2.0	2.0	2.0	2.0	2.0	2.0	2.0	2.0	2.0	2.0	2.0
**HIS A 172**	0.0	0.0	0.0	0.0	0.0	0.0	0.0	0.0	0.0	0.0	0.0	0.0
**ASP A 187**	1.2	1.2	1.2	1.2	1.3	1.3	1.3	1.3	1.4	1.4	1.4	1.4
**ARG A 188**	0.4	0.2	0.2	0.3	0.3	0.3	0.3	0.3	0.3	0.3	0.3	0.3
**GLN A 189**	0.0	0.0	0.0	0.1	0.1	0.1	0.1	0.1	0.1	0.2	0.2	0.2
**THR A 190**	1.3	1.4	1.4	1.4	1.5	1.5	1.5	1.5	1.4	1.4	1.4	1.4
**4WI A 401**	1.1	0.3	0.6	0.4	0.3	0.2	0.1	0.1	0.1	0.3	0.8	2.1
**sum**	28.9	27.8	24.7	22.6	22.6	24.4	26.0	27.6	29.5	31.6	33.8	28.1

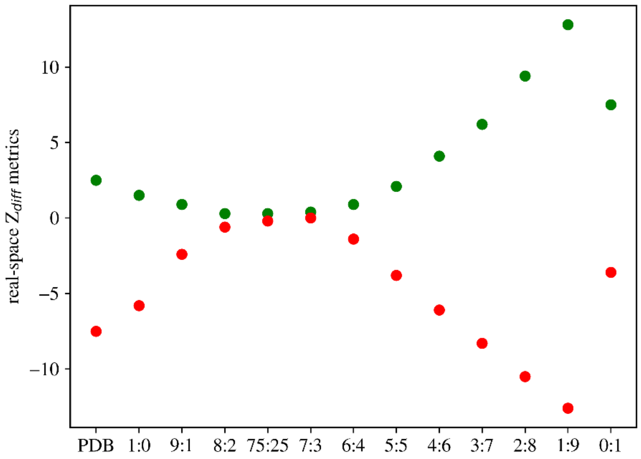

Supplementary Figure 48: Real-space Z-difference (RSZD) of the bonded and nonbonded forms nirmatrelvir (M9). RSZD+ (green) and RSZD- (red) scores of nirmatrelvir (4WI) + CYS in SARS-CoV-2 main protease with different occupations (bonded: nonbonded forms) from quantum refinement using **M9**. Those results for X-ray were taken from the experimental structure without our further refinement.

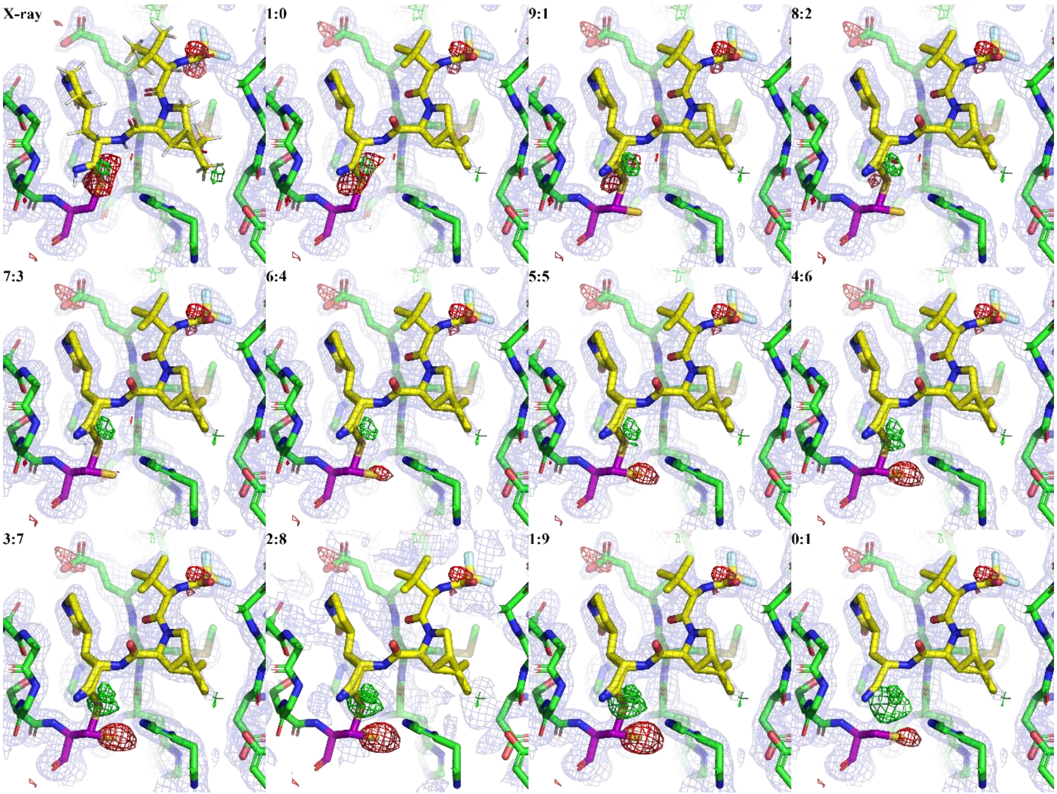

Supplementary Figure 49: Electron density maps of the bonded and nonbonded forms nirmatrelvir (M9). Structures for nirmatrelvir (4WI) in SARS-CoV-2 main protease with different occupations (bonded: nonbonded forms) from quantum refinement using **M9**, including the electron density maps (2mFo-DFc maps, contoured at 1.0 σ (blue), mFo-DFc maps, contoured at +3.0 σ (green), and mFo-DFc maps, contoured at -3.0 σ (red)). Those results for X-ray were taken from the experimental structure without our further refinement.

Bonded: Nonbonded = 7:3

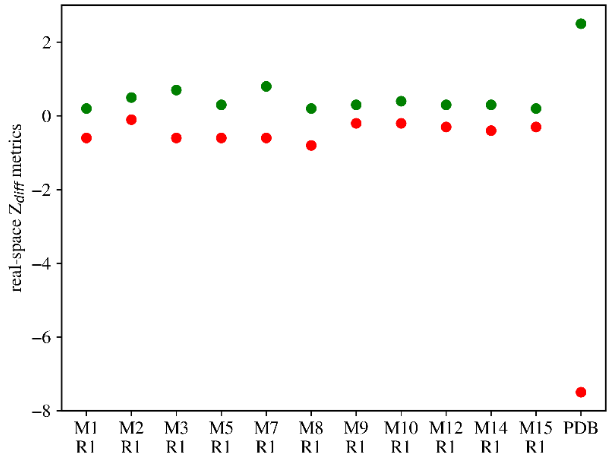

Supplementary Figure 50: Real-space Z-difference (RSZD) of the bonded and nonbonded forms nirmatrelvir (7:3). RSZD+ (green) and RSZD- (red) scores of nirmatrelvir (4WI) + CYS in SARS-CoV-2 main protease with 7:3 occupations (bonded: nonbonded forms) from various quantum refinement schemes (**M1-M10**). Those results for X-ray were taken from the experimental structure without our further refinement.

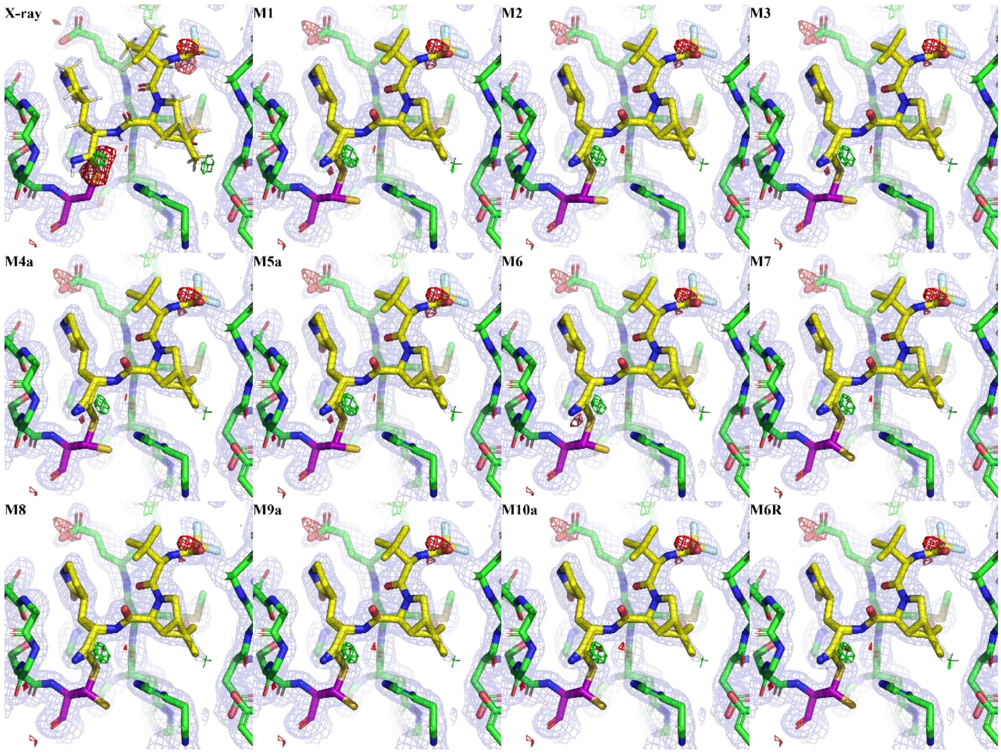

Supplementary Figure 51: Real-space Z-difference (RSZD) of the bonded and nonbonded forms nirmatrelvir (7:3). Structures for nirmatrelvir (4WI) in SARS-CoV-2 main protease with 7:3 occupations (bonded: nonbonded forms) from various quantum refinement schemes (**M1-M10**) and X-ray, including the electron density maps (2mFo-DFc maps, contoured at 1.0 σ (blue), mFo-DFc maps, contoured at +3.0 σ (green), and mFo-DFc maps, contoured at -3.0 σ (red)). Those results for X-ray were taken from the experimental structure without our further refinement.

Supplementary Table 42: Wild-type SARS-CoV-2 main protease with nirmatrelvir. Reported available structures of wild-type SARS-CoV-2 main protease with nirmatrelvir (link to CYS). Values in parentheses of 7RFW are the results of our quantum refinement for the bonded form by **M7**.

PDB ID	Resolution (Å)	C_{drug}-S_{cys} Distance (Å)	RSZD	
			Nirmatrelvir	CYS
7RFW[a]	1.73	1.922(1.812)	1.3(0.3)	6.5(0.2)
7RFS[a]	1.91	1.804	0.0	0.7
7SI9[b]	2.00	1.805	2.1	2.5
7TE0[c]	2.00	1.810	1.3	2.2
7VH8[d]	1.59	1.814/4.251	2.3	2.2
7VLO[e]	2.02	A: 1.766	2.6	0.2
		B: 1.765	0.9	0.5
7VLP[f]	1.50	A:1.765	1.5	1.2
		B: 1.763	2.6	0.8
7VLQ[f]	1.94	A: 1.766	0.2	0.3
		B: 1.767	0.0	0.5
8DZ2[g]	2.13	A: 1.808	1.0	1.8
		B: 1.771	0.4	0.6

a. (2021) Science 374: 1586-1593; b. (2022) Nat Commun 13: 2268. c. (2022) J Med Chem 65: 8686-8698. d. (2022) Protein Cell 13: 689-693. (2023). e. (2022) J Virol 96: e0201321-e0201321. f. (2022) J Virol 96: e0201321-e0201321. g. J Biol Chem 299: 103004-103004

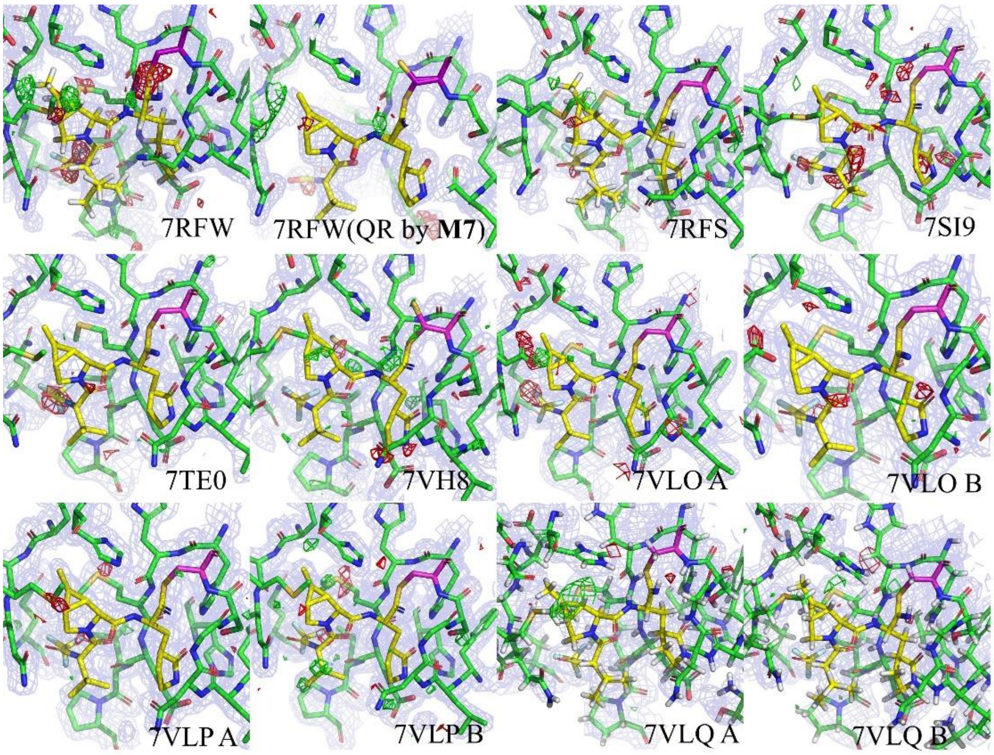

Supplementary Figure 52: Electron density maps of the available wild-type SARS-CoV-2 main protease with nirmatrelvir. Available crystal structures for nirmatrelvir (4WI) in wild-type SARS-CoV-2 main protease including the electron density maps (2mFo-DFc maps, contoured at 1.0 σ (blue), mFo-DFc maps, contoured at +3.0 σ (green), and mFo-DFc maps, contoured at -3.0 σ (red)).

(vi) 2W26 (Rivaroxaban in Factor Xa)

Protein preparations

Resolution: 2.08 Å

Ligand: RIV (Rivaroxaban, Xarelto); $C_{19}H_{18}ClN_3O_5S$

Residue flipped: Chain B: HIS13

Addition of the missing atoms: 34 missing heavy atoms

Protonation states (pH = 7.4): Chain A: HID57, HID83, HID91, HID145, HID199; Chain B: HIP13

CYX-CYX: A CYX22-A CYX27, A CYX42-A CYX58, A CYX122-B CYX 44, A CYX 168-A CYX 182, A CYX 191-A CYX 220, B CYX 1-B CYX 12, B CYX 8-B CYX 21, B CYX 23-B CYX 36

Optimized region: RIV

High layer: RIV

Medium layer: Chain A: LYS96, GLU97, THR98, TYR99, PHE174, ASP189, ALA190, CYX191, GLN192, TRP215, GLY216, GLU217, GLY219, CYX220, GLY226, ILE227, WAT2059, WAT2065

ω_α = 0.14880

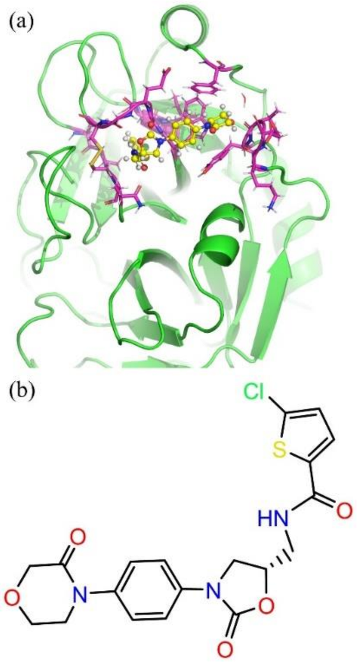

Supplementary Figure 53: Rivaroxaban in Factor Xa. (**a**) Crystal structure of Factor Xa with rivaroxaban (RIV). ONIOM layer by different color: yellow: high layer; red: medium layer; green: low layer. Ligand rivaroxaban is presented in stick and balls. (**b**) Structure of rivaroxaban.

Quantum refined structural results:

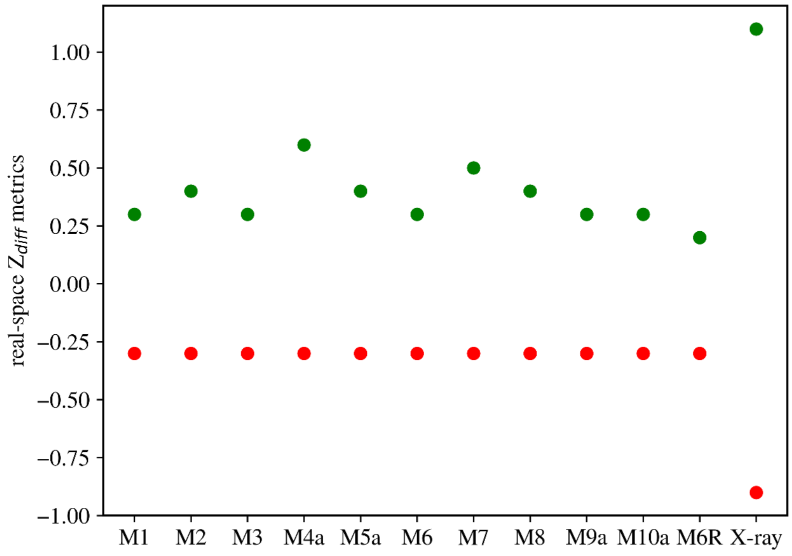

Supplementary Figure 54: Real-space Z-difference (RSZD) of rivaroxaban. RSZD+ (green) and RSZD- (red) scores of rivaroxaban (RIV) in Factor Xa from various quantum refinement schemes (**M1-M10**). Those results for X-ray were taken from the experimental structure without our further refinement.

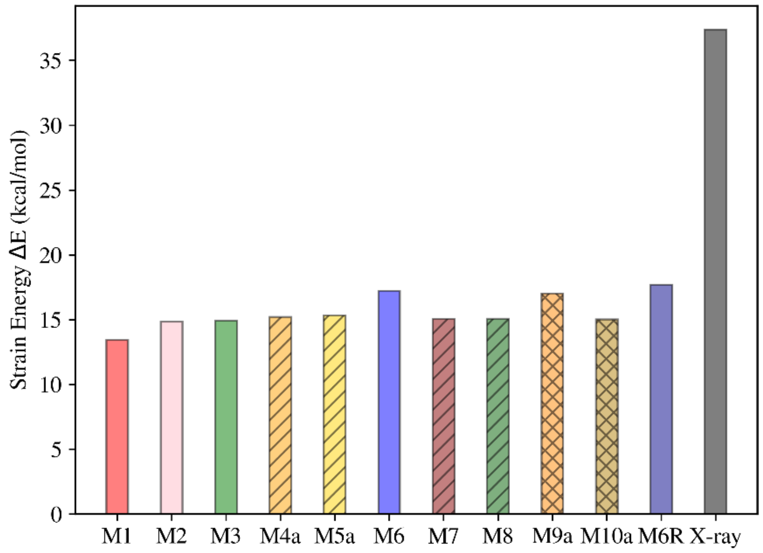

Supplementary Figure 55: Strain energy of rivaroxaban. Strain energy (ΔE, kcal·mol^{-1}) at ωB97X-D/6-31G(d) level for rivaroxaban (RIV) in Factor Xa determined by various quantum refinement schemes (**M1-M10**). Those results for X-ray were taken from the experimental structure without our further refinement.

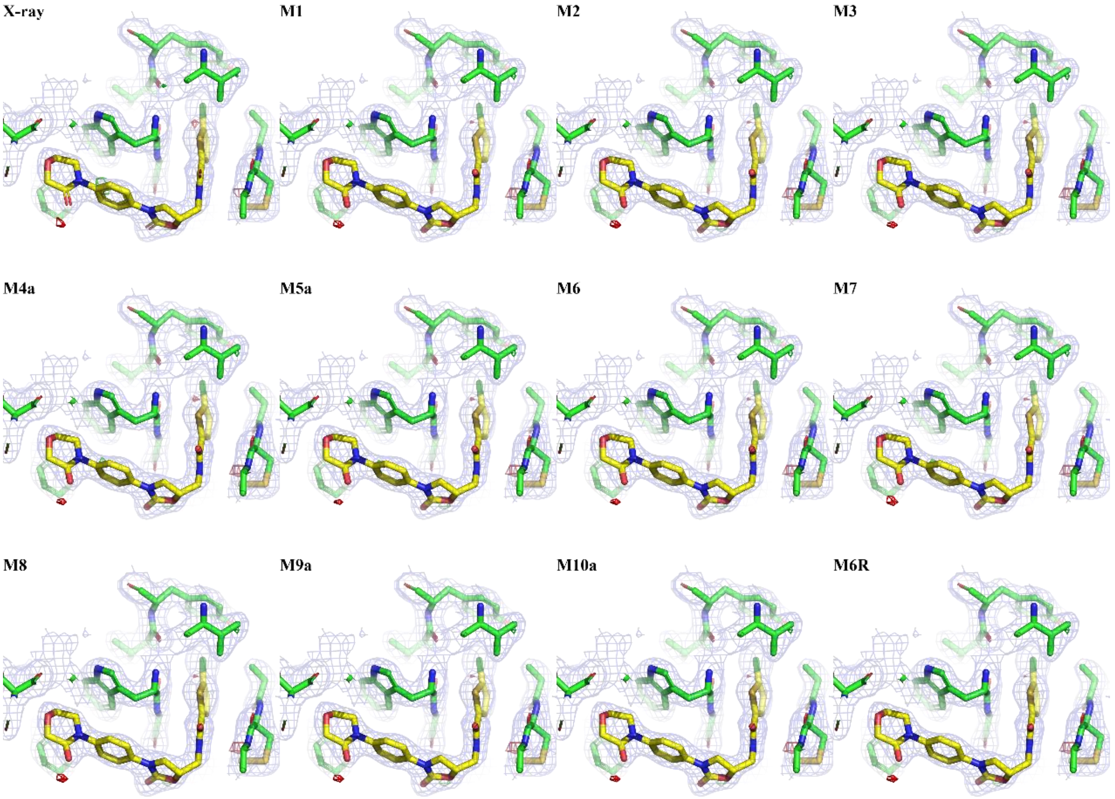

Supplementary Figure 56: Electron density maps of rivaroxaban. Structures for rivaroxaban (RIV) in Factor Xa from various quantum refinement schemes (**M1**-**M10**), including the electron density maps (2mFo-DFc maps, contoured at 1.0 σ (blue), mFo-DFc maps, contoured at +3.0 σ (green), and mFo-DFc maps, contoured at -3.0 σ (red)). Those results for X-ray were taken from the experimental structure without our further refinement.

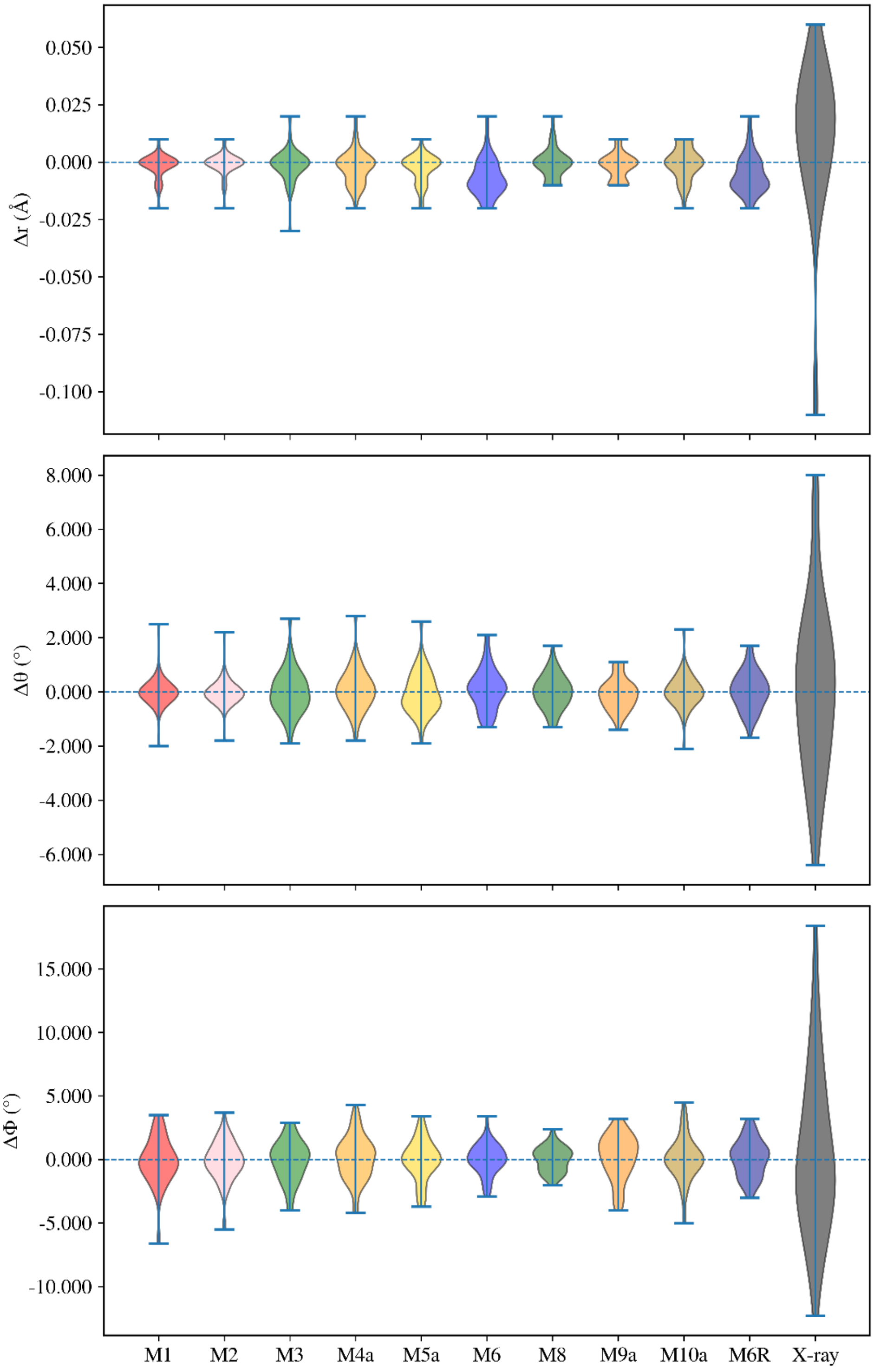

Supplementary Figure 57: Key coordinates of quantum refinement results of rivaroxaban. Deviation in the refined bond distances (Δr, n = 32), angles (Δθ, n = 45) and dihedrals (Δϕ, n = 17) of rivaroxaban (RIV) in Factor Xa from various quantum refinement schemes (**M1-M10**) and X-ray structure which are compared to those obtained from the most reliable **M7** scheme. The solid line represents the upper and lower values.

(vii) 5KR1 (Darunavir in HIV-1)

Protein preparations

Resolution: 2.08 Å

Ligand: 017 (Darunavir, Prezista); $C_{27}H_{37}N_3O_7S$

Residue flipped: Chain A: GLN2; Chain B: ASN98

Protonation states (pH = 5.0): Chain A: HIP69; Chain B: ASH30, GLH34, HIP69

Optimized region: 017

High layer: 017

Medium layer:

Chain A: ARG8, GLU21, LEU23, ASN25, GLY27, ALA28, ASP30, ILE47, GLY48, GLY49, ILE50, PRO81, VAL82, ILE84, WAT114, WAT144, WAT154, WAT162, WAT173, WAT177, WAT193

Chain B: ASN25, GLY27, ALA28, ASP29, ASH30, VAL32, ILE47, GLY48, ILE50, VAL82, ILE84, WAT219, WAT236, WAT257

ω_α = 0.30129

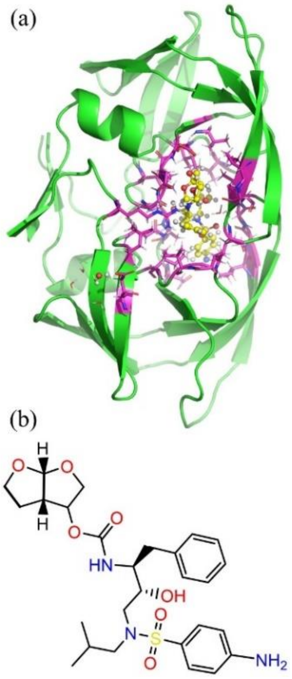

Supplementary Figure 58: Darunavir in HIV-1. (**a**) Crystal structure of HIV-1 with darunavir (017). ONIOM layer by different color: yellow: high layer; red: medium layer; green: low layer. Ligand darunavir is presented in stick and balls. (**b**) Structure of darunavir.

Quantum refined structural results:

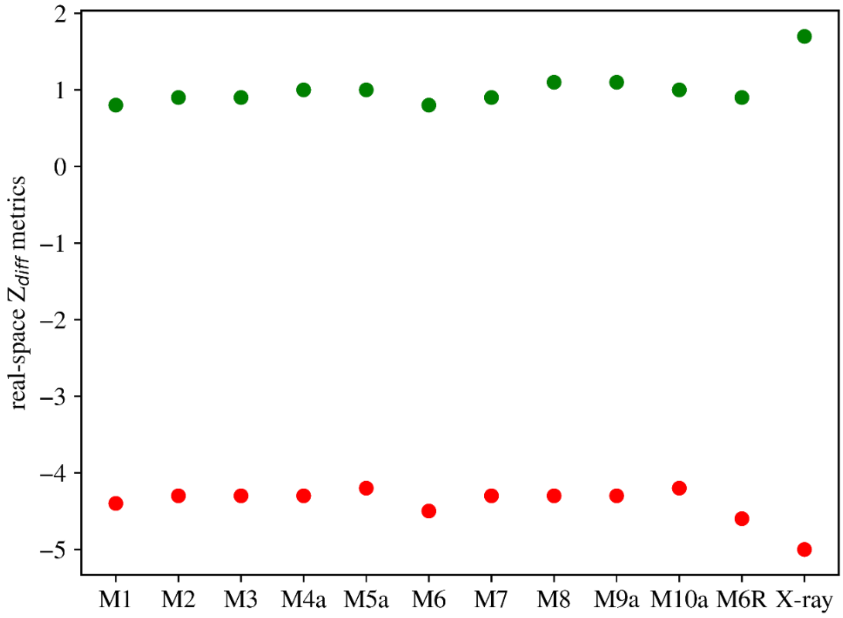

Supplementary Figure 59: Real-space Z-difference (RSZD) of darunavir. RSZD+ (green) and RSZD- (red) scores of darunavir (017) in HIV-1 from various quantum refinement schemes (**M1-M10**). Those results for X-ray were taken from the experimental structure without our further refinement.

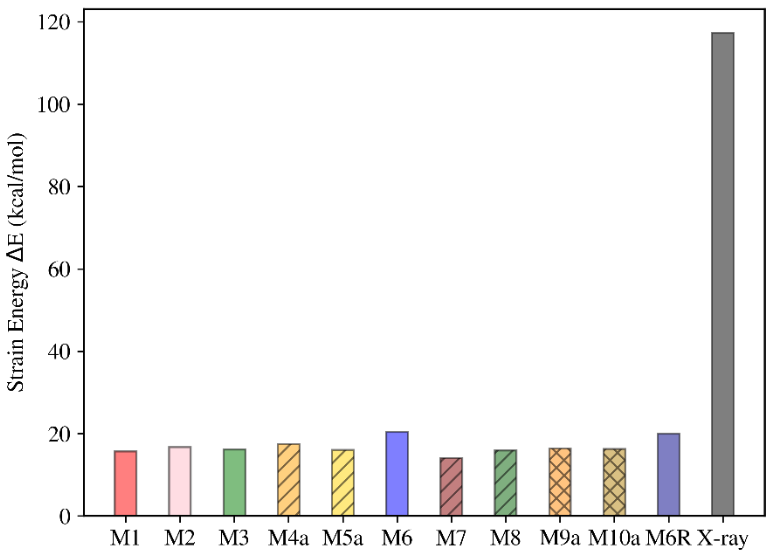

Supplementary Figure 60: Strain energy of darunavir. Strain energy (ΔE, kcal·mol^{-1}) at ωB97X-D/6-31G(d) level for darunavir (017) in HIV-1 determined by various quantum refinement schemes (**M1-M10**). Those results for X-ray were taken from the experimental structure without our further refinement.

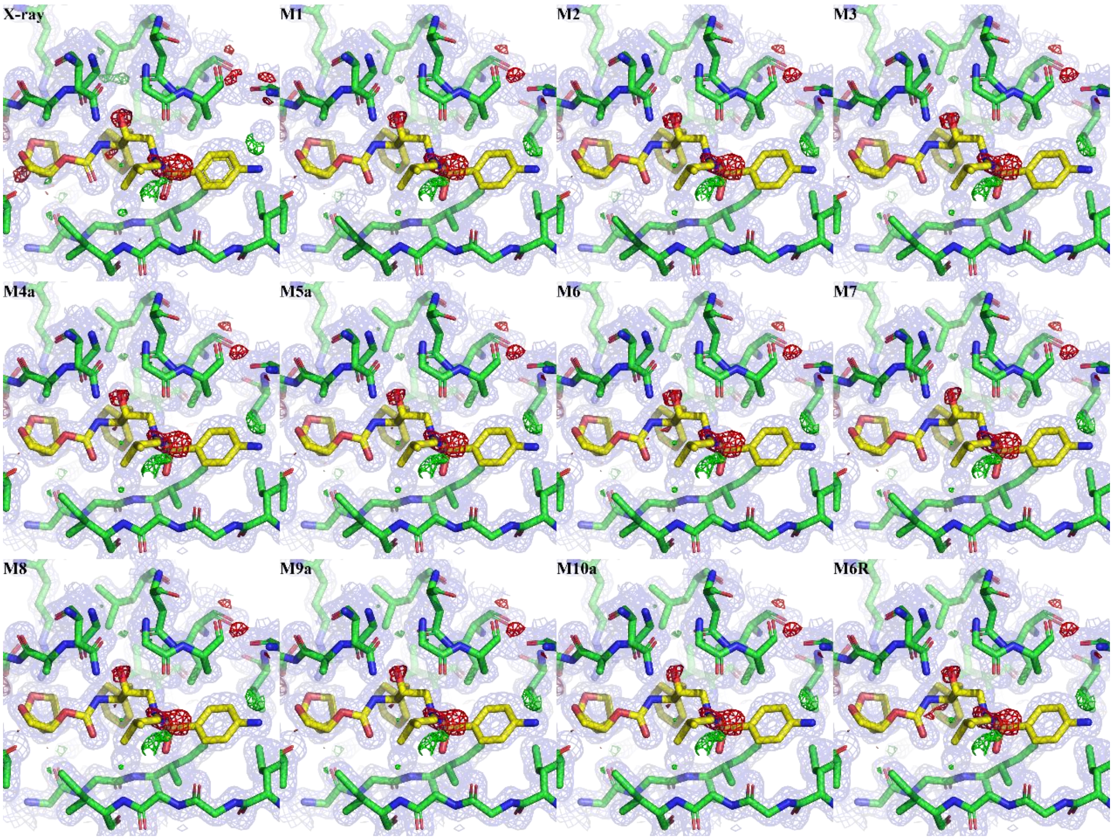

Supplementary Figure 61: Electron density maps of darunavir. Structures for darunavir (017) in HIV-1 from various quantum refinement schemes (**M1**-**M10**), including the electron density maps (2mFo-DFc maps, contoured at 1.0 σ (blue), mFo-DFc maps, contoured at +3.0 σ (green), and mFo-DFc maps, contoured at -3.0 σ (red)). Those results for X-ray were taken from the experimental structure without our further refinement.

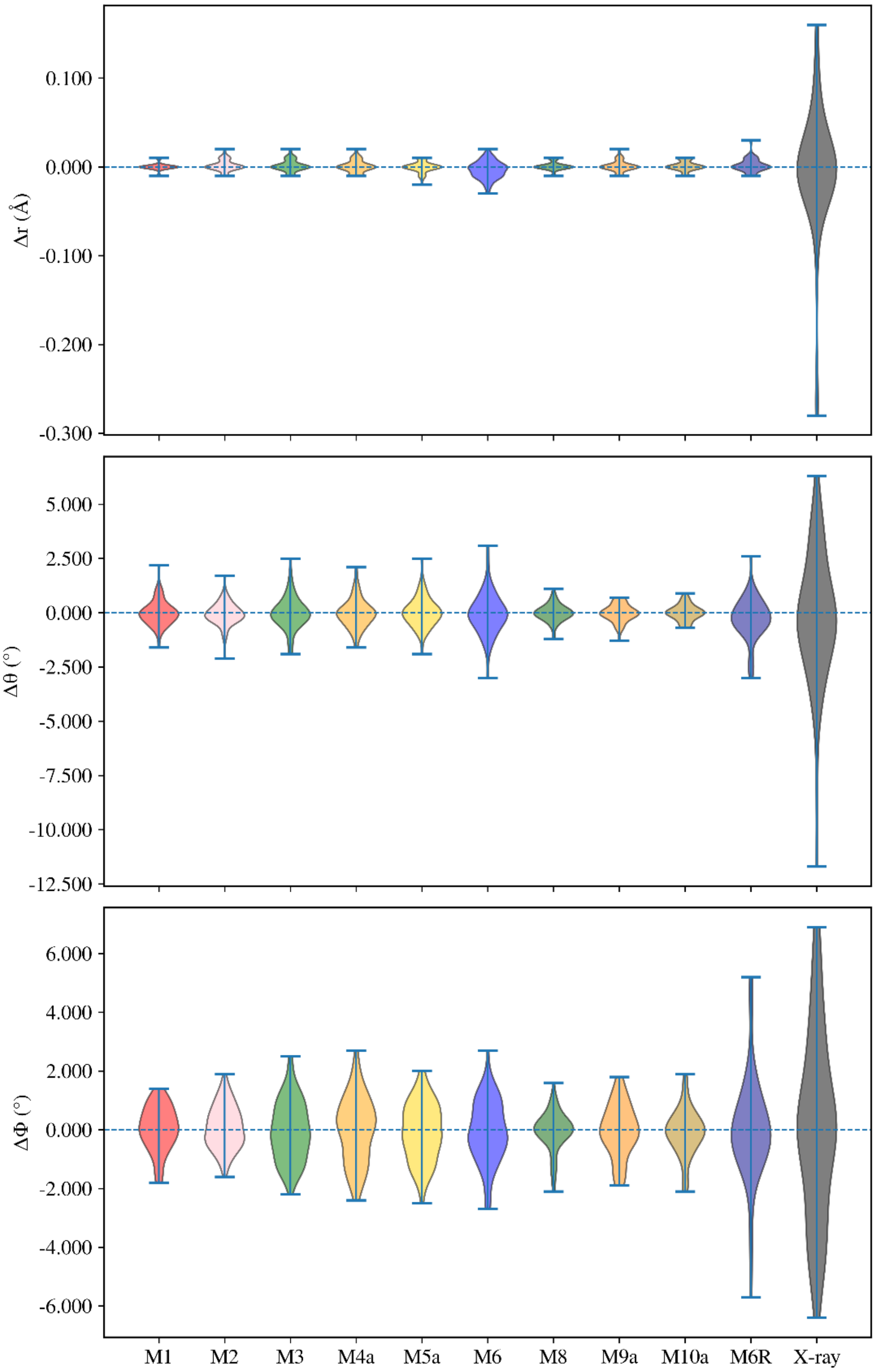

Supplementary Figure 62: Key coordinates of quantum refinement results of darunavir. Deviation in the refined bond distances (Δr, n = 41), angles (Δθ, n = 58) and dihedrals (Δϕ, n = 36) of darunavir (017) in HIV-1 from various quantum refinement schemes (**M1-M10**) and X-ray structure which are compared to those obtained from the most reliable **M7** scheme. The solid line represents the upper and lower values.

(viii) 5HLS (CPI-0610 in Bromodomain)

Protein preparations

Resolution: 2.18 Å

Ligand: 62G (CPI-0610, Pelabresib); $C_{20}H_{16}ClN_3O_2$

Residue flipped: Chain A: GLN59, GLN64, ASN93, ASN117

Protonation states (pH = 7.8): Chain A: HIE77

Optimized region: 62G

High layer: 62G

Medium layer: Chain A: TRP81, PRO82, PHE83, VAL87, LEU92, LEU94, TYR139, ASN140, ILE146, MET149, WAT323, WAT337, WAT347, WAT365, WAT395, WAT410

ω_α = 1.7995

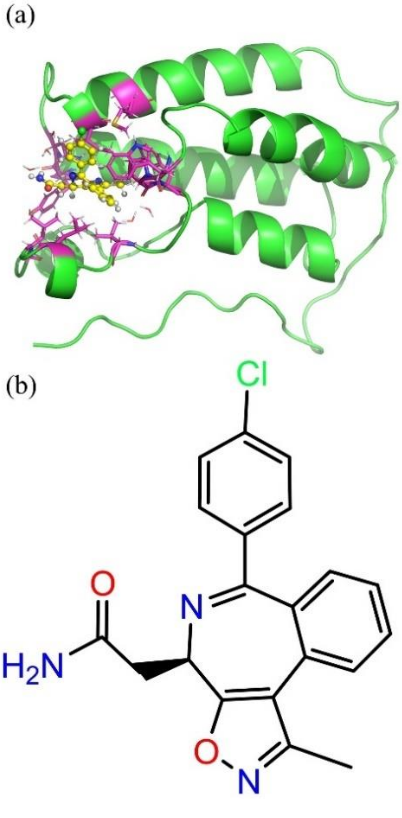

Supplementary Figure 63: CPI-0610 in Bromodomain. (**a**) Crystal structure of Bromodomain with CPI-0610 (62G). ONIOM layer by different color: yellow: high layer; red: medium layer; green: low layer. Ligand CPI-0610 is presented in stick and balls. (**b**) Structure of CPI-0610.

Quantum refined structural results:

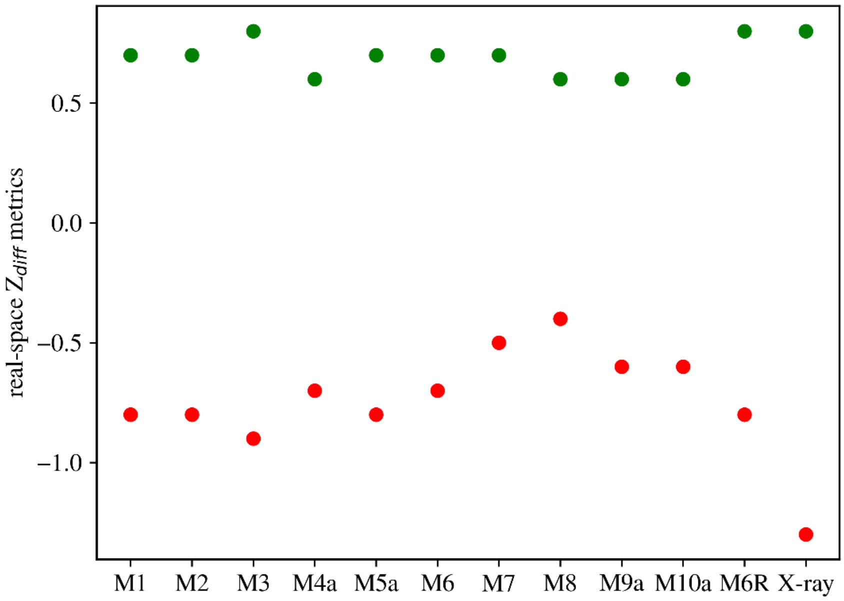

Supplementary Figure 64: Real-space Z-difference (RSZD) of CPI-0610. RSZD+ (green) and RSZD- (red) scores of CPI-0610 (62G) in Bromodomain from various quantum refinement schemes (**M1-M10**). Those results for X-ray were taken from the experimental structure without our further refinement.

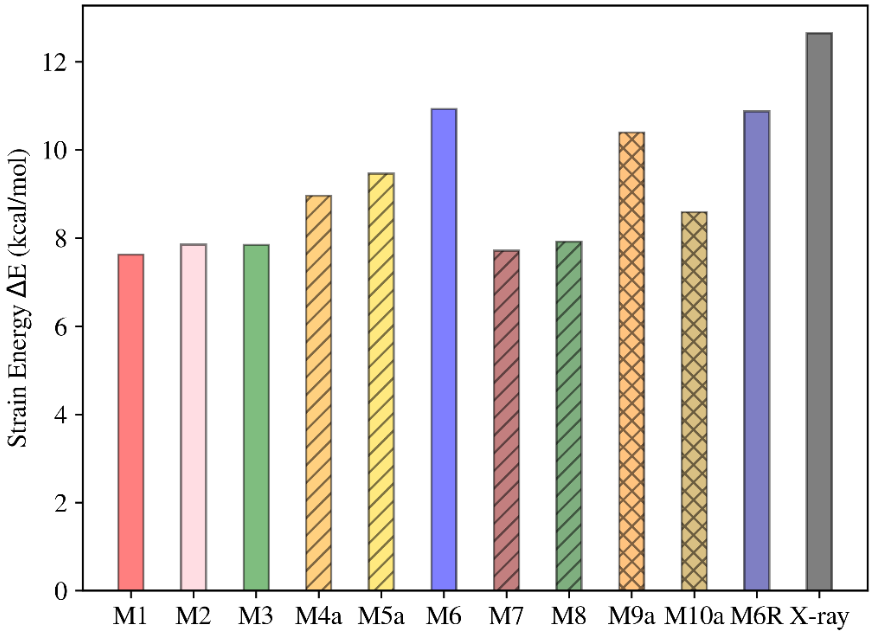

Supplementary Figure 65: Strain energy of CPI-0610. Strain energy (ΔE, kcal·mol^{-1}) at ωB97X-D/6-31G(d) level for CPI-0610 (62G) in Bromodomain determined by various quantum refinement schemes (**M1-M10**). Those results for X-ray were taken from the experimental structure without our further refinement.

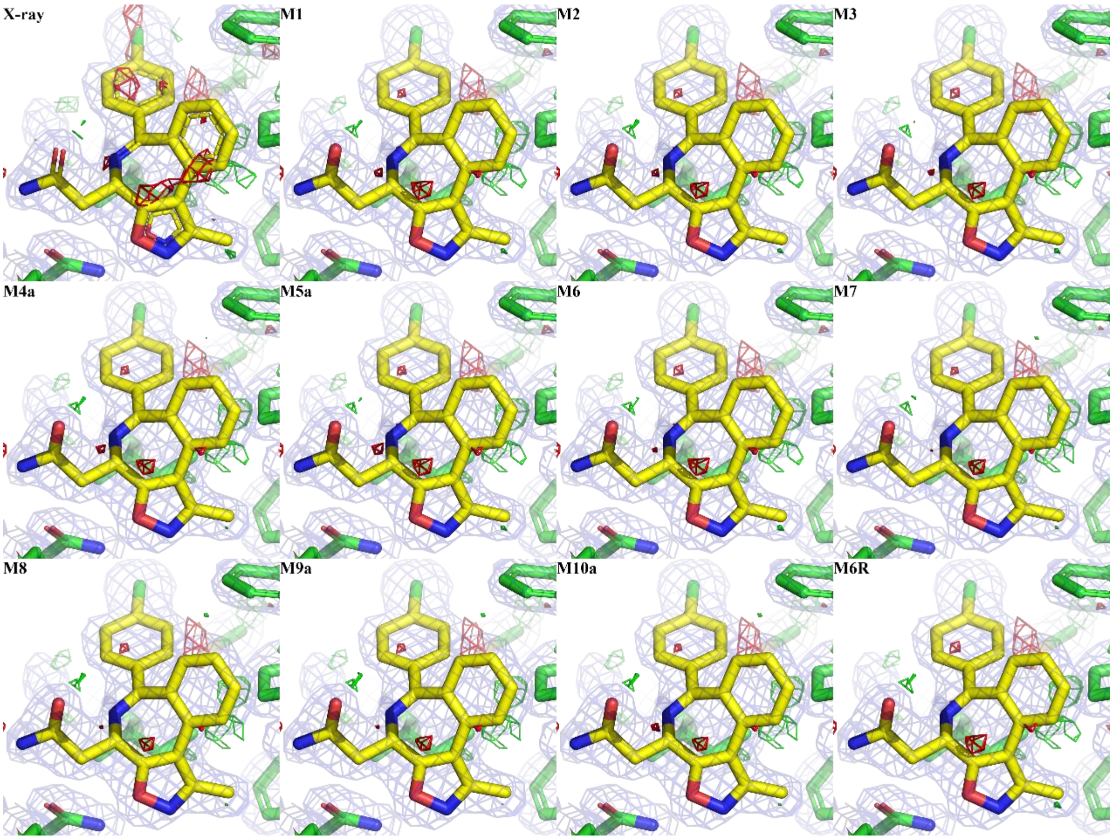

Supplementary Figure 66: Electron density maps of CPI-0610. Structures for CPI-0610 (62G) in Bromodomain from various quantum refinement schemes (**M1**-**M10**), including the electron density maps (2mFo-DFc maps, contoured at 1.0 σ (blue), mFo-DFc maps, contoured at +3.0 σ (green), and mFo-DFc maps, contoured at -3.0 σ (red)). Those results for X-ray were taken from the experimental structure without our further refinement.

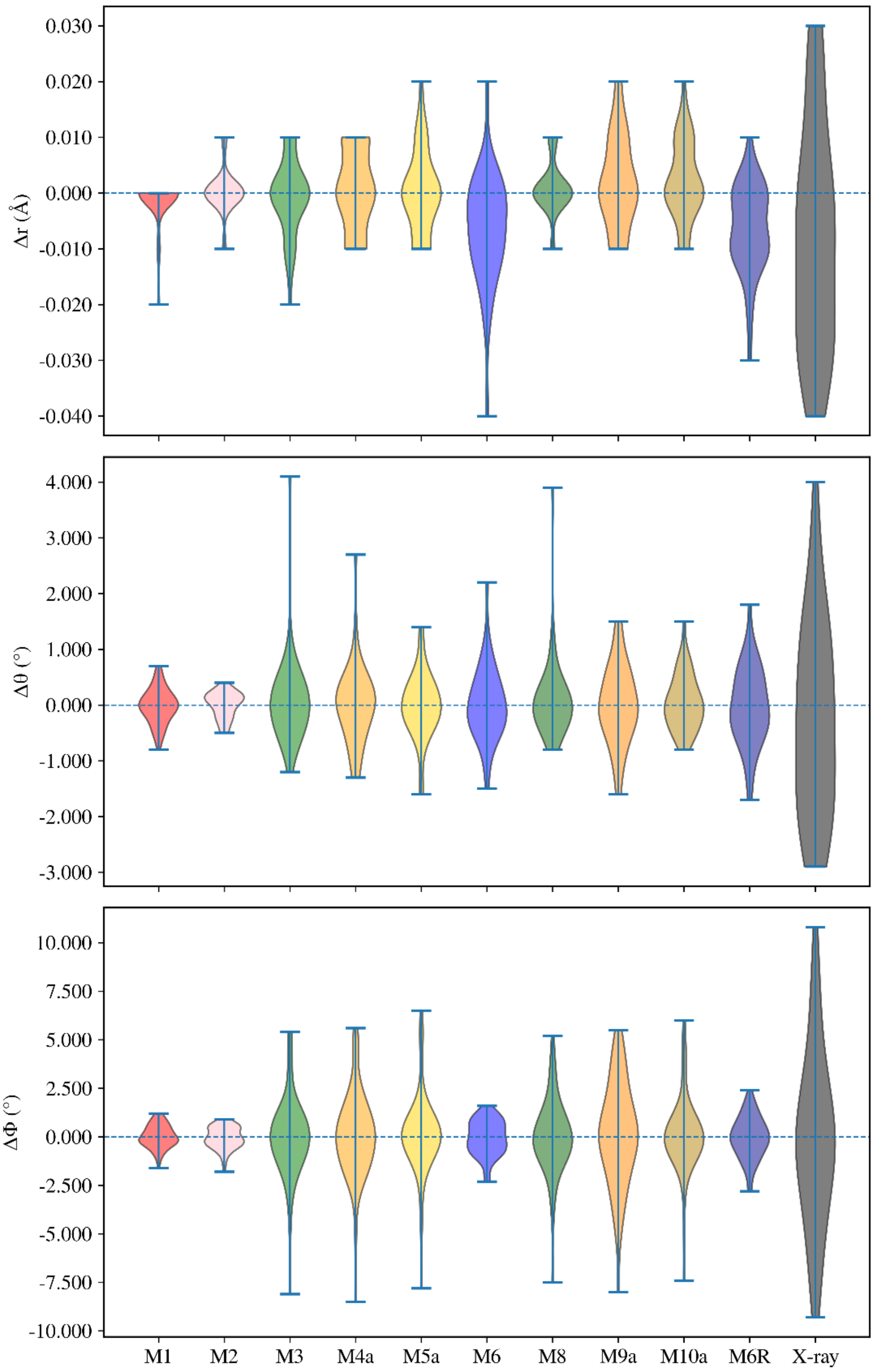

Supplementary Figure 67: Key coordinates of quantum refinement results of CPI-0610. Deviation in the refined bond distances (Δr, n = 29), angles (Δθ, n = 42) and dihedrals (Δϕ, n = 8) of CPI-0610 (62G) in Bromodomain from various quantum refinement schemes (**M1-M10**) and X-ray structure which are compared to those obtained from the most reliable **M7** scheme. The solid line represents the upper and lower values.

(ix) 4P6W (Mometasone in hormone receptor)

Protein preparations

Resolution: 1.95 Å

Ligand: MOF (Mometasone, Asmanex HFA); $C_{27}H_{30}Cl_2O_6$

Residue flipped: Chain A: HIS588, GLN570

Addition of the missing atoms: 2 missing heavy atoms

Protonation states (pH = 6.5): Chain A: HIP588, HID645, HID654, HIE726, HIE775

Optimized region: MOF

High layer: MOF

Medium layer: Chain A: MET560, LEU563, ASN564, LEU566, GLY567, GLN570, TRP600, MET601, MET604, ALA605, LEU608, ARG611, PHE623, ILE629, MET639, GLN642, CYS643, MET646, LEU732, TYR735, CYS736, THR739, WAT938

ω_α = 0.46877

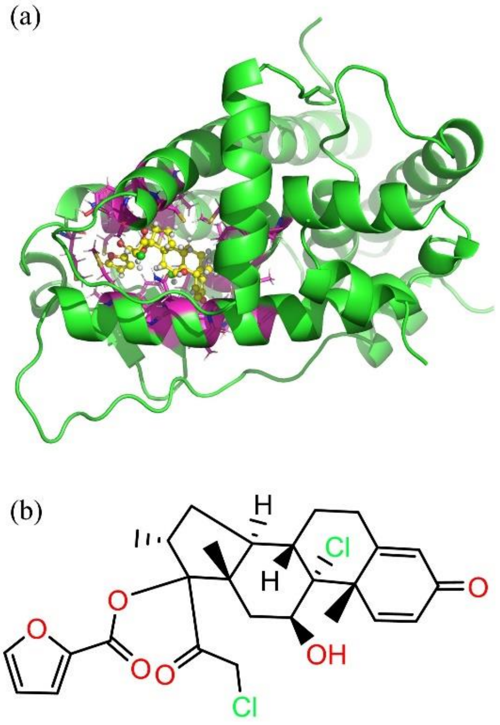

Supplementary Figure 68: Mometasone in hormone receptor. (**a**) Crystal structure of hormone receptor with mometasone (MOF). ONIOM layer by different color: yellow: high layer; red: medium layer; green: low layer. Ligand mometasone is presented in stick and balls. (**b**) Structure of mometasone.

Quantum refined structural results:

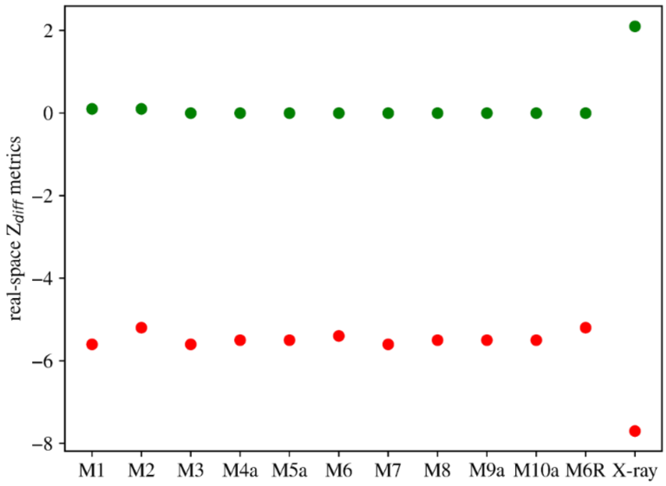

Supplementary Figure 69: Real-space Z-difference (RSZD) of mometasone. RSZD+ (green) and RSZD- (red) scores of mometasone (MOF) in hormone receptor from various quantum refinement schemes (**M1-M10**). Those results for X-ray were taken from the experimental structure without our further refinement.

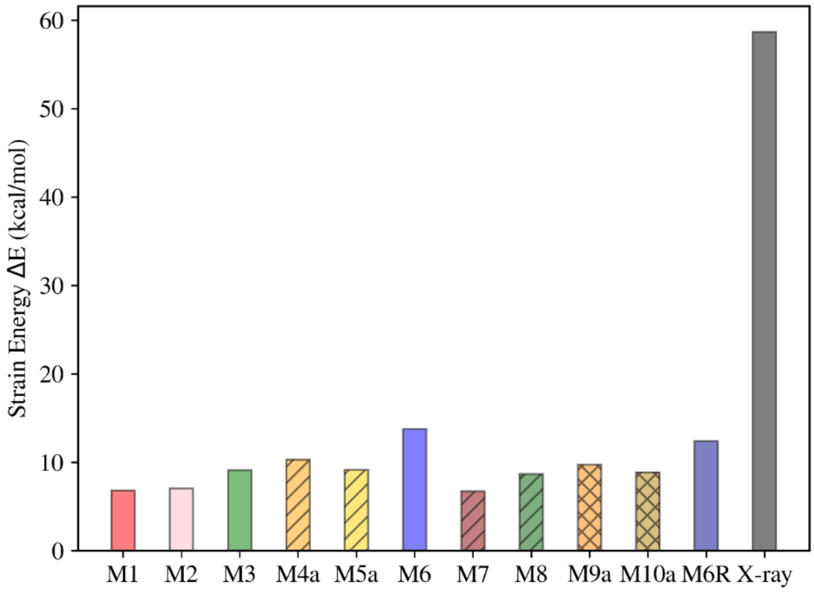

Supplementary Figure 70: Strain energy of mometasone. Strain energy (ΔE, kcal·mol^{-1}) at ωB97X-D/6-31G(d) level for mometasone (MOF) in hormone receptor determined by various quantum refinement schemes (**M1-M10**). Those results for X-ray were taken from the experimental structure without our further refinement.

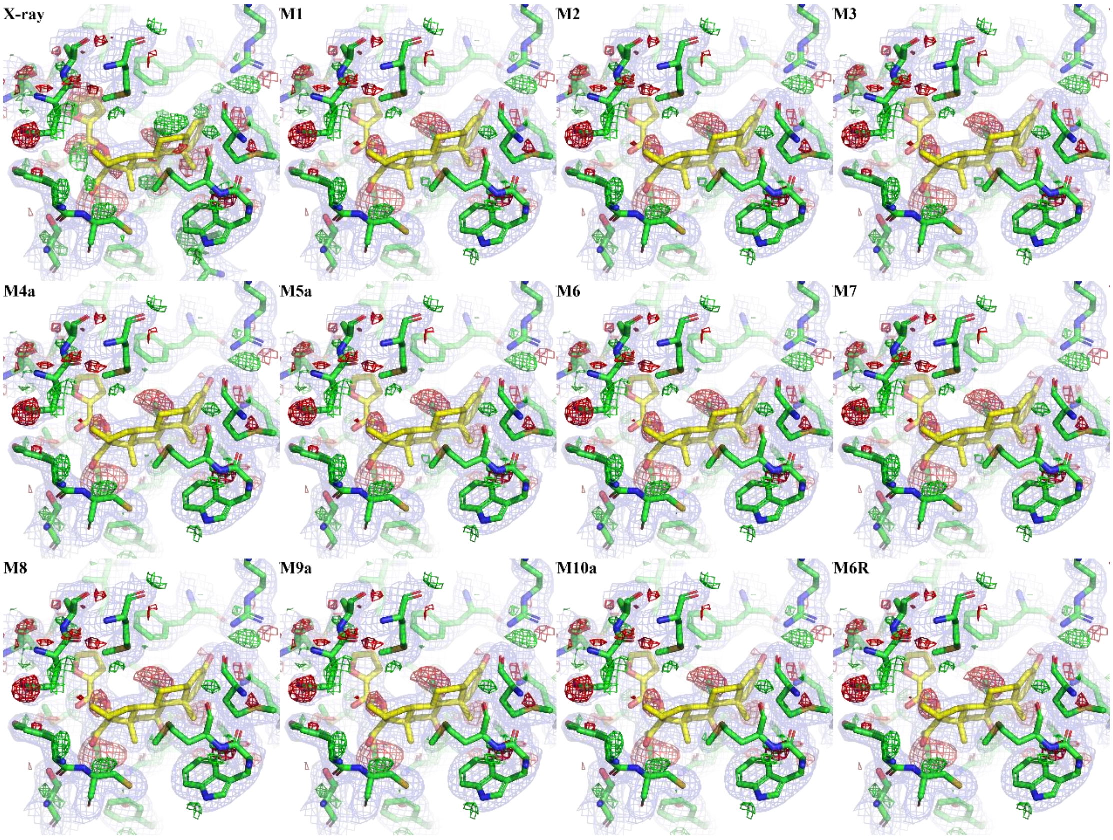

Supplementary Figure 71: Electron density maps of mometasone. Structures for mometasone (MOF) in hormone receptor from various quantum refinement schemes (**M1**-**M10**), including the electron density maps (2mFo-DFc maps, contoured at 1.0 σ (blue), mFo-DFc maps, contoured at +3.0 σ (green), and mFo-DFc maps, contoured at -3.0 σ (red)). Those results for X-ray were taken from the experimental structure without our further refinement.

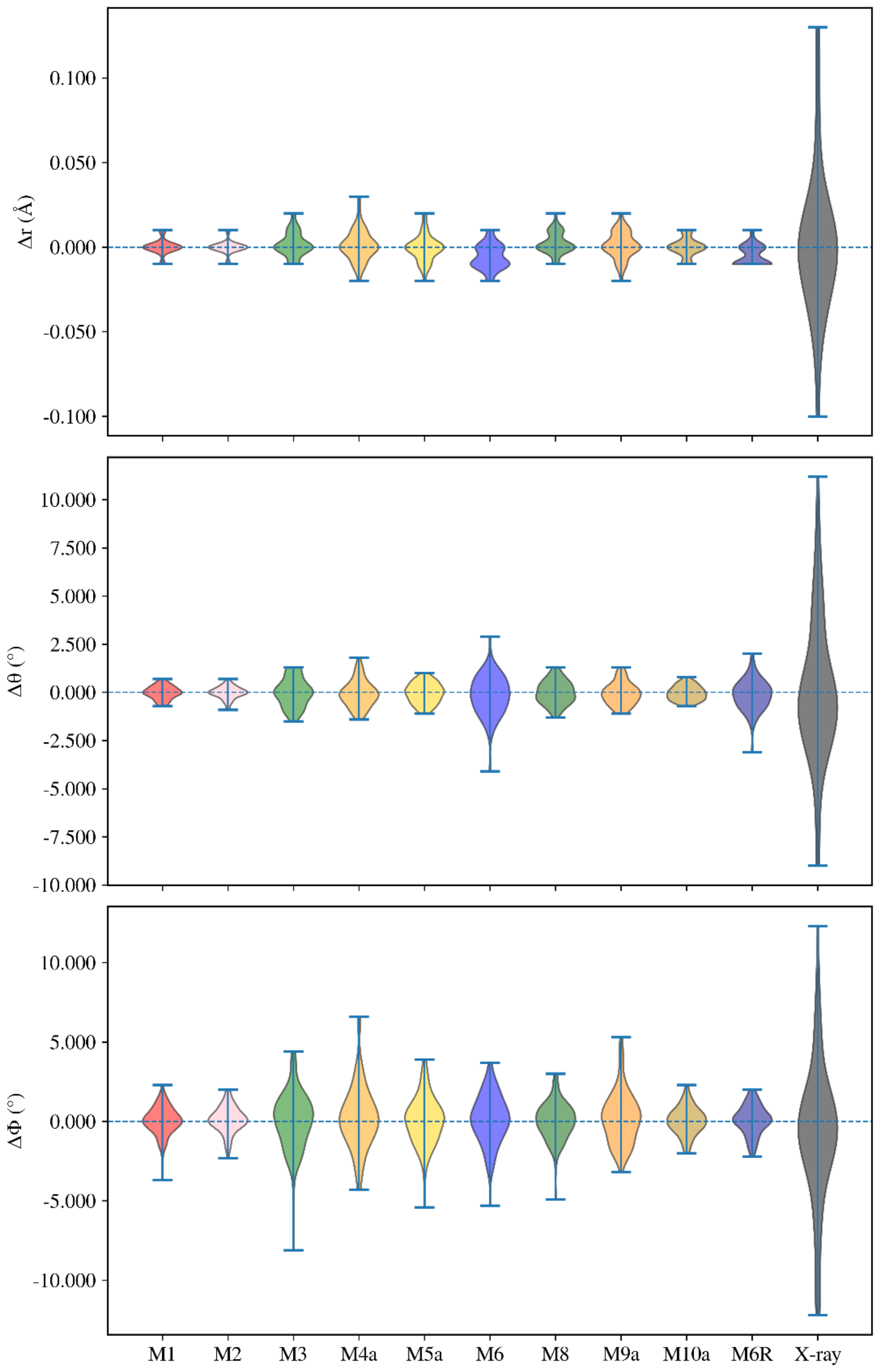

Supplementary Figure 72: Key coordinates of quantum refinement results of mometasone. Deviation in the refined bond distances (Δr, n = 39), angles (Δθ, n = 64) and dihedrals (Δϕ, n = 17) of mometasone (MOF) in hormone receptor from various quantum refinement schemes (**M1-M10**) and X-ray structure which are compared to those obtained from the most reliable **M7** scheme. The solid line represents the upper and lower values.

(x) 4KRA (Ciprofloxacin in salmonella typhi ompF)
Protein preparations

Resolution: 3.32 Å

Ligand: CPF (Ciprofloxacin, Proquin XR); $C_{17}H_{18}FN_3O_3$

Residue flipped:

Chain A: ASN9, GLN46, ASN74, ASN159, ASN205, ASN240, ASN250, ASN282, ASN303, ASN307, ASN315, ASN320,

Chain B: ASN9, HIS21, ASN139, GLN156, ASN159, ASN169, ASN307, ASN315,

Chain C: GLN34, ASN48, GLN70, ASN159, ASN166, ASN192, GLN262, ASN303

Addition of the missing atoms: 332 missing heavy atoms

Protonation states (pH = 5.6):

Chain A: HIE21, ASH33, GLH69, ASH92, GLH104, GLH116, HIP163, ASH201, GLH256, ASH311,

Chain B: HIE21, ASH33, ASH92, GLH104, ASH124, HIE163, GLH247, GLH256, ASH311,

Chain C: HIP21, ASH92, ASH102, GLH104, ASH108, HID163, ASH203, GLH256, ASH311

Optimized region: CPF

High layer: CPF

Medium layer: Chain C: ARG20, ARG60, ARG77, TYR112, PHE113, SER114, GLY115, GLU116, ALA121, ARG130

ω_{α} = 4.9010

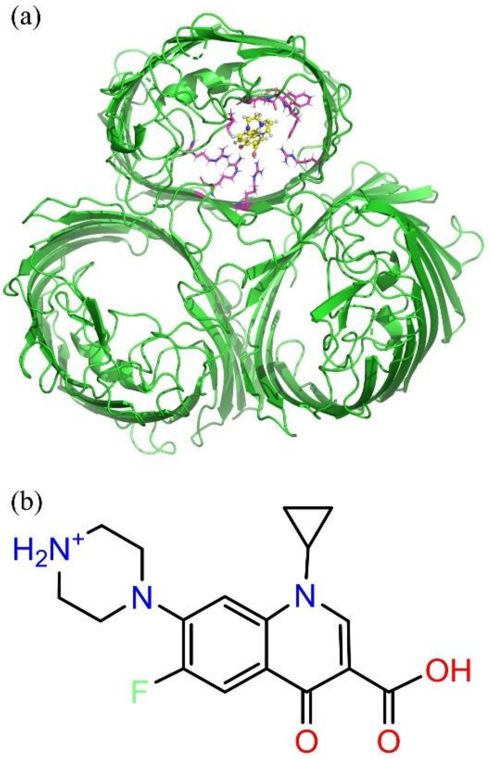

Supplementary Figure 73: Ciprofloxacin in salmonella typhi ompF. (**a**) Crystal structure of Salmonella typhi OmpF with ciprofloxacin (CPF). ONIOM layer by different color: yellow: high layer; red: medium layer; green: low layer. Ligand ciprofloxacin is presented in stick and balls. (**b**) Structure of ciprofloxacin.

Quantum refined structural results:

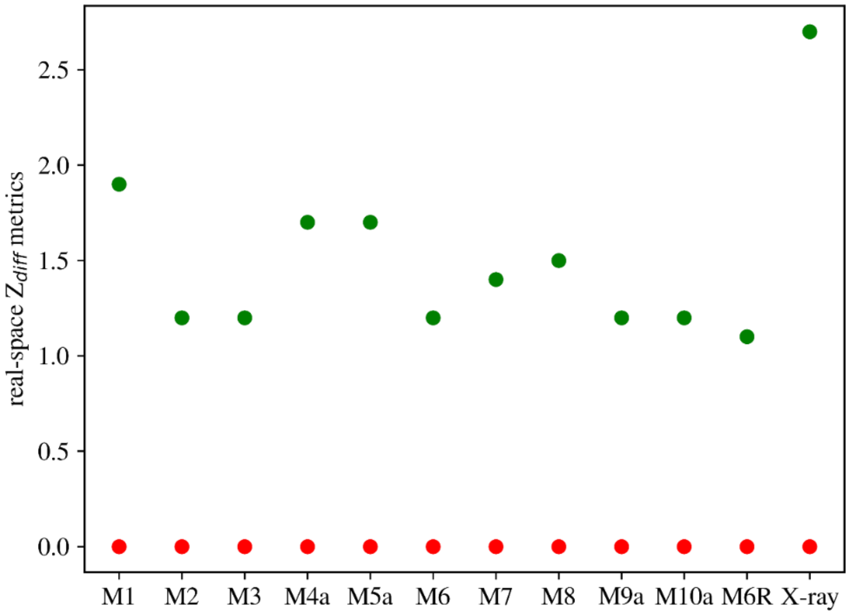

Supplementary Figure 74: Real-space Z-difference (RSZD) of ciprofloxacin. RSZD+ (green) and RSZD- (red) scores of ciprofloxacin (CPF) in Salmonella typhi OmpF from various quantum refinement schemes (**M1-M10**). Those results for X-ray were taken from the experimental structure without our further refinement.

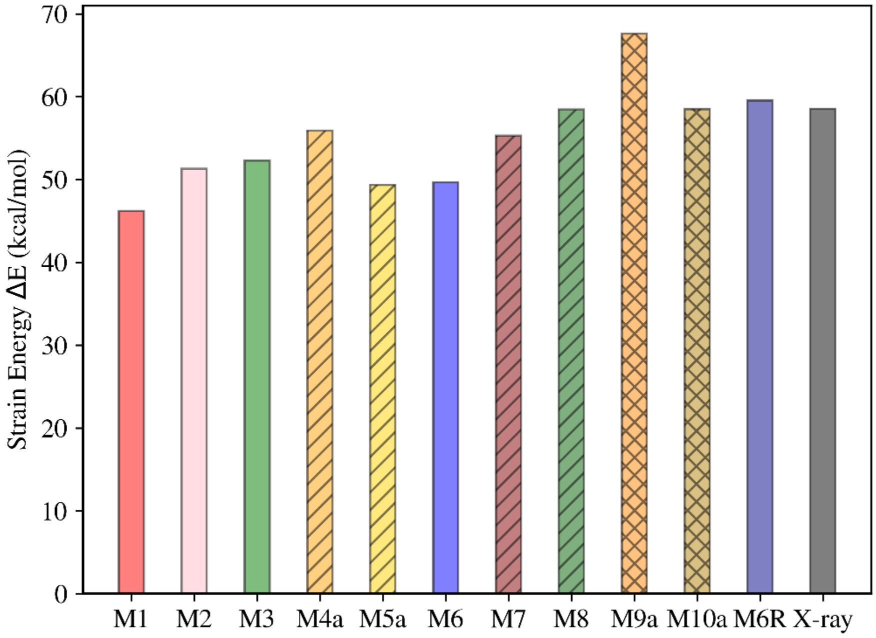

Supplementary Figure 75: Strain energy of ciprofloxacin. Strain energy (ΔE, kcal·mol^{-1}) at ωB97X-D/6-31G(d) level for ciprofloxacin (CPF) in Salmonella typhi OmpF determined by various quantum refinement schemes (**M1-M10**). Those results for X-ray were taken from the experimental structure without our further refinement.

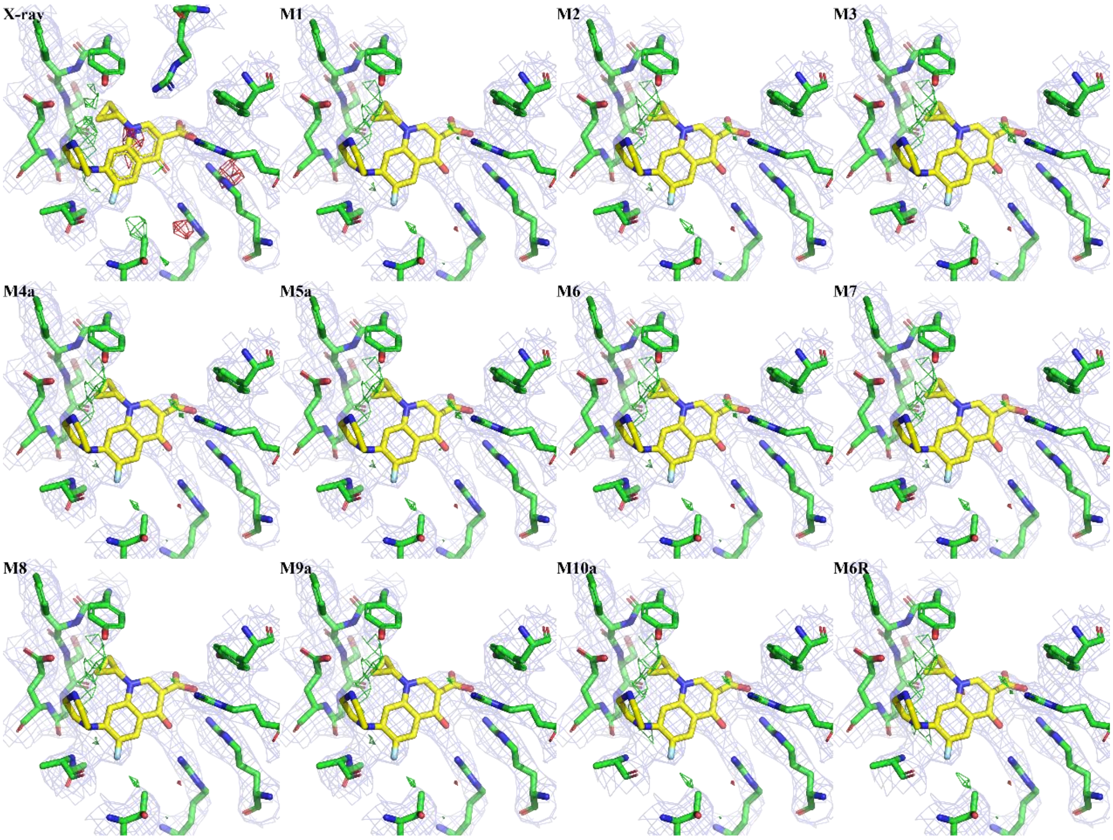

Supplementary Figure 76: Electron density maps of ciprofloxacin. Structures for ciprofloxacin (CPF) in Salmonella typhi OmpF from various quantum refinement schemes (**M1**-**M10**), including the electron density maps (2mFo-DFc maps, contoured at 1.0 σ (blue), mFo-DFc maps, contoured at +3.0 σ (green), and mFo-DFc maps, contoured at -3.0 σ (red)). Those results for X-ray were taken from the experimental structure without our further refinement.

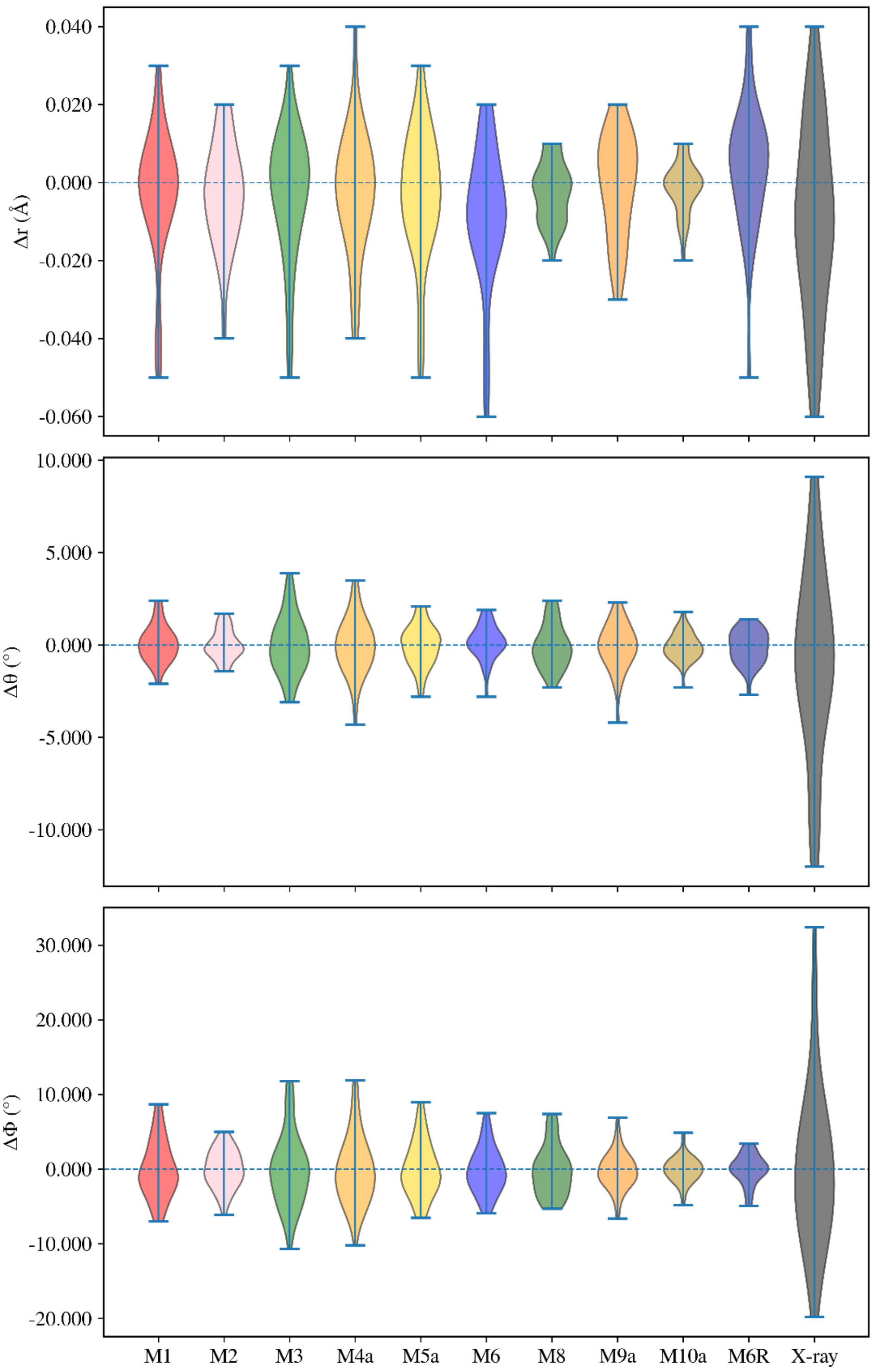

Supplementary Figure 77: Key coordinates of quantum refinement results of ciprofloxacin. Deviation in the refined bond distances (Δr, n = 27), angles (Δθ, n = 37) and dihedrals (Δϕ, n = 12) of ciprofloxacin (CPF) in Salmonella typhi OmpF from various quantum refinement schemes (**M1-M10**) and X-ray structure which are compared to those obtained from the most reliable **M7** scheme. The solid line represents the upper and lower values.

(xi) 3QQA (CmeR-bile acid complexes from Campylobacter jejuni)

Protein preparations:

Resolution: 2.20 Å

Ligand: TCH (Taurocholic acid); $C_{26}H_{45}NO_7S$

Residue flipped: Chain A: GLN81, HIS72, ASN157, ASN181

Protonation states (pH = 8): Chain A: HID72, HID74, HID125, LYN170, HID174, HID175, HID193

Optimized region: TCH

High layer: TCH

Medium layer: LEU65, ILE68, HID72, PHE103, ALA108, ILE115, TRP129, ILE130, GLN134, VAL163, LYN170, HID174, WAT257

ω_α = 1.2376

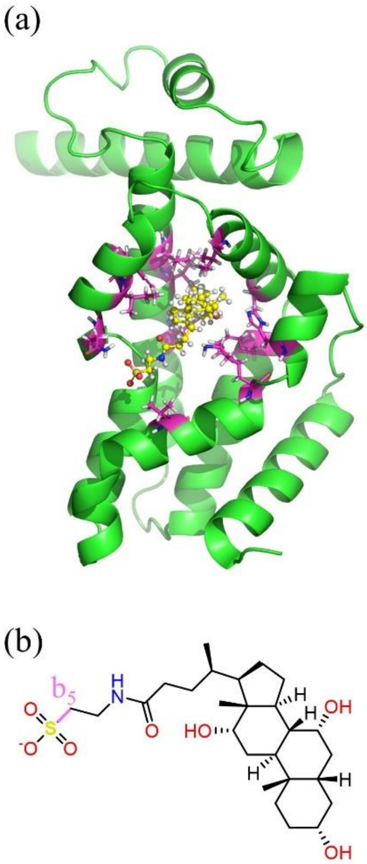

Supplementary Figure 78: CmeR-bile acid complexes from Campylobacter jejuni. (**a**) Crystal structure of CmeR with Taurocholic acid (TCH). ONIOM layers by different colors: yellow: high layer; red: medium layer; green: low layer. Ligand Taurocholic acid is presented in stick and balls. (**b**) Structure of Taurocholic acid.

Quantum refined structural results:

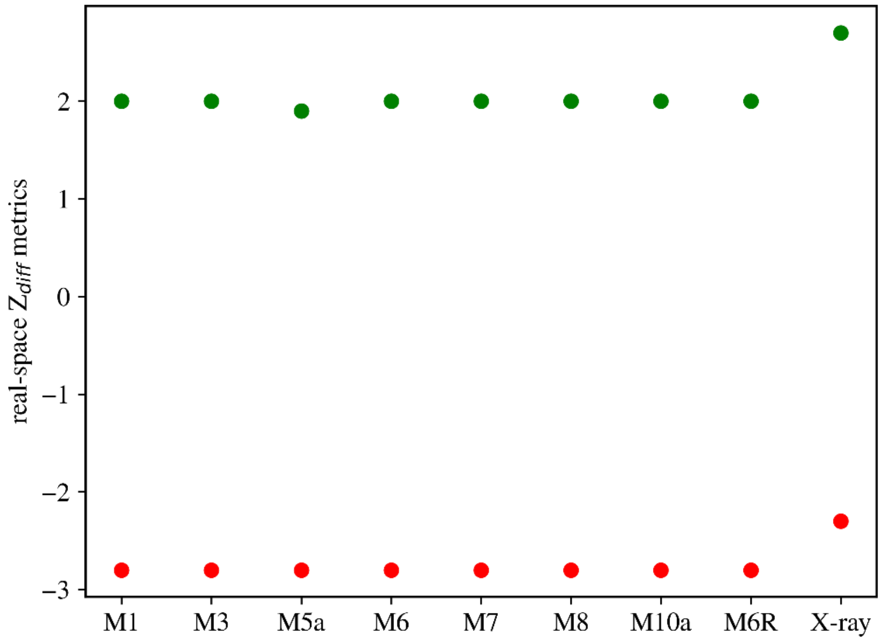

Supplementary Figure 79: Real-space Z-difference (RSZD) of taurocholic acid. RSZD+ (green) and RSZD- (red) scores of taurocholic acid (TCH) in CmeR from various quantum refinement schemes (**M1-M10**). Those results for X-ray were taken from the experimental structure without our further refinement.

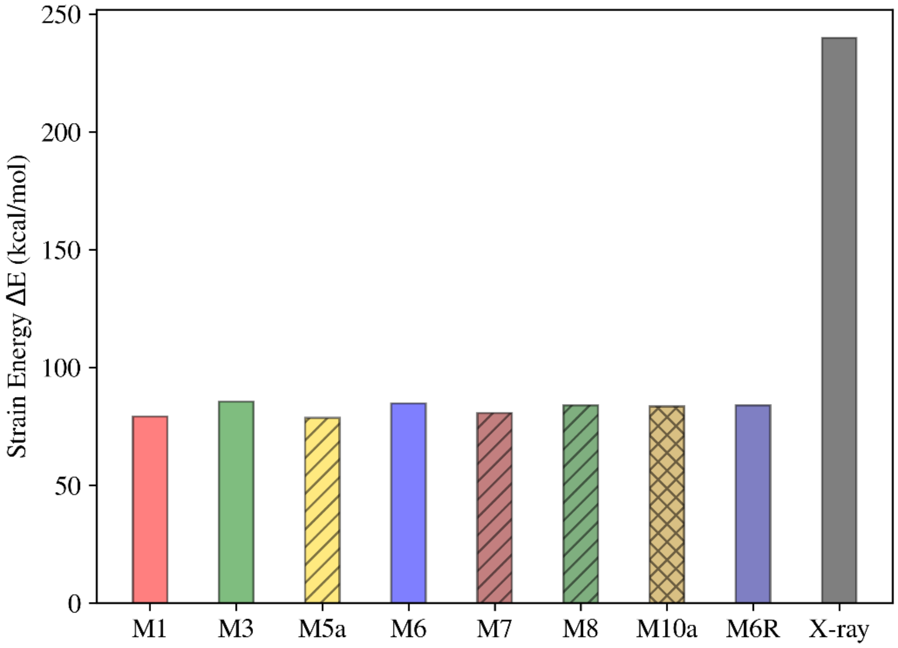

Supplementary Figure 80: Strain energy of taurocholic acid. Strain energy (ΔE, kcal·mol^{-1}) at ωB97X-D/6-31G(d) level for taurocholic acid (TCH) in CmeR determined by various quantum refinement schemes (**M1-M10**). Those results for X-ray were taken from the experimental structure without our further refinement.

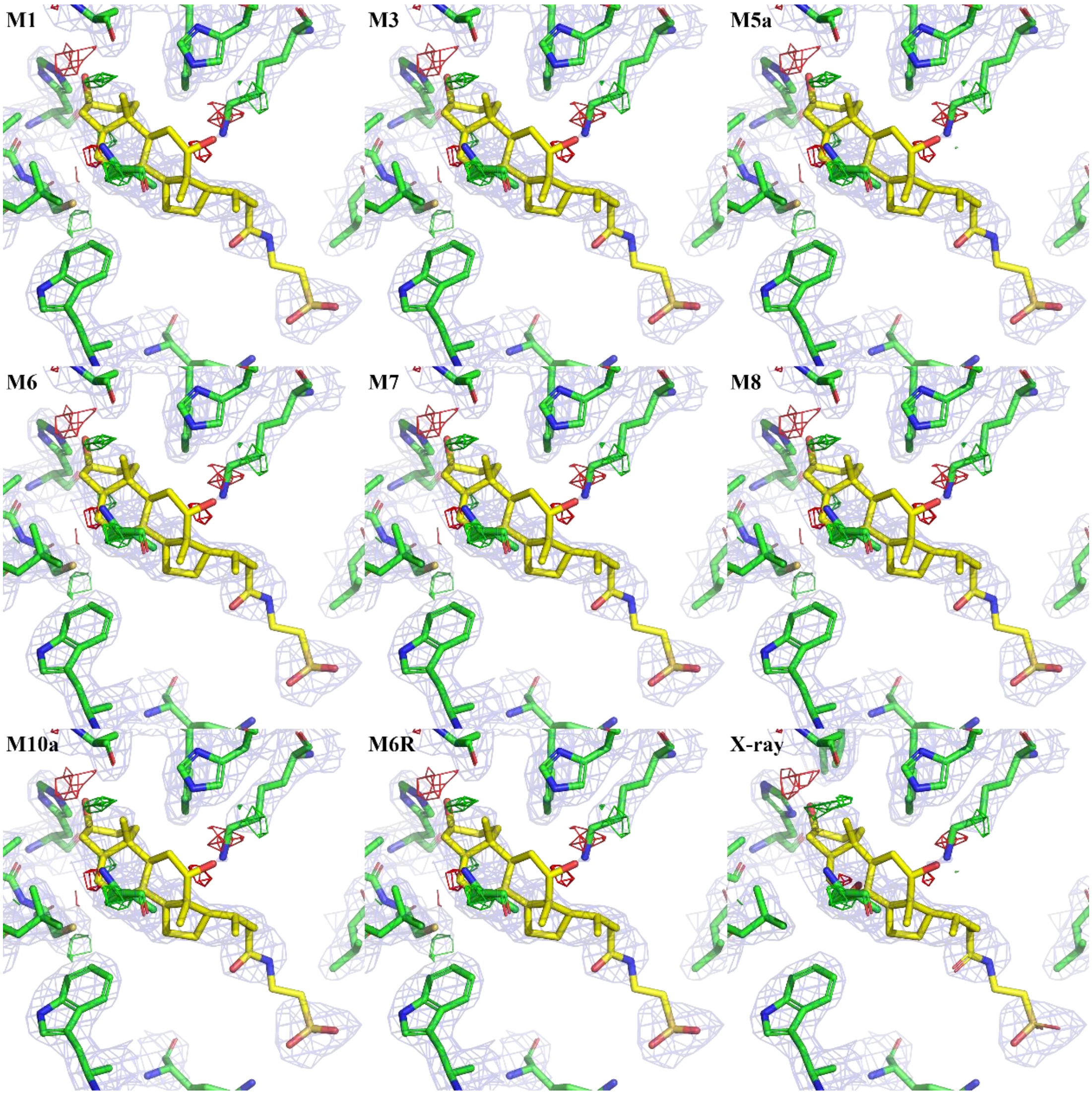

Supplementary Figure 81: Electron density maps of taurocholic acid. Structures for taurocholic acid (TCH) in CmeR from various quantum refinement schemes (**M1-M10**), including the electron density maps (2mFo-DFc maps, contoured at 1.0 σ (blue), mFo-DFc maps, contoured at +3.0 σ (green), and mFo-DFc maps, contoured at -3.0 σ (red)). Those results for X-ray were taken from the experimental structure without our further refinement.

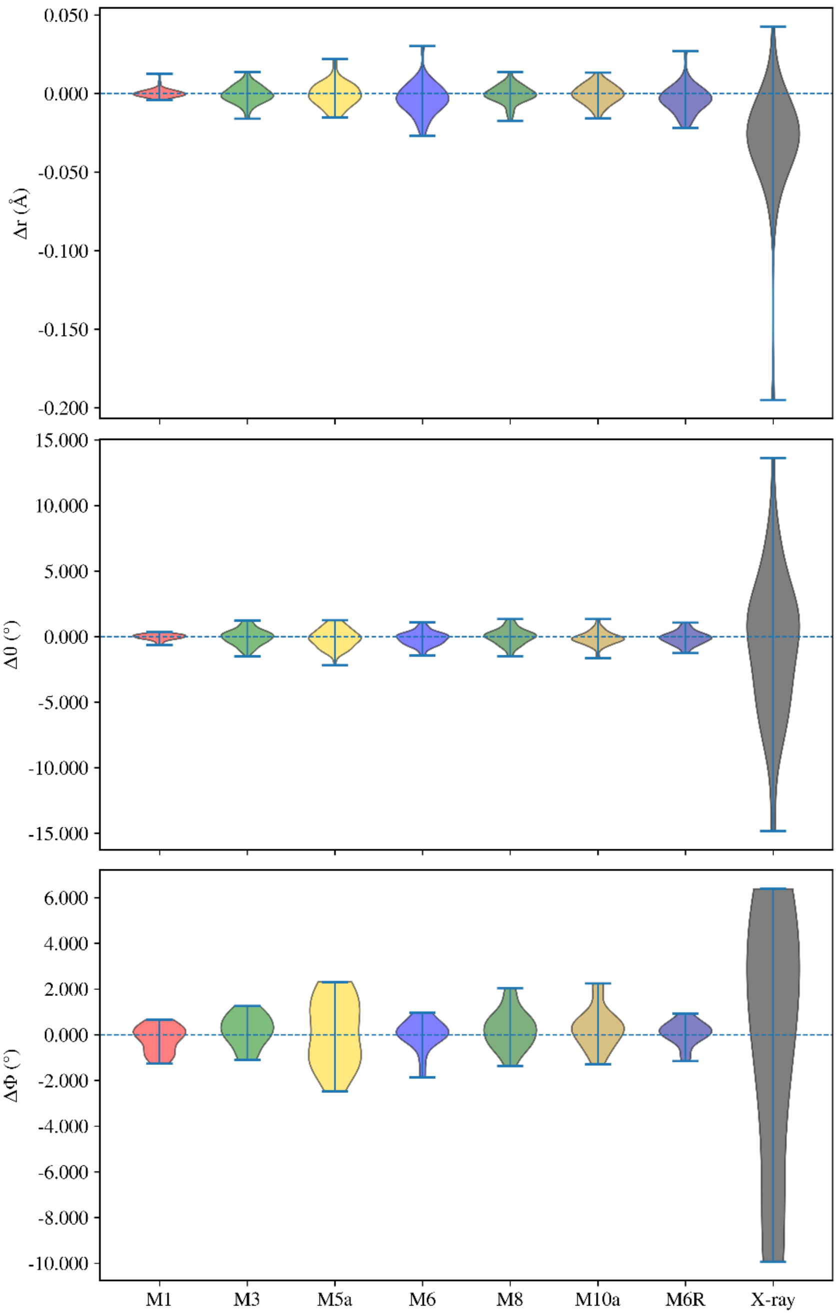

Supplementary Figure 82: Key coordinates of quantum refinement results of taurocholic acid. Deviation in the refined bond distances (Δr, n = 38), angles (Δθ, n = 60) and dihedrals (Δϕ, n = 16) of taurocholic acid (TCH) in CmeR from various quantum refinement schemes (**M1-M10**) and X-ray structure which are compared to those obtained from the most reliable **M7** scheme. The solid line represents the upper and lower values.

(xii) 4XH6 (Hispidulin in proto-oncogene kinase Pim1)

Protein preparations:

Resolution: 2.04 Å

Ligand: HUL (Hispidulin); $C_{16}H_{12}O_6$

Residue flipped: Chain A: HIS244, GLN297

Protonation states (pH = 6.5): Chain A: HID68, HID157, HID159, HID165, HID216, HID219, ASH234, HIP244, HID265, HID287, HID303, HIP305

Optimized region: HUL

High layer: HUL

Medium layer: Chain A: LEU44, VAL52, LYS67, LEU93, ILE104, LEU120, VAL126, LEU174, ILE185, ASP186, PHE187, WAT565

ω_α = 0.71562

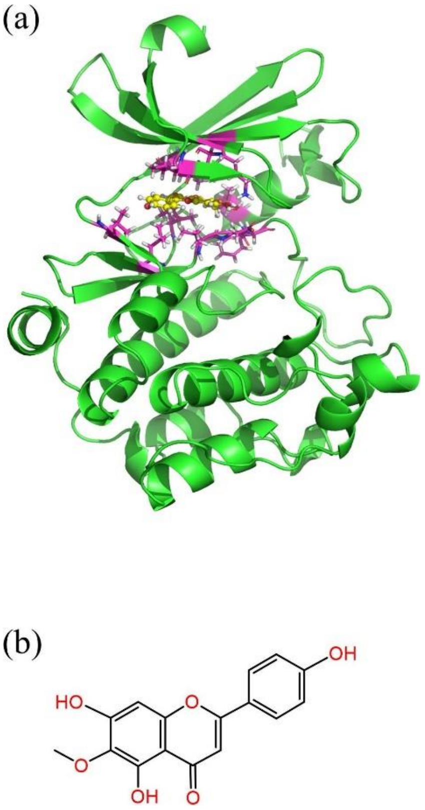

Supplementary Figure 83: Hispidulin in proto-oncogene kinase Pim1. (**a**) Crystal structure of Pim1 with hispidulin (HUL). ONIOM layers by different colors: yellow: high layer; red: medium layer; green: low layer. Ligand imatinib is presented in stick and balls. (**b**) Structure of hispidulin.

Quantum refined structural results:

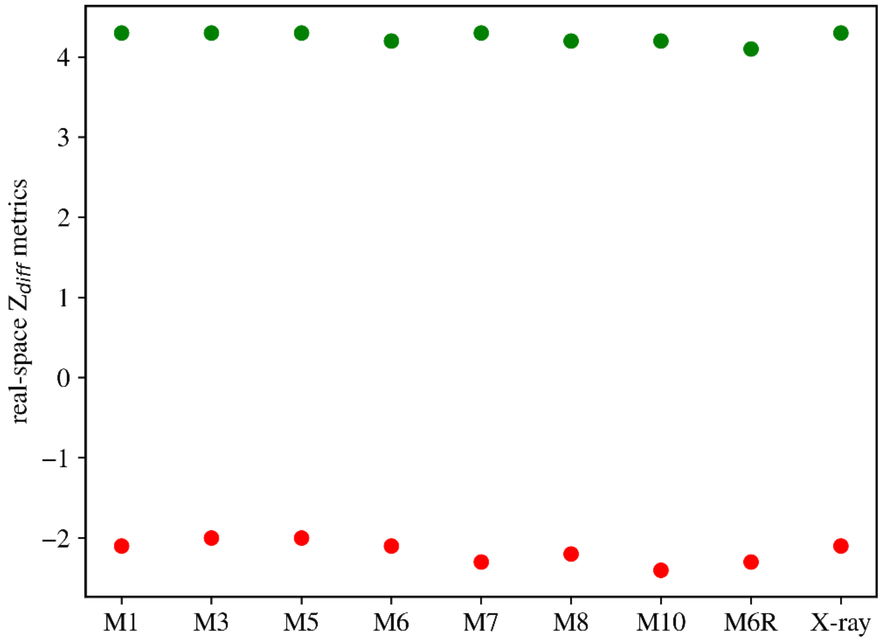

Supplementary Figure 84: Real-space Z-difference (RSZD) of hispidulin. RSZD+ (green) and RSZD- (red) scores of hispidulin (HUL) in Pim1 from various quantum refinement schemes (**M1-M10**). Those results for X-ray were taken from the experimental structure without our further refinement.

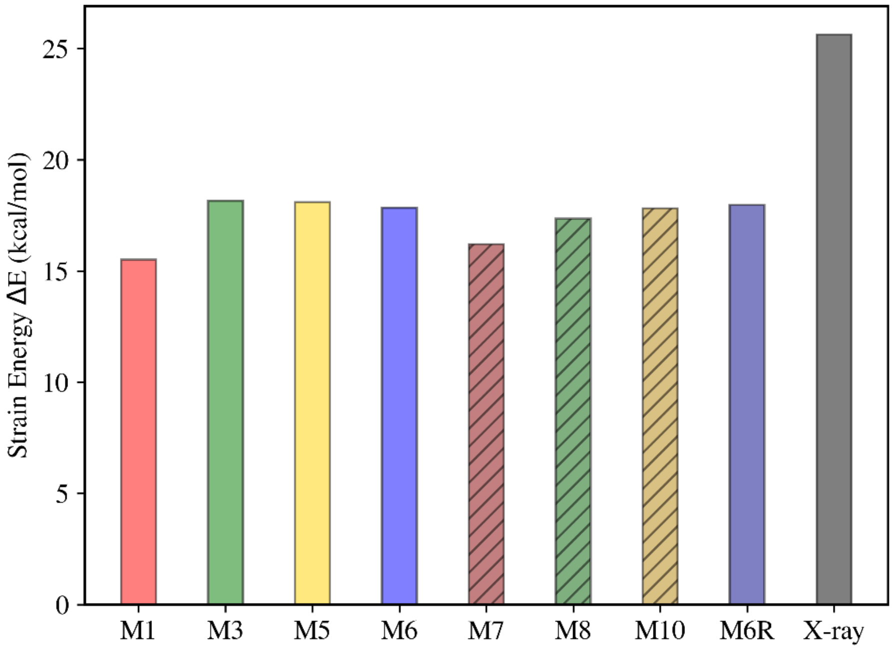

Supplementary Figure 85: Strain energy of hispidulin. Strain energy (ΔE, kcal·mol^{-1}) at ωB97X-D/6-31G(d) level for hispidulin (HUL) in Pim1 determined by various quantum refinement schemes (**M1-M10**). Those results for X-ray were taken from the experimental structure without our further refinement.

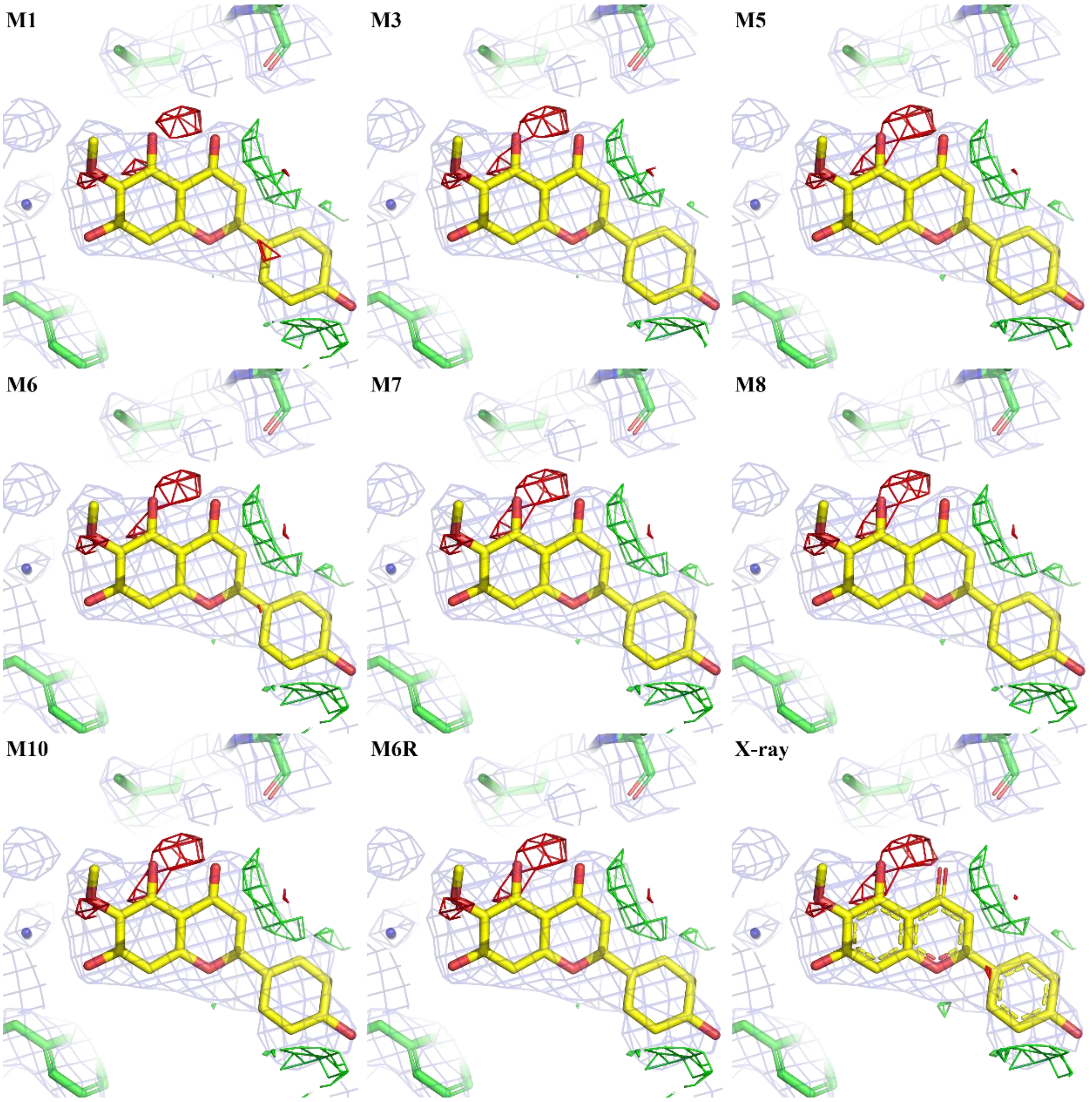

Supplementary Figure 86: Electron density maps of hispidulin. Structures for hispidulin (HUL) in Pim1 from various quantum refinement schemes (**M1**-**M10**), including the electron density maps (2mFo-DFc maps, contoured at 1.0 σ (blue), mFo-DFc maps, contoured at +3.0 σ (green), and mFo-DFc maps, contoured at -3.0 σ (red)). Those results for X-ray were taken from the experimental structure without our further refinement.

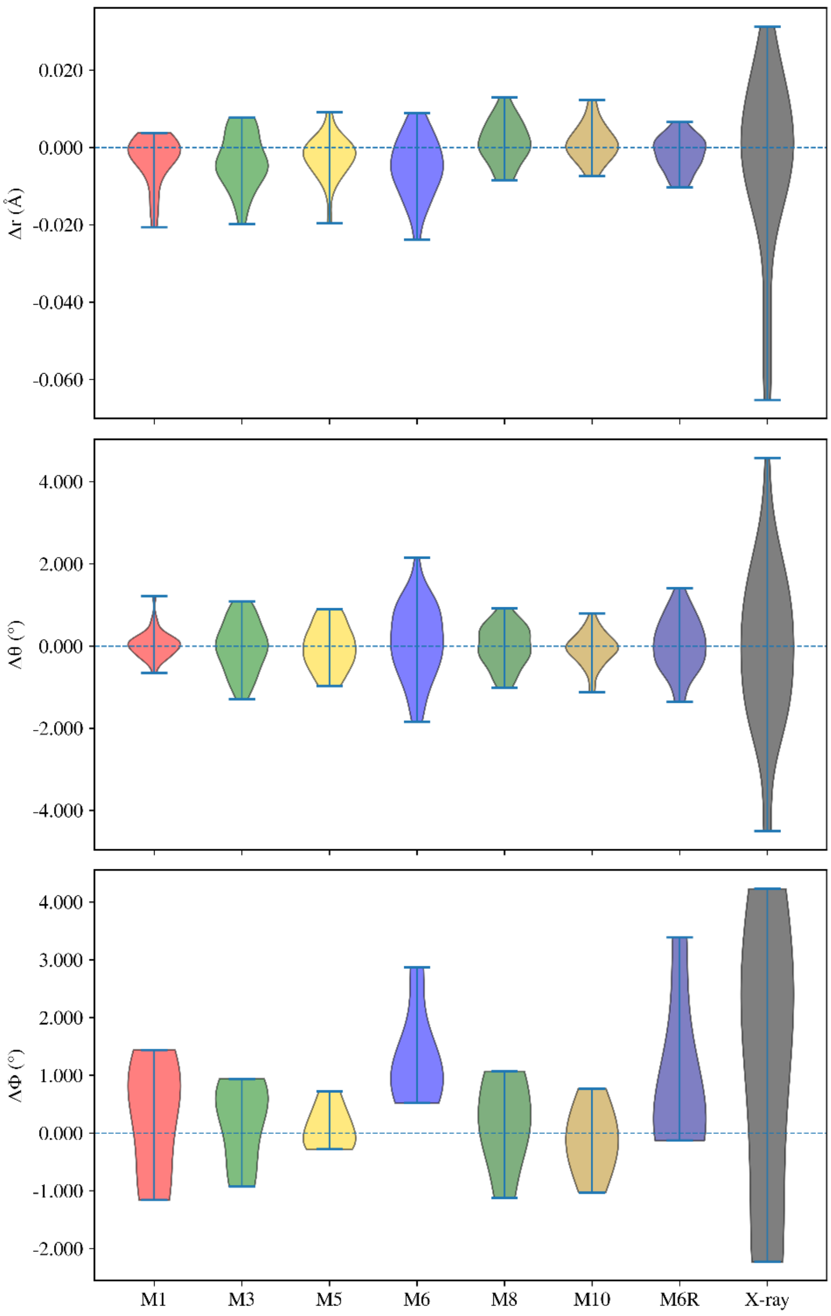

Supplementary Figure 87: Key coordinates of quantum refinement results of hispidulin. Deviation in the refined bond distances (Δr, n = 24), angles (Δθ, n = 35) and dihedrals (Δϕ, n = 6) of hispidulin (HUL) in Pim1 from various quantum refinement schemes (**M1-M10**) and X-ray structure which are compared to those obtained from the most reliable **M7** scheme. The solid line represents the upper and lower values.

(xiii) 4YHM (Anticoagulants with dabigatran)

Protein preparations:

Resolution: 2.16 Å

Ligand: 4CC (Dabigatran); $C_{25}H_{25}N_7O_3$

Residue flipped: Chain H: GLN1, GLN177, GLN198, ASN205

Protonation states (pH = 7.0):

Chain H: HID35, HID170, HID206

Chain L: HID31, GLH39, HID98, HID194, HID203

Optimized region: 4CC

High layer: 4CC

Medium layer: Chain H: H THR30, ASP31, TYR32, TYR33, HID35, GLU50, ASN52, PRO53, ARG54, GLY99, TYR103, TYR105, PHE106, WAT452, WAT482, WAT500, WAT549, WAT551, WAT567

ω_α = 0.82342

(a)

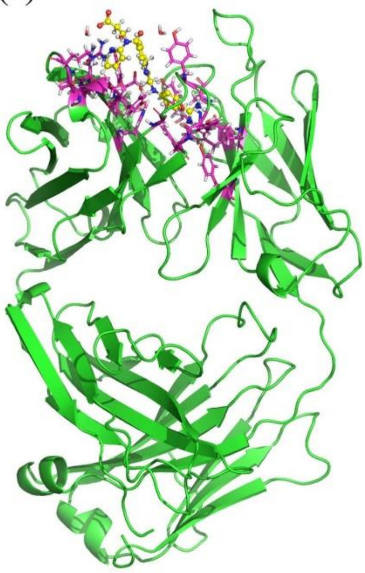

(b)

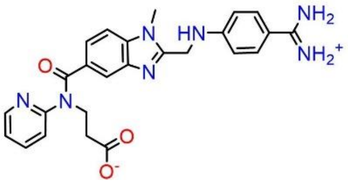

Supplementary Figure 88: Anticoagulants with dabigatran. (**a**) Crystal structure of anticoagulants with dabigatran (4CC). ONIOM layers by different colors: yellow: high layer; red: medium layer; green: low layer. Ligand dabigatran is presented in stick and balls. (**b**) Structure of dabigatran.

Quantum refined structural results:

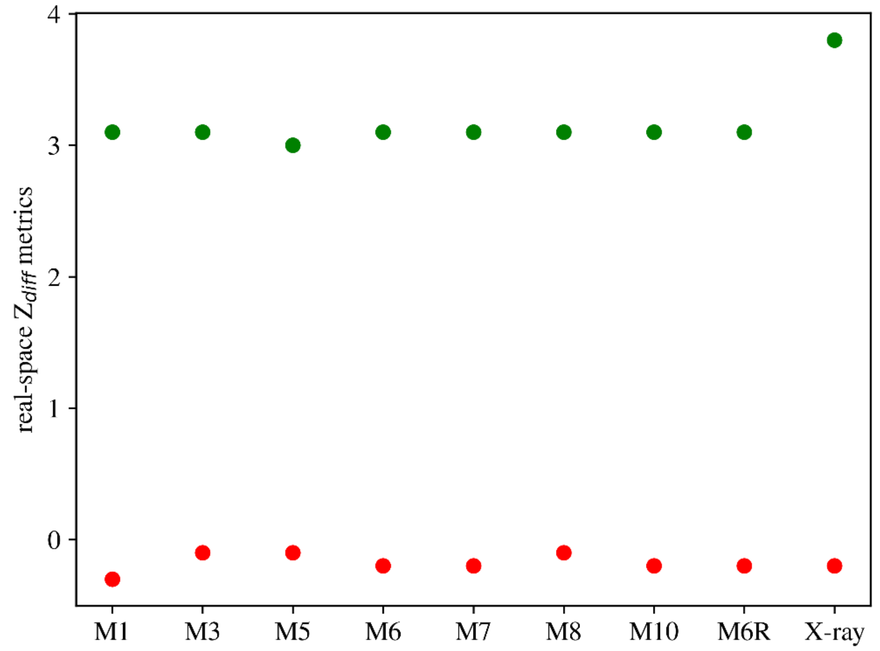

Supplementary Figure 89: Real-space Z-difference (RSZD) of dabigatran. RSZD+ (green) and RSZD- (red) scores of dabigatran (4CC) in anticoagulants from various quantum refinement schemes (**M1-M10**). Those results for X-ray were taken from the experimental structure without our further refinement.

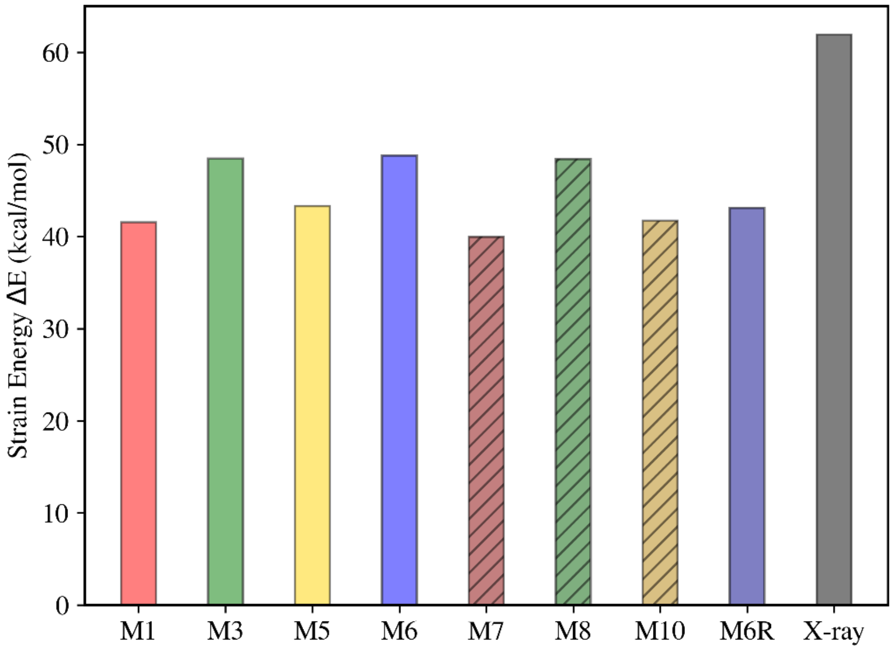

Supplementary Figure 90: Strain energy of dabigatran. Strain energy (ΔE, kcal·mol^{-1}) at ωB97X-D/6-31G(d) level for dabigatran (4CC) in anticoagulants determined by various quantum refinement schemes (**M1-M10**). Those results for X-ray were taken from the experimental structure without our further refinement.

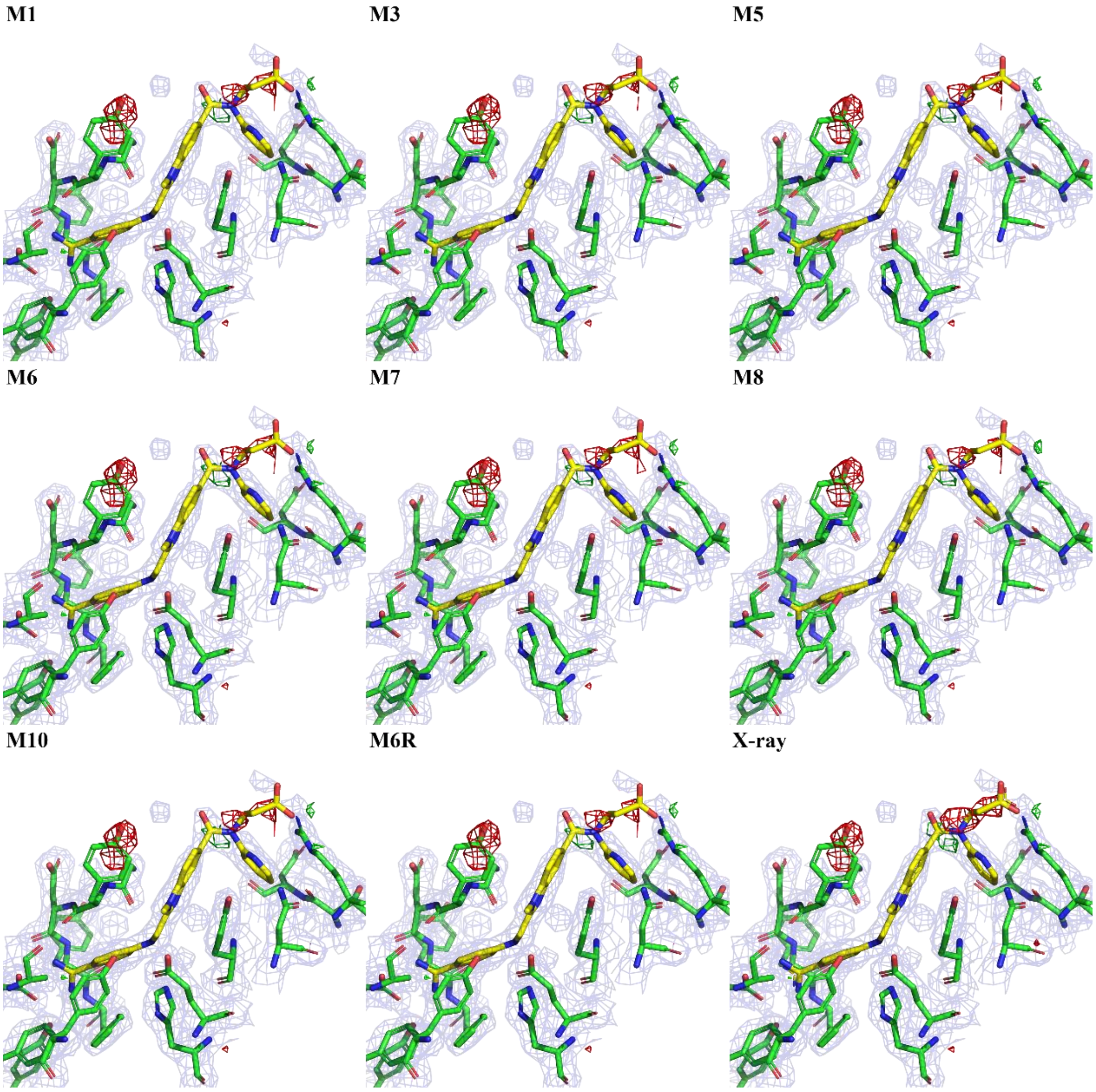

Supplementary Figure 91: Electron density maps of dabigatran. Structures for dabigatran (4CC) in anticoagulants from various quantum refinement schemes (**M1-M10**), including the electron density maps (2mFo-DFc maps, contoured at 1.0 σ (blue), mFo-DFc maps, contoured at +3.0 σ (green), and mFo-DFc maps, contoured at -3.0 σ (red)). Those results for X-ray were taken from the experimental structure without our further refinement.

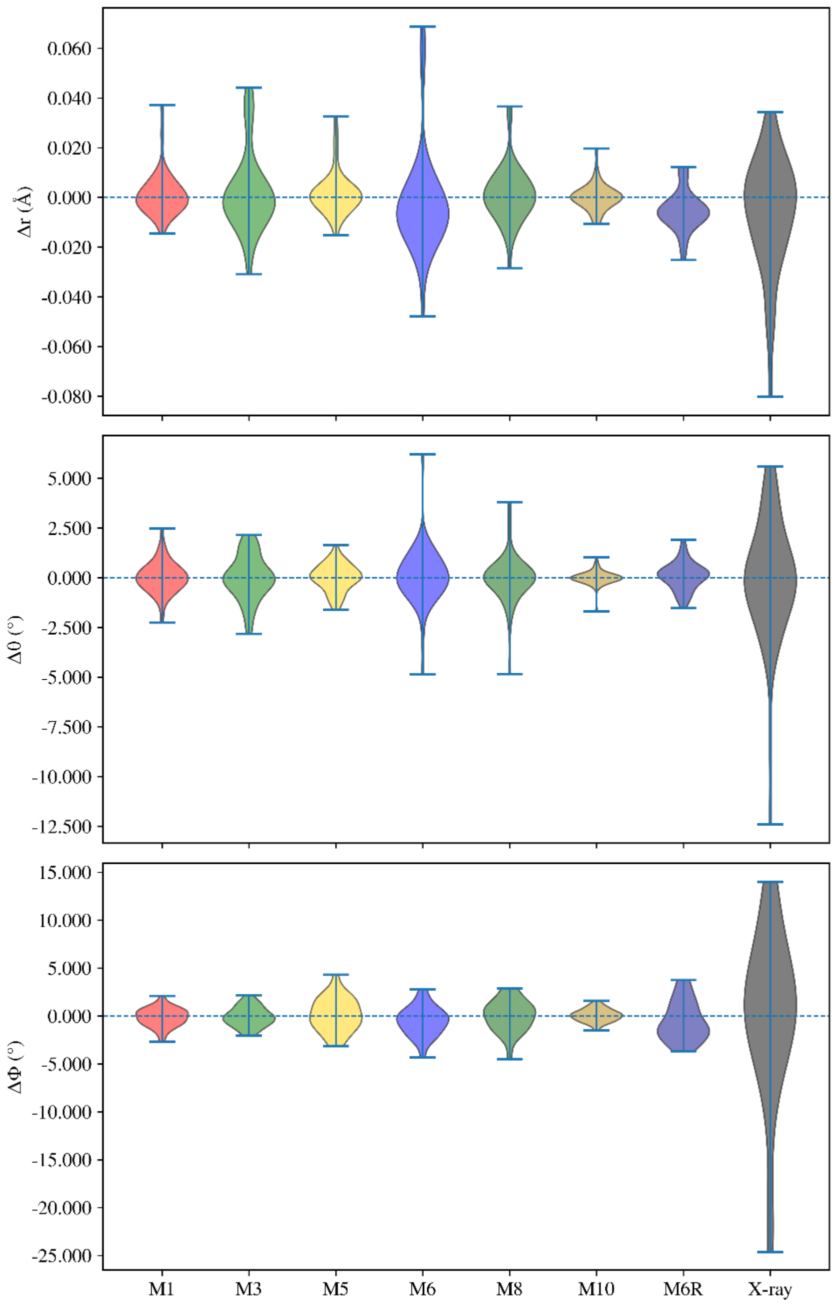

Supplementary Figure 92: Key coordinates of quantum refinement results of dabigatran. Deviation in the refined bond distances (Δr, n = 38), angles (Δθ, n = 53) and dihedrals (Δϕ, n = 26) of dabigatran (4CC) in anticoagulants from various quantum refinement schemes (**M1-M10**) and X-ray structure which are compared to those obtained from the most reliable **M7** scheme. The solid line represents the upper and lower values.

(xiv) 6V83 (beta-lactamase in complex with ceftazidime)

Protein preparations:

Resolution: 2.16 Å

Ligand: CAZ (Acylated ceftazidime); $C_{17}H_{19}N_5O_7S_2$

Residue flipped: GLN30

Protonation states (pH = 6.0):

LYN73, HIP112, HIP197, ASH246,

Optimized region: CAZ+SER70

High layer: CAZ+SER70

Medium layer: CYS69, SER70, LYN73, ASN104, SER130, ASN132, SER167, ASN170, THR235, GLY236, SER237, GLY238, GLY240, WAT404, WAT407, WAT436, WAT444, WAT460, WAT475, WAT535, WAT589

ω_α = 0.40324

(a)

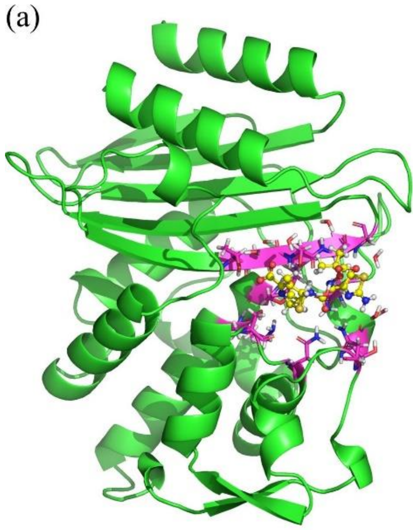

(b)

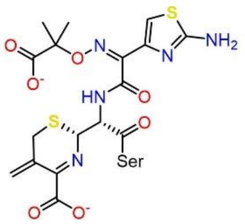

Supplementary Figure 93: Beta-lactamase in complex with ceftazidime. (**a**) Crystal structure of beta-lactamase in complex with ceftazidime (CAZ). ONIOM layers by different colors: yellow: high layer; red: medium layer; green: low layer. Ligand ceftazidime is presented in stick and balls. (**b**) Structure of ceftazidime.

Quantum refined structural results:

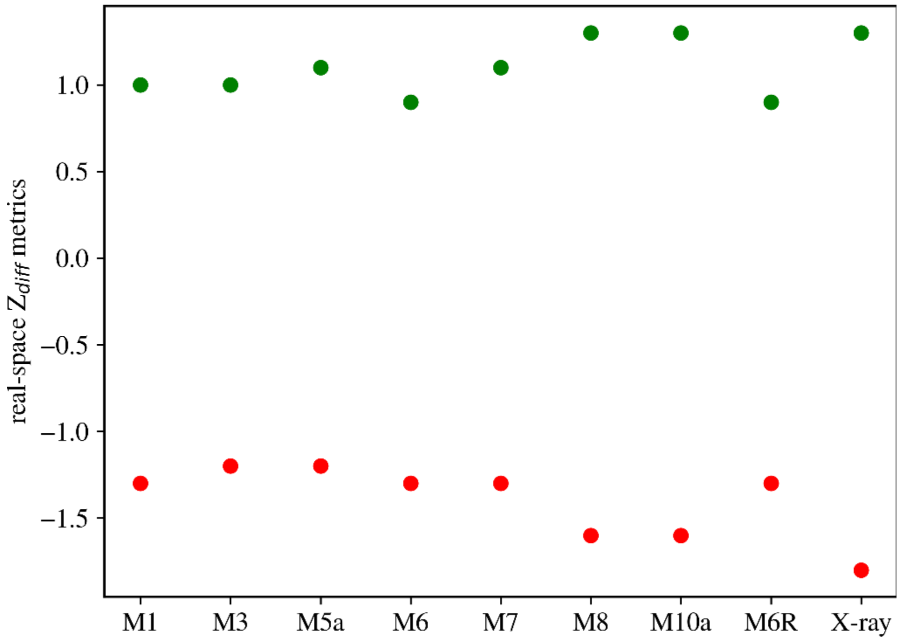

Supplementary Figure 94: Real-space Z-difference (RSZD) of ceftazidime. RSZD+ (green) and RSZD- (red) scores of ceftazidime (CAZ) in beta-lactamase from various quantum refinement schemes (**M1-M10**). Those results for X-ray were taken from the experimental structure without our further refinement.

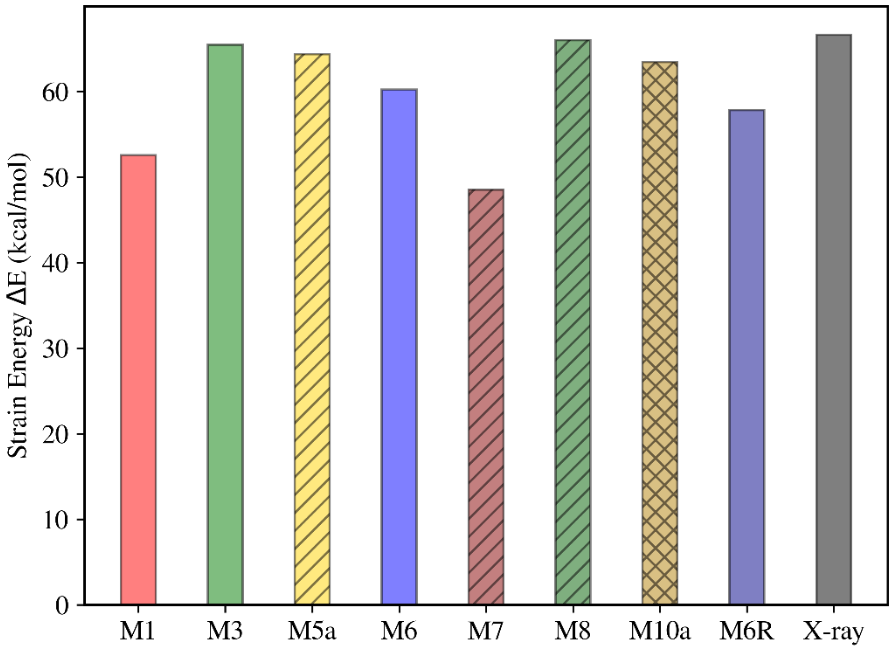

Supplementary Figure 95: Strain energy of ceftazidime. Strain energy (ΔE, kcal·mol^{-1}) at ωB97X-D/6-31G(d) level for ceftazidime (CAZ) in beta-lactamase determined by various quantum refinement schemes (**M1-M10**). Those results for X-ray were taken from the experimental structure without our further refinement.

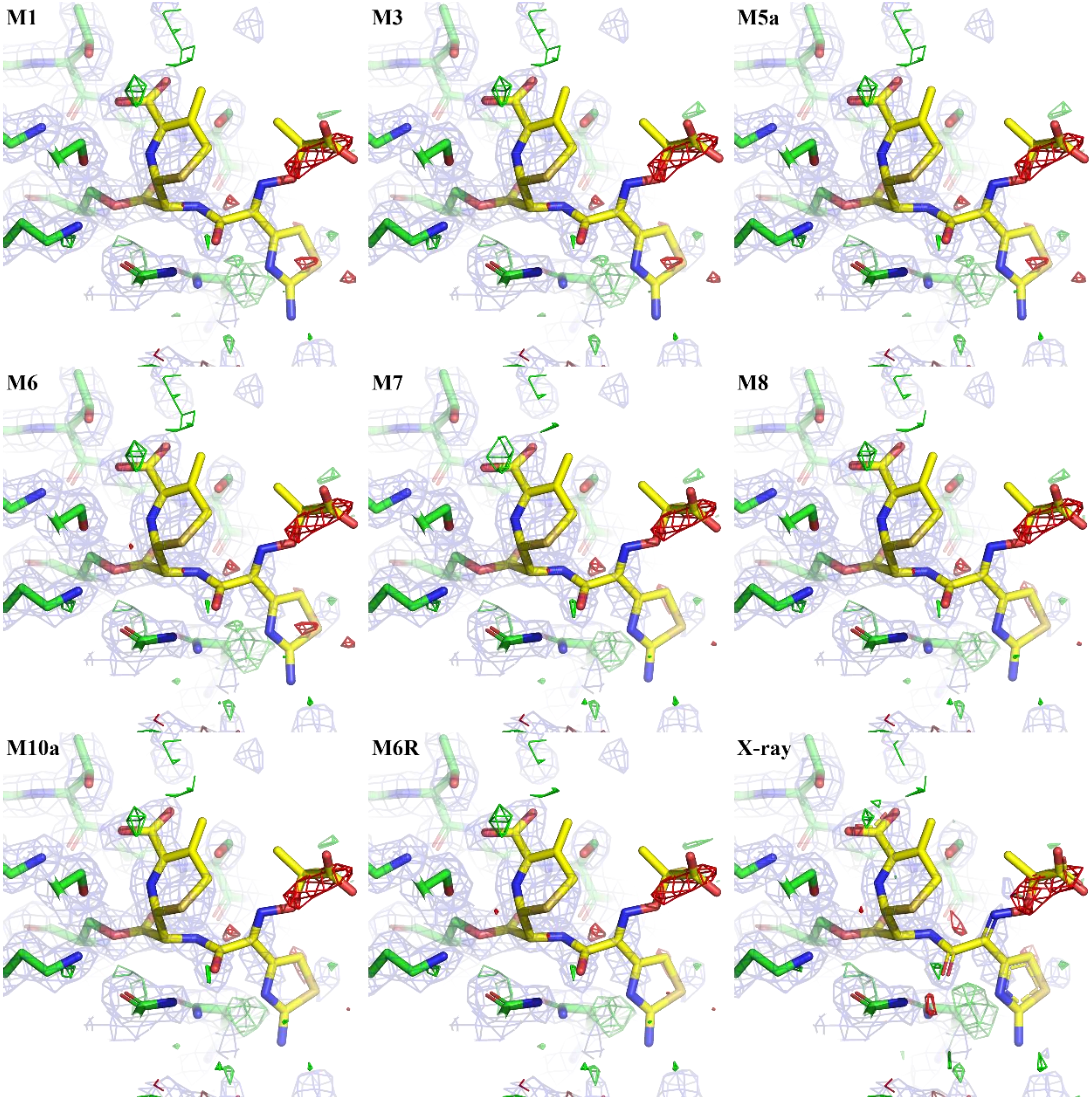

Supplementary Figure 96: Electron density maps of ceftazidime. Structures for ceftazidime (CAZ) in beta-lactamase from various quantum refinement schemes (**M1-M10**), including the electron density maps (2mFo-DFc maps, contoured at 1.0 σ (blue), mFo-DFc maps, contoured at +3.0 σ (green), and mFo-DFc maps, contoured at -3.0 σ (red)). Those results for X-ray were taken from the experimental structure without our further refinement.

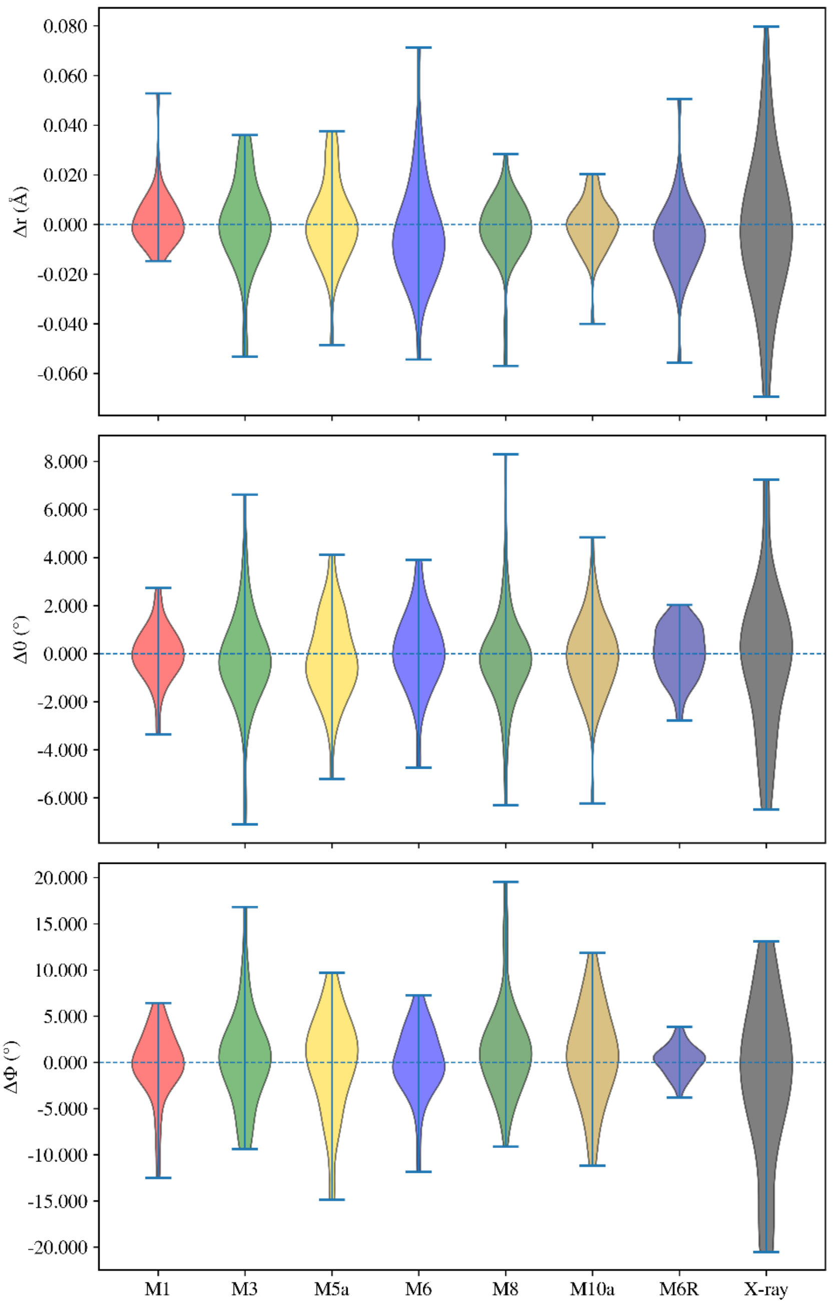

Supplementary Figure 97: Key coordinates of quantum refinement results of ceftazidime. Deviation in the refined bond distances (Δr, n = 38), angles (Δθ, n = 54) and dihedrals (Δϕ, n = 41) of ceftazidime (CAZ) in beta-lactamase from various quantum refinement schemes (**M1-M10**) and X-ray structure which are compared to those obtained from the most reliable **M7** scheme. The solid line represents the upper and lower values.

(xv) 1BR5 (Ricin a-chain complexes with neopterin)

Protein preparations:

Resolution: 2.50 Å

Ligand: NEO (D-threo-neopterin); $C_9H_{11}N_5O_4$

Residue flipped: ASN209, ANS222

Protonation states (pH = 5.5):

HID40, HID65, HID94, HID106

Optimized region: NEO

High layer: NEO

Medium layer: TYR80, VAL81, PHE93, GLY121, ASN122, TYR123, ILE172, GLU177, ARG180, GLU208, ASN209, WAT299, WAT334, WAT337, WAT343

ω_α = 1.4405

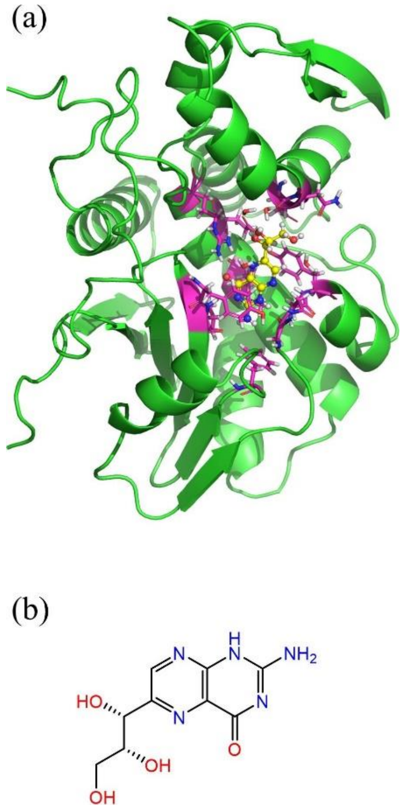

Supplementary Figure 98: Ricin a-chain complexes with neopterin. (**a**) Crystal structure of ricin a-chain complexes with neopterin (NEO). ONIOM layers by different colors: yellow: high layer; red: medium layer; green: low layer. Ligand neopterin is presented in stick and balls. (**b**) Structure of neopterin.

Quantum refined structural results:

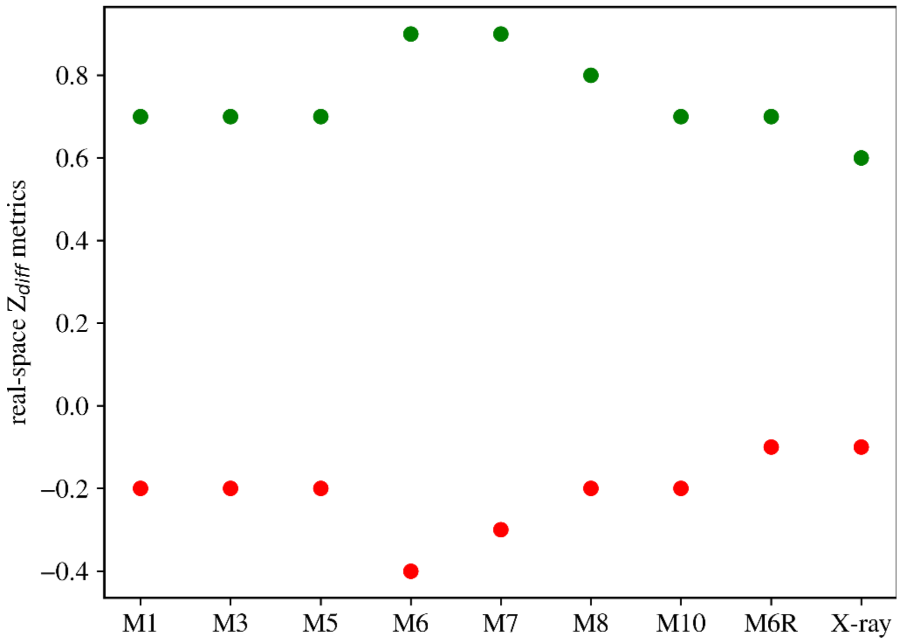

Supplementary Figure 99: Real-space Z-difference (RSZD) of neopterin. RSZD+ (green) and RSZD- (red) scores of neopterin (NEO) in ricin a-chain from various quantum refinement schemes (**M1-M10**). Those results for X-ray were taken from the experimental structure without our further refinement.

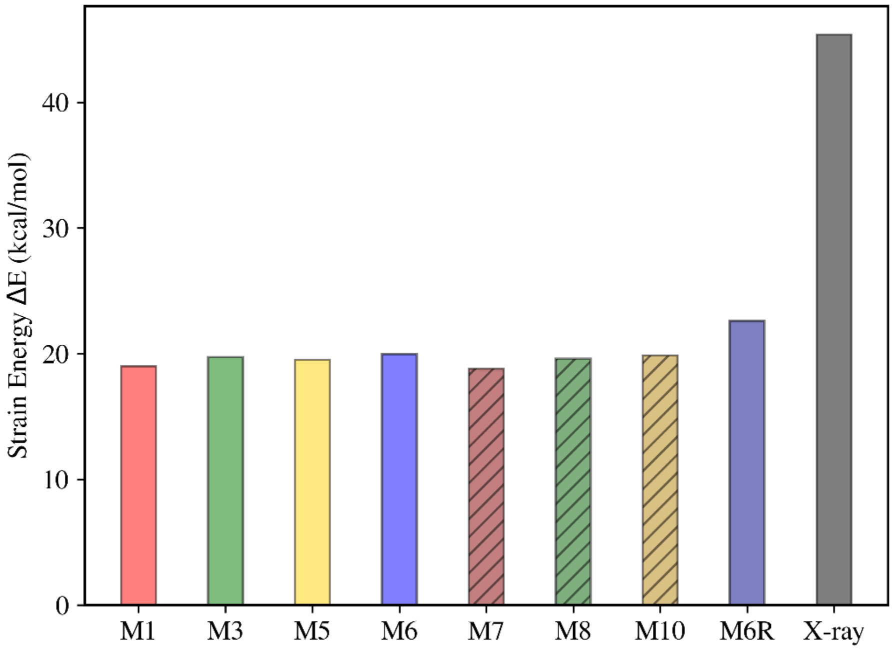

Supplementary Figure 100: Strain energy of neopterin. Strain energy (ΔE, kcal·mol^{-1}) at ωB97X-D/6-31G(d) level for neopterin (NEO) in ricin a-chain determined by various quantum refinement schemes (**M1-M10**). Those results for X-ray were taken from the experimental structure without our further refinement.

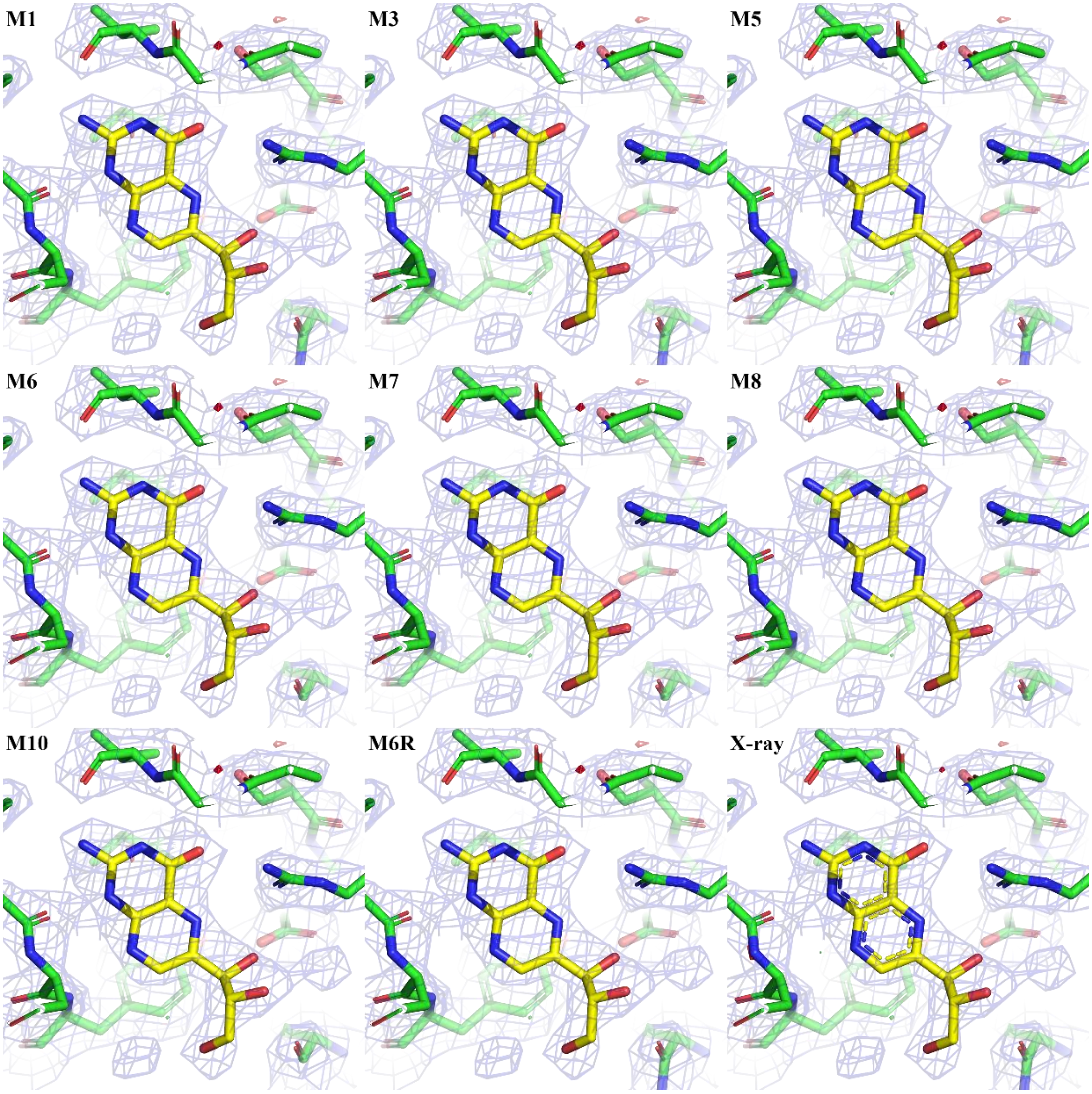

Supplementary Figure 101: Electron density maps of neopterin. Structures for neopterin (NEO) in ricin a-chain from various quantum refinement schemes (**M1**-**M10**), including the electron density maps (2mFo-DFc maps, contoured at 1.0 σ (blue), mFo-DFc maps, contoured at +3.0 σ (green), and mFo-DFc maps, contoured at -3.0 σ (red)). Those results for X-ray were taken from the experimental structure without our further refinement.

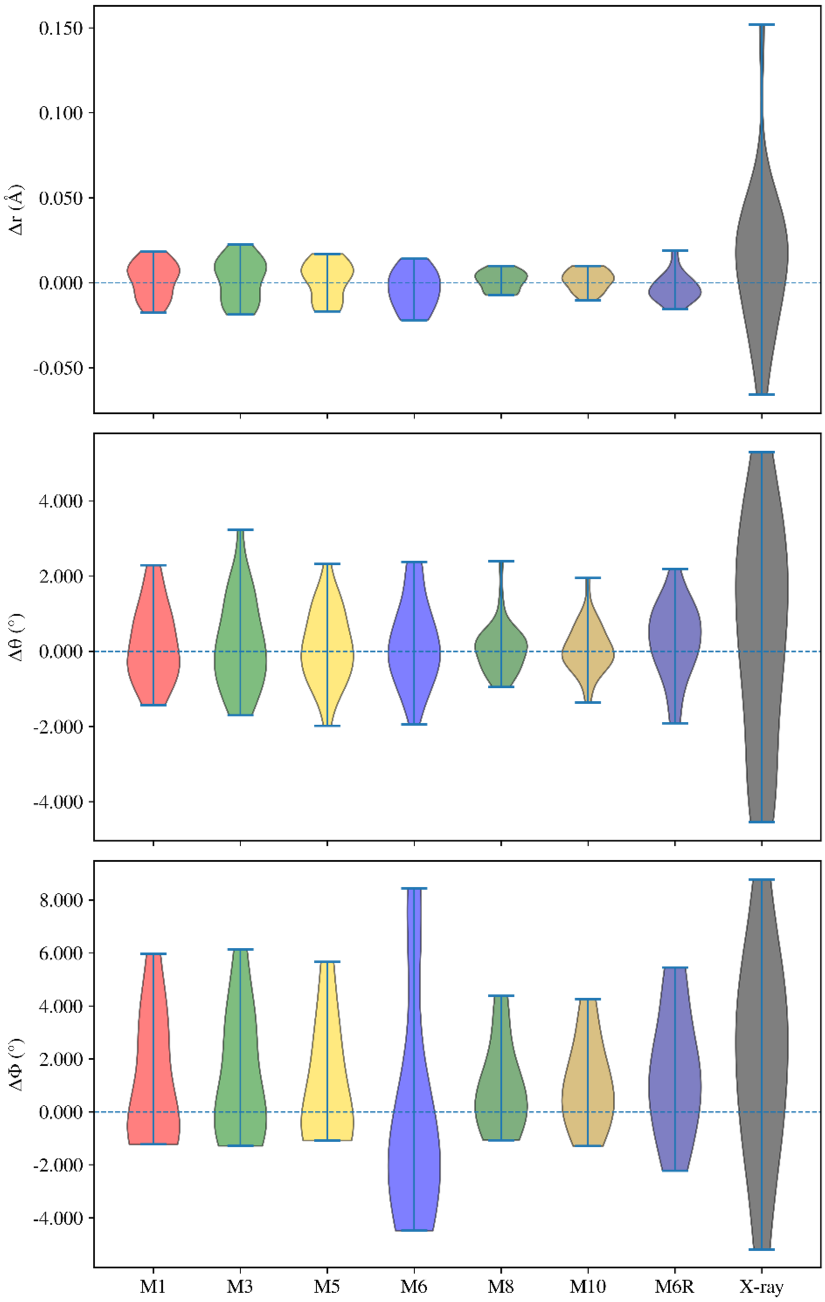

Supplementary Figure 102: Key coordinates of quantum refinement results of neopterin. Deviation in the refined bond distances (Δr, n = 19), angles (Δθ, n = 27) and dihedrals (Δϕ, n = 10) of neopterin (NEO) in ricin a-chain from various quantum refinement schemes (**M1-M10**) and X-ray structure which are compared to those obtained from the most reliable **M7** scheme. The solid line represents the upper and lower values.

(xvi) 1F9G (streptococcus pneumoniae hyaluronate lyase cocrystallized with ascorbic acid)

Protein preparations:

Resolution: 2.00 Å

Ligand: ASC (Ascorbic acid); $C_6H_8O_6$

Residue flipped:

ASN202, ASN254, ASN282, ASN303, ASN341, GLN392, GLN418, GLN424, HIS440, GLN498, ASN661, HIS667, GLN685, GLN698, GLN729, GLN759, ASN789, ASN820, GLN825, GLN832

Protonation states (pH = 6.0):

GLH247, HID277, HIP279, GLH295, HIP334, GLH369, GLH378, ASH398, HIP399, HID440, GLH454, GLH470, HIP472, GLH477, HID483, HIP578, ASH591, HID603, GLH622, HID667, HID786

Optimized region: ASC

High layer: ASC

Medium layer: ARG243, ASN290, ARG462, ARG466, ASN580

ω_α = 0.97178

(a)

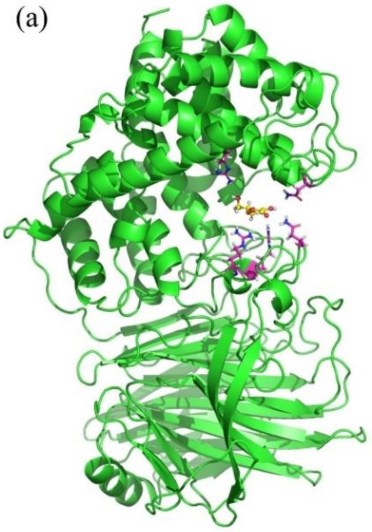

(b)

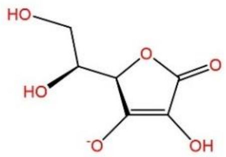

Supplementary Figure 103: Streptococcus pneumoniae hyaluronate lyase cocrystallized with ascorbic acid. (**a**) Crystal structure of streptococcus pneumoniae hyaluronate lyase with ascorbic acid (ASC). ONIOM layers by different colors: yellow: high layer; red: medium layer; green: low layer. Ligand ascorbic acid is presented in stick and balls. (**b**) Structure of ascorbic acid.

Quantum refined structural results:

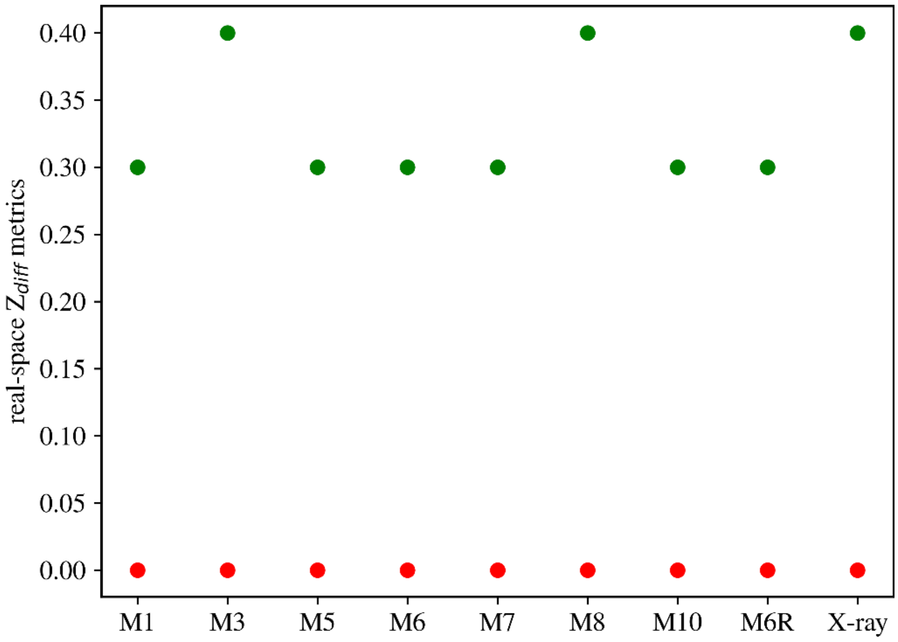

Supplementary Figure 104: Real-space Z-difference (RSZD) of ascorbic acid. RSZD+ (green) and RSZD- (red) scores of ascorbic acid (ASC) in streptococcus pneumoniae hyaluronate lyase from various quantum refinement schemes (**M1-M10**). Those results for X-ray were taken from the experimental structure without our further refinement.

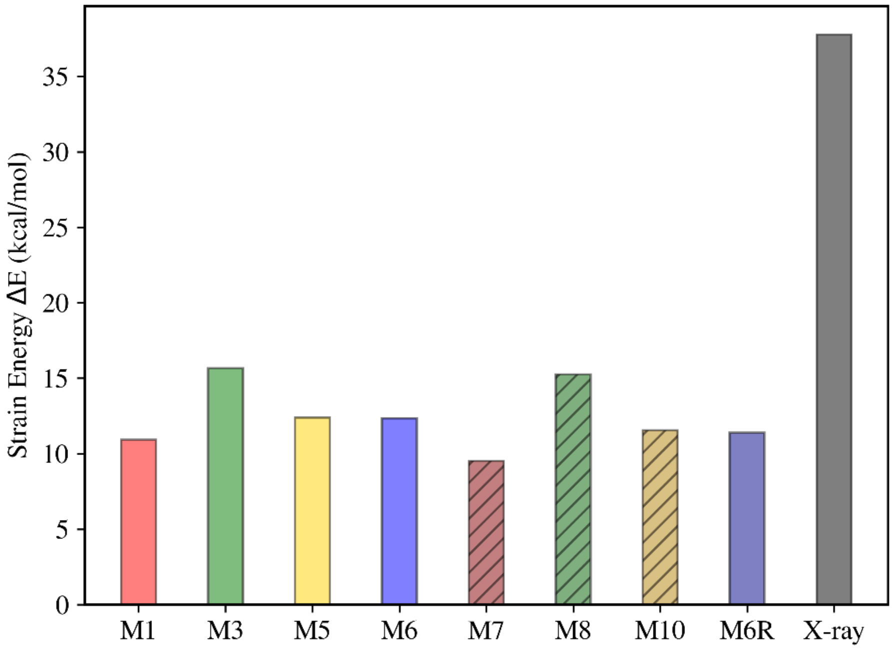

Supplementary Figure 105: Strain energy of ascorbic acid. Strain energy (ΔE, kcal·mol^{-1}) at ωB97X-D/6-31G(d) level for ascorbic acid (ASC) in streptococcus pneumoniae hyaluronate lyase determined by various quantum refinement schemes (**M1-M10**). Those results for X-ray were taken from the experimental structure without our further refinement.

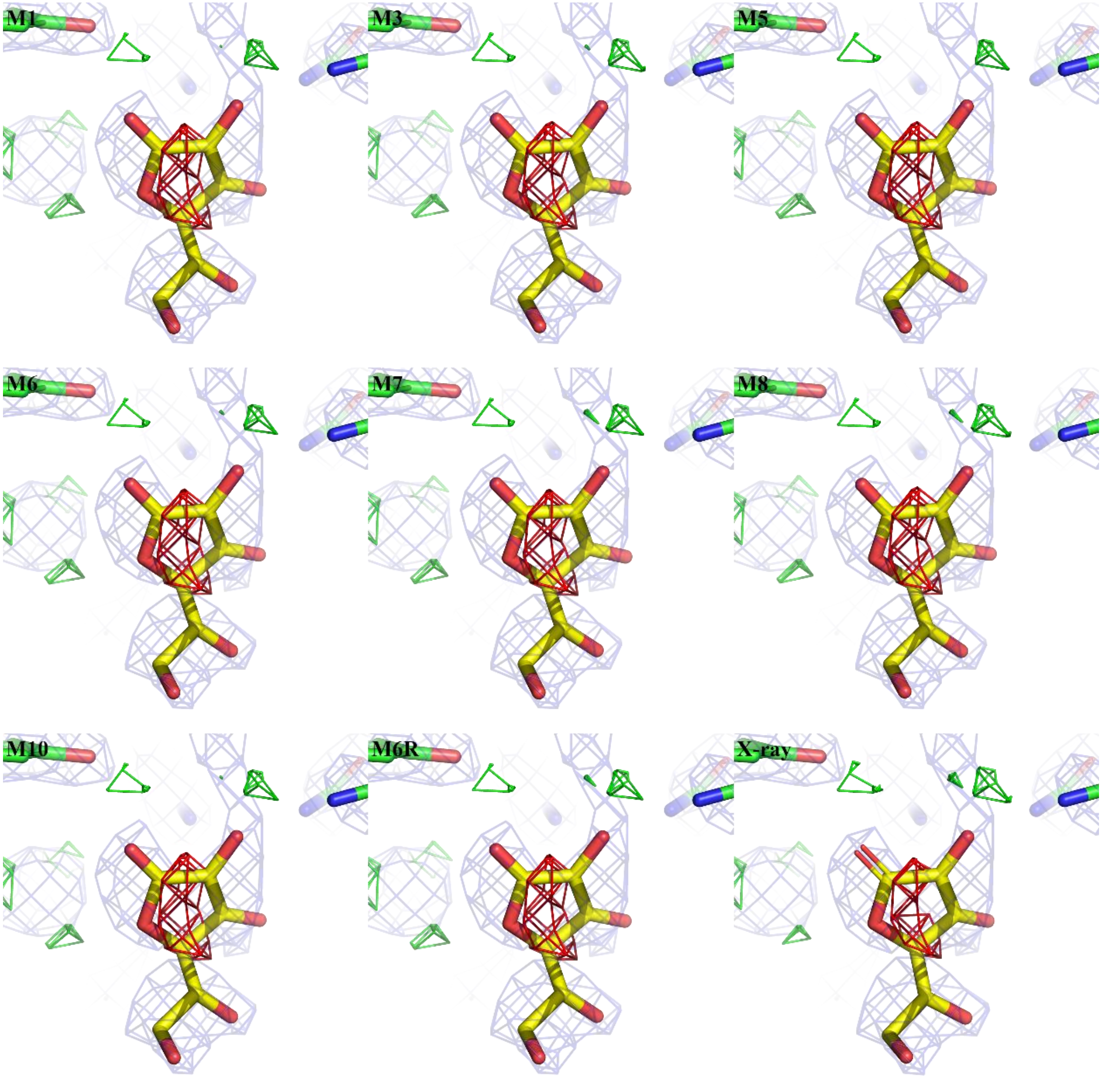

Supplementary Figure 106: Electron density maps of ascorbic acid. Structures for ascorbic acid (ASC) in streptococcus pneumoniae hyaluronate lyase from various quantum refinement schemes (**M1-M10**), including the electron density maps (2mFo-DFc maps, contoured at 1.0 σ (blue), mFo-DFc maps, contoured at +3.0 σ (green), and mFo-DFc maps, contoured at -3.0 σ (red)). Those results for X-ray were taken from the experimental structure without our further refinement.

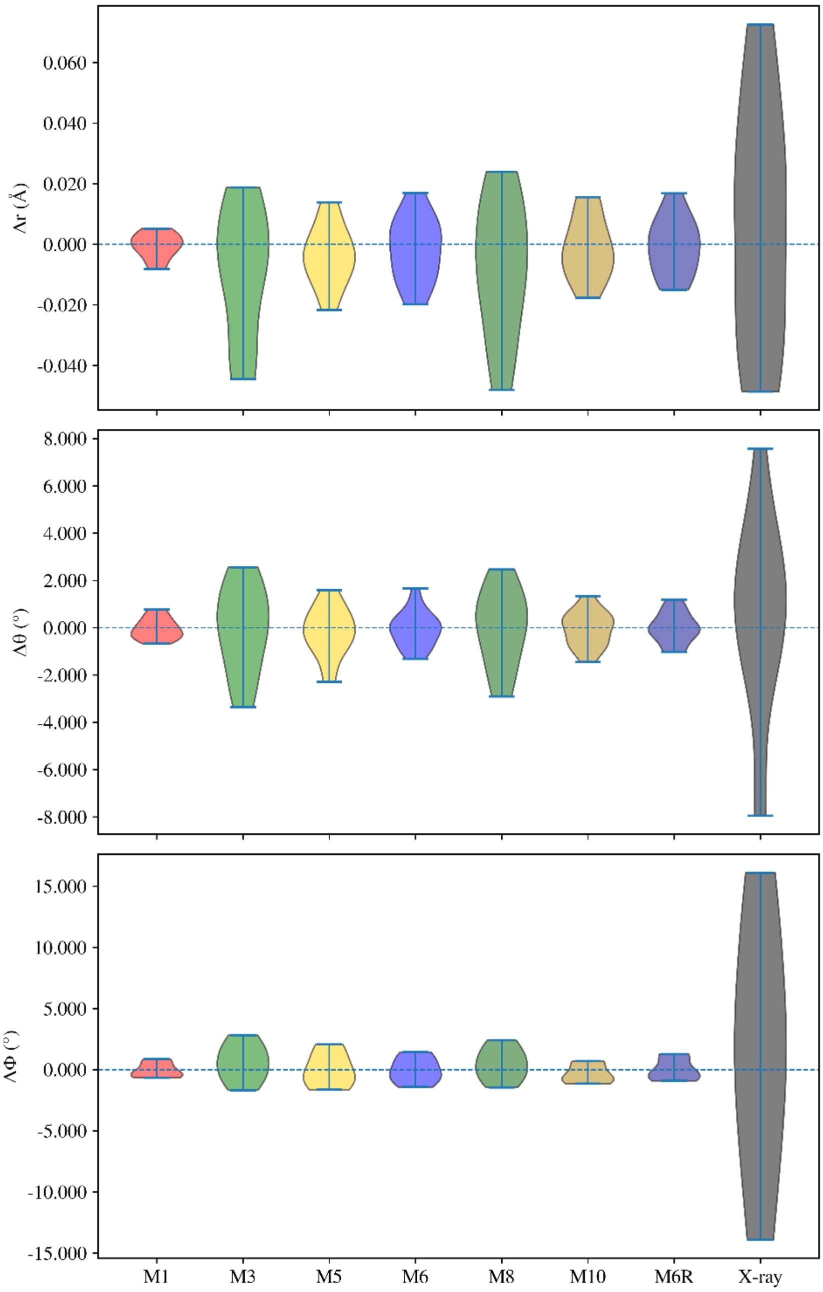

Supplementary Figure 107: Key coordinates of quantum refinement results of ascorbic acid. Deviation in the refined bond distances (Δr, n = 12), angles (Δθ, n = 17) and dihedrals (Δϕ, n = 6) of ascorbic acid (ASC) in streptococcus pneumoniae hyaluronate lyase from various quantum refinement schemes (**M1-M10**) and X-ray structure which are compared to those obtained from the most reliable **M7** scheme. The solid line represents the upper and lower values.

(xvii) 1LF3 (Plasmepsin II in complex with the inhibitor EH58)

Protein preparations:

Resolution: 2.70 Å

Ligand: EH5 (Inhibitor EH58); $C_{46}H_{45}N_3O_{10}$

Residue flipped: ASN3, ANS25, ASN39, GLN146, ASN151, ASN188, ASN317, ASN328

Protonation states (pH = 5.5):

ASH4, ASH10, ASH19, GLH21, ASH34, HIP56, GLH67, GLH104, GLH142, HIP161, HIP164, GLH179, GLH185, HIP189, HIP200, GLH207, ASH214, GLH262, GLH271, HIP276, GLH278, ASH303, HIP318,

Optimized region: EH5

High layer: EH5

Medium layer: MET15, ILE32, ASH34, GLY36, TYR77, VAL78, SER79, PHE111, THR114, PHE120, LEU131, TYR192, ASH214, GLY216, THR217, SER218, ILE290, LEU292, PHE294, ILE300

ω_α = 1.9853

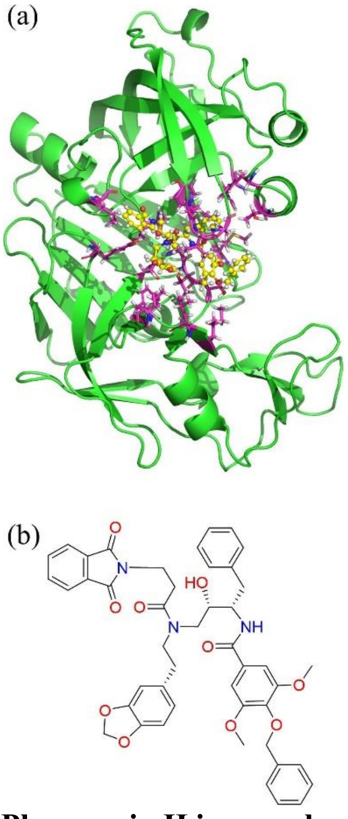

Supplementary Figure 108: Plasmepsin II in complex with the inhibitor EH58. (**a**) Crystal structure of Plasmepsin II in complex with the inhibitor EH58 (EH5). ONIOM layers by different colors: yellow: high layer; red: medium layer; green: low layer. Inhibitor EH58 is presented in stick and balls. (**b**) Structure of inhibitor EH58.

Quantum refined structural results:

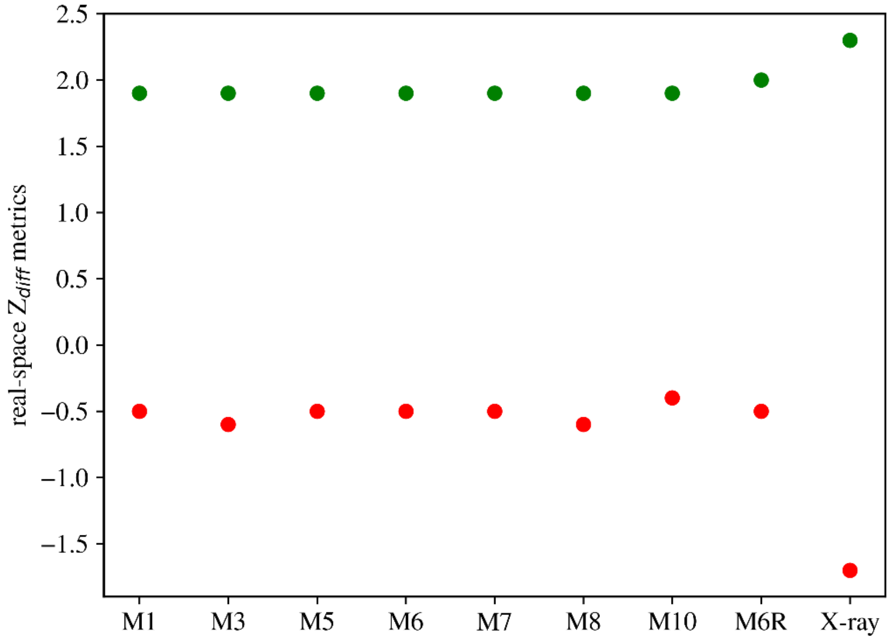

Supplementary Figure 109: Real-space Z-difference (RSZD) of inhibitor EH58. RSZD+ (green) and RSZD- (red) scores of inhibitor EH58 (EH5) in Plasmepsin II from various quantum refinement schemes (**M1-M10**). Those results for X-ray were taken from the experimental structure without our further refinement.

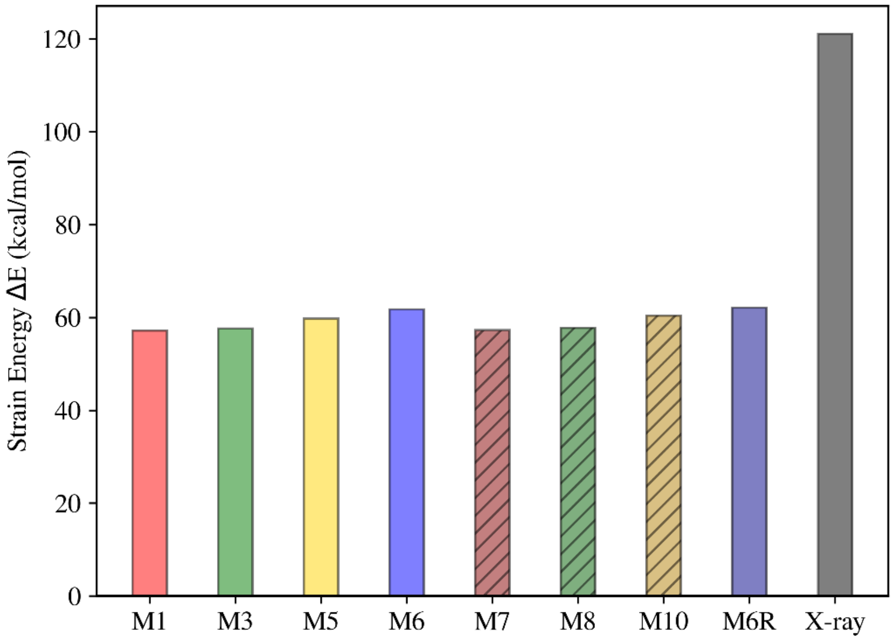

Supplementary Figure 110: Strain energy of inhibitor EH58. Strain energy (ΔE, kcal·mol^{-1}) at ωB97X-D/6-31G(d) level for inhibitor EH58 (EH5) in Plasmepsin II determined by various quantum refinement schemes (**M1-M10**). Those results for X-ray were taken from the experimental structure without our further refinement.

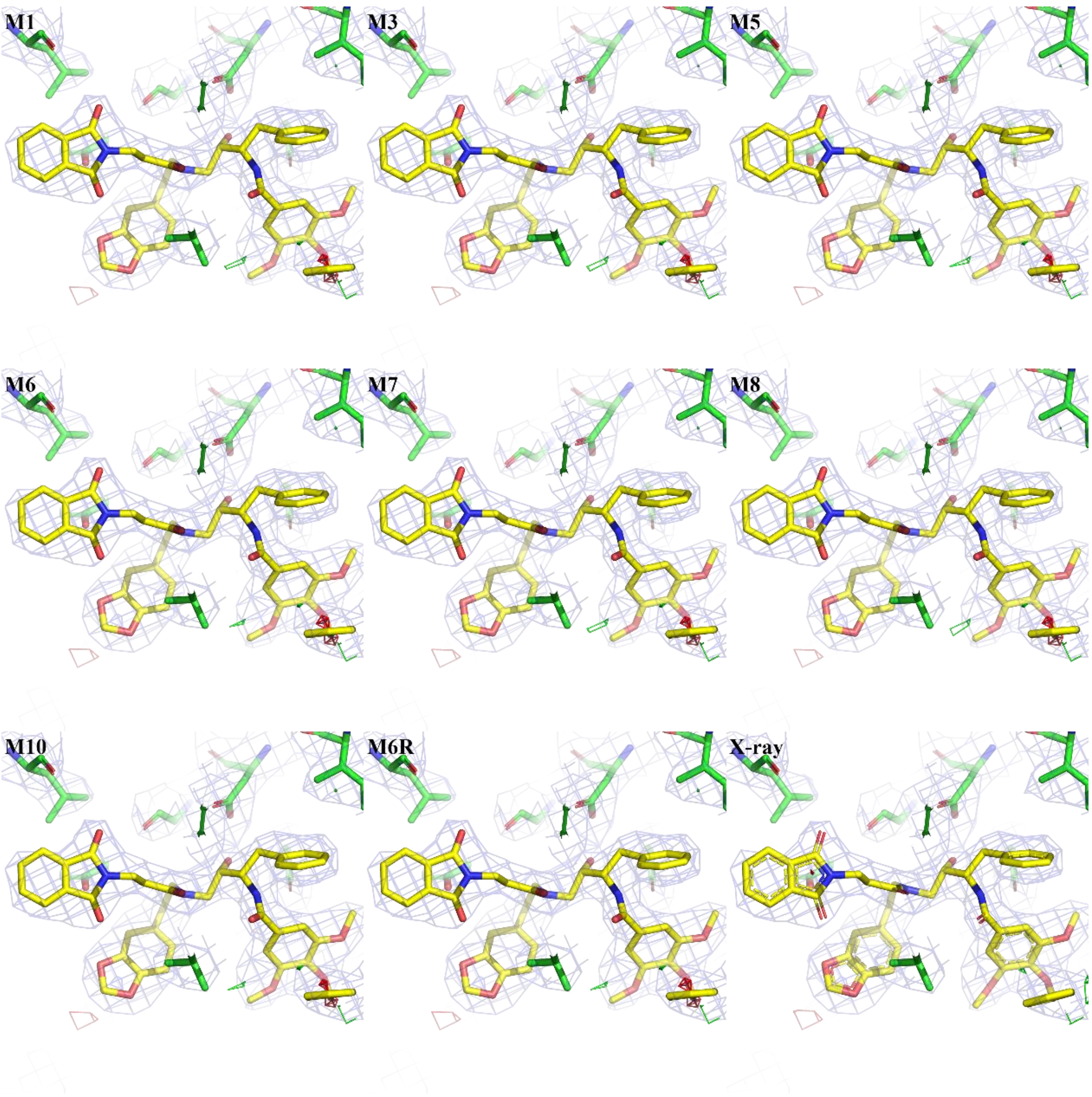

Supplementary Figure 111: Electron density maps of inhibitor EH58. Structures for inhibitor EH58 (EH5) in Plasmepsin II from various quantum refinement schemes (**M1**-**M10**), including the electron density maps (2mFo-DFc maps, contoured at 1.0 σ (blue), mFo-DFc maps, contoured at +3.0 σ (green), and mFo-DFc maps, contoured at -3.0 σ (red)). Those results for X-ray were taken from the experimental structure without our further refinement.

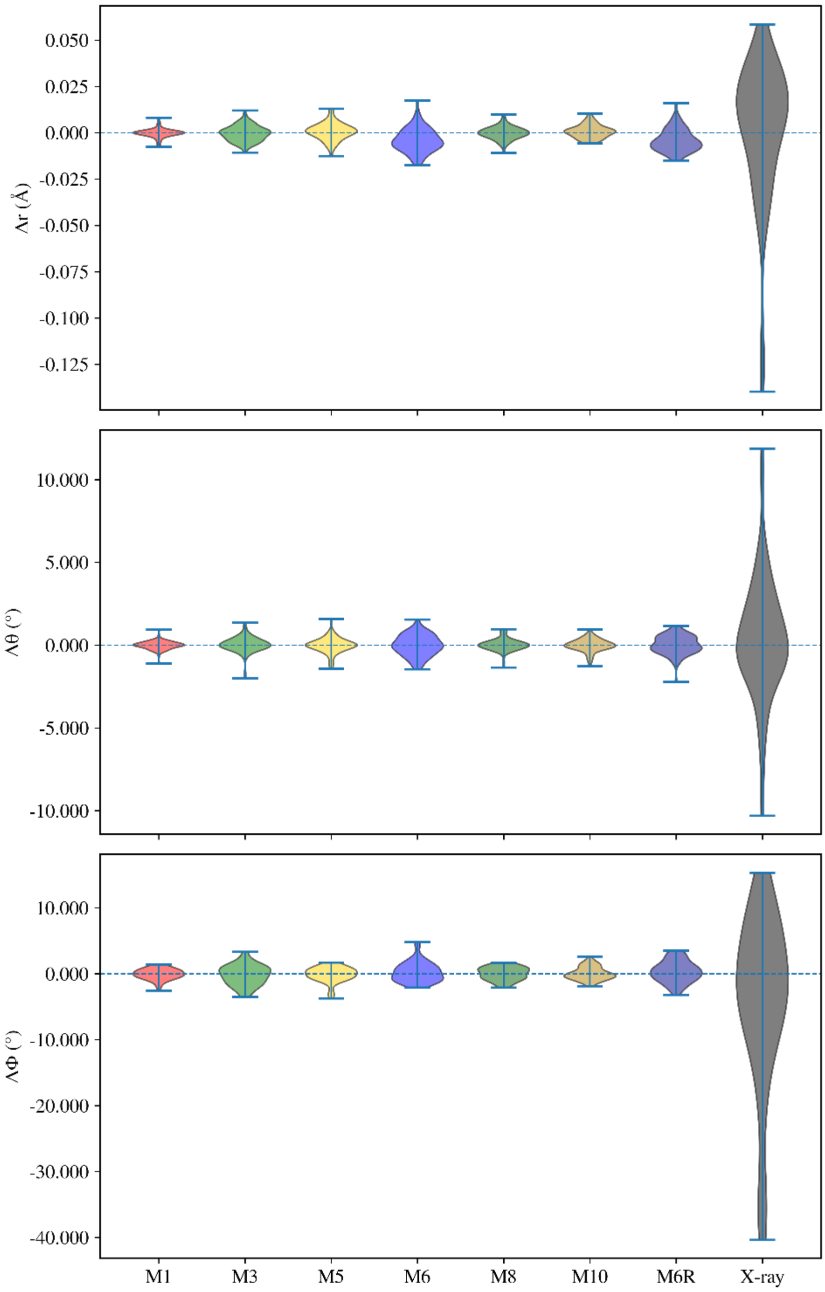

Supplementary Figure 112: Key coordinates of quantum refinement results of inhibitor EH58. Deviation in the refined bond distances (Δr, n = 65), angles (Δθ, n = 90) and dihedrals (Δϕ, n = 43) of inhibitor EH58 (EH5) in Plasmepsin II from various quantum refinement schemes (**M1-M10**) and X-ray structure which are compared to those obtained from the most reliable **M7** scheme. The solid line represents the upper and lower values.

(xviii) 4ZB8 (Glutathione transferase in complex with oxidized glutathione)

Protein preparations:

Resolution: 2.00 Å

Ligand: GDS (Glutathione disulfide); $C_{20}H_{32}N_6O_{12}S_2$

Residue flipped: GLN120

Protonation states (pH = 7.5):

HID3, HID12, HIE44, HID64, HID128, HID200

Optimized region: GDS

High layer: GDS

Medium layer: GLY15, PRO16, ASN17, PHE39, HIE44, ARG57, ILE58, PRO59, GLU73, SER74, GLY115, PRO116, GLY119, TRP175, WAT428, WAT437, WAT455, WAT473, WAT477, WAT488, WAT494, WAT513, WAT515, WAT536, WAT543, WAT571, WAT585

ω_α = 0.73089

(a)

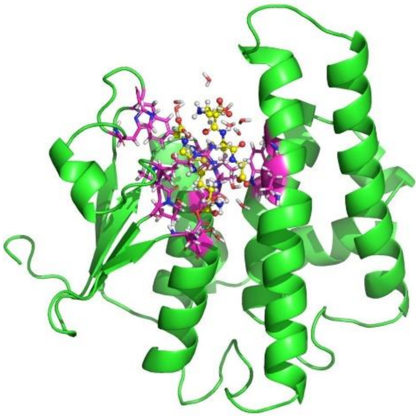

(b)

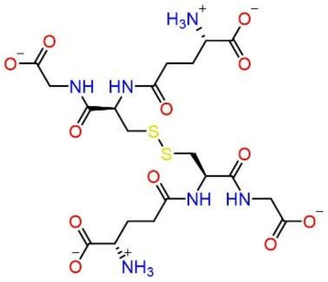

Supplementary Figure 113: Glutathione transferase in complex with oxidized glutathione. (**a**) Crystal structure of glutathione transferase in complex with oxidized glutathione (GDS). ONIOM layers by different colors: yellow: high layer; red: medium layer; green: low layer. Ligand neopterin is presented in stick and balls. (**b**) Structure of neopterin.

Quantum refined structural results:

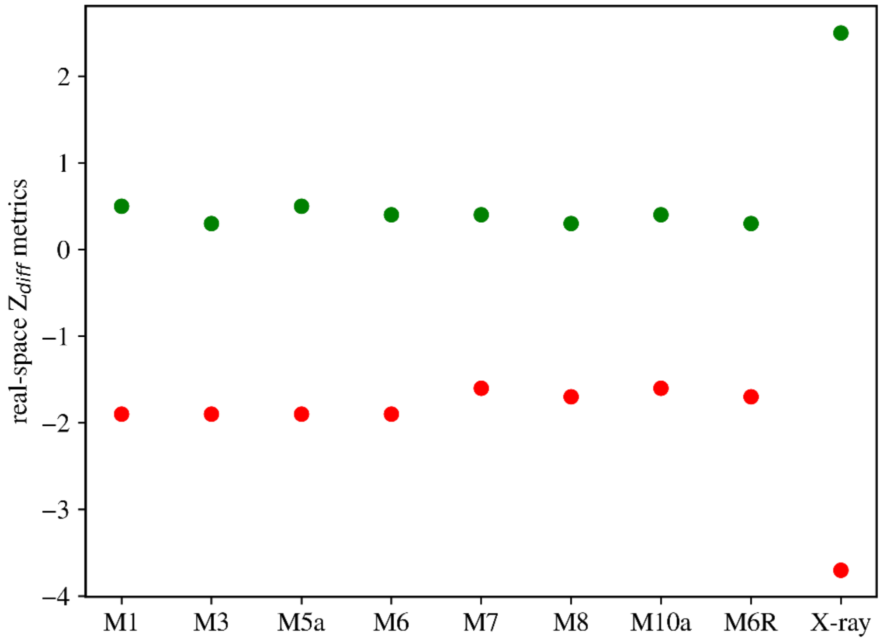

Supplementary Figure 114: Real-space Z-difference (RSZD) of oxidized glutathione. RSZD+ (green) and RSZD- (red) scores of oxidized glutathione (GDS). in glutathione transferase from various quantum refinement schemes (**M1-M10**). Those results for X-ray were taken from the experimental structure without our further refinement.

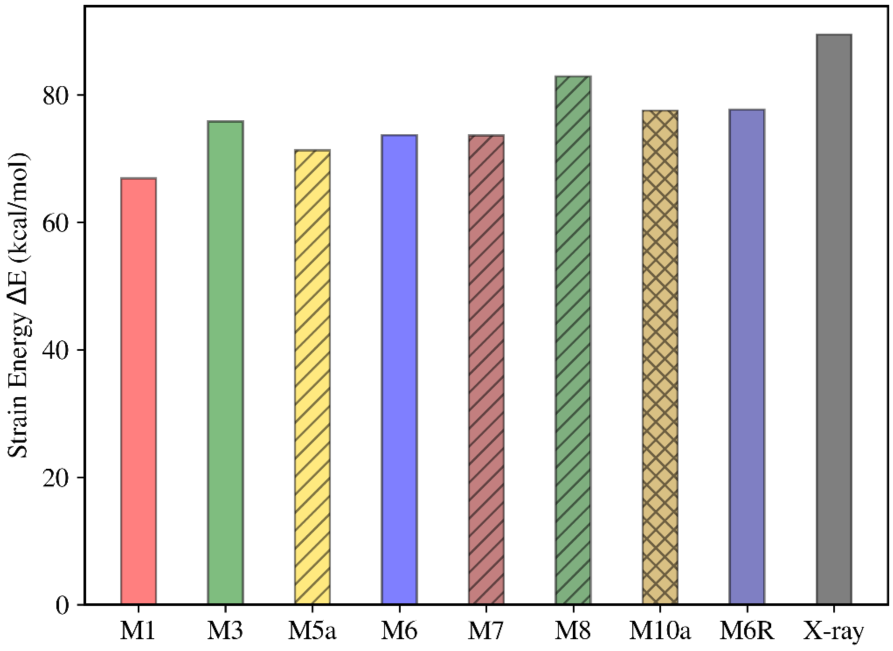

Supplementary Figure 115: Strain energy of oxidized glutathione. Strain energy (ΔE, kcal·mol^{-1}) at ωB97X-D/6-31G(d) level for oxidized glutathione (GDS). in glutathione transferase determined by various quantum refinement schemes (**M1-M10**). Those results for X-ray were taken from the experimental structure without our further refinement.

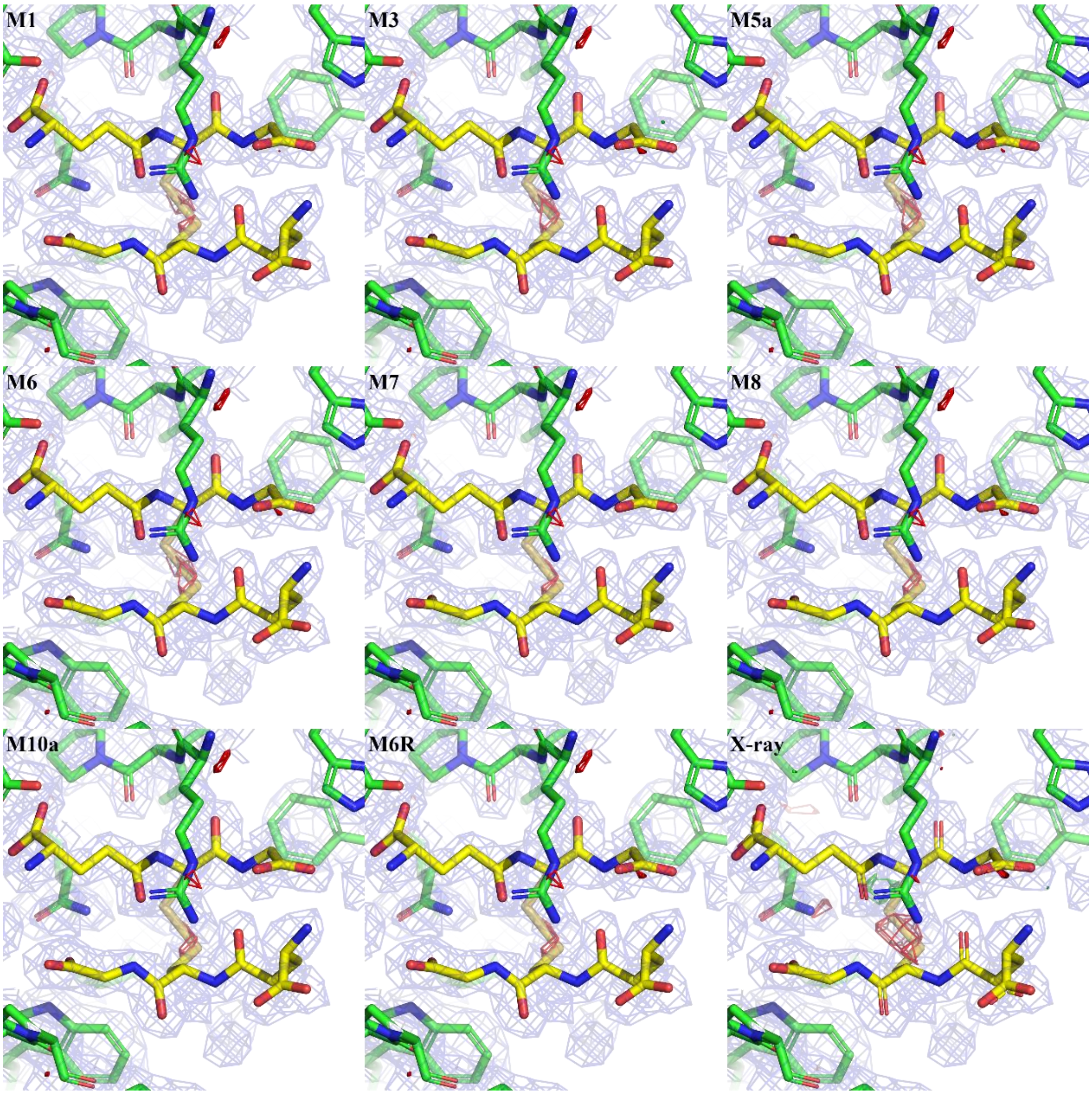

Supplementary Figure 116: Electron density maps of oxidized glutathione. Structures for oxidized glutathione (GDS). in glutathione transferase from various quantum refinement schemes (**M1**-**M10**), including the electron density maps (2mFo-DFc maps, contoured at 1.0 σ (blue), mFo-DFc maps, contoured at +3.0 σ (green), and mFo-DFc maps, contoured at -3.0 σ (red)). Those results for X-ray were taken from the experimental structure without our further refinement.

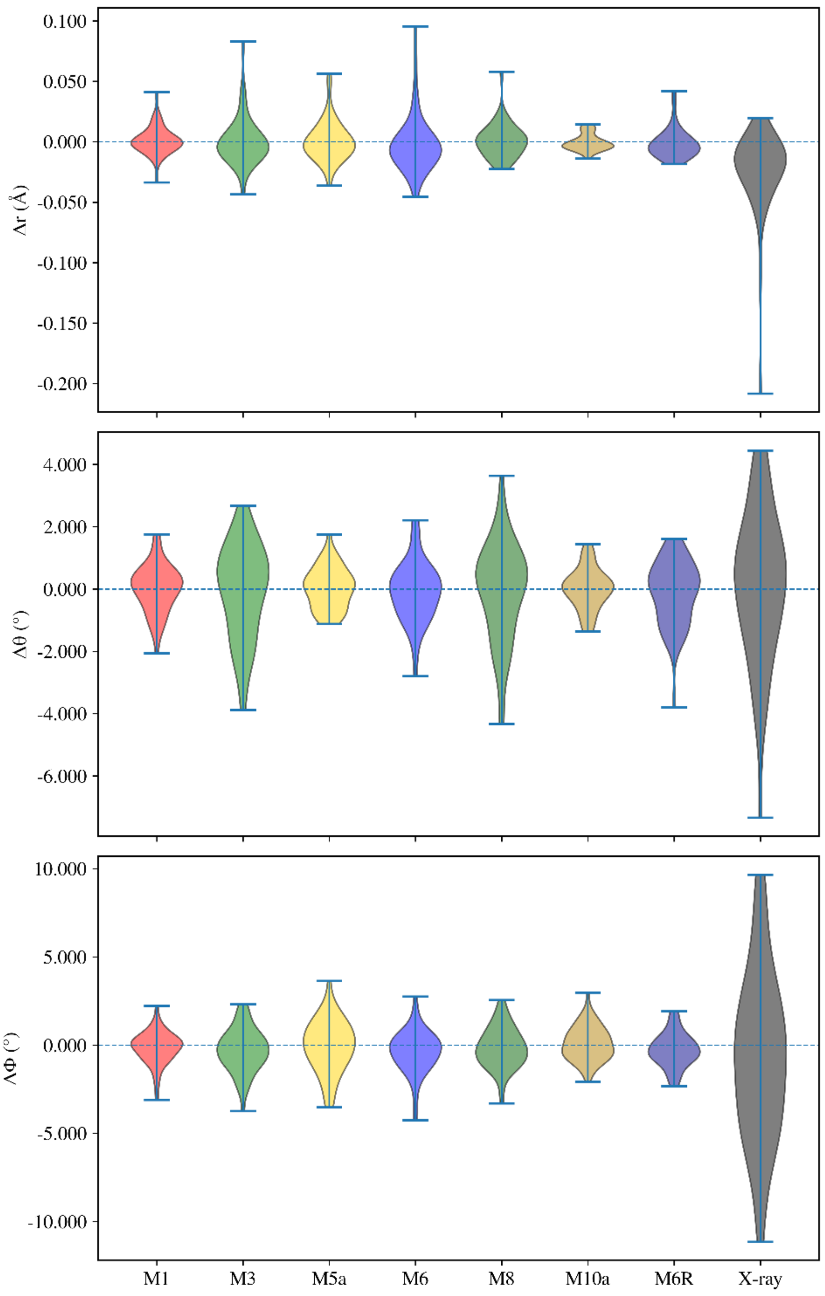

Supplementary Figure 117: Key coordinates of quantum refinement results of oxidized glutathione. Deviation in the refined bond distances (Δr, n = 39), angles (Δθ, n = 50) and dihedrals (Δϕ, n = 51) of oxidized glutathione (GDS). in glutathione transferase from various quantum refinement schemes (**M1-M10**) and X-ray structure which are compared to those obtained from the most reliable **M7** scheme. The solid line represents the upper and lower values.

(xix) **1P1O (GluR2 ligand-binding core (S1S2J) mutant L650T in complex with quisqualic acid)**

Protein preparations:

Resolution: 1.60 Å

Ligand: QUS (Quisqualic acid); $C_5H_7N_3O_5$

Residue flipped: GLN244

Protonation states (pH = 6.0):

GLH13, HIP23, HIP46

Optimized region: QUS

High layer: QUS

Medium layer: TYR61, PRO89, LEU90, THR91, ARG96, GLY141, SER142, THR143, LEU192, GLU193, MET196, TYR220, WAT436, WAT450

ω_α = 0.28902

(a)

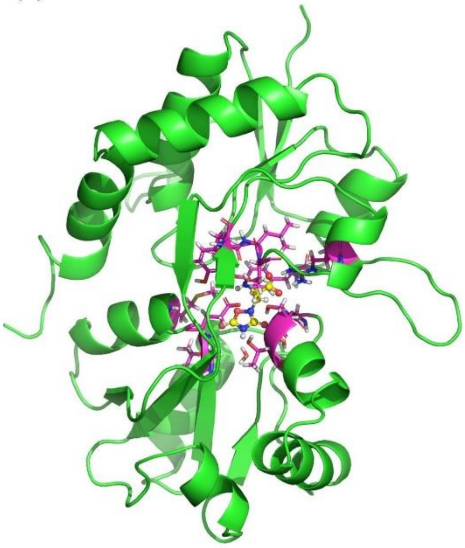

(b)

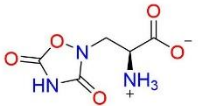

Supplementary Figure 118: GluR2 ligand-binding core (S1S2J) mutant L650T in complex with quisqualic acid. (**a**) Crystal structure of GluR2 ligand-binding core (S1S2J) mutant L650T in complex with quisqualic acid (QUS). ONIOM layers by different colors: yellow: high layer; red: medium layer; green: low layer. Ligand quisqualic acid is presented in stick and balls. (**b**) Structure of quisqualic acid.

Quantum refined structural results:

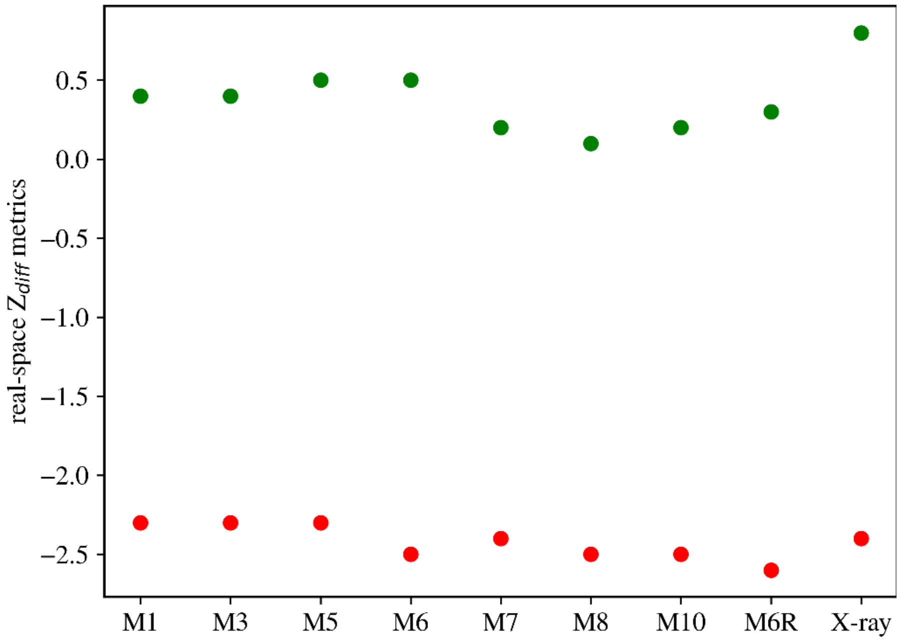

Supplementary Figure 119: Real-space Z-difference (RSZD) of quisqualic acid. RSZD+ (green) and RSZD- (red) scores of quisqualic acid (QUS) in GluR2 from various quantum refinement schemes (**M1-M10**). Those results for X-ray were taken from the experimental structure without our further refinement.

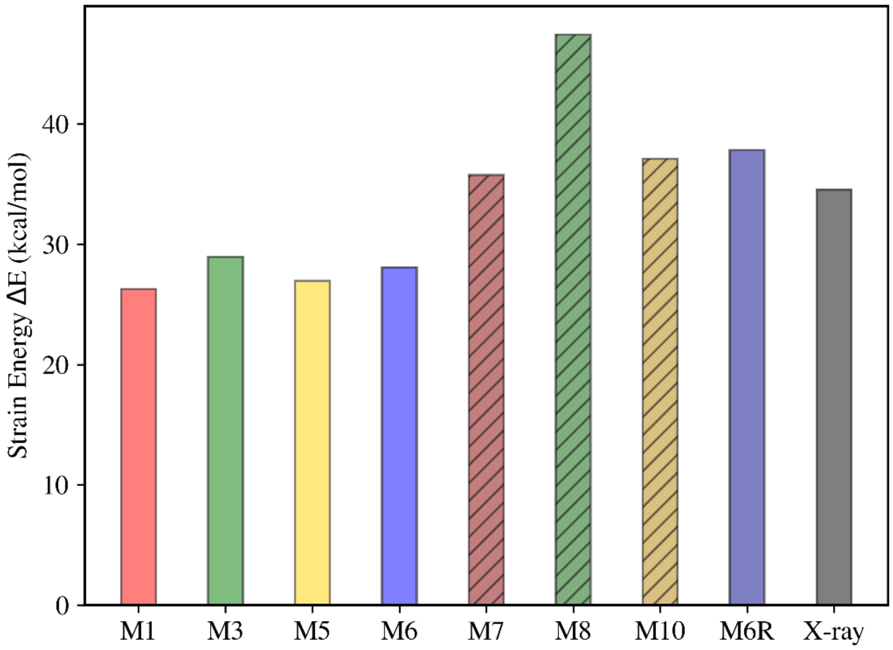

Supplementary Figure 120: Strain energy of quisqualic acid. Strain energy (ΔE, kcal·mol^{-1}) at ωB97X-D/6-31G(d) level for quisqualic acid (QUS) in GluR2 determined by various quantum refinement schemes (**M1-M10**). Those results for X-ray were taken from the experimental structure without our further refinement.

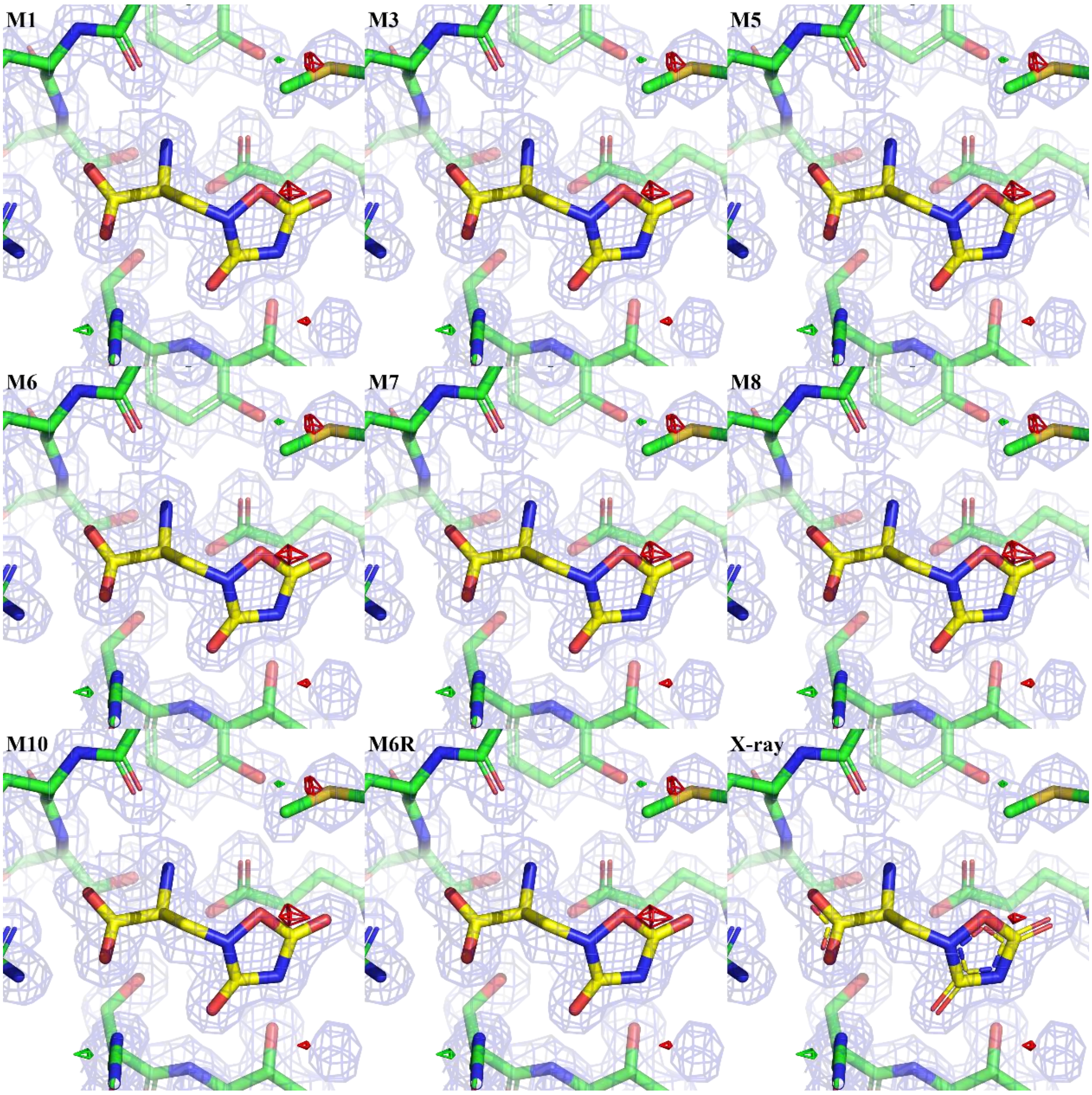

Supplementary Figure 121: Electron density maps of quisqualic acid. Structures for quisqualic acid (QUS) in GluR2 from various quantum refinement schemes (**M1-M10**), including the electron density maps (2mFo-DFc maps, contoured at 1.0 σ (blue), mFo-DFc maps, contoured at +3.0 σ (green), and mFo-DFc maps, contoured at -3.0 σ (red)). Those results for X-ray were taken from the experimental structure without our further refinement.

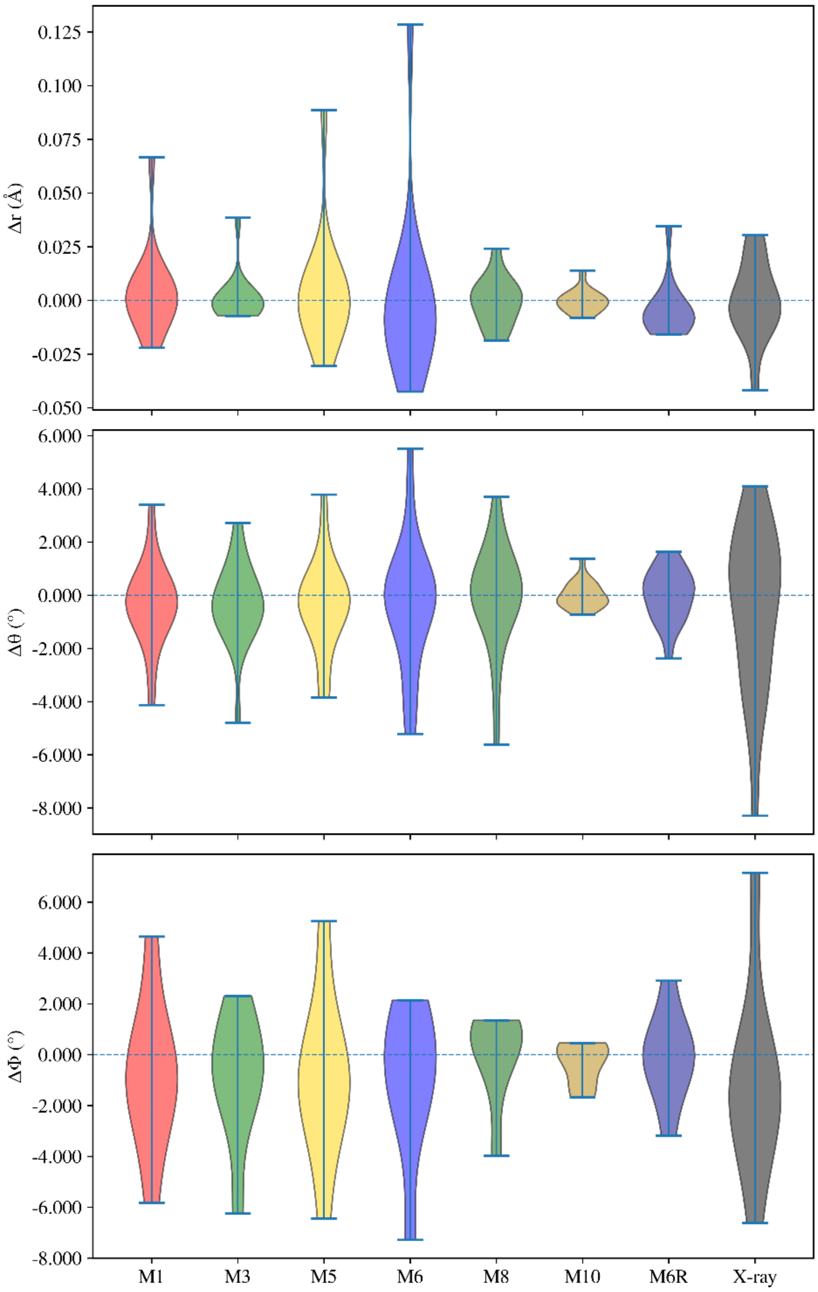

Supplementary Figure 122: Key coordinates of quantum refinement results of quisqualic acid. Deviation in the refined bond distances (Δr, n = 13), angles ($\Delta\theta$, n = 18) and dihedrals ($\Delta\phi$, n = 8) of quisqualic acid (QUS) in GluR2 from various quantum refinement schemes (**M1-M10**) and X-ray structure which are compared to those obtained from the most reliable **M7** scheme. The solid line represents the upper and lower values.

(xx) 1PF7 (Human PNP complexes with immucillin H)

Protein preparations:

Resolution: 2.50 Å

Ligand: IMH (Forodesine); $C_{11}H_{14}N_4O_4$

Residue flipped: GLN44, HIS86, GLN172, HIS257, GLN273

Protonation states (pH = 5.3):

HIP20, HID23, HIP64, HIP86, HID104, HID135, GLH201, HID230, HIP257, GLH259, GLH272

Optimized region: IMH

High layer: IMH

Medium layer: SER33, TYR88, ALA116, GLY118, VAL195, PHE200, GLH201, VAL217, GLY218, MET219, THR242, ASN243, HIP257, SO4293, WAT295, WAT311

ω_α = 0.85163

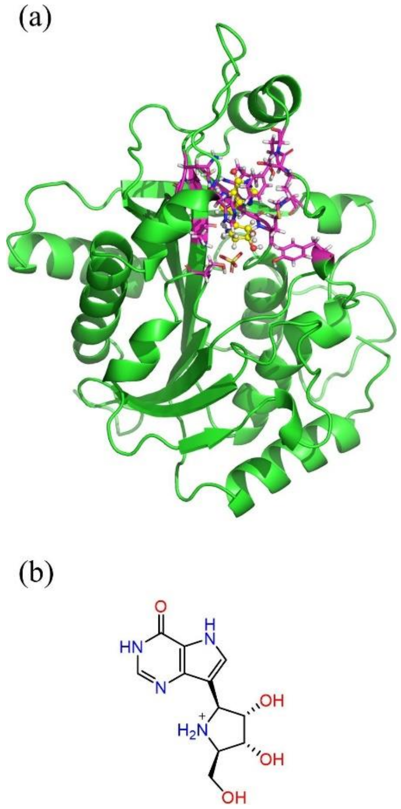

Supplementary Figure 123: Human PNP complexes with immucillin H. (**a**) Crystal structure of human PNP complexes with forodesine (IMH). ONIOM layers by different colors: yellow: high layer; red: medium layer; green: low layer. Ligand forodesine is presented in stick and balls. (**b**) Structure of forodesine.

Quantum refined structural results:

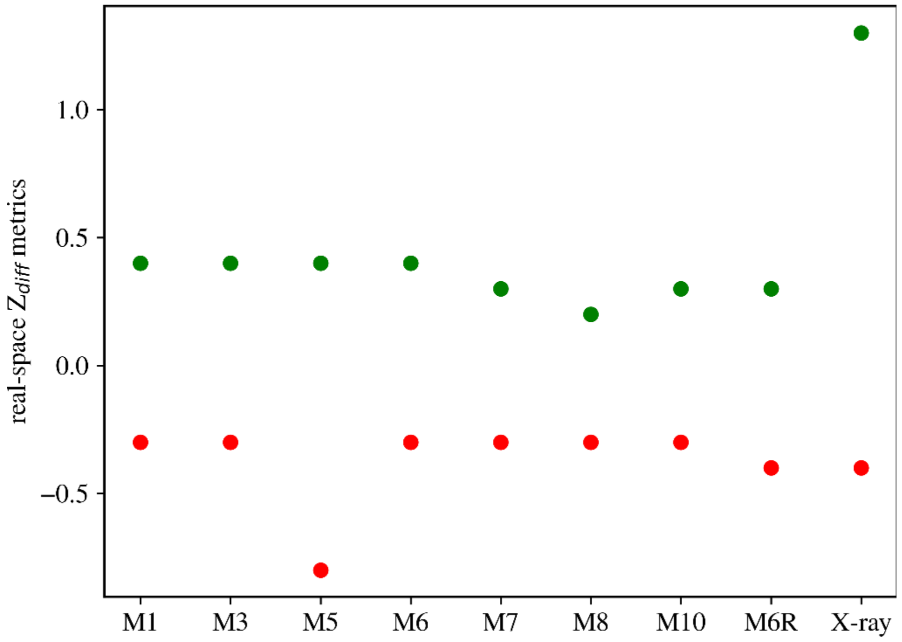

Supplementary Figure 124: Real-space Z-difference (RSZD) of forodesine. RSZD+ (green) and RSZD- (red) scores of forodesine (IMH) in human PNP from various quantum refinement schemes (**M1-M10**). Those results for X-ray were taken from the experimental structure without our further refinement.

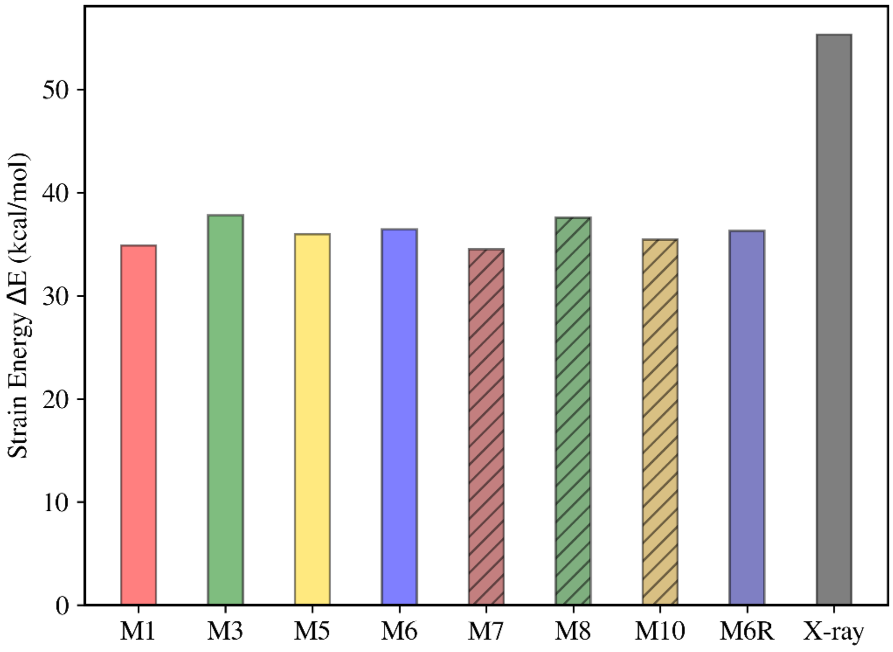

Supplementary Figure 125: Strain energy of forodesine. Strain energy (ΔE, kcal·mol^{-1}) at ωB97X-D/6-31G(d) level for forodesine (IMH) in human PNP determined by various quantum refinement schemes (**M1-M10**). Those results for X-ray were taken from the experimental structure without our further refinement.

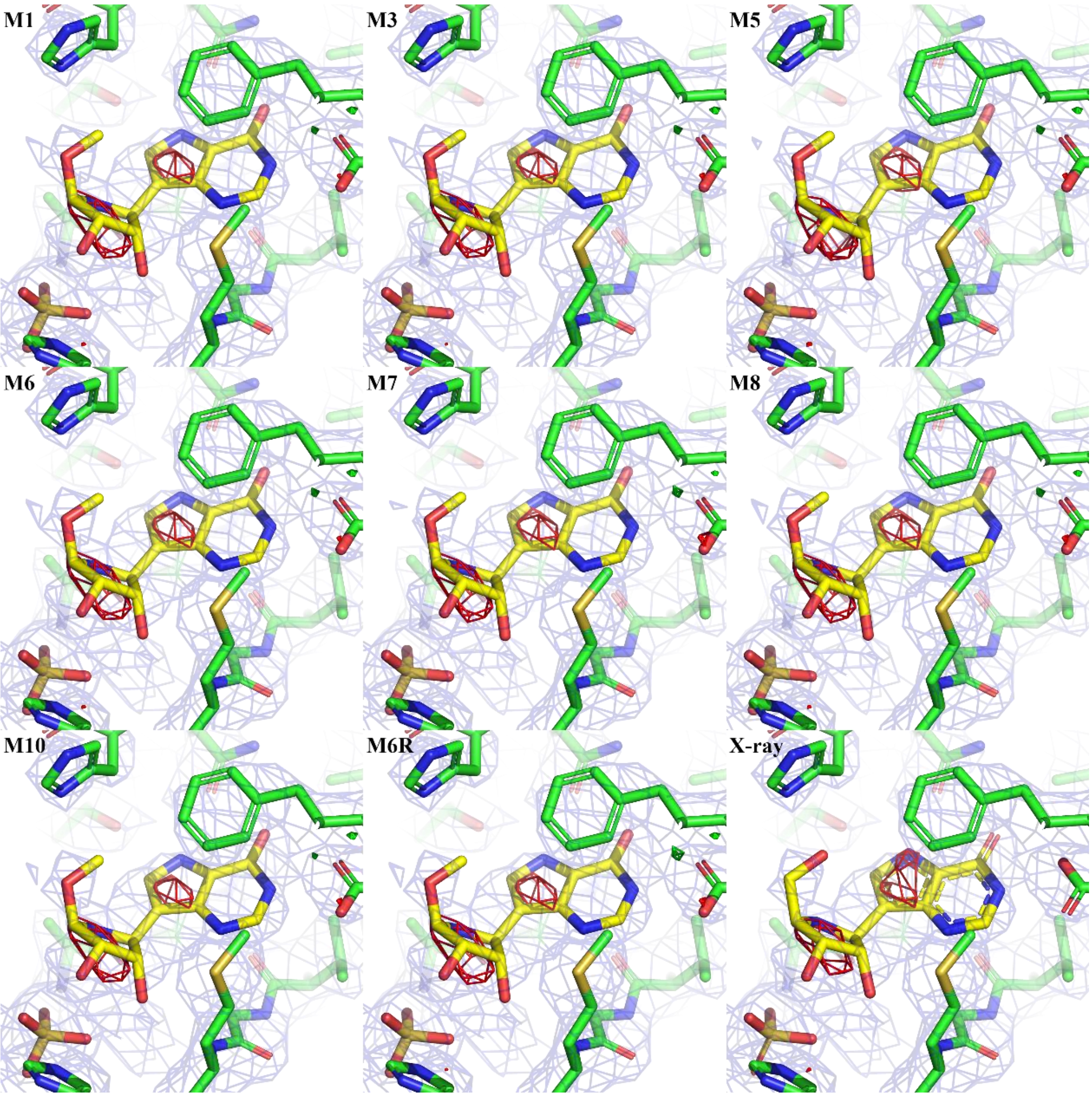

Supplementary Figure 126: Electron density maps of forodesine. Structures for forodesine (IMH) in human PNP from various quantum refinement schemes (**M1-M10**), including the electron density maps (2mFo-DFc maps, contoured at 1.0 σ (blue), mFo-DFc maps, contoured at +3.0 σ (green), and mFo-DFc maps, contoured at -3.0 σ (red)). Those results for X-ray were taken from the experimental structure without our further refinement.

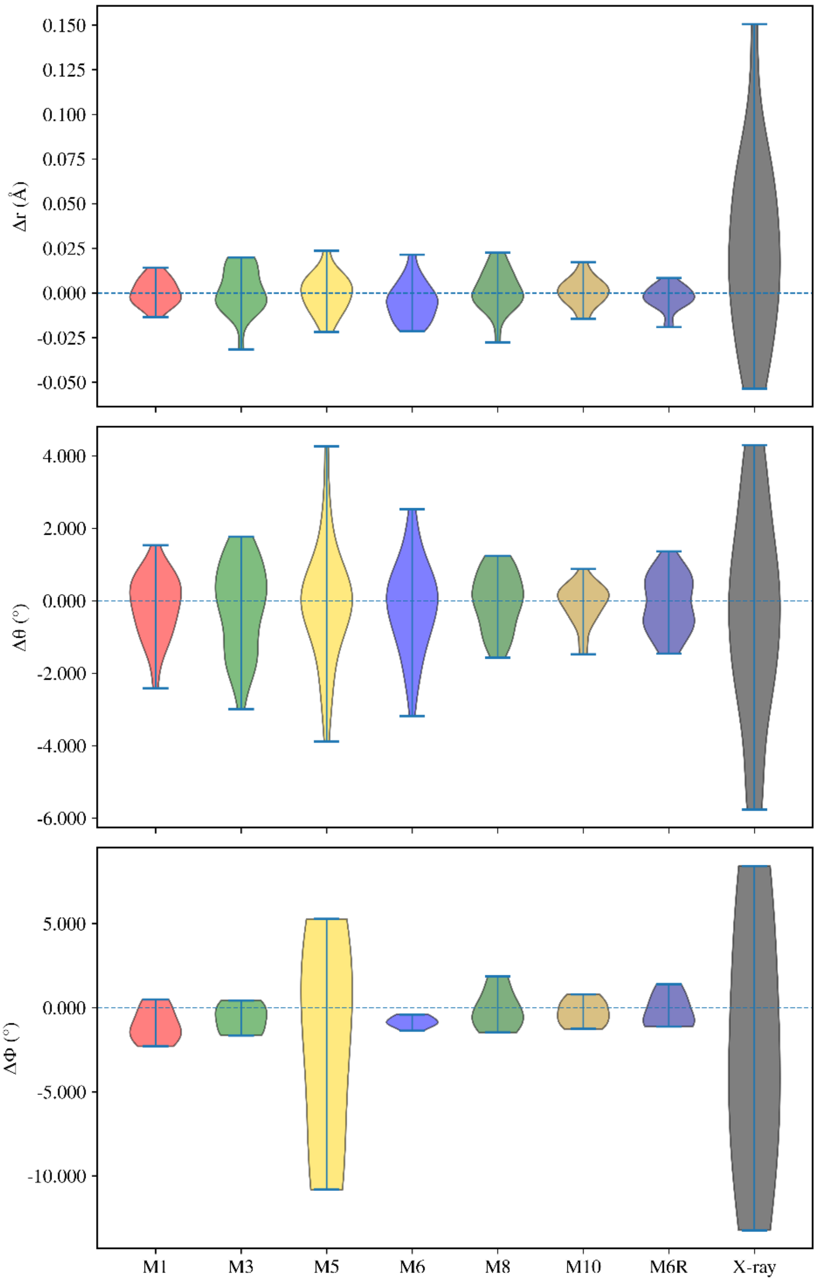

Supplementary Figure 127: Key coordinates of quantum refinement results of forodesine. Deviation in the refined bond distances (Δr, n = 21), angles (Δθ, n = 31) and dihedrals (Δϕ, n = 6) of forodesine (IMH) in human PNP from various quantum refinement schemes (**M1-M10**) and X-ray structure which are compared to those obtained from the most reliable **M7** scheme. The solid line represents the upper and lower values.

(xxi) 1M7Q (p38 MAP kinase in complex with a dihydroquinazolinone inhibitor)

Protein preparations:

Resolution: 2.40 Å

Ligand: DQQ; $C_{24}H_{20}Cl_2F_2N_4O$

Residue flipped: ASN100, GLN202, GLN310

Protonation states (pH = 7.0):

HID64, HID77, HID80, HID107, HID126, HID142, HID148, HIP174, HID199, HID228, HID305, HIP312

Optimized region: DQQ

High layer: DQQ

Medium layer: TYR35, VAL38, ALA51, LYS53, LEU75, ILE84, LEU104, THR106, HID107, LEU108, MET109, GLY110, ALA111, ASP112, ALA157, LEU167, ASP168, WAT696

ω_α = 1.7885

(a)

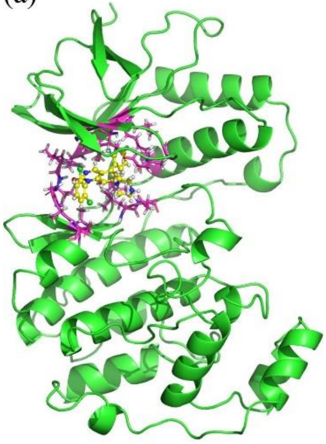

(b)

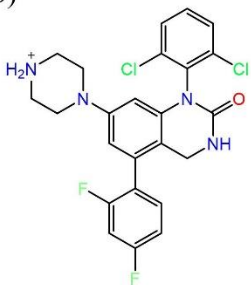

Supplementary Figure 128: P38 MAP kinase in complex with a dihydroquinazolinone inhibitor. (**a**) Crystal structure of p38 MAP kinase in complex with a dihydroquinazolinone inhibitor (DQQ). ONIOM layers by different colors: yellow: high layer; red: medium layer; green: low layer. Ligand dihydroquinazolinone inhibitor is presented in stick and balls. (**b**) Structure of dihydroquinazolinone inhibitor.

Quantum refined structural results:

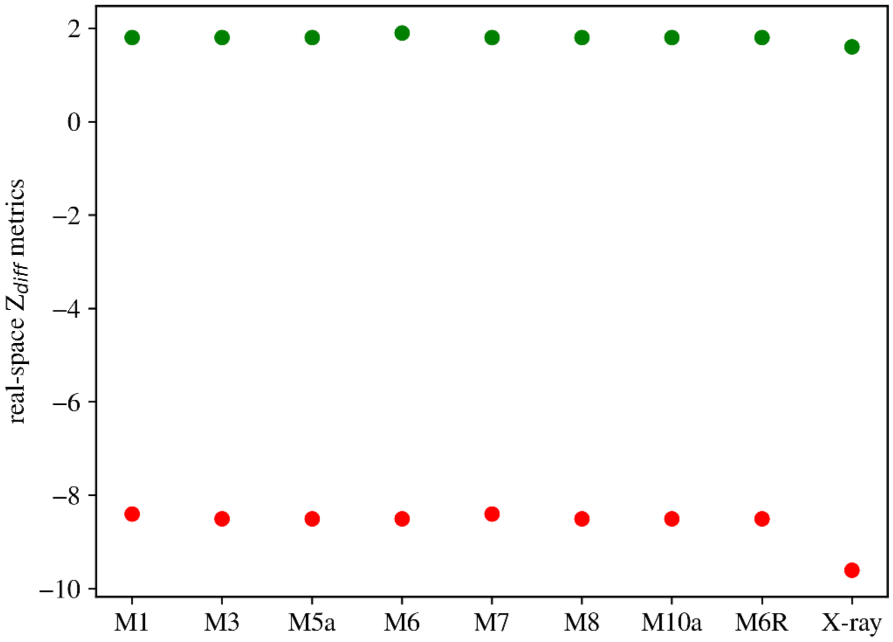

Supplementary Figure 129: Real-space Z-difference (RSZD) of dihydroquinazolinone inhibitor. RSZD+ (green) and RSZD- (red) scores of dihydroquinazolinone inhibitor (DQQ) in p38 MAP kinase from various quantum refinement schemes (**M1-M10**). Those results for X-ray were taken from the experimental structure without our further refinement.

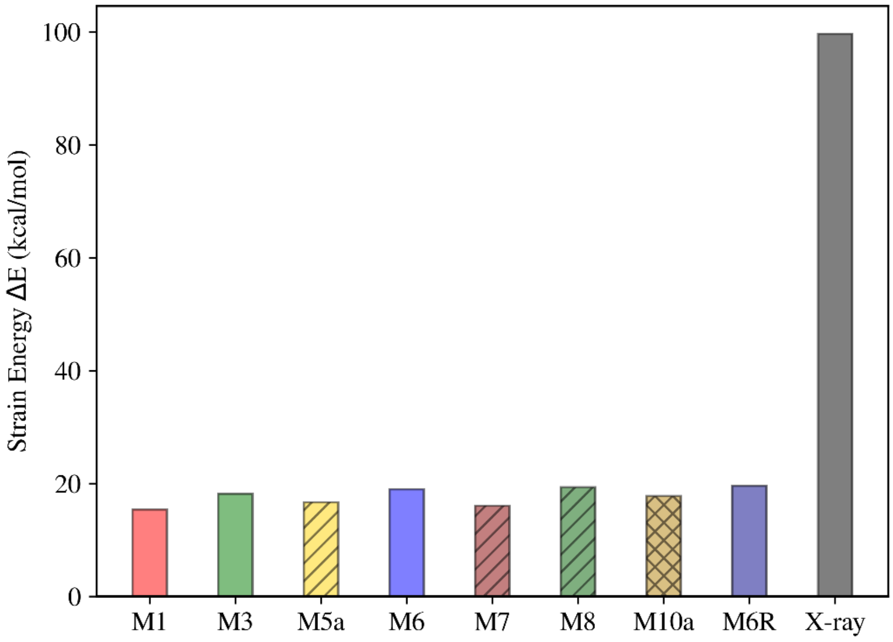

Supplementary Figure 130: Strain energy of dihydroquinazolinone inhibitor. Strain energy (ΔE, kcal·mol^{-1}) at ωB97X-D/6-31G(d) level for dihydroquinazolinone inhibitor (DQQ) in p38 MAP kinase determined by various quantum refinement schemes (**M1-M10**). Those results for X-ray were taken from the experimental structure without our further refinement.

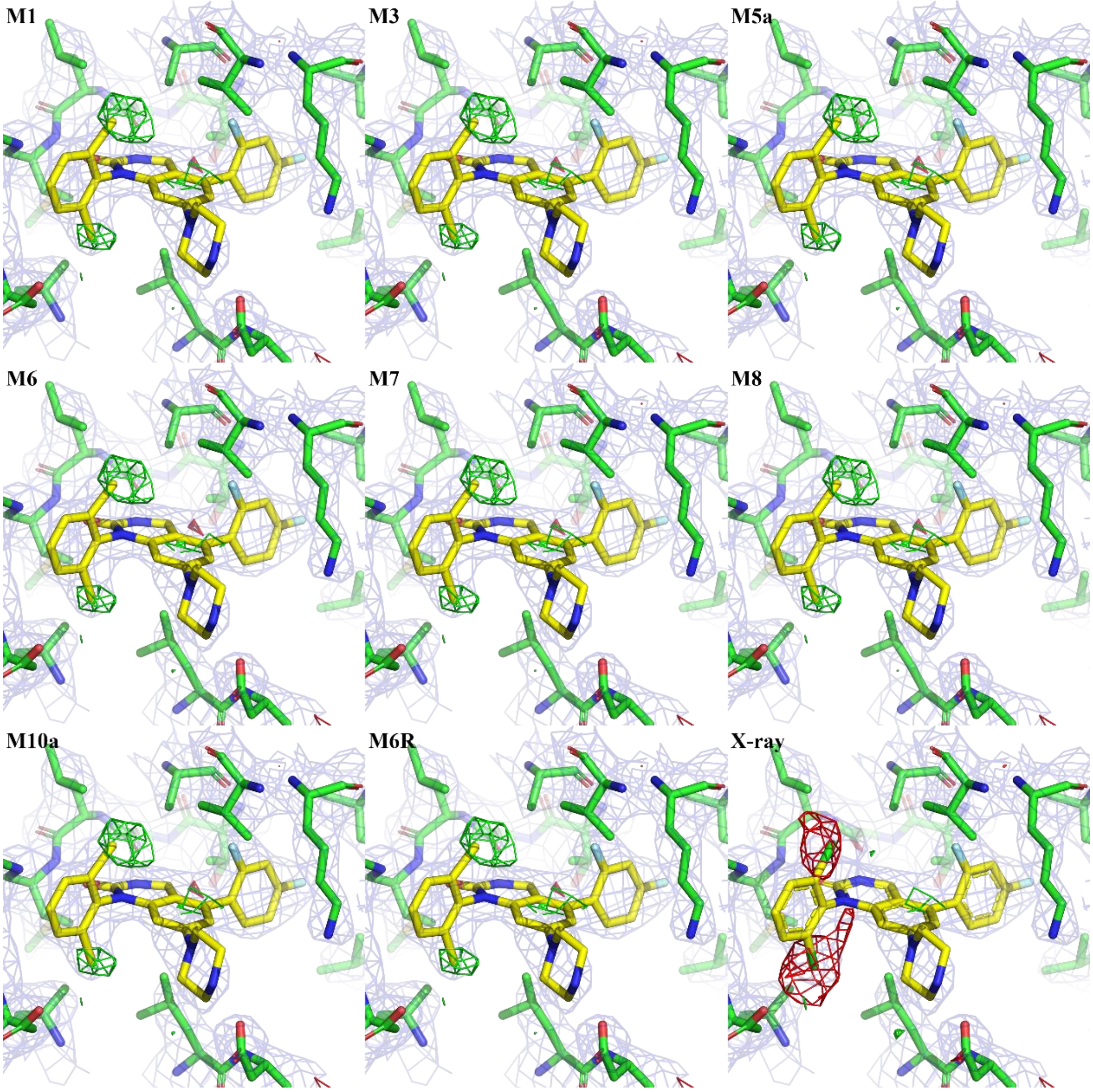

Supplementary Figure 131: Electron density maps of dihydroquinazolinone inhibitor. Structures for dihydroquinazolinone inhibitor (DQQ) in p38 MAP kinase from various quantum refinement schemes (**M1**-**M10**), including the electron density maps (2mFo-DFc maps, contoured at 1.0 σ (blue), mFo-DFc maps, contoured at +3.0 σ (green), and mFo-DFc maps, contoured at -3.0 σ (red)). Those results for X-ray were taken from the experimental structure without our further refinement.

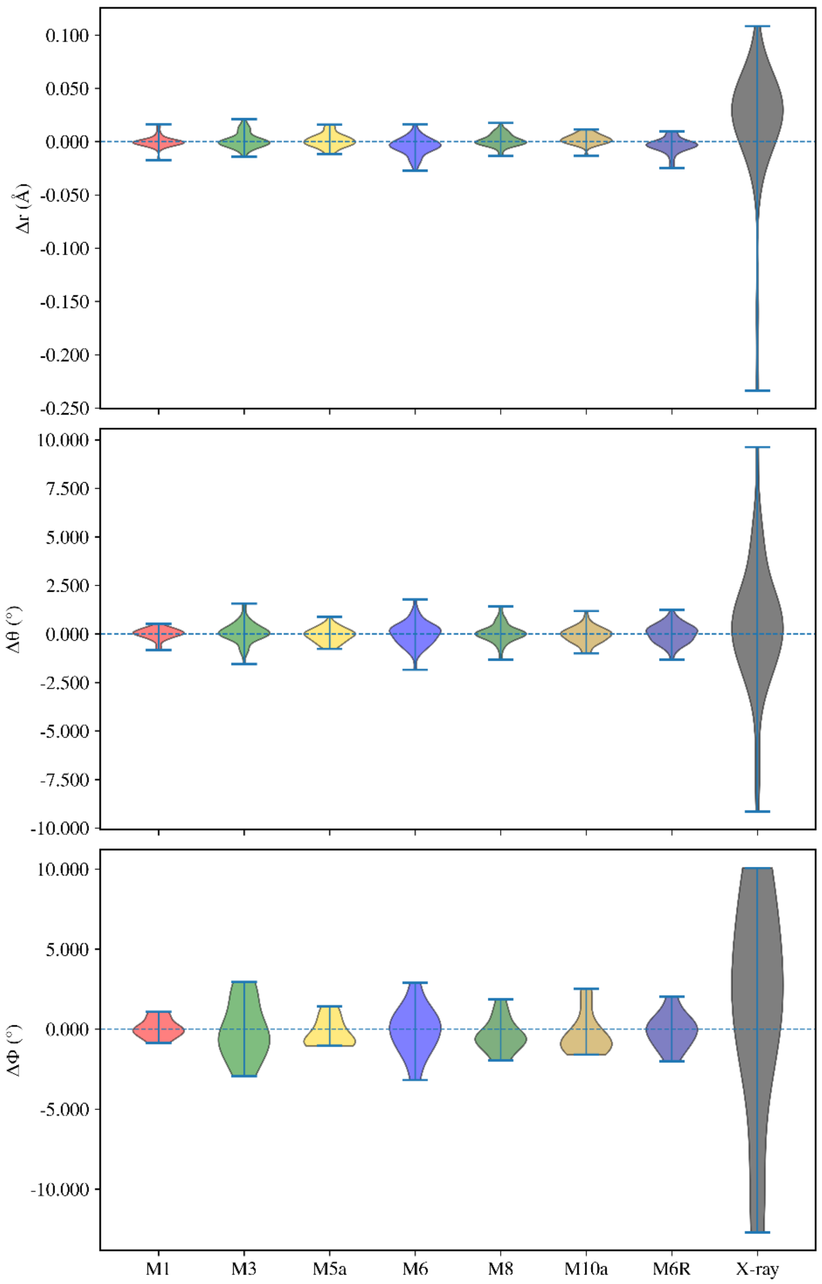

Supplementary Figure 132: Key coordinates of quantum refinement results of dihydroquinazolinone inhibitor. Deviation in the refined bond distances (Δr, n = 37), angles (Δθ, n = 54) and dihedrals (Δϕ, n = 12) of dihydroquinazolinone inhibitor (DQQ) in p38 MAP kinase from various quantum refinement schemes (**M1-M10**) and X-ray structure which are compared to those obtained from the most reliable **M7** scheme. The solid line represents the upper and lower values.

(xxii) 1PA9 (Yersinia Protein-Tyrosine Phosphatase complexed with pNCS)

Protein preparations:

Resolution: 2.50 Å

Ligand: CSN (nitrocatechol sulfate); $C_6H_5NO_7S$

Residue flipped: ASN44, GLN75, GLN142, GLN157, ASN258, GLN273, ASN305

Protonation states (pH = 5.5):

HID108, HID188, ASH194, HID240, CYM241

Optimized region: CSN

High layer: CSN

Medium layer: PHE67, ILE70, ASH194, GLN195, ARG242, ALA243, VAL245, GLY246, ARG247, ILE281, GLN284, WAT810

ω_{α} = 0.89892

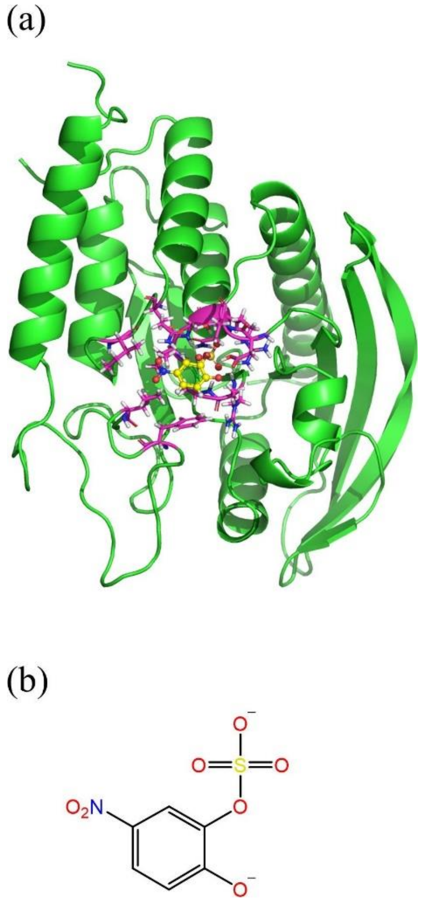

Supplementary Figure 133: Yersinia Protein-Tyrosine Phosphatase complexed with pNCS. (**a**) Crystal structure of Yersinia protein-tyrosine phosphatase complexed with nitrocatechol sulfate (CSN). ONIOM layers by different colors: yellow: high layer; red: medium layer; green: low layer. Ligand nitrocatechol sulfate is presented in stick and balls. (**b**) Structure of nitrocatechol sulfate.

Quantum refined structural results:

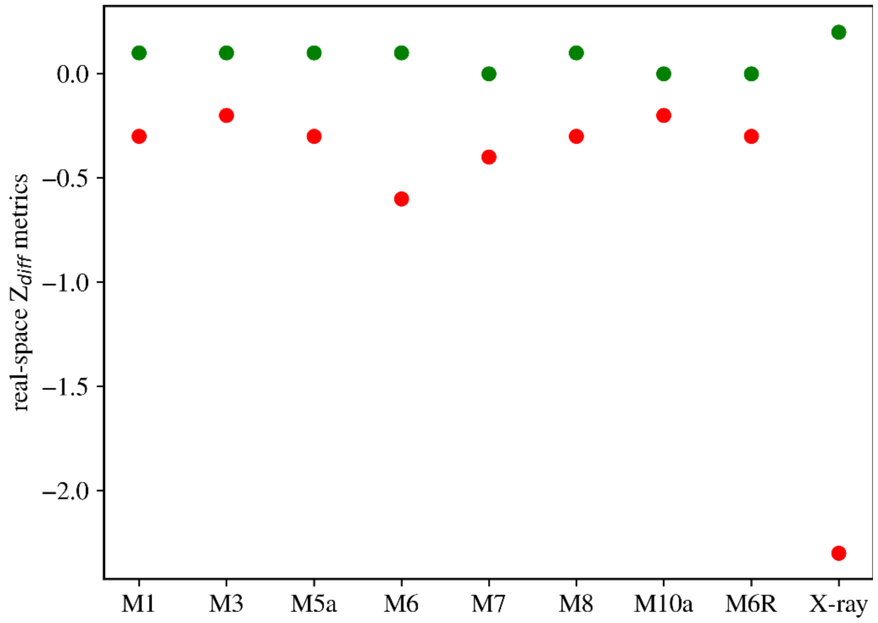

Supplementary Figure 134: Real-space Z-difference (RSZD) of nitrocatechol sulfate. RSZD+ (green) and RSZD- (red) scores of nitrocatechol sulfate (CSN) in Yersinia protein-tyrosine phosphatase various quantum refinement schemes (**M1-M10**). Those results for X-ray were taken from the experimental structure without our further refinement.

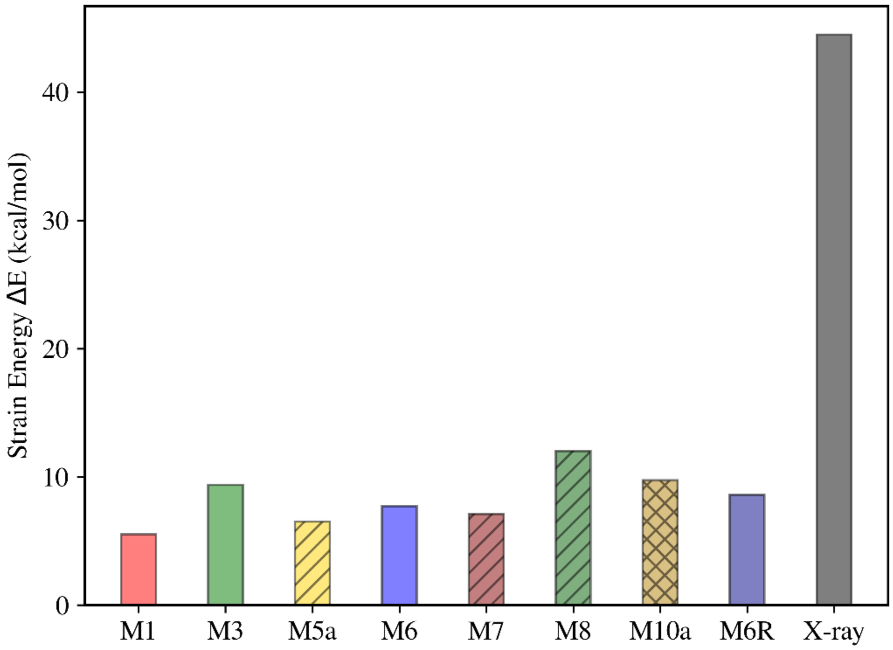

Supplementary Figure 135: Strain energy of nitrocatechol sulfate. Strain energy (ΔE, kcal·mol^{-1}) at ωB97X-D/6-31G(d) level for nitrocatechol sulfate (CSN) in Yersinia protein-tyrosine phosphatase determined by various quantum refinement schemes (**M1-M10**). Those results for X-ray were taken from the experimental structure without our further refinement.

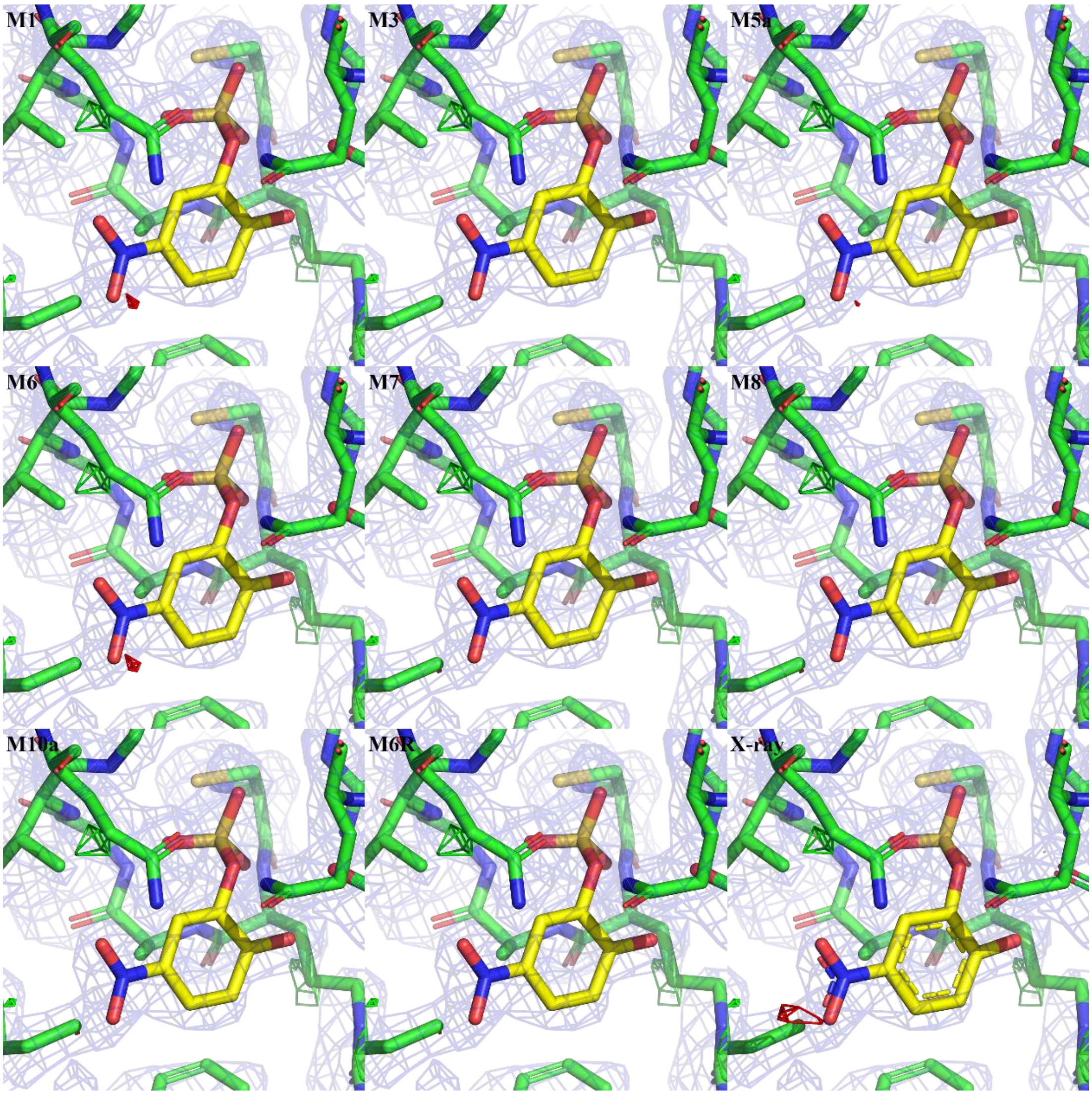

Supplementary Figure 136: Electron density maps of nitrocatechol sulfate. Structures for nitrocatechol sulfate (CSN) in Yersinia protein-tyrosine phosphatase from various quantum refinement schemes (**M1-M10**), including the electron density maps (2mFo-DFc maps, contoured at 1.0 σ (blue), mFo-DFc maps, contoured at +3.0 σ (green), and mFo-DFc maps, contoured at -3.0 σ (red)). Those results for X-ray were taken from the experimental structure without our further refinement.

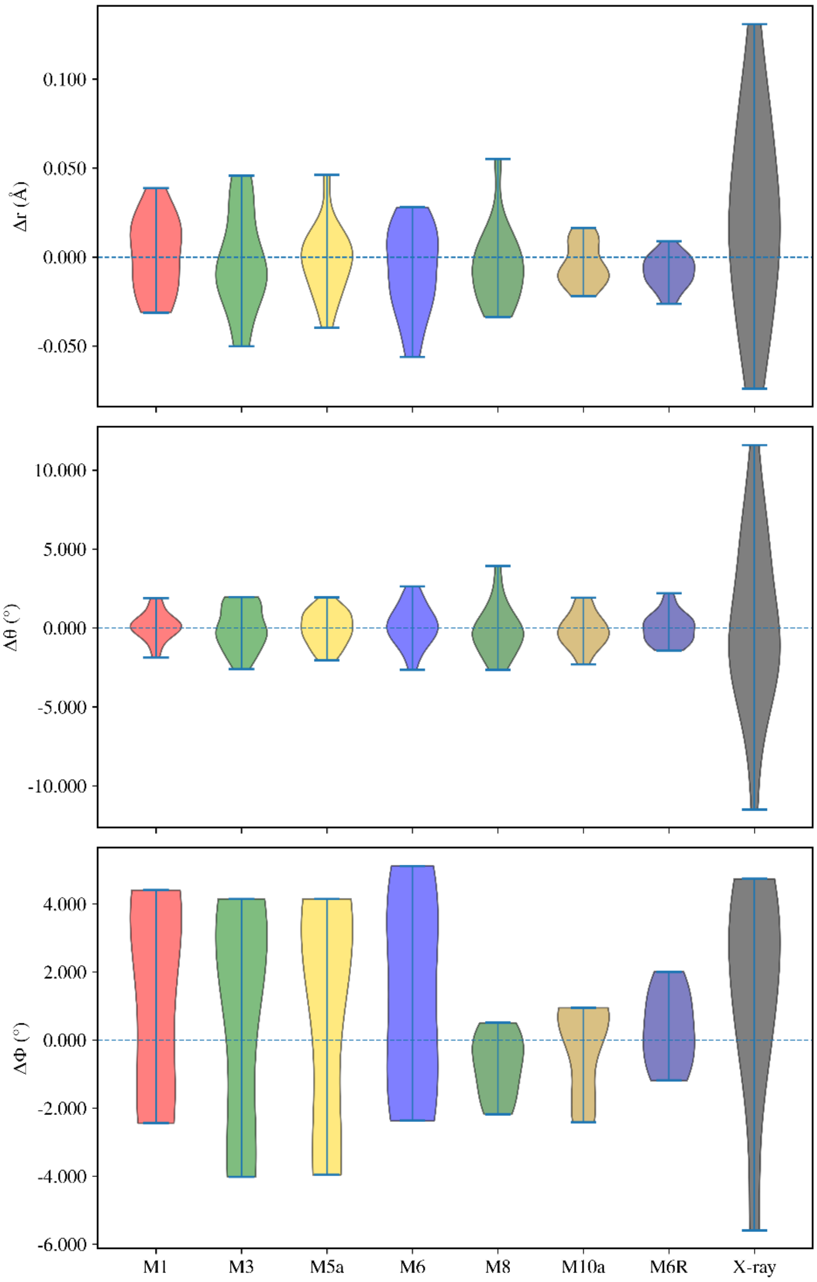

Supplementary Figure 137: Key coordinates of quantum refinement results of nitrocatechol sulfate. Deviation in the refined bond distances (Δr, n = 15), angles (Δθ, n = 22) and dihedrals (Δϕ, n = 9) of nitrocatechol sulfate (CSN) in Yersinia protein-tyrosine phosphatase from various quantum refinement schemes (**M1-M10**) and X-ray structure which are compared to those obtained from the most reliable **M7** scheme. The solid line represents the upper and lower values.

(xxiii) 1AJX (HIV-1 protease in complex with the cyclic urea inhibitor aha001)

Protein preparations:

Resolution: 2.00 Å

Ligand: AH1; $C_{33}H_{34}N_2O_5$

Residue flipped:

Chain A: GLN92

Chain B: GLN18

Protonation states (pH = 5.5):

Chain A: HIP69,

Chain B: ASH25, HIP69

Optimized region: AH1

High layer: AH1

Medium layer:

Chain A: ARG8, ASP25, GLY27, ALA28, ASP30, VAL32, GLY49, ILE50, PRO81, VAL82, ILE84, WAT330, WAT386

Chain B: ARG8, LEU23, ASH25, GLY27, ALA28, ASP29, ASP30, VAL32, GLY48, GLY49, ILE50, PRO81, ILE84, WAT301, WAT369, WAT401

ω_α = 0.52168

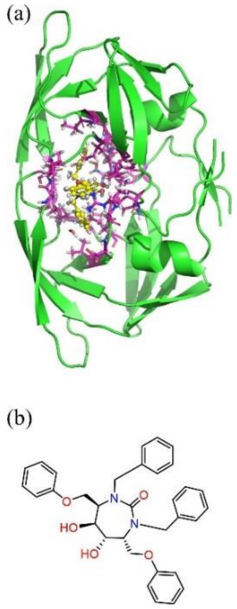

Supplementary Figure 138: HIV-1 protease in complex with the cyclic urea inhibitor aha001. (**a**) Crystal structure of HIV-1 protease complexed with AH1. ONIOM layers by different colors: yellow: high layer; red: medium layer; green: low layer. Ligand AH1 is presented in stick and balls. (**b**) Structure of AH1.

Quantum refined structural results:

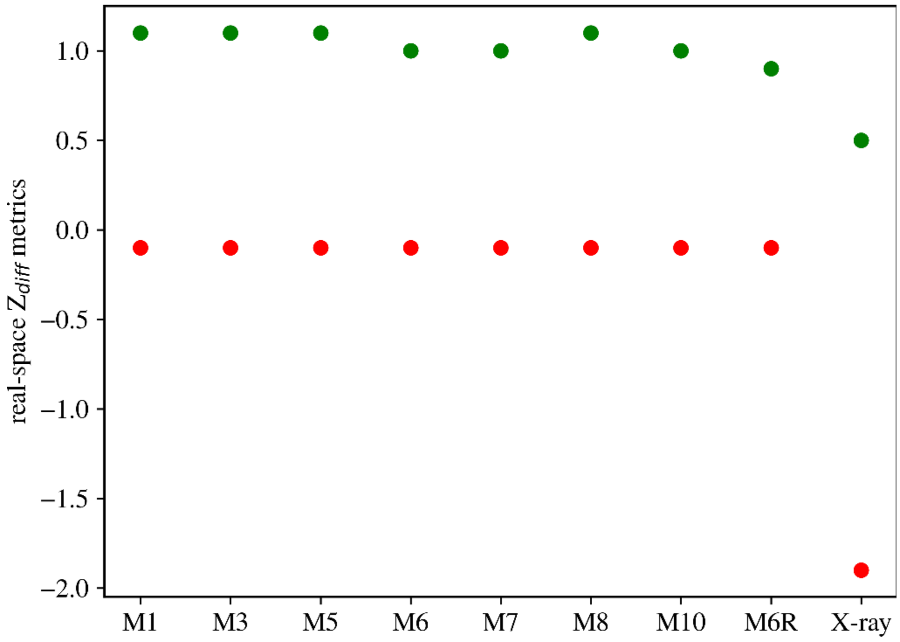

Supplementary Figure 139: Real-space Z-difference (RSZD) of AH1. RSZD+ (green) and RSZD- (red) scores of AH1 in HIV-1 protease from various quantum refinement schemes (**M1-M10**). Those results for X-ray were taken from the experimental structure without our further refinement.

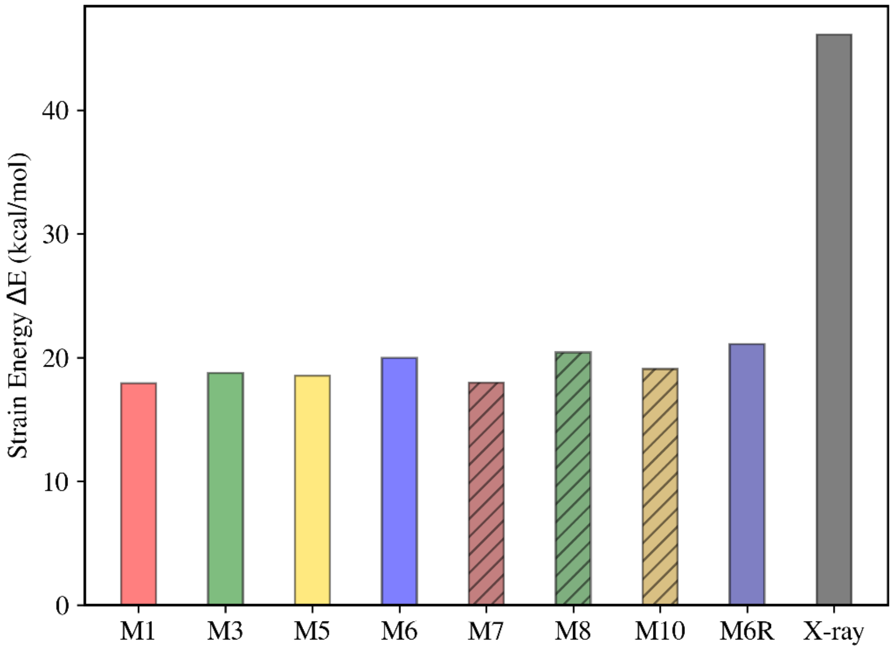

Supplementary Figure 140: Strain energy of AH1. Strain energy (ΔE, kcal·mol^{-1}) at ωB97X-D/6-31G(d) level of AH1 in HIV-1 protease determined by various quantum refinement schemes (**M1-M10**). Those results for X-ray were taken from the experimental structure without our further refinement.

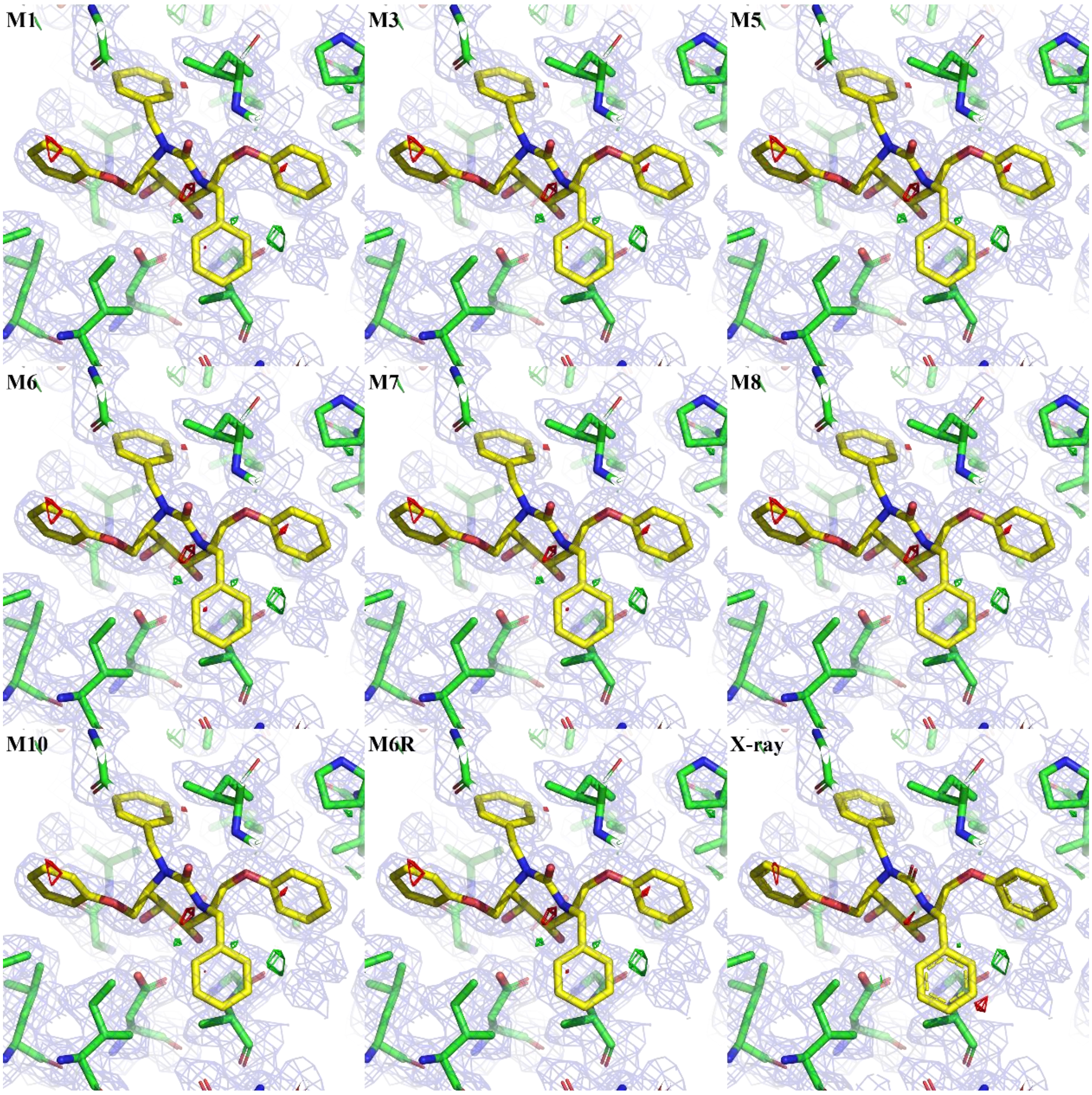

Supplementary Figure 141: Electron density maps of AH1. Structures for AH1 in HIV-1 protease from various quantum refinement schemes (**M1**-**M10**), including the electron density maps (2mFo-DFc maps, contoured at 1.0 σ (blue), mFo-DFc maps, contoured at +3.0 σ (green), and mFo-DFc maps, contoured at -3.0 σ (red)). Those results for X-ray were taken from the experimental structure without our further refinement.

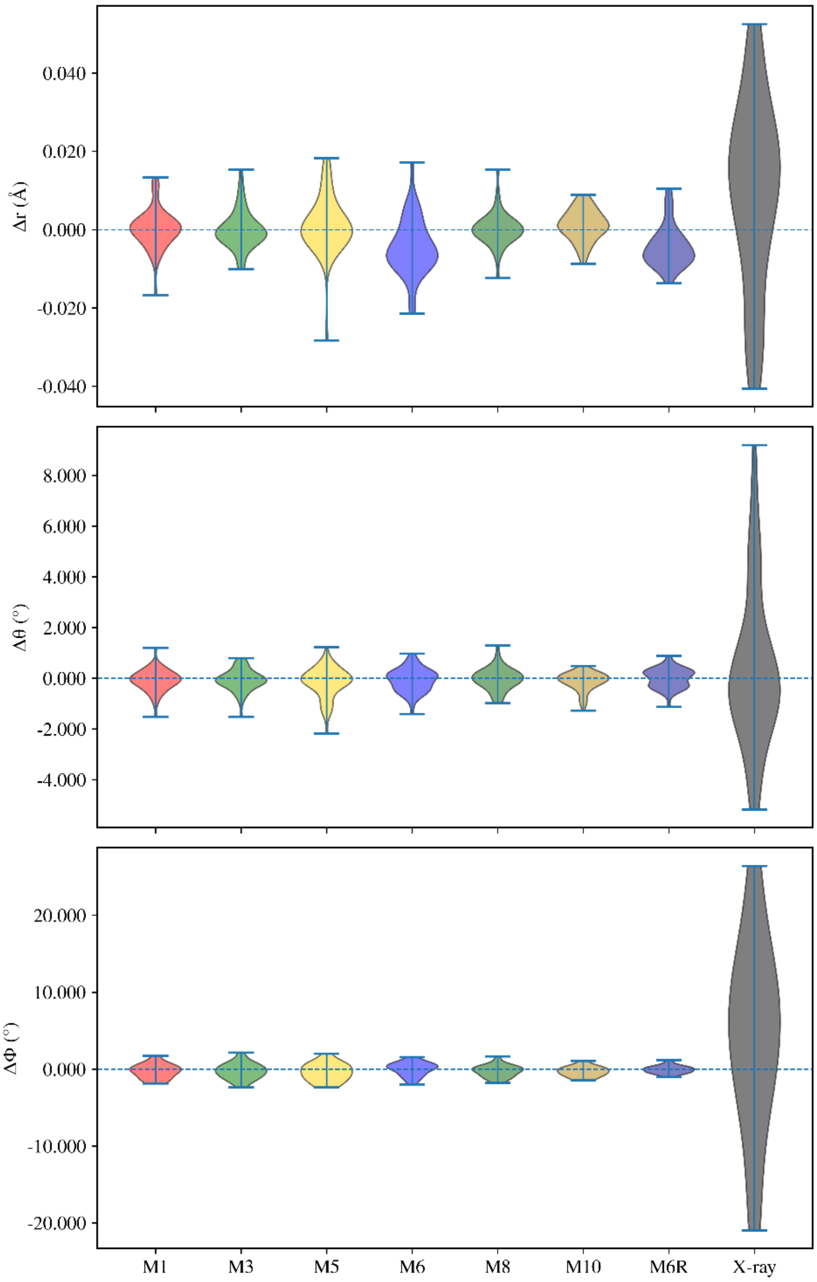

Supplementary Figure 142: Key coordinates of quantum refinement results of AH1. Deviation in the refined bond distances (Δr, n = 44), angles (Δθ, n = 59) and dihedrals (Δϕ, n = 18) of AH1 in HIV-1 protease from various quantum refinement schemes (**M1-M10**) and X-ray structure which are compared to those obtained from the most reliable **M7** scheme. The solid line represents the upper and lower values.

(xxiv) 1D4I (HIV-1 protease in complex with the inhibitor BEA425)

Protein preparations:

Resolution: 1.81 Å

Ligand: BEG; $C_{38}H_{40}N_2O_7$

Residue flipped: No

Protonation states (pH = 5.5):

Chain A: ASH25, HIP69,

Chain B: HIP69

Optimized region: BEG

High layer: BEG

Medium layer:

Chain A: ARG8, LEU23, ASH25, GLY27, ALA28, ASP29, ASP30, VAL32, GLY48, GLY49, ILE50, LEU76, PRO81, VAL82, ILE84, WAT319, WAT329, WAT339, WAT356, WAT361, WAT368

Chain B: ARG108, LEU123, ASP125, GLY127, ALA128, ASP129, ASP130, VAL132, ILE147, GLY148, GLY149, ILE150, LEU176, PRO181, ILE184, WAT349, WAT378, WAT425

ω_α = 0.35964

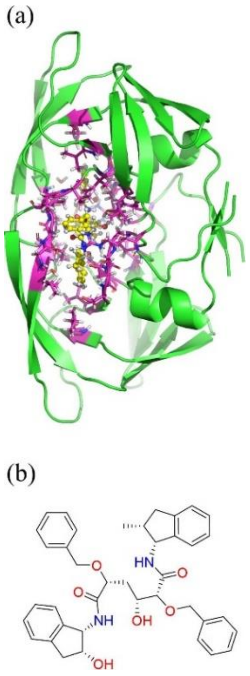

Supplementary Figure 143: HIV-1 protease in complex with the inhibitor BEA425. (**a**) Crystal structure of HIV-1 protease complexed with BEG. ONIOM layers by different colors: yellow: high layer; red: medium layer; green: low layer. Ligand BEG is presented in stick and balls. (**b**) Structure of BEG.

Quantum refined structural results:

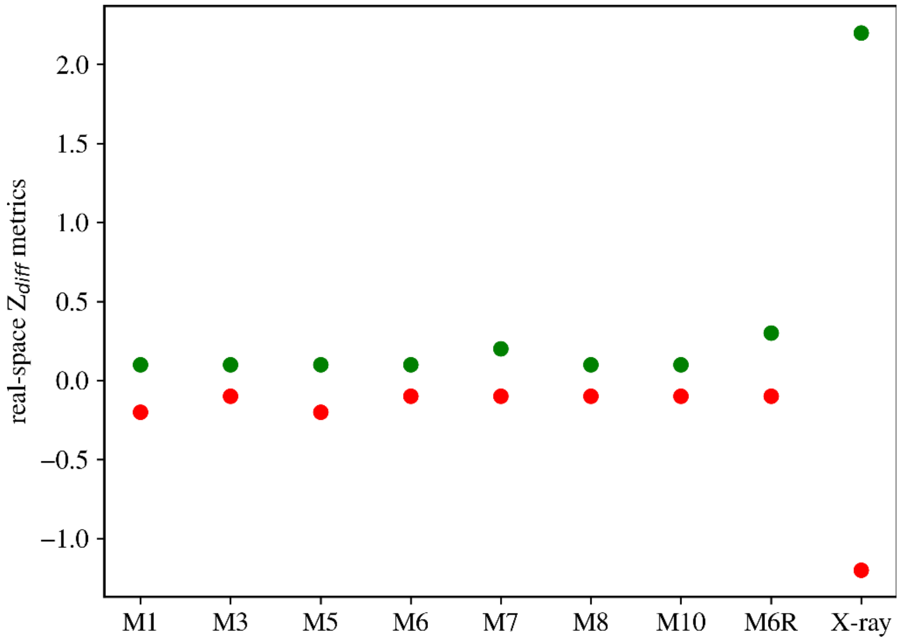

Supplementary Figure 144: Real-space Z-difference (RSZD) of BEG. RSZD+ (green) and RSZD- (red) scores of BEG in HIV-1 protease from various quantum refinement schemes (**M1-M10**). Those results for X-ray were taken from the experimental structure without our further refinement.

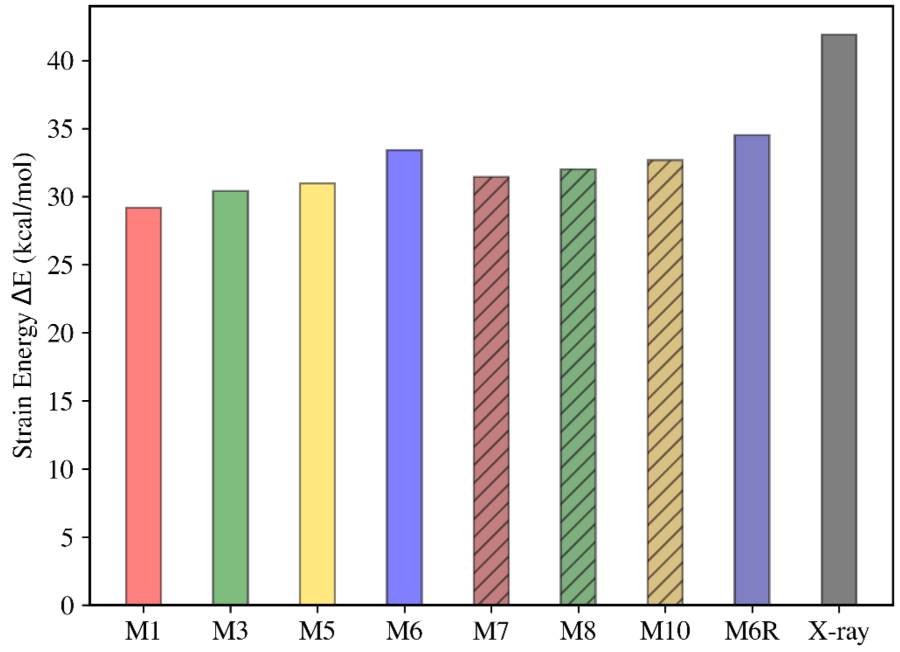

Supplementary Figure 145: Strain energy of BEG. Strain energy (ΔE, kcal·mol^{-1}) at ωB97X-D/6-31G(d) level of BEG in HIV-1 protease determined by various quantum refinement schemes (**M1-M10**). Those results for X-ray were taken from the experimental structure without our further refinement.

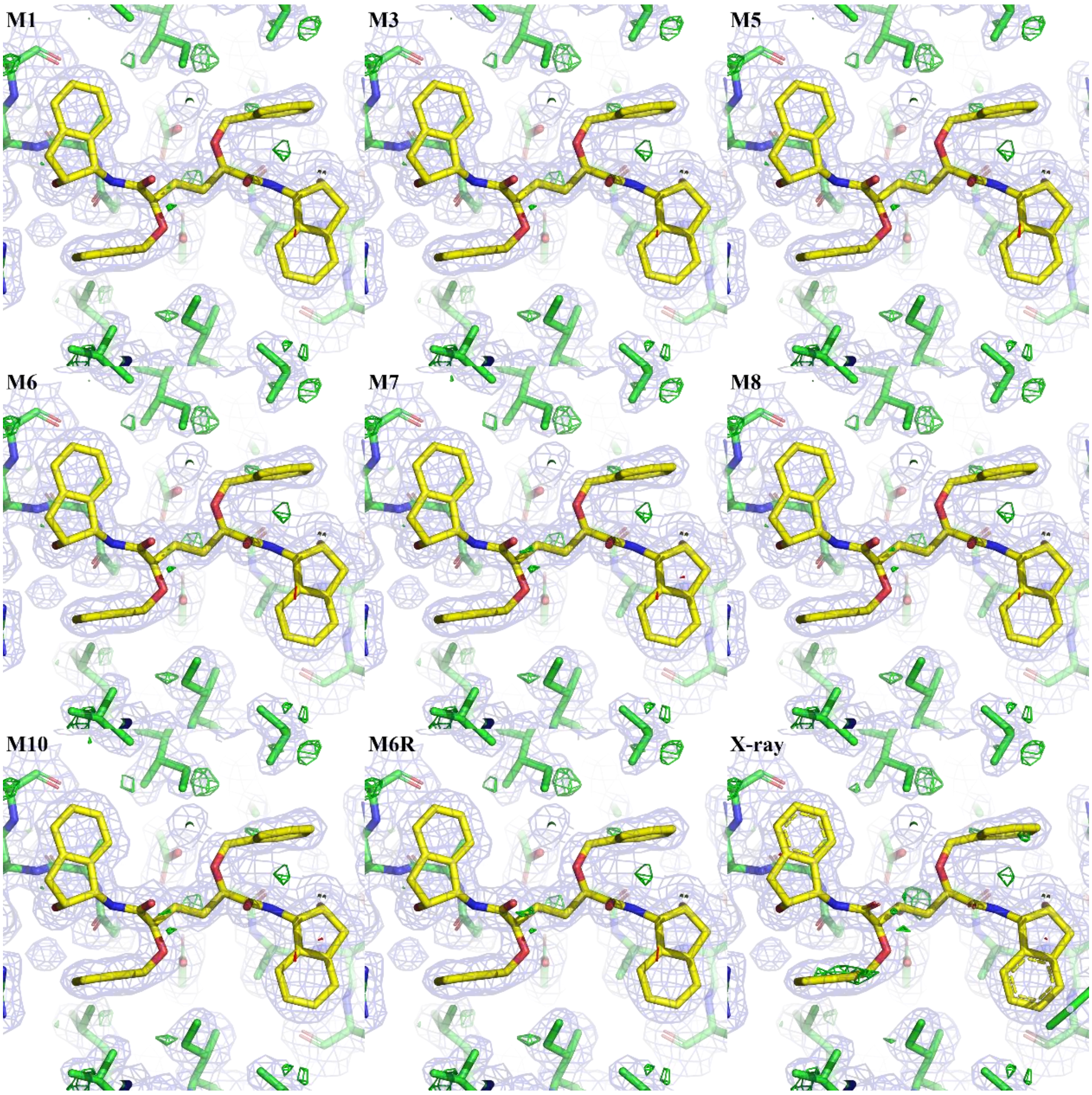

Supplementary Figure 146: Electron density maps of BEG. Structures for BEG in HIV-1 protease from various quantum refinement schemes (**M1-M10**), including the electron density maps (2mFo-DFc maps, contoured at 1.0 σ (blue), mFo-DFc maps, contoured at +3.0 σ (green), and mFo-DFc maps, contoured at -3.0 σ (red)). Those results for X-ray were taken from the experimental structure without our further refinement.

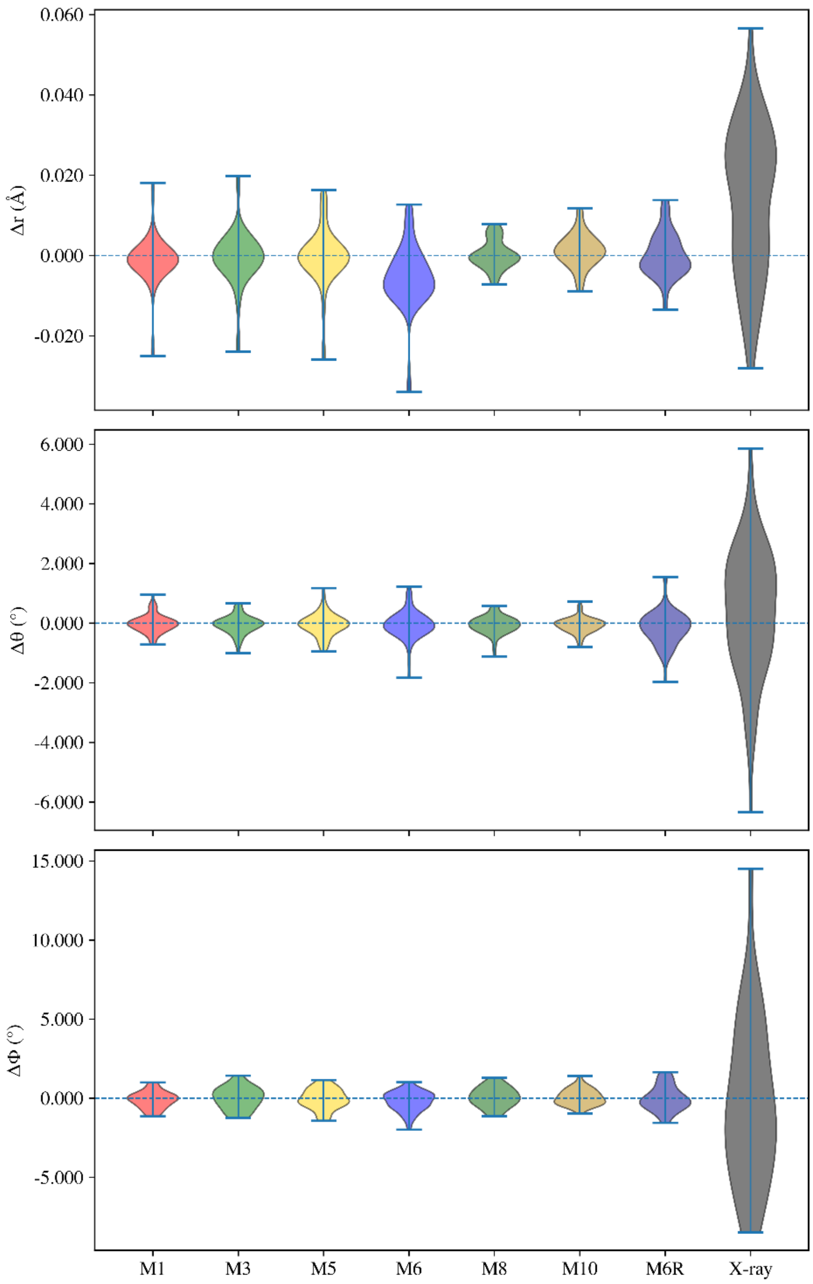

Supplementary Figure 147: Key coordinates of quantum refinement results of BEG. Deviation in the refined bond distances (Δr, n = 52), angles (Δθ, n = 72) and dihedrals (Δϕ, n = 34) of BEG in HIV-1 protease from various quantum refinement schemes (**M1-M10**) and X-ray structure which are compared to those obtained from the most reliable **M7** scheme. The solid line represents the upper and lower values.

(xxv) 1XOZ (Human Phosphodiesterase 5A complexed with Tadalafil)

Protein preparations:

Resolution: 2.50 Å

Ligand: CIA (Tadalafil); $C_{22}H_{19}N_3O_4$

Residue flipped: ASN698, GLN743, ASN811

Protonation states (pH = 7.0):

HID592, HID613, HID617, CYM624, HID653, HID657, HID683, HID684, HID685, HIP750, HID834

Optimized region: CIA

High layer: CIA

Medium layer: TYR612, ALA767, ILE768, GLN775, ILE778, ALA779, VAL782, ALA783, PHE786, LEU804, ILE813, MET816, GLN817, PHE820, WAT1014, WAT1017, WAT1128, WAT1148, WAT1169, WAT1232

$\omega_\alpha = 0.33074$

(a)

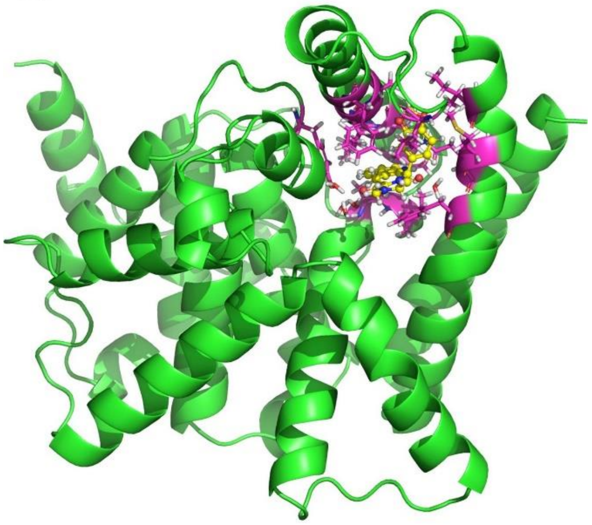

(b)

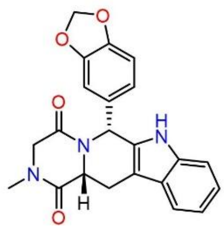

Supplementary Figure 148: Human Phosphodiesterase 5A complexed with Tadalafil. (**a**) Crystal structure of Human Phosphodiesterase 5A complexed with tadalafil (CIA). ONIOM layers by different colors: yellow: high layer; red: medium layer; green: low layer. Ligand tadalafil is presented in stick and balls. (**b**) Structure of tadalafil.

Quantum refined structural results:

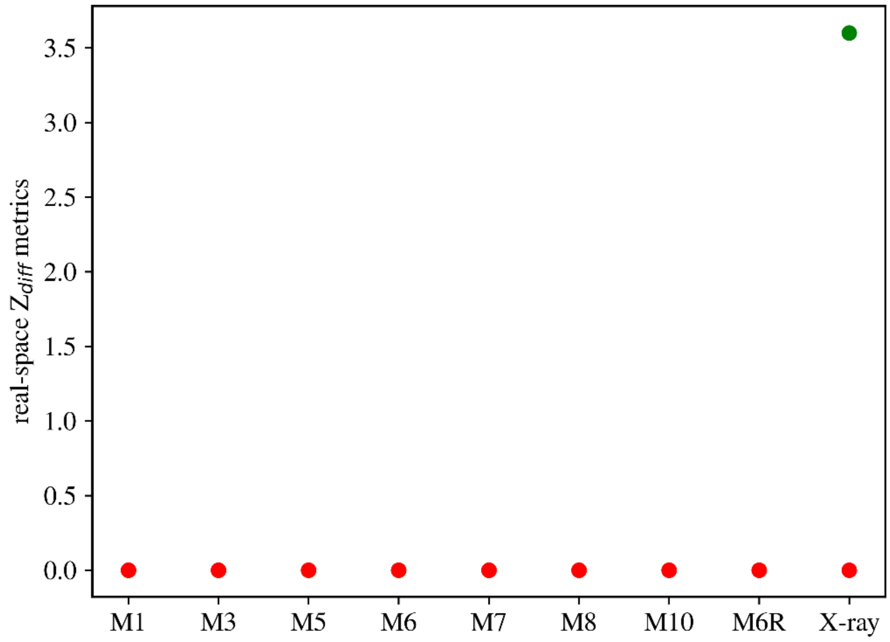

Supplementary Figure 149: Real-space Z-difference (RSZD) of tadalafil. RSZD+ (green) and RSZD- (red) scores of tadalafil (CIA) in Human Phosphodiesterase 5A from various quantum refinement schemes (**M1-M10**). Those results for X-ray were taken from the experimental structure without our further refinement.

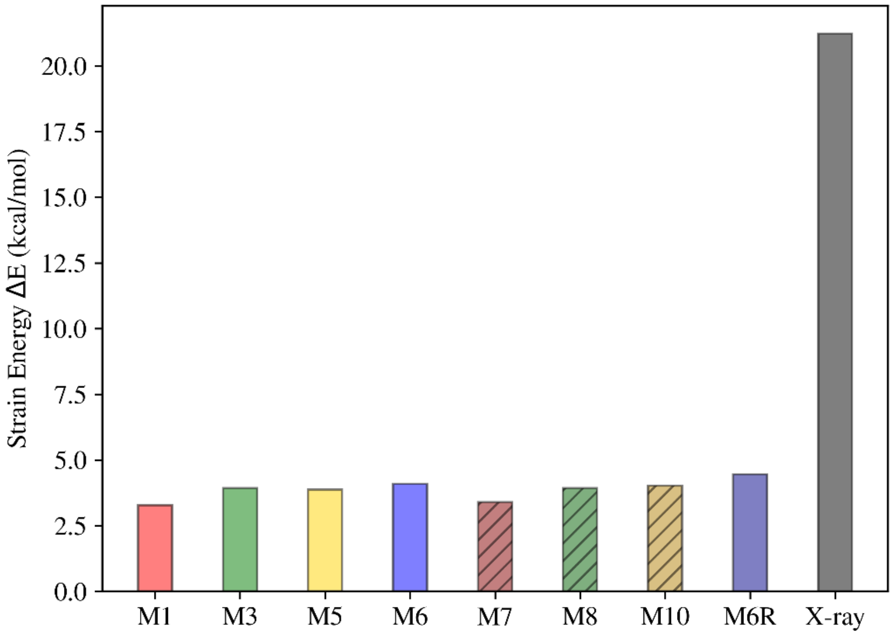

Supplementary Figure 150: Strain energy of tadalafil. Strain energy (ΔE, kcal·mol^{-1}) at ωB97X-D/6-31G(d) level for tadalafil (CIA) in Human Phosphodiesterase 5A determined by various quantum refinement schemes (**M1-M10**). Those results for X-ray were taken from the experimental structure without our further refinement.

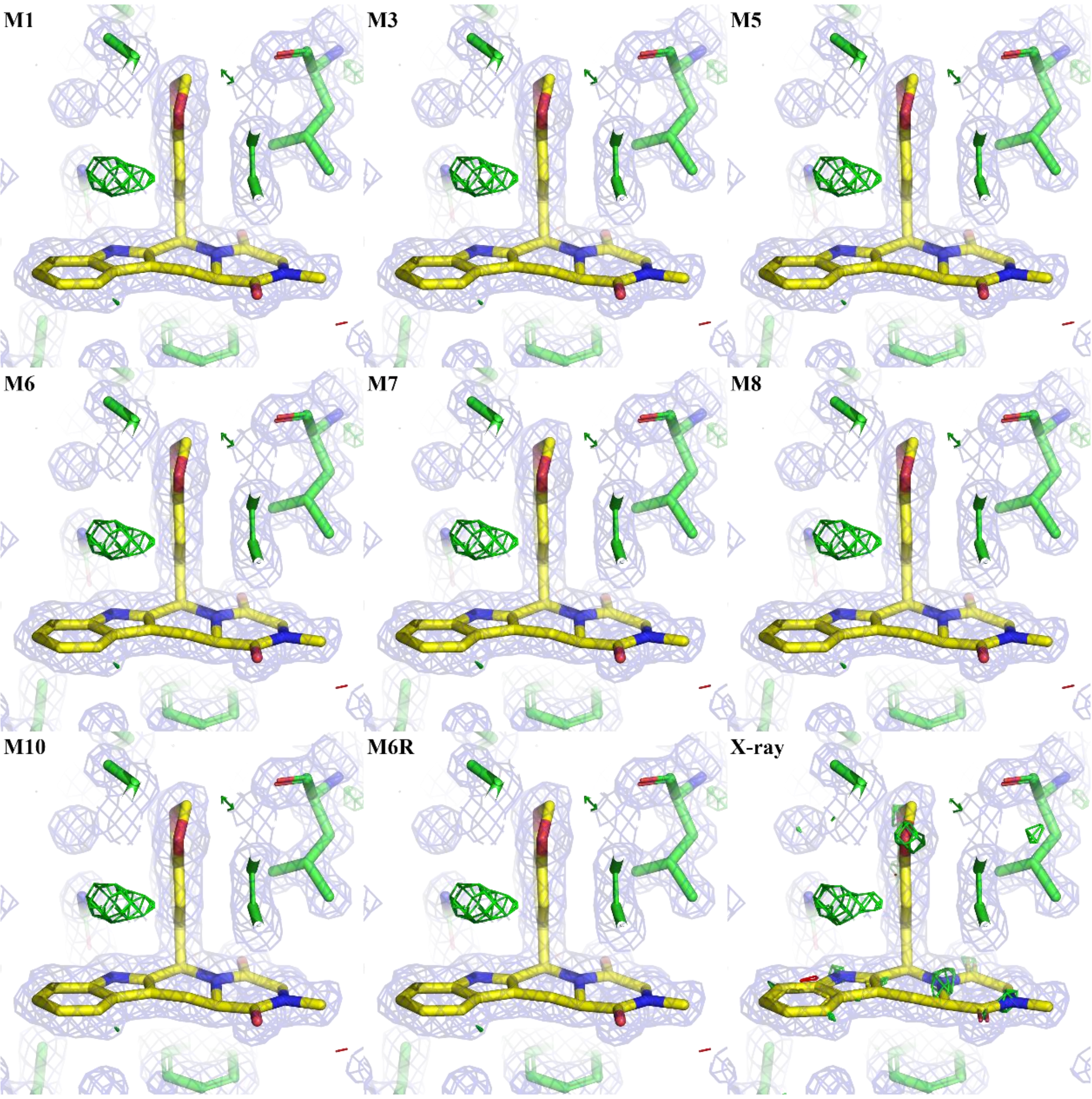

Supplementary Figure 151: Electron density maps of tadalafil. Structures for tadalafil (CIA) in Human Phosphodiesterase 5A from various quantum refinement schemes (**M1-M10**), including the electron density maps (2mFo-DFc maps, contoured at 1.0 σ (blue), mFo-DFc maps, contoured at +3.0 σ (green), and mFo-DFc maps, contoured at -3.0 σ (red)). Those results for X-ray were taken from the experimental structure without our further refinement.

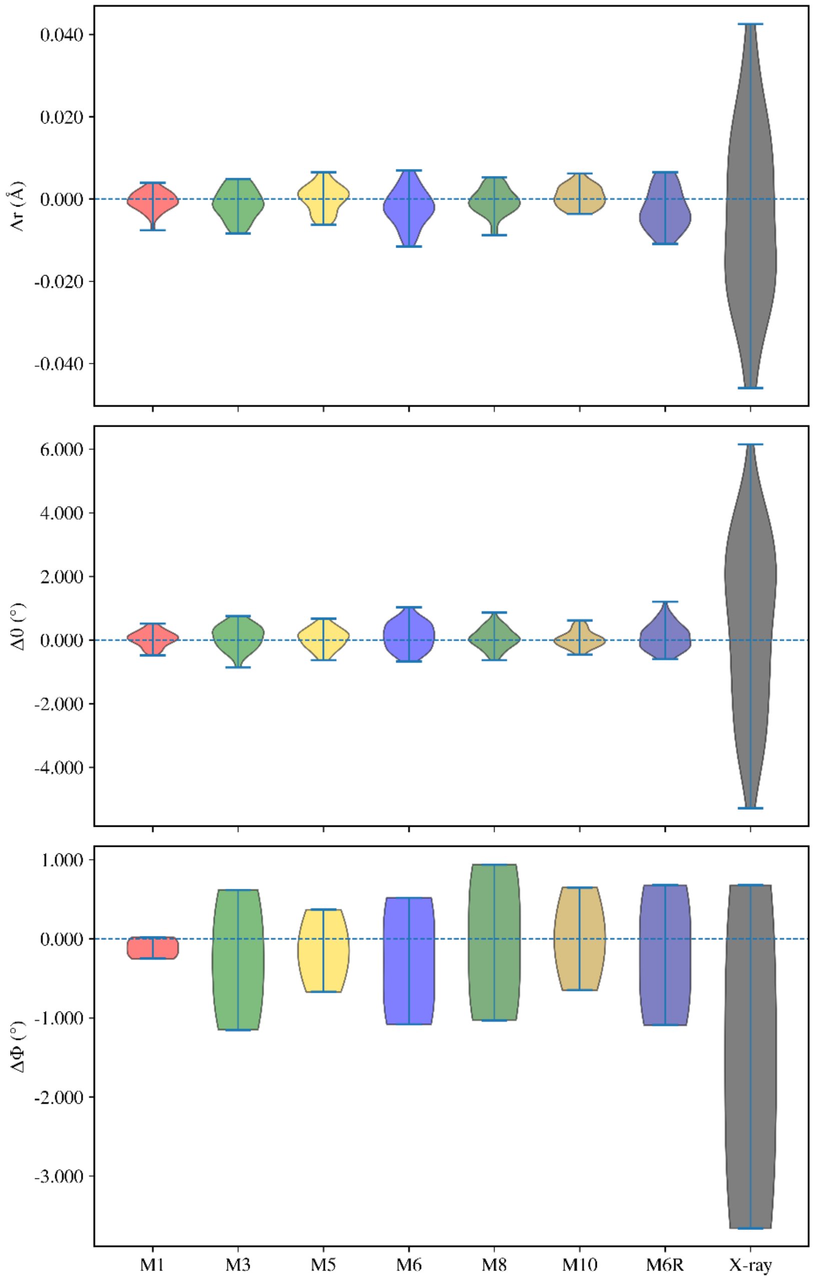

Supplementary Figure 152: Key coordinates of quantum refinement results of tadalafil. Deviation in the refined bond distances (Δr, n = 34), angles (Δθ, n = 52) and dihedrals (Δϕ, n = 4) of tadalafil (CIA) in Human Phosphodiesterase 5A from various quantum refinement schemes (**M1-M10**) and X-ray structure which are compared to those obtained from the most reliable **M7** scheme. The solid line represents the upper and lower values.

(xxvi) 2AX6 (Androgen receptor ligand binding domain T877A mutant in complex with hydroxyflutamide)

Protein preparations:

Resolution: 1.50 Å

Ligand: HFT (Flutamide); $C_{11}H_{11}F_3N_2O_4$

Residue flipped: ASN692, GLN733, ASN756, ASN823, ASN833, GLN875

Protonation states (pH = 7.5):

HID689, HID714, HID729, HID776, HID789, HID874, HID885, HID917

Optimized region: HFT

High layer: HFT

Medium layer: WAT108, WAT213, LEU701, LEU704, ASN705, LEU707, GLY708, GLN711, MET742, MET745, VAL746, MET749, ARG752, PHE764, MET780, LEU873, PHE876, ALA877, MET895

$\omega_\alpha = 0.49350$

(a)

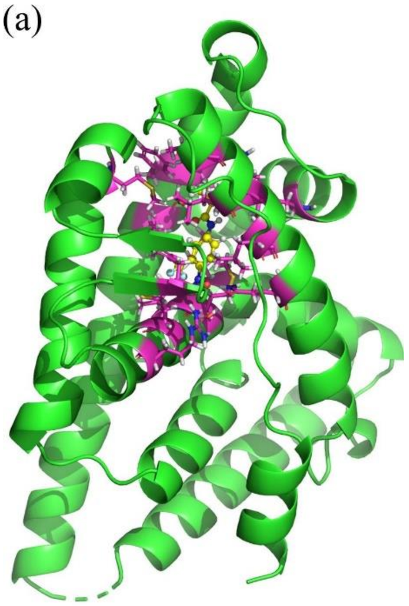

(b)

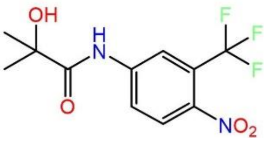

Supplementary Figure 153: Androgen receptor ligand binding domain T877A mutant in complex with hydroxyflutamide. (**a**) Crystal structure of androgen receptor ligand binding domain T877A mutant in complex with hydroxyflutamide (HFT). ONIOM layers by different colors: yellow: high layer; red: medium layer; green: low layer. Ligand hydroxyflutamide is presented in stick and balls. (**b**) Structure of hydroxyflutamide.

Quantum refined structural results:

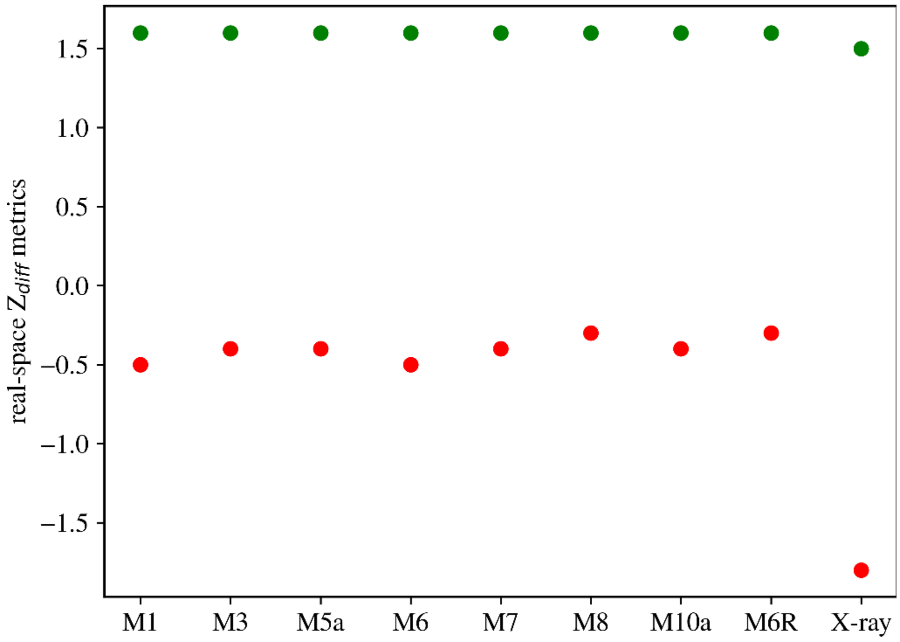

Supplementary Figure 154: Real-space Z-difference (RSZD) of hydroxyflutamide. RSZD+ (green) and RSZD- (red) scores of hydroxyflutamide (HFT) in androgen receptor various quantum refinement schemes (**M1-M10**). Those results for X-ray were taken from the experimental structure without our further refinement.

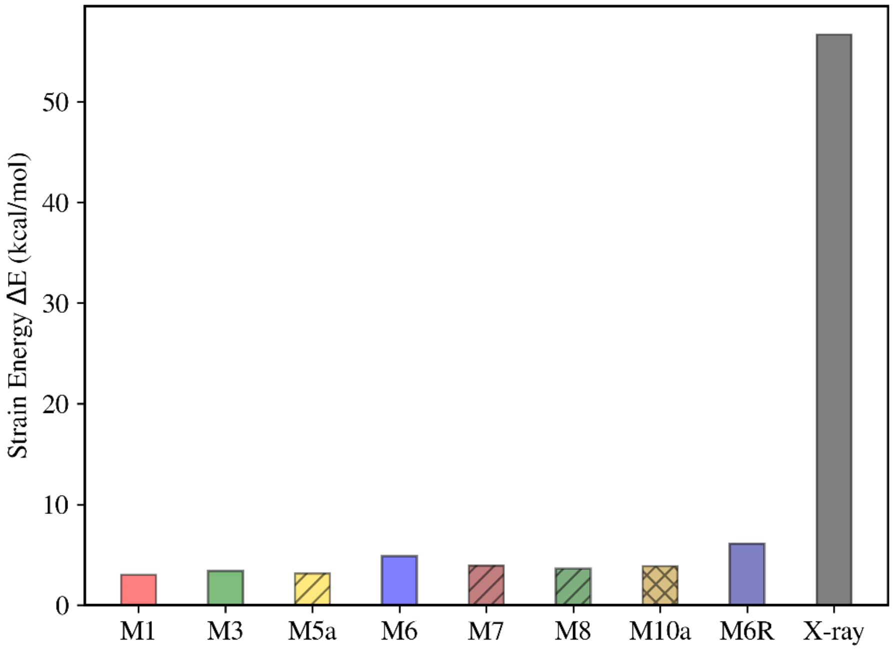

Supplementary Figure 155: Strain energy of hydroxyflutamide. Strain energy (ΔE, kcal·mol^{-1}) at ωB97X-D/6-31G(d) level for hydroxyflutamide (HFT) in androgen receptor determined by various quantum refinement schemes (**M1-M10**). Those results for X-ray were taken from the experimental structure without our further refinement.

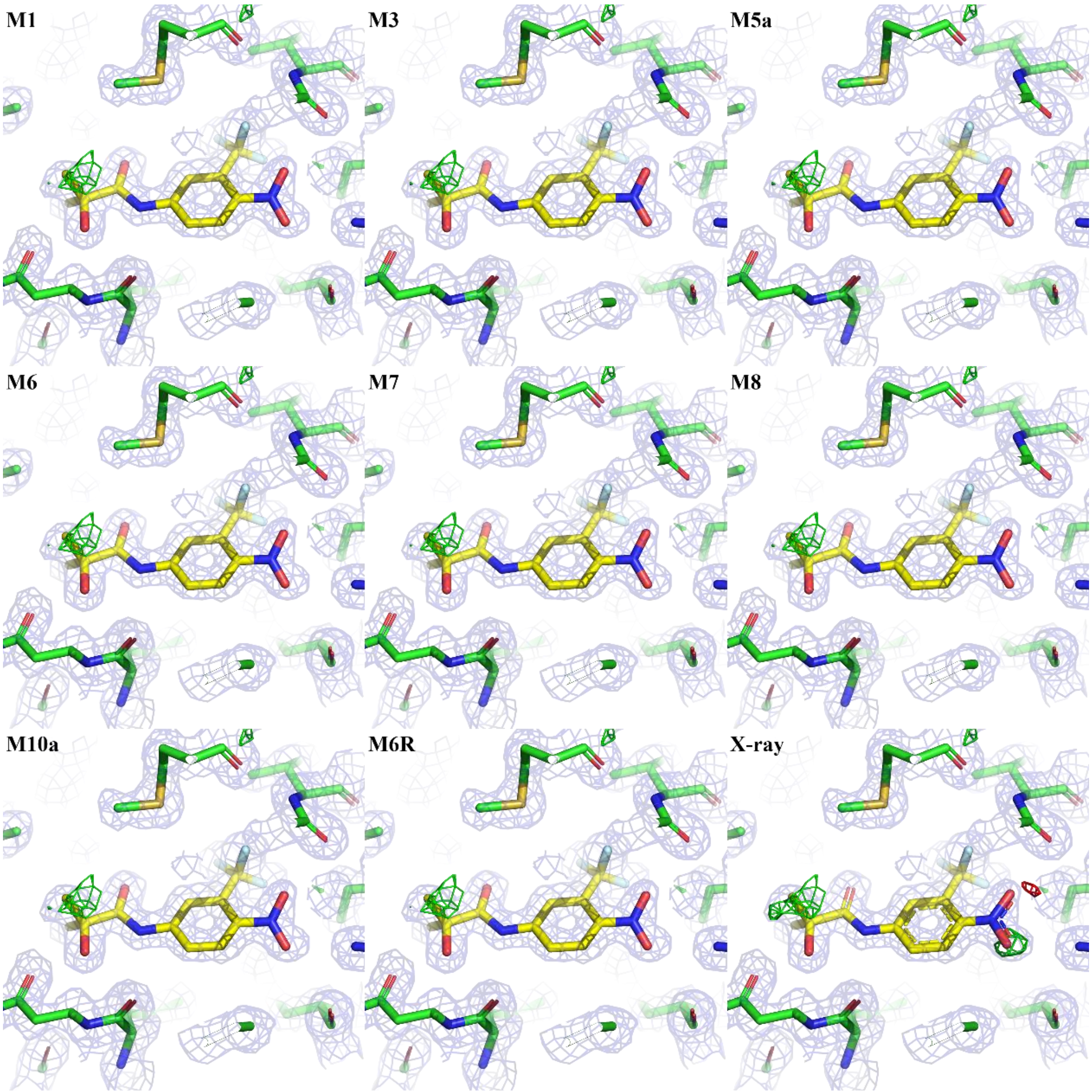

Supplementary Figure 156: Electron density maps of hydroxyflutamide. Structures for hydroxyflutamide (HFT) in androgen receptor from various quantum refinement schemes (**M1-M10**), including the electron density maps (2mFo-DFc maps, contoured at 1.0 σ (blue), mFo-DFc maps, contoured at +3.0 σ (green), and mFo-DFc maps, contoured at -3.0 σ (red)). Those results for X-ray were taken from the experimental structure without our further refinement.

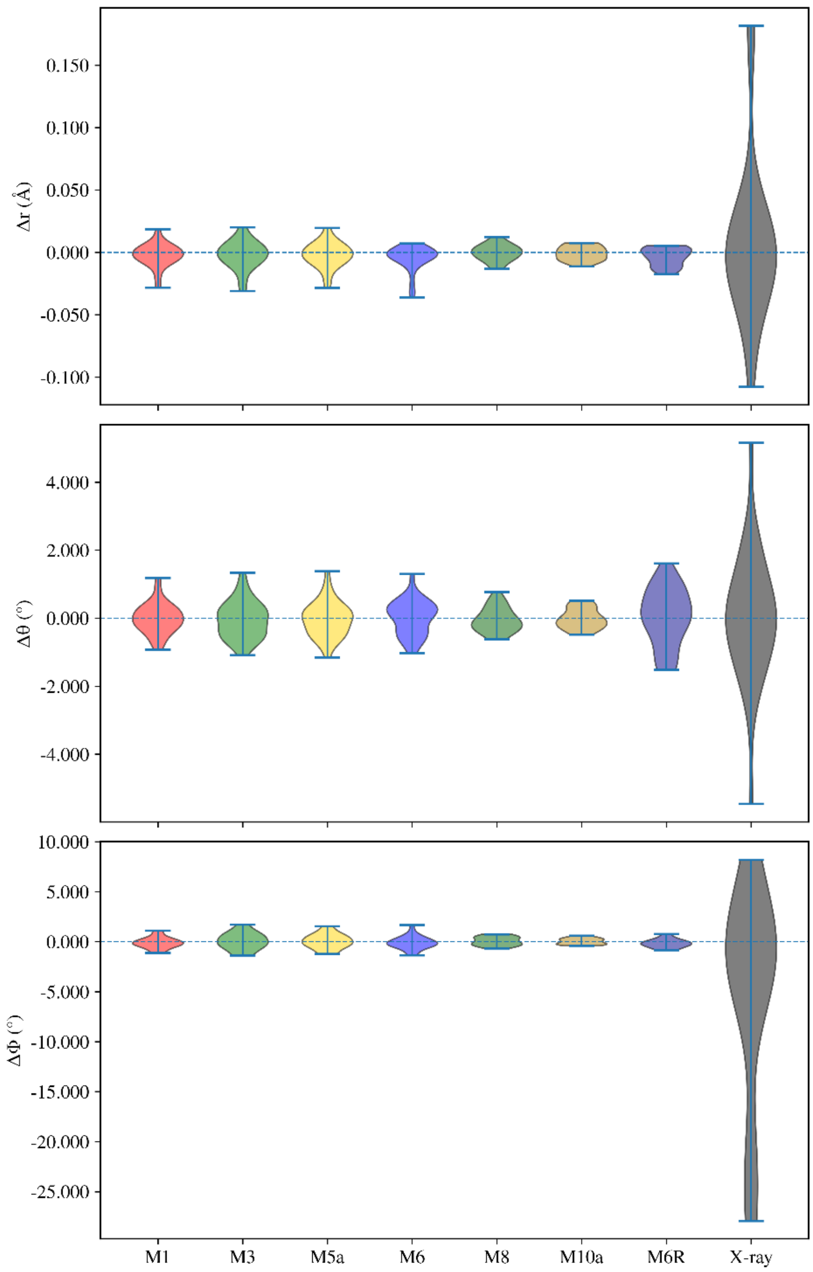

Supplementary Figure 157: Key coordinates of quantum refinement results of hydroxyflutamide. Deviation in the refined bond distances (Δr, n = 20), angles (Δθ, n = 31) and dihedrals (Δϕ, n = 20) of hydroxyflutamide (HFT) in androgen receptor from various quantum refinement schemes (**M1-M10**) and X-ray structure which are compared to those obtained from the most reliable **M7** scheme. The solid line represents the upper and lower values.

(xxvii) 2IWU (Analogues of radicicol bound to the ATP-binding site of HSP90)

Protein preparations:

Resolution: 2.80 Å

Ligand: NP5; $C_{16}H_{17}ClO_5$

Residue flipped: No

Protonation states (pH = 4.2):

HID197

Optimized region: NP5

High layer: NP5

Medium layer: ASN37, ASP40, ALA41, LYS44, ASP79, ILE82, MET84, LEU93, PHE124, THR171

ω_α = 3.0556

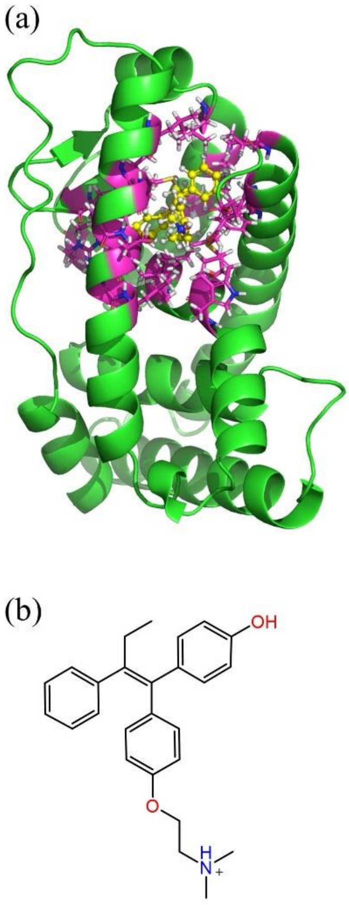

Supplementary Figure 158: Analogues of radicicol bound to HSP90. (**a**) Crystal structure of HSP90 complexed with analogues of radicicol (NP5). ONIOM layers by different colors: yellow: high layer; red: medium layer; green: low layer. Ligand analogues of radicicol is presented in stick and balls. (**b**) Structure of analogues of radicicol

Quantum refined structural results:

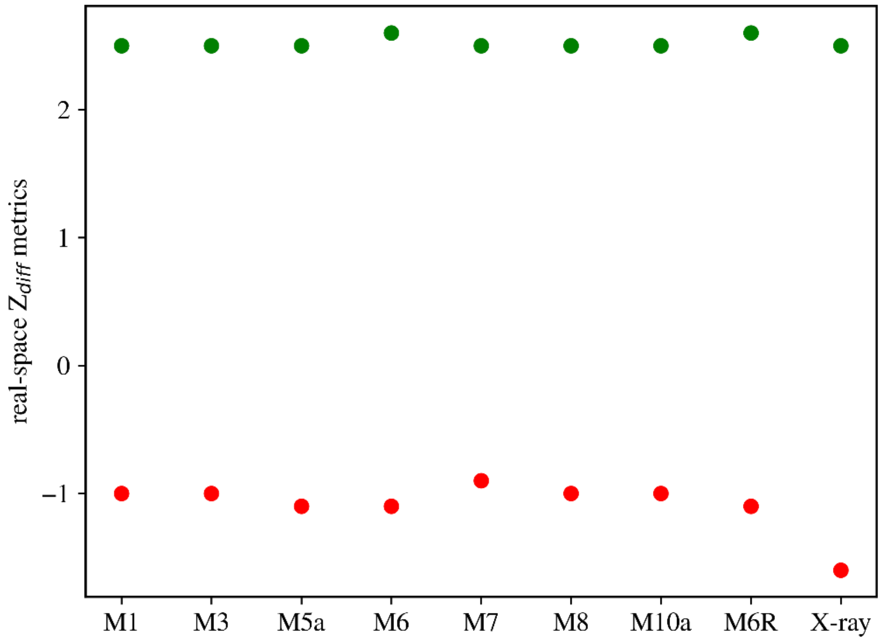

Supplementary Figure 159: Real-space Z-difference (RSZD) of analogues of radicicol. RSZD+ (green) and RSZD- (red) scores of analogues of radicicol (NP5) in HSP90 from various quantum refinement schemes (**M1-M10**). Those results for X-ray were taken from the experimental structure without our further refinement.

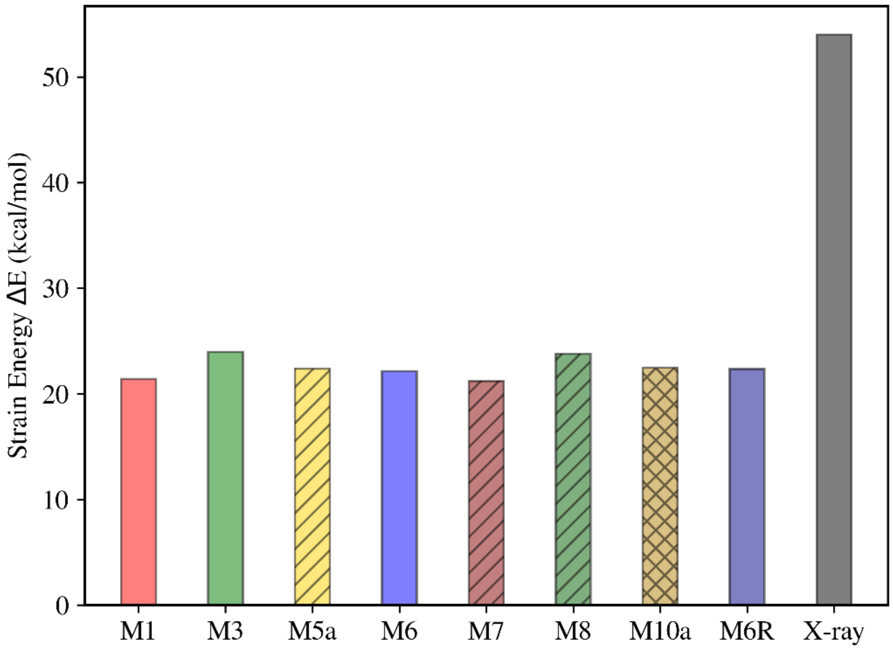

Supplementary Figure 160: Strain energy of analogues of radicicol. Strain energy (ΔE, kcal·mol^{-1}) at ωB97X-D/6-31G(d) level for analogues of radicicol (NP5) in HSP90 determined by various quantum refinement schemes (**M1-M10**). Those results for X-ray were taken from the experimental structure without our further refinement.

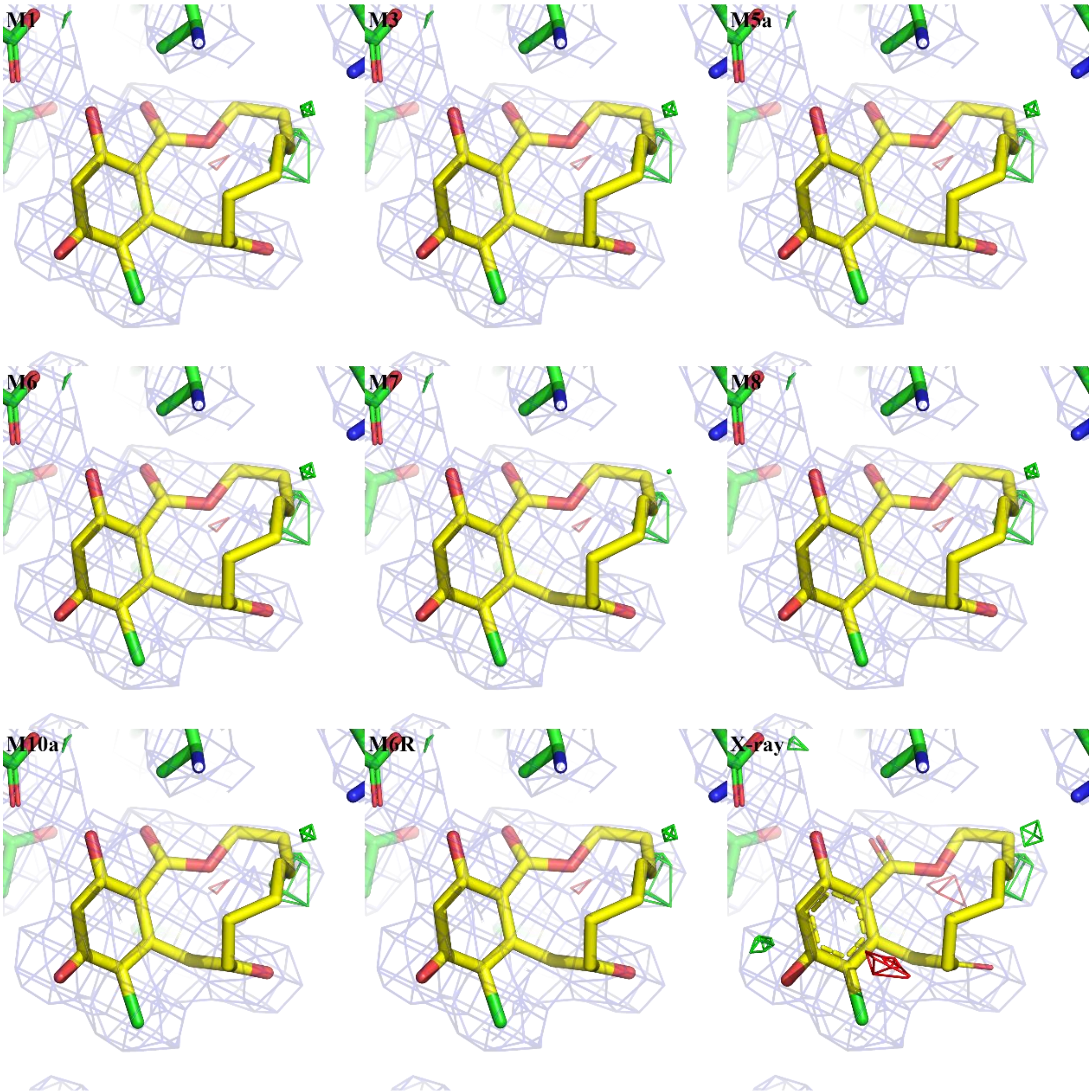

Supplementary Figure 161: Electron density maps of analogues of radicicol. Structures for analogues of radicicol (NP5) in HSP90 from various quantum refinement schemes (**M1-M10**), including the electron density maps (2mFo-DFc maps, contoured at 1.0 σ (blue), mFo-DFc maps, contoured at +3.0 σ (green), and mFo-DFc maps, contoured at -3.0 σ (red)). Those results for X-ray were taken from the experimental structure without our further refinement.

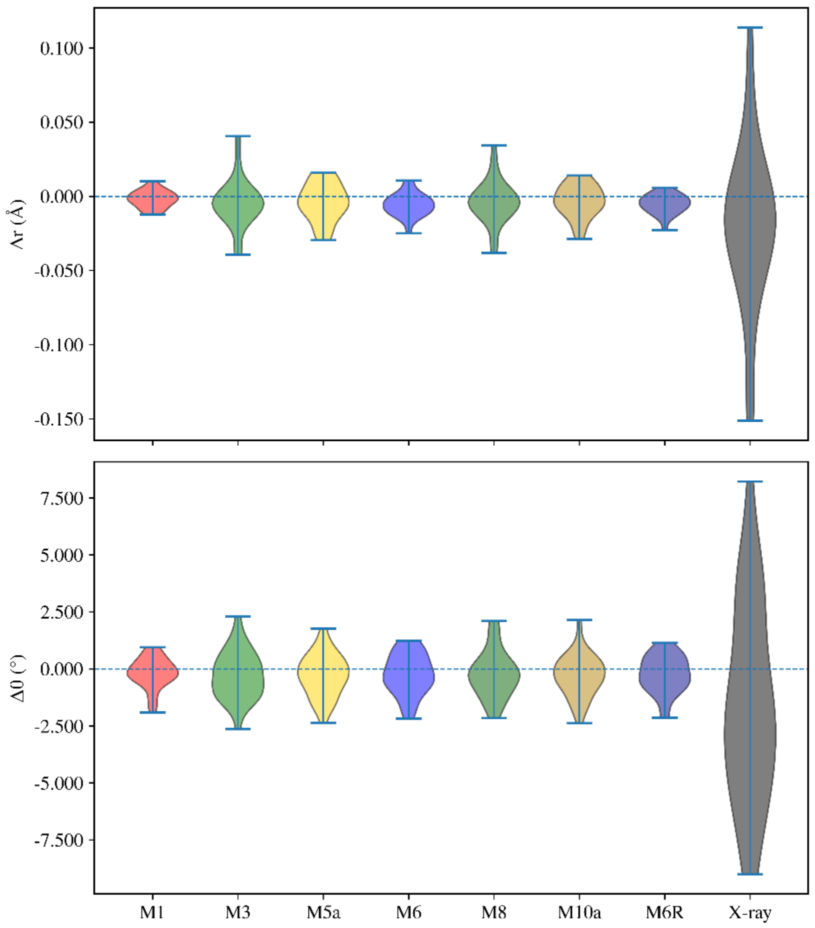

Supplementary Figure 162: Key coordinates of quantum refinement results of analogues of radicicol. Deviation in the refined bond distances (Δr, n = 23), angles (Δθ, n = 31) of analogues of radicicol (NP5) in HSP90 from various quantum refinement schemes (**M1-M10**) and X-ray structure which are compared to those obtained from the most reliable **M7** scheme. The solid line represents the upper and lower values.

(xxviii) 2P7Z (Estrogen related receptor gamma in complex with 4-hydroxy-tamoxifen)

Protein preparations:

Resolution: 2.50 Å

Ligand: OHT (Afimoxifene); $C_{26}H_{29}NO_2$

Residue flipped: GLN392, GLN433, HIS434

Protonation states (pH = 8.5):

HID240, HID285, HID381, HID396, HID407, HID434

Optimized region: OHT

High layer: OHT

Medium layer: LEU265, LEU268, CYS269, LEU271, ALA272, ASP273, GLU275, LEU276, TRP305, MET306, LEU309, ILE310, VAL313, ARG316, TYR326, LEU342, LEU345, ILE349, ALA431, PHE435, ILE438, GLU441, WAT525, WAT542

$\omega_\alpha = 1.3514$

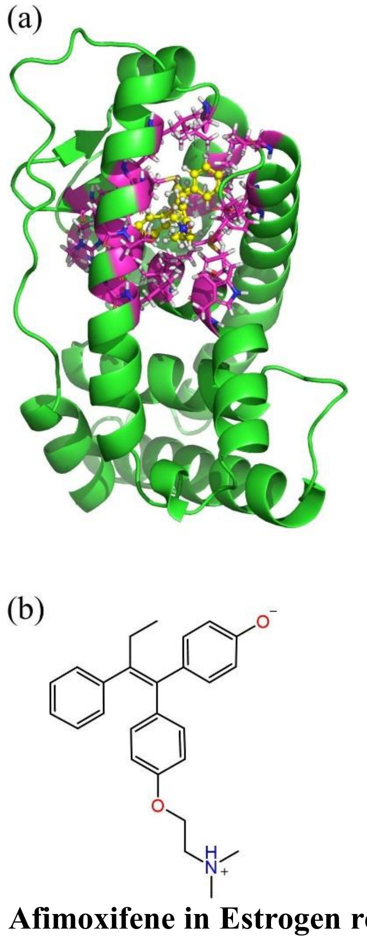

Supplementary Figure 163: Afimoxifene in Estrogen related receptor gamma. (**a**) Crystal structure of estrogen related receptor gamma complexed with afimoxifene (OHT). ONIOM layers by different colors: yellow: high layer; red: medium layer; green: low layer. Ligand afimoxifene is presented in stick and balls. (**b**) Structure of afimoxifene.

Quantum refined structural results:

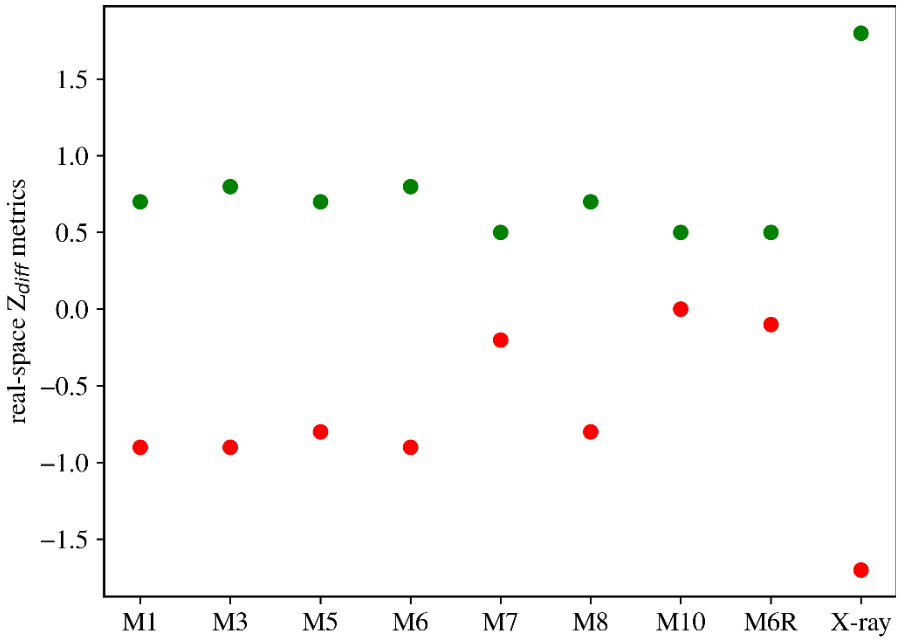

Supplementary Figure 164: Real-space Z-difference (RSZD) of afimoxifene. RSZD+ (green) and RSZD- (red) scores of afimoxifene (OHT) in estrogen related receptor gamma from various quantum refinement schemes (**M1-M10**). Those results for X-ray were taken from the experimental structure without our further refinement.

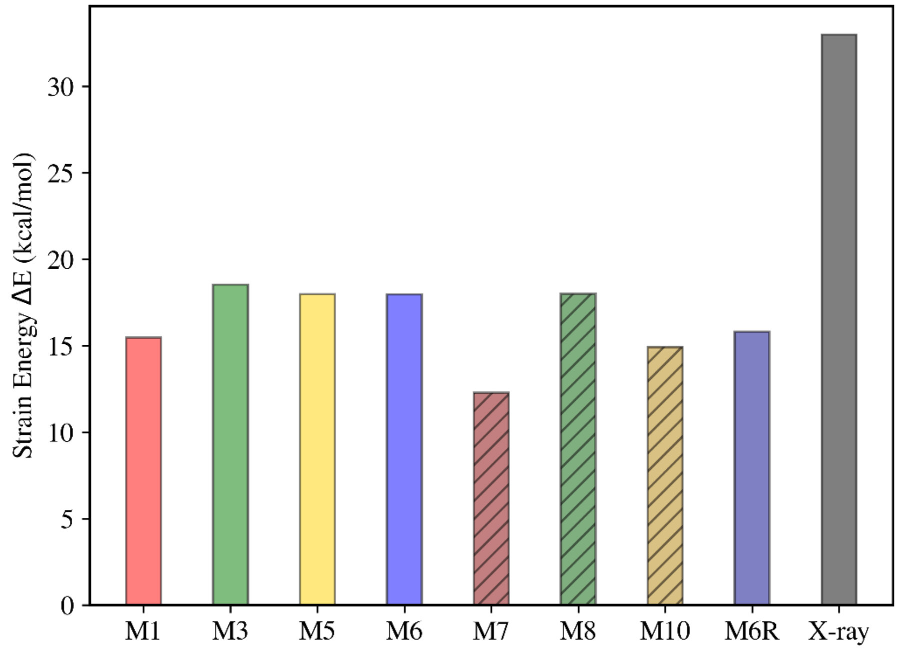

Supplementary Figure 165: Strain energy of afimoxifene. Strain energy (ΔE, kcal·mol^{-1}) at ωB97X-D/6-31G(d) level for afimoxifene (OHT) in estrogen related receptor gamma determined by various quantum refinement schemes (**M1-M10**). Those results for X-ray were taken from the experimental structure without our further refinement.

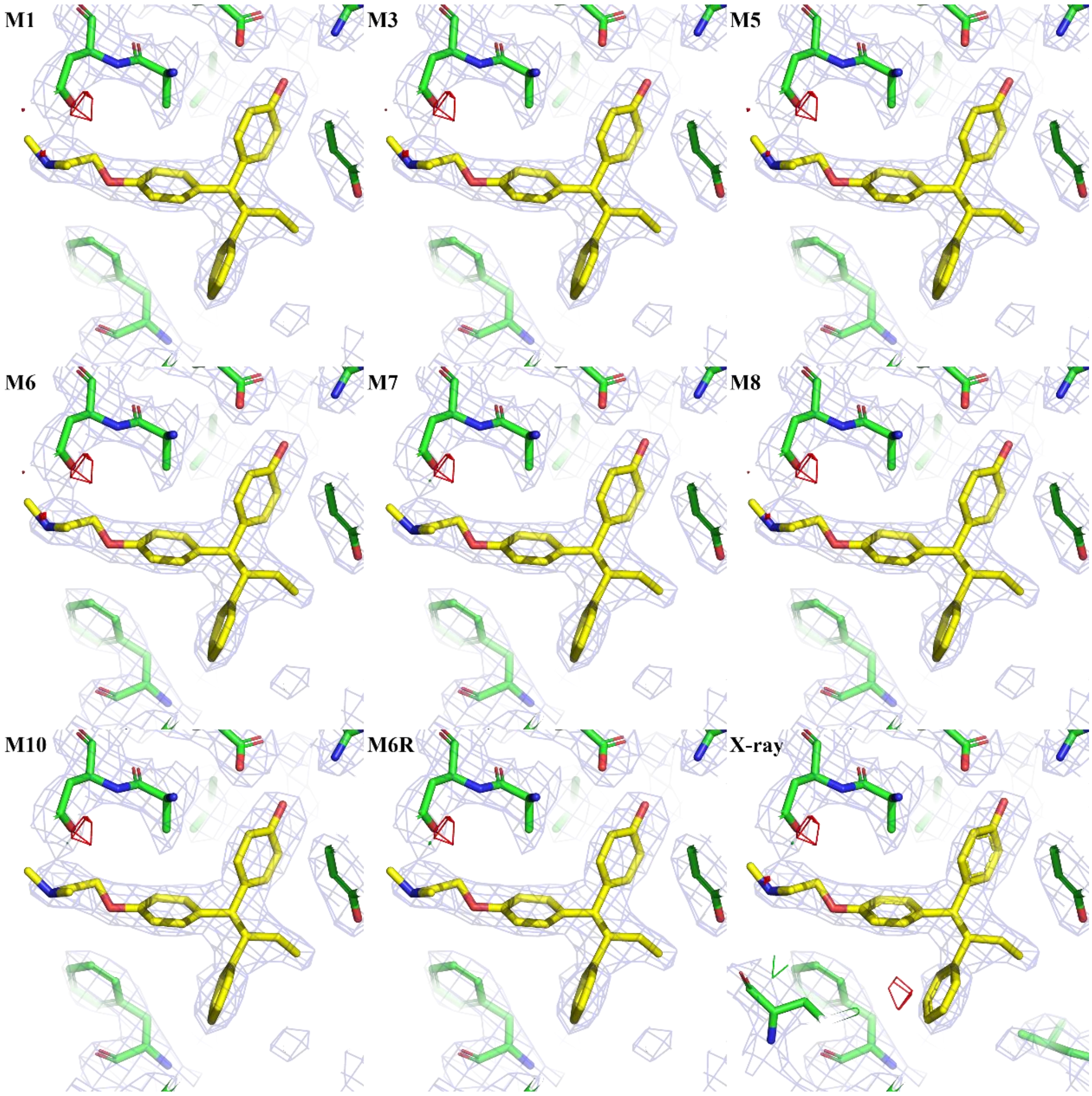

Supplementary Figure 166: Electron density maps of afimoxifene. Structures for afimoxifene (OHT) in estrogen related receptor gamma from various quantum refinement schemes (**M1**-**M10**), including the electron density maps (2mFo-DFc maps, contoured at 1.0 σ (blue), mFo-DFc maps, contoured at +3.0 σ (green), and mFo-DFc maps, contoured at -3.0 σ (red)). Those results for X-ray were taken from the experimental structure without our further refinement.

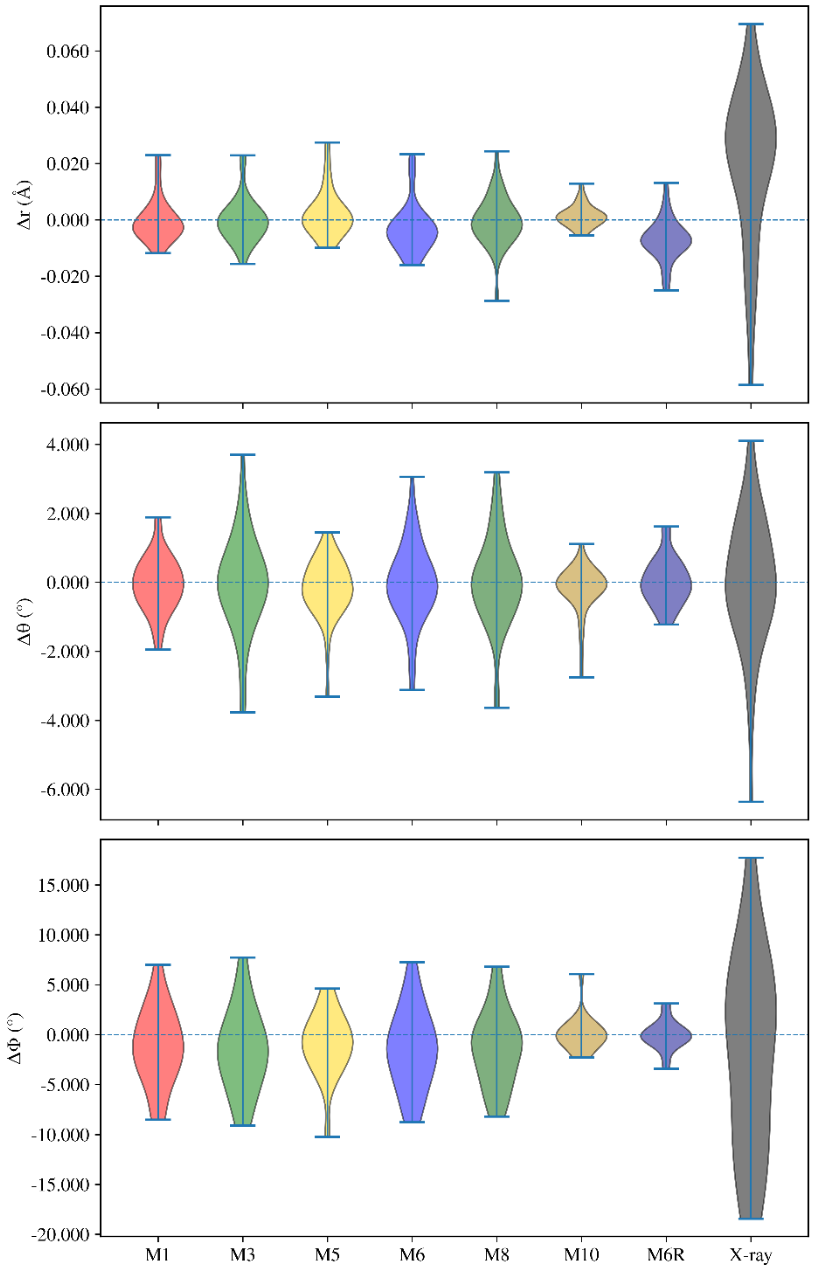

Supplementary Figure 167: Key coordinates of quantum refinement results of afimoxifene. Deviation in the refined bond distances (Δr, n = 31), angles (Δθ, n = 41) and dihedrals (Δϕ, n = 20) of afimoxifene (OHT) in estrogen related receptor gamma from various quantum refinement schemes (**M1-M10**) and X-ray structure which are compared to those obtained from the most reliable **M7** scheme. The solid line represents the upper and lower values.

(xxix) 1UY9 (Human HSP90-alpha with 8-Benzo[1,3]dioxol-,5-ylmethyl-9-butyl-9H-purin-6-ylamine)

Protein preparations:

Resolution: 2.00 Å

Ligand: PU6; $C_{17}H_{19}N_5O_2$

Residue flipped: ASN51, GLN85, HIS154, GLN194

Protonation states (pH = 6.5):

HID77, ASH93, HID154, HID189, HID210

Optimized region: PU6

High layer: PU6

Medium layer: ASN51, SER52, ALA55, ASH93, ILE96, MET98, LEU103, LEU107, PHE138, TYR139, VAL150, TRP162, THR184, WAT2024, WAT2058, WAT2060, WAT2061, WAT2062, WAT2066, WAT2067, WAT2089, WAT2135, WAT2144, WAT2145, WAT2172, WAT2175, WAT2176, WAT2274

$\omega_\alpha = 0.42433$

(a)

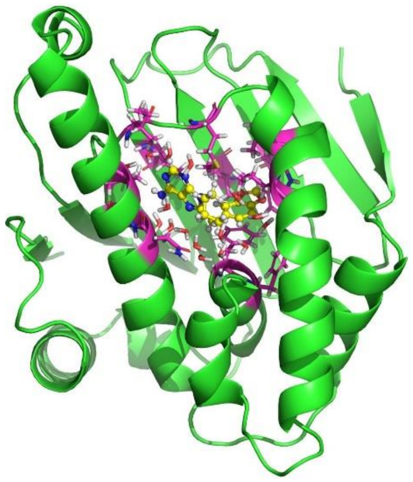

(b)

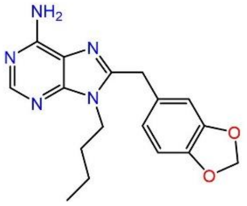

Supplementary Figure 168: Human HSP90-alpha with 8-Benzo[1,3]dioxol-,5-ylmethyl-9-butyl-9H-purin-6-ylamine. (**a**) Crystal structure of human HSP90-alpha complexed with inhibitor PU6. ONIOM layers by different colors: yellow: high layer; red: medium layer; green: low layer. Ligand inhibitor PU6 is presented in stick and balls. (**b**) Structure of inhibitor PU6.

Quantum refined structural results:

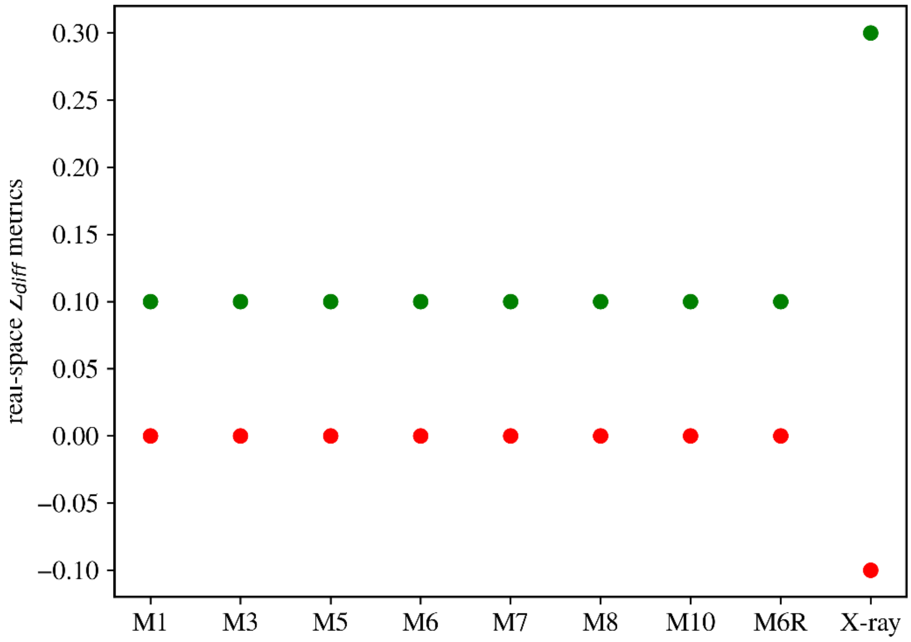

Supplementary Figure 169: Real-space Z-difference (RSZD) of inhibitor PU6. RSZD+ (green) and RSZD- (red) scores of inhibitor PU6 in human HSP90-alpha from various quantum refinement schemes (**M1-M10**). Those results for X-ray were taken from the experimental structure without our further refinement.

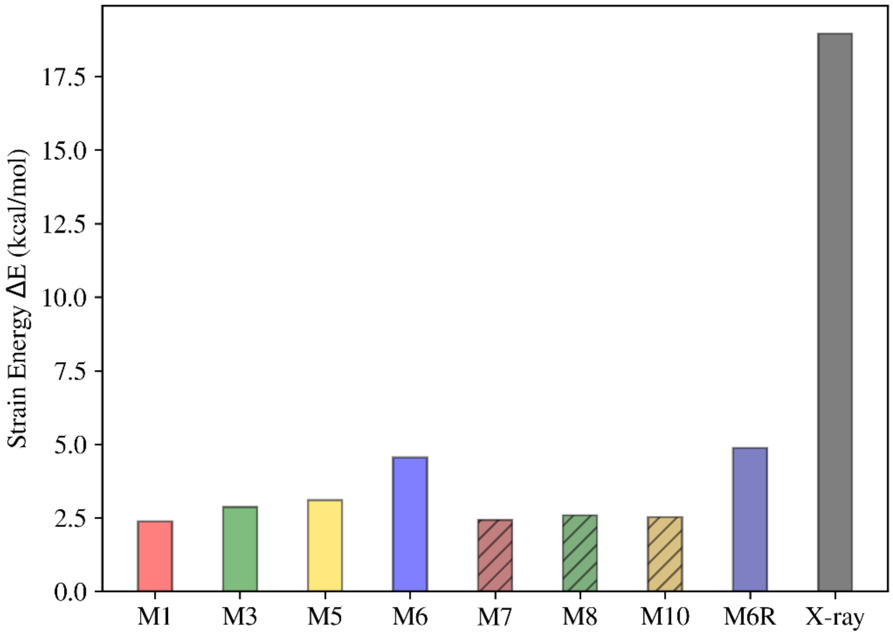

Supplementary Figure 170: Strain energy of inhibitor PU6. Strain energy (ΔE, kcal·mol^{-1}) at ωB97X-D/6-31G(d) level inhibitor PU6 in human HSP90-alpha determined by various quantum refinement schemes (**M1-M10**). Those results for X-ray were taken from the experimental structure without our further refinement.

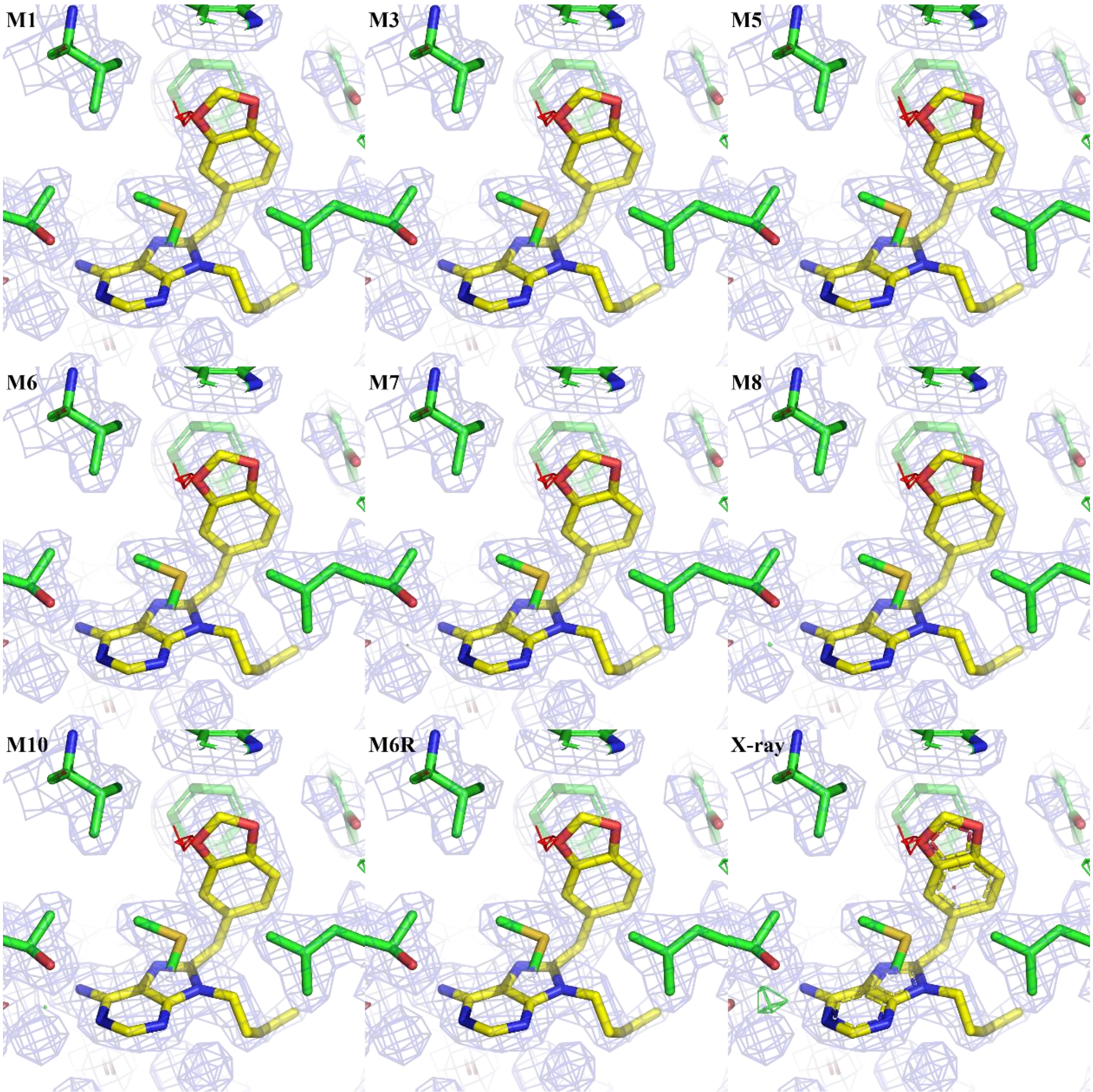

Supplementary Figure 171: Electron density maps of inhibitor PU6. Structures for inhibitor PU6 in human HSP90-alpha from various quantum refinement schemes (**M1-M10**), including the electron density maps (2mFo-DFc maps, contoured at 1.0 σ (blue), mFo-DFc maps, contoured at +3.0 σ (green), and mFo-DFc maps, contoured at -3.0 σ (red)). Those results for X-ray were taken from the experimental structure without our further refinement.

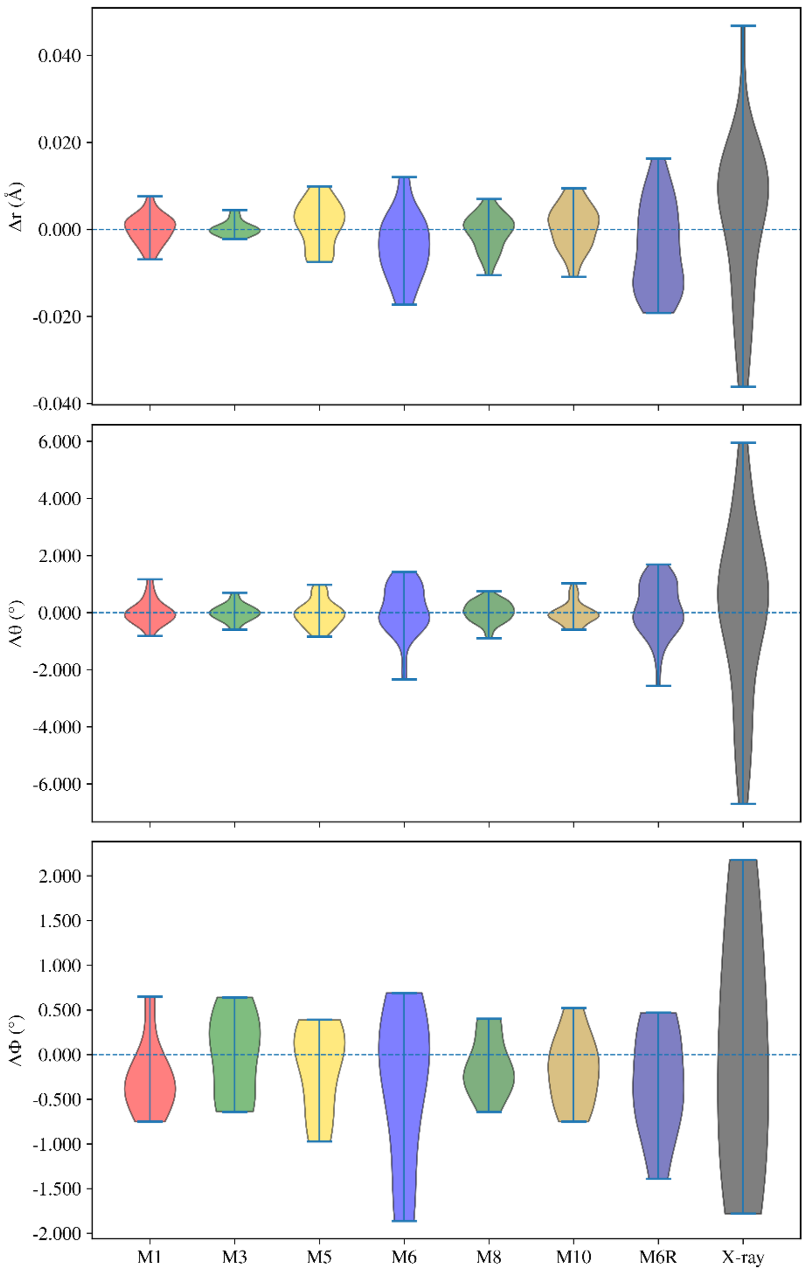

Supplementary Figure 172: Key coordinates of quantum refinement results of inhibitor PU6. Deviation in the refined bond distances (Δr, n = 27), angles (Δθ, n = 38) and dihedrals (Δϕ, n = 8) of inhibitor PU6 in human HSP90-alpha from various quantum refinement schemes (**M1-M10**) and X-ray structure which are compared to those obtained from the most reliable **M7** scheme. The solid line represents the upper and lower values.

(xxx) 3EQ7 (Prolyl oligopeptidase complexed with R-Pro-(decarboxy-Pro)-Type inhibitors)

Protein preparations:

Resolution: 2.89 Å

Ligand: X99; $C_{20}H_{21}F_2N_3O_4$

Residue flipped: ASN310, ASN347, ASN477, ASN555, HIS587, HIS593

Protonation states (pH = 5.5):

HID20, HID22, LYN75, HID79, HID180, HID207, HID213, HID307, HID333, HID355, HID364, HID409, HID456, HID466, HID494, HIP515, HID587, HID593, HID607, HID618, HID640, HID648, HID673, HID680, CYM703

Optimized region: X99

High layer: X99

Medium layer: PHE173, TYR473, PHE476, ILE478, SER554, ASN555, VAL580, ILE591, TRP595, TYR599, ARG643, VAL644

ω_α = 2.9971

(a)

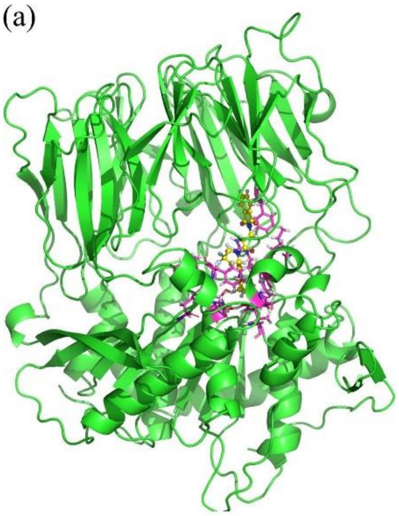

(b)

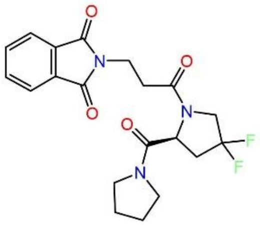

Supplementary Figure 173: Prolyl oligopeptidase complexed with R-Pro-(decarboxy-Pro)-Type inhibitors. (**a**) Crystal structure of prolyl oligopeptidase complexed with inhibitor X99. ONIOM layers by different colors: yellow: high layer; red: medium layer; green: low layer. Ligand inhibitor X99 is presented in stick and balls. (**b**) Structure of inhibitor X99.

Quantum refined structural results:

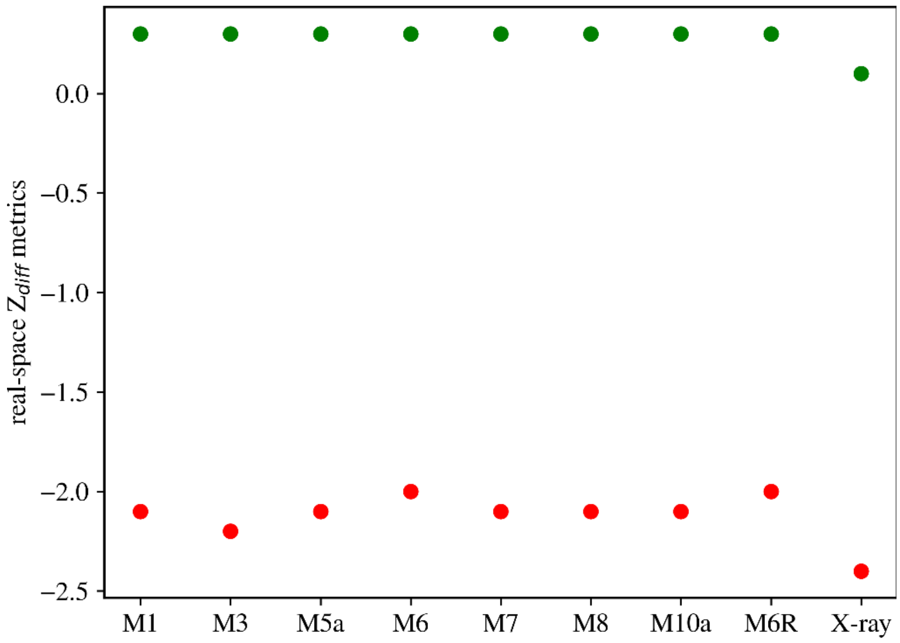

Supplementary Figure 174: Real-space Z-difference (RSZD) of inhibitor X99. RSZD+ (green) and RSZD- (red) scores of inhibitor X99 in prolyl oligopeptidase from various quantum refinement schemes (**M1-M10**). Those results for X-ray were taken from the experimental structure without our further refinement.

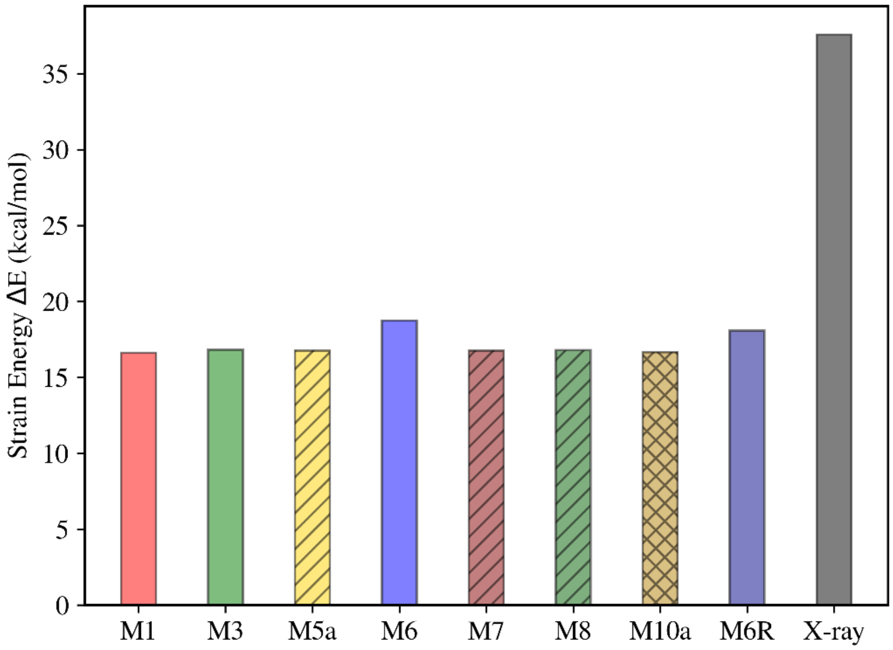

Supplementary Figure 175: Strain energy of inhibitor X99. Strain energy (ΔE, kcal·mol^{-1}) at ωB97X-D/6-31G(d) level for inhibitor X99 in prolyl oligopeptidase determined by various quantum refinement schemes (**M1-M10**). Those results for X-ray were taken from the experimental structure without our further refinement.

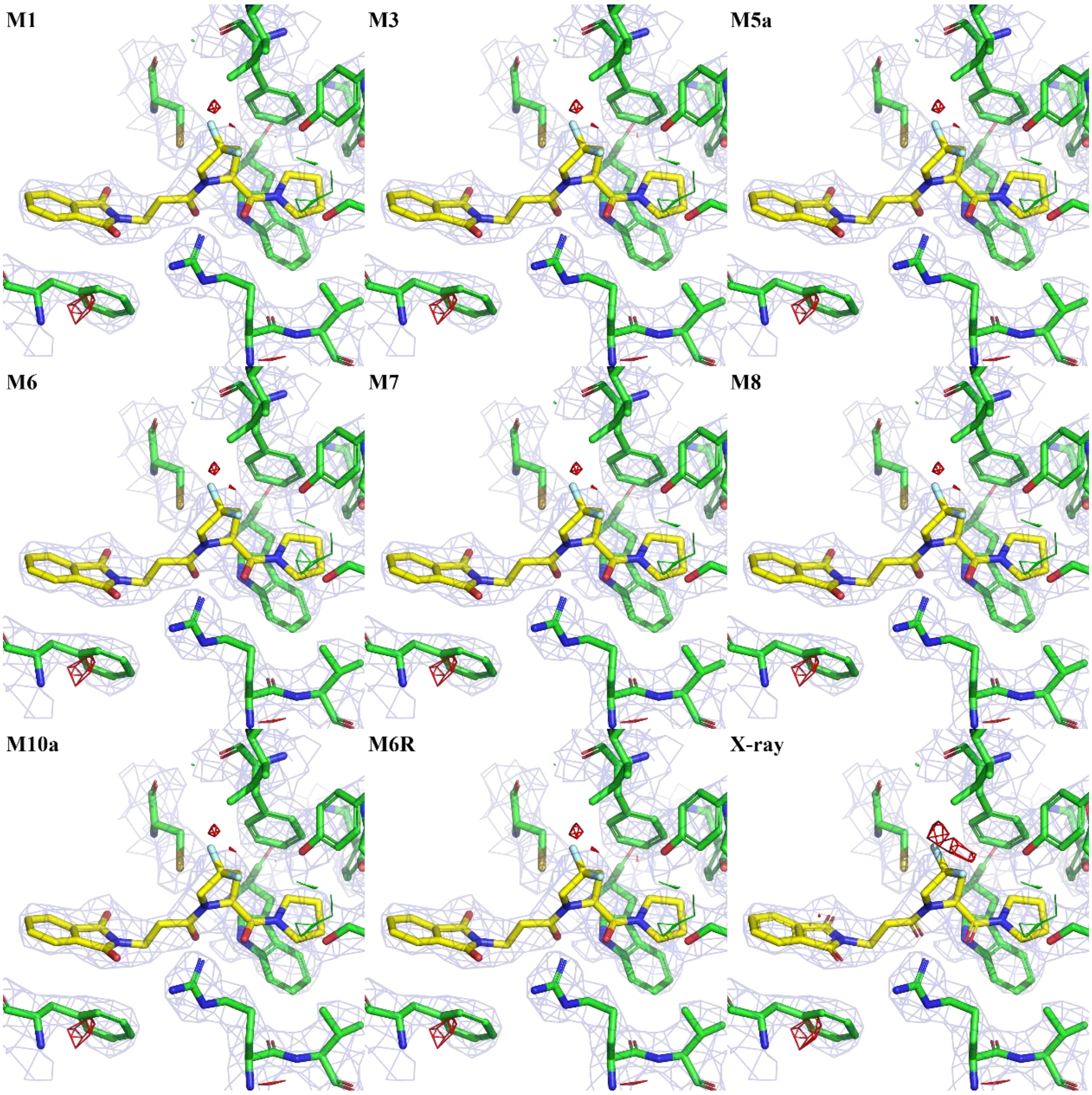

Supplementary Figure 176: Electron density maps of inhibitor X99. Structures for inhibitor X99 in prolyl oligopeptidase from various quantum refinement schemes (**M1-M10**), including the electron density maps (2mFo-DFc maps, contoured at 1.0 σ (blue), mFo-DFc maps, contoured at +3.0 σ (green), and mFo-DFc maps, contoured at -3.0 σ (red)). Those results for X-ray were taken from the experimental structure without our further refinement.

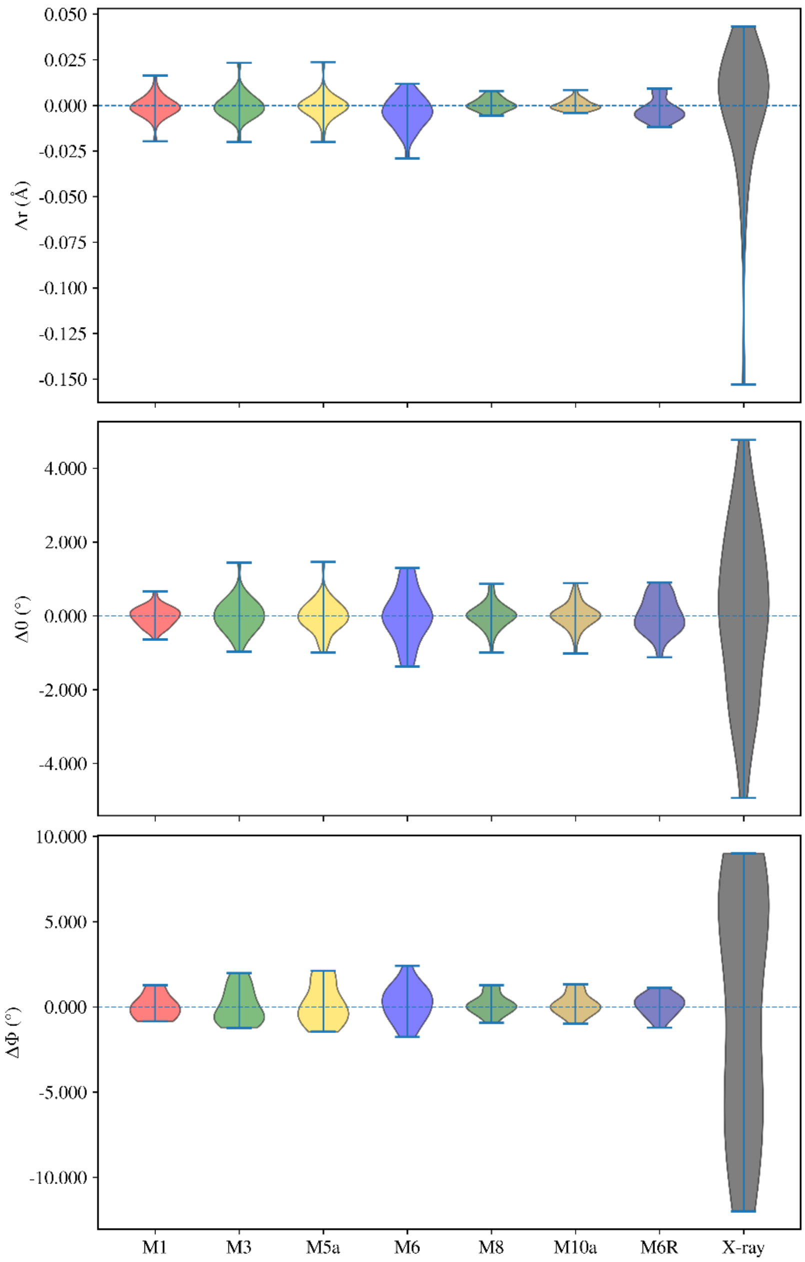

Supplementary Figure 177: Key coordinates of quantum refinement results of inhibitor X99. Deviation in the refined bond distances (Δr, n = 32), angles (Δθ, n = 48) and dihedrals (Δϕ, n = 17) of inhibitor X99 in prolyl oligopeptidase from various quantum refinement schemes (**M1-M10**) and X-ray structure which are compared to those obtained from the most reliable **M7** scheme. The solid line represents the upper and lower values.

(xxxi) 5Y1Y (Complex structure of nitroxoline with the first bromodomain of BRD4)

Protein preparations:

Resolution: 1.91 Å

Ligand: HNQ (Nitroxoline); $C_9H_6N_2O_3$

Residue flipped: ASN117

Protonation states (pH = 7.4):

HID77

Optimized region: HNQ

High layer: HNQ

Medium layer: PRO82, VAL87, LEU92, LEU94, CYS136, TYR139, ASN140, ILE146, WAT307, WAT329, WAT332

ω_α = 1.4873

(a)

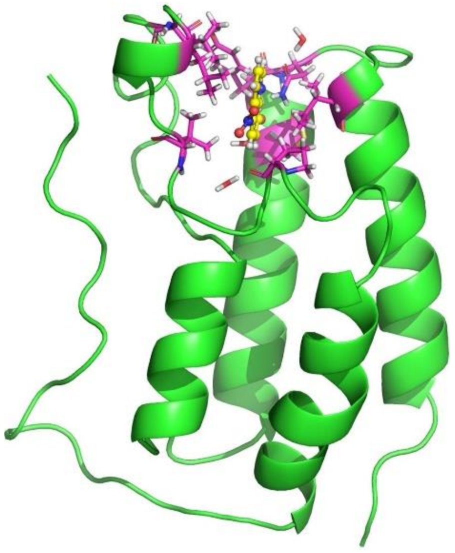

(b)

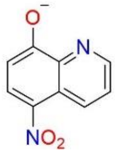

Supplementary Figure 178: Nitroxoline in bromodomain of BRD4. (**a**) Crystal structure of bromodomain of BRD4 complexed with nitroxoline (HNQ). ONIOM layers by different colors: yellow: high layer; red: medium layer; green: low layer. Ligand nitroxoline is presented in stick and balls. (**b**) Structure of nitroxoline.

Quantum refined structural results:

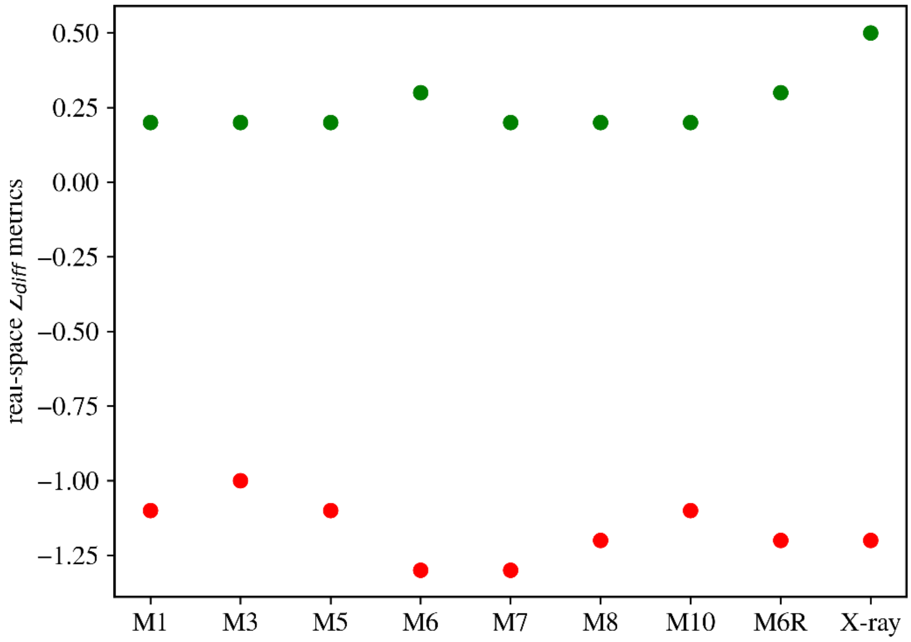

Supplementary Figure 179: Real-space Z-difference (RSZD) of nitroxoline. RSZD+ (green) and RSZD- (red) scores of nitroxoline (HNQ) in BRD4 from various quantum refinement schemes (**M1-M10**). Those results for X-ray were taken from the experimental structure without our further refinement.

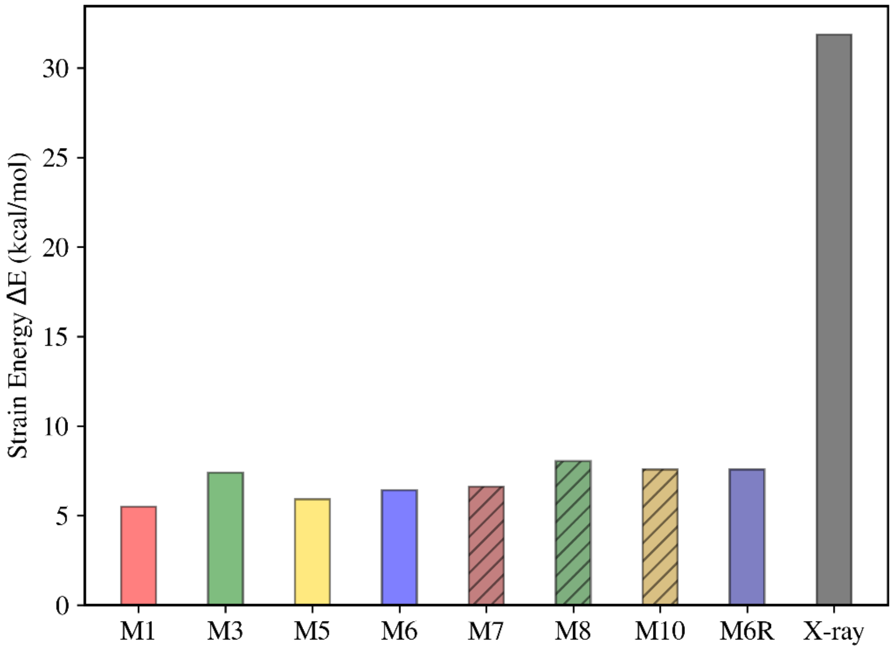

Supplementary Figure 180: Strain energy of nitroxoline. Strain energy (ΔE, kcal·mol^{-1}) at ωB97X-D/6-31G(d) level for nitroxoline (HNQ) in BRD4 determined by various quantum refinement schemes (**M1-M10**). Those results for X-ray were taken from the experimental structure without our further refinement.

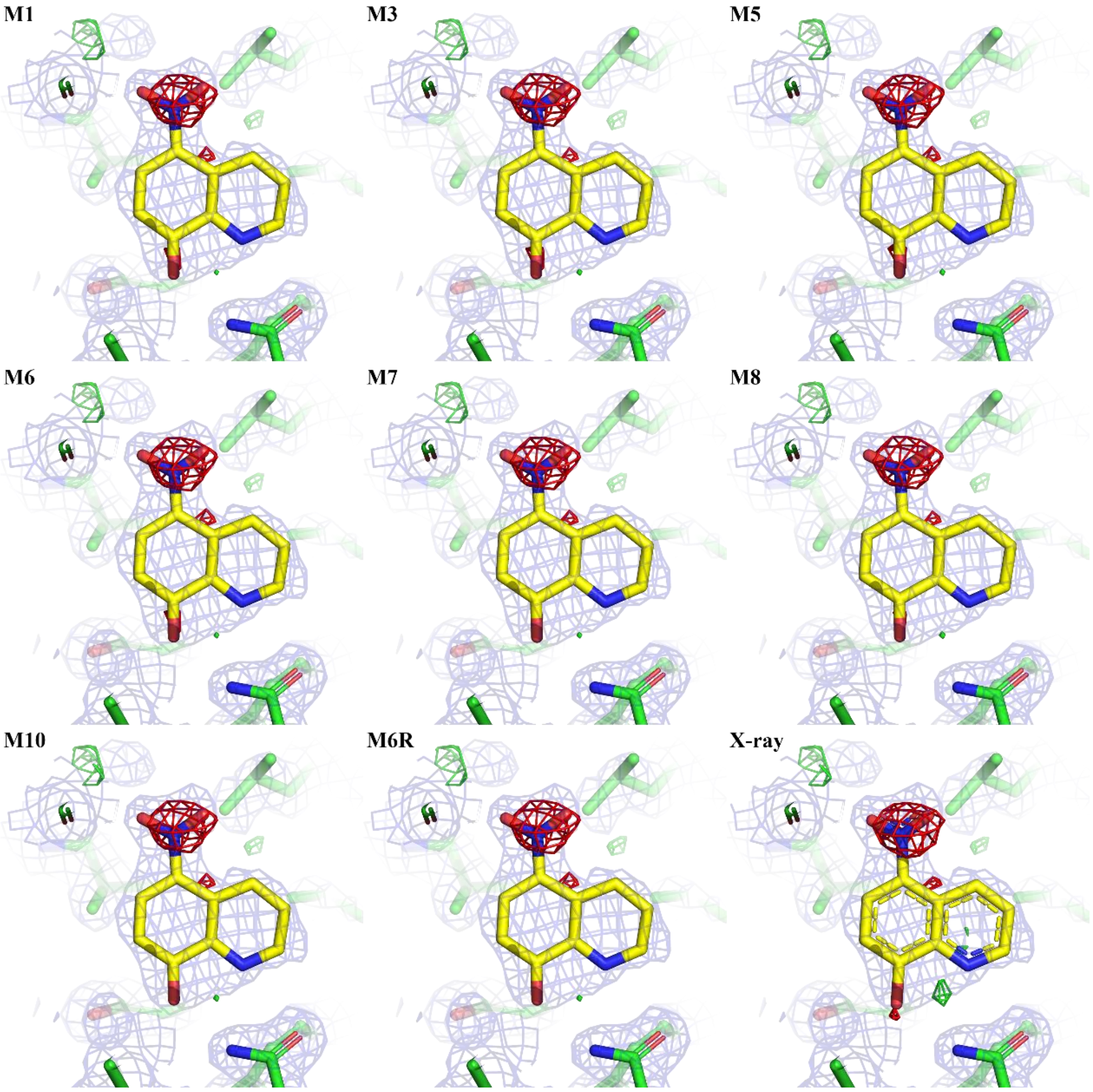

Supplementary Figure 181: Electron density maps of nitroxoline. Structures for nitroxoline (HNQ) in BRD4 from various quantum refinement schemes (**M1**-**M10**), including the electron density maps (2mFo-DFc maps, contoured at 1.0 σ (blue), mFo-DFc maps, contoured at +3.0 σ (green), and mFo-DFc maps, contoured at -3.0 σ (red)). Those results for X-ray were taken from the experimental structure without our further refinement.

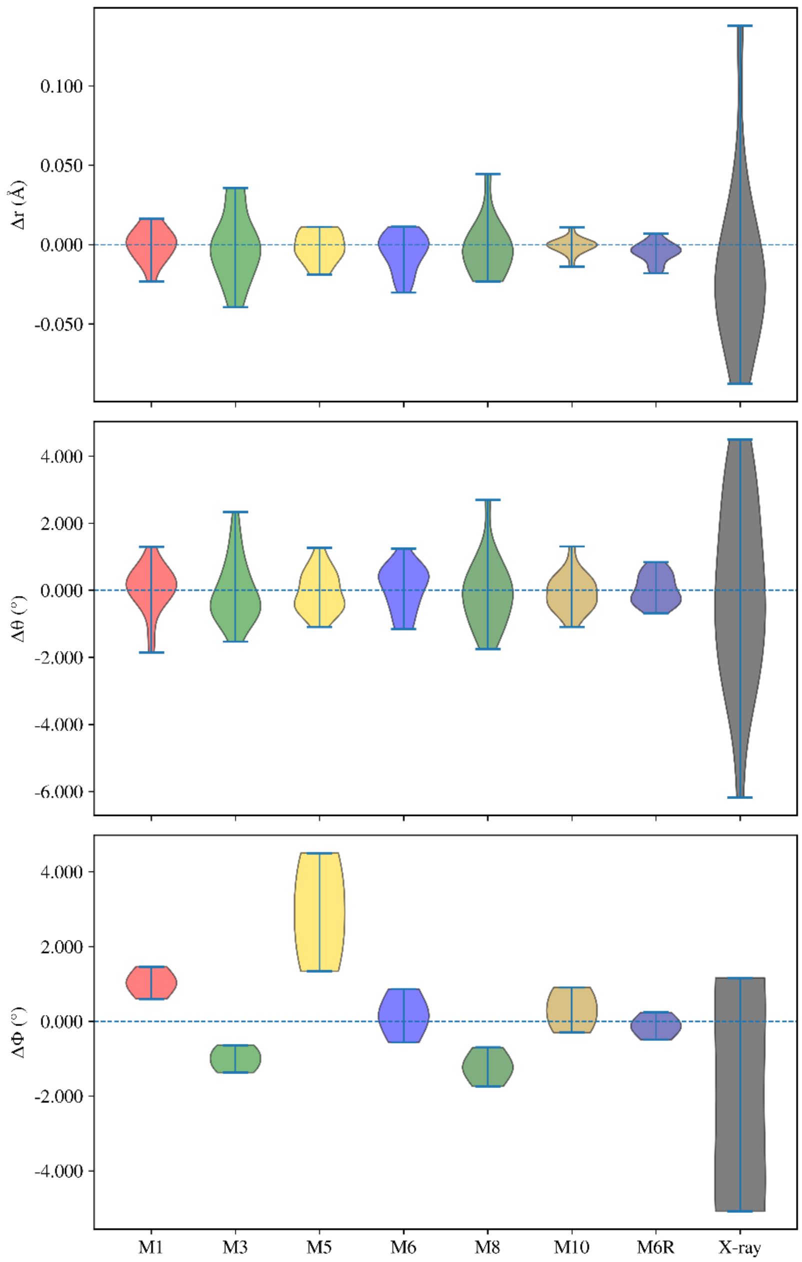

Supplementary Figure 182: Key coordinates of quantum refinement results of nitroxoline. Deviation in the refined bond distances (Δr, n = 15), angles (Δθ, n = 21) and dihedrals (Δϕ, n = 4) of nitroxoline (HNQ) in BRD4 from various quantum refinement schemes (**M1-M10**) and X-ray structure which are compared to those obtained from the most reliable **M7** scheme. The solid line represents the upper and lower values.

(xxxii) 5Y62 (YfiR complexed with GMP)

Protein preparations:

Resolution: 2.50 Å

Ligand: 5GP (Guanosine-5'-Monophosphate); $C_{10}H_{14}N_5O_8P$

Residue flipped: Chain B: HIS95

Protonation states (pH = 5.5):

Chain A: HID95, HID133, HID177

Chain B: HID95, HID133, HID177

Optimized region: 5GP

High layer: 5GP

Medium layer: Chain B: ARG60, ARG175, HID177

ω_α = 1.8041

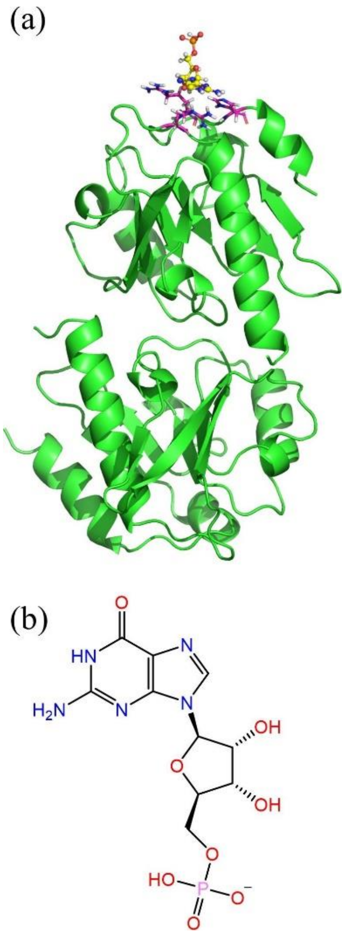

Supplementary Figure 183: YfiR complexed with GMP. (**a**) Crystal structure of YfiR complexed with guanosine-5'-monophosphate (5GP). ONIOM layers by different colors: yellow: high layer; red: medium layer; green: low layer. Ligand guanosine-5'-monophosphate is presented in stick and balls. (**b**) Structure of guanosine-5'-monophosphate.

Quantum refined structural results:

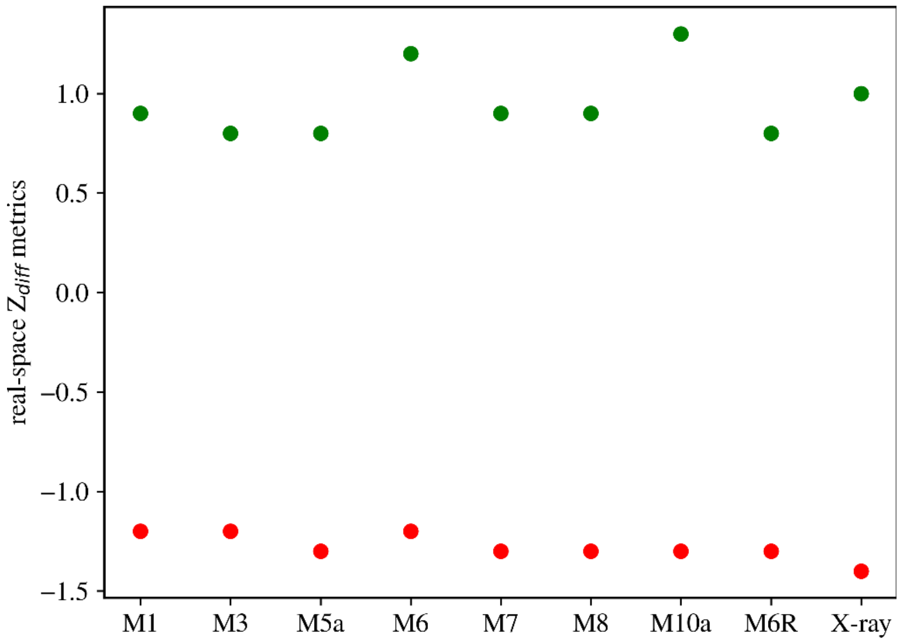

Supplementary Figure 184: Real-space Z-difference (RSZD) of guanosine-5'-monophosphate. RSZD+ (green) and RSZD- (red) scores of guanosine-5'-monophosphate (5GP) in YfiR from various quantum refinement schemes (**M1**-**M10**). Those results for X-ray were taken from the experimental structure without our further refinement.

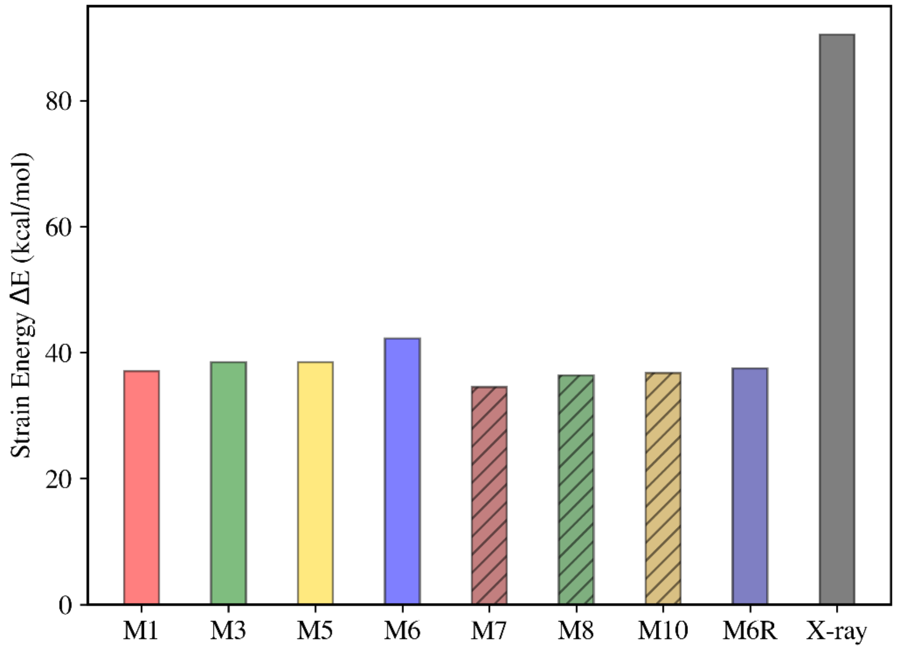

Supplementary Figure 185: Strain energy of guanosine-5'-monophosphate. Strain energy (ΔE, kcal·mol^{-1}) at ωB97X-D/6-31G(d) level for guanosine-5'-monophosphate (5GP) in YfiR determined by various quantum refinement schemes (**M1**-**M10**). Those results for X-ray were taken from the experimental structure without our further refinement.

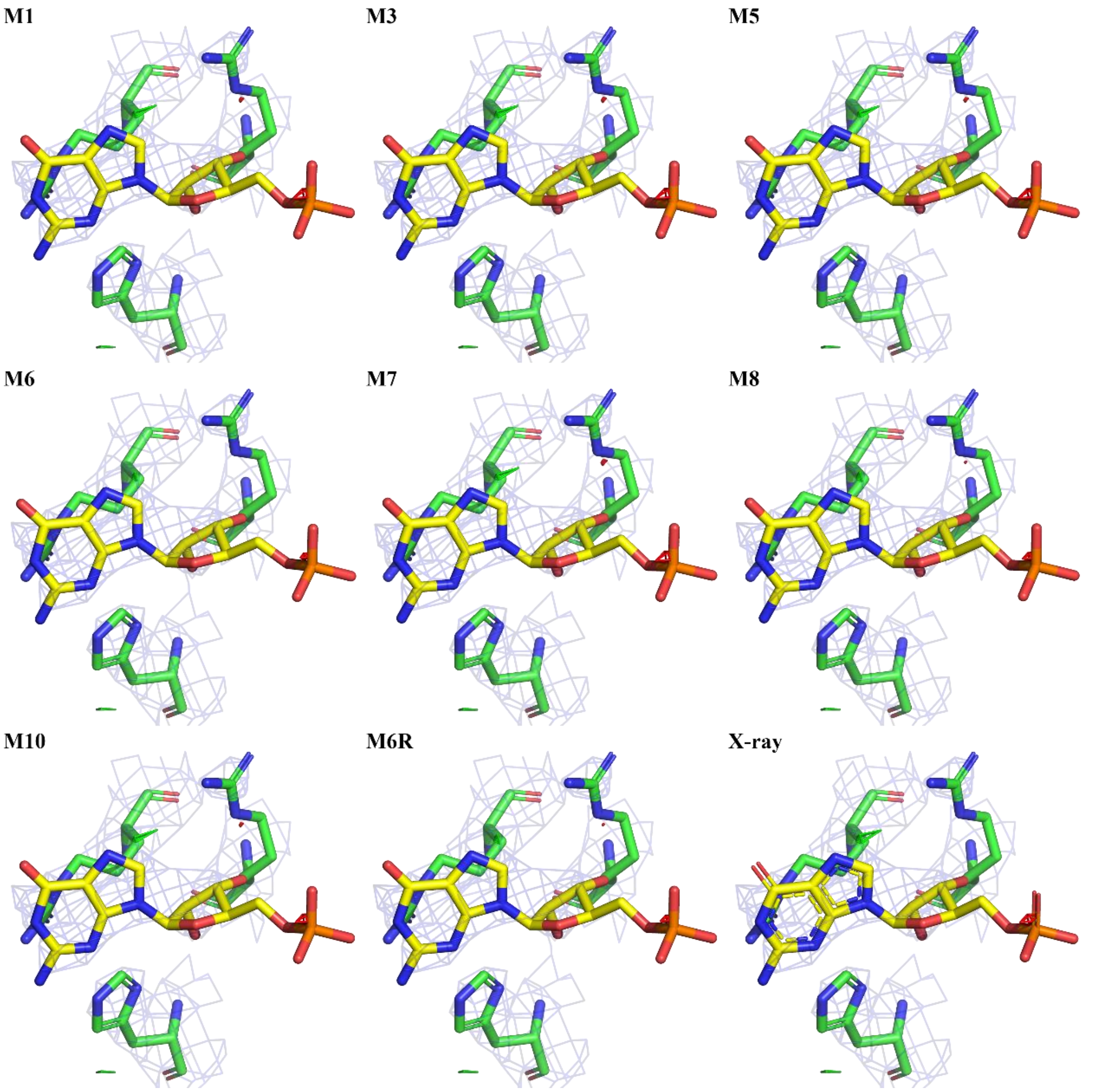

Supplementary Figure 186: Electron density maps of guanosine-5'-monophosphate. Structures for guanosine-5'-monophosphate (5GP) in YfiR from various quantum refinement schemes (**M1-M10**), including the electron density maps (2mFo-DFc maps, contoured at 1.0 σ (blue), mFo-DFc maps, contoured at +3.0 σ (green), and mFo-DFc maps, contoured at -3.0 σ (red)). Those results for X-ray were taken from the experimental structure without our further refinement.

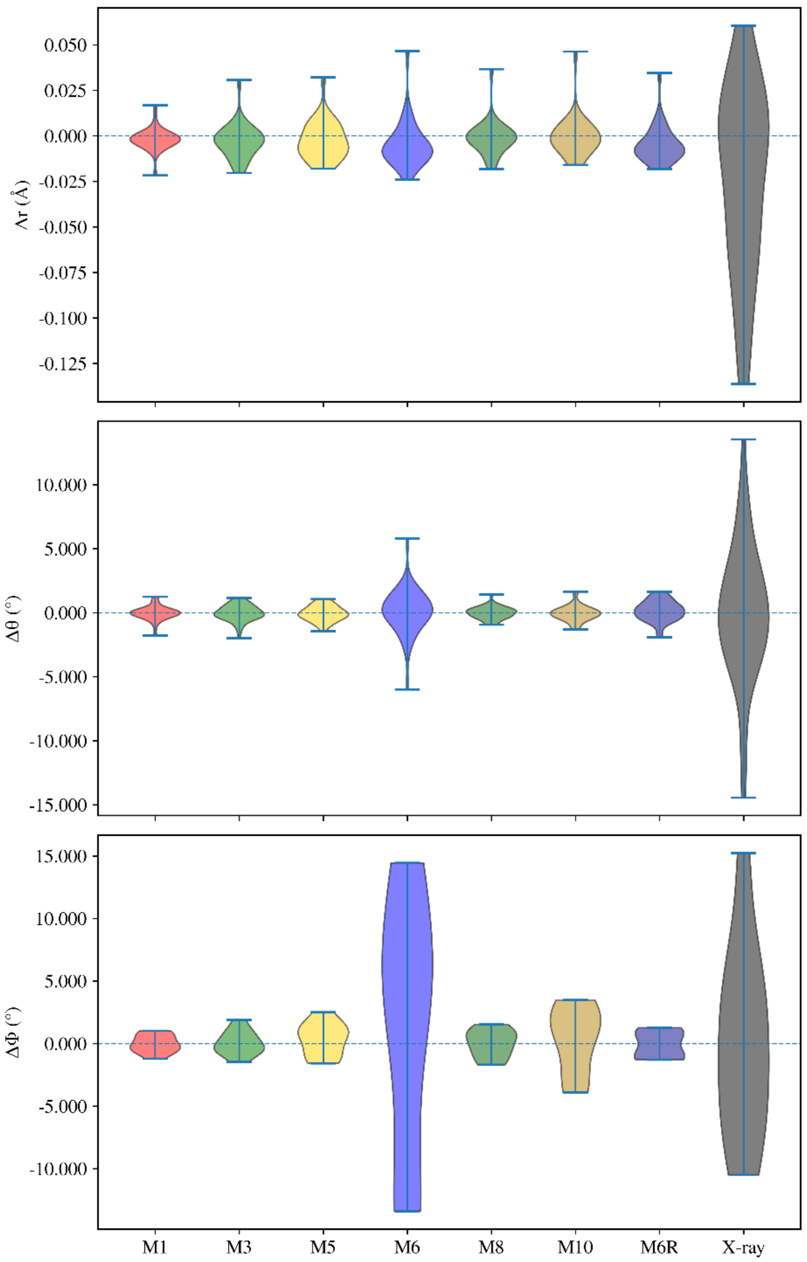

Supplementary Figure 187: Key coordinates of quantum refinement results of guanosine-5'-monophosphate. Deviation in the refined bond distances (Δr, n = 26), angles (Δθ, n = 40) and dihedrals (Δϕ, n = 10) of guanosine-5'-monophosphate (5GP) in YfiR from various quantum refinement schemes (**M1-M10**) and X-ray structure which are compared to those obtained from the most reliable **M7** scheme. The solid line represents the upper and lower values.

(xxxiii) 1NOX (NADH oxidase (NOX) from Thermus thermophilus)

Protein preparations:

Resolution: 2.50 Å

Ligand: FMN (Flavin mononucleotide); $C_{17}H_{21}N_4O_9P$

Residue flipped: No

Protonation states (pH = 5.5):

HID75, HIP95, HIP101, HIP194

Optimized region: FMN

High layer: FMN

Medium layer: ARG17, ARG18, SER19, ARG21, GLN73, TYR137, PRO156, MET157, LEU158, GLY159, ARG195, WAT302, WAT303, WAT311, WAT341, WAT353, WAT362, WAT372, WAT373

ω_α = 0.14576

(a)

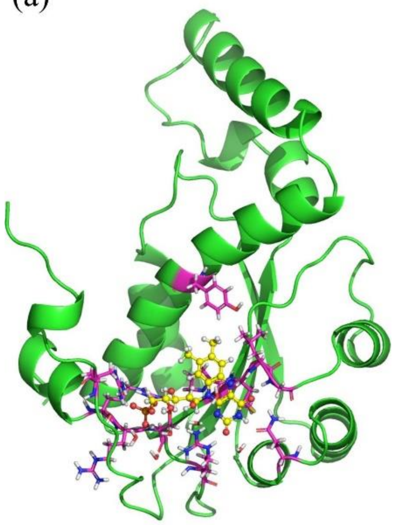

(b)

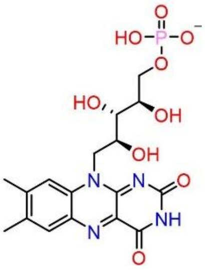

Supplementary Figure 188: NADH oxidase (NOX) from Thermus thermophilus. (**a**) Crystal structure of NOX complexed with flavin mononucleotide (FMN). ONIOM layers by different colors: yellow: high layer; red: medium layer; green: low layer. Ligand flavin mononucleotide is presented in stick and balls. (**b**) Structure of flavin mononucleotide.

Quantum refined structural results:

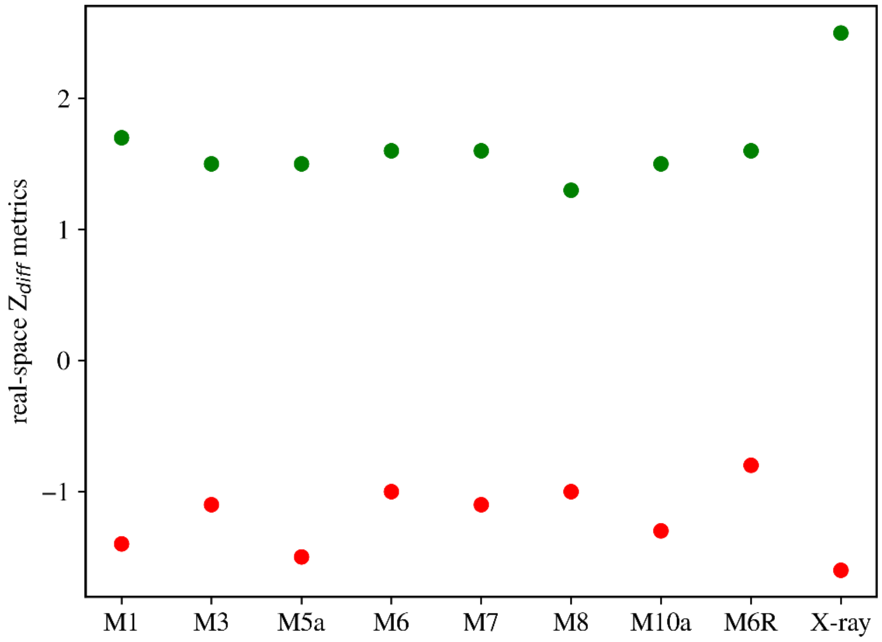

Supplementary Figure 189: Real-space Z-difference (RSZD) of flavin mononucleotide. RSZD+ (green) and RSZD- (red) scores of flavin mononucleotide (FMN) in NOX from various quantum refinement schemes (**M1-M10**). Those results for X-ray were taken from the experimental structure without our further refinement.

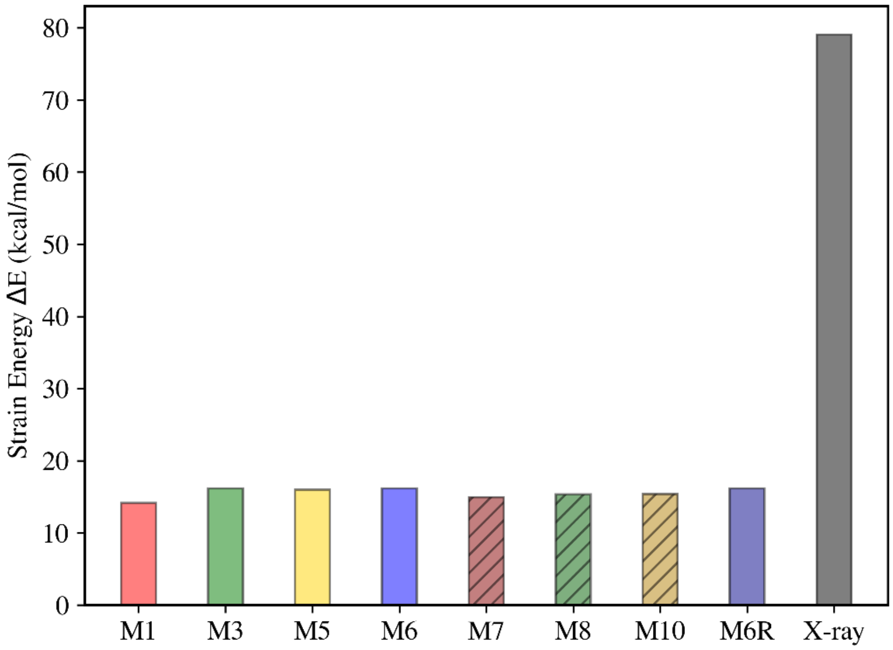

Supplementary Figure 190: Strain energy of flavin mononucleotide. Strain energy (ΔE, kcal·mol^{-1}) at ωB97X-D/6-31G(d) level for flavin mononucleotide (FMN) in NOX determined by various quantum refinement schemes (**M1-M10**). Those results for X-ray were taken from the experimental structure without our further refinement.

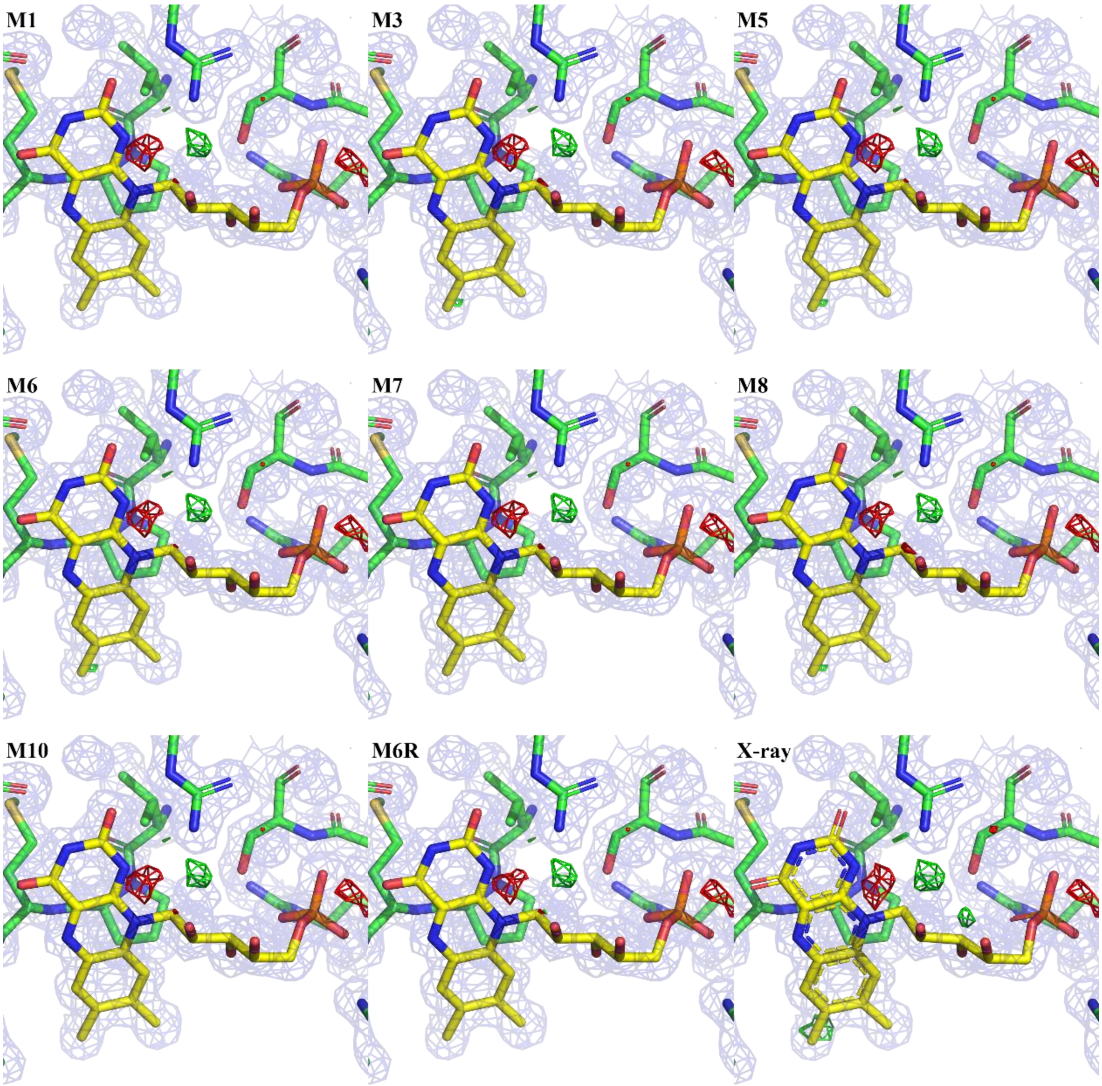

Supplementary Figure 191: Electron density maps of flavin mononucleotide. Structures for flavin mononucleotide (FMN) in NOX from various quantum refinement schemes (**M1-M10**), including the electron density maps (2mFo-DFc maps, contoured at 1.0 σ (blue), mFo-DFc maps, contoured at +3.0 σ (green), and mFo-DFc maps, contoured at -3.0 σ (red)). Those results for X-ray were taken from the experimental structure without our further refinement.

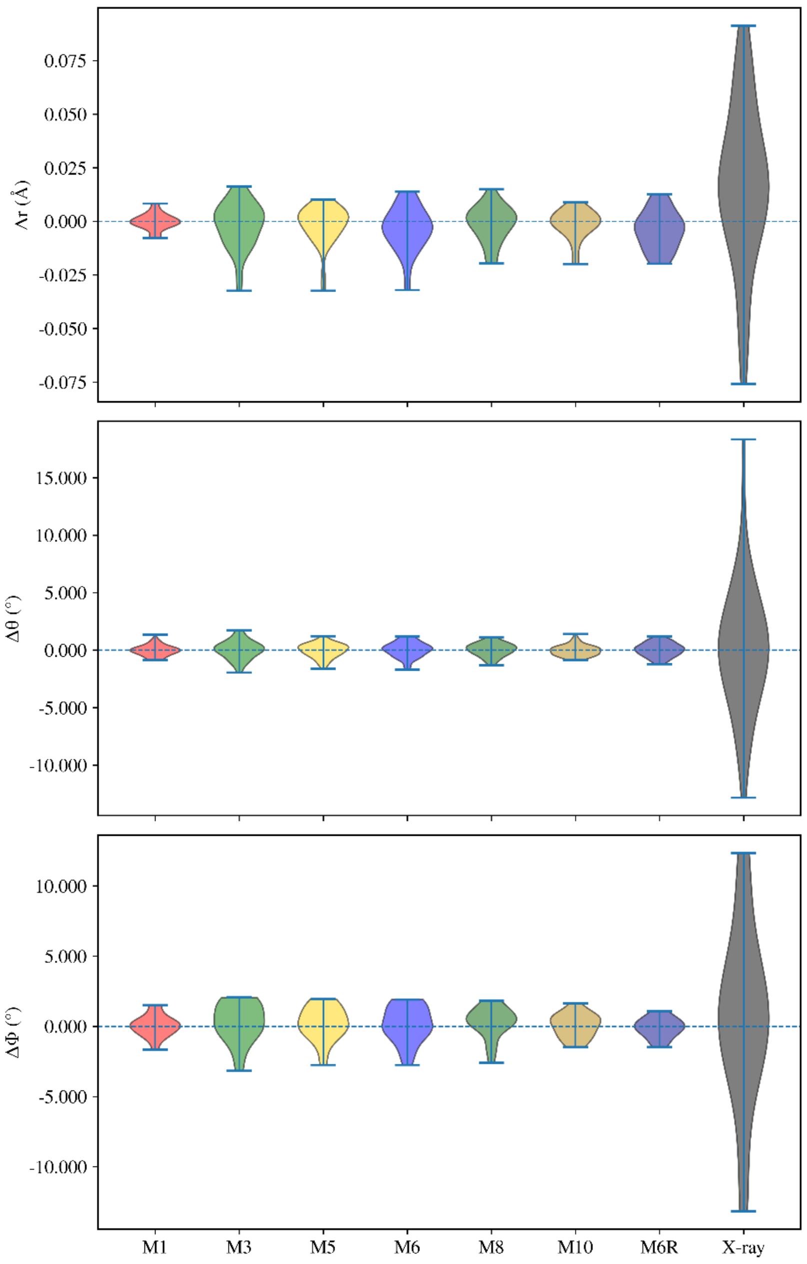

Supplementary Figure 192: Key coordinates of quantum refinement results of flavin mononucleotide. Deviation in the refined bond distances (Δr, n = 33), angles (Δθ, n = 50) and dihedrals (Δϕ, n = 18) of flavin mononucleotide (FMN) in NOX from various quantum refinement schemes (**M1-M10**) and X-ray structure which are compared to those obtained from the most reliable **M7** scheme. The solid line represents the upper and lower values.

(xxxiv) 1NJ1 (Prolyl-tRNA Synthetase from Methanothermobacter thermautotrophicus bound to cysteine sulfamoyl adenylate)

Protein preparations:

Resolution: 2.55 Å

Ligand: 5CA; $C_{13}H_{19}N_7O_7S_2$

Residue flipped: GLN241, HIS262, ASN398, ASN466

Protonation states (pH = 7.5):

HID108, HID188, ASH194, HID240, CYM241

Optimized region: CSN

High layer: CSN

Medium layer: THR117, GLU119, ARG148, GLU150, ARG159, VAL160, ILE163, PHE166, GLU168, PHE212, GLN232, ILE233, GLY234, THR235, HIE237, CYS265, TYR266, GLY267, LEU268, SER269, ARG271, WAT487

ω_α = 1.4537

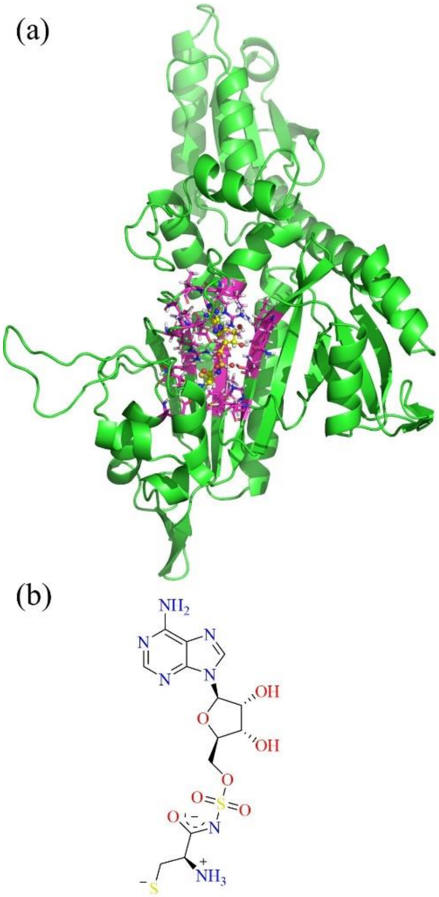

Supplementary Figure 193: Prolyl-tRNA Synthetase from Methanothermobacter thermautotrophicus bound to cysteine sulfamoyl adenylate. (**a**) Crystal structure of Prolyl-tRNA synthetase complexed with inhibitor 5CA. ONIOM layers by different colors: yellow: high layer; red: medium layer; green: low layer. Inhibitor 5CA is presented in stick and balls. (**b**) Structure of inhibitor 5CA.

Quantum refined structural results:

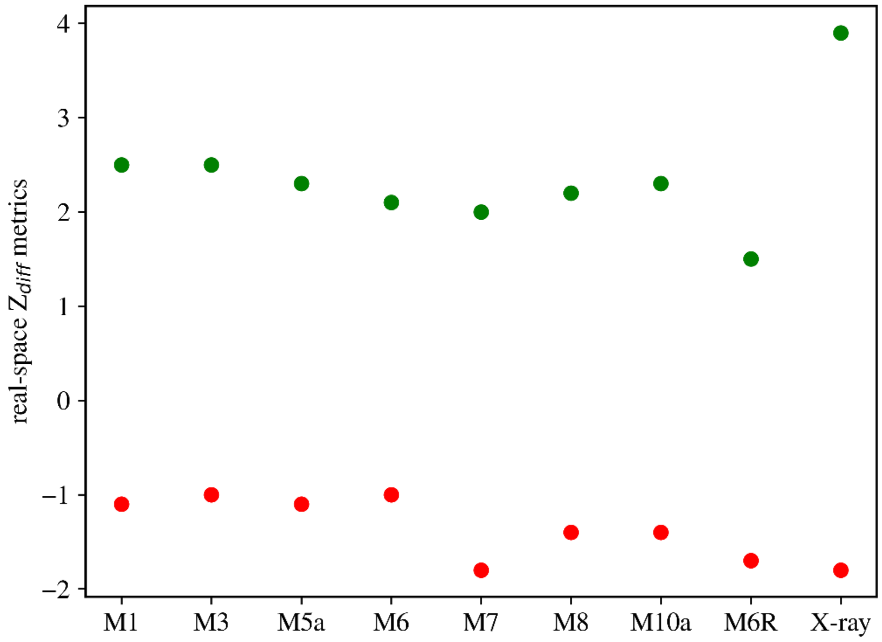

Supplementary Figure 194: Real-space Z-difference (RSZD) of inhibitor 5CA. RSZD+ (green) and RSZD- (red) scores of inhibitor 5CA in Prolyl-tRNA synthetase from various quantum refinement schemes (**M1-M10**). Those results for X-ray were taken from the experimental structure without our further refinement.

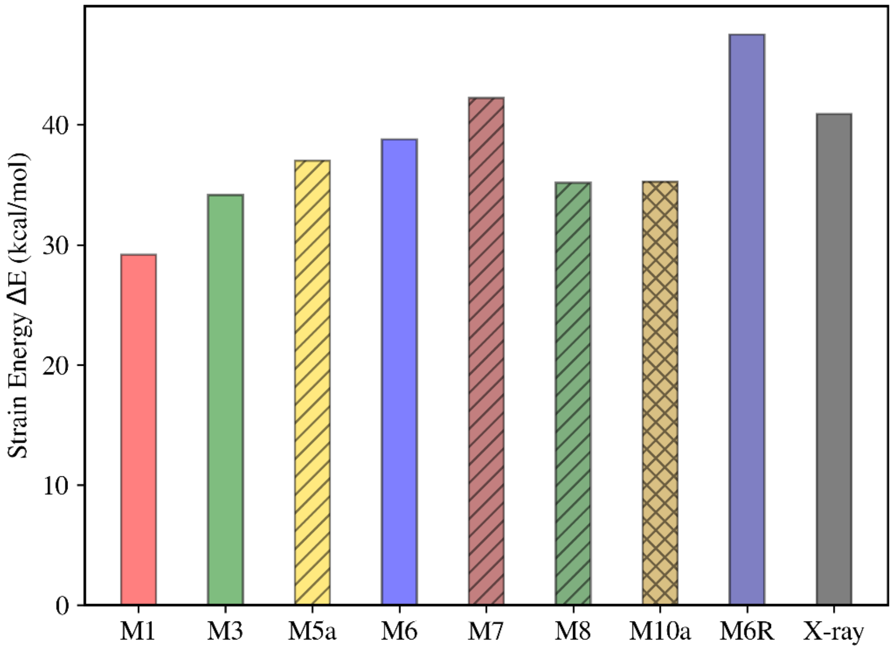

Supplementary Figure 195: Strain energy of inhibitor 5CA. Strain energy (ΔE, kcal·mol^{-1}) at ωB97X-D/6-31G(d) level for inhibitor 5CA in Prolyl-tRNA synthetase determined by various quantum refinement schemes (**M1-M10**). Those results for X-ray were taken from the experimental structure without our further refinement.

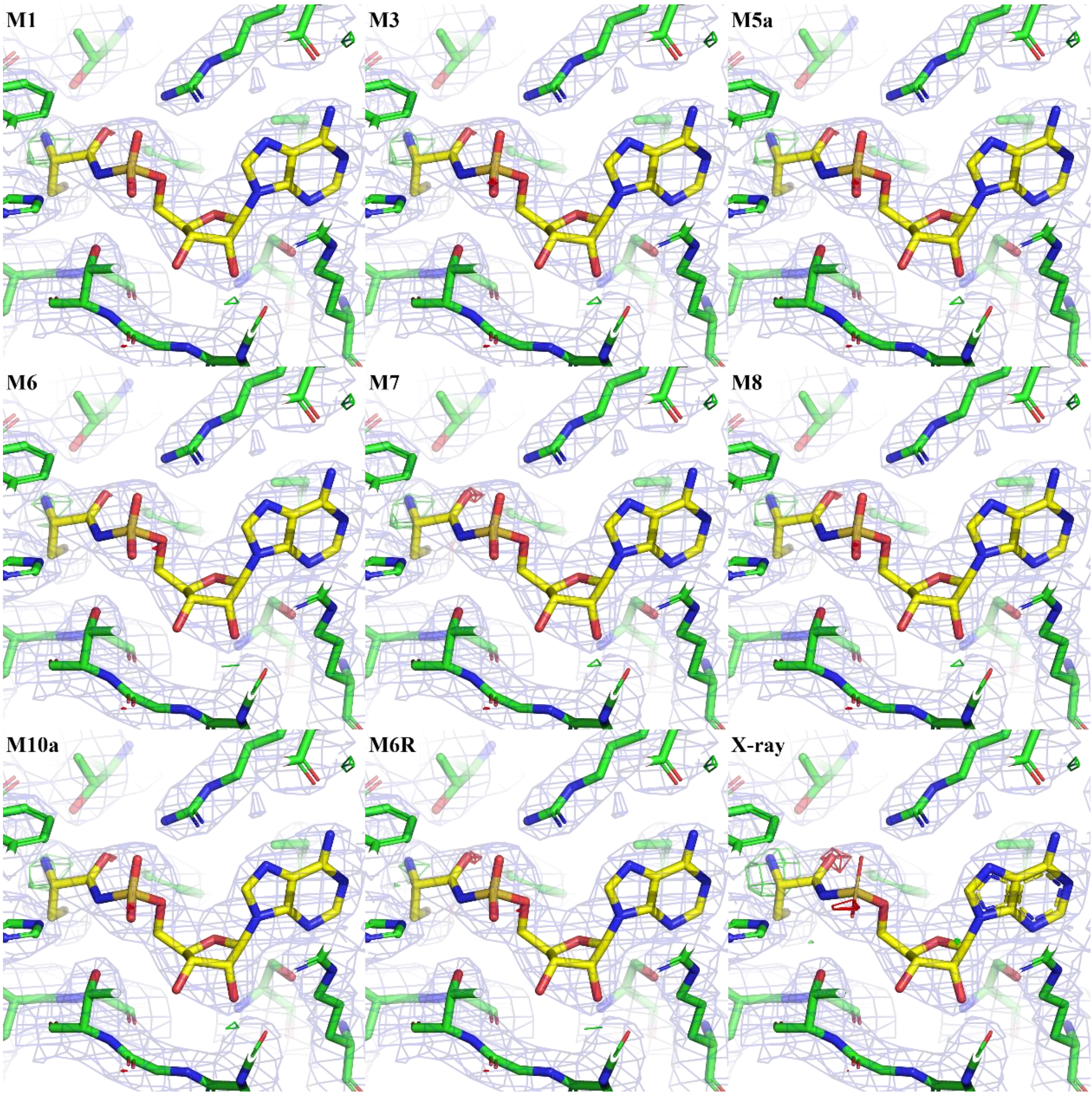

Supplementary Figure 196: Electron density maps of inhibitor 5CA. Structures for inhibitor 5CA in Prolyl-tRNA synthetase from various quantum refinement schemes (**M1-M10**), including the electron density maps (2mFo-DFc maps, contoured at 1.0 σ (blue), mFo-DFc maps, contoured at +3.0 σ (green), and mFo-DFc maps, contoured at -3.0 σ (red)). Those results for X-ray were taken from the experimental structure without our further refinement.

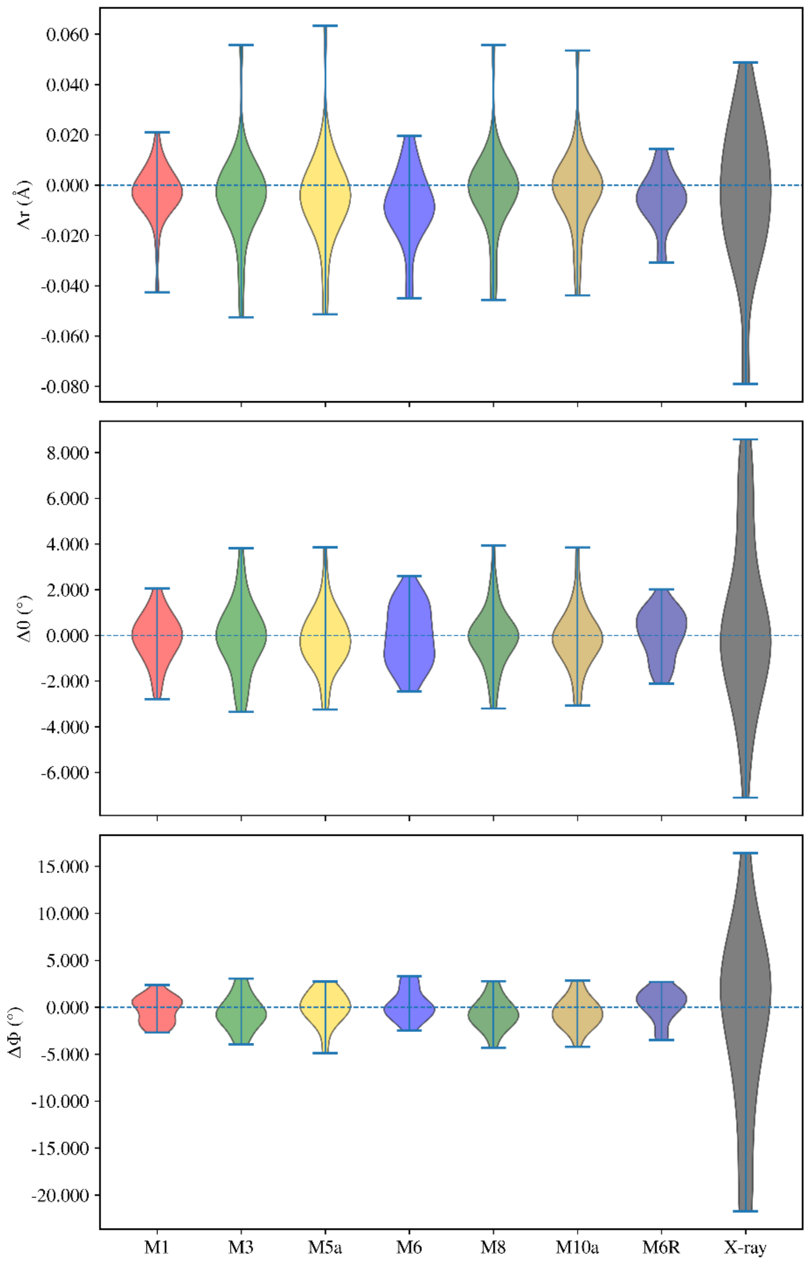

Supplementary Figure 197: Key coordinates of quantum refinement results of inhibitor 5CA. Deviation in the refined bond distances (Δr, n = 31), angles (Δθ, n = 46) and dihedrals (Δϕ, n = 21) of inhibitor 5CA in Prolyl-tRNA synthetase from various quantum refinement schemes (**M1-M10**) and X-ray structure which are compared to those obtained from the most reliable **M7** scheme. The solid line represents the upper and lower values.

(xxxv) 2A3I (Hormone Binding and Coactivator Assembly by Mineralocorticoid Receptor)

Protein preparations:

Resolution: 1.95 Å

Ligand: C0R (Corticosterone); $C_{21}H_{30}O_4$

Residue flipped: GLN825, HIS841, GLN916, GLN919, ASN975

Protonation states (pH = 7.9):

HID821, HID841, HID853, HID932, HID950, HID982

Optimized region: C0R

High layer: C0R

Medium layer: WAT2, LEU766, LEU769, ASN770, LEU772, ALA773, GLN776, TRP806, MET807, SER810, SER811, LEU814, ARG817, PHE829, MET845, MET852, LEU938, PHE941, CYS942, THR945, VAL954, PHE956

ω_α = 1.7952

(a)

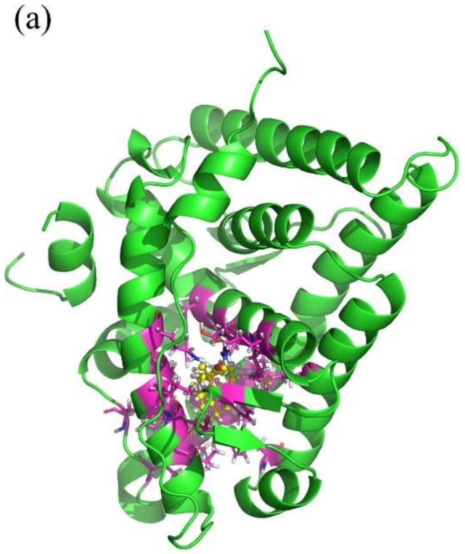

(b)

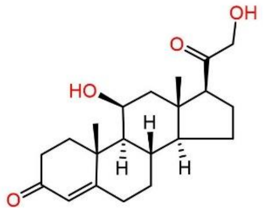

Supplementary Figure 198: Hormone Binding and Coactivator Assembly by Mineralocorticoid Receptor. (**a**) Crystal structure of mineralocorticoid receptor complexed with corticosterone (C0R). ONIOM layers by different colors: yellow: high layer; red: medium layer; green: low layer. Ligand corticosterone is presented in stick and balls. (**b**) Structure of corticosterone.

Quantum refined structural results:

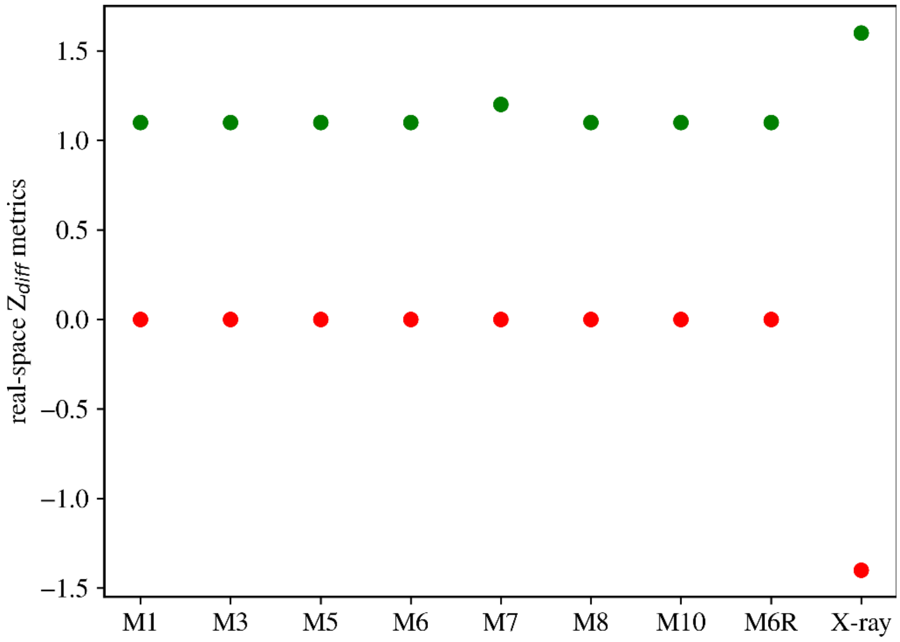

Supplementary Figure 199: Real-space Z-difference (RSZD) of corticosterone. RSZD+ (green) and RSZD- (red) scores of corticosterone (C0R) in mineralocorticoid receptor from various quantum refinement schemes (**M1-M10**). Those results for X-ray were taken from the experimental structure without our further refinement.

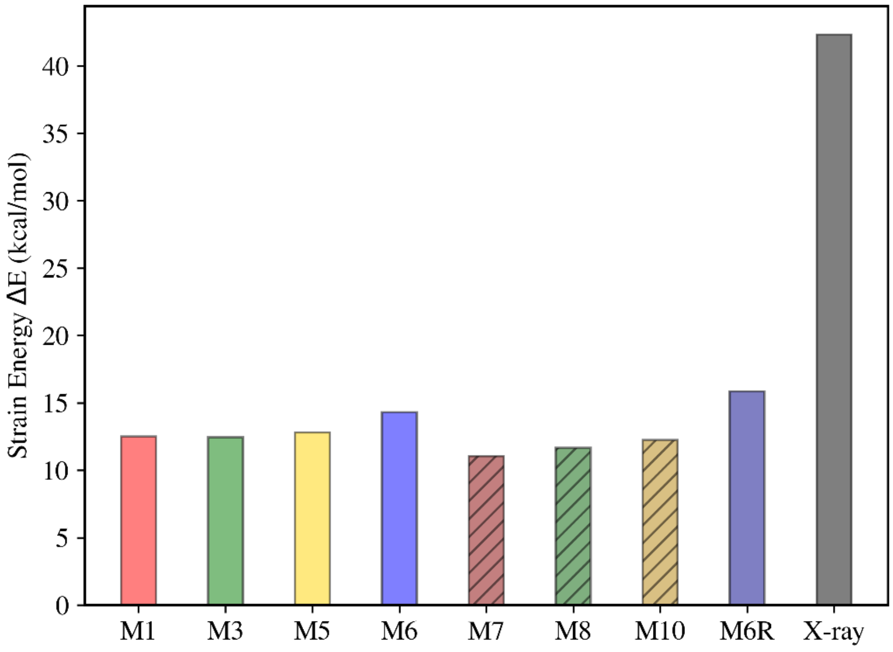

Supplementary Figure 200: Strain energy of corticosterone. Strain energy (ΔE, kcal·mol^{-1}) at ωB97X-D/6-31G(d) level for corticosterone (C0R) in mineralocorticoid receptor determined by various quantum refinement schemes (**M1-M10**). Those results for X-ray were taken from the experimental structure without our further refinement.

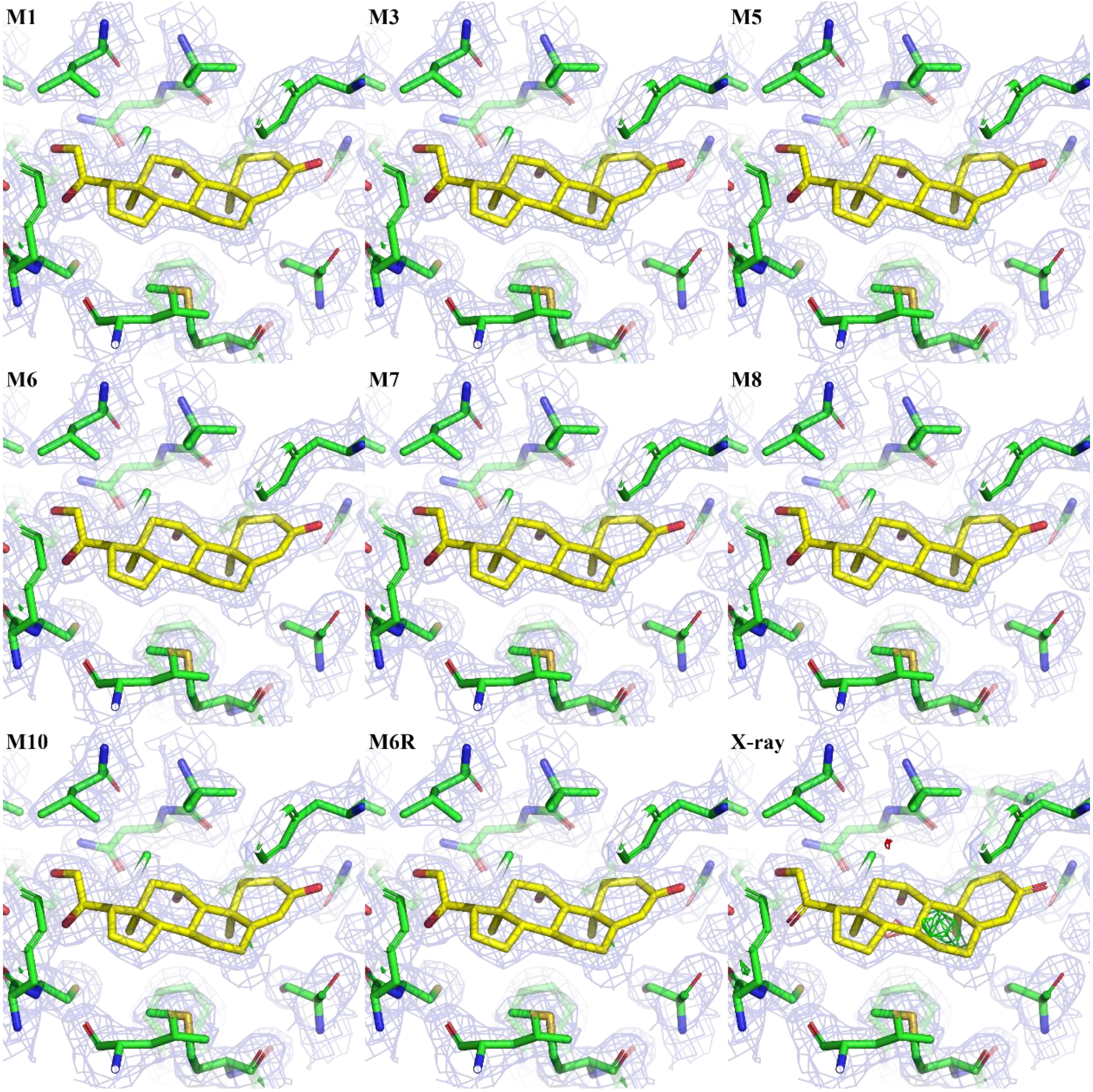

Supplementary Figure 201: Electron density maps of corticosterone. Structures for corticosterone (C0R) in mineralocorticoid receptor from various quantum refinement schemes (**M1-M10**), including the electron density maps (2mFo-DFc maps, contoured at 1.0 σ (blue), mFo-DFc maps, contoured at +3.0 σ (green), and mFo-DFc maps, contoured at -3.0 σ (red)). Those results for X-ray were taken from the experimental structure without our further refinement.

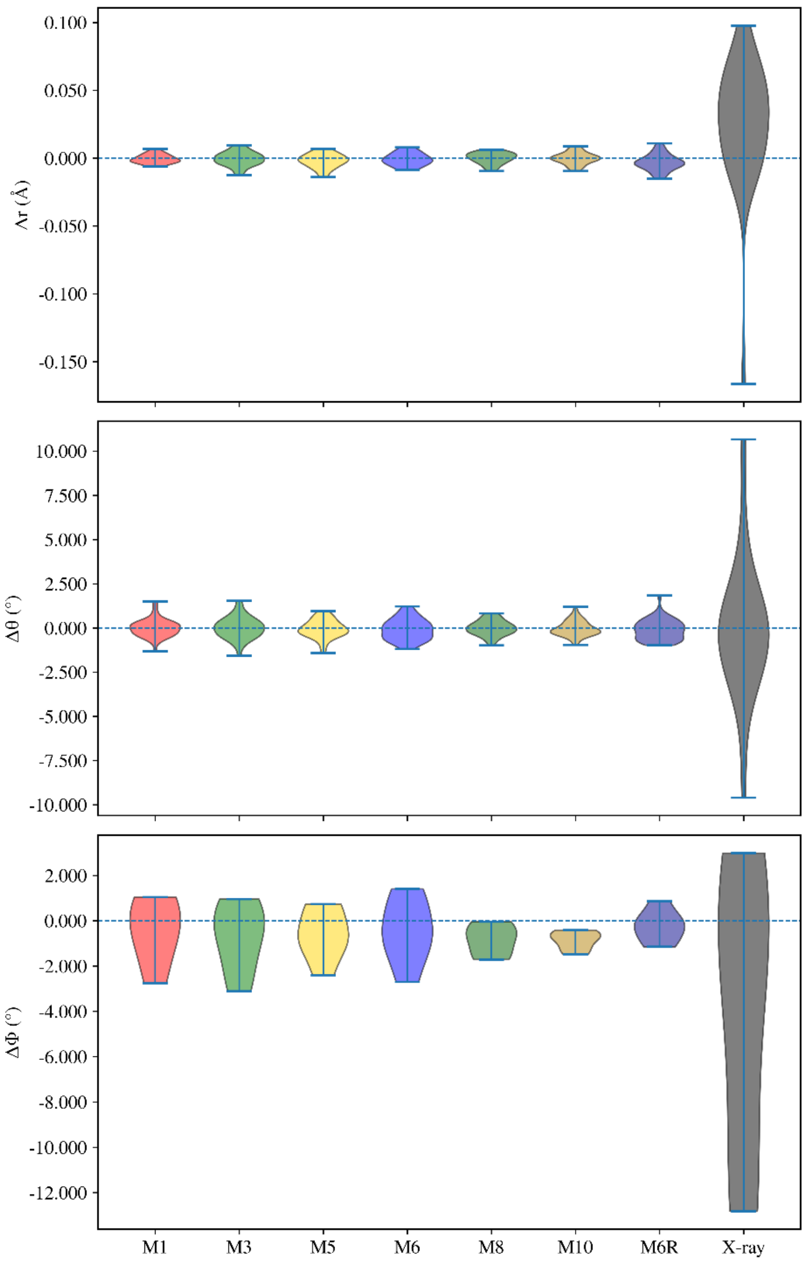

Supplementary Figure 202: Key coordinates of quantum refinement results of corticosterone. Deviation in the refined bond distances (Δr, n = 28), angles (Δθ, n = 45) and dihedrals (Δϕ, n = 6) of corticosterone (C0R) in mineralocorticoid receptor from various quantum refinement schemes (**M1-M10**) and X-ray structure which are compared to those obtained from the most reliable **M7** scheme. The solid line represents the upper and lower values.

(xxxvi) 5CT1 (NK1 fragment of HGF/SF complexed with CHES)

Protein preparations:

Resolution: 2.00 Å

Ligand: NHE (N-cyclohexyltaurine); $C_8H_{17}NO_3S$

Residue flipped:

Chain A: ASN77, GLN205

Chain B: ASN72, HIS158

Protonation states (pH = 7.5):

Chain A: HID40, HID114, HID158, HID160,

Chain B: HID40, HID114, HID158, HID160

Optimized region: NHE

High layer: NHE

Medium layer: Chain B: PHE162, ARG181, GLU183, GLY185, TRP188, PHE190, ARG197, TYR198

ω_α = 0.76343

(a)

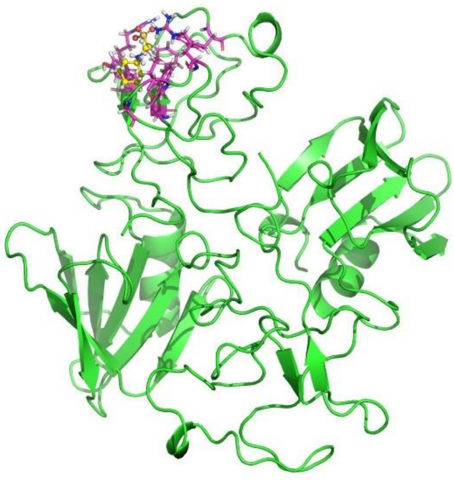

(b)

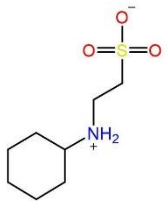

Supplementary Figure 203: NK1 fragment of HGF/SF complexed with CHES. (a) Crystal structure of NK1 fragment of HGF/SF complexed with N-cyclohexyltaurine (NHE). ONIOM layers by different colors: yellow: high layer; red: medium layer; green: low layer. Ligand N-cyclohexyltaurine is presented in stick and balls. (**b**) Structure of N-cyclohexyltaurine.

Quantum refined structural results:

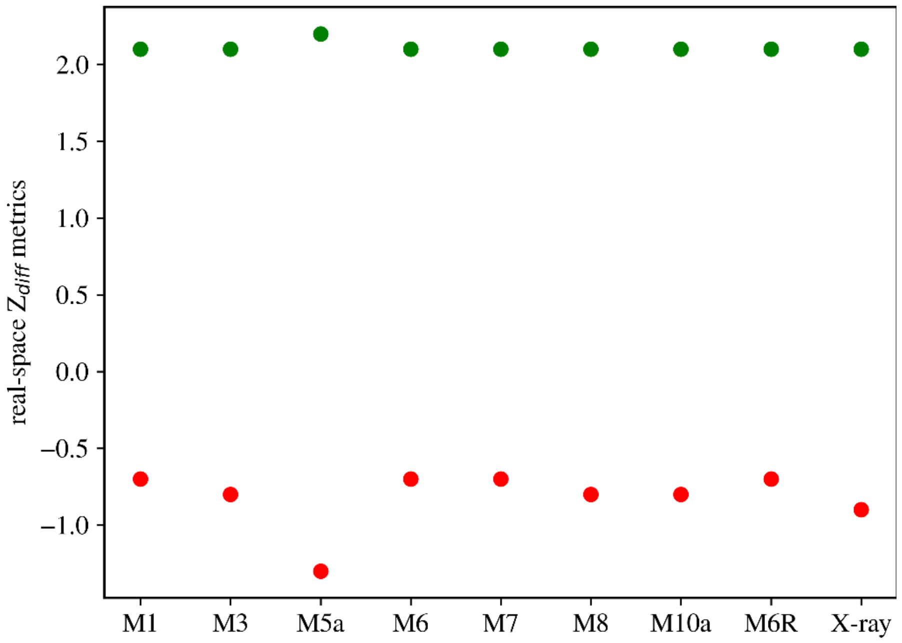

Supplementary Figure 204: Real-space Z-difference (RSZD) of N-cyclohexyltaurine. RSZD+ (green) and RSZD- (red) scores of N-cyclohexyltaurine (NHE) in HGF/SF from various quantum refinement schemes (**M1-M10**). Those results for X-ray were taken from the experimental structure without our further refinement.

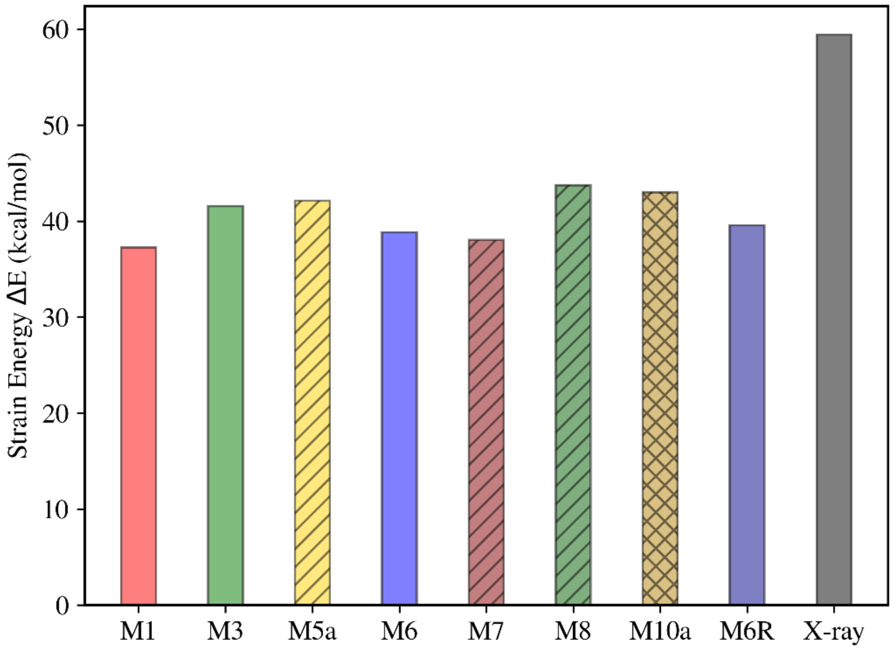

Supplementary Figure 205: Strain energy of N-cyclohexyltaurine. Strain energy (ΔE, kcal·mol^{-1}) at ωB97X-D/6-31G(d) level for N-cyclohexyltaurine (NHE) in HGF/SF determined by various quantum refinement schemes (**M1-M10**). Those results for X-ray were taken from the experimental structure without our further refinement.

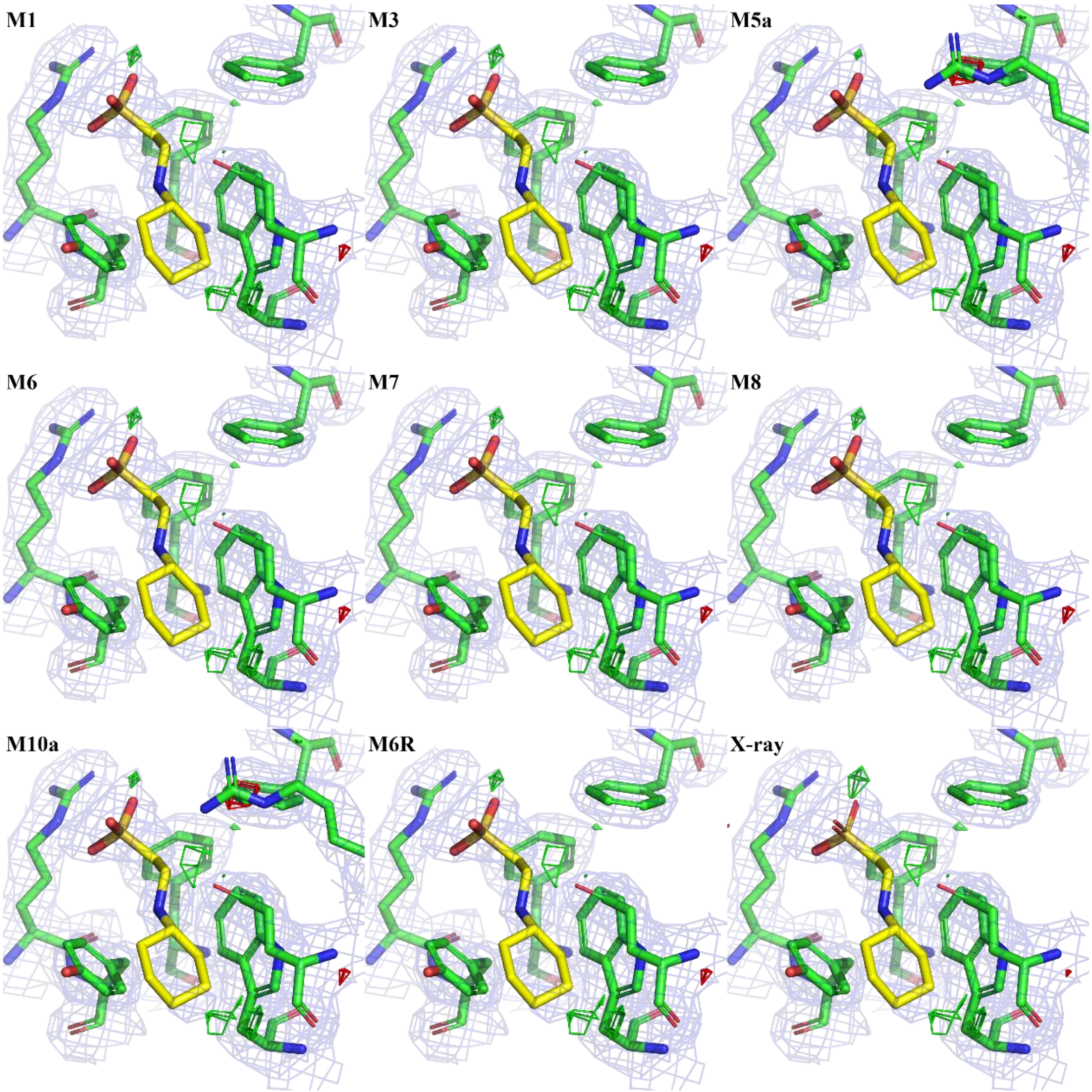

Supplementary Figure 206: Electron density maps of N-cyclohexyltaurine. Structures for N-cyclohexyltaurine (NHE) in HGF/SF from various quantum refinement schemes (**M1**-**M10**), including the electron density maps (2mFo-DFc maps, contoured at 1.0 σ (blue), mFo-DFc maps, contoured at +3.0 σ (green), and mFo-DFc maps, contoured at -3.0 σ (red)). Those results for X-ray were taken from the experimental structure without our further refinement.

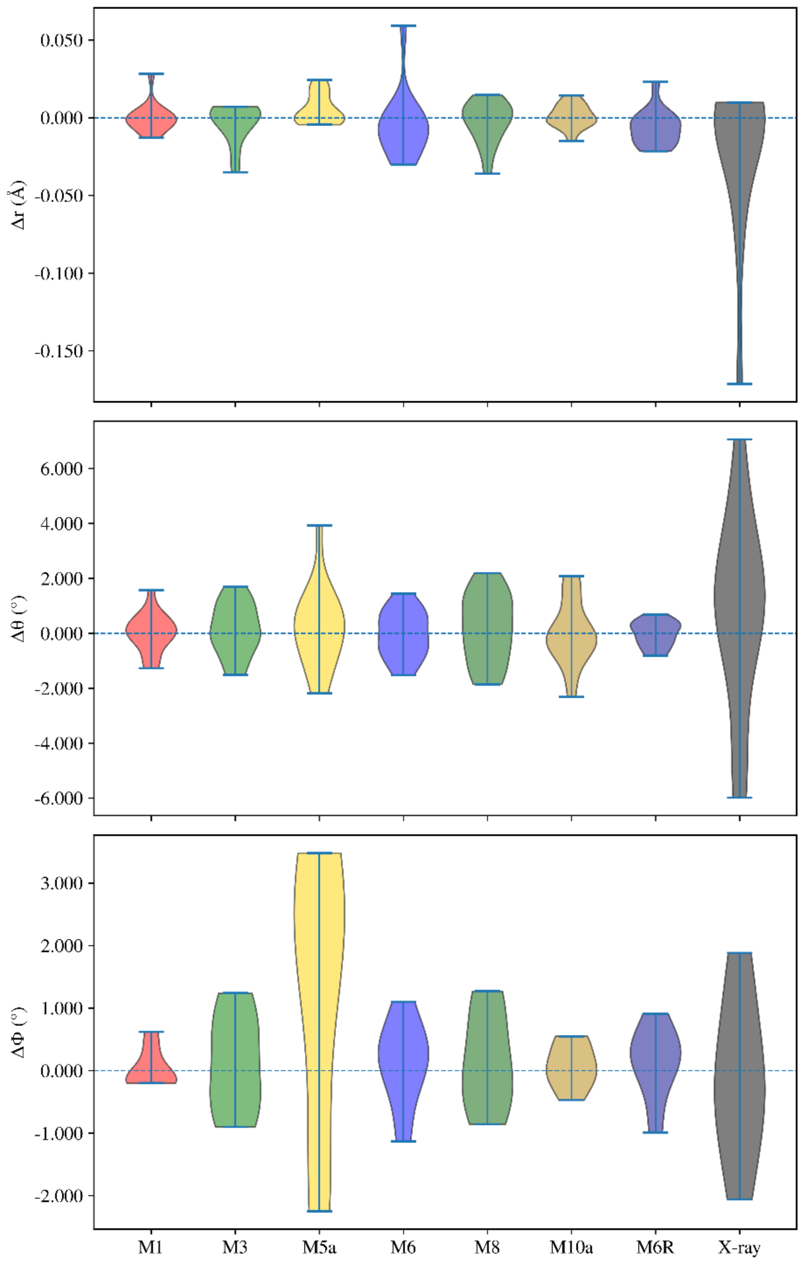

Supplementary Figure 207: Key coordinates of quantum refinement results of N-cyclohexyltaurine. Deviation in the refined bond distances (Δr, n = 13), angles (Δθ, n = 17) and dihedrals (Δϕ, n = 7) of N-cyclohexyltaurine (NHE) in HGF/SF from various quantum refinement schemes (**M1-M10**) and X-ray structure which are compared to those obtained from the most reliable **M7** scheme. The solid line represents the upper and lower values.

(xxxvii) 6AFA (DJ-1 with isatin)

Protein preparations:

Resolution: 2.50 Å

Ligand: ISN (Isatin); $C_8H_5NO_2$

Residue flipped: No

Protonation states (pH = 8.5):

CYM106, HID115, HID126, HID138

Optimized region: ISN + CYS(CYM106)

High layer: ISN + CYS(CYM106)

Medium layer: GLU15, GLU18, GLY74, GLY75, ASN76, ILE105, ALA107, GLY108, PRO109, THR125, HID126, LEU128, SER155, ARG156, GLY157, PRO158, WAT301, WAT371

ω_α = 0.27767

(a)

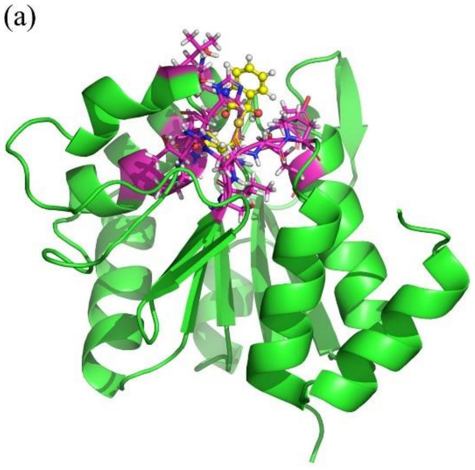

(b)

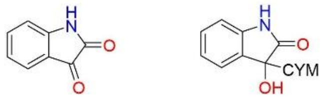

Supplementary Figure 208: DJ-1 with isatin. (**a**) Crystal structure of DJ-1 complexed with Isatin (ISN). ONIOM layers by different colors: yellow: high layer; red: medium layer; green: low layer. Ligand Isatin is presented in stick and balls. (**b**) Structure of Isatin (bonded and non-bonded).

Quantum refined structural results:

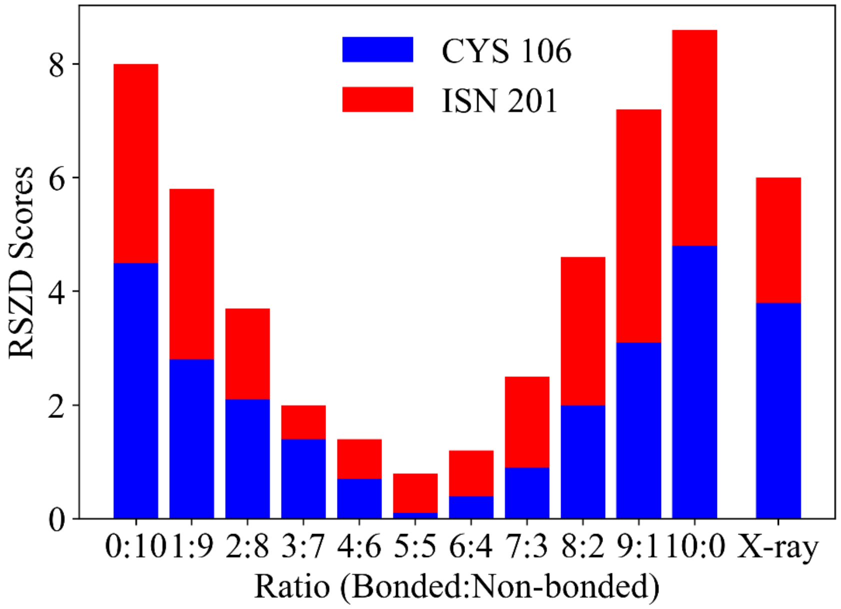

Supplementary Figure 209: Real-space Z-difference (RSZD) of Isatin (ISN) and CYS106. RSZD scores of Isatin (ISN) and CYS106 in DJ-1 with different occupations (bonded: Non-bonded) from **M7** quantum refinement schemes. Those results for X-ray were taken from the experimental structure without our further refinement.

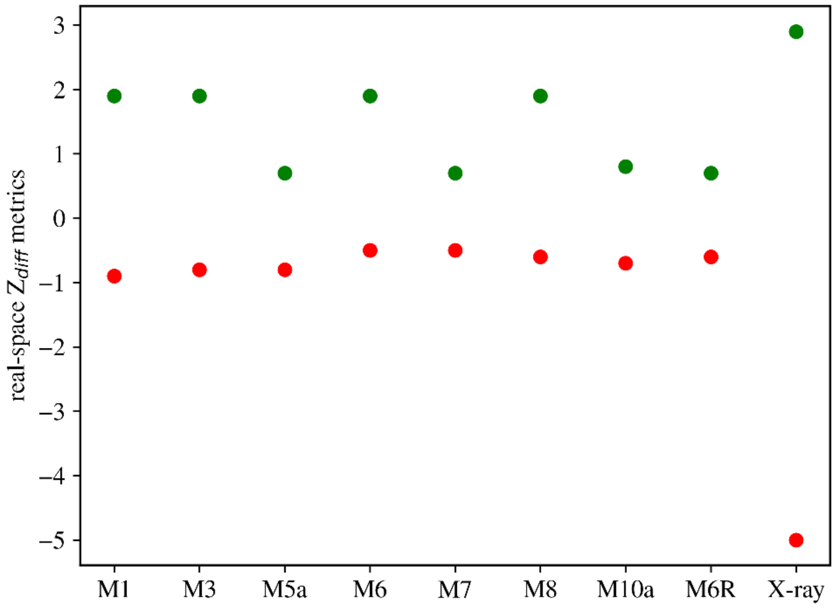

Supplementary Figure 210: Real-space Z-difference (RSZD) of Isatin (ISN) and CYS106. RSZD+ (green) and RSZD- (red) scores of sum of Isatin (ISN) and CYS(CYM)106 (bonded: Non-bonded=5:5) in DJ-1 from various quantum refinement schemes (**M1**-**M10**). Those results for X-ray were taken from the experimental structure without our further refinement.

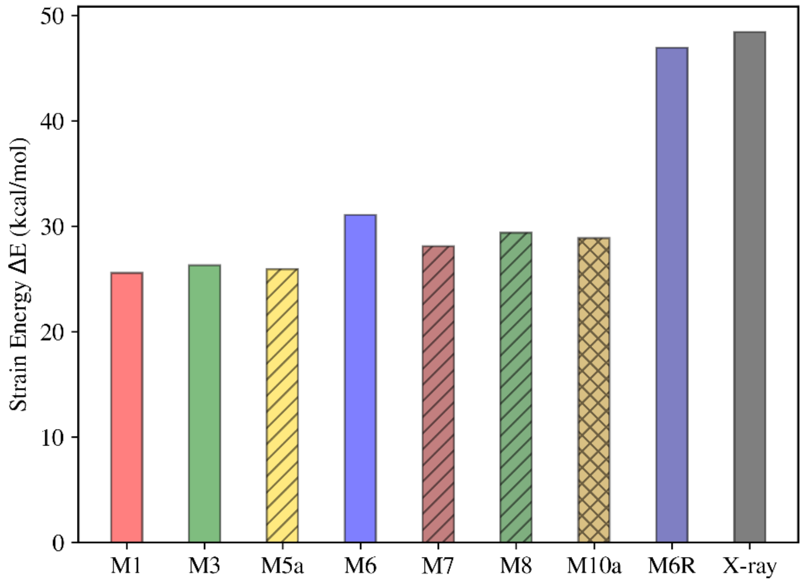

Supplementary Figure 211: Strain energy of Isatin (ISN) and CYM106 (bonded). Strain energy (ΔE, kcal·mol^{-1}) at ωB97X-D/6-31G(d) level for Isatin (ISN) and CYM106 in DJ-1 (bonded case) determined by various quantum refinement schemes (**M1-M10**). Those results for X-ray were taken from the experimental structure without our further refinement.

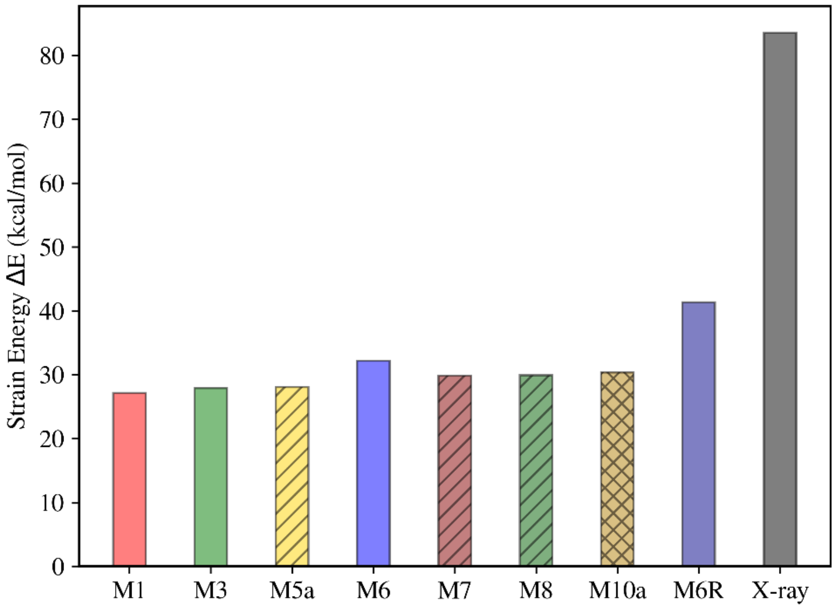

Supplementary Figure 212: Strain energy of Isatin (ISN) and CYM106 (non-bonded). Strain energy (ΔE, kcal·mol^{-1}) at ωB97X-D/6-31G(d) level for Isatin (ISN) and CYS106 in DJ-1 (non-bonded case) determined by various quantum refinement schemes (**M1-M10**). Those results for X-ray were taken from the experimental structure without our further refinement.

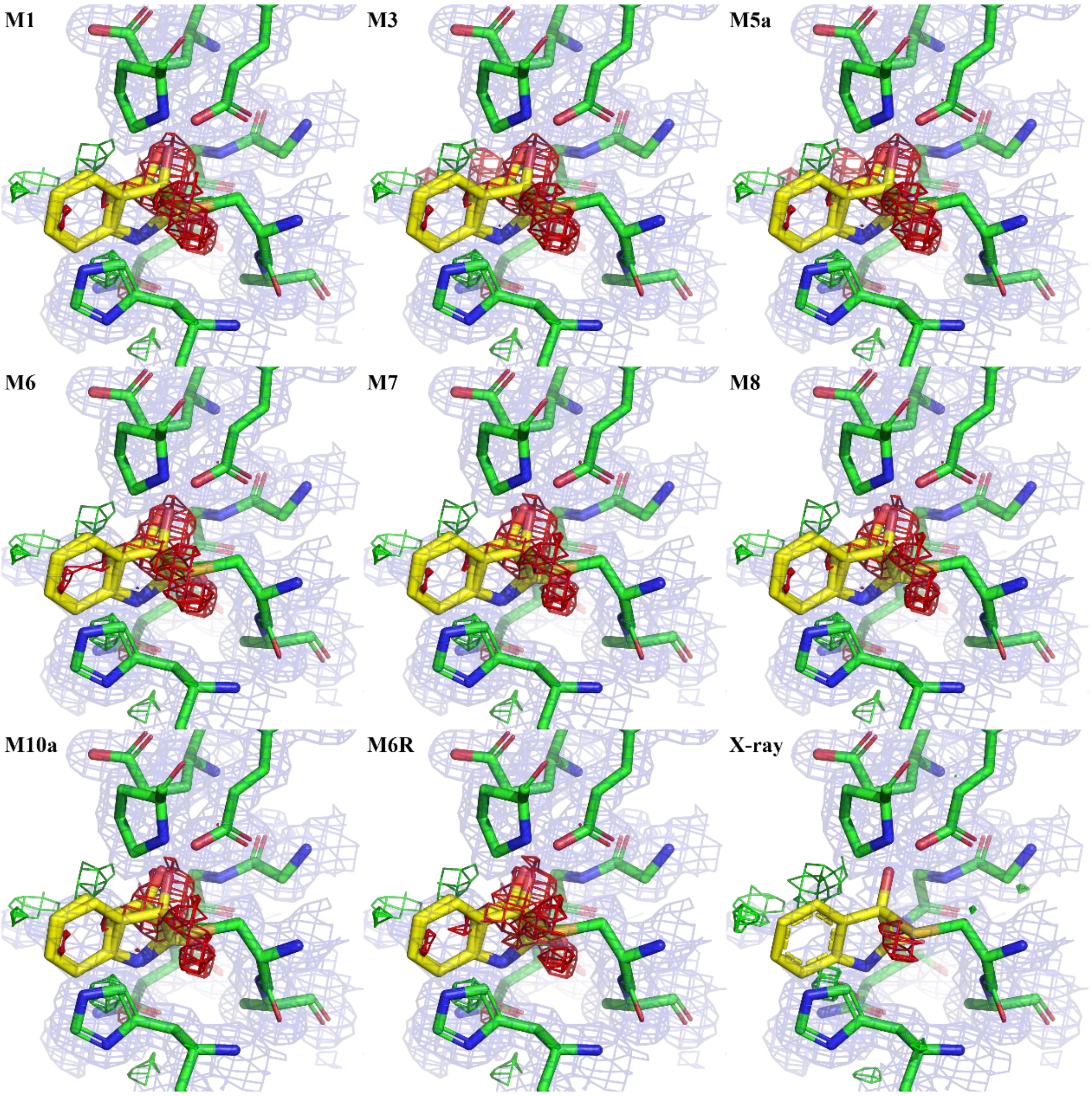

Supplementary Figure 213: Electron density maps of Isatin and CYS106. Structures for Isatin (ISN) and CYS(CYM)106 (bonded: non-bonded=5:5) in DJ-1 from various quantum refinement schemes (**M1**-**M10**), including the electron density maps (2mFo-DFc maps, contoured at 1.0 σ (blue), mFo-DFc maps, contoured at +3.0 σ (green), and mFo-DFc maps, contoured at -3.0 σ (red)). Those results for X-ray were taken from the experimental structure without our further refinement.

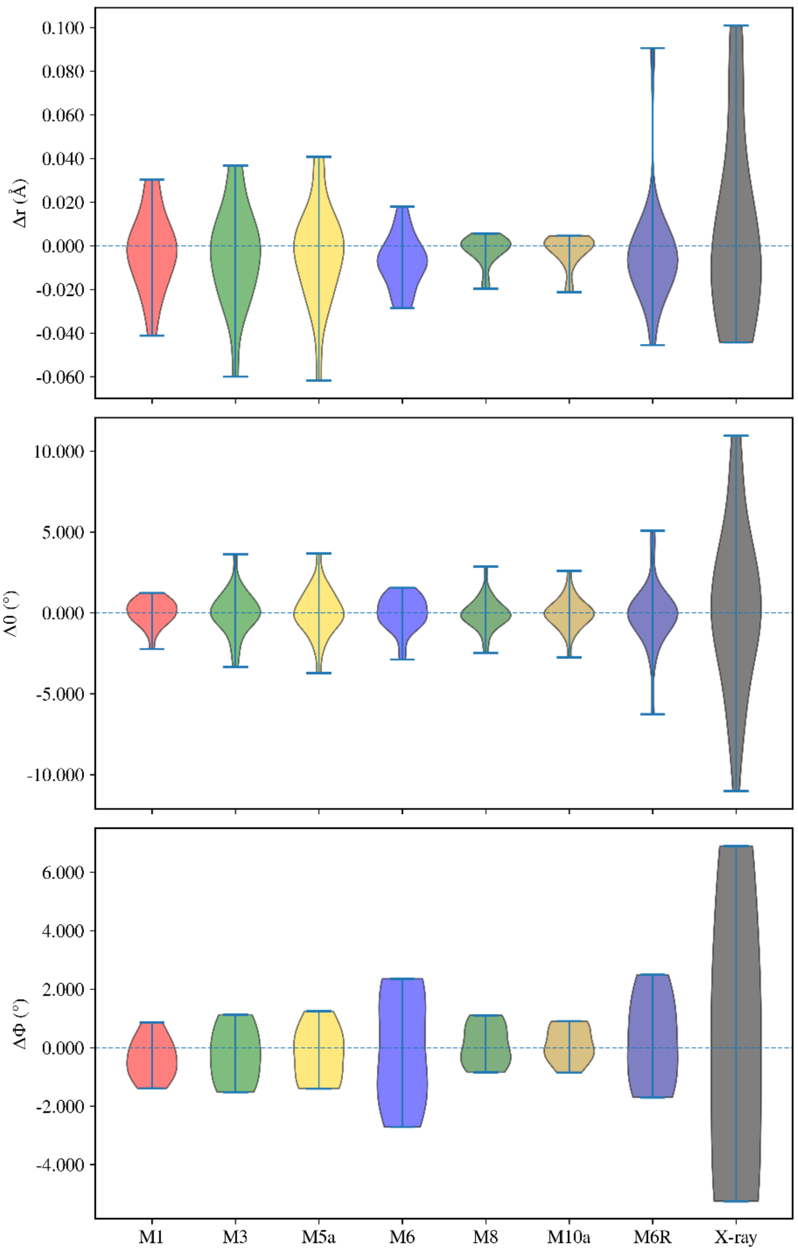

Supplementary Figure 214: Key coordinates of quantum refinement results of Isatin and CYM106 (bonded). Deviation in the refined bond distances (Δr, n = 18), angles (Δθ, n = 26) and dihedrals (Δϕ, n = 8) of Isatin (ISN) and CYM106 in DJ-1 (bonded case) from various quantum refinement schemes (**M1-M10**) and X-ray structure which are compared to those obtained from the most reliable **M7** scheme.

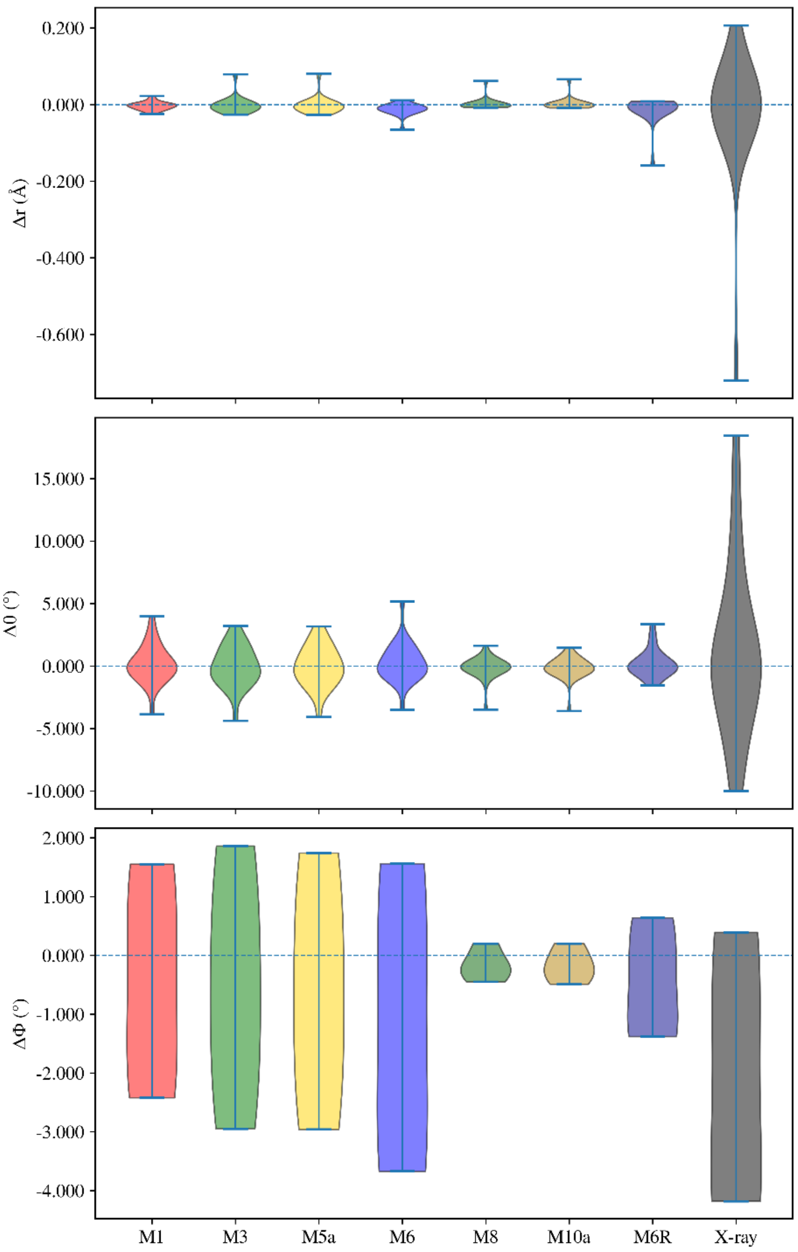

Supplementary Figure 215: Key coordinates of quantum refinement results of Isatin and CYM106 (non-bonded). Deviation in the refined bond distances (Δr, n = 18), angles (Δθ, n = 26) and dihedrals (Δϕ, n = 4) of Isatin (ISN) and CYM106 in DJ-1 (non-bonded case) from various quantum refinement schemes (**M1-M10**) and X-ray structure which are compared to those obtained from the most reliable **M7** scheme. The solid line represents the upper and lower values.

(xxxviii) 6PUB (Type B Chloramphenicol Acetyltransferase from Vibrio cholerae in the Complex with Crystal Violet)

Protein preparations:

Resolution: 2.50 Å

Ligand: CVI (Gentian violet cation); $C_{25}H_{30}N_3$

Residue flipped: HIS27, GLN91

Protonation states (pH = 6.5):

HID27, HID35, HIP37, HID47, HIP78, HID113, HID131, HIP158

Optimized region: CVI

High layer: CVI

Medium layer: MET1, PHE4, THR5, SER6, PRO7, PHE8, TYR33, TYR34, WAT427

ω_α = 1.3931

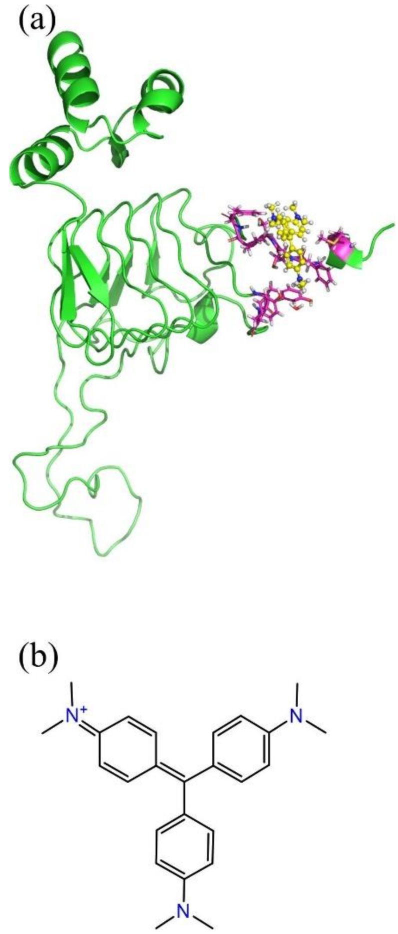

Supplementary Figure 216: Type B Chloramphenicol Acetyltransferase from Vibrio cholerae in the Complex with Crystal Violet. (**a**) Crystal structure of type B chloramphenicol acetyltransferase complexed with gentian violet cation (CVI). ONIOM layers by different colors: yellow: high layer; red: medium layer; green: low layer. Ligand gentian violet cation is presented in stick and balls. (**b**) Structure of gentian violet cation.

Quantum refined structural results:

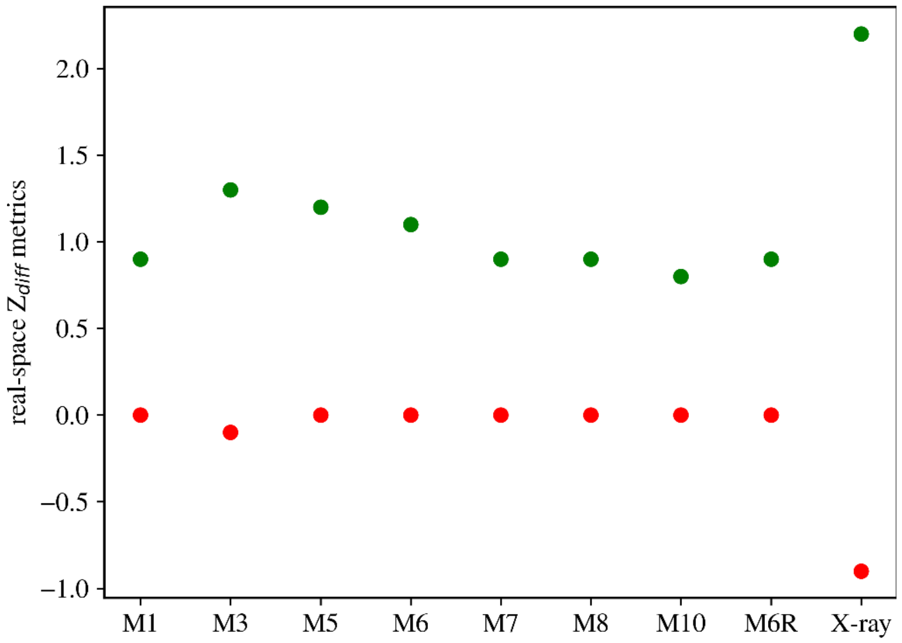

Supplementary Figure 217: Real-space Z-difference (RSZD) of gentian violet cation. RSZD+ (green) and RSZD- (red) scores of gentian violet cation (CVI) in type B chloramphenicol acetyltransferase from various quantum refinement schemes (**M1-M10**). Those results for X-ray were taken from the experimental structure without our further refinement.

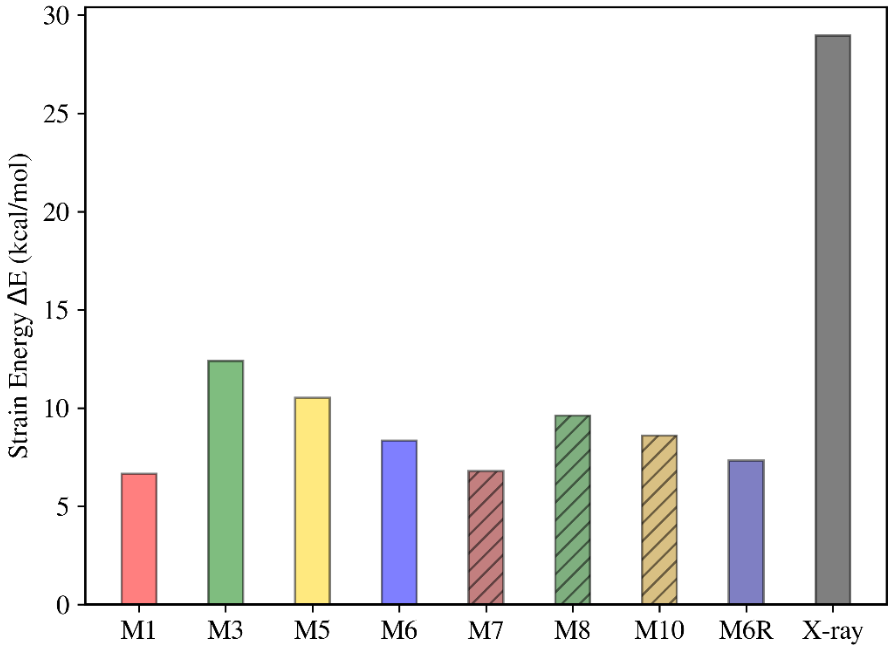

Supplementary Figure 218: Strain energy of gentian violet cation. Strain energy (ΔE, kcal·mol^{-1}) at ωB97X-D/6-31G(d) level for gentian violet cation (CVI) in type B chloramphenicol acetyltransferase determined by various quantum refinement schemes (**M1-M10**). Those results for X-ray were taken from the experimental structure without our further refinement.

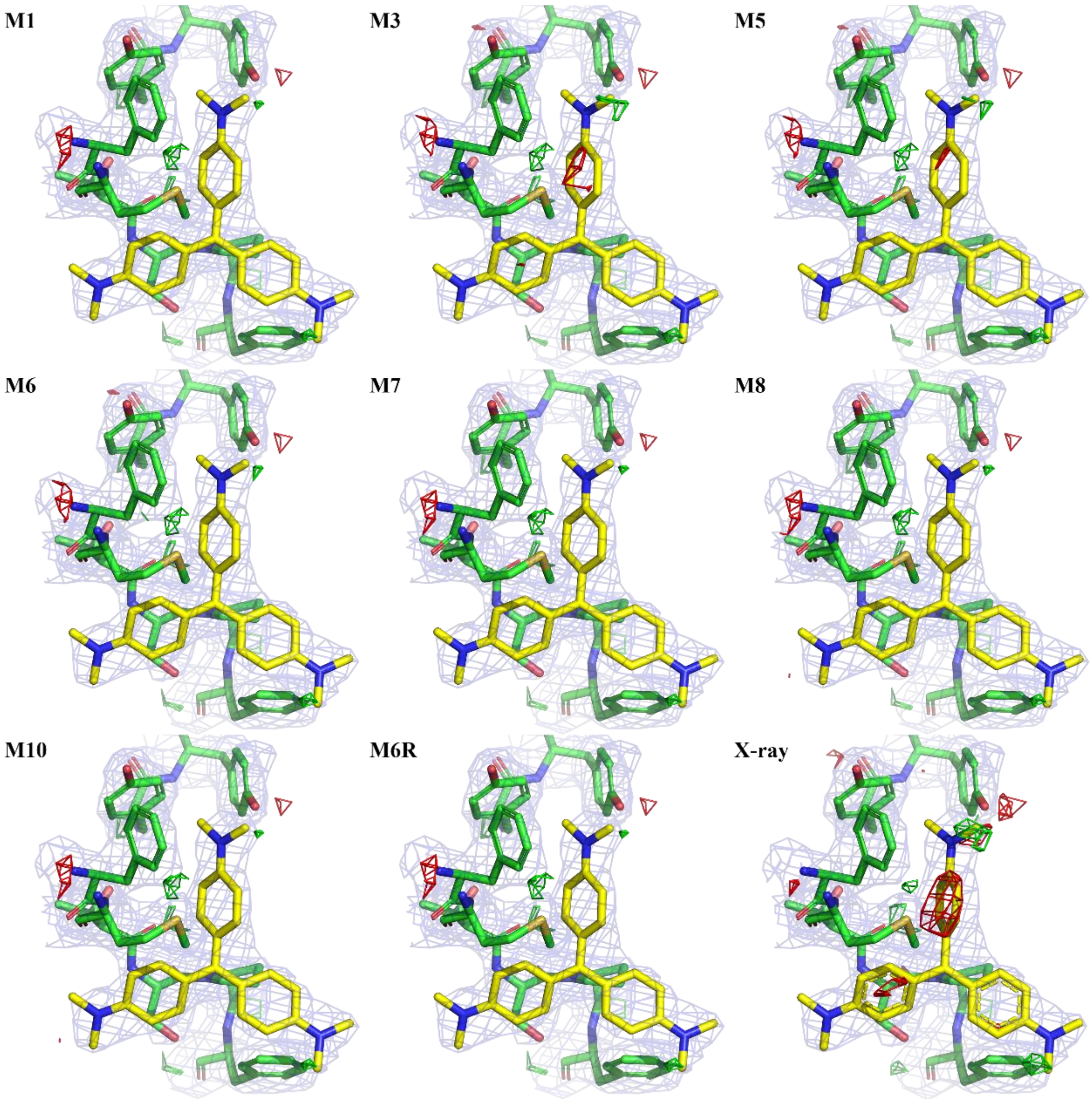

Supplementary Figure 219: Electron density maps of gentian violet cation. Structures for gentian violet cation (CVI) in type B chloramphenicol acetyltransferase from various quantum refinement schemes (**M1**-**M10**), including the electron density maps (2mFo-DFc maps, contoured at 1.0 σ (blue), mFo-DFc maps, contoured at +3.0 σ (green), and mFo-DFc maps, contoured at -3.0 σ (red)). Those results for X-ray were taken from the experimental structure without our further refinement.

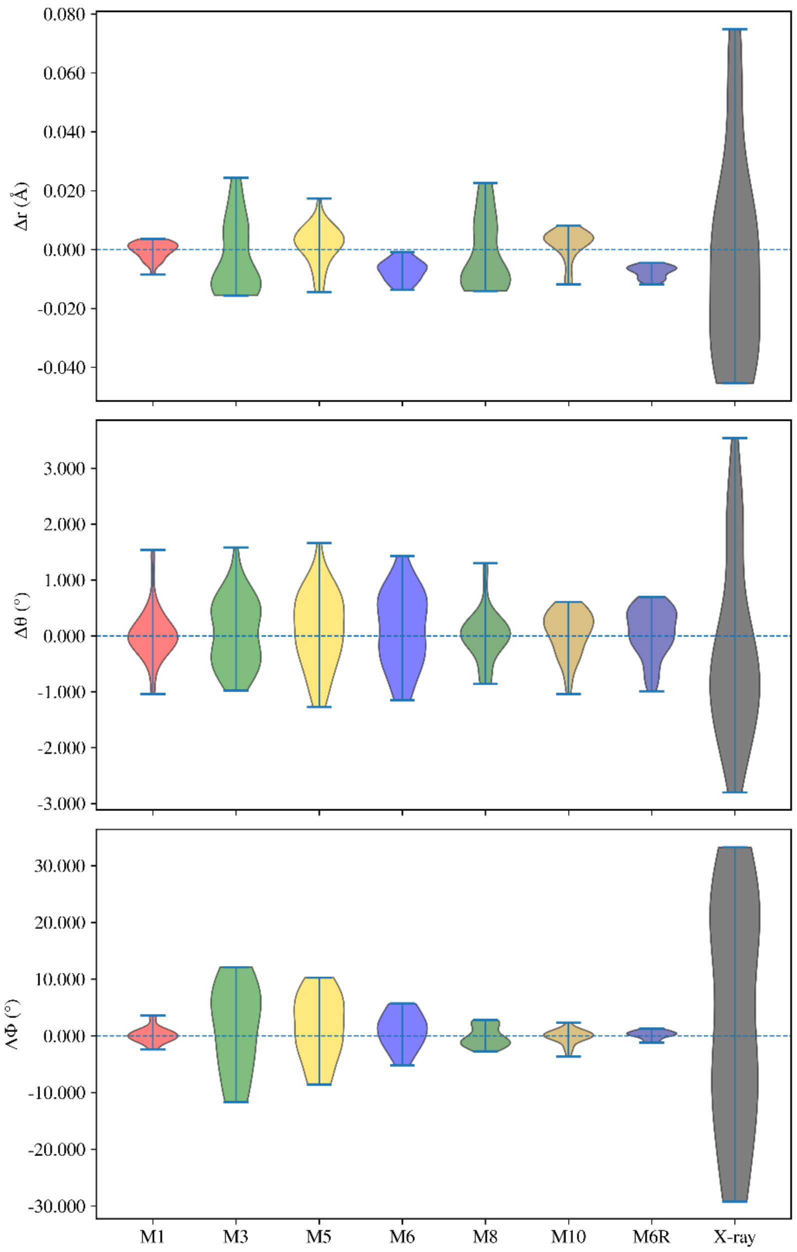

Supplementary Figure 220: Key coordinates of quantum refinement results of gentian violet cation. Deviation in the refined bond distances (Δr, n = 30), angles (Δθ, n = 42) and dihedrals (Δϕ, n = 24) of gentian violet cation (CVI) in type B chloramphenicol acetyltransferase from various quantum refinement schemes (**M1-M10**) and X-ray structure which are compared to those obtained from the most reliable **M7** scheme. The solid line represents the upper and lower values.

(xxxix) 1OWK (Substituted 2-Naphthamidine Inhibitors of Urokinase)

Protein preparations:

Resolution: 2.80 Å

Ligand: 303; $C_{24}H_{26}N_4O$

Residue flipped: ASN66, GLN167, HIS170, GLN206, HIS243

Protonation states (pH = 6.5):

GLH5, GLH10, HIP22, HIP45, GLH72, GLH76, GLH78, HIP83, HIP93, HIP94, GLH106, ASH127, GLH135, ASH146, HIP163, GLH165, HIP170, GLH175, HIP235, HIP243

CYX30-CYX46, CYX38-CYX109, CYX134-CYX203, CYX166-CYX182, CYX193-CYX221

Optimized region: 303

High layer: 303

Medium layer: HIP45, ASP49, ASP191, SER192, CYX193, GLN194, SER197, VAL215, SER216, TRP217, GLY220, CYX221, ALA222, GLY228, WAT249, WAT260, WAT262, WAT323

ω_α = 3.1890

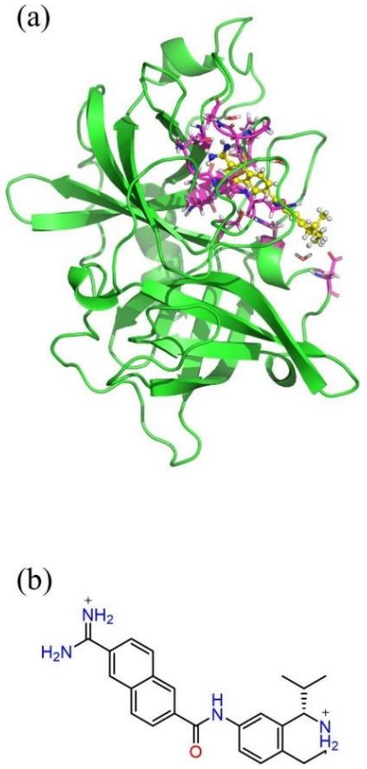

Supplementary Figure 221: Substituted 2-Naphthamidine Inhibitors of Urokinase. (**a**) Crystal structure of urokinase complexed with inhibitor 303. ONIOM layers by different colors: yellow: high layer; red: medium layer; green: low layer. Inhibitor 303 is presented in stick and balls. (**b**) Structure of inhibitor 303.

Quantum refined structural results:

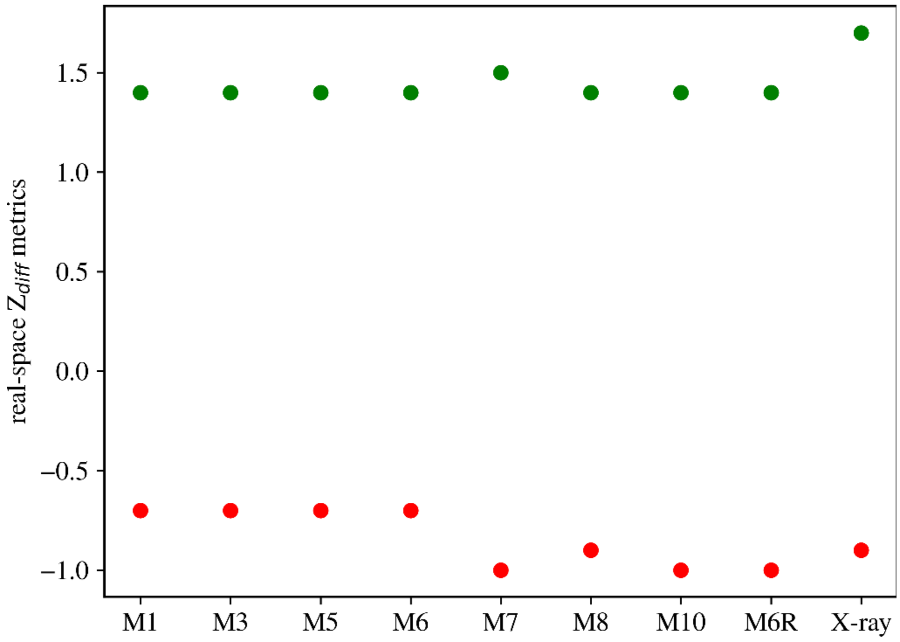

Supplementary Figure 222: Real-space Z-difference (RSZD) of inhibitor 303. RSZD+ (green) and RSZD- (red) scores of inhibitor 303 in urokinase from various quantum refinement schemes (**M1-M10**). Those results for X-ray were taken from the experimental structure without our further refinement.

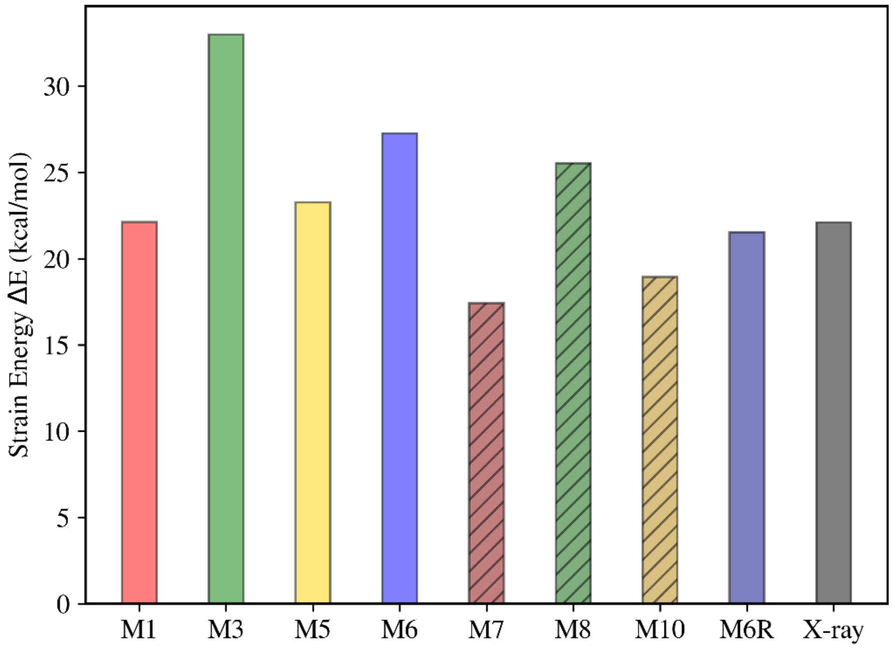

Supplementary Figure 223: Strain energy of inhibitor 303. Strain energy (ΔE, kcal·mol^{-1}) at ωB97X-D/6-31G(d) level for inhibitor 303 in urokinase determined by various quantum refinement schemes (**M1-M10**). Those results for X-ray were taken from the experimental structure without our further refinement.

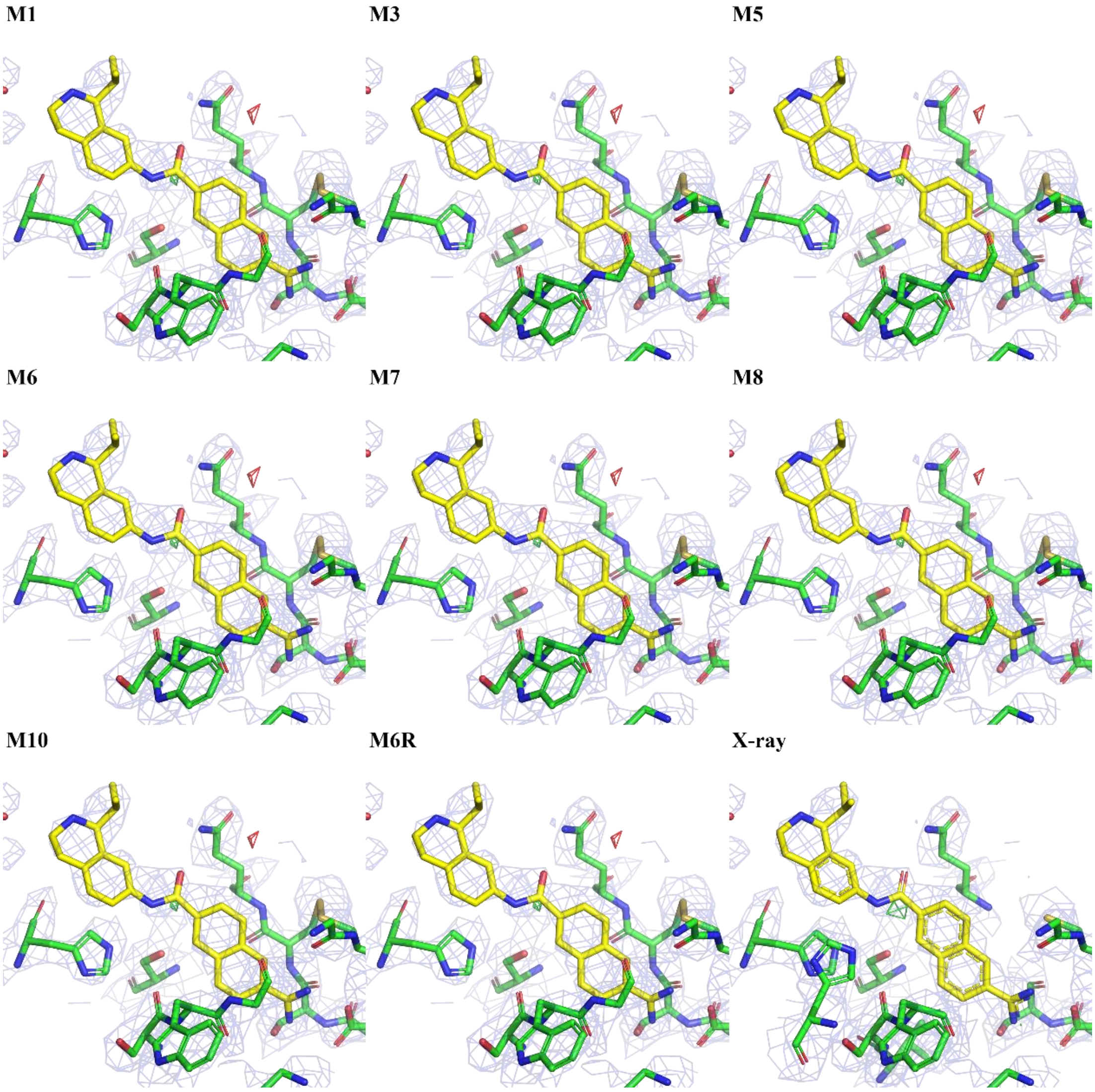

Supplementary Figure 224: Electron density maps of inhibitor 303. Structures for inhibitor 303 in urokinase from various quantum refinement schemes (**M1**-**M10**), including the electron density maps (2mFo-DFc maps, contoured at 1.0 σ (blue), mFo-DFc maps, contoured at +3.0 σ (green), and mFo-DFc maps, contoured at -3.0 σ (red)). Those results for X-ray were taken from the experimental structure without our further refinement.

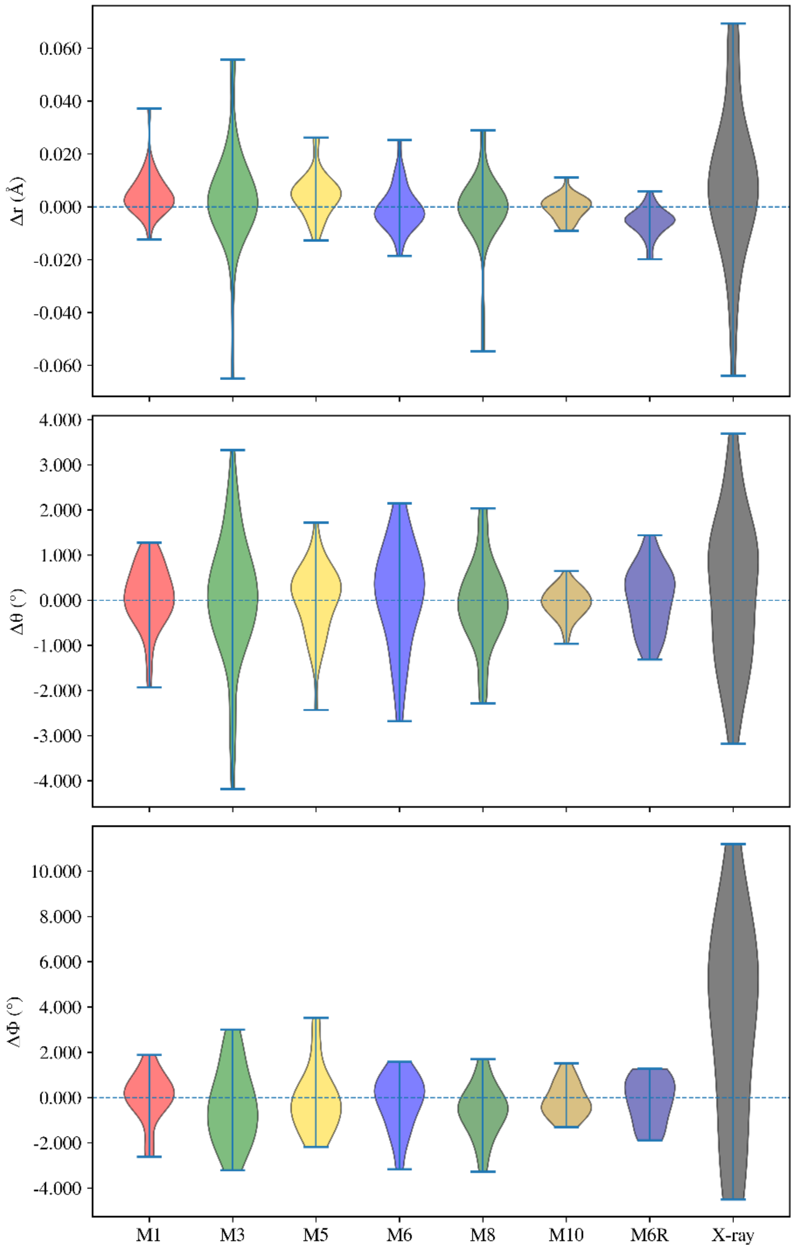

Supplementary Figure 225: Key coordinates of quantum refinement results of inhibitor 303. Deviation in the refined bond distances (Δr, n = 32), angles (Δθ, n = 46) and dihedrals (Δϕ, n = 16) of inhibitor 303 in urokinase from various quantum refinement schemes (**M1-M10**) and X-ray structure which are compared to those obtained from the most reliable **M7** scheme. The solid line represents the upper and lower values.

(xl) 2QBR (PTP1B-inhibitor complex)

Protein preparations:

Resolution: 2.50 Å

Ligand: 910; $C_{20}H_{16}BrNO_5S$

Residue flipped: HIS208

Protonation states (pH = 7.0):

HIP25, HID54, HID60, HID94, GLH97, HID173, HID175, HID208, HID214, HID296

Optimized region: 910

High layer: 910

Medium layer: YR46, ASP48, VAL49, LYS120, PHE182, SER216, ILE219, GLY220, ARG221, MET258, GLY259, GLN262, GLN266, WAT823, WAT901, WAT921, WAT922

ω_α = 1.0297

(a)

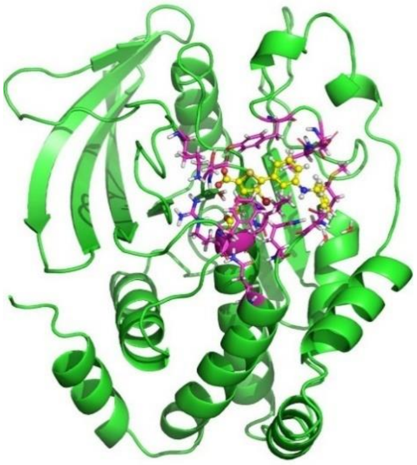

(b)

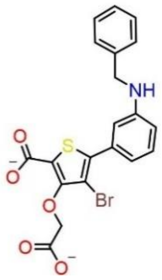

Supplementary Figure 226: PTP1B-inhibitor complex. (**a**) Crystal structure of PTP1B complexed with inhibitor 910. ONIOM layers by different colors: yellow: high layer; red: medium layer; green: low layer. Inhibitor 910 is presented in stick and balls. (**b**) Structure of inhibitor 910.

Quantum refined structural results:

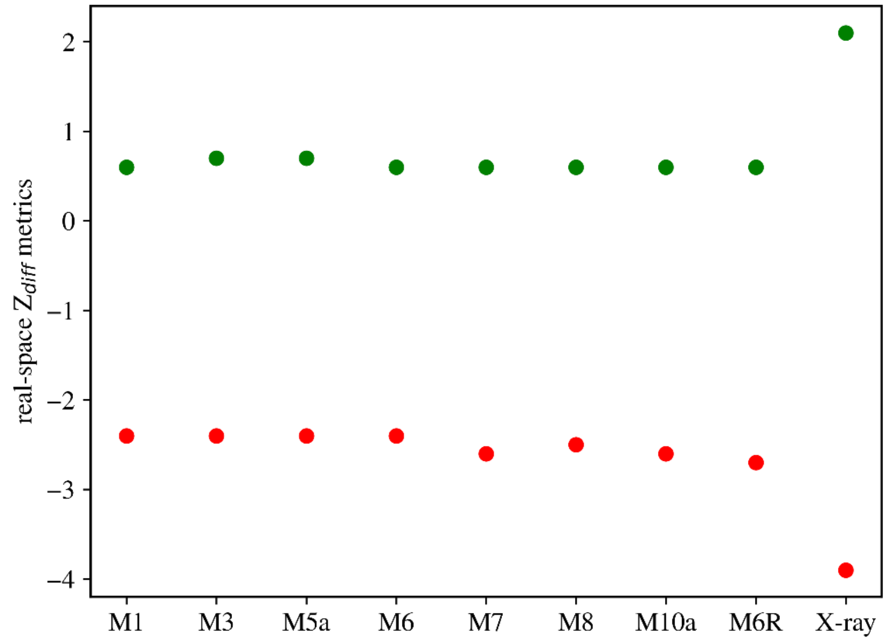

Supplementary Figure 227: Real-space Z-difference (RSZD) of inhibitor 910. RSZD+ (green) and RSZD- (red) scores of inhibitor 910 in PTP1B from various quantum refinement schemes (**M1-M10**). Those results for X-ray were taken from the experimental structure without our further refinement.

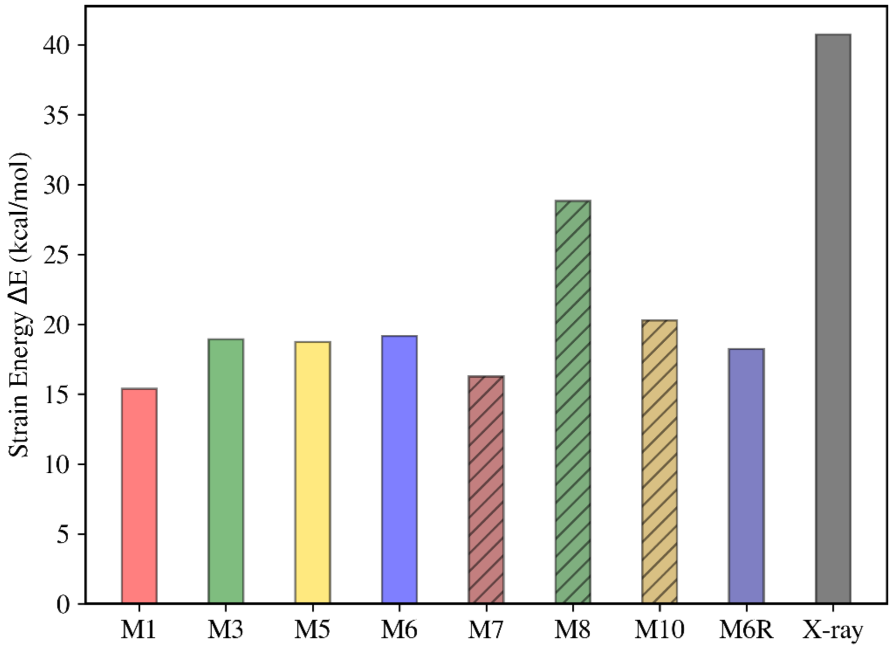

Supplementary Figure 228: Strain energy of inhibitor 910. Strain energy (ΔE, kcal·mol^{-1}) at ωB97X-D/6-31G(d) level for inhibitor 910 in PTP1B determined by various quantum refinement schemes (**M1-M10**). Those results for X-ray were taken from the experimental structure without our further refinement.

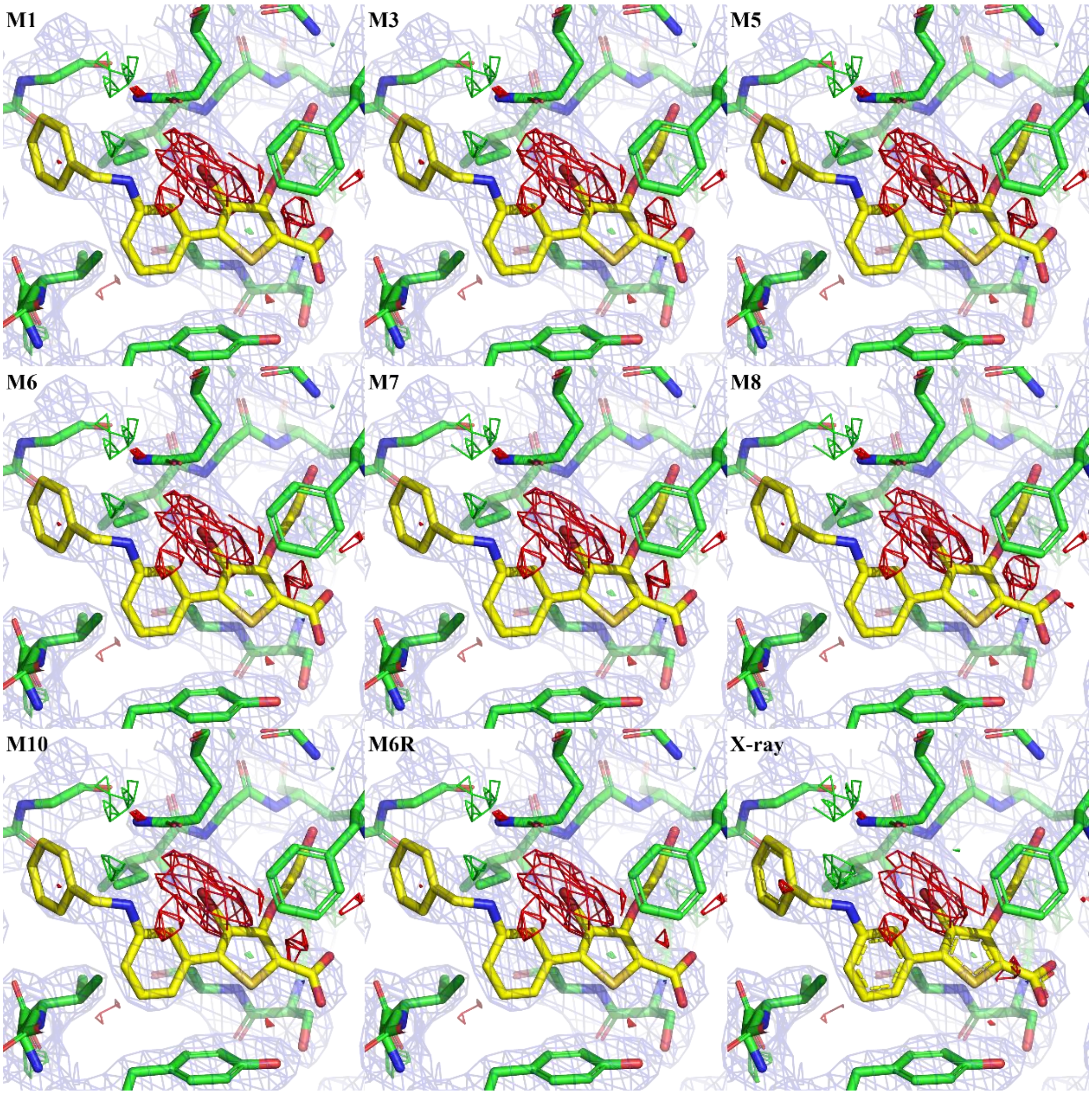

Supplementary Figure 229: Electron density maps of inhibitor 910. Structures for inhibitor 910 in PTP1B from various quantum refinement schemes (**M1**-**M10**), including the electron density maps (2mFo-DFc maps, contoured at 1.0 σ (blue), mFo-DFc maps, contoured at +3.0 σ (green), and mFo-DFc maps, contoured at -3.0 σ (red)). Those results for X-ray were taken from the experimental structure without our further refinement.

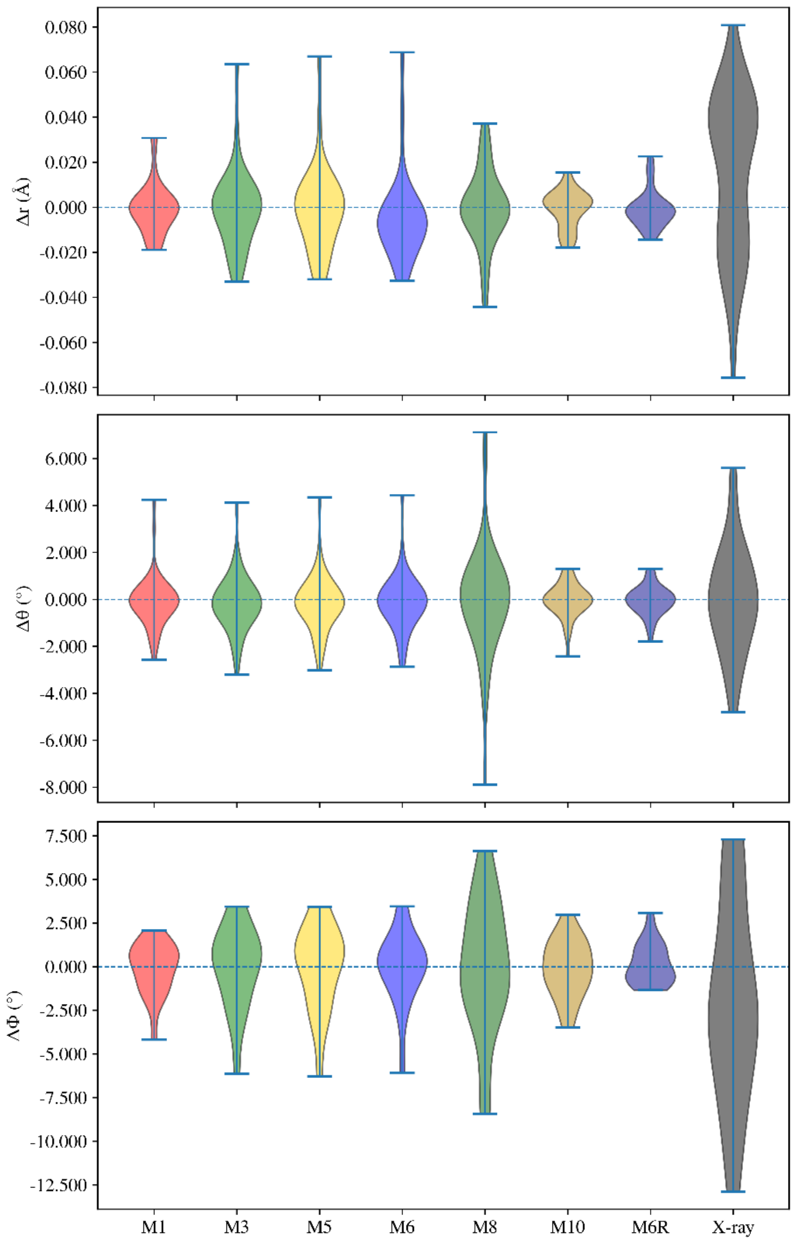

Supplementary Figure 230: Key coordinates of quantum refinement results of inhibitor 910. Deviation in the refined bond distances (Δr, n = 30), angles (Δθ, n = 41) and dihedrals (Δϕ, n = 18) of inhibitor 910 in PTP1B from various quantum refinement schemes (**M1-M10**) and X-ray structure which are compared to those obtained from the most reliable **M7** scheme. The solid line represents the upper and lower values.

(xli) 1YAT (Improved calcineurin inhibition by yeast FKBP12-drug complexes)

Protein preparations:

Resolution: 2.50 Å

Ligand: FK5 (Tacrolimus); $C_{44}H_{69}NO_{12}$

Residue flipped: GLN47, GLN54

Protonation states (pH = 8.75):

HID25

Optimized region: FK5

High layer: FK5

Medium layer: TYR26, PHE36, ASP37, ARG42, PHE46, GLN54, VAL55, ILE56, TRP59, ALA81, TYR82, LEU90, ILE91, PHE99, WAT219, WAT238

ω_α = 0.89566

(a)

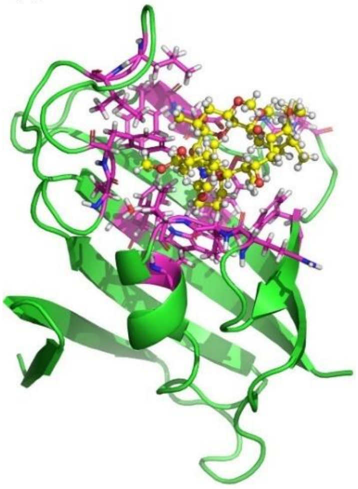

(b)

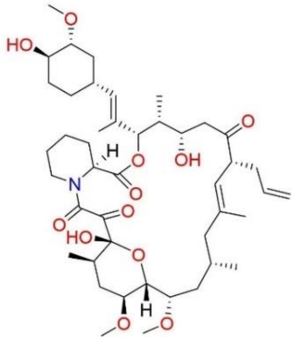

Supplementary Figure 231: Improved calcineurin inhibition by yeast FKBP12-drug complexes. (**a**) Crystal structure of calcineurin complexed with tacrolimus (FK5). ONIOM layers by different colors: yellow: high layer; red: medium layer; green: low layer. Ligand tacrolimus is presented in stick and balls. (**b**) Structure of tacrolimus.

Quantum refined structural results:

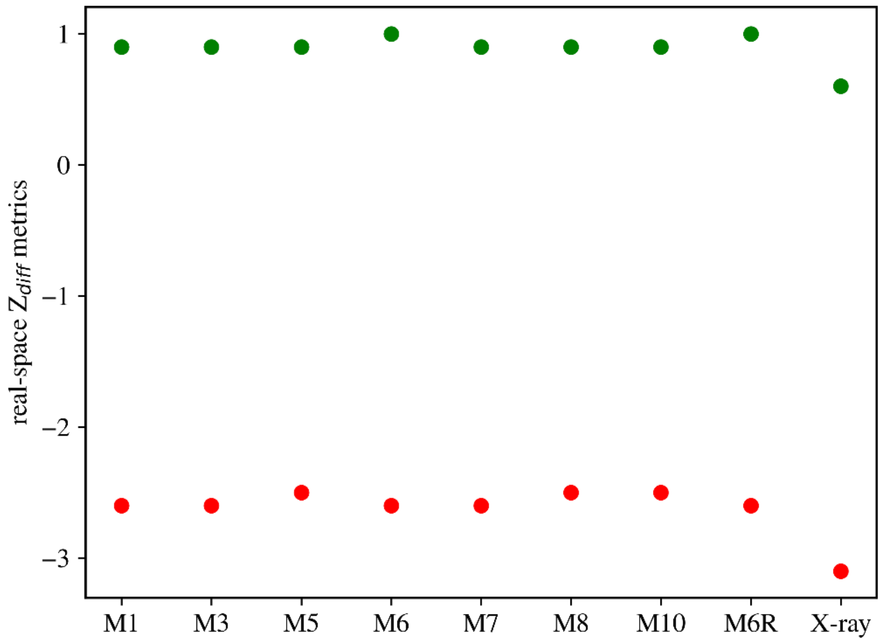

Supplementary Figure 232: Real-space Z-difference (RSZD) of tacrolimus. RSZD+ (green) and RSZD- (red) scores of tacrolimus (FK5) in calcineurin from various quantum refinement schemes (**M1-M10**). Those results for X-ray were taken from the experimental structure without our further refinement.

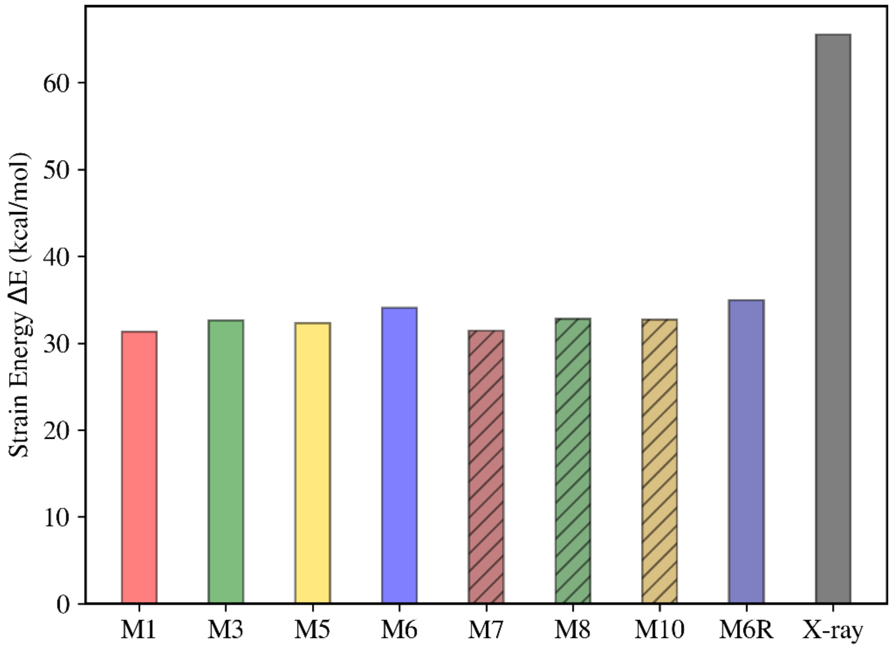

Supplementary Figure 233: Strain energy of tacrolimus. Strain energy (ΔE, kcal·mol^{-1}) at ωB97X-D/6-31G(d) level for tacrolimus (FK5) in calcineurin determined by various quantum refinement schemes (**M1-M10**). Those results for X-ray were taken from the experimental structure without our further refinement.

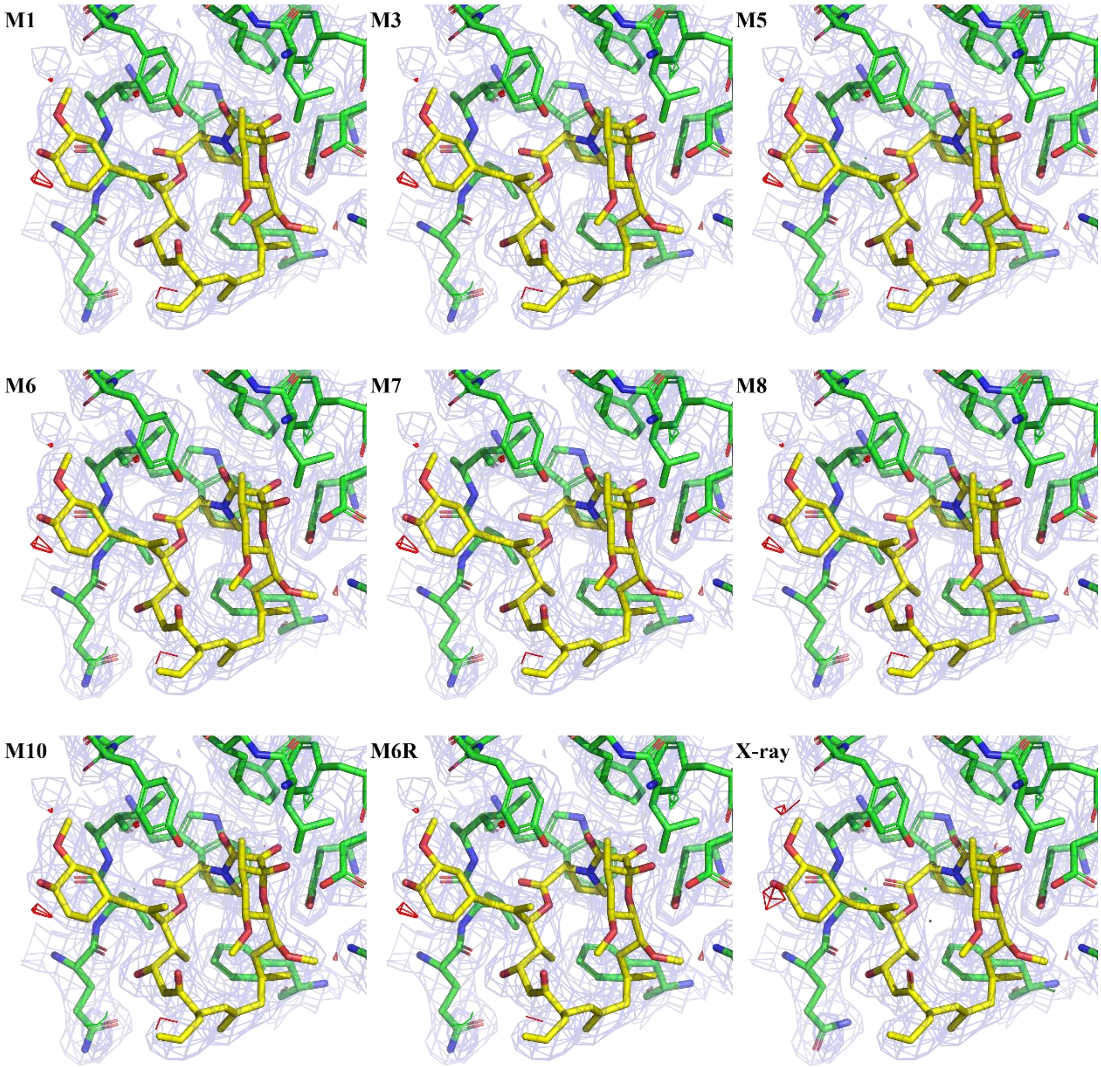

Supplementary Figure 234: Electron density maps of tacrolimus. Structures for tacrolimus (FK5) in calcineurin from various quantum refinement schemes (**M1**-**M10**), including the electron density maps (2mFo-DFc maps, contoured at 1.0 σ (blue), mFo-DFc maps, contoured at +3.0 σ (green), and mFo-DFc maps, contoured at -3.0 σ (red)). Those results for X-ray were taken from the experimental structure without our further refinement.

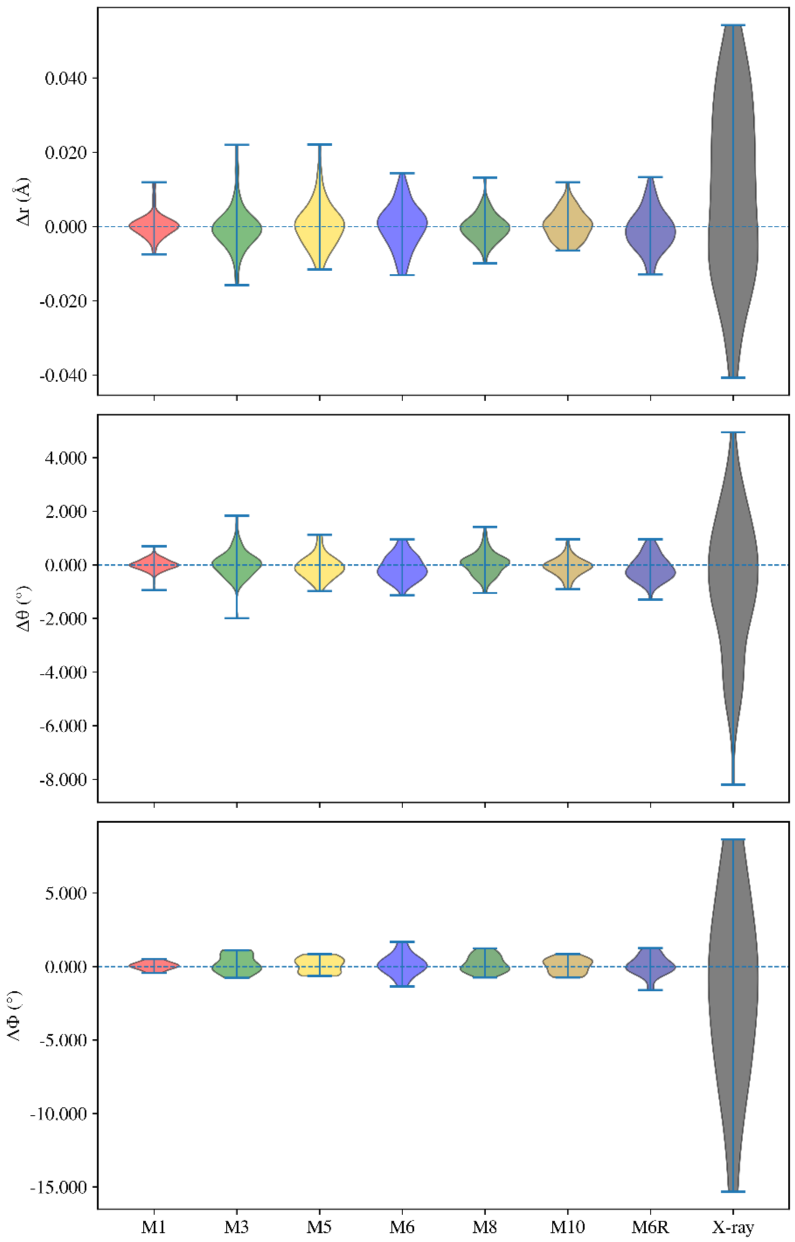

Supplementary Figure 235: Key coordinates of quantum refinement results of tacrolimus. Deviation in the refined bond distances (Δr, n = 60), angles (Δθ, n = 86) and dihedrals (Δϕ, n = 15) of tacrolimus (FK5) in calcineurin from various quantum refinement schemes (**M1-M10**) and X-ray structure which are compared to those obtained from the most reliable **M7** scheme. The solid line represents the upper and lower values.

(xlii) 1XP9 (Human estrogen receptor alpha ligand-binding domain in complex with compound 18)

Protein preparations:

Resolution: 1.80 Å

Ligand: AIJ; $C_{27}H_{29}NO_4S$

Residue flipped: HIS474, ASN532, HIS547

Protonation states (pH = 7.1):

HID356, HID373, HID377, HID398, HID474, HID476, HID488, HID501, HID513, HID516, HID524, HID547, HID550

Optimized region: AIJ

High layer: AIJ

Medium layer: LEU346, THR347, LEU349, ALA350, ASP351, GLU353, LEU354, TRP383, LEU384, LEU387, MET388, LEU391, ARG394, PHE404, MET421, ILE424, GLY521, HID524, LEU525, LYS531, LEU536, WAT1006, WAT1095

ω_α = 1.3215

(a)

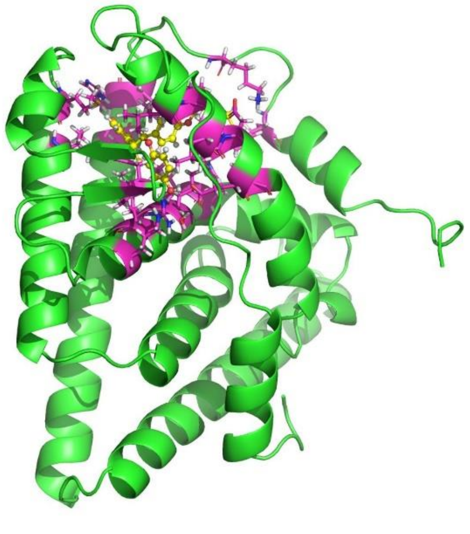

(b)

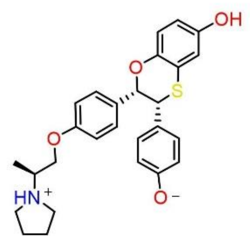

Supplementary Figure 236: Human estrogen receptor alpha ligand-binding domain in complex with compound 18. (**a**) Crystal structure of human estrogen receptor alpha complexed with inhibitor AIJ. ONIOM layers by different colors: yellow: high layer; red: medium layer; green: low layer. Inhibitor AIJ is presented in stick and balls. (**b**) Structure of inhibitor AIJ.

Quantum refined structural results:

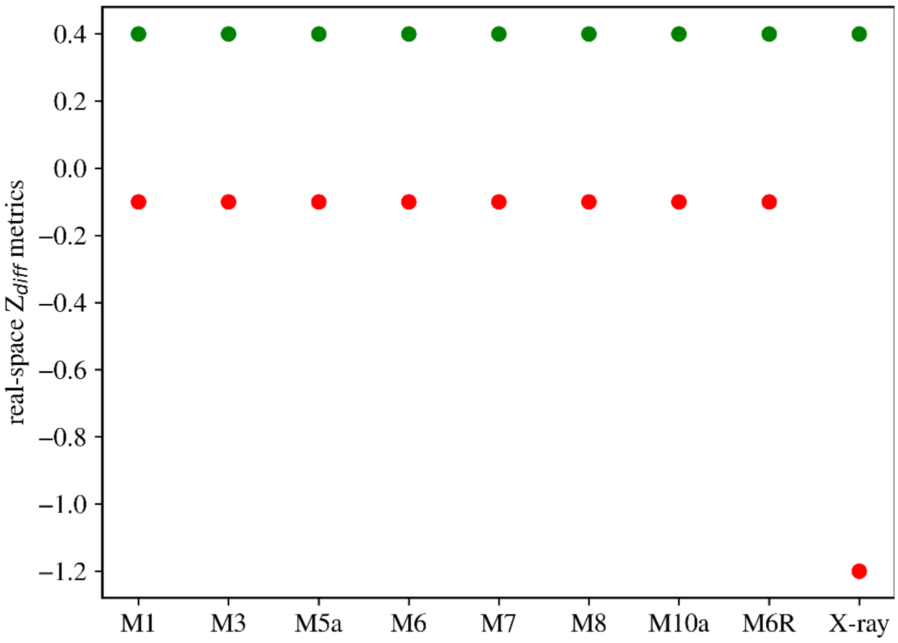

Supplementary Figure 237: Real-space Z-difference (RSZD) of inhibitor AIJ. RSZD+ (green) and RSZD- (red) scores of inhibitor AIJ in human estrogen receptor alpha from various quantum refinement schemes (**M1-M10**). Those results for X-ray were taken from the experimental structure without our further refinement.

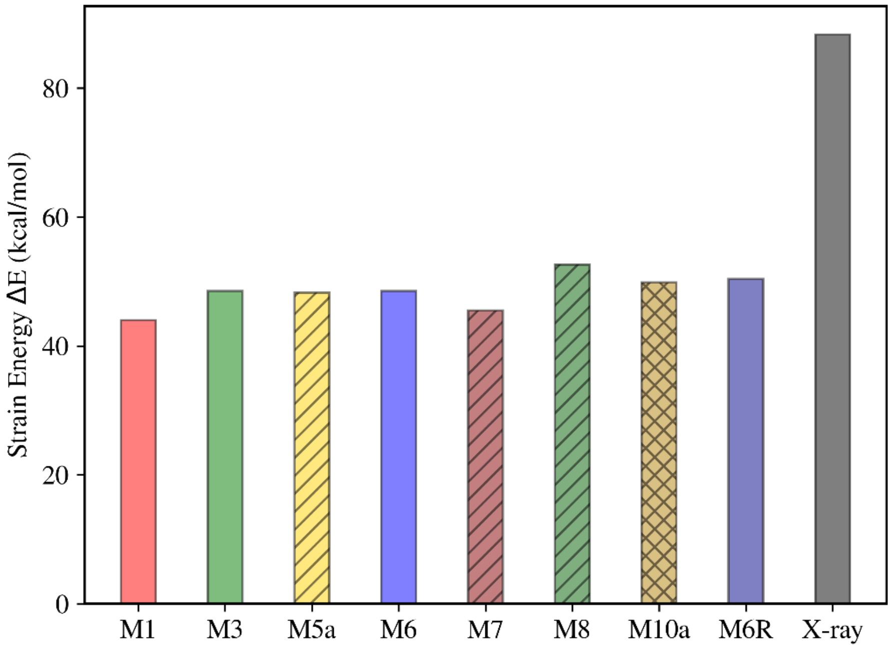

Supplementary Figure 238: Strain energy of inhibitor AIJ. Strain energy (ΔE, kcal·mol^{-1}) at ωB97X-D/6-31G(d) level for inhibitor AIJ in human estrogen receptor alpha determined by various quantum refinement schemes (**M1-M10**). Those results for X-ray were taken from the experimental structure without our further refinement.

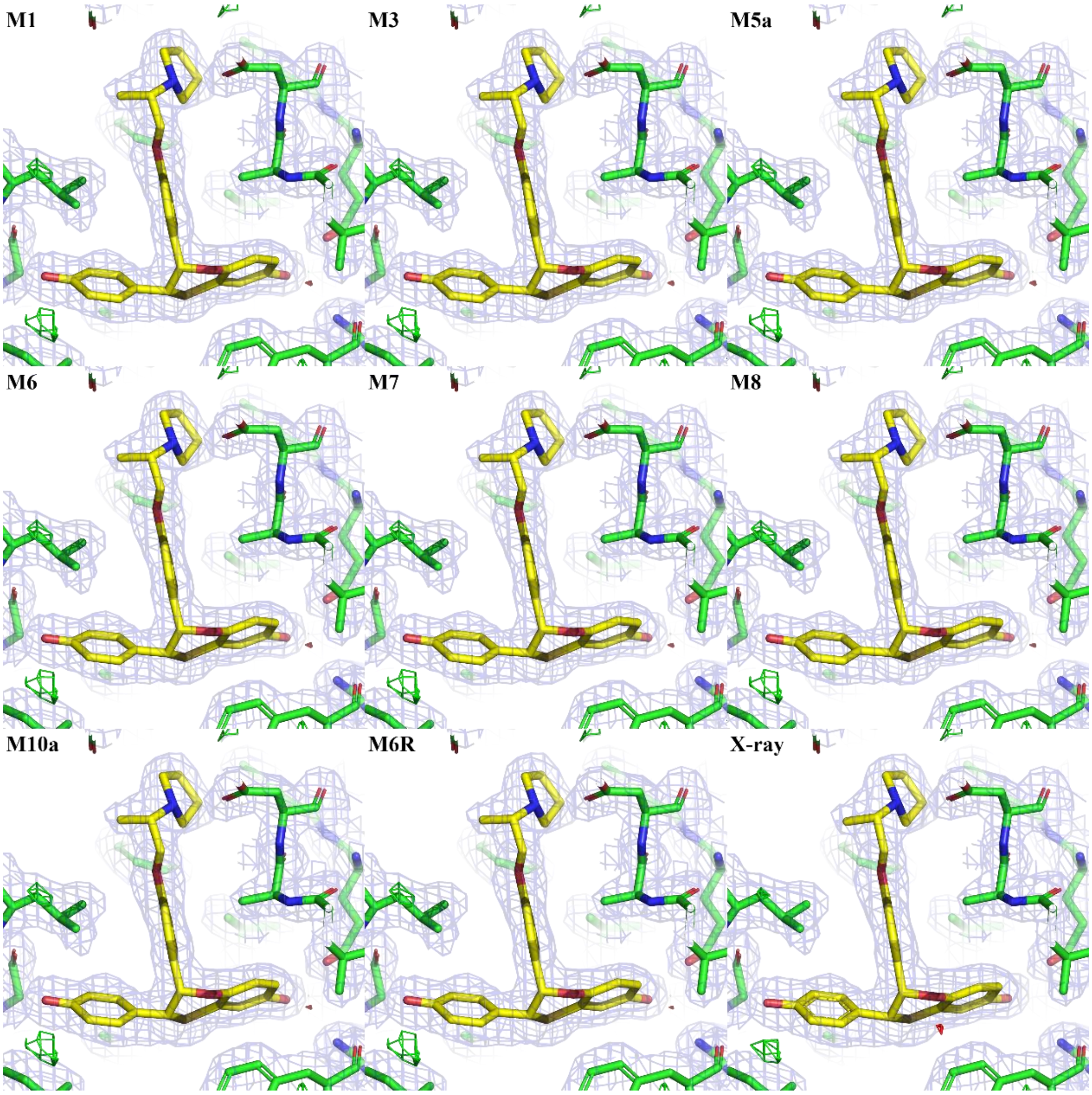

Supplementary Figure 239: Electron density maps of inhibitor AIJ. Structures for inhibitor AIJ in human estrogen receptor alpha from various quantum refinement schemes (**M1-M10**), including the electron density maps (2mFo-DFc maps, contoured at 1.0 σ (blue), mFo-DFc maps, contoured at +3.0 σ (green), and mFo-DFc maps, contoured at -3.0 σ (red)). Those results for X-ray were taken from the experimental structure without our further refinement.

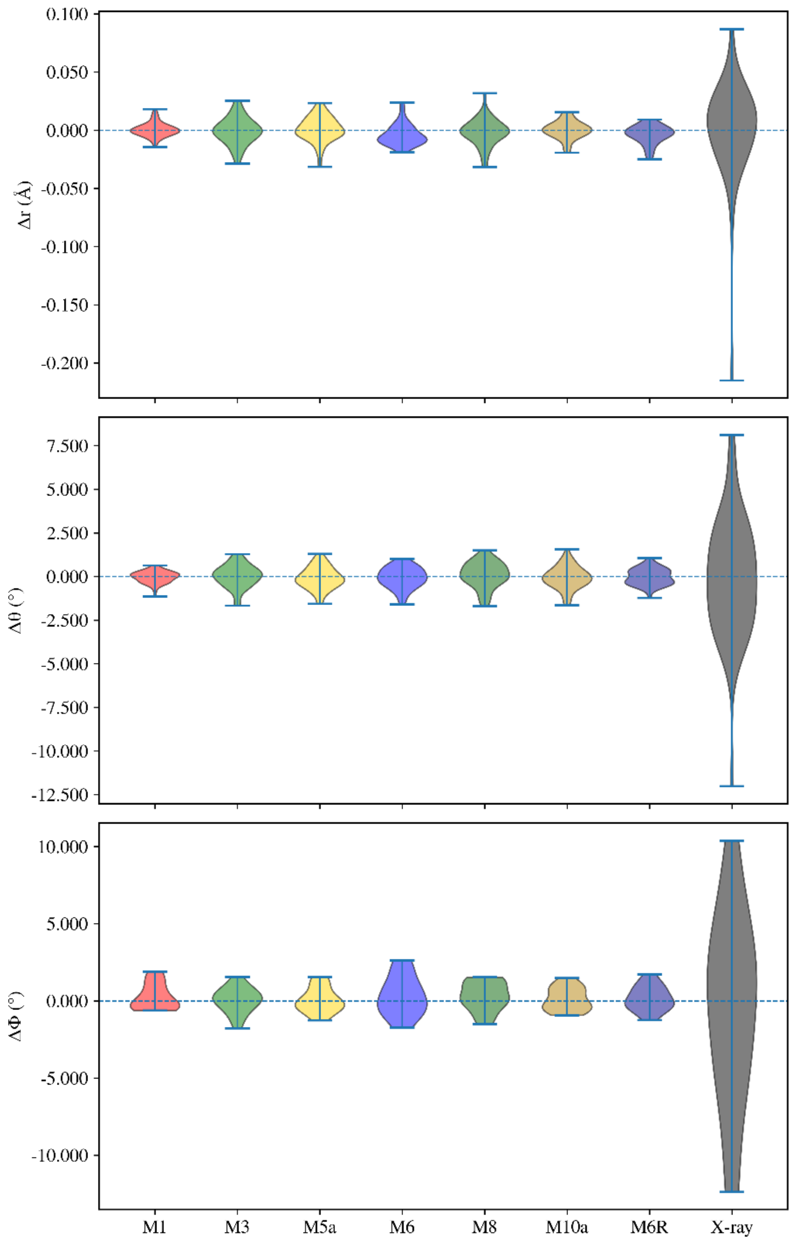

Supplementary Figure 240: Key coordinates of quantum refinement results of inhibitor AIJ. Deviation in the refined bond distances (Δr, n = 37), angles (Δθ, n = 52) and dihedrals (Δϕ, n = 17) of inhibitor AIJ in human estrogen receptor alpha from various quantum refinement schemes (**M1-M10**) and X-ray structure which are compared to those obtained from the most reliable **M7** scheme. The solid line represents the upper and lower values.

(xliii) 2HXM (Complex of UNG2 and a small Molecule synthetic Inhibitor)

Protein preparations:

Resolution: 1.30 Å

Ligand: 302; $C_{15}H_{14}N_4O_6$

Residue flipped: GLN161, ASN236, GLN250

Protonation states (pH = 8.0):

HID92, HID115, HID123, HID148, HID154, HID186, HID189, HID212, HID217, HID261, HID262, HID268, HID283

Optimized region: 302

High layer: 302

Medium layer: GLY143, GLN144, ASP145, TYR147, CYS157, PHE158, SER169, ASN204, GLY246, SER247, TYR248, HID268, SER270, WAT405, WAT410, WAT434, WAT438, WAT455, WAT472, WAT499, WAT528, WAT558, WAT576, WAT652, WAT671, WAT713

ω_α = 0.13136

(a)

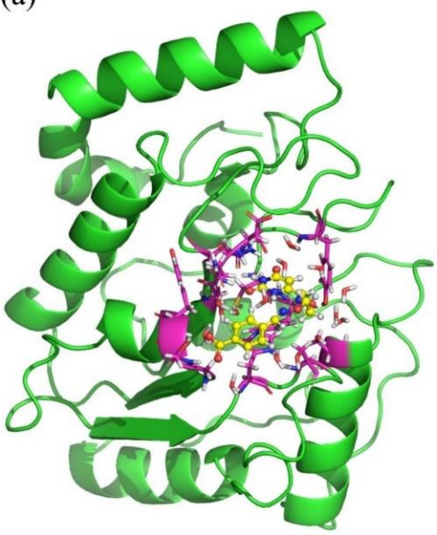

(b)

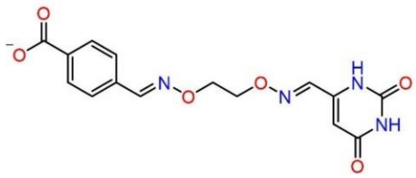

Supplementary Figure 241: Complex of UNG2 and a small Molecule synthetic Inhibitor. (**a**) Crystal structure of UNG2 complexed with inhibitor 302. ONIOM layers by different colors: yellow: high layer; red: medium layer; green: low layer. Inhibitor 302 is presented in stick and balls. (**b**) Structure of inhibitor 302.

Quantum refined structural results:

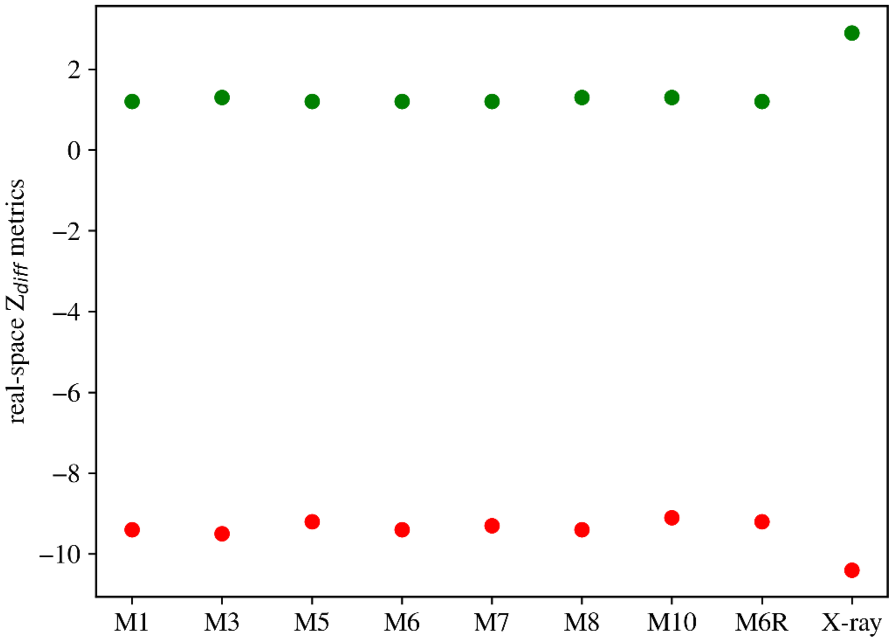

Supplementary Figure 242: Real-space Z-difference (RSZD) of inhibitor 302. RSZD+ (green) and RSZD- (red) scores of inhibitor 302 in UNG2 from various quantum refinement schemes (**M1-M10**). Those results for X-ray were taken from the experimental structure without our further refinement.

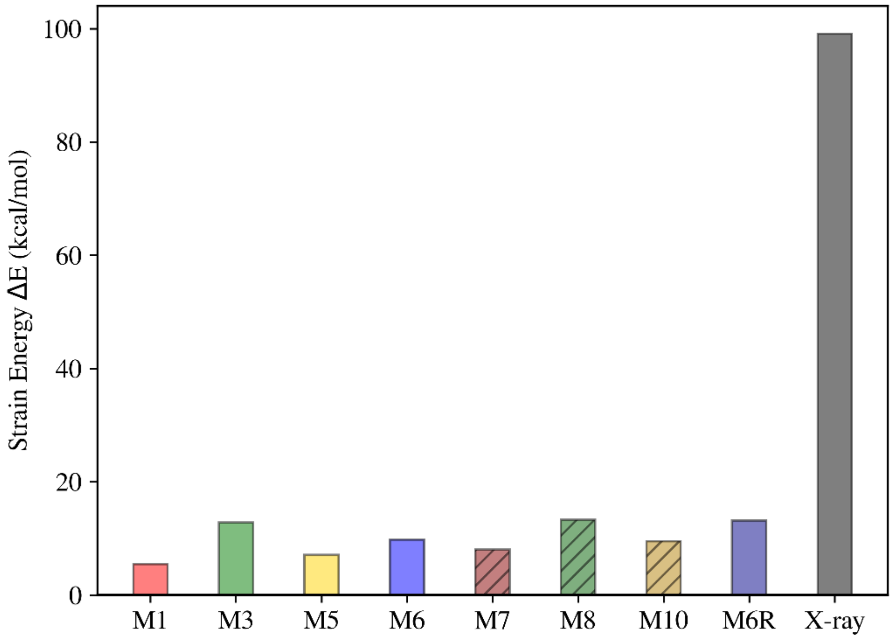

Supplementary Figure 243: Strain energy of inhibitor 302. Strain energy (ΔE, kcal·mol^{-1}) at ωB97X-D/6-31G(d) level for inhibitor 302 in UNG2 determined by various quantum refinement schemes (**M1-M10**). Those results for X-ray were taken from the experimental structure without our further refinement.

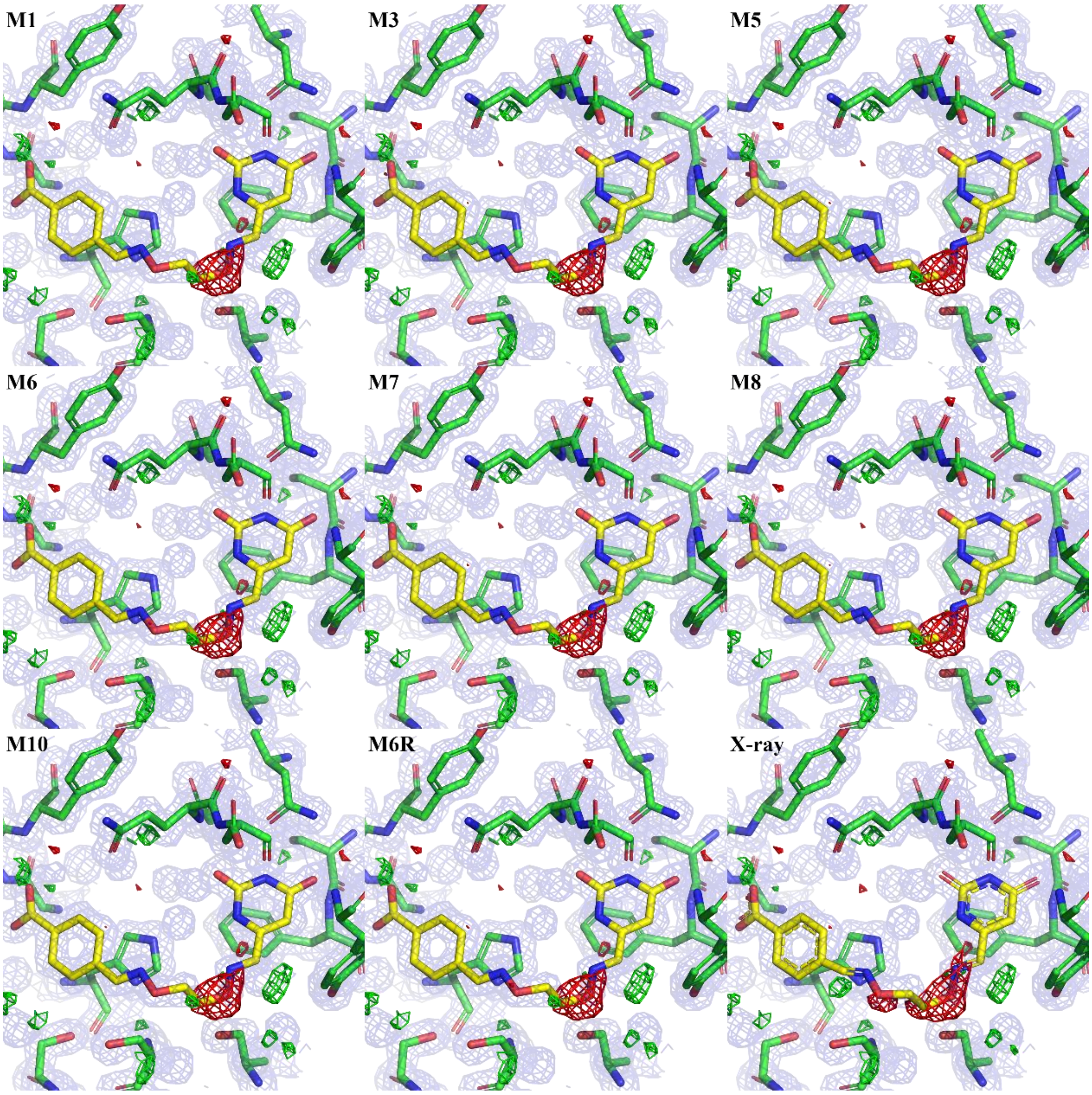

Supplementary Figure 244: Electron density maps of inhibitor 302. Structures for inhibitor 302 in UNG2 from various quantum refinement schemes (**M1**-**M10**), including the electron density maps (2mFo-DFc maps, contoured at 1.0 σ (blue), mFo-DFc maps, contoured at +3.0 σ (green), and mFo-DFc maps, contoured at -3.0 σ (red)). Those results for X-ray were taken from the experimental structure without our further refinement.

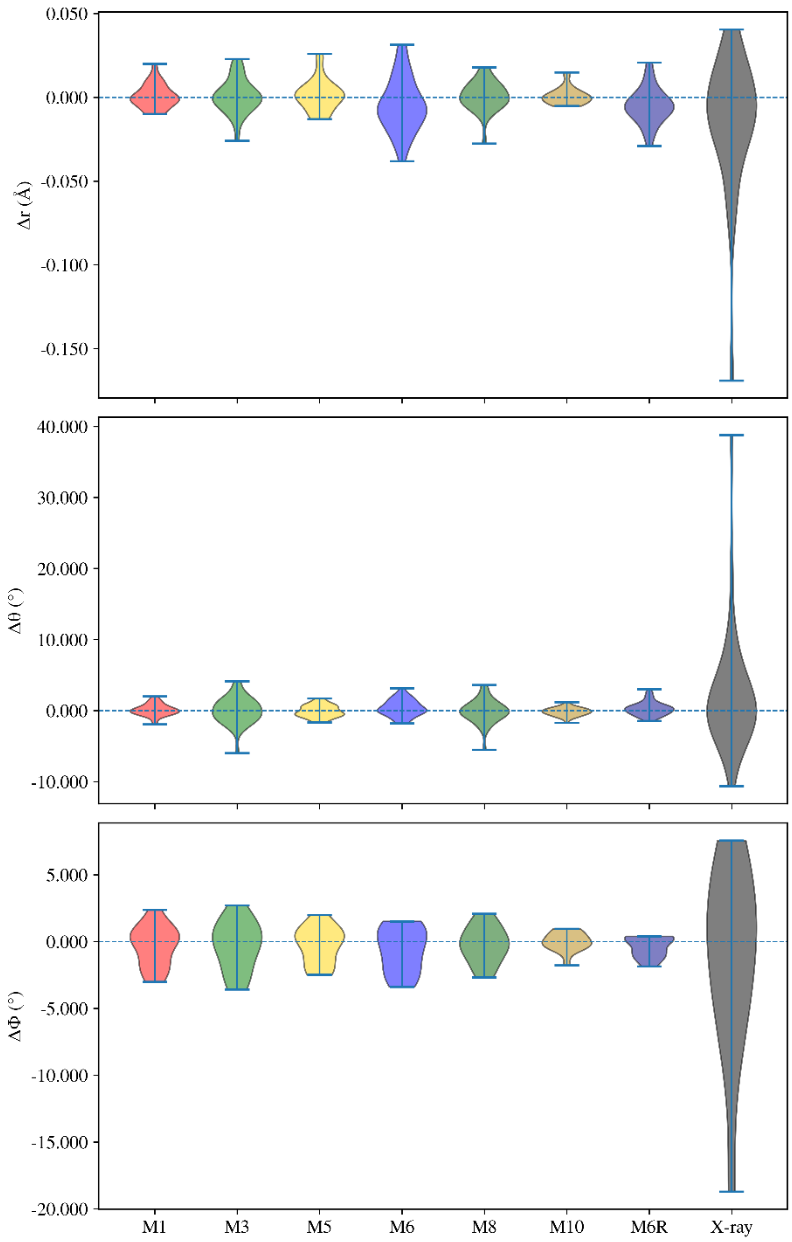

Supplementary Figure 245: Key coordinates of quantum refinement results of inhibitor 302. Deviation in the refined bond distances (Δr, n = 26), angles (Δθ, n = 33) and dihedrals (Δϕ, n = 14) of inhibitor 302 in UNG2 from various quantum refinement schemes (**M1-M10**) and X-ray structure which are compared to those obtained from the most reliable **M7** scheme. The solid line represents the upper and lower values.

(xliv) 2WCA (BtGH84 in complex with n-butyl pugnac)

Protein preparations:

Resolution: 2.30 Å

Ligand: NP6; $C_{17}H_{23}N_3O_7$

Residue flipped: GLN6, GLN10, GLN149, ASN156, ASN292, GLN543, ASN581

Protonation states (pH = 6.0):

HIP36, HID77, HIP143, HID171, HID207, ASH242, HID349, HID376, HID424, HIP433, GLH439, GLH494, HID500, HIP530, HIP583

Optimized region: NP6

High layer: NP6

Medium layer: GLY135, PHE136, TYR137, CYS278, TYR282, TRP286, THR310, VAL314, ILE315, TRP337, ASN339, VAL342, ASP344, TYR345, ASN372, HIP433, WAT2194

ω_α = 1.2692

(a)

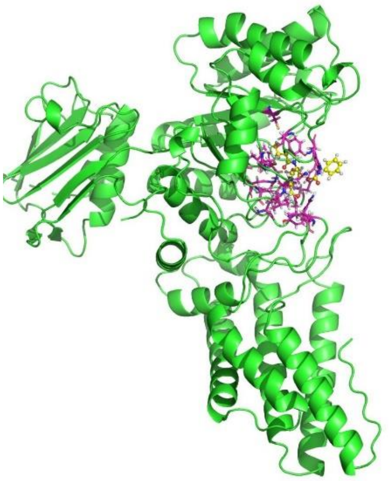

(b)

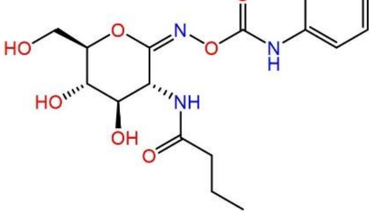

Supplementary Figure 246: BtGH84 in complex with n-butyl pugnac. (**a**) Crystal structure of BtGH84 complexed with inhibitor NP6. ONIOM layers by different colors: yellow: high layer; red: medium layer; green: low layer. Inhibitor NP6 is presented in stick and balls. (**b**) Structure of inhibitor NP6.

Quantum refined structural results:

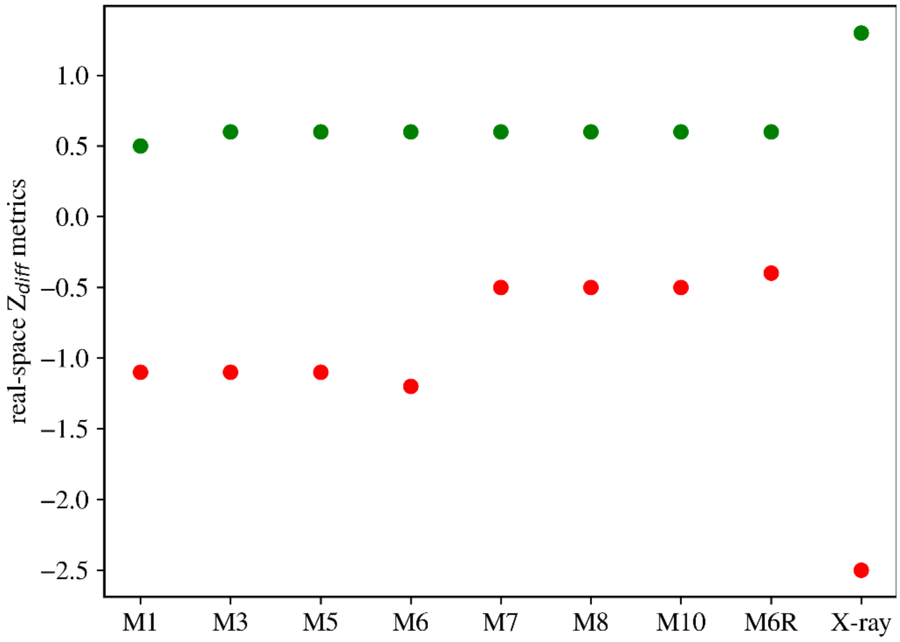

Supplementary Figure 247: Real-space Z-difference (RSZD) of inhibitor NP6. RSZD+ (green) and RSZD- (red) scores of inhibitor NP6 in BtGH84 from various quantum refinement schemes (**M1-M10**). Those results for X-ray were taken from the experimental structure without our further refinement.

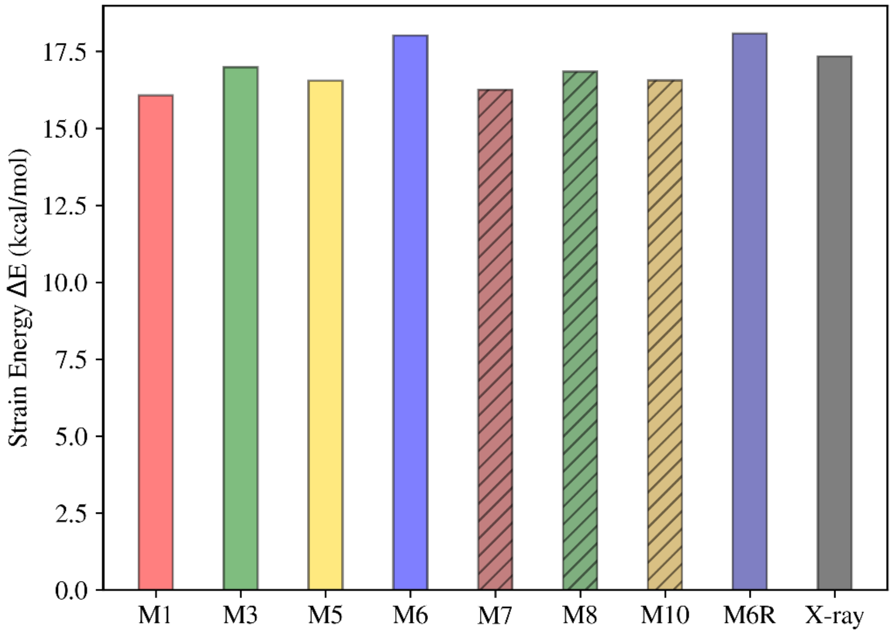

Supplementary Figure 248: Strain energy of inhibitor NP6. Strain energy (ΔE, kcal·mol^{-1}) at ωB97X-D/6-31G(d) level for inhibitor NP6 in BtGH84 determined by various quantum refinement schemes (**M1-M10**). Those results for X-ray were taken from the experimental structure without our further refinement.

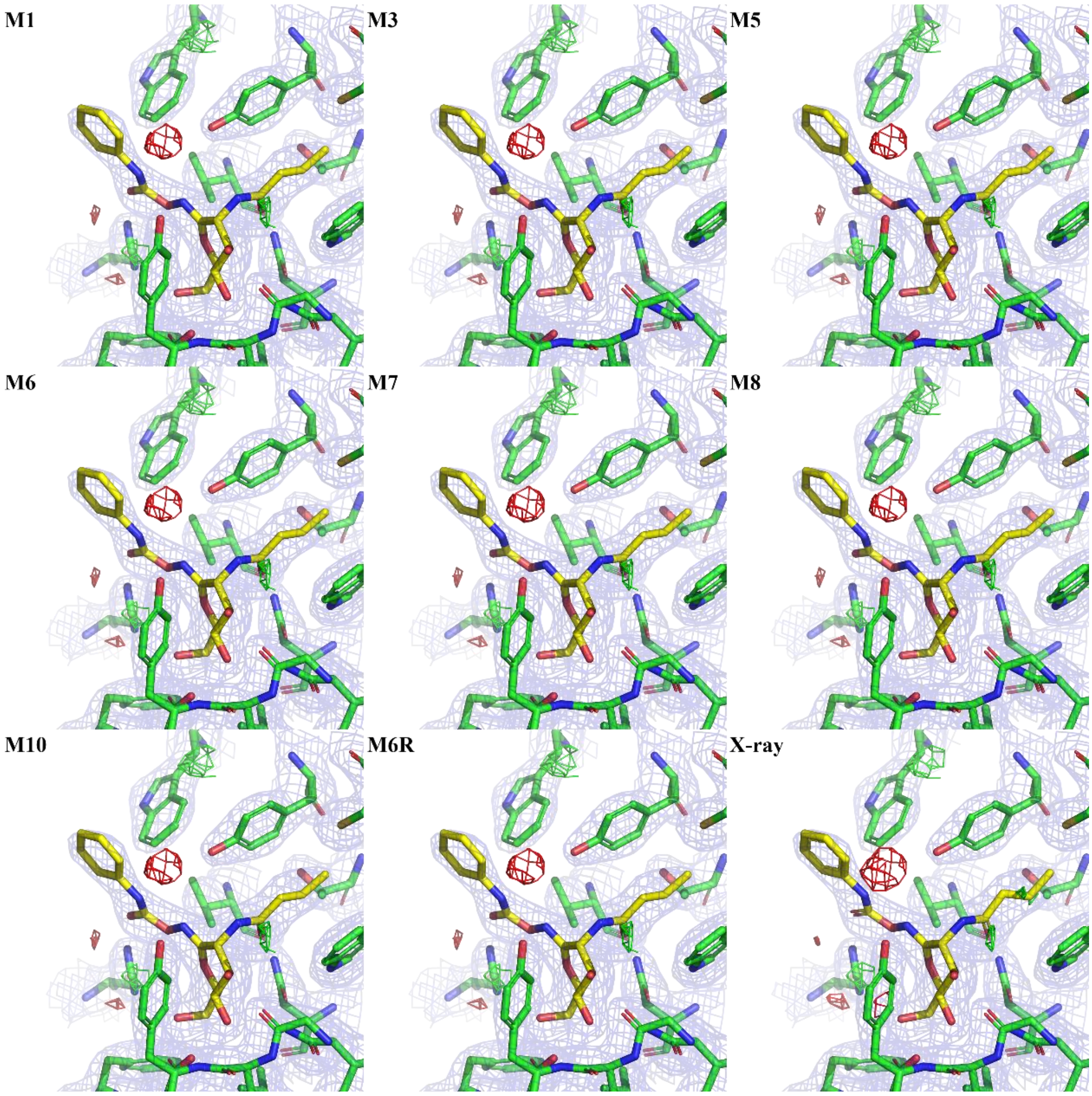

Supplementary Figure 249: Electron density maps of inhibitor NP6. Structures for inhibitor NP6 in BtGH84 from various quantum refinement schemes (**M1**-**M10**), including the electron density maps (2mFo-DFc maps, contoured at 1.0 σ (blue), mFo-DFc maps, contoured at +3.0 σ (green), and mFo-DFc maps, contoured at -3.0 σ (red)). Those results for X-ray were taken from the experimental structure without our further refinement.

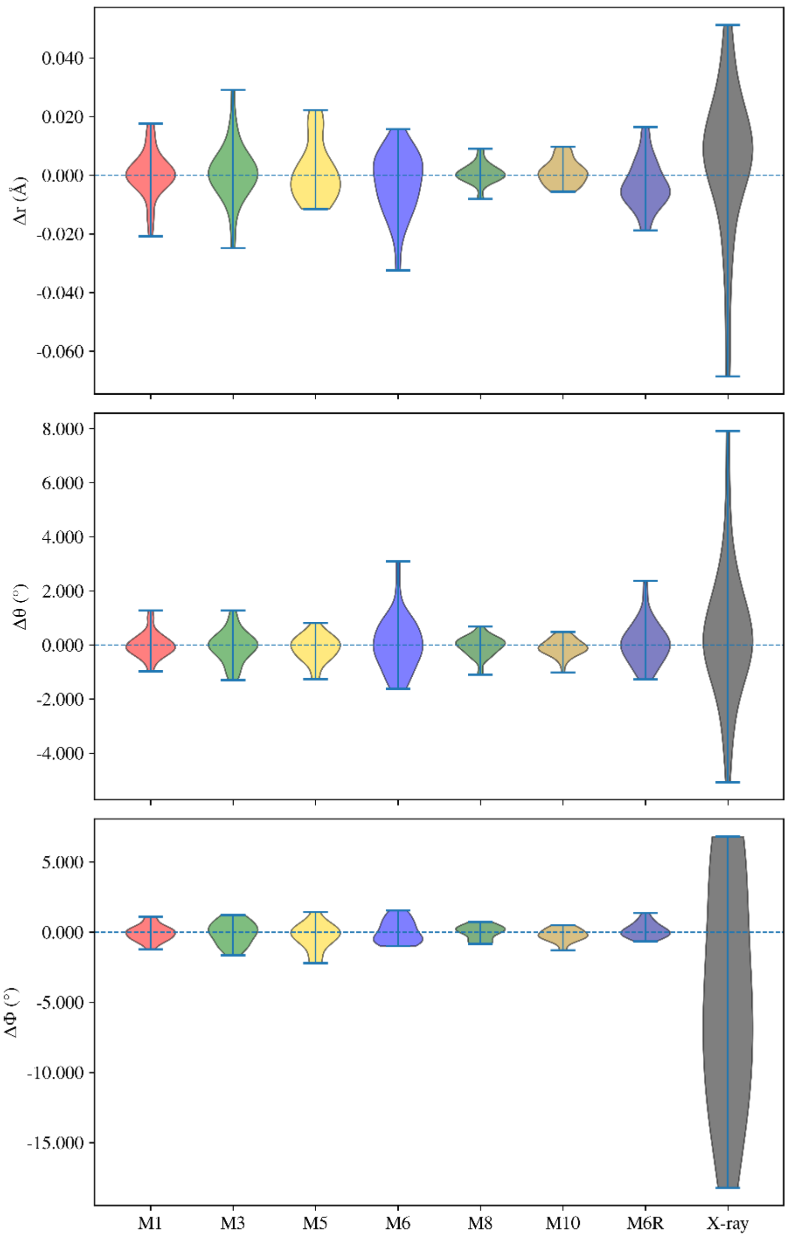

Supplementary Figure 250: Key coordinates of quantum refinement results of inhibitor NP6. Deviation in the refined bond distances (Δr, n = 28), angles (Δθ, n = 37) and dihedrals (Δϕ, n = 16) of inhibitor NP6 in BtGH84 from various quantum refinement schemes (**M1-M10**) and X-ray structure which are compared to those obtained from the most reliable **M7** scheme. The solid line represents the upper and lower values.

(xlv) 2WI4 (Orally Active 2-Amino Thienopyrimidine Inhibitors of the HSP90 Chaperone)

Protein preparations:

Resolution: 2.40 Å

Ligand: ZZ4; $C_{16}H_{11}Cl_2N_5$

Residue flipped: GLN28, ASN35, ASN40

Protonation states (pH = 6.5):

HID77, HID154, HID189, HID210

Optimized region: ZZ4

High layer: ZZ4

Medium layer: ASN51, SER52, ALA55, ASP93, MET98, ASP102, LEU107, PHE138, VAL150, THR184, WAT2031, WAT2037, WAT2083, WAT2114, WAT2140

ω_α = 1.6793

(a)

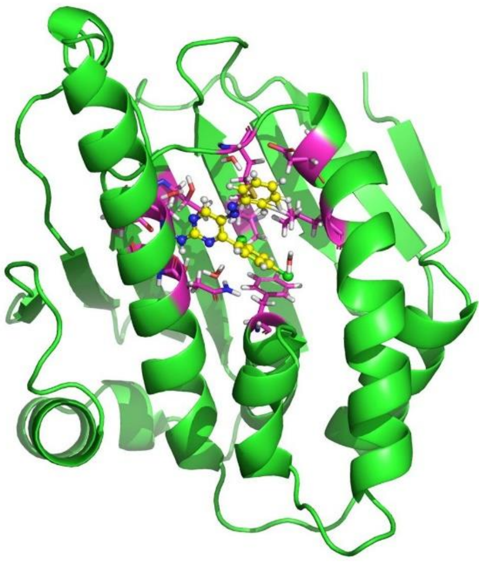

(b)

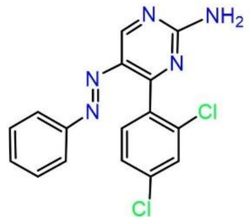

Supplementary Figure 251: Orally Active 2-Amino Thienopyrimidine Inhibitors of the HSP90 Chaperone. (**a**) Crystal structure of HSP90 chaperone complexed with inhibitor ZZ4. ONIOM layers by different colors: yellow: high layer; red: medium layer; green: low layer. Inhibitor ZZ4 is presented in stick and balls. (**b**) Structure of inhibitor ZZ4.

Quantum refined structural results:

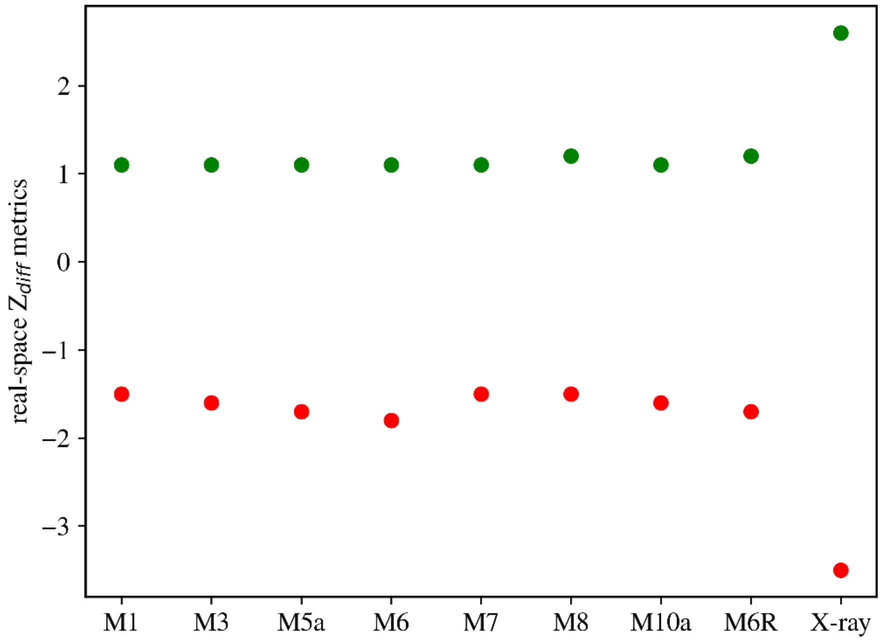

Supplementary Figure 252: Real-space Z-difference (RSZD) of inhibitor ZZ4. RSZD+ (green) and RSZD- (red) scores of inhibitor ZZ4 in HSP90 chaperone from various quantum refinement schemes (**M1-M10**). Those results for X-ray were taken from the experimental structure without our further refinement.

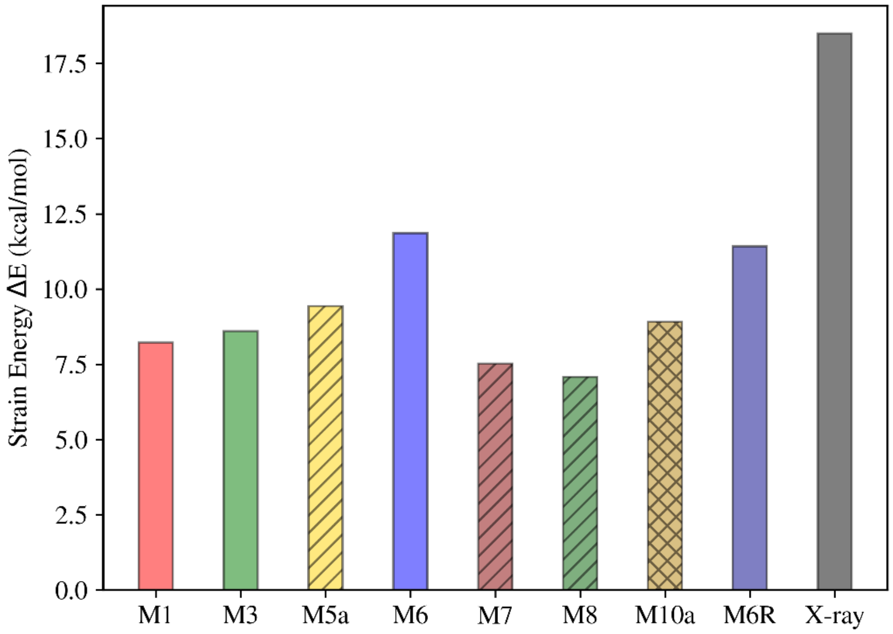

Supplementary Figure 253: Strain energy of inhibitor ZZ4. Strain energy (ΔE, kcal·mol^{-1}) at ωB97X-D/6-31G(d) level for inhibitor ZZ4 in HSP90 chaperone determined by various quantum refinement schemes (**M1-M10**). Those results for X-ray were taken from the experimental structure without our further refinement.

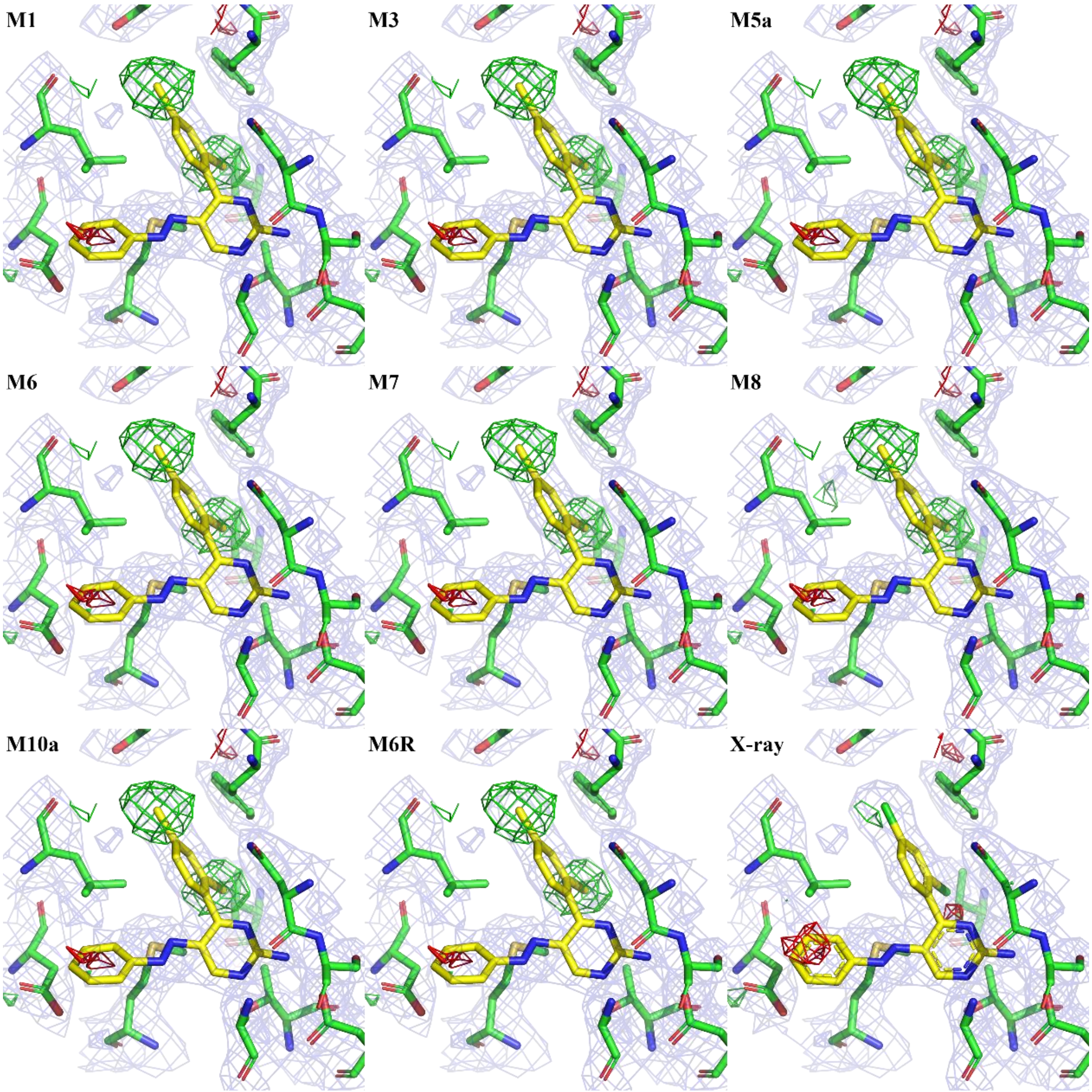

Supplementary Figure 254: Electron density maps of inhibitor ZZ4. Structures for inhibitor ZZ4 in HSP90 chaperone from various quantum refinement schemes (**M1-M10**), including the electron density maps (2mFo-DFc maps, contoured at 1.0 σ (blue), mFo-DFc maps, contoured at +3.0 σ (green), and mFo-DFc maps, contoured at -3.0 σ (red)). Those results for X-ray were taken from the experimental structure without our further refinement.

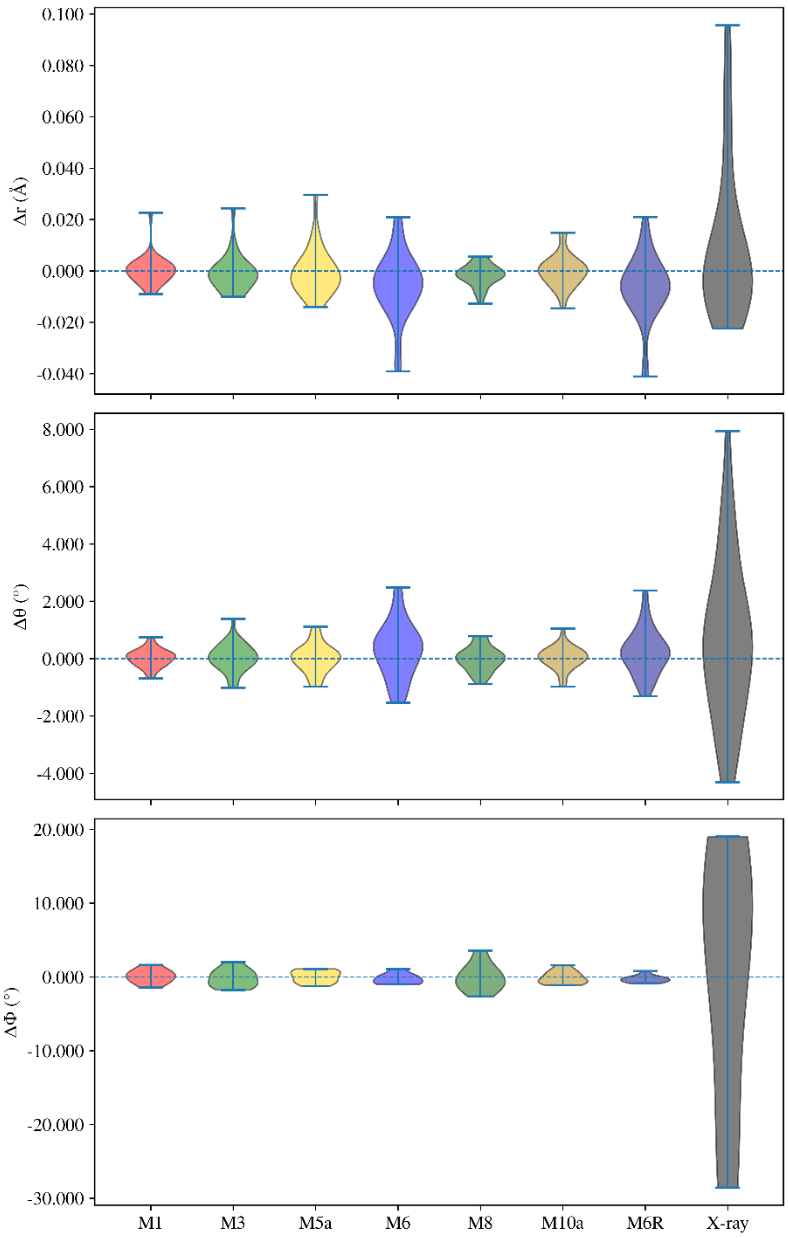

Supplementary Figure 255: Key coordinates of quantum refinement results of inhibitor ZZ4. Deviation in the refined bond distances (Δr, n = 25), angles (Δθ, n = 34) and dihedrals (Δϕ, n = 9) of inhibitor ZZ4 in HSP90 chaperone from various quantum refinement schemes (**M1-M10**) and X-ray structure which are compared to those obtained from the most reliable **M7** scheme. The solid line represents the upper and lower values.

(xlvi) 1UYG (Human HSP90-alpha with 8-(2,5-dimethoxy-benzyl)-2-fluoro-9H-purin-6-ylamine)

Protein preparations:

Resolution: 2.00 Å

Ligand: PU2; $C_{14}H_{14}FN_5O_2$

Residue flipped: GLN123, HIS154, GLN194

Protonation states (pH = 6.5):

HID77, HID154, HID189, HID210

Optimized region: PU2

High layer: PU2

Medium layer: ASN51, SER52, ALA55, ASP93, ILE96, GLY97, MET98, LEU103, LEU107, VAL136, PHE138, TYR139, VAL150, TRP162, THR184, WAT2058, WAT2059, WAT2062, WAT2121, WAT2123, WAT2131, WAT2152, WAT2155, WAT2156, WAT2235, WAT2236, WAT2237

ω_α = 1.3931

(a)

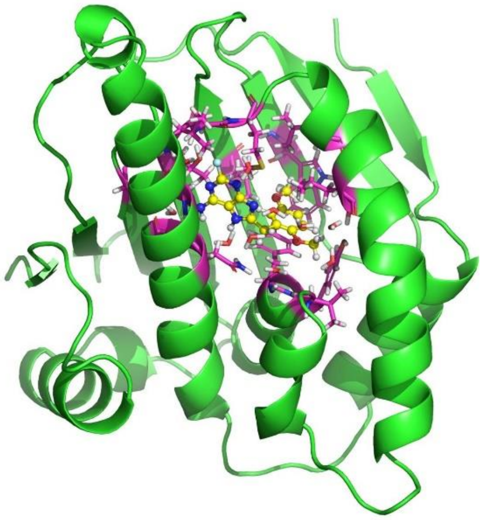

(b)

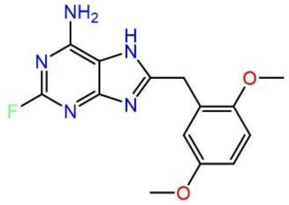

Supplementary Figure 256: Human HSP90-alpha with 8-(2,5-dimethoxy-benzyl)-2-fluoro-9H-purin-6-ylamine. (**a**) Crystal structure of human HSP90-alpha complexed with inhibitor PU2. ONIOM layers by different colors: yellow: high layer; red: medium layer; green: low layer. Inhibitor PU2 is presented in stick and balls. (**b**) Structure of inhibitor PU2.

Quantum refined structural results:

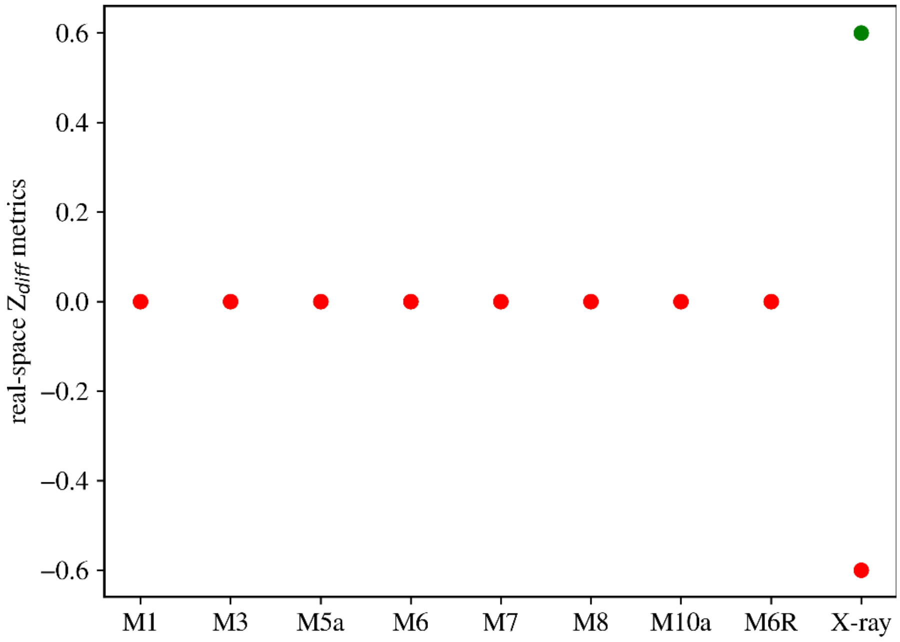

Supplementary Figure 257: Real-space Z-difference (RSZD) of inhibitor PU2. RSZD+ (green) and RSZD- (red) scores of inhibitor PU2 in human HSP90-alpha from various quantum refinement schemes (**M1-M10**). Those results for X-ray were taken from the experimental structure without our further refinement.

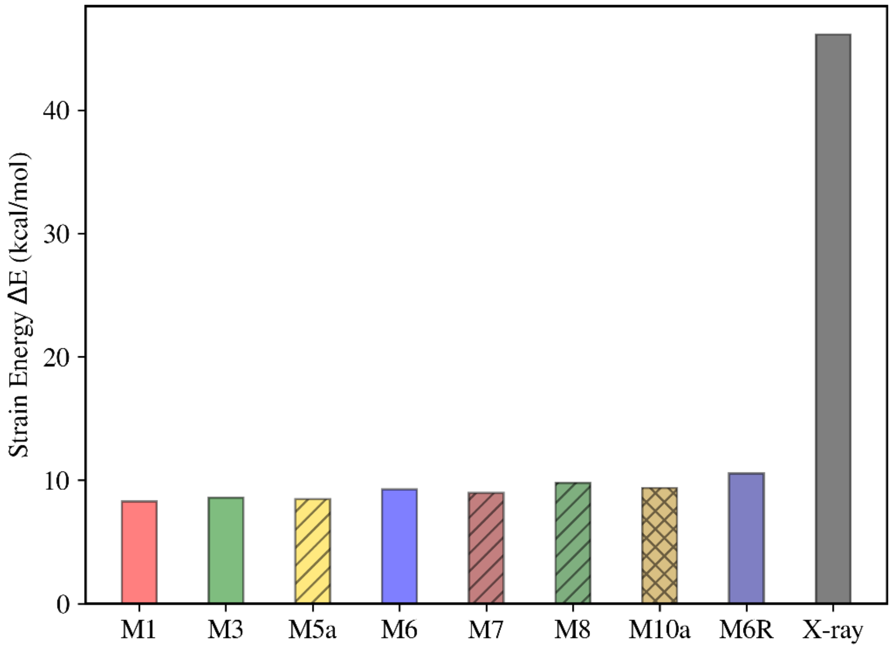

Supplementary Figure 258: Strain energy of inhibitor PU2. Strain energy (ΔE, kcal·mol^{-1}) at ωB97X-D/6-31G(d) level for inhibitor PU2 in human HSP90-alpha determined by various quantum refinement schemes (**M1-M10**). Those results for X-ray were taken from the experimental structure without our further refinement.

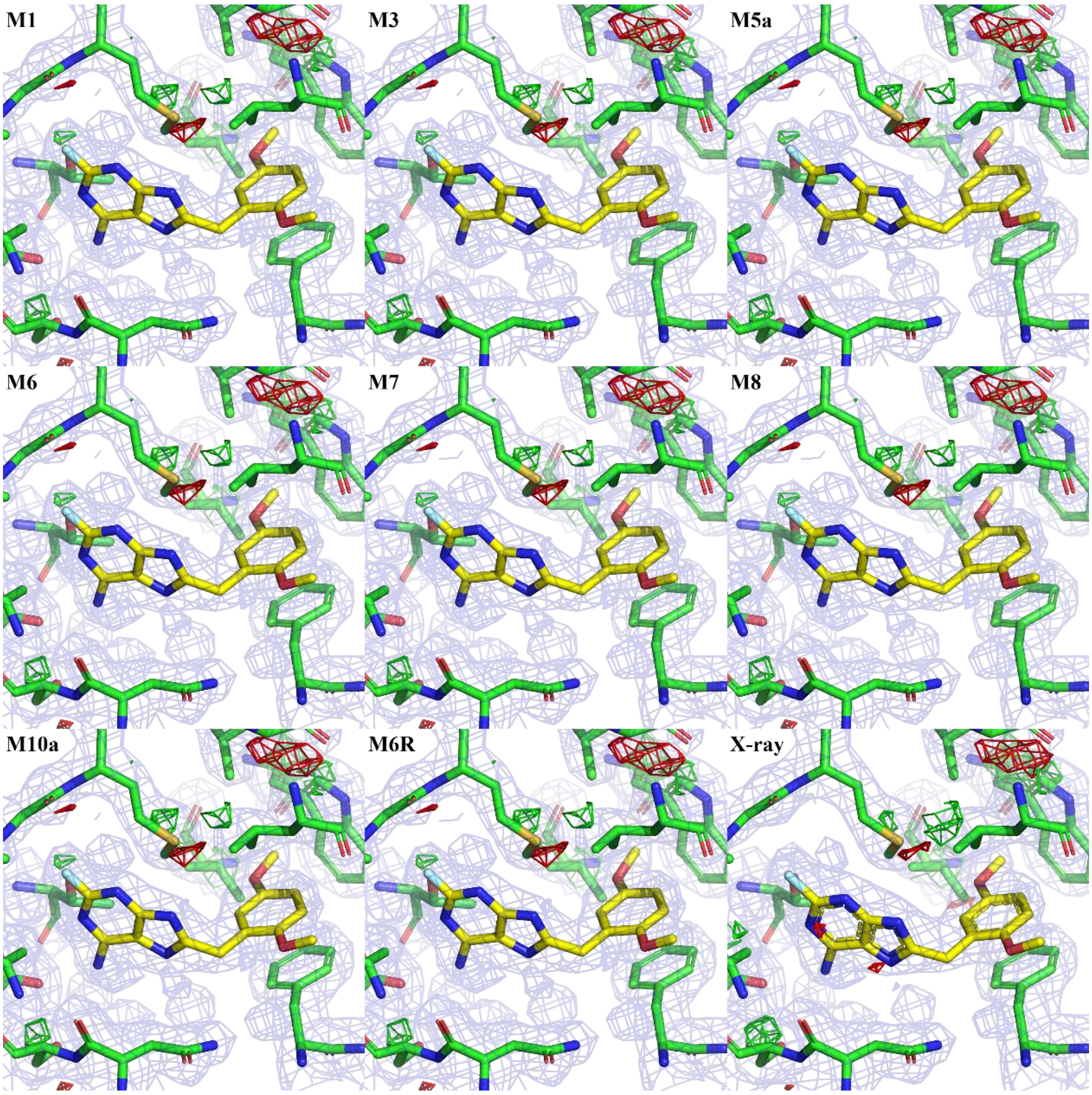

Supplementary Figure 259: Electron density maps of inhibitor PU2. Structures for inhibitor PU2 in human HSP90-alpha from various quantum refinement schemes (**M1-M10**), including the electron density maps (2mFo-DFc maps, contoured at 1.0 σ (blue), mFo-DFc maps, contoured at +3.0 σ (green), and mFo-DFc maps, contoured at -3.0 σ (red)). Those results for X-ray were taken from the experimental structure without our further refinement.

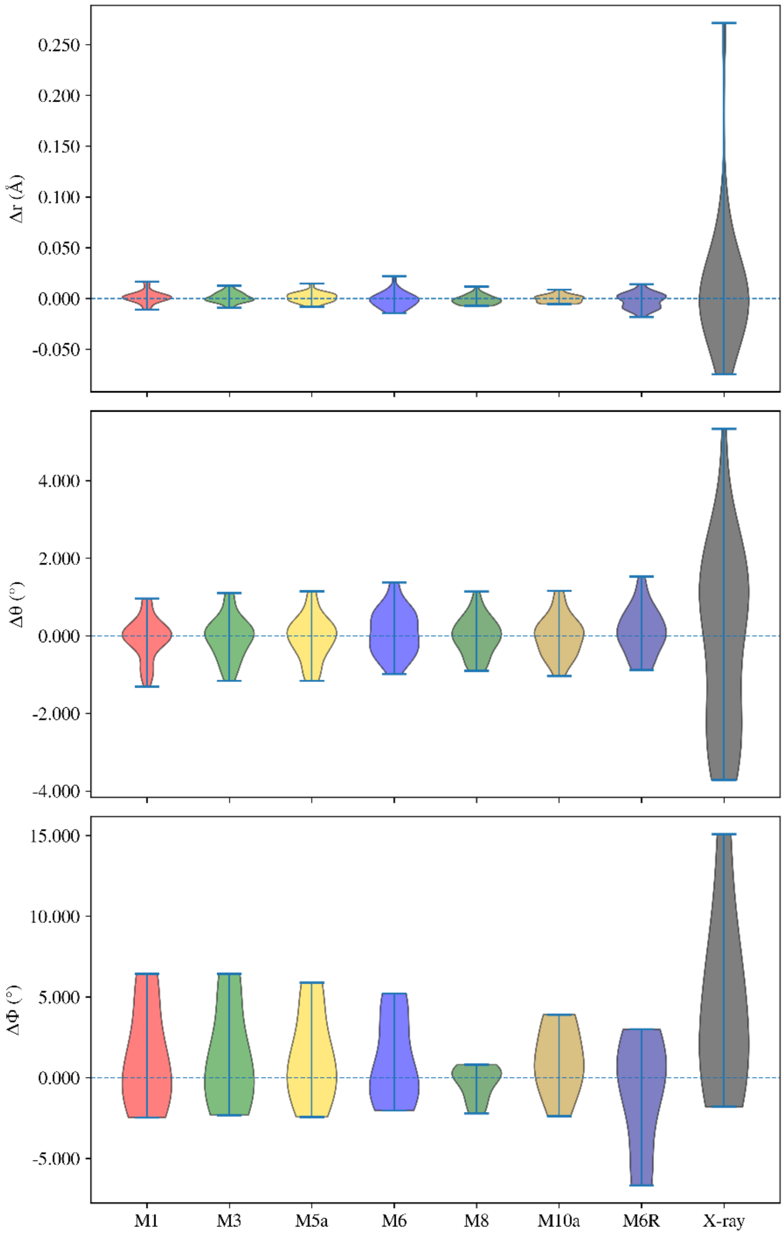

Supplementary Figure 260: Key coordinates of quantum refinement results of inhibitor PU2. Deviation in the refined bond distances (Δr, n = 24), angles (Δθ, n = 34) and dihedrals (Δϕ, n = 8) of inhibitor PU2 in human HSP90-alpha from various quantum refinement schemes (**M1-M10**) and X-ray structure which are compared to those obtained from the most reliable **M7** scheme. The solid line represents the upper and lower values.

(xlvii) 2WI1 (Orally Active 2-Amino Thienopyrimidine Inhibitors of the HSP90 Chaperone)

Protein preparations:

Resolution: 2.30 Å

Ligand: ZZ2; $C_8H_{13}N_3O_2$

Residue flipped: GLN23, ASN105

Protonation states (pH = 6.5):

HID77, HID154, HID189, HID210

Optimized region: ZZ2

High layer: ZZ2

Medium layer: ASN51, SER52, ASP54, ALA55, ASP93, ILE96, MET98, LEU107, PHE138, THR184, WAT2032, WAT2033, WAT2060, WAT2094, WAT2123

ω_α = 1.4337

(a)

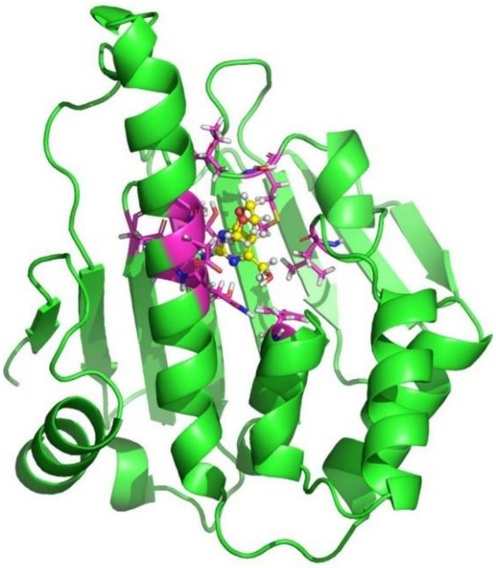

(b)

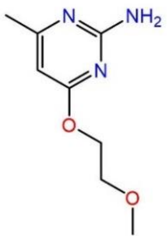

Supplementary Figure 261: 2-Amino Thienopyrimidine Inhibitors of the HSP90 Chaperone. (**a**) Crystal structure of HSP90 chaperone complexed with inhibitor ZZ2. ONIOM layers by different colors: yellow: high layer; red: medium layer; green: low layer. Inhibitor ZZ2 is presented in stick and balls. (**b**) Structure of inhibitor ZZ2.

Quantum refined structural results:

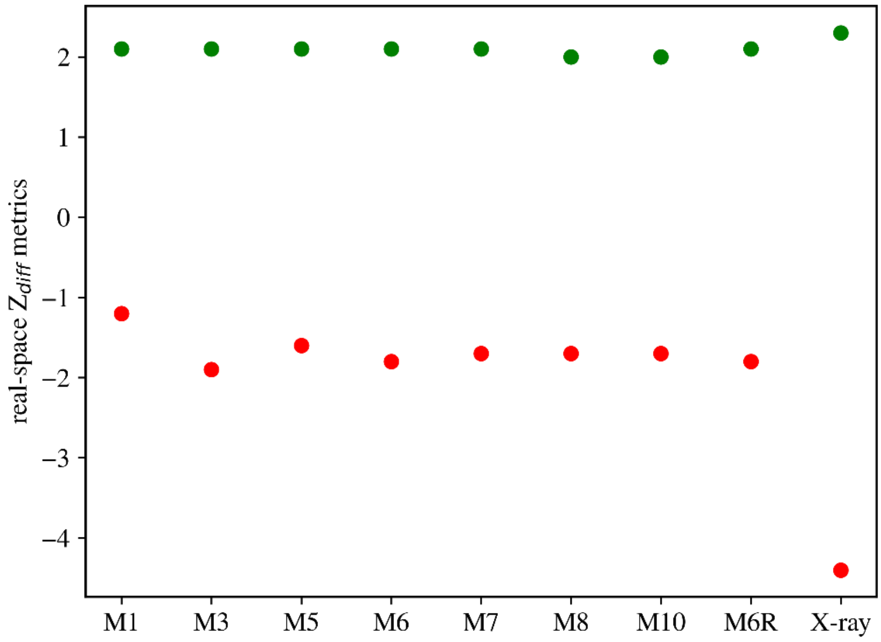

Supplementary Figure 262: Real-space Z-difference (RSZD) of inhibitor ZZ2. RSZD+ (green) and RSZD- (red) scores of inhibitor ZZ2 in HSP90 chaperone from various quantum refinement schemes (**M1-M10**). Those results for X-ray were taken from the experimental structure without our further refinement.

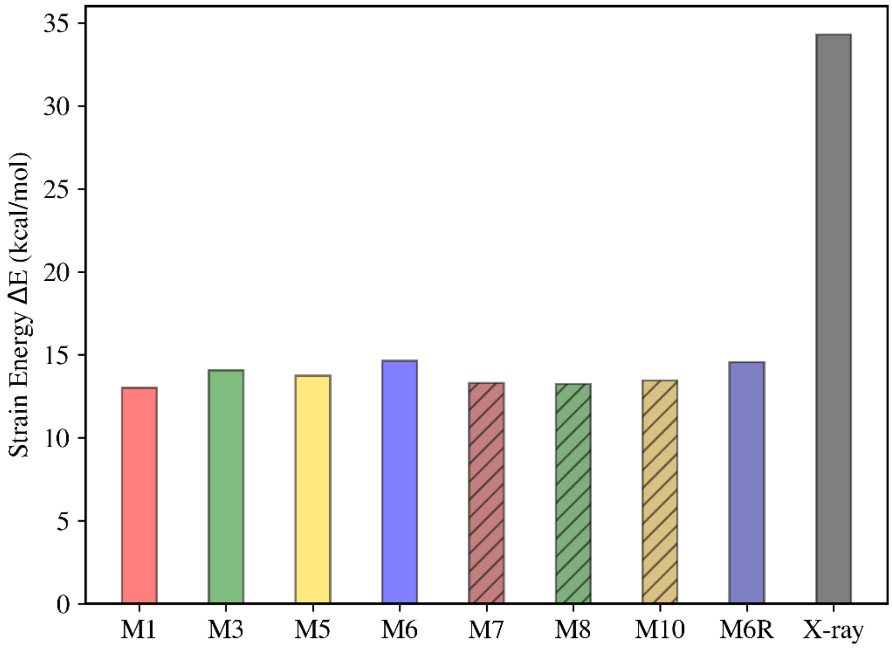

Supplementary Figure 263: Strain energy of inhibitor ZZ2. Strain energy (ΔE, kcal·mol^{-1}) at ωB97X-D/6-31G(d) level for inhibitor ZZ2 in HSP90 chaperone determined by various quantum refinement schemes (**M1-M10**). Those results for X-ray were taken from the experimental structure without our further refinement.

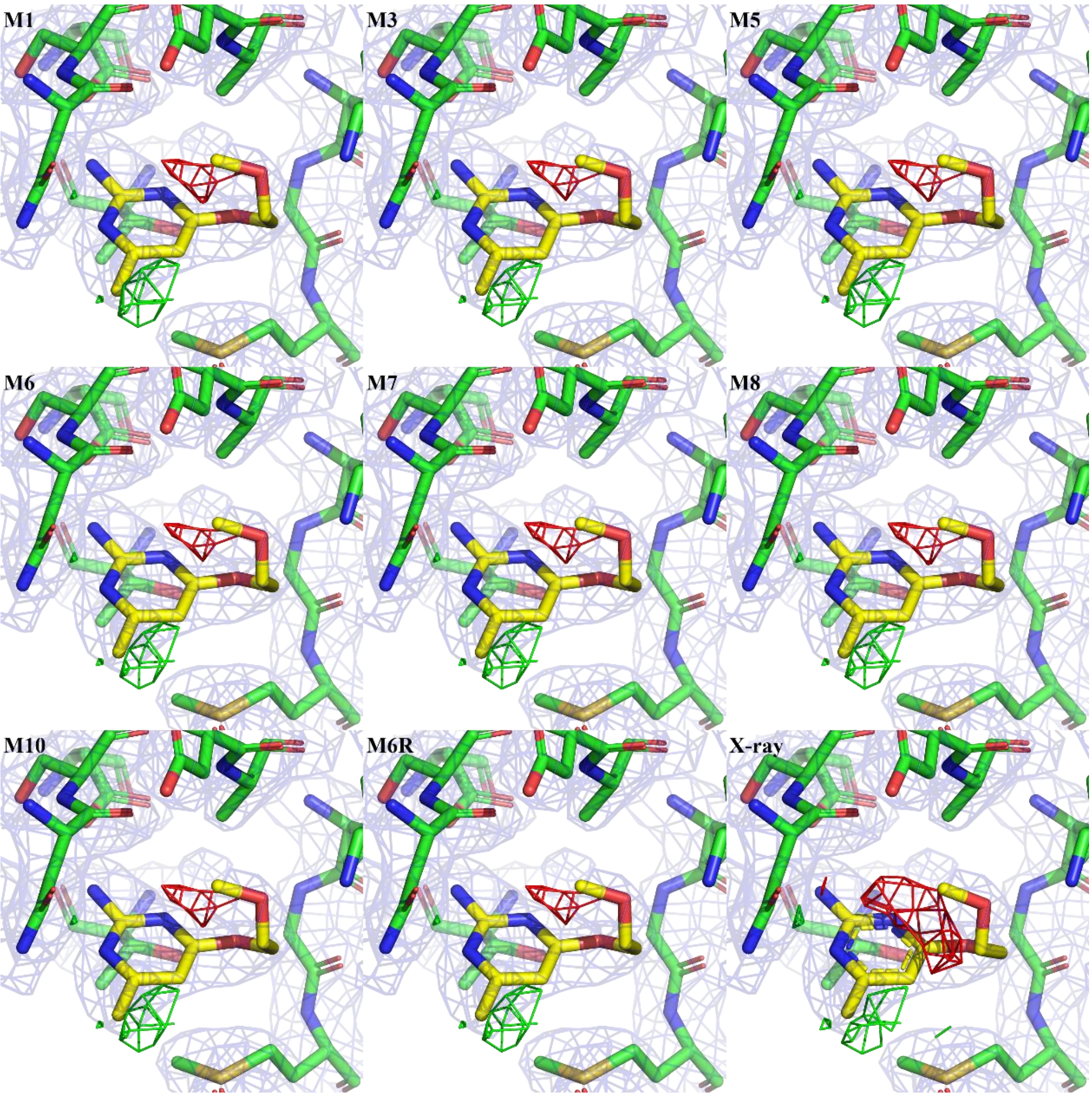

Supplementary Figure 264: Electron density maps of inhibitor ZZ2. Structures for inhibitor ZZ2 in HSP90 chaperone from various quantum refinement schemes (**M1-M10**), including the electron density maps (2mFo-DFc maps, contoured at 1.0 σ (blue), mFo-DFc maps, contoured at +3.0 σ (green), and mFo-DFc maps, contoured at -3.0 σ (red)). Those results for X-ray were taken from the experimental structure without our further refinement.

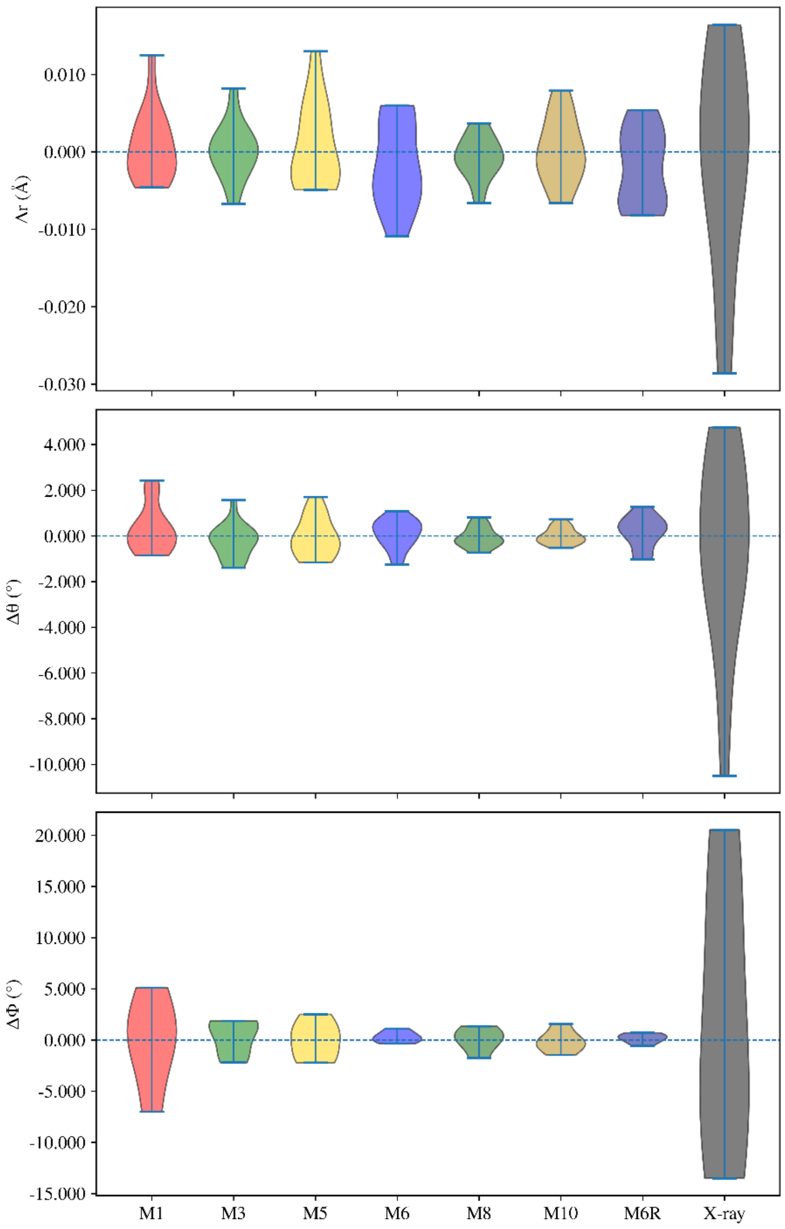

Supplementary Figure 265: Key coordinates of quantum refinement results of inhibitor ZZ2. Deviation in the refined bond distances (Δr, n = 13), angles (Δθ, n = 16) and dihedrals (Δϕ, n = 5) of inhibitor ZZ2 in HSP90 chaperone from various quantum refinement schemes (**M1-M10**) and X-ray structure which are compared to those obtained from the most reliable **M7** scheme. The solid line represents the upper and lower values.

(xlviii) **3FR2 (N-Benzyl-indolo carboxylic acids: Design and synthesis of potent and selective adipocyte Fatty-Acid Binding Protein (A-FABP) inhibitors)**

Protein preparations:

Resolution: 2.20 Å

Ligand: 8CA; $C_{20}H_{19}NO_2$

Residue flipped: No

Protonation states (pH = 7.0):

HID93

Optimized region: 8CA

High layer: 8CA

Medium layer: PHE16, TYR19, MET20, ALA33, PRO38, SER55, PHE57, LYS58, THR60, ALA75, ASP76, ARG78, ILE104, VAL115, ARG126, TYR128, WAT244, WAT255, WAT263, WAT264, WAT273, WAT277, WAT280

ω_α = 1.2647

(a)

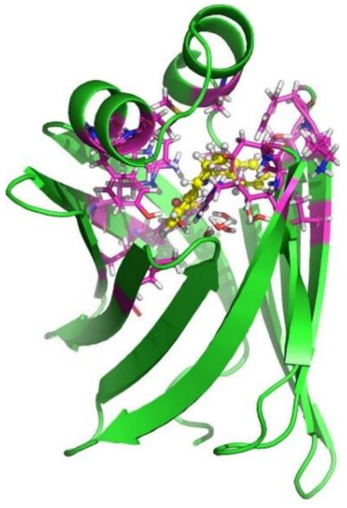

(b)

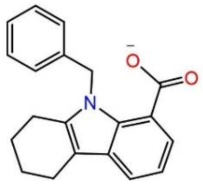

Supplementary Figure 266: N-Benzyl-indolo carboxylic acids in adipocyte Fatty-Acid Binding Protein (A-FABP). (**a**) Crystal structure of A-FABP complexed with inhibitor 8CA. ONIOM layers by different colors: yellow: high layer; red: medium layer; green: low layer. Inhibitor 8CA is presented in stick and balls. (**b**) Structure of inhibitor 8CA.

Quantum refined structural results:

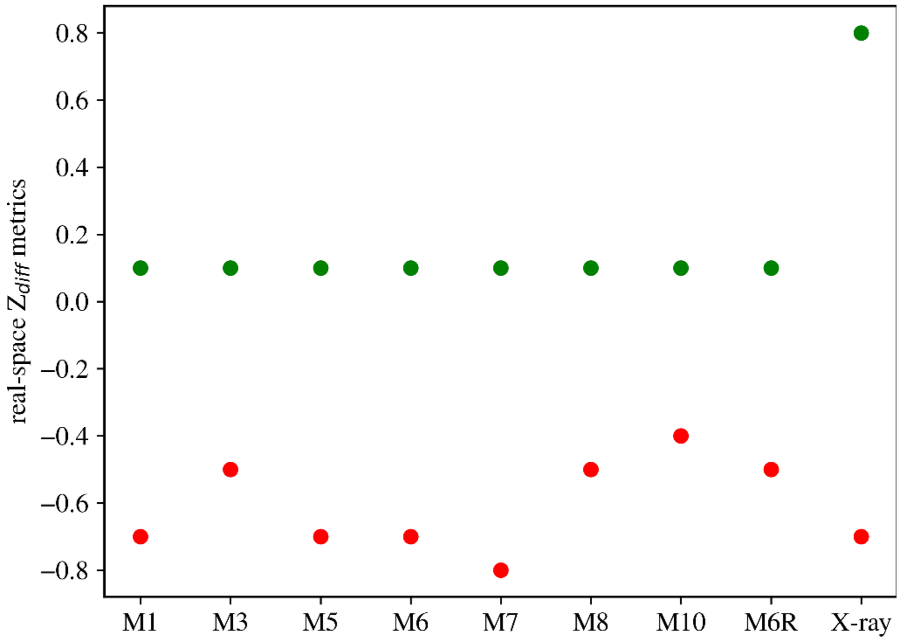

Supplementary Figure 267: Real-space Z-difference (RSZD) of inhibitor 8CA. RSZD+ (green) and RSZD- (red) scores of inhibitor 8CA in A-FABP from various quantum refinement schemes (**M1-M10**). Those results for X-ray were taken from the experimental structure without our further refinement.

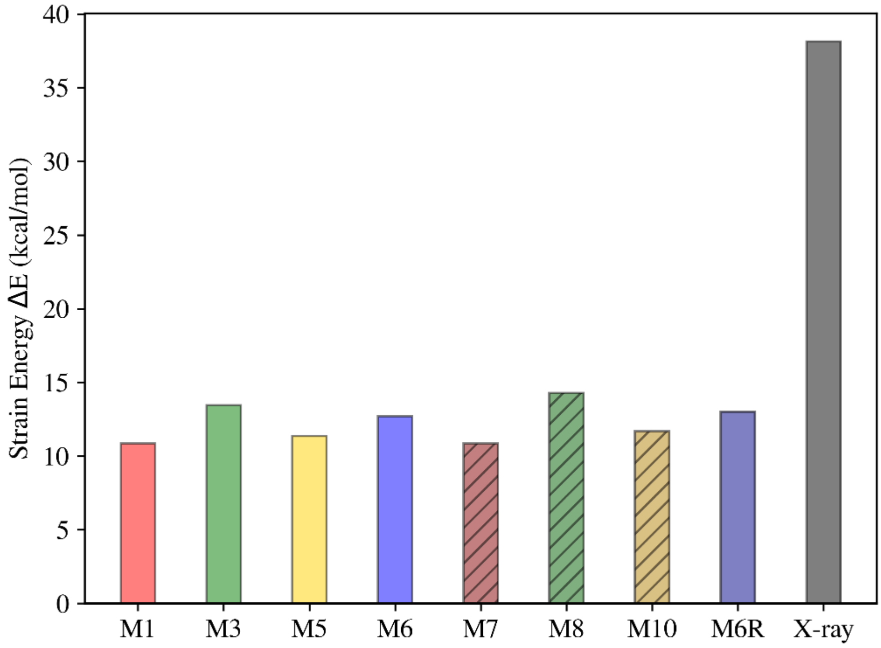

Supplementary Figure 268: Strain energy of inhibitor 8CA. Strain energy (ΔE, kcal·mol^{-1}) at ωB97X-D/6-31G(d) level for inhibitor 8CA in A-FABP determined by various quantum refinement schemes (**M1-M10**). Those results for X-ray were taken from the experimental structure without our further refinement.

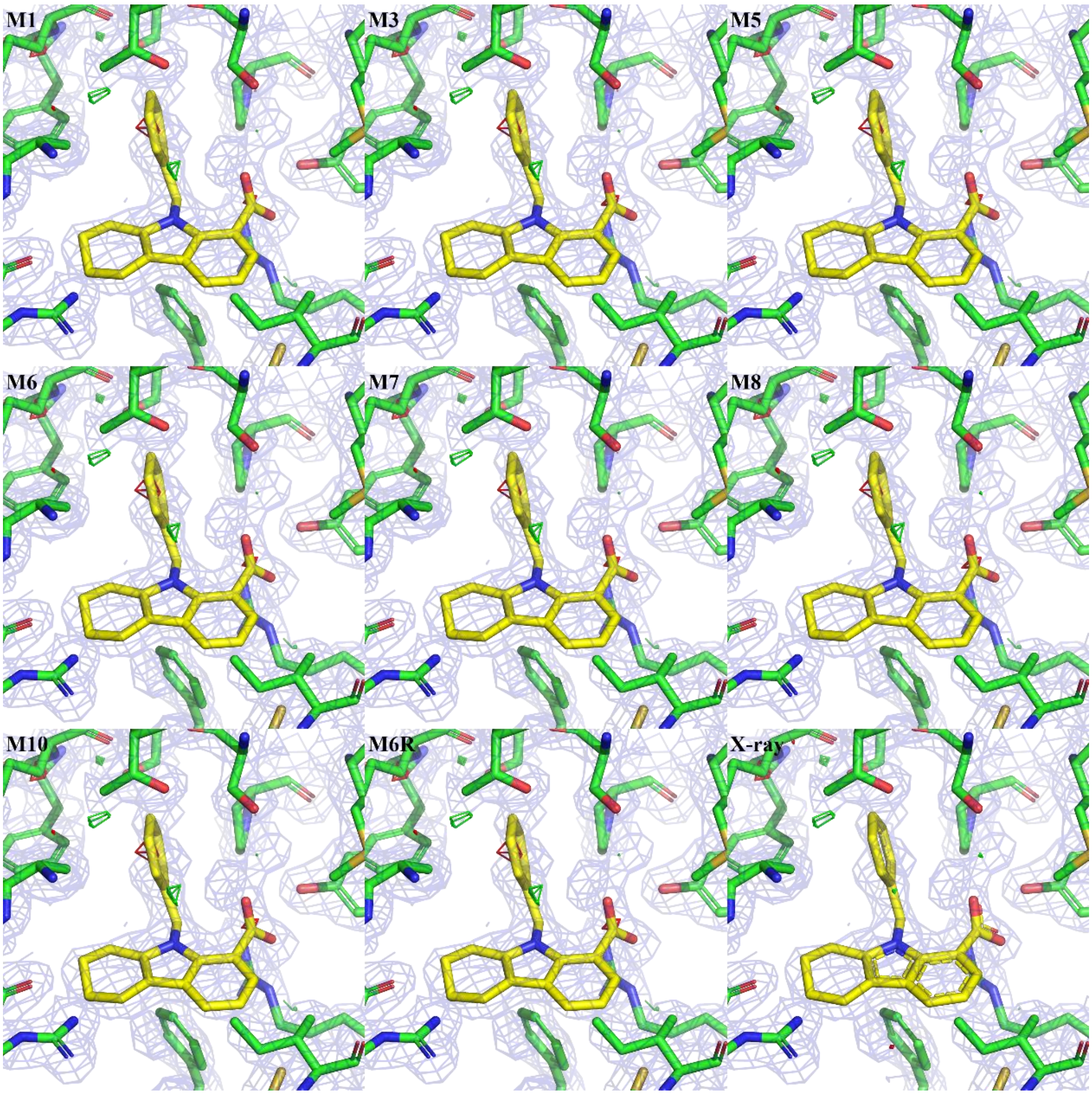

Supplementary Figure 269: Electron density maps of inhibitor 8CA. Structures for inhibitor 8CA in A-FABP from various quantum refinement schemes (**M1**-**M10**), including the electron density maps (2mFo-DFc maps, contoured at 1.0 σ (blue), mFo-DFc maps, contoured at +3.0 σ (green), and mFo-DFc maps, contoured at -3.0 σ (red)). Those results for X-ray were taken from the experimental structure without our further refinement.

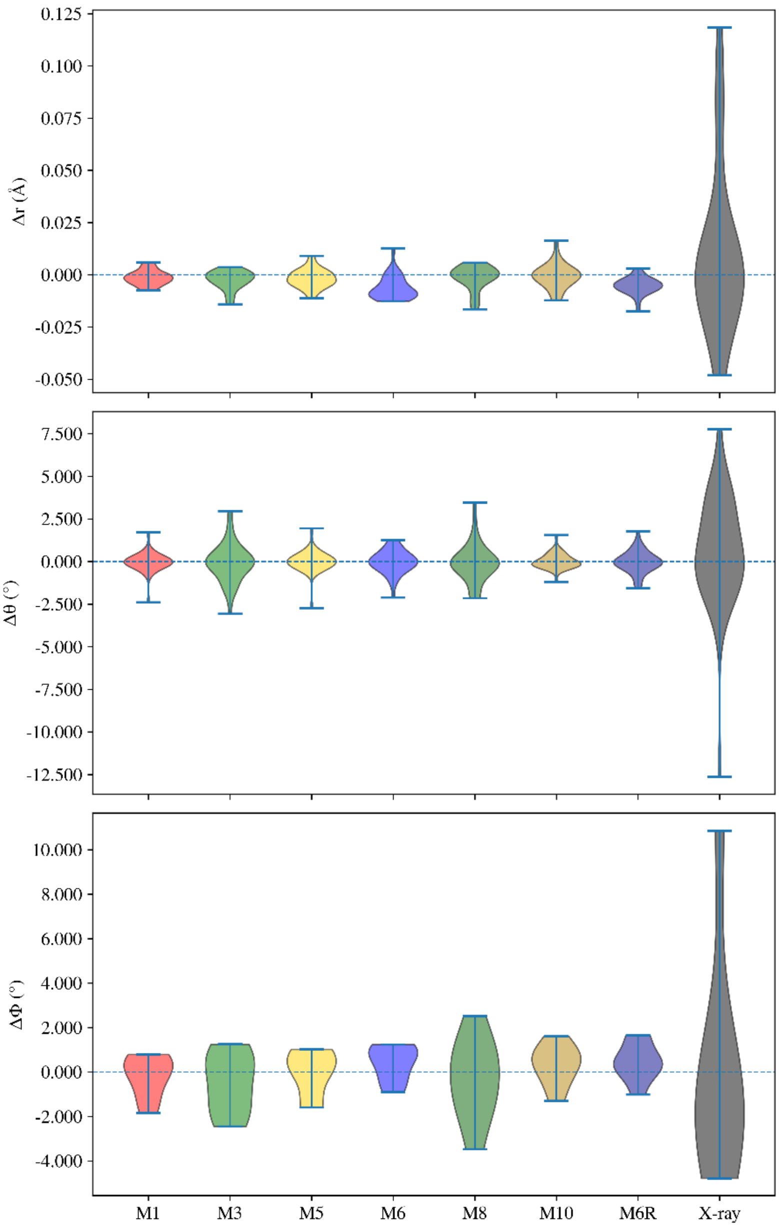

Supplementary Figure 270: Key coordinates of quantum refinement results of inhibitor 8CA. Deviation in the refined bond distances (Δr, n = 26), angles (Δθ, n = 37) and dihedrals (Δϕ, n = 8) of inhibitor 8CA in A-FABP from various quantum refinement schemes (**M1-M10**) and X-ray structure which are compared to those obtained from the most reliable **M7** scheme. The solid line represents the upper and lower values.

(xlix) **3ATM (trypsin complexed with 2-(1H-indol-3-yl)ethanamine)**

Protein preparations:

Resolution: 1.72 Å

Ligand: TSS (Tryptamine); $C_{10}H_{12}N_2$

Residue flipped: GLN51, HIS58, ASN74, ASN97, ASN101, GLN173, GLN217, ASN229

Protonation states (pH = 8.5):

HID41, HID58, HID91

CYX25-CYX155, CYX43-CYX59, CYX127-CYX228, CYX134-CYX201, CYX166-CYX180, CYX191-CYX215

Optimized region: TSS

High layer: TSS

Medium layer: ASP189, SER190, CYX191, GLN192, SER195, VAL209, TRP211, GLY212, GLY214, CYX215, GLY222, WAT331, WAT332, WAT365, WAT402, WAT450, WAT507

ω_α = 0.28433

(a)

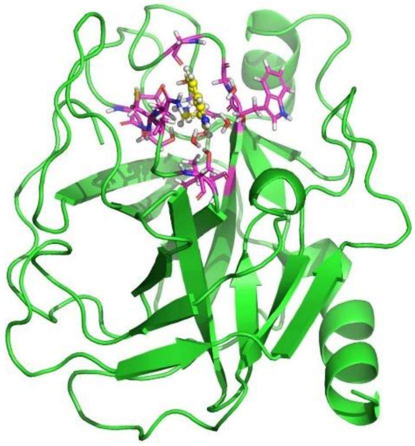

(b)

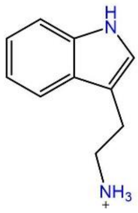

Supplementary Figure 271: Trypsin complexed with 2-(1H-indol-3-yl)ethanamine. (**a**) Crystal structure of trypsin complexed with tryptamine. ONIOM layers by different colors: yellow: high layer; red: medium layer; green: low layer. Ligand tryptamine is presented in stick and balls. (**b**) Structure of tryptamine.

Quantum refined structural results:

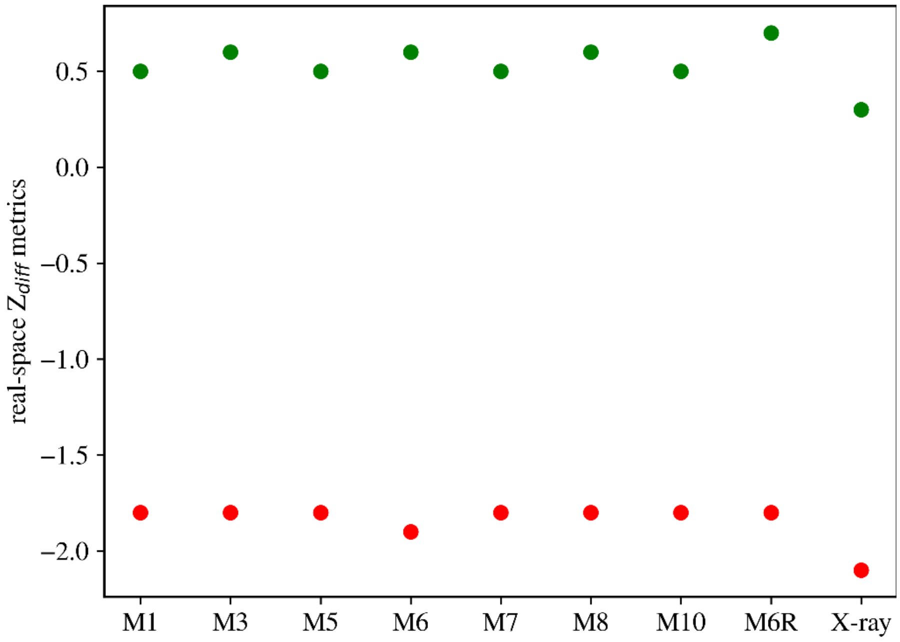

Supplementary Figure 272: Real-space Z-difference (RSZD) of tryptamine. RSZD+ (green) and RSZD- (red) scores of tryptamine in trypsin from various quantum refinement schemes (**M1-M10**). Those results for X-ray were taken from the experimental structure without our further refinement.

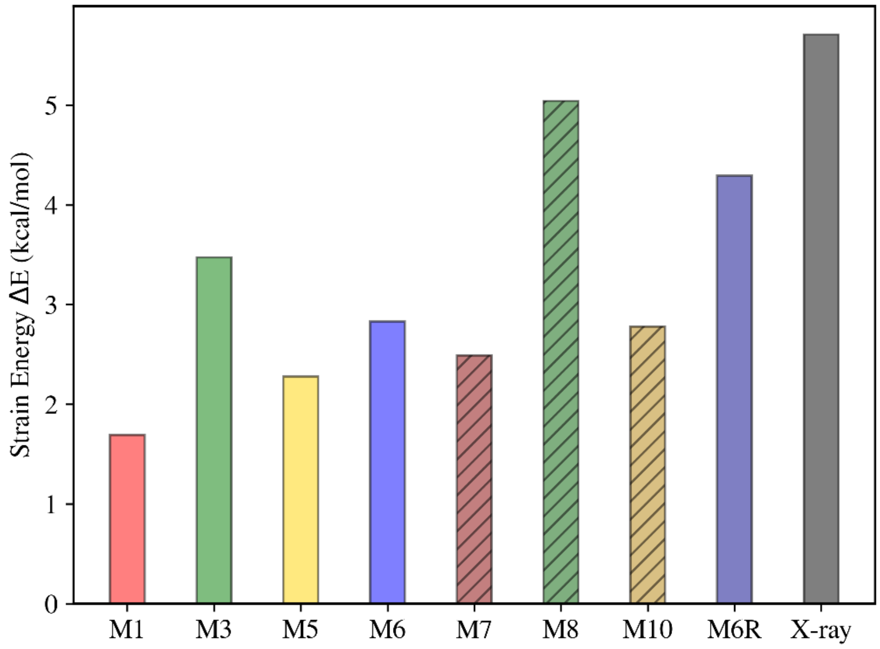

Supplementary Figure 273: Strain energy of tryptamine. Strain energy (ΔE, kcal·mol^{-1}) at ωB97X-D/6-31G(d) level for tryptamine in trypsin determined by various quantum refinement schemes (**M1-M10**). Those results for X-ray were taken from the experimental structure without our further refinement.

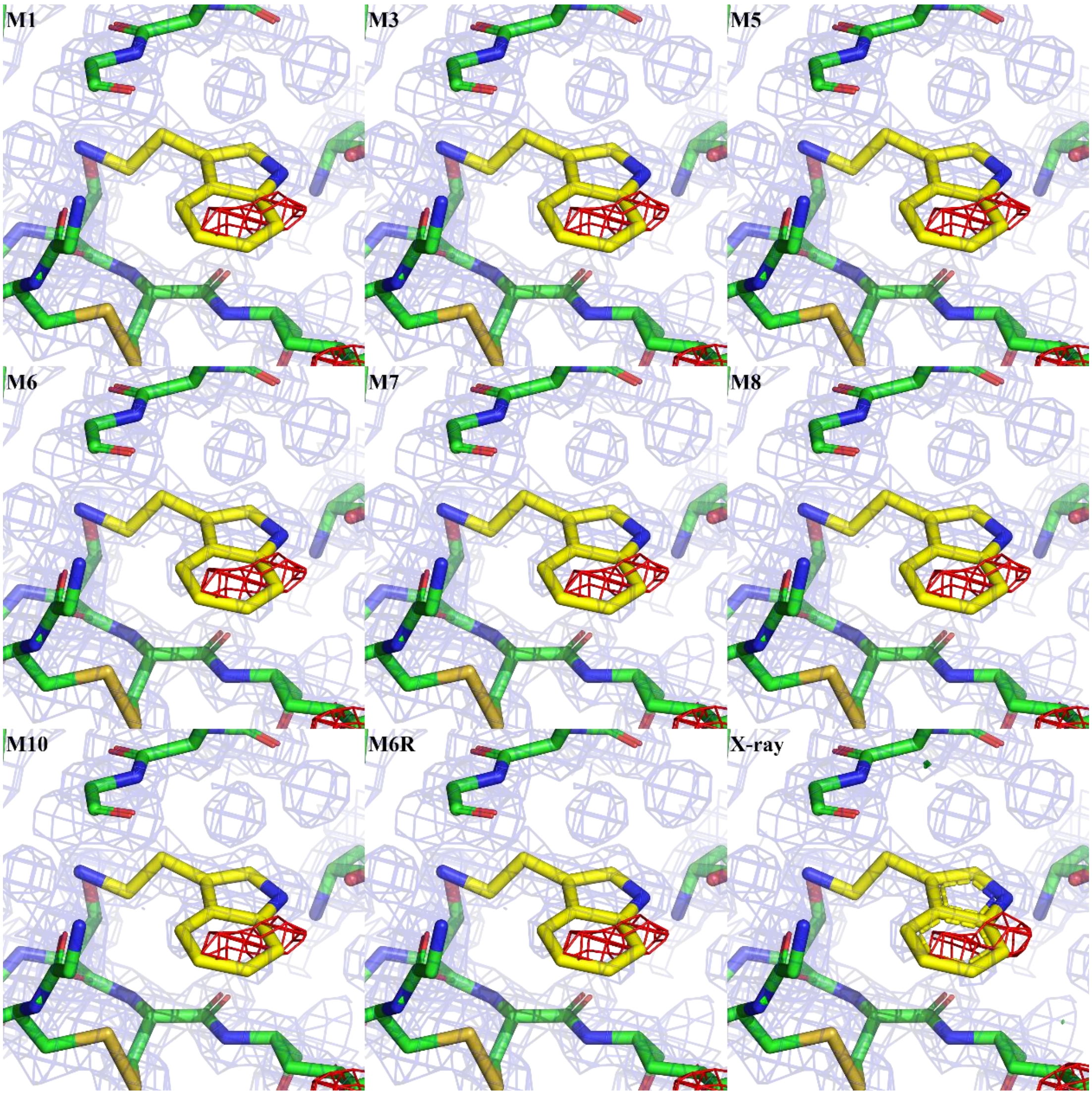

Supplementary Figure 274: Electron density maps of tryptamine. Structures for tryptamine in trypsin various quantum refinement schemes (**M1-M10**), including the electron density maps (2mFo-DFc maps, contoured at 1.0 σ (blue), mFo-DFc maps, contoured at +3.0 σ (green), and mFo-DFc maps, contoured at -3.0 σ (red)). Those results for X-ray were taken from the experimental structure without our further refinement.

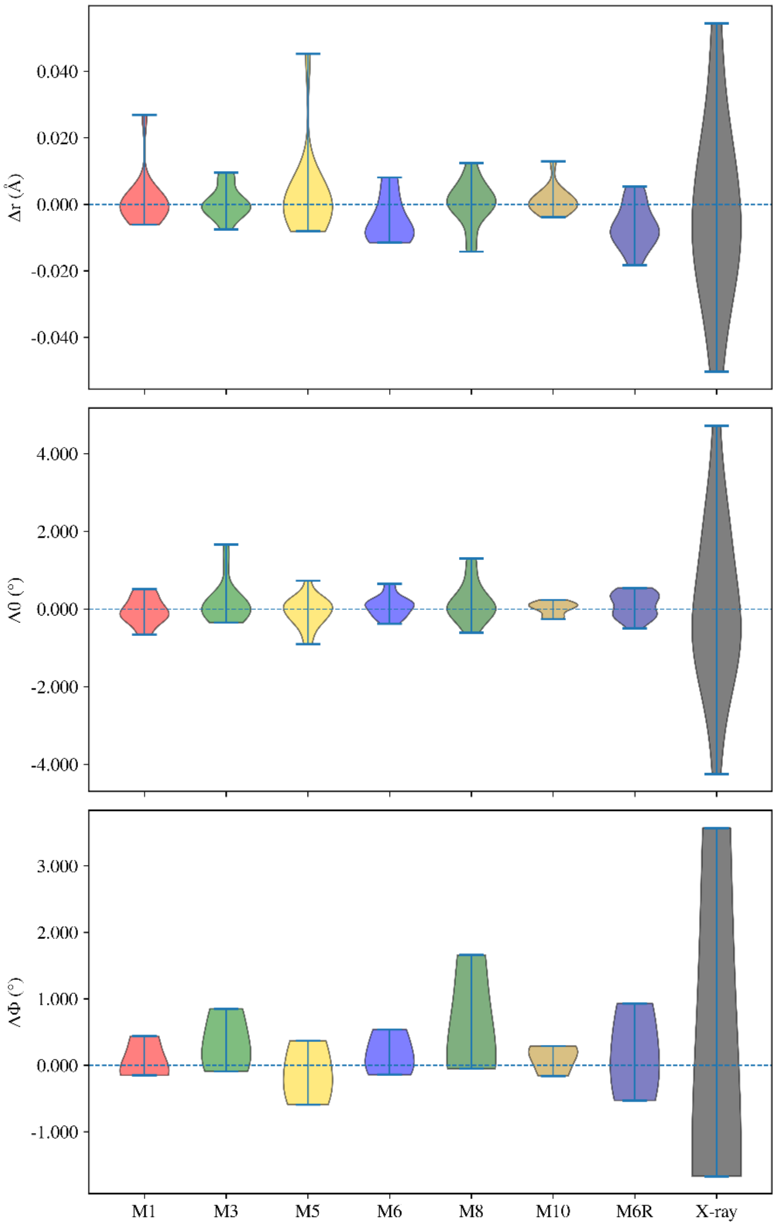

Supplementary Figure 275: Key coordinates of quantum refinement results of tryptamine. Deviation in the refined bond distances (Δr, n = 13), angles (Δθ, n = 17) and dihedrals (Δϕ, n = 3) of tryptamine in trypsin from various quantum refinement schemes (**M1-M10**) and X-ray structure which are compared to those obtained from the most reliable **M7** scheme. The solid line represents the upper and lower values.

(l) 5L7I (Structure of human Smoothened in complex with Vismodegib)

Protein preparations:

Resolution: 3.30 Å

Ligand: VIS(Vismodegib); $C_{19}H_{14}Cl_2N_2O_3S$

Residue flipped:

Chain A: ASN136, ASN1021, ASN476, GLN477 ASN521,

Chain B: HIS103, GLN123, ASN136, GLN380, GLN1040, ASN1114, HIS470, GLN477, ASN521

Protonation states (pH = 4.0):

Chain A: HIP63, GLH71, ASH97, GLH101, HIP103, GLH135, GLH140, GLH158, GLH160, GLH176, GLH194, GLH208, GLH211, HIP227, HIP231, ASH255, GLH292, GLH305, GLH310, HIP340, HIP361, ASH382, ASH384, ASH1017, GLH1019, ASH1020, GLH1023, GLH1033, HIP1078, ASH1089, GLH1096, GLH1101, GLH1107, GLH1122, GLH447, HIE470, ASH473, GLH481, GLH508, GLH518

Chain A: HIP63, GLH71, GLH101, HIP103, GLH135, GLH140, GLH158, GLH160, GLH176, GLH194, GLH208, ASH209, GLH211, GLH224, HIP227, HIP231, GLH292, GLH305, GLH310, HIP340, HIP361, ASH382, ASH384, GLH1019, ASH1020, GLH1023, ASH1054, HIP1078, ASH1089, GLH1101, GLH1107, GLH1122, GLH447, HID470, ASH473, GLH481, GLH508, GLH518

Optimized region: Chain A: VIS and Chain B VIS

High layer: Chain A: VIS and Chain B VIS

Medium layer:

Chain A: ASN219, LEU221, MET230, TRP281, ASH384, VAL386, SER387, ILE389, TYR394, ARG400, GLN477, TRP480, PHE484, PRO513, GLH518, ASN521, LEU522, MET525

Chain B: ASN219, LEU221, MET230, TRP281, ASH384, VAL386, SER387, ILE389, TYR394, ARG400, ASH473, GLN477, TRP480, PHE484, PRO513, GLH518, ASN521, LEU522, MET525

ω_α = 9.9079

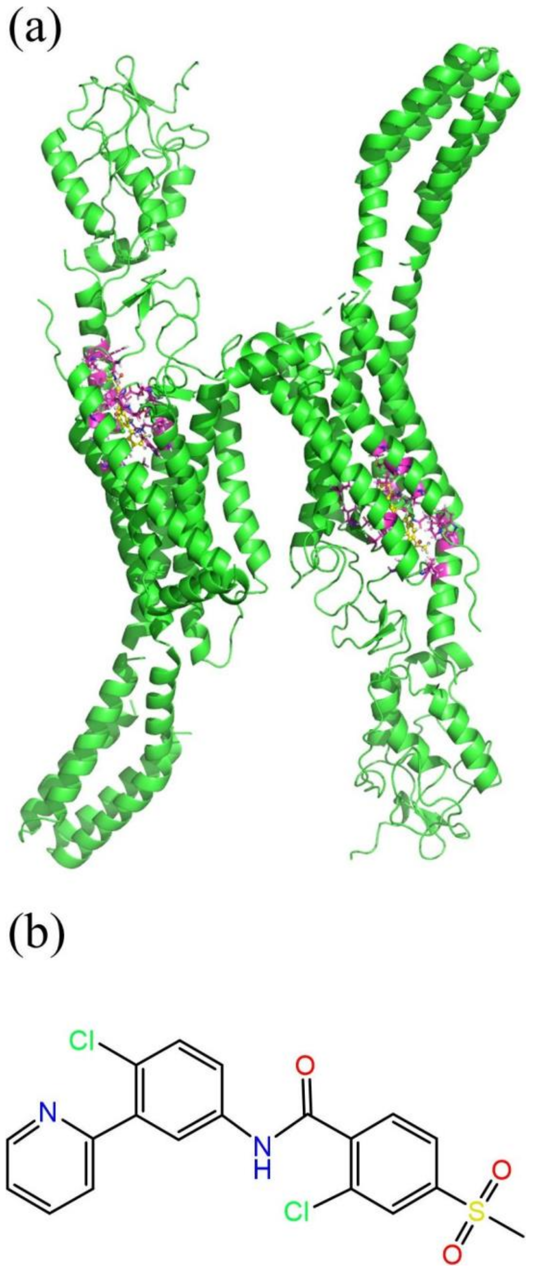

Supplementary Figure 276: Human Smoothened complexed with Vismodegib. (**a**) Crystal structure of human smoothened complexed with vismodegib. ONIOM layers by different colors: yellow: high layer; red: medium layer; green: low layer. Ligand vismodegib is presented in stick and balls. (**b**) Structure of vismodegib.

Quantum refined structural results:

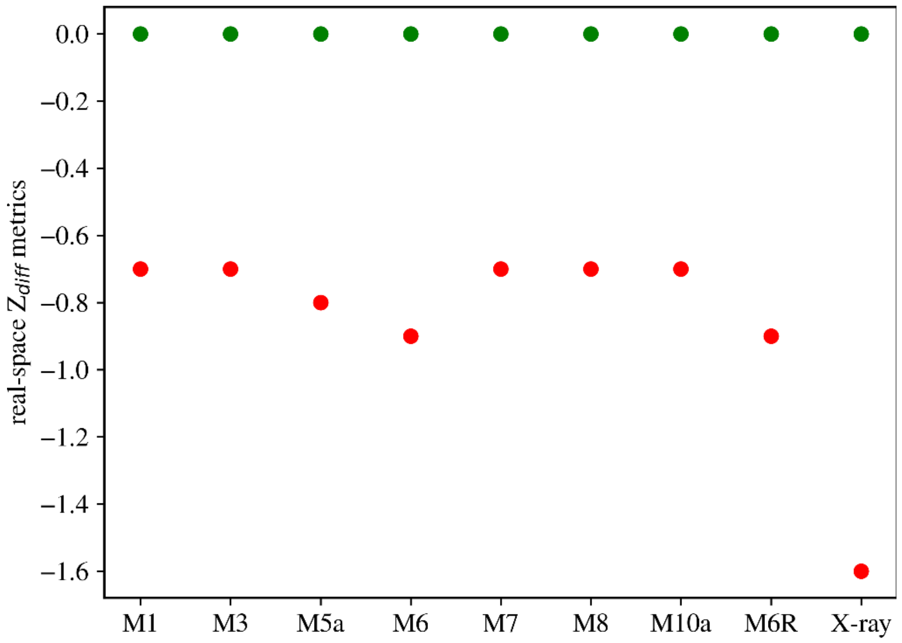

Supplementary Figure 277: Real-space Z-difference (RSZD) of vismodegib (Chain A). RSZD+ (green) and RSZD- (red) scores of vismodegib in human smoothened (Chain A) from various quantum refinement schemes (**M1-M10**). Those results for X-ray were taken from the experimental structure without our further refinement.

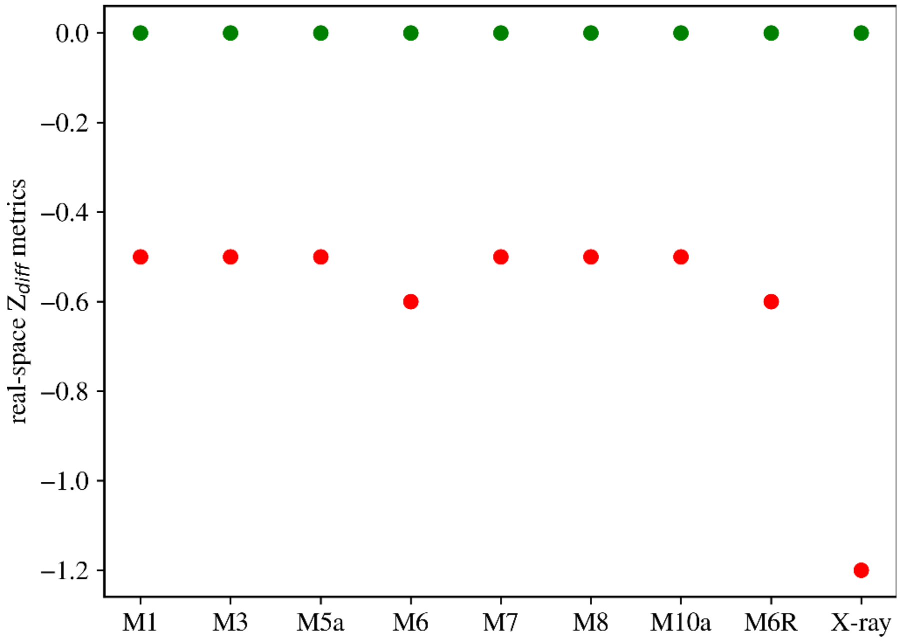

Supplementary Figure 278: Real-space Z-difference (RSZD) of vismodegib (Chain B). RSZD+ (green) and RSZD- (red) scores of vismodegib in human smoothened (Chain B) from various quantum refinement schemes (**M1-M10**). Those results for X-ray were taken from the experimental structure without our further refinement.

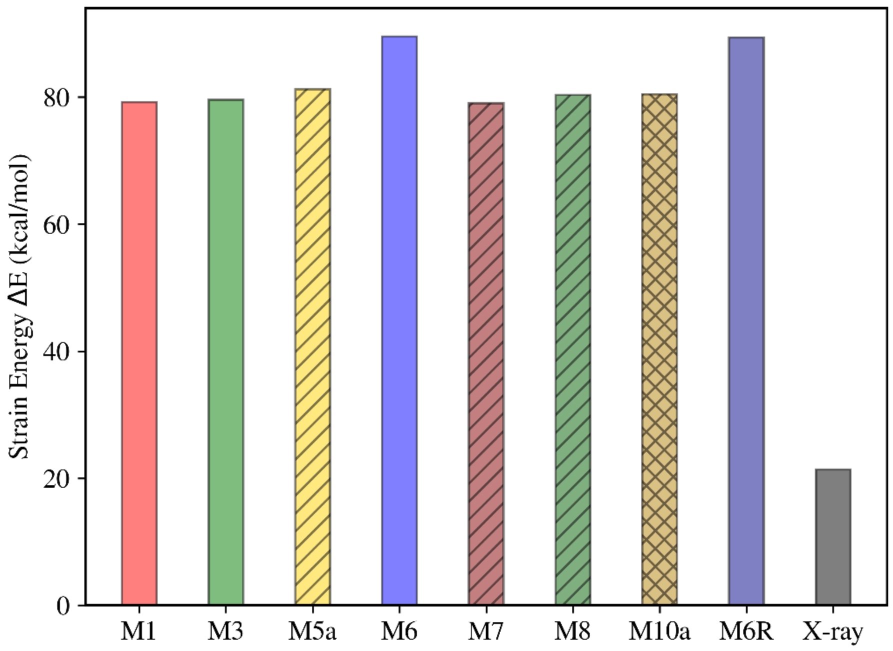

Supplementary Figure 279: Strain energy of vismodegib. Strain energy (ΔE, kcal·mol^{-1}) at ωB97X-D/6-31G(d) level for vismodegib in human smoothened (Chain A and Chain B) determined by various quantum refinement schemes (**M1**-**M10**). Those results for X-ray were taken from the experimental structure without our further refinement.

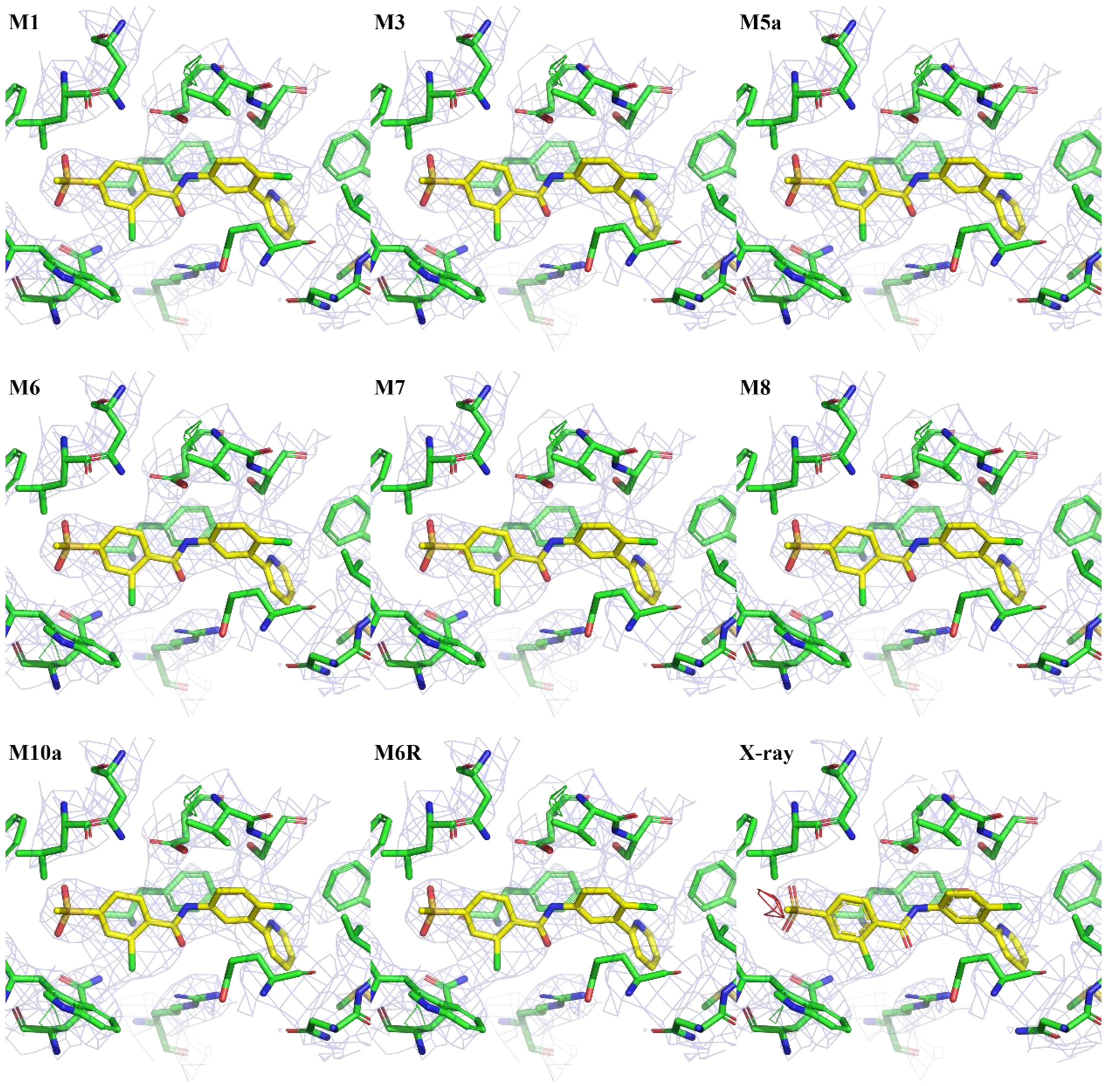

Supplementary Figure 280: Electron density maps of vismodegib (chain A). Structures for vismodegib in human smoothened (chain A) from various quantum refinement schemes (**M1**-**M10**), including the electron density maps (2mFo-DFc maps, contoured at 1.0 σ (blue), mFo-DFc maps, contoured at +3.0 σ (green), and mFo-DFc maps, contoured at -3.0 σ (red)). Those results for X-ray were taken from the experimental structure without our further refinement.

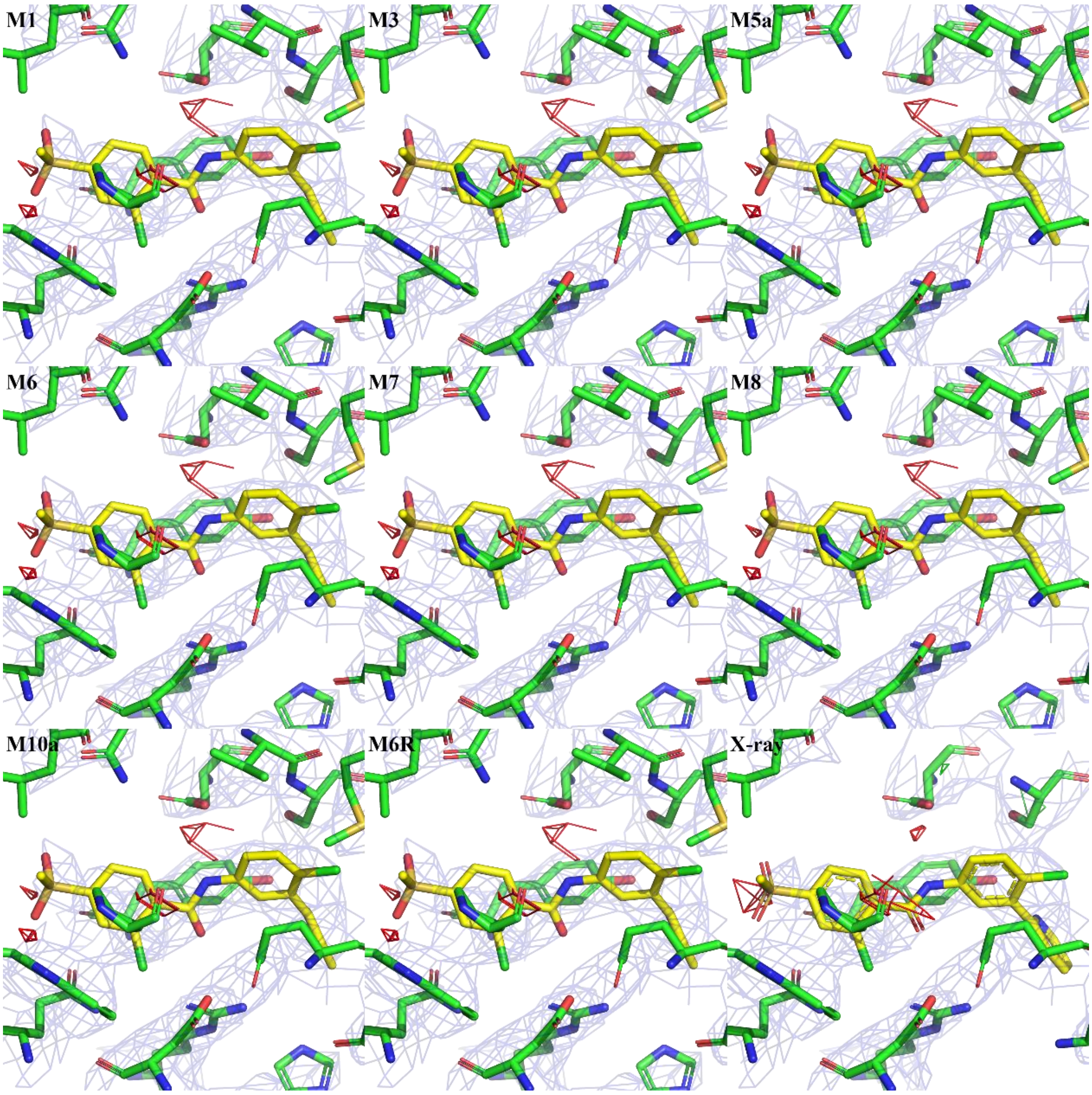

Supplementary Figure 281: Electron density maps of vismodegib (chain B). Structures vismodegib in human smoothened (chain B) from various quantum refinement schemes (**M1**-**M10**), including the electron density maps (2mFo-DFc maps, contoured at 1.0 σ (blue), mFo-DFc maps, contoured at +3.0 σ (green), and mFo-DFc maps, contoured at -3.0 σ (red)). Those results for X-ray were taken from the experimental structure without our further refinement.

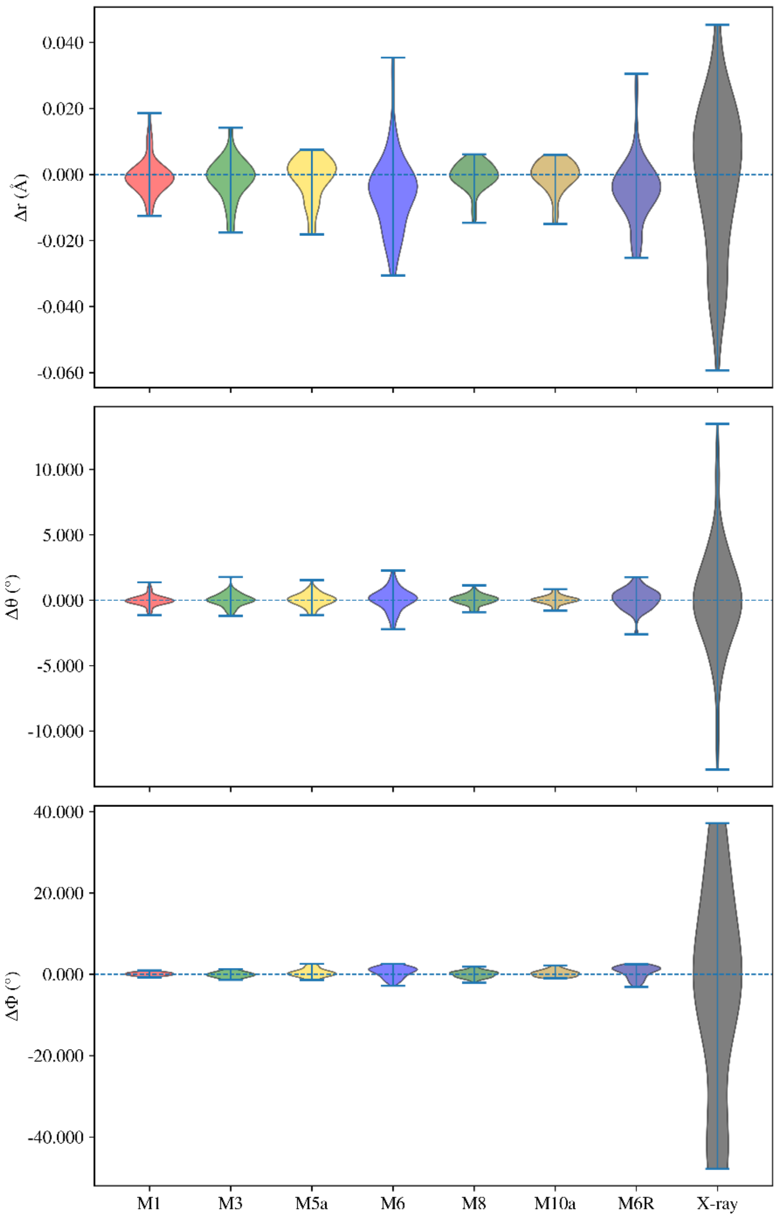

Supplementary Figure 282: Key coordinates of quantum refinement results of vismodegib. Deviation in the refined bond distances (Δr, n = 58), angles (Δθ, n = 84) and dihedrals (Δϕ, n = 36) of vismodegib in human smoothened from various quantum refinement schemes (**M1-M10**) and X-ray structure which are compared to those obtained from the most reliable **M7** scheme. The solid line represents the upper and lower values.
