# Supplementary material for: Accelerating reliable multiscale quantum refinement of protein–drug systems enabled by machine learning
Source: Nat Commun. 2024 May 16;15:4181. doi: 10.1038/s41467-024-48453-4 (PMC11099068; doi:10.1038/s41467-024-48453-4)
Supplement: Supplementary file 3 — Description of Additional Supplementary Files [file 41467_2024_48453_MOESM3_ESM.pdf]

Description of Additional Supplementary Files

File Name: Supplementary Data

Description: Cartesian coordinates of optimized structures in the gas phase and in proteins for QR50 dataset.
